# Supplementary material for: An AI Agent for Fully Automated Multi‐Omic Analyses
Source: Adv Sci (Weinh). 2024 Oct 3;11(44):2407094. doi: 10.1002/advs.202407094 (PMC11600294; doi:10.1002/advs.202407094)
Supplement: Supplementary file 1 — Supporting Information [file ADVS-11-2407094-s002.pdf]

ADVANCED SCIENCE
Open Access

Supporting Information

for *Adv. Sci.*, DOI 10.1002/advs.202407094

An AI Agent for Fully Automated Multi-Omic Analyses

Juexiao Zhou, Bin Zhang, Guowei Li, Xiuying Chen, Haoyang Li, Xiaopeng Xu, Siyuan Chen, Wenjia He, Chencheng Xu, Liwei Liu and Xin Gao**

Supplementary Information of An AI Agent for Fully Automated Multi-omic Analyses

Juexiao Zhou[1,2,†], Bin Zhang[1,2,†], Guowei Li[4], Xiuying Chen[1,2], Haoyang Li[1,2], Xiaopeng Xu[1,2], Siyuan Chen[1,2], Wenjia He[1,2], Chencheng Xu[1,2], Liwei Liu[3,*], Xin Gao[1,2,*]

[1] Computer Science Program, Computer, Electrical and Mathematical Sciences and Engineering Division, King Abdullah University of Science and Technology (KAUST), Thuwal 23955-6900, Kingdom of Saudi Arabia
[2] Computational Bioscience Research Center, King Abdullah University of Science and Technology (KAUST), Thuwal 23955-6900, Kingdom of Saudi Arabia
[3] Advanced Computing and Storage Laboratory, Central Research Institute, 2012 Laboratories. Huawei Technologies Co., Ltd., Nanjing 210000, Jiangsu, China
[4] Laboratory of Health Intelligence, Huawei Technologies Co., Ltd., Shenzhen, China
† These authors contributed equally.
* To whom correspondence should be addressed; E-mail: xin.gao@kaust.edu.sa.

Content Outline

1. Prompt design for planning phase of AutoBA 11

2. Prompt design for code generation phase of AutoBA 12

3. Prompt design for directly using other LLMs 13

4. Legend of Supplementary Video 14

5. Results of AutoBA (online version) 15

WGS data analysis Case 1.1: Genome assembly 15

WGS/WES data analysis Case 2.1 and Case 2.2 : Somatic SNV+indel calling and annotation 17

WGS/WES data analysis Case 2.3: Structure variation identification with normal 21

WGS/WES data analysis Case 2.4: Structure variation identification without normal 26

ChIP-Seq Analysis Case 3.1: Call Peaks 28

ChIP-Seq Analysis Case 3.2: Motif Discovery 31

ChIP-Seq Analysis Case 3.3: Functional Enrichment 33

Bisulfite-Seq data analysis Case 4.1: Identifying DNA methylation 36

ATAC-seq data analysis Case 5.1: Identifying open chromatin regions 39

DNase-seq data analysis Case 6.1: Identifying Dnasel hypersensitive site 41

4C-seq data analysis Case 7.1: Find genomics interactions 43

Nanopore DNA sequencing data analysis Case 8.1: Genome assembly 45

Nanopore DNA sequencing data analysis Case 8.2: Tandem repeats variation identification 48

PacBio DNA sequencing data analysis Case 9.1: Genome assembly 50

RNA-Seq Analysis Case 10.1: Find differentially expressed genes 52

RNA-Seq Analysis Case 10.2: Identify top5 down-regulated genes in HiGlu group 55

RNA-Seq Analysis Case 10.3: Predict Fusion genes 58

RNA-Seq Analysis Case 10.4: isoform expression 60

RNA-seq Analysis Case 10.5: splicing analysis 62

RNA-seq Analysis Case 10.6: APA analysis 66

RNA-seq Analysis Case 10.7: RNA editing ... 69

RNA-seq Analysis Case 10.8: circular RNA analysis ... 73

Small RNA-seq Analysis Case 11.1 and Case 11.2: miRNA analysis ... 76

CAGE-seq Analysis Case 12.1: TSS analysis ... 80

3'end-seq Analysis Case 13.1: APA analysis based on 3'end-seq data ... 83

ONT sequencing Analysis Case 14.1: isoform expression ... 86

PacBio iso-seq Analysis Case 15.1: isoform analysis ... 89

CLIP-seq Analysis Case 16.1: CLIP-seq data analysis ... 91

RIP-Seq Analysis Case 16.2: Identify enriched genes bounded by RPB ... 94

Ribo-Seq Analysis Case 17.1: Identify translated ORFs ... 97

scRNA-Seq Analysis Case 18.1: Cluster cell into groups based on raw fastq files ... 100

scRNA-Seq Analysis Case 18.2: Clustering and Find the differentially expressed genes ... 102

scRNA-Seq Analysis Case 18.3: Identify Marker Genes ... 104

scRNA-Seq Analysis Case 18.4: Clustering and Visualization ... 105

Spatial Transcriptomics Case 19.1: Neighborhood enrichment analysis ... 106

Spatial Transcriptomics Case 19.2: Single-cell mapping ... 107

Proteomics Case 20.1: protein expression quantification ... 110

Metabolomics Case 21.1: metabolites concentration ... 115

6. Results of AutoBA (local version) ... 119

WGS data analysis Case 1.1: Genome assembly ... 119

WGS/WES data analysis Case 2.1 and Case 2.2 : Somatic SNV+indel calling and annotation ... 121

WGS/WES data analysis Case 2.3: Structure variation identification with normal ... 123

WGS/WES data analysis Case 2.4: Structure variation identification without normal ... 125

ChIP-Seq Analysis Case 3.1: Call Peaks ... 127

ChIP-Seq Analysis Case 3.2: Motif Discovery ... 129

ChIP-Seq Analysis Case 3.3: Functional Enrichment ... 131

Bisulfite-Seq data analysis Case 4.1: Identifying DNA methylation ... 133

ATAC-seq data analysis Case 5.1: Identifying open chromatin regions 135

DNase-seq data analysis Case 6.1: Identifying Dnasel hypersensitive site 137

4C-seq data analysis Case 7.1: Find genomics interactions 139

Nanopore DNA sequencing data analysis Case 8.1: Genome assembly 140

Nanopore DNA sequencing data analysis Case 8.2: Tandem repeats variation identification 141

PacBio DNA sequencing data analysis Case 9.1: Genome assembly 143

RNA-Seq Analysis Case 10.1: Find differentially expressed genes 145

RNA-Seq Analysis Case 10.2: Identify top5 down-regulated genes in HiGlu group 147

RNA-Seq Analysis Case 10.3: Predict Fusion genes 149

RNA-Seq Analysis Case 10.4: isoform expression 150

RNA-seq Analysis Case 10.5: splicing analysis 152

RNA-seq Analysis Case 10.6: APA analysis 154

RNA-seq Analysis Case 10.7: RNA editing 156

RNA-seq Analysis Case 10.8: circular RNA analysis 158

Small RNA-seq Analysis Case 11.1 and Case 11.2: miRNA analysis 160

CAGE-seq Analysis Case 12.1: TSS analysis 162

3'end-seq Analysis Case 13.1: APA analysis based on 3'end-seq data 164

ONT sequencing Analysis Case 14.1: isoform expression 167

PacBio iso-seq Analysis Case 15.1: isoform analysis 168

CLIP-seq Analysis Case 16.1: CLIP-seq data analysis 170

RIP-Seq Analysis Case 16.2: Identify enriched genes bounded by RPB 171

Ribo-Seq Analysis Case 17.1: Identify translated ORFs 173

scRNA-Seq Analysis Case 18.1: Cluster cell into groups based on raw fastq files 175

scRNA-Seq Analysis Case 18.2: Clustering and Find the differentially expressed genes 176

scRNA-Seq Analysis Case 18.3: Identify Marker Genes 177

scRNA-Seq Analysis Case 18.4: Clustering and Visualization 178

Spatial Transcriptomics Case 19.1: Neighborhood enrichment analysis 179

Spatial Transcriptomics Case 19.2: Single-cell mapping 180

Proteomics Case 20.1: protein expression quantification 182

Metabolomics Case 21.1: metabolites concentration 184

7. Results of AutoGPT 186

WGS data analysis Case 1.1: Genome assembly 186

WGS/WES data analysis Case 2.1 and Case 2.2 : Somatic SNV+indel calling and annotation 189

WGS/WES data analysis Case 2.3: Structure variation identification with normal 191

WGS/WES data analysis Case 2.4: Structure variation identification without normal 193

ChIP-Seq Analysis Case 3.1: Call Peaks 199

ChIP-Seq Analysis Case 3.2: Motif Discovery 201

ChIP-Seq Analysis Case 3.3: Functional Enrichment 211

Bisulfite-Seq data analysis Case 4.1: Identifying DNA methylation 213

ATAC-seq data analysis Case 5.1: Identifying open chromatin regions 223

DNase-seq data analysis Case 6.1: Identifying DnaseI hypersensitive site 225

4C-seq data analysis Case 7.1: Find genomics interactions 227

Nanopore DNA sequencing data analysis Case 8.1: Genome assembly 229

Nanopore DNA sequencing data analysis Case 8.2: Tandem repeats variation identification 231

PacBio DNA sequencing data analysis Case 9.1: Genome assembly 233

RNA-Seq Analysis Case 10.1: Find differentially expressed genes 234

RNA-Seq Analysis Case 10.2: Identify top5 down-regulated genes in HiGlu group 236

RNA-Seq Analysis Case 10.3: Predict Fusion genes 237

RNA-Seq Analysis Case 10.4: isoform expression 243

RNA-seq Analysis Case 10.5: splicing analysis 245

RNA-seq Analysis Case 10.6: APA analysis 247

RNA-seq Analysis Case 10.7: RNA editing 249

RNA-seq Analysis Case 10.8: circular RNA analysis 259

Small RNA-seq Analysis Case 11.1 and Case 11.2: miRNA analysis 262

CAGE-seq Analysis Case 12.1: TSS analysis....264
3'end-seq Analysis Case 13.1: APA analysis based on 3'end-seq data....266
ONT sequencing Analysis Case 14.1: isoform expression....268
PacBio iso-seq Analysis Case 15.1: isoform analysis....270
CLIP-seq Analysis Case 16.1: CLIP-seq data analysis....273
RIP-Seq Analysis Case 16.2: Identify enriched genes bounded by RPB....275
Ribo-Seq Analysis Case 17.1: Identify translated ORFs....277
scRNA-Seq Analysis Case 18.1: Cluster cell into groups based on raw fastq files....279
scRNA-Seq Analysis Case 18.2: Clustering and Find the differentially expressed genes....281
scRNA-Seq Analysis Case 18.3: Identify Marker Genes....285
scRNA-Seq Analysis Case 18.4: Clustering and Visualization....295
Spatial Transcriptomics Case 19.1: Neighborhood enrichment analysis....296
Spatial Transcriptomics Case 19.2: Single-cell mapping....301
Proteomics Case 20.1: protein expression quantification....303
Metabolomics Case 21.1: metabolites concentration....313
8. Results of ChatGPT-3.5....326
WGS data analysis Case 1.1: Genome assembly....326
WGS/WES data analysis Case 2.1 and Case 2.2 : Somatic SNV+indel calling and annotation....328
WGS/WES data analysis Case 2.3: Structure variation identification with normal....331
WGS/WES data analysis Case 2.4: Structure variation identification without normal....333
ChIP-Seq Analysis Case 3.1: Call Peaks....335
ChIP-Seq Analysis Case 3.2: Motif Discovery....337
ChIP-Seq Analysis Case 3.3: Functional Enrichment....339
Bisulfite-Seq data analysis Case 4.1: Identifying DNA methylation....341
ATAC-seq data analysis Case 5.1: Identifying open chromatin regions....343
DNase-seq data analysis Case 6.1: Identifying DnaseI hypersensitive site....345
4C-seq data analysis Case 7.1: Find genomics interactions....347

Nanopore DNA sequencing data analysis Case 8.1: Genome assembly 349

Nanopore DNA sequencing data analysis Case 8.2: Tandem repeats variation identification 351

PacBio DNA sequencing data analysis Case 9.1: Genome assembly 352

RNA-Seq Analysis Case 10.1: Find differentially expressed genes 354

RNA-Seq Analysis Case 10.2: Identify top5 down-regulated genes in HiGlu group 356

RNA-Seq Analysis Case 10.3: Predict Fusion genes 359

RNA-Seq Analysis Case 10.4: isoform expression 361

RNA-seq Analysis Case 10.5: splicing analysis 364

RNA-seq Analysis Case 10.6: APA analysis 366

RNA-seq Analysis Case 10.7: RNA editing 369

RNA-seq Analysis Case 10.8: circular RNA analysis 372

Small RNA-seq Analysis Case 11.1 and Case 11.2: miRNA analysis 375

CAGE-seq Analysis Case 12.1: TSS analysis 377

3'end-seq Analysis Case 13.1: APA analysis based on 3'end-seq data 379

ONT sequencing Analysis Case 14.1: isoform expression 381

PacBio iso-seq Analysis Case 15.1: isoform analysis 383

CLIP-seq Analysis Case 16.1: CLIP-seq data analysis 385

RIP-Seq Analysis Case 16.2: Identify enriched genes bounded by RPB 387

Ribo-Seq Analysis Case 17.1: Identify translated ORFs 389

scRNA-Seq Analysis Case 18.1: Cluster cell into groups based on raw fastq files 391

scRNA-Seq Analysis Case 18.2: Clustering and Find the differentially expressed genes 393

scRNA-Seq Analysis Case 18.3: Identify Marker Genes 395

scRNA-Seq Analysis Case 18.4: Clustering and Visualization 397

Spatial Transcriptomics Case 19.1: Neighborhood enrichment analysis 399

Spatial Transcriptomics Case 19.2: Single-cell mapping 401

Proteomics Case 20.1: protein expression quantification 403

Metabolomics Case 21.1: metabolites concentration 405

9. Results of ChatGPT-4 407
WGS data analysis Case 1.1: Genome assembly 407
WGS/WES data analysis Case 2.1 and Case 2.2 : Somatic SNV+indel calling and annotation 409
WGS/WES data analysis Case 2.3: Structure variation identification with normal 413
WGS/WES data analysis Case 2.4: Structure variation identification without normal 416
ChIP-Seq Analysis Case 3.1: Call Peaks 419
ChIP-Seq Analysis Case 3.2: Motif Discovery 421
ChIP-Seq Analysis Case 3.3: Functional Enrichment 423
Bisulfite-Seq data analysis Case 4.1: Identifying DNA methylation 425
ATAC-seq data analysis Case 5.1: Identifying open chromatin regions 427
DNase-seq data analysis Case 6.1: Identifying Dnasel hypersensitive site 429
4C-seq data analysis Case 7.1: Find genomics interactions 431
Nanopore DNA sequencing data analysis Case 8.1: Genome assembly 433
Nanopore DNA sequencing data analysis Case 8.2: Tandem repeats variation identification 435
PacBio DNA sequencing data analysis Case 9.1: Genome assembly 437
RNA-Seq Analysis Case 10.1: Find differentially expressed genes 439
RNA-Seq Analysis Case 10.2: Identify top5 down-regulated genes in HiGlu group 442
RNA-Seq Analysis Case 10.3: Predict Fusion genes 445
RNA-Seq Analysis Case 10.4: isoform expression 447
RNA-seq Analysis Case 10.5: splicing analysis 449
RNA-seq Analysis Case 10.6: APA analysis 452
RNA-seq Analysis Case 10.7: RNA editing 455
RNA-seq Analysis Case 10.8: circular RNA analysis 458
Small RNA-seq Analysis Case 11.1 and Case 11.2: miRNA analysis 461
CAGE-seq Analysis Case 12.1: TSS analysis 464
3'end-seq Analysis Case 13.1: APA analysis based on 3'end-seq data 467
ONT sequencing Analysis Case 14.1: isoform expression 470

PacBio iso-seq Analysis Case 15.1: isoform analysis 473

CLIP-seq Analysis Case 16.1: CLIP-seq data analysis 476

RIP-Seq Analysis Case 16.2: Identify enriched genes bounded by RPB 479

Ribo-Seq Analysis Case 17.1: Identify translated ORFs 483

scRNA-Seq Analysis Case 18.1: Cluster cell into groups based on raw fastq files 486

scRNA-Seq Analysis Case 18.2: Clustering and Find the differentially expressed genes 489

scRNA-Seq Analysis Case 18.3: Identify Marker Genes 491

scRNA-Seq Analysis Case 18.4: Clustering and Visualization 493

Spatial Transcriptomics Case 19.1: Neighborhood enrichment analysis 495

Spatial Transcriptomics Case 19.2: Single-cell mapping 497

Proteomics Case 20.1: protein expression quantification 499

Metabolomics Case 21.1: metabolites concentration 502

10. Results of CodeLlama-34B-Instruct 507

WGS data analysis Case 1.1: Genome assembly 507

WGS/WES data analysis Case 2.1 and Case 2.2 : Somatic SNV+indel calling and annotation 510

WGS/WES data analysis Case 2.3: Structure variation identification with normal 512

WGS/WES data analysis Case 2.4: Structure variation identification without normal 515

ChIP-Seq Analysis Case 3.1: Call Peaks 517

ChIP-Seq Analysis Case 3.2: Motif Discovery 519

ChIP-Seq Analysis Case 3.3: Functional Enrichment 522

Bisulfite-Seq data analysis Case 4.1: Identifying DNA methylation 524

ATAC-seq data analysis Case 5.1: Identifying open chromatin regions 527

DNase-seq data analysis Case 6.1: Identifying DnaseI hypersensitive site 529

4C-seq data analysis Case 7.1: Find genomics interactions 532

Nanopore DNA sequencing data analysis Case 8.1: Genome assembly 534

Nanopore DNA sequencing data analysis Case 8.2: Tandem repeats variation identification 536

PacBio DNA sequencing data analysis Case 9.1: Genome assembly 539

RNA-Seq Analysis Case 10.1: Find differentially expressed genes....541

RNA-Seq Analysis Case 10.2: Identify top5 down-regulated genes in HiGlu group....543

RNA-Seq Analysis Case 10.3: Predict Fusion genes....545

RNA-Seq Analysis Case 10.4: isoform expression....547

RNA-seq Analysis Case 10.5: splicing analysis....550

RNA-seq Analysis Case 10.6: APA analysis....553

RNA-seq Analysis Case 10.7: RNA editing....556

RNA-seq Analysis Case 10.8: circular RNA analysis....559

Small RNA-seq Analysis Case 11.1 and Case 11.2: miRNA analysis....563

CAGE-seq Analysis Case 12.1: TSS analysis....565

3'end-seq Analysis Case 13.1: APA analysis based on 3'end-seq data....568

ONT sequencing Analysis Case 14.1: isoform expression....572

PacBio iso-seq Analysis Case 15.1: isoform analysis....576

CLIP-seq Analysis Case 16.1: CLIP-seq data analysis....578

RIP-Seq Analysis Case 16.2: Identify enriched genes bounded by RPB....581

Ribo-Seq Analysis Case 17.1: Identify translated ORFs....584

scRNA-Seq Analysis Case 18.1: Cluster cell into groups based on raw fastq files....586

scRNA-Seq Analysis Case 18.2: Clustering and Find the differentially expressed genes....588

scRNA-Seq Analysis Case 18.3: Identify Marker Genes....590

scRNA-Seq Analysis Case 18.4: Clustering and Visualization....591

Spatial Transcriptomics Case 19.1: Neighborhood enrichment analysis....593

Spatial Transcriptomics Case 19.2: Single-cell mapping....595

Proteomics Case 20.1: protein expression quantification....597

Metabolomics Case 21.1: metabolites concentration....600

1. Prompt design for planning phase of AutoBA

```
prompt = {
        "role": "Act as a bioinformatician, the rules must be strictly
followed!",
        "rules": [
            "When acting as a bioinformatician, you strictly cannot stop
acting as a bioinformatician.",
            "All rules must be followed strictly.",
            "You should use information in input to write a detailed plan
to finish your goal.",
            f"You should include the software name and should not use those
software: {self.blacklist}.",
            "You should only respond in JSON format with my fixed format.",
            "Your JSON response should only be enclosed in double quotes.",
            "You should not write loading data as a separate step.",
            "You should not write anything else except for your JSON
response.",
            "You should make your answer as detailed as possible."
        ],
        "input": [
                "You have the following information in a list with the
format file path: file description. I provide those files to you, so you
don't need to prepare the data.",
                data_list
            ],
        "goal": self.current_goal,
        "fixed format for JSON response": {
            "plan": [
                "Your detailed step-by-step sub-tasks in a list to finish
your goal, for example: ['step 1: content', 'step 2: content', 'step 3:
content']."
            ]
        }
    }
```

2. Prompt design for code generation phase of AutoBA

```
prompt = {
    "role": "Act as a bioinformatician, the rules must be strictly followed!",
    "rules": [
        "When acting as a bioinformatician, you strictly cannot stop acting as a bioinformatician.",
        "All rules must be followed strictly.",
        "You are provided a system with specified constraints."
        "The history of what you have done is provided, you should take the name changes of some files into account, or use some output from previous steps.",
        "You should use all information you have to write bash codes to finish your current task.",
        "All code requirements must be followed strictly when you write codes.",
        "You should only respond in JSON format with my fixed format.",
        "Your JSON response should only be enclosed in double quotes.",
        "You should make your answer as simple as possible.",
        "You should not write anything else except for your JSON response."
    ],
    "system": [
        "You have a Ubuntu 22.04 system",
        "You have a conda environment named abc_runtime",
        "You do not have any other software installed"
    ],
    "input": [
            "You have the following information in a list with the format file path: file description. I provide those files to you, so you don't need to prepare the data.",
            data_list
        ],
    "history": self.history_summary,
    "current task": self.current_goal,
    "code requirement": [
        f"You should not use that software: {self.blacklist}.",
        "You don't need to create and activate the conda environment abc_runtime.",
        'You should always add conda-forge and bioconda to the list of channels',
        'You should always install dependencies and software you need to use with conda or pip with -y.',
        'You should pay attention to the number of input files and do not miss any.',
        'You should process each file independently and can not use FOR loop.',
        'You should use the path for all files according to input and history.',
        'You should use the default values for all parameters that are not specified.',
        'You should not repeat what you have done in history.',
        'You should only use software directly you installed with conda or pip.',
        'If you use Rscript -e, you should make sure all variables exist in your command, otherwise, you need to check your history to repeat previous steps and generate those variables.',
        "You should not write anything else except for your JSON response."
    ],
    "fixed format for JSON response": {
        "tool": "name of the tool you use",
        "code": "bash code to finish the current task"
    }
}
```

3. Prompt design for directly using other LLMs

```
prompt = {
    You are a bioinformatician who specializes in writing code and performing
analysis for a variety of bioinformatic data analysis tasks. Now I provide you
with an Ubuntu 18.04 system where I save the following data, each line
including the data path and data description.

<file path: file description>

I need you to help me plan what needs to be done in each step of the analysis
process, and help me write the code for each step to install softwares, use
tools and accomplish the following main goal:

<goal>
}
```

4. Legend of Supplementary Video

Video S1. AutoBA running demo.

5. Results of AutoBA (online version)

WGS data analysis Case 1.1: Genome assembly

<table>
<tr><td>User's Inputs</td><td>Data and Description
SRR1234567_R1.fastq.gz: Paired-end Illumina WGS reads, forward
SRR1234567_R2.fastq.gz: Paired-end Illumina WGS reads, reverse
TruSeq3-PE.fa: Adapter sequences for trimming
Reference.fasta: Reference genome for the organism under study in FASTA format

Goal
To perform genome assembly using paired-end WGS data</td></tr>
<tr><td>Generated Plans by AutoBA</td><td>1. Validate the quality of the raw reads using FastQC. This will provide a quality report for the raw sequencing data. Command: 'fastqc ./examples/case1.1/data/SRR1234567_R1.fastq.gz ./examples/case1.1/data/SRR1234567_R2.fastq.gz'
2. Trim the adapters and low-quality bases from the raw reads using Trimmomatic. This will improve the quality of the reads and remove any adapter contamination. Command: 'trimmomatic PE ./examples/case1.1/data/SRR1234567_R1.fastq.gz ./examples/case1.1/data/SRR1234567_R2.fastq.gz trimmed_R1.fastq.gz trimmed_R2.fastq.gz ILLUMINACLIP:./examples/case1.1/data/TruSeq3-PE.fa:2:30:10 LEADING:3 TRAILING:3 SLIDINGWINDOW:4:15 MINLEN:36'
3. Validate the quality of the trimmed reads using FastQC. This will provide a quality report for the trimmed sequencing data. Command: 'fastqc trimmed_R1.fastq.gz trimmed_R2.fastq.gz'
4. Assemble the genome using SPAdes. This will generate a draft genome assembly from the trimmed reads. Command: 'spades.py -1 trimmed_R1.fastq.gz -2 trimmed_R2.fastq.gz -o assembly'
5. Validate the quality of the assembly using QUAST. This will provide a quality report for the draft genome assembly. Command: 'quast.py assembly/contigs.fasta -r ./examples/case1.1/data/Reference.fasta -o quast_report'</td></tr>
<tr><td>Generated Codes by AutoBA</td><td># Step 1
#!/bin/bash

Activate the conda environment
source activate abc

Install FastQC with conda
conda install -y fastqc

Validate the quality of the raw reads using FastQC
fastqc ./examples/case1.1/data/SRR1234567_R1.fastq.gz
fastqc ./examples/case1.1/data/SRR1234567_R2.fastq.gz

Step 2
#!/bin/bash

Activate the conda environment
source activate abc</td></tr>
</table>

	# Install Trimmomatic with conda<br>conda install -y trimmomatic<br><br># Trim the adapters and low-quality bases from the raw reads using Trimmomatic<br>trimmomatic<br>PE ./examples/case1.1/data/SRR1234567_R1.fastq.gz ./examples/case1.1/data/SRR1234567_R2.fastq.gz ./examples/case1.1/output/trimmed_R1.fastq.gz ./examples/case1.1/output/trimmed_R2.fastq.gz<br>ILLUMINACLIP:./examples/case1.1/data/TruSeq3-PE.fa:2:30:10<br>LEADING:3 TRAILING:3 SLIDINGWINDOW:4:15 MINLEN:36<br><br>**# Step 3**<br>#!/bin/bash<br><br># Activate the conda environment<br>source activate abc<br><br># Install FastQC with conda<br>conda install -y fastqc<br><br># Validate the quality of the trimmed reads using FastQC<br>fastqc ./examples/case1.1/output/trimmed_R1.fastq.gz<br>fastqc ./examples/case1.1/output/trimmed_R2.fastq.gz<br><br>**# Step 4**<br>#!/bin/bash<br><br># Activate the conda environment<br>source activate abc<br><br># Install SPAdes with conda<br>conda install -y spades<br><br># Assemble the genome using SPAdes<br>spades -1 ./examples/case1.1/output/trimmed_R1.fastq.gz -<br>2 ./examples/case1.1/output/trimmed_R2.fastq.gz -<br>o ./examples/case1.1/output/assembly<br><br>**# Step 5**<br>#!/bin/bash<br><br># Activate the conda environment<br>source activate abc<br><br># Install QUAST with conda<br>conda install -y quast<br><br># Validate the quality of the assembly using QUAST<br>quast ./examples/case1.1/output/assembly/contigs.fasta -<br>r ./examples/case1.1/data/Reference.fasta -<br>o ./examples/case1.1/output/quast_report

WGS/WES data analysis **Case 2.1 and Case 2.2 : Somatic SNV+indel calling and annotation**

**User's Inputs**	**Data and Description**<br>SRR23015874_R1.fastq.gz: paired end read1 from human whole genome sequencing, normal samples replicate 1<br>SRR23015874_R2.fastq.gz: paired end read2 from human whole genome sequencing, normal samples replicate 1<br>SRR23015876_R1.fastq.gz: paired end read1 from human whole genome sequencing, tumor samples replicate 1<br>SRR23015876_R2.fastq.gz: paired end read2 from human whole genome sequencing, tumor samples replicate 1<br>SRR23015877_R1.fastq.gz: paired end read1 from human whole genome sequencing, tumor samples replicate 2<br>SRR23015877_R2.fastq.gz: paired end read2 from human whole genome sequencing, tumor samples replicate 2<br>TruSeq3-PE.fa: trimming adapter<br>hg38.fa: human hg38 reference genome fasta<br><br>**Goal**<br>call somatic mutations in tumor samples over the matched normal samples and annotate the identified mutations
**Generated Plans by AutoBA**	1. First, we will use the software 'FastQC' to perform quality control checks on the raw sequence data. The input files for this step will be './experiments/case2.2/data/SRR23015874_R1.fastq.gz', './experiments/case2.2/data/SRR23015874_R2.fastq.gz', './experiments/case2.2/data/SRR23015876_R1.fastq.gz', './experiments/case2.2/data/SRR23015876_R2.fastq.gz', './experiments/case2.2/data/SRR23015877_R1.fastq.gz', and './experiments/case2.2/data/SRR23015877_R2.fastq.gz'.<br>2. Next, we will use 'Trimmomatic' to trim the low-quality bases and adapter sequences from the raw data. The input files for this step will be the same as in step 1, along with the adapter sequence file './experiments/case2.2/data/TruSeq3-PE.fa'.<br>3. After trimming, we will align the reads to the human reference genome using 'BWA'. The input files for this step will be the output files from step 2 and the reference genome file './experiments/case2.2/data/hg38.fa'.<br>4. The aligned reads will then be sorted and duplicates will be marked using 'Picard'. The input file for this step will be the output file from step 3.<br>5. We will then use 'GATK' to perform local realignment around indels and base quality score recalibration. The input file for this step will be the output file from step 4.<br>6. Next, we will use 'MuTect2' to call somatic mutations in the tumor samples over the matched normal samples. The input files for this step will be the output files from step 5.<br>7. Finally, we will use 'VEP' to annotate the identified mutations. The input file for this step will be the output file from step 6.
**Generated Codes by AutoBA**	**# step 1**<br>source activate abc<br>conda config --add channels conda-forge<br>conda config --add channels bioconda<br>conda install -y fastqc<br>fastqc ./experiments/case2.2/data/SRR23015874_R1.fastq.gz -o ./experiments/case2.2/output

	fastqc ./experiments/case2.2/data/SRR23015874_R2.fastq.gz -o ./experiments/case2.2/output<br>fastqc ./experiments/case2.2/data/SRR23015876_R1.fastq.gz -o ./experiments/case2.2/output<br>fastqc ./experiments/case2.2/data/SRR23015876_R2.fastq.gz -o ./experiments/case2.2/output<br>fastqc ./experiments/case2.2/data/SRR23015877_R1.fastq.gz -o ./experiments/case2.2/output<br>fastqc ./experiments/case2.2/data/SRR23015877_R2.fastq.gz -o ./experiments/case2.2/output<br><br>**# step 2**<br>source activate abc<br>conda config --add channels conda-forge<br>conda config --add channels bioconda<br>conda install -y trimmomatic<br>trimmomatic PE -phred33 ./experiments/case2.2/data/SRR23015874_R1.fastq.gz ./experiments/case2.2/data/SRR23015874_R2.fastq.gz ./experiments/case2.2/output/SRR23015874_R1_paired.fastq.gz ./experiments/case2.2/output/SRR23015874_R1_unpaired.fastq.gz ./experiments/case2.2/output/SRR23015874_R2_paired.fastq.gz ./experiments/case2.2/output/SRR23015874_R2_unpaired.fastq.gz ILLUMINACLIP:./experiments/case2.2/data/TruSeq3-PE.fa:2:30:10<br>trimmomatic PE -phred33 ./experiments/case2.2/data/SRR23015876_R1.fastq.gz ./experiments/case2.2/data/SRR23015876_R2.fastq.gz ./experiments/case2.2/output/SRR23015876_R1_paired.fastq.gz ./experiments/case2.2/output/SRR23015876_R1_unpaired.fastq.gz ./experiments/case2.2/output/SRR23015876_R2_paired.fastq.gz ./experiments/case2.2/output/SRR23015876_R2_unpaired.fastq.gz ILLUMINACLIP:./experiments/case2.2/data/TruSeq3-PE.fa:2:30:10<br>trimmomatic PE -phred33 ./experiments/case2.2/data/SRR23015877_R1.fastq.gz ./experiments/case2.2/data/SRR23015877_R2.fastq.gz ./experiments/case2.2/output/SRR23015877_R1_paired.fastq.gz ./experiments/case2.2/output/SRR23015877_R1_unpaired.fastq.gz ./experiments/case2.2/output/SRR23015877_R2_paired.fastq.gz ./experiments/case2.2/output/SRR23015877_R2_unpaired.fastq.gz ILLUMINACLIP:./experiments/case2.2/data/TruSeq3-PE.fa:2:30:10<br><br>**# step 3**<br>source activate abc<br>conda config --add channels conda-forge<br>conda config --add channels bioconda<br>conda install -y bwa<br>bwa index ./experiments/case2.2/data/hg38.fa<br>bwa mem ./experiments/case2.2/data/hg38.fa ./experiments/case2.2/output/SRR23015874_R1_paired.fastq.gz ./experiments/case2.2/output/SRR23015874_R2_paired.fastq.gz > ./experiments/case2.2/output/SRR23015874.sam<br>bwa mem ./experiments/case2.2/data/hg38.fa ./experiments/case2.2/output/SRR

	23015876_R1_paired.fastq.gz ./experiments/case2.2/output/SRR23015876_R2_paired.fastq.gz > ./experiments/case2.2/output/SRR23015876.sam<br>bwa<br>mem ./experiments/case2.2/data/hg38.fa ./experiments/case2.2/output/SRR23015877_R1_paired.fastq.gz ./experiments/case2.2/output/SRR23015877_R2_paired.fastq.gz > ./experiments/case2.2/output/SRR23015877.sam<br><br>**#step 4**<br>source activate abc<br>conda config --add channels conda-forge<br>conda config --add channels bioconda<br>conda install -y picard<br>picard SortSam I=./experiments/case2.2/output/SRR23015874.sam<br>O=./experiments/case2.2/output/SRR23015874_sorted.bam<br>SORT_ORDER=coordinate<br>picard MarkDuplicates<br>I=./experiments/case2.2/output/SRR23015874_sorted.bam<br>O=./experiments/case2.2/output/SRR23015874_sorted_dedup.bam<br>M=./experiments/case2.2/output/SRR23015874_sorted_dedup.metrics<br>picard SortSam I=./experiments/case2.2/output/SRR23015876.sam<br>O=./experiments/case2.2/output/SRR23015876_sorted.bam<br>SORT_ORDER=coordinate<br>picard MarkDuplicates<br>I=./experiments/case2.2/output/SRR23015876_sorted.bam<br>O=./experiments/case2.2/output/SRR23015876_sorted_dedup.bam<br>M=./experiments/case2.2/output/SRR23015876_sorted_dedup.metrics<br>picard SortSam I=./experiments/case2.2/output/SRR23015877.sam<br>O=./experiments/case2.2/output/SRR23015877_sorted.bam<br>SORT_ORDER=coordinate<br>picard MarkDuplicates<br>I=./experiments/case2.2/output/SRR23015877_sorted.bam<br>O=./experiments/case2.2/output/SRR23015877_sorted_dedup.bam<br>M=./experiments/case2.2/output/SRR23015877_sorted_dedup.metrics<br><br>**# step 5**<br>source activate abc<br>conda config --add channels conda-forge<br>conda config --add channels bioconda<br>conda install -y gatk<br><br># Perform local realignment around indels<br>gatk -T RealignerTargetCreator -R ./experiments/case2.2/data/hg38.fa -I ./experiments/case2.2/output/SRR23015874_sorted_dedup.bam -o ./experiments/case2.2/output/SRR23015874_realignment_targets.list<br><br># Apply the realignment to the BAM file<br>gatk -T IndelRealigner -R ./experiments/case2.2/data/hg38.fa -I ./experiments/case2.2/output/SRR23015874_sorted_dedup.bam -targetIntervals ./experiments/case2.2/output/SRR23015874_realignment_targets.list -o ./experiments/case2.2/output/SRR23015874_realigned.bam<br><br># Perform local realignment around indels

	gatk -T RealignerTargetCreator -R ./experiments/case2.2/data/hg38.fa -I ./experiments/case2.2/output/SRR23015876_sorted_dedup.bam -o ./experiments/case2.2/output/SRR23015876_realignment_targets.list<br><br># Apply the realignment to the BAM file<br>gatk -T IndelRealigner -R ./experiments/case2.2/data/hg38.fa -I ./experiments/case2.2/output/SRR23015876_sorted_dedup.bam -targetIntervals ./experiments/case2.2/output/SRR23015876_realignment_targets.list -o ./experiments/case2.2/output/SRR23015876_realigned.bam<br><br># Perform local realignment around indels<br>gatk -T RealignerTargetCreator -R ./experiments/case2.2/data/hg38.fa -I ./experiments/case2.2/output/SRR23015877_sorted_dedup.bam -o ./experiments/case2.2/output/SRR23015877_realignment_targets.list<br><br># Apply the realignment to the BAM file<br>gatk -T IndelRealigner -R ./experiments/case2.2/data/hg38.fa -I ./experiments/case2.2/output/SRR23015877_sorted_dedup.bam -targetIntervals ./experiments/case2.2/output/SRR23015877_realignment_targets.list -o ./experiments/case2.2/output/SRR23015877_realigned.bam<br><br>**# step 6**<br>source activate abc<br>conda config --add channels conda-forge<br>conda config --add channels bioconda<br>conda install -y gatk<br><br># Call somatic mutations in the tumor samples over the matched normal samples<br>gatk Mutect2 -R ./experiments/case2.2/data/hg38.fa -I ./experiments/case2.2/output/SRR23015874_realigned.bam -I ./experiments/case2.2/output/SRR23015876_realigned.bam -tumor SRR23015876 -normal SRR23015874 -O ./experiments/case2.2/output/SRR23015876_mutect2.vcf<br>gatk Mutect2 -R ./experiments/case2.2/data/hg38.fa -I ./experiments/case2.2/output/SRR23015874_realigned.bam -I ./experiments/case2.2/output/SRR23015877_realigned.bam -tumor SRR23015877 -normal SRR23015874 -O ./experiments/case2.2/output/SRR23015877_mutect2.vcf<br><br>**# step 7**<br>source activate abc<br>conda config --add channels conda-forge<br>conda config --add channels bioconda<br>conda install -y ensembl-vep<br>vep -i ./experiments/case2.2/output/SRR23015876_mutect2.vcf -o ./experiments/case2.2/output/SRR23015876_annotated.vcf<br>vep -i ./experiments/case2.2/output/SRR23015877_mutect2.vcf -o ./experiments/case2.2/output/SRR23015877_annotated.vcf

WGS/WES data analysis **Case 2.3: Structure variation identification with normal**

<table>
<tr><td>User's Inputs</td><td>Data and Description
SRR23015874_R1.fastq.gz: paired end read1 from human whole genome sequencing, the matched normal samples replicate 1
SRR23015874_R2.fastq.gz: paired end read2 from human whole genome sequencing, the matched normal samples replicate 1
SRR23015876_R1.fastq.gz: paired end read1 from human whole genome sequencing, tumor samples replicate 1
SRR23015876_R2.fastq.gz: paired end read2 from human whole genome sequencing, tumor samples replicate 1
SRR23015877_R1.fastq.gz: paired end read1 from human whole genome sequencing, tumor samples replicate 2
SRR23015877_R2.fastq.gz: paired end read2 from human whole genome sequencing, tumor samples replicate 2
TruSeq3-PE.fa: trimming adapter
hg38.fa: human hg38 reference genome fasta

Goal
To identify structural variations, which can include large insertions, deletions, inversions, duplications, and translocations, by comparing the sequencing data from the tumor sample over the matched normal sample</td></tr>
<tr><td>Generated Plans by AutoBA</td><td>1. First, we will use the 'FastQC' software to perform quality control checks on the raw sequence data.
2. Next, we will use the 'Trimmomatic' software to trim the Illumina adapters from the raw sequence data. The adapter file 'TruSeq3-PE.fa' will be used for this purpose.
3. After trimming, we will align the reads to the human reference genome 'hg38.fa' using the 'BWA-MEM' software. This will generate SAM files.
4. The SAM files will then be converted to BAM files using the 'SAMtools' software.
5. The BAM files will be sorted and indexed, again using 'SAMtools'.
6. We will then use the 'GATK' software to mark duplicates in the BAM files.
7. The 'GATK' software will also be used to perform local realignment around indels.
8. Base quality score recalibration (BQSR) will be performed using 'GATK'.
9. Finally, we will use the 'Manta' software to identify structural variations in the tumor sample compared to the matched normal sample. This will help us identify large insertions, deletions, inversions, duplications, and translocations.</td></tr>
<tr><td>Generated Codes by AutoBA</td><td># Step 1
source activate abc

Install FastQC
conda install -y fastqc

Perform quality control checks on raw sequence data
fastqc ./examples/case5.1/SRR23015874_R1.fastq.gz -o ../output/case2.3
fastqc ./examples/case5.1/SRR23015874_R2.fastq.gz -o ../output/case2.3
fastqc ./examples/case5.1/SRR23015876_R1.fastq.gz -o ../output/case2.3
fastqc ./examples/case5.1/SRR23015876_R2.fastq.gz -o ../output/case2.3
fastqc ./examples/case5.1/SRR23015877_R1.fastq.gz -o ../output/case2.3
fastqc ./examples/case5.1/SRR23015877_R2.fastq.gz -o ../output/case2.3</td></tr>
</table>

```
# Step 2
source activate abc

# Install Trimmomatic
conda install -y trimmomatic

# Trim the Illumina adapters from the raw sequence data
trimmomatic PE -
phred33 ./examples/case5.1/SRR23015874_R1.fastq.gz ./examples/case5.
1/SRR23015874_R2.fastq.gz ../output/case2.3/SRR23015874_R1_paired.f
astq.gz ../output/case2.3/SRR23015874_R1_unpaired.fastq.gz ../output/cas
e2.3/SRR23015874_R2_paired.fastq.gz ../output/case2.3/SRR23015874_R
2_unpaired.fastq.gz ILLUMINACLIP:./examples/case5.1/TruSeq3-
PE.fa:2:30:10
trimmomatic PE -
phred33 ./examples/case5.1/SRR23015876_R1.fastq.gz ./examples/case5.
1/SRR23015876_R2.fastq.gz ../output/case2.3/SRR23015876_R1_paired.f
astq.gz ../output/case2.3/SRR23015876_R1_unpaired.fastq.gz ../output/cas
e2.3/SRR23015876_R2_paired.fastq.gz ../output/case2.3/SRR23015876_R
2_unpaired.fastq.gz ILLUMINACLIP:./examples/case5.1/TruSeq3-
PE.fa:2:30:10
trimmomatic PE -
phred33 ./examples/case5.1/SRR23015877_R1.fastq.gz ./examples/case5.
1/SRR23015877_R2.fastq.gz ../output/case2.3/SRR23015877_R1_paired.f
astq.gz ../output/case2.3/SRR23015877_R1_unpaired.fastq.gz ../output/cas
e2.3/SRR23015877_R2_paired.fastq.gz ../output/case2.3/SRR23015877_R
2_unpaired.fastq.gz ILLUMINACLIP:./examples/case5.1/TruSeq3-
PE.fa:2:30:10

# Step 3
source activate abc

# Install BWA
conda install -y bwa

# Align the trimmed sequence reads to the human reference genome
bwa
mem ./examples/case5.1/hg38.fa ../output/case2.3/SRR23015874_R1_pair
ed.fastq.gz ../output/case2.3/SRR23015874_R2_paired.fastq.gz > ../output/
case2.3/SRR23015874.sam
bwa
mem ./examples/case5.1/hg38.fa ../output/case2.3/SRR23015876_R1_pair
ed.fastq.gz ../output/case2.3/SRR23015876_R2_paired.fastq.gz > ../output/
case2.3/SRR23015876.sam
bwa
mem ./examples/case5.1/hg38.fa ../output/case2.3/SRR23015877_R1_pair
ed.fastq.gz ../output/case2.3/SRR23015877_R2_paired.fastq.gz > ../output/
case2.3/SRR23015877.sam

# Step 4
source activate abc
```

```
# Install SAMtools
conda install -y samtools

# Convert the SAM files to BAM files
samtools view -
bS ../output/case2.3/SRR23015874.sam > ../output/case2.3/SRR23015874.
bam
samtools view -
bS ../output/case2.3/SRR23015876.sam > ../output/case2.3/SRR23015876.
bam
samtools view -
bS ../output/case2.3/SRR23015877.sam > ../output/case2.3/SRR23015877.
bam

# Step 5
source activate abc

# Sort and index the BAM files
samtools sort ../output/case2.3/SRR23015874.bam -
o ../output/case2.3/SRR23015874.sorted.bam
samtools index ../output/case2.3/SRR23015874.sorted.bam

samtools sort ../output/case2.3/SRR23015876.bam -
o ../output/case2.3/SRR23015876.sorted.bam
samtools index ../output/case2.3/SRR23015876.sorted.bam

samtools sort ../output/case2.3/SRR23015877.bam -
o ../output/case2.3/SRR23015877.sorted.bam
samtools index ../output/case2.3/SRR23015877.sorted.bam

# Step 6
source activate abc

# Install GATK
conda install -y gatk

# Mark duplicates in the BAM files
gatk MarkDuplicates -I ../output/case2.3/SRR23015874.sorted.bam -
O ../output/case2.3/SRR23015874.marked.bam -
M ../output/case2.3/SRR23015874.metrics.txt

gatk MarkDuplicates -I ../output/case2.3/SRR23015876.sorted.bam -
O ../output/case2.3/SRR23015876.marked.bam -
M ../output/case2.3/SRR23015876.metrics.txt

gatk MarkDuplicates -I ../output/case2.3/SRR23015877.sorted.bam -
O ../output/case2.3/SRR23015877.marked.bam -
M ../output/case2.3/SRR23015877.metrics.txt

# Step 7
# Activate the conda environment
source activate abc
```

```
# Perform local realignment around indels

# For SRR23015874
# Create a target list of intervals to be realigned
gatk -T RealignerTargetCreator -R ./examples/case5.1/hg38.fa -
I ../output/case2.3/SRR23015874.marked.bam -
o ../output/case2.3/SRR23015874.target_intervals.list

# Perform the realignment
gatk -T IndelRealigner -R ./examples/case5.1/hg38.fa -
I ../output/case2.3/SRR23015874.marked.bam -
targetIntervals ../output/case2.3/SRR23015874.target_intervals.list -
o ../output/case2.3/SRR23015874.realigned.bam

# For SRR23015876
# Create a target list of intervals to be realigned
gatk -T RealignerTargetCreator -R ./examples/case5.1/hg38.fa -
I ../output/case2.3/SRR23015876.marked.bam -
o ../output/case2.3/SRR23015876.target_intervals.list

# Perform the realignment
gatk -T IndelRealigner -R ./examples/case5.1/hg38.fa -
I ../output/case2.3/SRR23015876.marked.bam -
targetIntervals ../output/case2.3/SRR23015876.target_intervals.list -
o ../output/case2.3/SRR23015876.realigned.bam

# For SRR23015877
# Create a target list of intervals to be realigned
gatk -T RealignerTargetCreator -R ./examples/case5.1/hg38.fa -
I ../output/case2.3/SRR23015877.marked.bam -
o ../output/case2.3/SRR23015877.target_intervals.list

# Perform the realignment
gatk -T IndelRealigner -R ./examples/case5.1/hg38.fa -
I ../output/case2.3/SRR23015877.marked.bam -
targetIntervals ../output/case2.3/SRR23015877.target_intervals.list -
o ../output/case2.3/SRR23015877.realigned.bam

# Step 8
source activate abc

# Perform Base Quality Score Recalibration (BQSR)

# For SRR23015874
# Create a table of covariates
gatk BaseRecalibrator -R ./examples/case5.1/hg38.fa -
I ../output/case2.3/SRR23015874.realigned.bam -
O ../output/case2.3/SRR23015874.recal_data.table

# Apply the recalibration to the BAM file
gatk ApplyBQSR -R ./examples/case5.1/hg38.fa -
I ../output/case2.3/SRR23015874.realigned.bam --bqsr-recal-
```

```
file ../output/case2.3/SRR23015874.recal_data.table -
O ../output/case2.3/SRR23015874.recalibrated.bam

# For SRR23015876
# Create a table of covariates
gatk BaseRecalibrator -R ./examples/case5.1/hg38.fa -
I ../output/case2.3/SRR23015876.realigned.bam -
O ../output/case2.3/SRR23015876.recal_data.table

# Apply the recalibration to the BAM file
gatk ApplyBQSR -R ./examples/case5.1/hg38.fa -
I ../output/case2.3/SRR23015876.realigned.bam --bqsr-recal-
file ../output/case2.3/SRR23015876.recal_data.table -
O ../output/case2.3/SRR23015876.recalibrated.bam

# For SRR23015877
# Create a table of covariates
gatk BaseRecalibrator -R ./examples/case5.1/hg38.fa -
I ../output/case2.3/SRR23015877.realigned.bam -
O ../output/case2.3/SRR23015877.recal_data.table

# Apply the recalibration to the BAM file
gatk ApplyBQSR -R ./examples/case5.1/hg38.fa -
I ../output/case2.3/SRR23015877.realigned.bam --bqsr-recal-
file ../output/case2.3/SRR23015877.recal_data.table -
O ../output/case2.3/SRR23015877.recalibrated.bam

#step 9
source activate abc

# Install Manta
conda install -y manta

# Use Manta to identify structural variations in the tumor sample compared
to the matched normal sample

# For SRR23015874
manta --normalBam ../output/case2.3/SRR23015874.recalibrated.bam --
tumorBam ../output/case2.3/SRR23015876.recalibrated.bam --
referenceFasta ./examples/case5.1/hg38.fa --
runDir ../output/case2.3/manta_SRR23015874
../output/case2.3/manta_SRR23015874/runWorkflow.py

# For SRR23015876
manta --normalBam ../output/case2.3/SRR23015874.recalibrated.bam --
tumorBam ../output/case2.3/SRR23015877.recalibrated.bam --
referenceFasta ./examples/case5.1/hg38.fa --
runDir ../output/case2.3/manta_SRR23015876
../output/case2.3/manta_SRR23015876/runWorkflow.py
```

WGS/WES data analysis **Case 2.4: Structure variation identification without normal**

<table>
<tr><td>User's Inputs</td><td>Data and Description
SRR23015876_R1.fastq.gz: paired end read1 from human whole genome sequencing, tumor samples replicate 1
SRR23015876_R2.fastq.gz: paired end read2 from human whole genome sequencing, tumor samples replicate 1
SRR23015877_R1.fastq.gz: paired end read1 from human whole genome sequencing, tumor samples replicate 2
SRR23015877_R2.fastq.gz: paired end read2 from human whole genome sequencing, tumor samples replicate 2
TruSeq3-PE.fa: trimming adapter
hg38.fa: human hg38 reference genome fasta

Goal
To identify structural variations, which can include large insertions, deletions, inversions, duplications, and translocations based on the sequencing data from the tumor sample.</td></tr>
<tr><td>Generated Plans by AutoBA</td><td>1. First, we will use the 'FastQC' software to perform quality control checks on the raw sequence data.
2. Next, we will use the 'Trimmomatic' software to trim the low-quality bases and adapter sequences from the raw sequence data.
3. After that, we will use the 'BWA-MEM' software to align the trimmed sequence reads to the human reference genome.
4. Then, we will use the 'SAMtools' software to convert the alignment output from the previous step into a sorted BAM file.
5. Next, we will use the 'Pindel' software to detect structural variations in the sorted BAM file from the previous step.
6. Finally, we will use the 'SnpEff' software to annotate the detected structural variations and predict their effects on genes</td></tr>
<tr><td>Generated Codes by AutoBA</td><td># Step 1
source activate abc
conda install -y fastqc
fastqc ./examples/case5.1/SRR23015876_R1.fastq.gz -o ../output/case2.31
fastqc ./examples/case5.1/SRR23015876_R2.fastq.gz -o ../output/case2.31
fastqc ./examples/case5.1/SRR23015877_R1.fastq.gz -o ../output/case2.31
fastqc ./examples/case5.1/SRR23015877_R2.fastq.gz -o ../output/case2.31

Step 2
source activate abc
conda install -y trimmomatic
trimmomatic PE -
phred33 ./examples/case5.1/SRR23015876_R1.fastq.gz ./examples/case5.1/SRR23015876_R2.fastq.gz ../output/case2.31/SRR23015876_R1_paired.fastq.gz ../output/case2.31/SRR23015876_R1_unpaired.fastq.gz ../output/case2.31/SRR23015876_R2_paired.fastq.gz ../output/case2.31/SRR23015876_R2_unpaired.fastq.gz ILLUMINACLIP:./examples/case5.1/TruSeq3-PE.fa:2:30:10
trimmomatic PE -
phred33 ./examples/case5.1/SRR23015877_R1.fastq.gz ./examples/case5.1/SRR23015877_R2.fastq.gz ../output/case2.31/SRR23015877_R1_paired.fastq.gz ../output/case2.31/SRR23015877_R1_unpaired.fastq.gz ../output/case2.31/SRR23015877_R2_paired.fastq.gz ../output/case2.31/SRR230158</td></tr>
</table>

	77_R2_unpaired.fastq.gz ILLUMINACLIP:./examples/case5.1/TruSeq3-PE.fa:2:30:10<br><br>**# Step 3**<br>source activate abc<br>conda install -y bwa<br>bwa index ./examples/case5.1/hg38.fa<br>bwa mem ./examples/case5.1/hg38.fa ../output/case2.31/SRR23015876_R1_paired.fastq.gz ../output/case2.31/SRR23015876_R2_paired.fastq.gz > ../output/case2.31/SRR23015876.sam<br>bwa mem ./examples/case5.1/hg38.fa ../output/case2.31/SRR23015877_R1_paired.fastq.gz ../output/case2.31/SRR23015877_R2_paired.fastq.gz > ../output/case2.31/SRR23015877.sam<br><br>**# Step 4**<br>source activate abc<br>conda install -y samtools<br>samtools view -S -b ../output/case2.31/SRR23015876.sam > ../output/case2.31/SRR23015876.bam<br>samtools sort ../output/case2.31/SRR23015876.bam -o ../output/case2.31/SRR23015876_sorted.bam<br>samtools view -S -b ../output/case2.31/SRR23015877.sam > ../output/case2.31/SRR23015877.bam<br>samtools sort ../output/case2.31/SRR23015877.bam -o ../output/case2.31/SRR23015877_sorted.bam<br><br>**# Step 5**<br>source activate abc<br>conda install -y pindel<br>pindel -f ./examples/case5.1/hg38.fa -i ../output/case2.31/SRR23015876_sorted.bam -o ../output/case2.31/SRR23015876_pindel<br>pindel -f ./examples/case5.1/hg38.fa -i ../output/case2.31/SRR23015877_sorted.bam -o ../output/case2.31/SRR23015877_pindel<br><br>**# Step 6**<br>source activate abc<br>conda install -y snpeff<br>snpeff ann hg38 ../output/case2.31/SRR23015876_pindel > ../output/case2.31/SRR23015876_pindel_ann<br>snpeff ann hg38 ../output/case2.31/SRR23015877_pindel > ../output/case2.31/SRR23015877_pindel_ann

ChIP-Seq Analysis Case 3.1: Call Peaks

**User's Inputs**	**Data and Description**<br>SRR620204.fastq.gz: chip-seq data for Ring1B<br>SRR620205.fastq.gz: chip-seq data for cbx7<br>SRR620206.fastq.gz: chip-seq data for SUZ12<br>SRR620208.fastq.gz: chip-seq data for IgGold<br>mm39.ncbiRefSeq.gtf: genome annotations mouse<br>mm39.fa: mouse genome<br><br>**Goal**<br>Call peaks for protein cbx7 with IgGold as control
**Generated Plans by AutoBA**	1. Load the provided data files. The files include chip-seq data for Ring1B, cbx7, SUZ12, IgGold, genome annotations for mouse, and the mouse genome.<br>2. Use the software 'FastQC' to perform quality control checks on raw sequence data coming from high throughput sequencing pipelines.<br>3. Use the software 'Trim Galore' to trim low-quality ends from reads in addition to adapter removal.<br>4. Use the software 'Bowtie2' to align the trimmed reads to the mouse genome. Note: We are not using 'STAR' or 'java' based software as per the rules.<br>5. Use the software 'Samtools' to convert the aligned reads from SAM format to BAM format for easier handling and reduced storage space.<br>6. Use the software 'MACS2' to call peaks for the protein cbx7 with IgGold as control. This software will identify the regions in the genome that have been enriched with reads (peaks) from the chip-seq data.<br>7. Use the software 'Bedtools' to annotate the peaks with the genome annotations.<br>8. Finally, visualize the peaks using the software 'IGV' to confirm the peak calling.
**Generated Codes by AutoBA**	**# Step 1**<br># Activate the conda environment<br>source activate abc<br># Load the data files<br># Chip-seq data for Ring1B<br>zcat ./examples/case3.1/data/SRR620204.fastq.gz > ./examples/case3.1/data/SRR620204.fastq<br># Chip-seq data for cbx7<br>zcat ./examples/case3.1/data/SRR620205.fastq.gz > ./examples/case3.1/data/SRR620205.fastq<br># Chip-seq data for SUZ12<br>zcat ./examples/case3.1/data/SRR620206.fastq.gz > ./examples/case3.1/data/SRR620206.fastq<br># Chip-seq data for IgGold<br>zcat ./examples/case3.1/data/SRR620208.fastq.gz > ./examples/case3.1/data/SRR620208.fastq<br># Genome annotations for mouse<br>zcat ./examples/case3.1/data/mm39.ncbiRefSeq.gtf.gz > ./examples/case3.1/data/mm39.ncbiRefSeq.gtf<br># Mouse genome<br>zcat ./examples/case3.1/data/mm39.fa.gz > ./examples/case3.1/data/mm39.fa

```
# Step 2
# Activate the conda environment
source activate abc
# Install FastQC
conda install -y fastqc
# Perform quality control checks on raw sequence data
fastqc ./examples/case3.1/data/SRR620204.fastq
fastqc ./examples/case3.1/data/SRR620205.fastq
fastqc ./examples/case3.1/data/SRR620206.fastq
fastqc ./examples/case3.1/data/SRR620208.fastq

# Step 3
# Activate the conda environment
source activate abc
# Install Trim Galore
conda install -y trim-galore
# Trim low-quality ends from reads in addition to adapter removal
trim_galore ./examples/case3.1/data/SRR620204.fastq -
o ./examples/case3.1/output
trim_galore ./examples/case3.1/data/SRR620205.fastq -
o ./examples/case3.1/output
trim_galore ./examples/case3.1/data/SRR620206.fastq -
o ./examples/case3.1/output
trim_galore ./examples/case3.1/data/SRR620208.fastq -
o ./examples/case3.1/output

# Step 4
# Activate the conda environment
source activate abc
# Install Bowtie2
conda install -y bowtie2
# Build the index for the mouse genome
bowtie2-build ./examples/case3.1/data/mm39.fa mm39
# Align the trimmed reads to the mouse genome
bowtie2 -x mm39 -U ./examples/case3.1/output/SRR620204_val_1.fq -
S ./examples/case3.1/output/SRR620204.sam
bowtie2 -x mm39 -U ./examples/case3.1/output/SRR620205_val_1.fq -
S ./examples/case3.1/output/SRR620205.sam
bowtie2 -x mm39 -U ./examples/case3.1/output/SRR620206_val_1.fq -
S ./examples/case3.1/output/SRR620206.sam
bowtie2 -x mm39 -U ./examples/case3.1/output/SRR620208_val_1.fq -
S ./examples/case3.1/output/SRR620208.sam

# Step 5
# Activate the conda environment
source activate abc
# Install Samtools
conda install -y samtools
# Convert the aligned reads from SAM format to BAM format
samtools view -
bS ./examples/case3.1/output/SRR620204.sam > ./examples/case3.1/outpu
t/SRR620204.bam
```

	samtools view -<br>bS ./examples/case3.1/output/SRR620205.sam > ./examples/case3.1/outpu<br>t/SRR620205.bam<br>samtools view -<br>bS ./examples/case3.1/output/SRR620206.sam > ./examples/case3.1/outpu<br>t/SRR620206.bam<br>samtools view -<br>bS ./examples/case3.1/output/SRR620208.sam > ./examples/case3.1/outpu<br>t/SRR620208.bam<br><br>**# Step 6**<br># Activate the conda environment<br>source activate abc<br># Install MACS2<br>conda install -y macs2<br># Call peaks for the protein cbx7 with IgGold as control<br>macs2 callpeak -t ./examples/case3.1/output/SRR620205.bam -<br>c ./examples/case3.1/output/SRR620208.bam -f BAM -g mm -n<br>cbx7_vs_IgGold -B -q 0.01 --outdir ./examples/case3.1/output<br><br>**# Step 7**<br># Activate the conda environment<br>source activate abc<br># Install Bedtools<br>conda install -y bedtools<br># Annotate the peaks with the genome annotations<br>bedtools intersect -<br>a ./examples/case3.1/output/cbx7_vs_IgGold_peaks.narrowPeak -<br>b ./examples/case3.1/data/mm39.ncbiRefSeq.gtf > ./examples/case3.1/outp<br>ut/cbx7_vs_IgGold_peaks_annotated.bed<br><br>**# Step 8**<br># Activate the conda environment<br>source activate abc<br># Install IGV<br>conda install -y igv<br># Visualize the peaks<br>igv -<br>g ./examples/case3.1/data/mm39.fa ./examples/case3.1/output/cbx7_vs_Ig<br>Gold_peaks_annotated.bed

ChIP-Seq Analysis Case 3.2: Motif Discovery

<table>
<tr><td>User's Inputs</td><td>Data and Description
SRR620204.fastq.gz: chip-seq data for Ring1B
SRR620205.fastq.gz: chip-seq data for cbx7
SRR620206.fastq.gz: chip-seq data for SUZ12
SRR620208.fastq.gz: chip-seq data for IgGold
mm39.ncbiRefSeq.gtf: genome annotations mouse
mm39.fa: mouse genome

Goal
Discover motifs within the peaks of protein SUZ12 with IgGold as control</td></tr>
<tr><td>Generated Plans by AutoBA</td><td>1. Load the provided chip-seq data files for SUZ12 and IgGold using a software like 'FastQC' for quality control checks.
2. Use a software like 'Trim Galore!' to trim low quality bases and adapter sequences from the reads.
3. Align the trimmed reads to the mouse genome using a software like 'Bowtie2', avoiding the use of 'STAR' and 'java'.
4. Use a software like 'MACS2' to call peaks from the aligned reads, using IgGold data as control.
5. Use a software like 'HOMER' to discover motifs within the peaks of SUZ12.
6. Validate the discovered motifs using a software like 'MEME'.</td></tr>
<tr><td>Generated Codes by AutoBA</td><td># Step 1
Activate the conda environment
source activate abc
Install FastQC
conda install -y fastqc
Run FastQC on the SUZ12 data
fastqc ./examples/case3.2/data/SRR620206.fastq.gz -
o ./examples/case3.2/output
Run FastQC on the IgGold data
fastqc ./examples/case3.2/data/SRR620208.fastq.gz -
o ./examples/case3.2/output

Step 2
Activate the conda environment
source activate abc
Install Trim Galore!
conda install -y trim-galore
Trim low quality bases and adapter sequences from the SUZ12 data
trim_galore ./examples/case3.2/data/SRR620206.fastq.gz -
o ./examples/case3.2/output
Trim low quality bases and adapter sequences from the IgGold data
trim_galore ./examples/case3.2/data/SRR620208.fastq.gz -
o ./examples/case3.2/output

Step 3
Activate the conda environment
source activate abc
Install Bowtie2
conda install -y bowtie2
Build the Bowtie2 index for the mouse genome</td></tr>
</table>

	bowtie2-<br>build ./examples/case3.2/data/mm39.fa ./examples/case3.2/output/mm39<br># Align the trimmed reads of SUZ12 to the mouse genome<br>bowtie2 -x ./examples/case3.2/output/mm39 -<br>U ./examples/case3.2/output/SRR620206_trimmed.fq.gz -<br>S ./examples/case3.2/output/SRR620206.sam<br># Align the trimmed reads of IgGold to the mouse genome<br>bowtie2 -x ./examples/case3.2/output/mm39 -<br>U ./examples/case3.2/output/SRR620208_trimmed.fq.gz -<br>S ./examples/case3.2/output/SRR620208.sam<br><br>**# Step 4**<br># Activate the conda environment<br>source activate abc<br># Install MACS2<br>conda install -y macs2<br># Call peaks from the aligned reads of SUZ12 using IgGold data as control<br>macs2 callpeak -t ./examples/case3.2/output/SRR620206.sam -<br>c ./examples/case3.2/output/SRR620208.sam -f SAM -g mm -n SUZ12 -B -<br>q 0.01 --outdir ./examples/case3.2/output<br><br>**# Step 5**<br># Activate the conda environment<br>source activate abc<br># Install HOMER<br>conda install -y homer<br># Discover motifs within the peaks of SUZ12<br>findMotifsGenome.pl ./examples/case3.2/output/SUZ12_peaks.narrowPeak<br>mm ./examples/case3.2/output/HOMER_motifs -size given<br><br>**# Step 6**<br># Activate the conda environment<br>source activate abc<br># Install MEME<br>conda install -y meme<br># Validate the discovered motifs using MEME<br>meme ./examples/case3.2/output/HOMER_motifs -<br>oc ./examples/case3.2/output/MEME_motifs

ChIP-Seq Analysis Case 3.3: Functional Enrichment

<table>
<tr><td>User's Inputs</td><td>Data and Description
SRR620204.fastq.gz: chip-seq data for Ring1B
SRR620205.fastq.gz: chip-seq data for cbx7
SRR620206.fastq.gz: chip-seq data for SUZ12
SRR620208.fastq.gz: chip-seq data for IgGold
mm39.ncbiRefSeq.gtf: genome annotations mouse
mm39.fa: mouse genome

Goal
Perform functional enrichment for protein Ring1B, use protein IgGold as the control</td></tr>
<tr><td>Generated Plans by AutoBA</td><td>1. Load the provided chip-seq data files for Ring1B and IgGold using a software like FastQC for quality control checks.
2. Use a software like BWA (Burrows-Wheeler Aligner) to align the chip-seq data for Ring1B and IgGold to the mouse genome (mm39.fa).
3. Use a software like MACS (Model-based Analysis of ChIP-Seq) to identify the peaks in the chip-seq data for Ring1B and IgGold.
4. Use the genome annotations file (mm39.ncbiRefSeq.gtf) to annotate the identified peaks using a software like BEDTools.
5. Use a software like DESeq2 to perform differential binding analysis between Ring1B and IgGold.
6. The genes associated with the differential peaks will be identified and used for functional enrichment analysis.
7. Use a software like DAVID (Database for Annotation, Visualization and Integrated Discovery) or g:Profiler for functional enrichment analysis.
8. Interpret the results of the functional enrichment analysis to understand the biological processes and pathways associated with Ring1B.</td></tr>
<tr><td>Generated Codes by AutoBA</td><td># Step 1
Activate the conda environment
source activate abc
Install FastQC
conda install -y fastqc
Load the chip-seq data files for Ring1B and IgGold
fastqc ./examples/case3.3/data/SRR620204.fastq.gz -
o ./examples/case3.3/output
fastqc ./examples/case3.3/data/SRR620208.fastq.gz -
o ./examples/case3.3/output

Step 2
Activate the conda environment
source activate abc
Install BWA
conda install -y bwa
Align the chip-seq data for Ring1B to the mouse genome
bwa
mem ./examples/case3.3/data/mm39.fa ./examples/case3.3/data/SRR6202
04.fastq.gz > ./examples/case3.3/output/Ring1B.sam
Align the chip-seq data for IgGold to the mouse genome</td></tr>
</table>

```
bwa
mem ./examples/case3.3/data/mm39.fa ./examples/case3.3/data/SRR6202
08.fastq.gz > ./examples/case3.3/output/IgGold.sam

# Step 3
# Activate the conda environment
source activate abc
# Install MACS
conda install -y macs
# Identify the peaks in the chip-seq data for Ring1B
macs2 callpeak -t ./examples/case3.3/output/Ring1B.sam -f SAM -g mm -n
Ring1B -B -q 0.01 --outdir ./examples/case3.3/output
# Identify the peaks in the chip-seq data for IgGold
macs2 callpeak -t ./examples/case3.3/output/IgGold.sam -f SAM -g mm -n
IgGold -B -q 0.01 --outdir ./examples/case3.3/output

# Step 4
# Activate the conda environment
source activate abc
# Install BEDTools
conda install -y bedtools
# Annotate the identified peaks for Ring1B
bedtools intersect -a ./examples/case3.3/output/Ring1B_peaks.narrowPeak
-b ./examples/case3.3/data/mm39.ncbiRefSeq.gtf -wa -
wb > ./examples/case3.3/output/Ring1B_annotated_peaks.bed
# Annotate the identified peaks for IgGold
bedtools intersect -a ./examples/case3.3/output/IgGold_peaks.narrowPeak -
b ./examples/case3.3/data/mm39.ncbiRefSeq.gtf -wa -
wb > ./examples/case3.3/output/IgGold_annotated_peaks.bed

# Step 5
# Activate the conda environment
source activate abc
# Install DESeq2
conda install -y bioconductor-deseq2
# Perform differential binding analysis between Ring1B and IgGold
Rscript -e "library('DESeq2');
ring1b_counts <-
read.table('./examples/case3.3/output/Ring1B_annotated_peaks.bed',
header = TRUE, stringsAsFactors = FALSE);
igGold_counts <-
read.table('./examples/case3.3/output/IgGold_annotated_peaks.bed',
header = TRUE, stringsAsFactors = FALSE);
counts <- cbind(ring1b_counts, igGold_counts);
colData <- data.frame(condition = factor(c('Ring1B', 'IgGold')));
dds <- DESeqDataSetFromMatrix(countData = counts, colData = colData,
design = ~ condition);
dds <- DESeq(dds);
res <- results(dds);
write.table(res, file =
'./examples/case3.3/output/differential_binding_analysis.txt', sep = '  ', quote
= FALSE, row.names = TRUE)"
```

```
# Step 6
# Activate the conda environment
source activate abc
# Install BEDTools
conda install -y bedtools
# Identify the genes associated with the differential peaks for Ring1B
bedtools intersect -
a ./examples/case3.3/output/Ring1B_annotated_peaks.bed -
b ./examples/case3.3/data/mm39.ncbiRefSeq.gtf -wa -
wb > ./examples/case3.3/output/Ring1B_genes.bed
# Identify the genes associated with the differential peaks for IgGold
bedtools intersect -
a ./examples/case3.3/output/IgGold_annotated_peaks.bed -
b ./examples/case3.3/data/mm39.ncbiRefSeq.gtf -wa -
wb > ./examples/case3.3/output/IgGold_genes.bed

# Step 7
# Activate the conda environment
source activate abc
# Install g:Profiler
conda install -y gprofiler
# Perform functional enrichment analysis for Ring1B
Rscript -e "library('gProfileR');
ring1b_genes <- read.table('./examples/case3.3/output/Ring1B_genes.bed',
header = TRUE, stringsAsFactors = FALSE);
ring1b_enrichment <- gprofiler(ring1b_genes$V4, organism='mmusculus');
write.table(ring1b_enrichment, file =
'./examples/case3.3/output/Ring1B_enrichment.txt', sep = '\t', quote =
FALSE, row.names = TRUE)"
# Perform functional enrichment analysis for IgGold
Rscript -e "library('gProfileR');
igGold_genes <- read.table('./examples/case3.3/output/IgGold_genes.bed',
header = TRUE, stringsAsFactors = FALSE);
igGold_enrichment <- gprofiler(igGold_genes$V4, organism='mmusculus');

# Step 8
# Activate the conda environment
source activate abc
# Install R
conda install -y r-base
# Interpret the results of the functional enrichment analysis for Ring1B
Rscript -e "ring1b_enrichment <-
read.table('./examples/case3.3/output/Ring1B_enrichment.txt', header =
TRUE, stringsAsFactors = FALSE);
ring1b_enrichment <- ring1b_enrichment[order(-
ring1b_enrichment$p.value), ];
ring1b_enrichment <- ring1b_enrichment[ring1b_enrichment$p.value <
0.05, ];
write.table(ring1b_enrichment, file =
'./examples/case3.3/output/Ring1B_enrichment_significant.txt', sep = '\t',
quote = FALSE, row.names = TRUE)"
```

Bisulfite-Seq data analysis   Case 4.1: Identifying DNA methylation

<table>
<tr><td>User's Inputs</td><td>Data and Description
Sample_R1.fastq.gz: Raw sequencing reads from the bisulfite-converted DNA, typically paired-end reads. These reads contain information about the converted DNA, where unmethylated cytosines are converted to uracils (T) during library preparation.
Sample_R2.fastq.gz: Paired-end reads that complement the R1 reads. These are typically used for quality control and paired-end analysis.
TruSeq3-PE.fa: Adapter sequences for trimming
Reference.fasta: Reference genome for the organism under study in FASTA format, include bisulfite conversion.

Goal
To identify DNA methylation patterns in the genome based on the Bisulfite-Seq data.</td></tr>
<tr><td>Generated Plans by AutoBA</td><td>1. Load the raw sequencing reads from the bisulfite-converted DNA, typically paired-end reads. These reads contain information about the converted DNA, where unmethylated cytosines are converted to uracils (T) during library preparation. The file path is './experiments/case4.1/data/Sample_R1.fastq.gz'.
2. Load the paired-end reads that complement the R1 reads. These are typically used for quality control and paired-end analysis. The file path is './experiments/case4.1/data/Sample_R2.fastq.gz'.
3. Load the adapter sequences for trimming. The file path is './experiments/case4.1/data/TruSeq3-PE.fa'.
4. Load the reference genome for the organism under study in FASTA format, include bisulfite conversion. The file path is './experiments/case4.1/data/Reference.fasta'.
5. Use the software 'Trim Galore!' to trim the adapter sequences from the raw sequencing reads. This step is necessary to ensure that the subsequent analysis is not affected by the adapter sequences.
6. Use the software 'Bismark' to align the trimmed reads to the reference genome. This step is necessary to identify the location of each read in the genome.
7. Use the software 'Bismark' to extract the methylation information from the aligned reads. This step is necessary to identify the methylation status of each cytosine in the genome.
8. Use the software 'Bismark' to generate a report of the methylation analysis. This report will contain information about the methylation status of each cytosine in the genome, as well as other relevant information such as the coverage and the bisulfite conversion rate.
9. Use the software 'IGV' to visualize the methylation patterns in the genome. This step is necessary to interpret the results of the methylation analysis.</td></tr>
<tr><td>Generated Codes by AutoBA</td><td># Step 1-5
#!/bin/bash

Activate the conda environment
source activate abc

Add conda-forge and bioconda to the list of channels
conda config --add channels conda-forge</td></tr>
</table>

	conda config --add channels bioconda<br><br># Install Trim Galore!<br>conda install -y trim-galore<br><br># Trim the adapter sequences from the raw sequencing reads<br>trim_galore --<br>paired ./experiments/case4.1/data/Sample_R1.fastq.gz ./experiments/case4<br>.1/data/Sample_R2.fastq.gz --output_dir ./experiments/case4.1/output<br><br>**# Step 6**<br>#!/bin/bash<br><br># Activate the conda environment<br>source activate abc<br><br># Add conda-forge and bioconda to the list of channels<br>conda config --add channels conda-forge<br>conda config --add channels bioconda<br><br># Install Bismark<br>conda install -y bismark<br><br># Align the trimmed reads to the reference genome<br>bismark ./experiments/case4.1/data/Reference.fasta --<br>output_dir ./experiments/case4.1/output --<br>un ./experiments/case4.1/output/Sample_R1_val_1.fq.gz ./experiments/cas<br>e4.1/output/Sample_R2_val_2.fq.gz<br><br>**# Step 7**<br>#!/bin/bash<br><br># Activate the conda environment<br>source activate abc<br><br># Add conda-forge and bioconda to the list of channels<br>conda config --add channels conda-forge<br>conda config --add channels bioconda<br><br># Extract the methylation information from the aligned reads<br>bismark_methylation_extractor --output ./experiments/case4.1/output --gzip<br>--comprehensive --<br>bedGraph ./experiments/case4.1/output/Sample_R1_val_1_bismark_bt2_p<br>e.bam<br><br>**# Step 8**<br>#!/bin/bash<br><br># Activate the conda environment<br>source activate abc<br><br># Add conda-forge and bioconda to the list of channels<br>conda config --add channels conda-forge

	conda config --add channels bioconda<br><br># Generate a report of the methylation analysis<br>bismark2report --output ./experiments/case4.1/output --<br>dir ./experiments/case4.1/output<br><br>**# Step 9**<br>#!/bin/bash<br><br># Activate the conda environment<br>source activate abc<br><br># Add conda-forge and bioconda to the list of channels<br>conda config --add channels conda-forge<br>conda config --add channels bioconda<br><br># Install IGV<br>conda install -y igv<br><br># Visualize the methylation patterns in the genome<br>igv -<br>g ./experiments/case4.1/data/Reference.fasta ./experiments/case4.1/output/<br>Sample_R1_val_1_bismark_bt2_pe.bam

ATAC-seq data analysis **Case 5.1: Identifying open chromatin regions**

**User's Inputs**	**Data and Description**<br>Sample_R1.fastq.gz: Raw sequencing reads from the ATAC-seq experiment. These are typically paired-end reads representing fragments of open chromatin regions.<br>Sample_R2.fastq.gz: Paired-end reads that complement the R1 reads.<br>TruSeq3-PE.fa: Adapter sequences for trimming<br>Reference.fasta: Reference genome for the organism under study in FASTA format. It matches the genome used for the ATAC-seq library preparation.<br><br>**Goal**<br>To identify open chromatin regions within the genome based on the ATAC-seq data.
**Generated Plans by AutoBA**	1. Load the raw sequencing reads from the ATAC-seq experiment. The file path is './experiments/case5.1/data/Sample_R1.fastq.gz'.<br>2. Load the paired-end reads that complement the R1 reads. The file path is './experiments/case5.1/data/Sample_R2.fastq.gz'.<br>3. Load the adapter sequences for trimming. The file path is './experiments/case5.1/data/TruSeq3-PE.fa'.<br>4. Load the reference genome for the organism under study in FASTA format. The file path is './experiments/case5.1/data/Reference.fasta'.<br>5. Use the software 'Trim Galore!' to trim the adapter sequences from the raw sequencing reads. This will ensure that the subsequent analysis is not affected by the presence of these sequences.<br>6. Use the software 'BWA' to align the trimmed reads to the reference genome. This will generate a BAM file containing the aligned reads.<br>7. Use the software 'Samtools' to sort and index the BAM file. This will facilitate the subsequent analysis of the aligned reads.<br>8. Use the software 'MACS2' to identify peaks in the aligned reads. These peaks represent open chromatin regions within the genome.<br>9. Use the software 'BEDTools' to convert the peaks identified by MACS2 into a BED file. This file can be used for further analysis of the open chromatin regions.<br>10. Use the software 'UCSC Genome Browser' to visualize the open chromatin regions within the genome. This will provide a graphical representation of the regions identified by the ATAC-seq experiment.
**Generated Codes by AutoBA**	**# Step 1-5**<br>source activate abc<br>conda config --add channels conda-forge<br>conda config --add channels bioconda<br>conda install -y trim-galore<br>trim_galore --<br>paired ./experiments/case5.1/data/Sample_R1.fastq.gz ./experiments/case5<br>.1/data/Sample_R2.fastq.gz --output_dir ./experiments/case5.1/output<br><br>**# Step 6**<br>source activate abc<br>conda config --add channels conda-forge<br>conda config --add channels bioconda<br>conda install -y bwa

	bwa<br>mem ./experiments/case5.1/data/Reference.fasta ./experiments/case5.1/output/Sample_R1_val_1.fq.gz ./experiments/case5.1/output/Sample_R2_val_2.fq.gz > ./experiments/case5.1/output/aligned_reads.bam<br><br>**# Step 7**<br>source activate abc<br>conda config --add channels conda-forge<br>conda config --add channels bioconda<br>conda install -y samtools<br>samtools sort ./experiments/case5.1/output/aligned_reads.bam -o ./experiments/case5.1/output/sorted_reads.bam<br>samtools index ./experiments/case5.1/output/sorted_reads.bam<br><br>**# Step 8**<br>source activate abc<br>conda config --add channels conda-forge<br>conda config --add channels bioconda<br>conda install -y macs2<br>macs2 callpeak -t ./experiments/case5.1/output/sorted_reads.bam -f BAM -g hs -n ./experiments/case5.1/output/peaks<br><br>**# Step 9**<br>source activate abc<br>conda config --add channels conda-forge<br>conda config --add channels bioconda<br>conda install -y bedtools<br>bedtools bamtobed -i ./experiments/case5.1/output/peaks_peaks.narrowPeak > ./experiments/case5.1/output/peaks.bed

DNase-seq data analysis **Case 6.1: Identifying DnaseI hypersensitive site**

**User's Inputs**	**Data and Description**<br>Sample_R1.fastq.gz: Raw sequencing reads from the DNase-seq experiment.<br>Sample_R2.fastq.gz: Paired-end reads that complement the R1 reads, often used for quality control and paired-end analysis.<br>TruSeq3-PE.fa: Adapter sequences for trimming.<br>Reference.fasta: Reference genome for the organism under study in FASTA format<br><br>**Goal**<br>To identify DNase I hypersensitive sites (regions of open chromatin) within the genome based on the DNase-seq data.
**Generated Plans by AutoBA**	1. Load the raw sequencing reads from the DNase-seq experiment (Sample_R1.fastq.gz) and the paired-end reads (Sample_R2.fastq.gz) into a quality control software such as FastQC. This will allow us to assess the quality of the sequencing data.<br>2. Use a software like Cutadapt to trim the adapter sequences (TruSeq3-PE.fa) from the raw sequencing reads. This step is necessary to ensure that the subsequent analysis is not affected by the presence of these adapter sequences.<br>3. Align the trimmed sequencing reads to the reference genome (Reference.fasta) using a software like BWA. This step will generate a BAM file that contains the aligned reads.<br>4. Use a software like MACS2 to identify the DNase I hypersensitive sites within the genome. This software identifies regions of the genome that are enriched in the aligned reads, which correspond to the open chromatin regions.<br>5. Visualize the identified DNase I hypersensitive sites using a genome browser software like IGV. This will allow us to visually inspect the identified regions and assess the quality of the analysis.<br>6. Finally, perform downstream analysis such as functional annotation of the identified DNase I hypersensitive sites using a software like GREAT. This will provide insights into the potential biological functions of these open chromatin regions.
**Generated Codes by AutoBA**	**# Step 1**<br>source activate abc<br>conda config --add channels conda-forge<br>conda config --add channels bioconda<br>conda install -y fastqc<br>fastqc ./experiments/case5.1/data/Sample_R1.fastq.gz<br>fastqc ./experiments/case5.1/data/Sample_R2.fastq.gz<br><br>**# Step 2**<br>source activate abc<br>conda config --add channels conda-forge<br>conda config --add channels bioconda<br>conda install -y cutadapt<br>cutadapt -a file:./experiments/case5.1/data/TruSeq3-PE.fa -o ./experiments/case5.1/output/Sample_R1_trimmed.fastq.gz ./experiments/case5.1/data/Sample_R1.fastq.gz

	cutadapt -a file:./experiments/case5.1/data/TruSeq3-PE.fa -<br>o ./experiments/case5.1/output/Sample_R2_trimmed.fastq.gz ./experiments<br>/case5.1/data/Sample_R2.fastq.gz<br><br>**# Step 3**<br>source activate abc<br>conda config --add channels conda-forge<br>conda config --add channels bioconda<br>conda install -y bwa<br>bwa index ./experiments/case5.1/data/Reference.fasta<br>bwa<br>mem ./experiments/case5.1/data/Reference.fasta ./experiments/case5.1/out<br>put/Sample_R1_trimmed.fastq.gz > ./experiments/case5.1/output/Sample_<br>R1_aligned.sam<br>bwa<br>mem ./experiments/case5.1/data/Reference.fasta ./experiments/case5.1/out<br>put/Sample_R2_trimmed.fastq.gz > ./experiments/case5.1/output/Sample_<br>R2_aligned.sam<br><br>**# Step 4**<br>source activate abc<br>conda config --add channels conda-forge<br>conda config --add channels bioconda<br>conda install -y macs2<br>macs2 callpeak -t ./experiments/case5.1/output/Sample_R1_aligned.sam -<br>n ./experiments/case5.1/output/Sample_R1_peaks<br>macs2 callpeak -t ./experiments/case5.1/output/Sample_R2_aligned.sam -<br>n ./experiments/case5.1/output/Sample_R2_peaks<br><br>**# Step 5**<br>source activate abc<br>conda config --add channels conda-forge<br>conda config --add channels bioconda<br>conda install -y igv<br>igv -<br>g ./experiments/case5.1/data/Reference.fasta ./experiments/case5.1/output/<br>Sample_R1_peaks.narrowPeak ./experiments/case5.1/output/Sample_R2_<br>peaks.narrowPeak<br><br>**# Step 6**<br>source activate abc<br>conda config --add channels conda-forge<br>conda config --add channels bioconda<br># Unfortunately, GREAT is a web-based tool and cannot be run directly<br>from the command line. However, the following steps can be followed to<br>use GREAT:<br># 1. Go to the GREAT website (http://great.stanford.edu/public/html/)<br># 2. Upload the peak files (Sample_R1_peaks.narrowPeak and<br>Sample_R2_peaks.narrowPeak) in the "Submit Your Regions" section<br># 3. Select the appropriate species and genome version in the "Select<br>Species and Assembly" section<br># 4. Click on the "Submit" button to run the analysis

4C-seq data analysis Case 7.1: Find genomics interactions

<table>
<tr><td>User's Inputs</td><td>Data and Description
sample_4C-seq_data.fastq: Raw sequencing reads or aligned data from the 4C-seq experiment, depending on the starting point.
viewpoint_coordinates.bed: A file specifying the genomic coordinates of the viewpoint we want to study.
reference_genome.fasta: A reference genome in FASTA format for the organism under study. This should match the genome used for the 4C-seq library preparation.

Goal
To identify genomic interactions originating from a specific genomic viewpoint using 4C-seq data.</td></tr>
<tr><td>Generated Plans by AutoBA</td><td>1. Load the raw 4C-seq data from './experiments/case7.1/data/sample_4C-seq_data.fastq'. This file contains the raw sequencing reads or aligned data from the 4C-seq experiment.
2. Load the viewpoint coordinates from './experiments/case7.1/data/viewpoint_coordinates.bed'. This file specifies the genomic coordinates of the viewpoint we want to study.
3. Load the reference genome from './experiments/case7.1/data/reference_genome.fasta'. This file contains a reference genome in FASTA format for the organism under study. This should match the genome used for the 4C-seq library preparation.
4. Use a software like 'Bowtie2' to align the raw 4C-seq data to the reference genome. This step will generate an alignment file that maps the sequencing reads to the reference genome.
5. Use a software like 'SAMtools' to convert the alignment file into a sorted BAM file. This step will make the data easier to handle and analyze.
6. Use a software like 'BEDTools' to intersect the sorted BAM file with the viewpoint coordinates. This step will identify the genomic interactions originating from the specific genomic viewpoint.
7. Use a software like 'R' to visualize the genomic interactions. This step will generate a plot showing the genomic interactions originating from the specific genomic viewpoint.</td></tr>
<tr><td>Generated Codes by AutoBA</td><td># Step 1
source activate abc
conda config --add channels conda-forge
conda config --add channels bioconda
conda install -y fastqc
fastqc ./experiments/case7.1/data/sample_4C-seq_data.fastq

Step 2
source activate abc
conda config --add channels conda-forge
conda config --add channels bioconda
conda install -y bedtools
bedtools getfasta -fi ./experiments/case7.1/data/reference_genome.fasta -bed ./experiments/case7.1/data/viewpoint_coordinates.bed

Step 3
source activate abc
conda config --add channels conda-forge
conda config --add channels bioconda</td></tr>
</table>

	conda install -y samtools<br>samtools faidx ./experiments/case7.1/data/reference_genome.fasta<br><br>**# Step 4**<br>source activate abc<br>conda config --add channels conda-forge<br>conda config --add channels bioconda<br>conda install -y bowtie2<br>bowtie2-build ./experiments/case7.1/data/reference_genome.fasta reference<br>bowtie2 -x reference -U ./experiments/case7.1/data/sample_4C-seq_data.fastq -S ./experiments/case7.1/output/sample_4C-seq_data.sam<br><br>**# Step 5**<br>source activate abc<br>conda config --add channels conda-forge<br>conda config --add channels bioconda<br>conda install -y samtools<br>samtools view -S -b ./experiments/case7.1/output/sample_4C-seq_data.sam > ./experiments/case7.1/output/sample_4C-seq_data.bam<br>samtools sort ./experiments/case7.1/output/sample_4C-seq_data.bam -o ./experiments/case7.1/output/sample_4C-seq_data_sorted.bam<br><br>**# Step 6**<br>source activate abc<br>conda config --add channels conda-forge<br>conda config --add channels bioconda<br>conda install -y bedtools<br>bedtools intersect -a ./experiments/case7.1/output/sample_4C-seq_data_sorted.bam -b ./experiments/case7.1/data/viewpoint_coordinates.bed > ./experiments/case7.1/output/intersected.bam<br><br>**# Step 7**<br>source activate abc<br>conda config --add channels conda-forge<br>conda config --add channels bioconda<br>conda install -y r-base<br>Rscript -e "library(ggplot2); library(GenomicRanges); library(rtracklayer); bam_file <- system.file('./experiments/case7.1/output/intersected.bam'); track <- import(bam_file); plot(track)"}

Nanopore DNA sequencing data analysis Case 8.1: Genome assembly

<table>
<tr><td>User's Inputs</td><td>Data and Description
sample_nanopore_reads.fastq: Raw Nanopore sequencing data in fastq format.
reference_genome.fasta: A reference genome in FASTA format, if a closely related reference is available and used as a guide during the assembly

Goal
To assemble a genome from the Nanopore sequencing data.</td></tr>
<tr><td>Generated Plans by AutoBA</td><td>1. Load the raw Nanopore sequencing data from './experiments/case8.1/data/sample_nanopore_reads.fastq'.
2. Load the reference genome from './experiments/case8.1/data/reference_genome.fasta'.
3. Use the software 'Canu' to correct the errors in the raw Nanopore sequencing data. This step will improve the quality of the data and make it more suitable for genome assembly.
4. Use the software 'Minimap2' to align the corrected Nanopore sequencing data to the reference genome. This step will help to identify the regions of the genome that are covered by the sequencing data.
5. Use the software 'Racon' to polish the alignment. This step will further improve the quality of the alignment by correcting any remaining errors.
6. Use the software 'Flye' to assemble the genome from the polished alignment. This step will generate a draft genome assembly.
7. Use the software 'Medaka' to polish the draft genome assembly. This step will correct any errors in the assembly and improve its quality.
8. Use the software 'Bandage' to visualize the final genome assembly. This step will allow us to inspect the assembly and identify any potential issues.</td></tr>
<tr><td>Generated Codes by AutoBA</td><td># Step 1-3
Activate the conda environment
source activate abc

Add conda-forge and bioconda to the list of channels
conda config --add channels conda-forge
conda config --add channels bioconda

Install Canu
conda install -y canu

# Define the raw data and output directory
raw_data='./experiments/case8.1/data/sample_nanopore_reads.fastq'
output_dir='./experiments/case8.1/output'

Use Canu to correct the errors in the raw Nanopore sequencing data
canu -correct -p ecoli -d $output_dir genomeSize=4.8m -nanopore-raw $raw_data

Step 4
Activate the conda environment
source activate abc

Add conda-forge and bioconda to the list of channels
conda config --add channels conda-forge
conda config --add channels bioconda</td></tr>
</table>

```
# Install Minimap2
conda install -y minimap2

# Define the corrected data and reference genome
corrected_data='./experiments/case8.1/output/ecoli.correctedReads.fasta.g
z'
reference_genome='./experiments/case8.1/data/reference_genome.fasta'

# Use Minimap2 to align the corrected Nanopore sequencing data to the
reference genome
minimap2 -a $reference_genome
$corrected_data > ./experiments/case8.1/output/alignment.sam

# Step 5
# Activate the conda environment
source activate abc

# Add conda-forge and bioconda to the list of channels
conda config --add channels conda-forge
conda config --add channels bioconda

# Install Racon
conda install -y racon

# Define the alignment file and reference genome
alignment='./experiments/case8.1/output/alignment.sam'
reference_genome='./experiments/case8.1/data/reference_genome.fasta'

# Use Racon to polish the alignment
racon $alignment
$reference_genome > ./experiments/case8.1/output/polished_alignment.fas
ta

# Step 6
# Activate the conda environment
source activate abc

# Add conda-forge and bioconda to the list of channels
conda config --add channels conda-forge
conda config --add channels bioconda

# Install Flye
conda install -y flye

# Define the polished alignment file
polished_alignment='./experiments/case8.1/output/polished_alignment.fasta
'
# Step 7
# Use Flye to assemble the genome from the polished alignment
flye --nano-raw $polished_alignment --out-
dir ./experiments/case8.1/output/flye_assembly
```

	# Activate the conda environment<br>source activate abc<br><br># Add conda-forge and bioconda to the list of channels<br>conda config --add channels conda-forge<br>conda config --add channels bioconda<br><br># Install Medaka<br>conda install -y medaka<br><br># Define the draft genome assembly file<br>draft_assembly='./experiments/case8.1/output/flye_assembly/assembly.fast<br>a'<br><br># Use Medaka to polish the draft genome assembly<br>medaka_consensus -i $draft_assembly -<br>d ./experiments/case8.1/output/flye_assembly -<br>o ./experiments/case8.1/output/medaka_polished<br><br>**# Step 8**<br># Activate the conda environment<br>source activate abc<br><br># Add conda-forge and bioconda to the list of channels<br>conda config --add channels conda-forge<br>conda config --add channels bioconda<br><br># Install Bandage<br>conda install -y bandage<br><br># Define the final genome assembly file<br>final_assembly='./experiments/case8.1/output/medaka_polished/consensus<br>.fasta'<br><br># Use Bandage to visualize the final genome assembly<br>Bandage image<br>$final_assembly ./experiments/case8.1/output/final_assembly.png

Nanopore DNA sequencing data analysis   Case 8.2: Tandem repeats variation identification

<table>
<tr><td>User's Inputs</td><td>Data and Description
sample_nanopore_reads.fastq: Raw Nanopore sequencing data in fastq format.
reference_genome.fasta: A reference genome in FASTA format, if a closely related reference is available and used as a guide during the assembly

Goal
To identify tandem repeats.</td></tr>
<tr><td>Generated Plans by AutoBA</td><td>1. Load the raw Nanopore sequencing data and the reference genome from the provided file paths './experiments/case8.2/data/sample_nanopore_reads.fastq' and './experiments/case8.2/data/reference_genome.fasta' respectively.
2. Use the software 'Minimap2' to map the raw sequencing reads to the reference genome. This will help in identifying the regions of the genome that have been sequenced.
3. Use the software 'Samtools' to convert the output of Minimap2 (which is in SAM format) to BAM format. This is a binary format that is more efficient for downstream analysis.
4. Use the software 'Samtools' again to sort the BAM file. This will arrange the mapped reads in the order of their position on the reference genome.
5. Use the software 'Samtools' once more to index the sorted BAM file. This will create an index file (.bai) that allows for quick access to any part of the BAM file.
6. Use the software 'TRF' (Tandem Repeats Finder) to identify tandem repeats in the reference genome. This software uses an algorithm to find and analyze tandem repeats in DNA sequences.
7. The output of TRF will be a list of tandem repeats, their location on the reference genome, and other details about the repeats. This information can be used for further analysis.</td></tr>
<tr><td>Generated Codes by AutoBA</td><td># Step 1-2
source activate abc
conda config --add channels conda-forge
conda config --add channels bioconda
conda install -y minimap2
conda install -y samtools
conda install -y trf
minimap2 -a ./experiments/case8.2/data/reference_genome.fasta ./experiments/case8.2/data/sample_nanopore_reads.fastq > ./experiments/case8.2/output/aligned.sam

Step 3
source activate abc
conda config --add channels conda-forge
conda config --add channels bioconda
conda install -y samtools
samtools view -bS ./experiments/case8.2/output/aligned.sam > ./experiments/case8.2/output/aligned.bam

Step 4
source activate abc</td></tr>
</table>

	conda config --add channels conda-forge<br>conda config --add channels bioconda<br>conda install -y samtools<br>samtools sort ./experiments/case8.2/output/aligned.bam -<br>o ./experiments/case8.2/output/aligned_sorted.bam<br><br>**# Step 5**<br>source activate abc<br>conda config --add channels conda-forge<br>conda config --add channels bioconda<br>conda install -y samtools<br>samtools index ./experiments/case8.2/output/aligned_sorted.bam<br><br>**# Step 6**<br>source activate abc<br>conda config --add channels conda-forge<br>conda config --add channels bioconda<br>conda install -y trf<br>trf ./experiments/case8.2/data/reference_genome.fasta ./experiments/case8<br>.2/output/tandem_repeats<br><br>**# Step 7**<br>echo 'The output of TRF is stored<br>at ./experiments/case8.2/output/tandem_repeats. This file contains a list of tandem repeats, their location on the reference genome, and other details about the repeats. This information can be used for further analysis.'

PacBio DNA sequencing data analysis **Case 9.1: Genome assembly**

<table>
<tr><td>User’s Inputs</td><td>Data and Description
sample_pacbio_reads.fastq: Raw PacBio sequencing data in fastq format.
eference_genome.fasta: A reference genome in FASTA format, if a closely related reference is available and used as a guide during the assembly

Goal
To assemble a genome from the PacBio sequencing data.</td></tr>
<tr><td>Generated Plans by AutoBA</td><td>1. Load the raw PacBio sequencing data from './experiments/case9.1/data/sample_pacbio_reads.fastq'.
2. Load the reference genome from './experiments/case9.1/data/reference_genome.fasta'.
3. Use the software 'Canu' for the genome assembly. This software is specifically designed for long-read sequencing data and does not violate the software restrictions.
4. Configure 'Canu' to use the PacBio data by setting the 'genomeSize' parameter to an estimate of the genome size and the 'pacbio-raw' option to the path of the raw data file.
5. Run 'Canu' to assemble the genome. This will produce a set of contigs, which are sequences of DNA that together represent the genome.
6. Validate the assembly using the software 'Quast'. This software will compare the assembled genome to the reference genome and provide statistics on the quality of the assembly.
7. If the assembly is of sufficient quality, save the assembled genome to a file. If not, adjust the parameters in 'Canu' and repeat the assembly process.</td></tr>
<tr><td>Generated Codes by AutoBA</td><td># Step 1-7
source activate abc
conda config --add channels conda-forge
conda config --add channels bioconda
conda install -y canu

# Load the raw PacBio sequencing data
pacbio_data='./experiments/case9.1/data/sample_pacbio_reads.fastq'
echo $pacbio_data
# Load the reference genome
reference_genome='./experiments/case9.1/data/reference_genome.fasta'

Use Canu for genome assembly
canu -p pacbio -d ./experiments/case9.1/output genomeSize=500m -pacbio-raw $pacbio_data

conda install -y quast

Validate the assembly using Quast
quast ./experiments/case9.1/output/pacbio.contigs.fasta -r $reference_genome -o ./experiments/case9.1/output/quast_report

# Check the quality of the assembly
quast_quality=$(cat ./experiments/case9.1/output/quast_report/report.txt | grep "Total length (>= 0 bp)" | awk '{print $4}')</td></tr>
</table>

	# If the assembly is of sufficient quality, save the assembled genome to a file<br>if [$quast_quality -ge 500000000]; then<br><br>cp ./experiments/case9.1/output/pacbio.contigs.fasta ./experiments/case9.1/output/final_assembly.fasta<br>else<br># If not, adjust the parameters in Canu and repeat the assembly process<br>canu -p pacbio -d ./experiments/case9.1/output genomeSize=600m -pacbio-raw $pacbio_data<br>fi

RNA-Seq Analysis **Case 10.1: Find differentially expressed genes**

<table>
<tr><td>User’s Inputs</td><td>Data and Description
SRR1374921.fastq.gz: single-end mouse rna-seq reads, replicate 1 in LoGlu group
SRR1374922.fastq.gz: single-end mouse rna-seq reads, replicate 2 in LoGlu group
SRR1374923.fastq.gz: single-end mouse rna-seq reads, replicate 1 in HiGlu group
SRR1374924.fastq.gz: single-end mouse rna-seq reads, replicate 2 in HiGlu group
TruSeq3-SE.fa: trimming adapter
mm39.fa: mouse mm39 genome fasta
mm39.ncbiRefSeq.gtf: mouse mm39 genome annotation

Goal
Find the differentially expressed genes</td></tr>
<tr><td>Generated Plans by AutoBA</td><td>1. First, I will use the 'Trimmomatic' software to trim the adapters from the single-end mouse rna-seq reads. The input files for this step will be './examples/case1.1/data/SRR1374921.fastq.gz', './examples/case1.1/data/SRR1374922.fastq.gz', './examples/case1.1/data/SRR1374923.fastq.gz', './examples/case1.1/data/SRR1374924.fastq.gz' and the adapter file './examples/case1.1/data/TruSeq3-SE.fa’.
2. Next, I will use the 'Hisat2' software to align the trimmed reads to the mouse mm39 genome. The input files for this step will be the output files from the previous step and the genome file './examples/case1.1/data/mm39.fa’.
3. Then, I will use the 'Samtools' software to convert the alignment output files from Hisat2 (in SAM format) to BAM format.
4. After that, I will use the ‘HTSeq’ software to count the number of reads mapped to each gene. The input files for this step will be the BAM files from the previous step and the annotation file './examples/case1.1/data/mm39.ncbiRefSeq.gtf’.
5. Finally, I will use the 'DESeq2' software to identify the differentially expressed genes. The input files for this step will be the count files from the previous step.</td></tr>
<tr><td>Generated Codes by AutoBA</td><td># Step 1
source activate abc
conda install -y trimmomatic
trimmomatic SE -phred33 ./examples/case1.1/data/SRR1374921.fastq.gz ./examples/case1.1/output/SRR1374921_trimmed.fastq.gz ILLUMINACLIP:./examples/case1.1/data/TruSeq3-SE.fa:2:30:10
trimmomatic SE -phred33 ./examples/case1.1/data/SRR1374922.fastq.gz ./examples/case1.1/output/SRR1374922_trimmed.fastq.gz ILLUMINACLIP:./examples/case1.1/data/TruSeq3-SE.fa:2:30:10
trimmomatic SE -phred33 ./examples/case1.1/data/SRR1374923.fastq.gz ./examples/case1.</td></tr>
</table>

```
1/output/SRR1374923_trimmed.fastq.gz
ILLUMINACLIP:./examples/case1.1/data/TruSeq3-SE.fa:2:30:10
trimmomatic SE -
phred33 ./examples/case1.1/data/SRR1374924.fastq.gz ./examples/case1.
1/output/SRR1374924_trimmed.fastq.gz
ILLUMINACLIP:./examples/case1.1/data/TruSeq3-SE.fa:2:30:10

# Step 2
source activate abc
conda install -y hisat2
conda install -y hisat2
hisat2-
build ./examples/case1.1/data/mm39.fa ./examples/case1.1/data/mm39
hisat2 -x ./examples/case1.1/data/mm39 -
U ./examples/case1.1/output/SRR1374921_trimmed.fastq.gz -
S ./examples/case1.1/output/SRR1374921.sam hisat2 -
x ./examples/case1.1/data/mm39 -
U ./examples/case1.1/output/SRR1374922_trimmed.fastq.gz -
S ./examples/case1.1/output/SRR1374922.sam hisat2 -
x ./examples/case1.1/data/mm39 -
U ./examples/case1.1/output/SRR1374923_trimmed.fastq.gz -
S ./examples/case1.1/output/SRR1374923.sam hisat2 -
x ./examples/case1.1/data/mm39 -
U ./examples/case1.1/output/SRR1374924_trimmed.fastq.gz -
S ./examples/case1.1/output/SRR1374924.sam

# Step 3
source activate abc
conda install -y samtools
samtools view -S -
b ./examples/case1.1/output/SRR1374921.sam > ./examples/case1.1/outpu
t/SRR1374921.bam
samtools view -S -
b ./examples/case1.1/output/SRR1374922.sam > ./examples/case1.1/outpu
t/SRR1374922.bam
samtools view -S -
b ./examples/case1.1/output/SRR1374923.sam > ./examples/case1.1/outpu
t/SRR1374923.bam
samtools view -S -
b ./examples/case1.1/output/SRR1374924.sam > ./examples/case1.1/outpu
t/SRR1374924.bam

# Step 4
source activate abc
conda install -y htseq
htseq-count -f bam -s
no ./examples/case1.1/output/SRR1374921.bam ./examples/case1.1/data/
mm39.ncbiRefSeq.gtf > ./examples/case1.1/output/SRR1374921.count
htseq-count -f bam -s
no ./examples/case1.1/output/SRR1374922.bam ./examples/case1.1/data/
mm39.ncbiRefSeq.gtf > ./examples/case1.1/output/SRR1374922.count
```

	htseq-count -f bam -s no ./examples/case1.1/output/SRR1374923.bam ./examples/case1.1/data/mm39.ncbiRefSeq.gtf > ./examples/case1.1/output/SRR1374923.count<br>htseq-count -f bam -s no ./examples/case1.1/output/SRR1374924.bam ./examples/case1.1/data/mm39.ncbiRefSeq.gtf > ./examples/case1.1/output/SRR1374924.count<br><br>**# Step 5**<br>source activate abc<br>conda install -y bioconductor-deseq2<br>Rscript -e “library(‘DESeq2’); countData1 <- read.table('./examples/case1.1/output/SRR1374921.count', header=TRUE, row.names=1); countData2 <- read.table('./examples/case1.1/output/SRR1374922.count', header=TRUE, row.names=1); countData3 <- read.table('./examples/case1.1/output/SRR1374923.count', header=TRUE, row.names=1); countData4 <- read.table('./examples/case1.1/output/SRR1374924.count', header=TRUE, row.names=1); countData <- cbind(countData1, countData2, countData3, countData4); colData <- DataFrame(condition=factor(c('LoGlu', 'LoGlu', 'HiGlu', 'HiGlu'))); dds <- DESeqDataSetFromMatrix(countData=countData, colData=colData, design=~condition); dds <- DESeq(dds); res <- results(dds); write.csv(as.data.frame(res), file='./examples/case1.1/output/differential_expression_results.csv')"

RNA-Seq Analysis   Case 10.2: Identify top5 down-regulated genes in HiGlu group

<table>
<tr><td>User’s Inputs</td><td>Data and Description
SRR1374921.fastq.gz: single-end mouse rna-seq reads, replicate 1 in LoGlu group
SRR1374922.fastq.gz: single-end mouse rna-seq reads, replicate 2 in LoGlu group
SRR1374923.fastq.gz: single-end mouse rna-seq reads, replicate 1 in HiGlu group
SRR1374924.fastq.gz: single-end mouse rna-seq reads, replicate 2 in HiGlu group
TruSeq3-SE.fa: trimming adapter
mm39.fa: mouse mm39 genome fasta
mm39.ncbiRefSeq.gtf: mouse mm39 genome annotation

Goal
Identify top5 down-regulated genes in HiGlu group</td></tr>
<tr><td>Generated Plans by AutoBA</td><td>1. First, we will use the 'Trimmomatic' software to trim the adapters from the RNA-seq reads. The input for this step will be the RNA-seq reads files ('./examples/case1.2/data/SRR1374921.fastq.gz', './examples/case1.2/data/SRR1374922.fastq.gz', './examples/case1.2/data/SRR1374923.fastq.gz', './examples/case1.2/data/SRR1374924.fastq.gz') and the trimming adapter file ('./examples/case1.2/data/TruSeq3-SE.fa').
2. Next, we will use the 'Hisat2' software to align the trimmed reads to the mouse mm39 genome. The input for this step will be the trimmed reads from the previous step and the mouse mm39 genome fasta file ('./examples/case1.2/data/mm39.fa').
3. After alignment, we will use the 'Samtools' software to convert the alignment output to a sorted BAM file. The input for this step will be the alignment output from the previous step.
4. Then, we will use the 'HTSeq' software to count the number of reads mapped to each gene. The input for this step will be the sorted BAM file from the previous step and the mouse mm39 genome annotation file ('./examples/case1.2/data/mm39.ncbiRefSeq.gtf').
5. After obtaining the read counts, we will use the 'DESeq2' software to perform differential gene expression analysis between the LoGlu and HiGlu groups. The input for this step will be the read counts from the previous step.
6. Finally, we will use the 'pheatmap' software to visualize the top 5 down-regulated genes in the HiGlu group. The input for this step will be the differential gene expression results from the previous step.</td></tr>
<tr><td>Generated Codes by AutoBA</td><td># Step 1
source activate abc
conda install -y trimmomatic
trimmomatic SE -phred33 ./examples/case1.2/data/SRR1374921.fastq.gz ./examples/case1.2/output/SRR1374921_trimmed.fastq.gz ILLUMINACLIP:./examples/case1.2/data/TruSeq3-SE.fa:2:30:10
trimmomatic SE -phred33 ./examples/case1.2/data/SRR1374922.fastq.gz ./examples/case1.2/output/SRR1374922_trimmed.fastq.gz ILLUMINACLIP:./examples/case1.2/data/TruSeq3-SE.fa:2:30:10</td></tr>
</table>

	trimmomatic SE -<br>phred33 ./examples/case1.2/data/SRR1374923.fastq.gz ./examples/case1.2/output/SRR1374923_trimmed.fastq.gz<br>ILLUMINACLIP:./examples/case1.2/data/TruSeq3-SE.fa:2:30:10<br>trimmomatic SE -<br>phred33 ./examples/case1.2/data/SRR1374924.fastq.gz ./examples/case1.2/output/SRR1374924_trimmed.fastq.gz<br>ILLUMINACLIP:./examples/case1.2/data/TruSeq3-SE.fa:2:30:10<br><br>**# Step 2**<br>source activate abc<br>conda install -y hisat2<br>hisat2-<br>build ./examples/case1.1/data/mm39.fa ./examples/case1.1/data/mm39<br>hisat2 -x ./examples/case1.2/data/mm39 -<br>U ./examples/case1.2/output/SRR1374921_trimmed.fastq.gz -<br>S ./examples/case1.2/output/SRR1374921.sam<br>hisat2 -x ./examples/case1.2/data/mm39 -<br>U ./examples/case1.2/output/SRR1374922_trimmed.fastq.gz -<br>S ./examples/case1.2/output/SRR1374922.sam<br>hisat2 -x ./examples/case1.2/data/mm39 -<br>U ./examples/case1.2/output/SRR1374923_trimmed.fastq.gz -<br>S ./examples/case1.2/output/SRR1374923.sam<br>hisat2 -x ./examples/case1.2/data/mm39 -<br>U ./examples/case1.2/output/SRR1374924_trimmed.fastq.gz -<br>S ./examples/case1.2/output/SRR1374924.sam<br><br>**# Step 3**<br>source activate abc<br>conda install -y samtools<br>samtools view -S -<br>b ./examples/case1.2/output/SRR1374921.sam > ./examples/case1.2/output/SRR1374921.bam<br>samtools sort ./examples/case1.2/output/SRR1374921.bam -<br>o ./examples/case1.2/output/SRR1374921_sorted.bam<br>samtools view -S -<br>b ./examples/case1.2/output/SRR1374922.sam > ./examples/case1.2/output/SRR1374922.bam<br>samtools sort ./examples/case1.2/output/SRR1374922.bam -<br>o ./examples/case1.2/output/SRR1374922_sorted.bam<br>samtools view -S -<br>b ./examples/case1.2/output/SRR1374923.sam > ./examples/case1.2/output/SRR1374923.bam<br>samtools sort ./examples/case1.2/output/SRR1374923.bam -<br>o ./examples/case1.2/output/SRR1374923_sorted.bam<br>samtools view -S -<br>b ./examples/case1.2/output/SRR1374924.sam > ./examples/case1.2/output/SRR1374924.bam<br>samtools sort ./examples/case1.2/output/SRR1374924.bam -<br>o ./examples/case1.2/output/SRR1374924_sorted.bam<br><br>**# Step 4**<br>source activate abc

	conda install -y htseq<br>htseq-count -f bam -s<br>no ./examples/case1.2/output/SRR1374921_sorted.bam ./examples/case1.2/data/mm39.ncbiRefSeq.gtf > ./examples/case1.2/output/SRR1374921_counts.txt<br>htseq-count -f bam -s<br>no ./examples/case1.2/output/SRR1374922_sorted.bam ./examples/case1.2/data/mm39.ncbiRefSeq.gtf > ./examples/case1.2/output/SRR1374922_counts.txt<br>htseq-count -f bam -s<br>no ./examples/case1.2/output/SRR1374923_sorted.bam ./examples/case1.2/data/mm39.ncbiRefSeq.gtf > ./examples/case1.2/output/SRR1374923_counts.txt<br>htseq-count -f bam -s<br>no ./examples/case1.2/output/SRR1374924_sorted.bam ./examples/case1.2/data/mm39.ncbiRefSeq.gtf > ./examples/case1.2/output/SRR1374924_counts.txt<br><br>**# Step 5**<br>source activate abc<br>conda install -y -c bioconda bioconductor-deseq2<br>Rscript -e “library(‘DESeq2’); countData1 <- read.table('./examples/case1.2/output/SRR1374921.count', header=TRUE, row.names=1); countData2 <- read.table('./examples/case1.2/output/SRR1374922.count', header=TRUE, row.names=1); countData3 <- read.table('./examples/case1.2/output/SRR1374923.count', header=TRUE, row.names=1); countData4 <- read.table('./examples/case1.2/output/SRR1374924.count', header=TRUE, row.names=1); countData <- cbind(countData1, countData2, countData3, countData4); colData <- DataFrame(condition=factor(c('LoGlu', 'LoGlu', 'HiGlu', 'HiGlu'))); dds <- DESeqDataSetFromMatrix(countData=countData, colData=colData, design=~condition); dds <- DESeq(dds); res <- results(dds); write.csv(as.data.frame(res), file='./examples/case1.2/output/differential_expression_results.csv')"<br><br>**# Step 6**<br>source activate abc<br>conda install -y -c conda-forge r-pheatmap<br>Rscript -e "library('pheatmap'); library('DESeq2'); res <- read.csv('./examples/case1.2/output/differential_expression_results.csv', row.names=1); res_ordered <- res[order(res$log2FoldChange),]; top5_downregulated <- head(res_ordered, 5); pheatmap(top5_downregulated)"

RNA-Seq Analysis **Case 10.3: Predict Fusion genes**

**User's Inputs**	**Data and Description**<br>rnaseq_1.fastq.gz: RNA-Seq read 1 data (left read)<br>rnaseq_2.fastq.gz: RNA-Seq read 2 data (right read)<br>CTAT_HumanFusionLib.mini.dat.gz: a small fusion annotation library<br>minigenome.fa: small genome sequence consisting of ~750 genes<br>minigenome.gtf: transcript structure annotations for these genes<br><br>**Goal**<br>Predict Fusion genes
**Generated Plans by AutoBA**	1. Load the RNA-Seq read 1 data (left read) from './examples/case1.3/data/rnaseq_1.fastq.gz<br>2. Load the RNA-Seq read 2 data (right read) from './examples/case1.3/data/rnaseq_2.fastq.gz<br>3. Load the small fusion annotation library from './examples/case1.3/data/CTAT_HumanFusionLib.mini.dat.gz'<br>4. Load the small genome sequence consisting of ~750 genes from './examples/case1.3/data/minigenome.fa'.<br>5. Load the transcript structure annotations for these genes from './examples/case1.3/data/minigenome.gtf'.<br>6. Use a software alternative to STAR and java, such as HISAT2, to align the RNA-Seq reads to the small genome sequence.<br>7. Use the aligned reads and the fusion annotation library to predict fusion genes. This can be done using a software like FusionCatcher.<br>8. Validate the predicted fusion genes using the transcript structure annotations.<br>9. Document the results and the process for future reference and reproducibility.
**Generated Codes by AutoBA**	**# Step 1-5**<br>source activate abc<br>conda install -y gunzip<br>gunzip -<br>c ./examples/case1.3/data/rnaseq_1.fastq.gz > ./examples/case1.3/data/rna<br>seq_1.fastq<br>gunzip -<br>c ./examples/case1.3/data/rnaseq_2.fastq.gz > ./examples/case1.3/data/rna<br>seq_2.fastq<br>gunzip -<br>c ./examples/case1.3/data/CTAT_HumanFusionLib.mini.dat.gz > ./example<br>s/case1.3/data/CTAT_HumanFusionLib.mini.dat<br>gunzip -<br>c ./examples/case1.3/data/minigenome.fa > ./examples/case1.3/data/minig<br>enome<br><br>**# Step 6**<br>source activate abc<br>conda install -y hisat2<br>hisat2-<br>build ./examples/case1.3/data/minigenome ./examples/case1.3/data/minige<br>nome_index<br>hisat2 -x ./examples/case1.3/data/minigenome_index -<br>1 ./examples/case1.3/data/rnaseq_1.fastq -

	2 ./examples/case1.3/data/rnaseq_2.fastq -<br>S ./examples/case1.3/output/aligned.sam<br><br>**# Step 7**<br>source activate abc<br>conda install -y fusioncatcher<br>fusioncatcher -d ./examples/case1.3/data/CTAT_HumanFusionLib.mini.dat -<br>i ./examples/case1.3/output/aligned.sam -<br>o ./examples/case1.3/output/fusioncatcher_out<br><br>**# Step 8**<br>source activate abc<br>conda install -y gffcompare<br>gffcompare -r ./examples/case1.3/data/minigenome.gtf -<br>o ./examples/case1.3/output/gffcompare_out ./examples/case1.3/output/fusi<br>oncatcher_out/final-list_candidate-fusion-genes.GRCh37.gff3<br><br>**# Step 9**<br>source activate abc<br>echo 'The results and the process for future reference and reproducibility<br>are documented in the output directory: ./examples/case1.3/output’

RNA-Seq Analysis   Case 10.4: isoform expression

**User's Inputs**	**Data and Description**<br>SRR14463651_1.fastq.gz: paired end RNA sequencing data from human lymphoma cell, read 1 of lymphoma samples replicate 1<br>SRR14463651_2.fastq.gz: paired end RNA sequencing data from human lymphoma cell, read 2 of lymphoma samples replicate 1<br>SRR14463652_1.fastq.gz: paired end RNA sequencing data from human lymphoma cell, read 1 of lymphoma samples replicate 2<br>SRR14463652_2.fastq.gz: paired end RNA sequencing data from human lymphoma cell, read 2 of lymphoma samples replicate 2<br>SRR14463680_1.fastq.gz: paired end RNA sequencing data from human lymphoma cell, read 1 of control samples replicate 1<br>SRR14463680_2.fastq.gz: paired end RNA sequencing data from human lymphoma cell, read 2 of control samples replicate 1<br>SRR14463681_1.fastq.gz: paired end RNA sequencing data from human lymphoma cell, read 1 of control samples replicate 2<br>SRR14463681_2.fastq.gz: paired end RNA sequencing data from human lymphoma cell, read 2 of control samples replicate 2<br>gencode.v44.transcripts.fa: transcriptome annotation<br>TruSeq3-PE.fa: trimming adapter<br>**Goal**<br>quantify RNA isoform expression based on transcriptome annotation
**Generated Plans by AutoBA**	1. First, we need to trim the adapters from the raw sequencing data. We can use the software 'Trim Galore!' for this task.<br>2. After trimming, we need to align the trimmed reads to the reference transcriptome. We can use the software 'HISAT2' for this task.<br>3. After alignment, we need to sort and index the alignment files. We can use the software 'SAMtools' for this task.<br>4. Finally, we need to quantify the RNA isoform expression. We can use the software 'StringTie' for this task.
**Generated Codes by AutoBA**	**# step 1**<br>source activate abc<br>conda install -y trim-galore<br>trim_galore --paired --adapter ../example_data/TruSeq3-PE.fa ../example_data/case6.2/SRR14463651_1.fastq.gz ../example_data/case6.2/SRR14463651_2.fastq.gz -o ../output/case6.21<br>trim_galore --paired --adapter ../example_data/TruSeq3-PE.fa ../example_data/case6.2/SRR14463652_1.fastq.gz ../example_data/case6.2/SRR14463652_2.fastq.gz -o ../output/case6.21<br>trim_galore --paired --adapter ../example_data/TruSeq3-PE.fa ../example_data/case6.2/SRR14463680_1.fastq.gz ../example_data/case6.2/SRR14463680_2.fastq.gz -o ../output/case6.21<br>trim_galore --paired --adapter ../example_data/TruSeq3-PE.fa ../example_data/case6.2/SRR14463681_1.fastq.gz ../example_data/case6.2/SRR14463681_2.fastq.gz -o ../output/case6.21<br>**# step 2**<br>source activate abc<br>conda install -y hisat2<br>hisat2 -x ../example_data/case6.2/gencode.v44.transcripts.fa -1 ../output/case6.21/SRR14463651_1_val_1.fq.gz -2 ../output/case6.21/SRR14463651_2_val_2.fq.gz -S ../output/case6.21/SRR14463651.sam

	hisat2 -x ../example_data/case6.2/gencode.v44.transcripts.fa -<br>1 ../output/case6.21/SRR14463652_1_val_1.fq.gz -<br>2 ../output/case6.21/SRR14463652_2_val_2.fq.gz -<br>S ../output/case6.21/SRR14463652.sam<br>hisat2 -x ../example_data/case6.2/gencode.v44.transcripts.fa -<br>1 ../output/case6.21/SRR14463680_1_val_1.fq.gz -<br>2 ../output/case6.21/SRR14463680_2_val_2.fq.gz -<br>S ../output/case6.21/SRR14463680.sam<br>hisat2 -x ../example_data/case6.2/gencode.v44.transcripts.fa -<br>1 ../output/case6.21/SRR14463681_1_val_1.fq.gz -<br>2 ../output/case6.21/SRR14463681_2_val_2.fq.gz -<br>S ../output/case6.21/SRR14463681.sam<br>**# step 3**<br>source activate abc<br>conda install -y samtools<br>samtools sort ../output/case6.21/SRR14463651.sam -<br>o ../output/case6.21/SRR14463651_sorted.bam<br>samtools index ../output/case6.21/SRR14463651_sorted.bam<br>samtools sort ../output/case6.21/SRR14463652.sam -<br>o ../output/case6.21/SRR14463652_sorted.bam<br>samtools index ../output/case6.21/SRR14463652_sorted.bam<br>samtools sort ../output/case6.21/SRR14463680.sam -<br>o ../output/case6.21/SRR14463680_sorted.bam<br>samtools index ../output/case6.21/SRR14463680_sorted.bam<br>samtools sort ../output/case6.21/SRR14463681.sam -<br>o ../output/case6.21/SRR14463681_sorted.bam<br>samtools index ../output/case6.21/SRR14463681_sorted.bam<br>**# step 4**<br>source activate abc<br>conda install -y stringtie<br>stringtie ../output/case6.21/SRR14463651_sorted.bam -<br>G ../example_data/case6.2/gencode.v44.transcripts.fa -<br>o ../output/case6.21/SRR14463651.gtf<br>stringtie ../output/case6.21/SRR14463652_sorted.bam -<br>G ../example_data/case6.2/gencode.v44.transcripts.fa -<br>o ../output/case6.21/SRR14463652.gtf<br>stringtie ../output/case6.21/SRR14463680_sorted.bam -<br>G ../example_data/case6.2/gencode.v44.transcripts.fa -<br>o ../output/case6.21/SRR14463680.gtf<br>stringtie ../output/case6.21/SRR14463681_sorted.bam -<br>G ../example_data/case6.2/gencode.v44.transcripts.fa -<br>o ../output/case6.21/SRR14463681.gtf

RNA-seq Analysis   Case 10.5: splicing analysis

<table>
<tr><td>User's Inputs</td><td>Data and Description
SRR14463651_1.fastq.gz: paired end RNA sequencing data from human lymphoma cell, read 1 of lymphoma samples replicate 1
SRR14463651_2.fastq.gz: paired end RNA sequencing data from human lymphoma cell, read 2 of lymphoma samples replicate 1
SRR14463652_1.fastq.gz: paired end RNA sequencing data from human lymphoma cell, read 1 of lymphoma samples replicate 2
SRR14463652_2.fastq.gz: paired end RNA sequencing data from human lymphoma cell, read 2 of lymphoma samples replicate 2
SRR14463680_1.fastq.gz: paired end RNA sequencing data from human lymphoma cell, read 1 of control samples replicate 1
SRR14463680_2.fastq.gz: paired end RNA sequencing data from human lymphoma cell, read 2 of control samples replicate 1
SRR14463681_1.fastq.gz: paired end RNA sequencing data from human lymphoma cell, read 1 of control samples replicate 2
SRR14463681_2.fastq.gz: paired end RNA sequencing data from human lymphoma cell, read 2 of control samples replicate 2
hg38.fa: reference genome
gencode.v38.annotation.gtf: gene annotation
TruSeq3-PE.fa: trimming adapter
Goal
identify alternative splicing events in lymphoma and control samples and compare them to get differentially spliced events</td></tr>
<tr><td>Generated Plans by AutoBA</td><td>1. First, we need to quality control and trim the adapters from the raw sequencing data.
2. Next, we need to align the trimmed reads to the reference genome. We can use the software 'HISAT2' for this step.
3. After alignment, we need to sort and index the alignment files. We can use the software 'Samtools' for this step.
4. Next, we need to assemble the transcripts. We can use the software 'StringTie' for this step.
5. After assembling the transcripts, we need to prepare the count matrix. We can use the software 'featureCounts' for this step. 'gencode.v38.annotation.gtf' file for the gene annotation.
6. Finally, we can conduct the differential splicing analysis. We can use the software 'rMATS' for this step.</td></tr>
<tr><td>Generated Codes by AutoBA</td><td># Step 1:
source activate abc

Install FastQC and Trimmomatic
conda install -y fastqc trimmomatic

Quality control and trimming adapters
fastqc ../example_data/case6.2/SRR14463651_1.fastq.gz -o ../output/case6.33
fastqc ../example_data/case6.2/SRR14463651_2.fastq.gz -o ../output/case6.33
trimmomatic PE ../example_data/case6.2/SRR14463651_1.fastq.gz ../example_data/case6.2/SRR14463651_2.fastq.gz ../output/case6.33/SRR14463651_1.trimmed.fastq.gz ../output/case6.33/SRR14463651_2.trimmed.fastq.gz ILLUMINACLIP:../example_data/TruSeq3-PE.fa:2:30:10</td></tr>
</table>

	fastqc ../example_data/case6.2/SRR14463652_1.fastq.gz -<br>o ../output/case6.33<br>fastqc ../example_data/case6.2/SRR14463652_2.fastq.gz -<br>o ../output/case6.33<br>trimmomatic<br>PE ../example_data/case6.2/SRR14463652_1.fastq.gz ../example_data/case6.2/SRR14463652_2.fastq.gz ../output/case6.33/SRR14463652_1.trimmed.fastq.gz ../output/case6.33/SRR14463652_2.trimmed.fastq.gz<br>ILLUMINACLIP:../example_data/TruSeq3-PE.fa:2:30:10<br><br>fastqc ../example_data/case6.2/SRR14463680_1.fastq.gz -<br>o ../output/case6.33<br>fastqc ../example_data/case6.2/SRR14463680_2.fastq.gz -<br>o ../output/case6.33<br>trimmomatic<br>PE ../example_data/case6.2/SRR14463680_1.fastq.gz ../example_data/case6.2/SRR14463680_2.fastq.gz ../output/case6.33/SRR14463680_1.trimmed.fastq.gz ../output/case6.33/SRR14463680_2.trimmed.fastq.gz<br>ILLUMINACLIP:../example_data/TruSeq3-PE.fa:2:30:10<br><br>fastqc ../example_data/case6.2/SRR14463681_1.fastq.gz -<br>o ../output/case6.33<br>fastqc ../example_data/case6.2/SRR14463681_2.fastq.gz -<br>o ../output/case6.33<br>trimmomatic<br>PE ../example_data/case6.2/SRR14463681_1.fastq.gz ../example_data/case6.2/SRR14463681_2.fastq.gz ../output/case6.33/SRR14463681_1.trimmed.fastq.gz ../output/case6.33/SRR14463681_2.trimmed.fastq.gz<br>ILLUMINACLIP:../example_data/TruSeq3-PE.fa:2:30:10<br><br>**# step 2**<br>source activate abc<br><br>conda install -y hisat2<br>hisat2 -x ../example_data/hg38.intg.virg.fa -<br>1 ../output/case6.3/SRR14463651_1_val_1.fq.gz -<br>2 ../output/case6.3/SRR14463651_2_val_2.fq.gz -<br>S ../output/case6.3/SRR14463651.sam<br>hisat2 -x ../example_data/hg38.intg.virg.fa -<br>1 ../output/case6.3/SRR14463652_1_val_1.fq.gz -<br>2 ../output/case6.3/SRR14463652_2_val_2.fq.gz -<br>S ../output/case6.3/SRR14463652.sam<br>hisat2 -x ../example_data/hg38.intg.virg.fa -<br>1 ../output/case6.3/SRR14463680_1_val_1.fq.gz -<br>2 ../output/case6.3/SRR14463680_2_val_2.fq.gz -<br>S ../output/case6.3/SRR14463680.sam<br>hisat2 -x ../example_data/hg38.intg.virg.fa -<br>1 ../output/case6.3/SRR14463681_1_val_1.fq.gz -<br>2 ../output/case6.3/SRR14463681_2_val_2.fq.gz -<br>S ../output/case6.3/SRR14463681.sam<br><br>**# step 3**

	source activate abc<br><br># Install Samtools<br>conda install -y samtools<br><br># Sort and index the alignment files<br>samtools sort ../output/case6.33/SRR14463651.sam -<br>o ../output/case6.33/SRR14463651.sorted.bam<br>samtools index ../output/case6.33/SRR14463651.sorted.bam<br><br>samtools sort ../output/case6.33/SRR14463652.sam -<br>o ../output/case6.33/SRR14463652.sorted.bam<br>samtools index ../output/case6.33/SRR14463652.sorted.bam<br><br>samtools sort ../output/case6.33/SRR14463680.sam -<br>o ../output/case6.33/SRR14463680.sorted.bam<br>samtools index ../output/case6.33/SRR14463680.sorted.bam<br><br>samtools sort ../output/case6.33/SRR14463681.sam -<br>o ../output/case6.33/SRR14463681.sorted.bam<br>samtools index ../output/case6.33/SRR14463681.sorted.bam<br><br>**# step 4**<br>source activate abc<br>conda install -y stringtie<br># Assemble the aligned reads into transcripts<br>stringtie -p 8 -G ../example_data/gencode.v38.annotation.gtf -<br>o ../output/case6.3/SRR14463651.gtf ../output/case6.3/SRR14463651.sam<br>stringtie -p 8 -G ../example_data/gencode.v38.annotation.gtf -<br>o ../output/case6.3/SRR14463652.gtf ../output/case6.3/SRR14463652.sam<br>stringtie -p 8 -G ../example_data/gencode.v38.annotation.gtf -<br>o ../output/case6.3/SRR14463680.gtf ../output/case6.3/SRR14463680.sam<br>stringtie -p 8 -G ../example_data/gencode.v38.annotation.gtf -<br>o ../output/case6.3/SRR14463681.gtf ../output/case6.3/SRR14463681.sam<br><br>**# step 5**<br>source activate abc<br><br># Install featureCounts<br>conda install -y subread<br><br># Prepare the count matrix<br>featureCounts -a ../example_data/gencode.v38.annotation.gtf -<br>o ../output/case6.33/SRR14463651.counts ../output/case6.33/SRR1446365<br>1.sorted.bam<br>featureCounts -a ../example_data/gencode.v38.annotation.gtf -<br>o ../output/case6.33/SRR14463652.counts ../output/case6.33/SRR1446365<br>2.sorted.bam<br>featureCounts -a ../example_data/gencode.v38.annotation.gtf -<br>o ../output/case6.33/SRR14463680.counts ../output/case6.33/SRR1446368<br>0.sorted.bam

	featureCounts -a ../example_data/gencode.v38.annotation.gtf -<br>o ../output/case6.33/SRR14463681.counts ../output/case6.33/SRR1446368<br>1.sorted.bam<br><br>**# step 6**<br>source activate abc<br><br># Install rMATS<br>conda install -y rmats<br><br># Conduct the differential splicing analysis<br>rmats.py --<br>b1 ../output/case6.33/SRR14463651.sorted.bam,../output/case6.33/SRR14<br>463652.sorted.bam --<br>b2 ../output/case6.33/SRR14463680.sorted.bam,../output/case6.33/SRR14<br>463681.sorted.bam --gtf ../example_data/gencode.v38.annotation.gtf --<br>od ../output/case6.33 --tmp ../output/case6.33/tmp

RNA-seq Analysis **Case 10.6: APA analysis**

<table>
<tr><td>User's Inputs</td><td>Data and Description
SRR14463651_1.fastq.gz: paired end RNA sequencing data from human lymphoma cell, read 1 of lymphoma samples replicate 1
SRR14463651_2.fastq.gz: paired end RNA sequencing data from human lymphoma cell, read 2 of lymphoma samples replicate 1
SRR14463652_1.fastq.gz: paired end RNA sequencing data from human lymphoma cell, read 1 of lymphoma samples replicate 2
SRR14463652_2.fastq.gz: paired end RNA sequencing data from human lymphoma cell, read 2 of lymphoma samples replicate 2
SRR14463680_1.fastq.gz: paired end RNA sequencing data from human lymphoma cell, read 1 of control samples replicate 1
SRR14463680_2.fastq.gz: paired end RNA sequencing data from human lymphoma cell, read 2 of control samples replicate 1
SRR14463681_1.fastq.gz: paired end RNA sequencing data from human lymphoma cell, read 1 of control samples replicate 2
SRR14463681_2.fastq.gz: paired end RNA sequencing data from human lymphoma cell, read 2 of control samples replicate 2
hg38.fa: reference genome
gencode.v38.annotation.gtf: gene annotation
TruSeq3-PE.fa: trimming adapter
Goal
identify and quantify alternative polyadenylation between lymphoma and control samples</td></tr>
<tr><td>Generated Plans by AutoBA</td><td>1. First, we will use the software 'Trim Galore' to trim the adapters from the raw sequencing data.
2. Next, we will use the software 'HISAT2' to align the trimmed reads to the reference genome.
3. After alignment, we will use the software 'StringTie' to assemble the aligned reads into transcripts.
4. Then, we will use the software 'StringTie' again to quantify the expression levels of the assembled transcripts.
5. Finally, we will use the software 'DaPars' to identify and quantify alternative polyadenylation between lymphoma and control samples.</td></tr>
<tr><td>Generated Codes by AutoBA</td><td># step 1
source activate abc
Install Trim Galore
conda install -y trim-galore
Trim adapters for each file
trim_galore --paired --adapter ../example_data/TruSeq3-PE.fa ../example_data/case6.2/SRR14463651_1.fastq.gz ../example_data/case6.2/SRR14463651_2.fastq.gz -o ../output/case6.4
trim_galore --paired --adapter ../example_data/TruSeq3-PE.fa ../example_data/case6.2/SRR14463652_1.fastq.gz ../example_data/case6.2/SRR14463652_2.fastq.gz -o ../output/case6.4
trim_galore --paired --adapter ../example_data/TruSeq3-PE.fa ../example_data/case6.2/SRR14463680_1.fastq.gz ../example_data/case6.2/SRR14463680_2.fastq.gz -o ../output/case6.4
trim_galore --paired --adapter ../example_data/TruSeq3-PE.fa ../example_data/case6.2/SRR14463681_1.fastq.gz ../example_data/case6.2/SRR14463681_2.fastq.gz -o ../output/case6.4
step 2
source activate abc</td></tr>
</table>

	# Install HISAT2<br>conda install -y hisat2<br># Align the trimmed reads to the reference genome<br>hisat2 -x ../example_data/hg38.intg.virg.fa -<br>1 ../output/case6.4/SRR14463651_1_val_1.fq.gz -<br>2 ../output/case6.4/SRR14463651_2_val_2.fq.gz -<br>S ../output/case6.4/SRR14463651.sam<br>hisat2 -x ../example_data/hg38.intg.virg.fa -<br>1 ../output/case6.4/SRR14463652_1_val_1.fq.gz -<br>2 ../output/case6.4/SRR14463652_2_val_2.fq.gz -<br>S ../output/case6.4/SRR14463652.sam<br>hisat2 -x ../example_data/hg38.intg.virg.fa -<br>1 ../output/case6.4/SRR14463680_1_val_1.fq.gz -<br>2 ../output/case6.4/SRR14463680_2_val_2.fq.gz -<br>S ../output/case6.4/SRR14463680.sam<br>hisat2 -x ../example_data/hg38.intg.virg.fa -<br>1 ../output/case6.4/SRR14463681_1_val_1.fq.gz -<br>2 ../output/case6.4/SRR14463681_2_val_2.fq.gz -<br>S ../output/case6.4/SRR14463681.sam<br>**# step 3**<br>source activate abc<br># Install StringTie<br>conda install -y stringtie<br># Assemble the aligned reads into transcripts<br>stringtie ../output/case6.4/SRR14463651.sam -<br>G ../example_data/gencode.v38.annotation.gtf -<br>o ../output/case6.4/SRR14463651.gtf<br>stringtie ../output/case6.4/SRR14463652.sam -<br>G ../example_data/gencode.v38.annotation.gtf -<br>o ../output/case6.4/SRR14463652.gtf<br>stringtie ../output/case6.4/SRR14463680.sam -<br>G ../example_data/gencode.v38.annotation.gtf -<br>o ../output/case6.4/SRR14463680.gtf<br>stringtie ../output/case6.4/SRR14463681.sam -<br>G ../example_data/gencode.v38.annotation.gtf -<br>o ../output/case6.4/SRR14463681.gtf<br>**# step 4**<br>source activate abc<br># Quantify the expression levels of the assembled transcripts<br>stringtie -e -B -p 8 -G ../output/case6.4/SRR14463651.gtf -<br>o ../output/case6.4/SRR14463651_abundance.gtf ../output/case6.4/SRR14<br>463651.sam<br>stringtie -e -B -p 8 -G ../output/case6.4/SRR14463652.gtf -<br>o ../output/case6.4/SRR14463652_abundance.gtf ../output/case6.4/SRR14<br>463652.sam<br>stringtie -e -B -p 8 -G ../output/case6.4/SRR14463680.gtf -<br>o ../output/case6.4/SRR14463680_abundance.gtf ../output/case6.4/SRR14<br>463680.sam<br>stringtie -e -B -p 8 -G ../output/case6.4/SRR14463681.gtf -<br>o ../output/case6.4/SRR14463681_abundance.gtf ../output/case6.4/SRR14<br>463681.sam<br>**# step 5**<br># Activate the environment

	source activate abc<br># Install DaPars<br>conda install -y dapars<br># Identify and quantify alternative polyadenylation<br>DaPars -i ../output/case6.4/SRR14463651_abundance.gtf -o ../output/case6.4/SRR14463651_DaPars_result.txt<br>DaPars -i ../output/case6.4/SRR14463652_abundance.gtf -o ../output/case6.4/SRR14463652_DaPars_result.txt<br>DaPars -i ../output/case6.4/SRR14463680_abundance.gtf -o ../output/case6.4/SRR14463680_DaPars_result.txt<br>DaPars -i ../output/case6.4/SRR14463681_abundance.gtf -o ../output/case6.4/SRR14463681_DaPars_result.txt

RNA-seq Analysis Case 10.7: RNA editing

**User's Inputs**	**Data and Description**<br>SRR14463651_1.fastq.gz: paired end RNA sequencing data from human lymphoma cell, read 1 of lymphoma samples replicate 1<br>SRR14463651_2.fastq.gz: paired end RNA sequencing data from human lymphoma cell, read 2 of lymphoma samples replicate 1<br>SRR14463652_1.fastq.gz: paired end RNA sequencing data from human lymphoma cell, read 1 of lymphoma samples replicate 2<br>SRR14463652_2.fastq.gz: paired end RNA sequencing data from human lymphoma cell, read 2 of lymphoma samples replicate 2<br>SRR14463680_1.fastq.gz: paired end RNA sequencing data from human lymphoma cell, read 1 of control samples replicate 1<br>SRR14463680_2.fastq.gz: paired end RNA sequencing data from human lymphoma cell, read 2 of control samples replicate 1<br>SRR14463681_1.fastq.gz: paired end RNA sequencing data from human lymphoma cell, read 1 of control samples replicate 2<br>SRR14463681_2.fastq.gz: paired end RNA sequencing data from human lymphoma cell, read 2 of control samples replicate 2<br>hg38.fa: reference genome<br>gencode.v38.annotation.gtf: gene annotation<br>TruSeq3-PE.fa: trimming adapter<br>**Goal**<br>identifying RNA edting events in lymphoma and control samples
**Generated Plans by AutoBA**	1. First, we will use the software 'FastQC' to perform quality control checks on raw sequence data coming from high throughput sequencing pipelines.<br>2. Next, we will use the software 'Trimmomatic' to trim or remove the adapters and low quality bases from the sequence data.<br>3. After trimming, we will use the software 'Hisat2' to align the trimmed reads to the reference genome<br>4. The aligned reads will then be sorted and indexed using the software 'Samtools'.<br>5. The software 'StringTie' will be used to assemble the aligned reads into transcripts and estimate their abundances. The gene annotation file '../example_data/gencode.v38.annotation.gtf' will be used for this purpose.", "6. The software 'Ballgown' will be used to perform differential expression analysis between the lymphoma and control samples.", "7. Finally, the software 'GATK' will be used to identify RNA editing events in the lymphoma and control samples. The identified RNA editing events will be annotated using the gene annotation file '../example_data/gencode.v38.annotation.gtf'."
**Generated Codes by AutoBA**	**# step 1**<br>source activate abc<br>conda install -y fastqc<br>fastqc ../example_data/case6.2/SRR14463651_1.fastq.gz -<br>o ../output/case6.5<br>fastqc ../example_data/case6.2/SRR14463651_2.fastq.gz -<br>o ../output/case6.5<br>fastqc ../example_data/case6.2/SRR14463652_1.fastq.gz -<br>o ../output/case6.5<br>fastqc ../example_data/case6.2/SRR14463652_2.fastq.gz -<br>o ../output/case6.5<br>fastqc ../example_data/case6.2/SRR14463680_1.fastq.gz -<br>o ../output/case6.5

	fastqc ../example_data/case6.2/SRR14463680_2.fastq.gz -<br>o ../output/case6.5<br>fastqc ../example_data/case6.2/SRR14463681_1.fastq.gz -<br>o ../output/case6.5<br>fastqc ../example_data/case6.2/SRR14463681_2.fastq.gz -<br>o ../output/case6.5<br>**# step 2**<br>source activate abc<br>conda install -y trimmomatic<br>trimmomatic PE -<br>phred33 ../example_data/case6.2/SRR14463651_1.fastq.gz ../example_dat<br>a/case6.2/SRR14463651_2.fastq.gz ../output/case6.5/SRR14463651_1.trim<br>med.fastq.gz ../output/case6.5/SRR14463651_2.trimmed.fastq.gz<br>ILLUMINACLIP:../example_data/TruSeq3-PE.fa:2:30:10<br>trimmomatic PE -<br>phred33 ../example_data/case6.2/SRR14463652_1.fastq.gz ../example_dat<br>a/case6.2/SRR14463652_2.fastq.gz ../output/case6.5/SRR14463652_1.trim<br>med.fastq.gz ../output/case6.5/SRR14463652_2.trimmed.fastq.gz<br>ILLUMINACLIP:../example_data/TruSeq3-PE.fa:2:30:10<br>trimmomatic PE -<br>phred33 ../example_data/case6.2/SRR14463680_1.fastq.gz ../example_dat<br>a/case6.2/SRR14463680_2.fastq.gz ../output/case6.5/SRR14463680_1.trim<br>med.fastq.gz ../output/case6.5/SRR14463680_2.trimmed.fastq.gz<br>ILLUMINACLIP:../example_data/TruSeq3-PE.fa:2:30:10<br>trimmomatic PE -<br>phred33 ../example_data/case6.2/SRR14463681_1.fastq.gz ../example_dat<br>a/case6.2/SRR14463681_2.fastq.gz ../output/case6.5/SRR14463681_1.trim<br>med.fastq.gz ../output/case6.5/SRR14463681_2.trimmed.fastq.gz<br>ILLUMINACLIP:../example_data/TruSeq3-PE.fa:2:30:10<br>**# step 3**<br>source activate abc<br>conda install -y hisat2<br>hisat2 -x ../example_data/hg38.intg.virg.fa -<br>1 ../output/case6.5/SRR14463651_1.trimmed.fastq.gz -<br>2 ../output/case6.5/SRR14463651_2.trimmed.fastq.gz -<br>S ../output/case6.5/SRR14463651.sam<br>hisat2 -x ../example_data/hg38.intg.virg.fa -<br>1 ../output/case6.5/SRR14463652_1.trimmed.fastq.gz -<br>2 ../output/case6.5/SRR14463652_2.trimmed.fastq.gz -<br>S ../output/case6.5/SRR14463652.sam<br>hisat2 -x ../example_data/hg38.intg.virg.fa -<br>1 ../output/case6.5/SRR14463680_1.trimmed.fastq.gz -<br>2 ../output/case6.5/SRR14463680_2.trimmed.fastq.gz -<br>S ../output/case6.5/SRR14463680.sam<br>hisat2 -x ../example_data/hg38.intg.virg.fa -<br>1 ../output/case6.5/SRR14463681_1.trimmed.fastq.gz -<br>2 ../output/case6.5/SRR14463681_2.trimmed.fastq.gz -<br>S ../output/case6.5/SRR14463681.sam<br>**# step 4**<br>source activate abc<br>conda install -y samtools<br>samtools sort ../output/case6.5/SRR14463651.sam -<br>o ../output/case6.5/SRR14463651.sorted.bam

```
samtools index ../output/case6.5/SRR14463651.sorted.bam
samtools sort ../output/case6.5/SRR14463652.sam -
o ../output/case6.5/SRR14463652.sorted.bam
samtools index ../output/case6.5/SRR14463652.sorted.bam
samtools sort ../output/case6.5/SRR14463680.sam -
o ../output/case6.5/SRR14463680.sorted.bam
samtools index ../output/case6.5/SRR14463680.sorted.bam
samtools sort ../output/case6.5/SRR14463681.sam -
o ../output/case6.5/SRR14463681.sorted.bam
samtools index ../output/case6.5/SRR14463681.sorted.bam
# step 5
source activate abc
conda install -y stringtie
stringtie -p 8 -G ../example_data/gencode.v38.annotation.gtf -
o ../output/case6.5/SRR14463651.gtf ../output/case6.5/SRR14463651.sorte
d.bam
stringtie -p 8 -G ../example_data/gencode.v38.annotation.gtf -
o ../output/case6.5/SRR14463652.gtf ../output/case6.5/SRR14463652.sorte
d.bam
stringtie -p 8 -G ../example_data/gencode.v38.annotation.gtf -
o ../output/case6.5/SRR14463680.gtf ../output/case6.5/SRR14463680.sorte
d.bam
stringtie -p 8 -G ../example_data/gencode.v38.annotation.gtf -
o ../output/case6.5/SRR14463681.gtf ../output/case6.5/SRR14463681.sorte
d.bam
# step 6
source activate abc
conda install -y ballgown
Rscript -e "library(ballgown); data_directory = '../output/case6.5';
sample_pattern = 'SRR'; bg = ballgown(dataDir=data_directory,
samplePattern=sample_pattern); results_genes=stattest(bg, feature='gene',
covariate='group', getFC=TRUE, meas='FPKM');
results_transcripts=stattest(bg, feature='transcript', covariate='group',
getFC=TRUE, meas='FPKM'); write.table(results_genes,
file='../output/case6.5/gene_results.tsv', sep='	');
write.table(results_transcripts, file='../output/case6.5/transcript_results.tsv',
sep='	')"
# step 7
source activate abc
conda install -y gatk
# Identify RNA editing events
gatk HaplotypeCaller -R ../example_data/hg38.intg.virg.fa -
I ../output/case6.5/SRR14463651.sorted.bam -
O ../output/case6.5/SRR14463651.vcf
# Annotate RNA editing events
gatk Funcotator -R ../example_data/hg38.intg.virg.fa -
V ../output/case6.5/SRR14463651.vcf -
O ../output/case6.5/SRR14463651.funcotated.vcf --ref-version hg38 --data-
sources-path ../example_data/gencode.v38.annotation.gtf
# Repeat for other samples

# Identify RNA editing events
```

	gatk HaplotypeCaller -R ../example_data/hg38.intg.virg.fa -<br>I ../output/case6.5/SRR14463652.sorted.bam -<br>O ../output/case6.5/SRR14463652.vcf<br><br># Annotate RNA editing events<br>gatk Funcotator -R ../example_data/hg38.intg.virg.fa -<br>V ../output/case6.5/SRR14463652.vcf -<br>O ../output/case6.5/SRR14463652.funcotated.vcf --ref-version hg38 --data-sources-path ../example_data/gencode.v38.annotation.gtf<br><br># Identify RNA editing events<br>gatk HaplotypeCaller -R ../example_data/hg38.intg.virg.fa -<br>I ../output/case6.5/SRR14463680.sorted.bam -<br>O ../output/case6.5/SRR14463680.vcf<br><br># Annotate RNA editing events<br>gatk Funcotator -R ../example_data/hg38.intg.virg.fa -<br>V ../output/case6.5/SRR14463680.vcf -<br>O ../output/case6.5/SRR14463680.funcotated.vcf --ref-version hg38 --data-sources-path ../example_data/gencode.v38.annotation.gtf<br><br># Identify RNA editing events<br>gatk HaplotypeCaller -R ../example_data/hg38.intg.virg.fa -<br>I ../output/case6.5/SRR14463681.sorted.bam -<br>O ../output/case6.5/SRR14463681.vcf<br><br># Annotate RNA editing events<br>gatk Funcotator -R ../example_data/hg38.intg.virg.fa -<br>V ../output/case6.5/SRR14463681.vcf -<br>O ../output/case6.5/SRR14463681.funcotated.vcf --ref-version hg38 --data-sources-path ../example_data/gencode.v38.annotation.gtf

RNA-seq Analysis Case 10.8: circular RNA analysis

<table>
<tr><td>User’s Inputs</td><td>Data and Description
Data and Description
SRR14463651_1.fastq.gz: paired end RNA sequencing data from human lymphoma cell, read 1 of lymphoma samples replicate 1
SRR14463651_2.fastq.gz: paired end RNA sequencing data from human lymphoma cell, read 2 of lymphoma samples replicate 1
SRR14463652_1.fastq.gz: paired end RNA sequencing data from human lymphoma cell, read 1 of lymphoma samples replicate 2
SRR14463652_2.fastq.gz: paired end RNA sequencing data from human lymphoma cell, read 2 of lymphoma samples replicate 2
SRR14463680_1.fastq.gz: paired end RNA sequencing data from human lymphoma cell, read 1 of control samples replicate 1
SRR14463680_2.fastq.gz: paired end RNA sequencing data from human lymphoma cell, read 2 of control samples replicate 1
SRR14463681_1.fastq.gz: paired end RNA sequencing data from human lymphoma cell, read 1 of control samples replicate 2
SRR14463681_2.fastq.gz: paired end RNA sequencing data from human lymphoma cell, read 2 of control samples replicate 2
hg38.fa: reference genome
gencode.v38.annotation.gtf: gene annotation
TruSeq3-PE.fa: trimming adapter
Goal
identify and quantify circular RNA in lymphoma and control samples</td></tr>
<tr><td>Generated Plans by AutoBA</td><td>1. First, we will use the software 'Trim Galore' to trim the adapters from the raw sequencing data.
2. Next, we will use the software 'HISAT2' to align the trimmed reads to the reference genome.
3. After alignment, we will use the software 'CIRI2' to identify circular RNAs from the aligned reads.
4. Finally, we will use the software 'CIRIquant' to quantify the identified circular RNAs.</td></tr>
<tr><td>Generated Codes by AutoBA</td><td># step 1
source activate abc
Install Trim Galore
conda install -y trim-galore
Trim adapters for each file
trim_galore --paired --illumina ../example_data/case6.2/SRR14463651_1.fastq.gz ../example_data/case6.2/SRR14463651_2.fastq.gz -o ../output/case6.6
trim_galore --paired --illumina ../example_data/case6.2/SRR14463652_1.fastq.gz ../example_data/case6.2/SRR14463652_2.fastq.gz -o ../output/case6.6
trim_galore --paired --illumina ../example_data/case6.2/SRR14463680_1.fastq.gz ../example_data/case6.2/SRR14463680_2.fastq.gz -o ../output/case6.6
trim_galore --paired --illumina ../example_data/case6.2/SRR14463681_1.fastq.gz ../example_data/case6.2/SRR14463681_2.fastq.gz -o ../output/case6.6
step 2
source activate abc
Install HISAT2
conda install -y hisat2</td></tr>
</table>

	# Align the trimmed reads to the reference genome<br>hisat2 -x ../example_data/hg38.intg.virg.fa -<br>1 ../output/case6.6/SRR14463651_1_val_1.fq.gz -<br>2 ../output/case6.6/SRR14463651_2_val_2.fq.gz -<br>S ../output/case6.6/SRR14463651.sam<br>hisat2 -x ../example_data/hg38.intg.virg.fa -<br>1 ../output/case6.6/SRR14463652_1_val_1.fq.gz -<br>2 ../output/case6.6/SRR14463652_2_val_2.fq.gz -<br>S ../output/case6.6/SRR14463652.sam<br>hisat2 -x ../example_data/hg38.intg.virg.fa -<br>1 ../output/case6.6/SRR14463680_1_val_1.fq.gz -<br>2 ../output/case6.6/SRR14463680_2_val_2.fq.gz -<br>S ../output/case6.6/SRR14463680.sam<br>hisat2 -x ../example_data/hg38.intg.virg.fa -<br>1 ../output/case6.6/SRR14463681_1_val_1.fq.gz -<br>2 ../output/case6.6/SRR14463681_2_val_2.fq.gz -<br>S ../output/case6.6/SRR14463681.sam<br>**# step 3**<br>source activate abc<br># Install CIRI2<br>conda install -y ciri2<br># Identify circular RNAs from the aligned reads<br>CIRI2 -I ../output/case6.6/SRR14463651.sam -<br>O ../output/case6.6/SRR14463651_ciri2_output -<br>F ../example_data/hg38.intg.virg.fa -<br>A ../example_data/gencode.v38.annotation.gtf<br>CIRI2 -I ../output/case6.6/SRR14463652.sam -<br>O ../output/case6.6/SRR14463652_ciri2_output -<br>F ../example_data/hg38.intg.virg.fa -<br>A ../example_data/gencode.v38.annotation.gtf<br>CIRI2 -I ../output/case6.6/SRR14463680.sam -<br>O ../output/case6.6/SRR14463680_ciri2_output -<br>F ../example_data/hg38.intg.virg.fa -<br>A ../example_data/gencode.v38.annotation.gtf<br>CIRI2 -I ../output/case6.6/SRR14463681.sam -<br>O ../output/case6.6/SRR14463681_ciri2_output -<br>F ../example_data/hg38.intg.virg.fa -<br>A ../example_data/gencode.v38.annotation.gtf<br>**# step 4**<br>source activate abc<br># Install CIRIquant<br>conda install -y CIRIquant<br># Quantify the identified circular RNAs<br>CIRIquant -i ../output/case6.6/SRR14463651_ciri2_output -<br>o ../output/case6.6/SRR14463651_ciriquant_output -<br>a ../example_data/gencode.v38.annotation.gtf<br>CIRIquant -i ../output/case6.6/SRR14463652_ciri2_output -<br>o ../output/case6.6/SRR14463652_ciriquant_output -<br>a ../example_data/gencode.v38.annotation.gtf<br>CIRIquant -i ../output/case6.6/SRR14463680_ciri2_output -<br>o ../output/case6.6/SRR14463680_ciriquant_output -<br>a ../example_data/gencode.v38.annotation.gtf

	CIRIquant -i ../output/case6.6/SRR14463681_ciri2_output -<br>o ../output/case6.6/SRR14463681_ciriquant_output -<br>a ../example_data/gencode.v38.annotation.gtf

Small RNA-seq Analysis **Case 11.1 and Case 11.2: miRNA analysis**

<table>
<tr><td>User's Inputs</td><td>Data and Description
SRR10915820.fastq.gz: single end small RNA sequencing data from human cell line, control samples replicate 1
SRR10915821.fastq.gz: single end small RNA sequencing data from human cell line, control samples replicate 1
SRR10915822.fastq.gz: single end small RNA sequencing data from human cell line, control samples replicate 1
SRR10915823.fastq.gz: single end small RNA sequencing data from human cell line, control samples replicate 1
hsa.gff3: microRNA genomic coordinate from miRbase
TGGAATTCTCGGGTGCCAAGG: adaptor sequence
hg38.fa: the reference genome

Goal
quantify microRNA expression based on miRbase and predict novel microRNA</td></tr>
<tr><td>Generated Plans by AutoBA</td><td>1. Validate the provided files
2. Use 'FastQC' software to perform quality control checks on raw sequence data coming from high throughput sequencing pipelines.
3. Use 'Cutadapt' software to remove the provided adaptor sequence 'TGGAATTCTCGGGTGCCAAGG' from the raw data.
4. Use 'Bowtie' software to align the trimmed reads to the reference genome 'hg38.fa'.
5. Use 'Samtools' software to convert the alignment output to sorted BAM files.
6. Use 'featureCounts' software to quantify the microRNA expression based on 'hsa.gff3' from miRbase.
7. Use 'miRDeep2' software to predict novel microRNA from the sorted BAM files and the reference genome 'hg38.fa'.
8. Use 'DESeq2' software to perform differential expression analysis between control and treatment samples.
9. Use 'edgeR' software to validate the differential expression results from 'DESeq2'.</td></tr>
<tr><td>Generated Codes by AutoBA</td><td># step 1
if [-f './examples/case6.1/SRR10915820.fastq.gz'] && [-f './examples/case6.1/SRR10915821.fastq.gz'] && [-f './examples/case6.1/SRR10915822.fastq.gz'] && [-f './examples/case6.1/SRR10915823.fastq.gz'] && [-f 'hsa.gff3'] && [-f 'hg38.fa']; then
   echo 'All files are valid.'
else
   echo 'Some files are missing.'
fi
step 2
source activate abc
conda install -y fastqc
fastqc ./examples/case6.1/SRR10915820.fastq.gz -o ../output/case7.3
fastqc ./examples/case6.1/SRR10915821.fastq.gz -o ../output/case7.3
fastqc ./examples/case6.1/SRR10915822.fastq.gz -o ../output/case7.3
fastqc ./examples/case6.1/SRR10915823.fastq.gz -o ../output/case7.3

step 3</td></tr>
</table>

	source activate abc<br>conda install -y cutadapt<br>cutadapt -a TGGAATTCTCGGGTGCCAAGG -<br>o ../output/case7.3/SRR10915820_trimmed.fastq.gz ./examples/case6.1/SR<br>R10915820.fastq.gz<br>cutadapt -a TGGAATTCTCGGGTGCCAAGG -<br>o ../output/case7.3/SRR10915821_trimmed.fastq.gz ./examples/case6.1/SR<br>R10915821.fastq.gz<br>cutadapt -a TGGAATTCTCGGGTGCCAAGG -<br>o ../output/case7.3/SRR10915822_trimmed.fastq.gz ./examples/case6.1/SR<br>R10915822.fastq.gz<br>cutadapt -a TGGAATTCTCGGGTGCCAAGG -<br>o ../output/case7.3/SRR10915823_trimmed.fastq.gz ./examples/case6.1/SR<br>R10915823.fastq.gz<br>**# step 4**<br>source activate abc<br>conda install -y bowtie<br>bowtie-build hg38.fa hg38<br>bowtie -q -v 2 -p 4 -S -m 1 --best --strata<br>hg38 ../output/case7.3/SRR10915820_trimmed.fastq.gz > ../output/case7.3/<br>SRR10915820.sam<br>bowtie -q -v 2 -p 4 -S -m 1 --best --strata<br>hg38 ../output/case7.3/SRR10915821_trimmed.fastq.gz > ../output/case7.3/<br>SRR10915821.sam<br>bowtie -q -v 2 -p 4 -S -m 1 --best --strata<br>hg38 ../output/case7.3/SRR10915822_trimmed.fastq.gz > ../output/case7.3/<br>SRR10915822.sam<br>bowtie -q -v 2 -p 4 -S -m 1 --best --strata<br>hg38 ../output/case7.3/SRR10915823_trimmed.fastq.gz > ../output/case7.3/<br>SRR10915823.sam<br>**# step 5**<br>source activate abc<br>conda install -y samtools<br>samtools view -S -<br>b ../output/case7.3/SRR10915820.sam > ../output/case7.3/SRR10915820.b<br>am<br>samtools sort ../output/case7.3/SRR10915820.bam -<br>o ../output/case7.3/SRR10915820_sorted.bam<br>samtools view -S -<br>b ../output/case7.3/SRR10915821.sam > ../output/case7.3/SRR10915821.b<br>am<br>samtools sort ../output/case7.3/SRR10915821.bam -<br>o ../output/case7.3/SRR10915821_sorted.bam<br>samtools view -S -<br>b ../output/case7.3/SRR10915822.sam > ../output/case7.3/SRR10915822.b<br>am<br>samtools sort ../output/case7.3/SRR10915822.bam -<br>o ../output/case7.3/SRR10915822_sorted.bam<br>samtools view -S -<br>b ../output/case7.3/SRR10915823.sam > ../output/case7.3/SRR10915823.b<br>am<br>samtools sort ../output/case7.3/SRR10915823.bam -<br>o ../output/case7.3/SRR10915823_sorted.bam

```
# step 6
source activate abc
conda install -y subread
featureCounts -a hsa.gff3 -
o ../output/case7.3/SRR10915820_counts.txt ../output/case7.3/SRR109158
20_sorted.bam
featureCounts -a hsa.gff3 -
o ../output/case7.3/SRR10915821_counts.txt ../output/case7.3/SRR109158
21_sorted.bam
featureCounts -a hsa.gff3 -
o ../output/case7.3/SRR10915822_counts.txt ../output/case7.3/SRR109158
22_sorted.bam
featureCounts -a hsa.gff3 -
o ../output/case7.3/SRR10915823_counts.txt ../output/case7.3/SRR109158
23_sorted.bam

# step 7
source activate abc
conda install -y mirdeep2
miRDeep2.pl ../output/case7.3/SRR10915820_sorted.bam
hg38.fa ../output/case7.3/SRR10915820_counts.txt none none hsa
2>../output/case7.3/SRR10915820_mirdeep2.log
miRDeep2.pl ../output/case7.3/SRR10915821_sorted.bam
hg38.fa ../output/case7.3/SRR10915821_counts.txt none none hsa
2>../output/case7.3/SRR10915821_mirdeep2.log
miRDeep2.pl ../output/case7.3/SRR10915822_sorted.bam
hg38.fa ../output/case7.3/SRR10915822_counts.txt none none hsa
2>../output/case7.3/SRR10915822_mirdeep2.log
miRDeep2.pl ../output/case7.3/SRR10915823_sorted.bam
hg38.fa ../output/case7.3/SRR10915823_counts.txt none none hsa
2>../output/case7.3/SRR10915823_mirdeep2.log
# step 8
source activate abc
conda install -y bioconductor-deseq2
Rscript -e "library(DESeq2); control <-
read.table('../output/case7.3/SRR10915820_counts.txt', header=TRUE,
row.names=1); treatment <-
read.table('../output/case7.3/SRR10915822_counts.txt', header=TRUE,
row.names=1); dds <- DESeqDataSetFromMatrix(countData =
cbind(control, treatment), colData = data.frame(condition = c('control',
'treatment')), design = ~ condition); dds <- DESeq(dds); res <- results(dds);
write.csv(res, file='../output/case7.3/differential_expression_results.csv')"

Rscript -e "library(DESeq2); control <-
read.table('../output/case7.3/SRR10915821_counts.txt', header=TRUE,
row.names=1); treatment <-
read.table('../output/case7.3/SRR10915823_counts.txt', header=TRUE,
row.names=1); dds <- DESeqDataSetFromMatrix(countData =
cbind(control, treatment), colData = data.frame(condition = c('control',
'treatment')), design = ~ condition); dds <- DESeq(dds); res <- results(dds);
write.csv(res,
file='../output/case7.3/differential_expression_results_replicate.csv')"
# step 9
```

	source activate abc<br>conda install -y bioconductor-edger<br><br>Rscript -e "library(edgeR); control <- read.table('../output/case7.3/SRR10915820_counts.txt', header=TRUE, row.names=1); treatment <- read.table('../output/case7.3/SRR10915822_counts.txt', header=TRUE, row.names=1); group <- factor(c('control', 'treatment')); y <- DGEList(counts=cbind(control, treatment), group=group); y <- calcNormFactors(y); design <- model.matrix(~group); y <- estimateDisp(y, design); fit <- glmQLFit(y, design); qlf <- glmQLFTest(fit, coef=2); topTags(qlf)"<br><br>Rscript -e "library(edgeR); control <- read.table('../output/case7.3/SRR10915821_counts.txt', header=TRUE, row.names=1); treatment <- read.table('../output/case7.3/SRR10915823_counts.txt', header=TRUE, row.names=1); group <- factor(c('control', 'treatment')); y <- DGEList(counts=cbind(control, treatment), group=group); y <- calcNormFactors(y); design <- model.matrix(~group); y <- estimateDisp(y, design); fit <- glmQLFit(y, design); qlf <- glmQLFTest(fit, coef=2); topTags(qlf)"

CAGE-seq Analysis   Case 12.1: TSS analysis

**User's Inputs**	**Data and Description**<br>SRR11351697.fastq.gz: CAGE-seq data from human cell line, control samples replicate 1<br>SRR11351698.fastq.gz: CAGE-seq data from human cell line, control samples replicate 2<br>SRR11351700.fastq.gz: CAGE-seq data from human cell line, treatment samples replicate 1<br>SRR11351701.fastq.gz: CAGE-seq data from human cell line, treatment samples replicate 1<br>hg38.fa: the human reference genome<br>gencode.v37.annotation.gtf: gene annotation<br>TruSeq3-SE.fa: trimming adapter<br><br>**Goal**<br>Identify transcription start site (TSS) in the cell line based on CAGE-seq data
**Generated Plans by AutoBA**	1. First, we will use the 'FastQC' software to perform quality control checks on the raw sequence data.<br>2. Next, we will use the 'Trimmomatic' software to trim the low-quality bases and adapter sequences from the raw data.<br>3. After that, we will use the 'Hisat2' software to align the trimmed reads to the human reference genome.<br>4. Then, we will use the 'HTSeq' software to count the number of reads mapped to each gene.<br>5. Finally, we will use the 'CAGEr' package in R to identify the transcription start sites (TSS) based on the CAGE-seq data.
**Generated Codes by AutoBA**	**# step 1**<br>source activate abc<br># Install FastQC with conda<br>conda install -y fastqc<br># Run FastQC on each input file<br>fastqc ../example_data/case8.1/SRR11351697.fastq.gz -o ../output/case8.2<br>fastqc ../example_data/case8.1/SRR11351698.fastq.gz -o ../output/case8.2<br>fastqc ../example_data/case8.1/SRR11351700.fastq.gz -o ../output/case8.2<br>fastqc ../example_data/case8.1/SRR11351701.fastq.gz -o ../output/case8.2<br><br>**# step 2**<br>source activate abc<br># Install Trimmomatic with conda<br>conda install -y trimmomatic<br># Run Trimmomatic on each input file<br>trimmomatic SE -phred33 ../example_data/case8.1/SRR11351697.fastq.gz ../output/case8.2/SRR11351697_trimmed.fastq.gz ILLUMINACLIP:../example_data/TruSeq3-SE.fa:2:30:10<br>trimmomatic SE -phred33 ../example_data/case8.1/SRR11351698.fastq.gz ../output/case8.2/SRR11351698_trimmed.fastq.gz ILLUMINACLIP:../example_data/TruSeq3-SE.fa:2:30:10<br>trimmomatic SE -phred33 ../example_data/case8.1/SRR11351700.fastq.gz ../output/case8.2/

```
SRR11351700_trimmed.fastq.gz ILLUMINACLIP:../example_data/TruSeq3-
SE.fa:2:30:10
trimmomatic SE -
phred33 ../example_data/case8.1/SRR11351701.fastq.gz ../output/case8.2/
SRR11351701_trimmed.fastq.gz ILLUMINACLIP:../example_data/TruSeq3-
SE.fa:2:30:10

# step 3
conda install -y hisat2
# Build the Hisat2 index for the reference genome
hisat2-build ../example_data/case8.1/hg38.fa hg38_index
# Run Hisat2 on each input file
hisat2 -x hg38_index -U ../output/case8.2/SRR11351697_trimmed.fastq.gz -
S ../output/case8.2/SRR11351697_aligned.sam
hisat2 -x hg38_index -U ../output/case8.2/SRR11351698_trimmed.fastq.gz -
S ../output/case8.2/SRR11351698_aligned.sam
hisat2 -x hg38_index -U ../output/case8.2/SRR11351700_trimmed.fastq.gz -
S ../output/case8.2/SRR11351700_aligned.sam
hisat2 -x hg38_index -U ../output/case8.2/SRR11351701_trimmed.fastq.gz -
S ../output/case8.2/SRR11351701_aligned.sam

# step 4
source activate abc
# Install HTSeq with conda
conda install -y htseq
# Run HTSeq on each input file
htseq-count -f sam -s
no ../output/case8.2/SRR11351697_aligned.sam ../example_data/case8.1/g
encode.v37.annotation.gtf > ../output/case8.2/SRR11351697_counts.txt
htseq-count -f sam -s
no ../output/case8.2/SRR11351698_aligned.sam ../example_data/case8.1/g
encode.v37.annotation.gtf > ../output/case8.2/SRR11351698_counts.txt
htseq-count -f sam -s
no ../output/case8.2/SRR11351700_aligned.sam ../example_data/case8.1/g
encode.v37.annotation.gtf > ../output/case8.2/SRR11351700_counts.txt
htseq-count -f sam -s
no ../output/case8.2/SRR11351701_aligned.sam ../example_data/case8.1/g
encode.v37.annotation.gtf > ../output/case8.2/SRR11351701_counts.txt

# step 5
source activate abc
# Install R and CAGEr with conda
conda install -y r r-cager
# Run CAGEr on each input file
Rscript -e "library(CAGEr); setwd('../output/case8.2'); cager_obj <-
new('CAGEset', genomeName = 'hg38', inputFiles =
c('SRR11351697_counts.txt', 'SRR11351698_counts.txt',
'SRR11351700_counts.txt', 'SRR11351701_counts.txt'), inputFilesType =
'counts', sampleLabels = c('control_1', 'control_2', 'treatment_1',
'treatment_2')); cager_obj <- normalizeTagCount(cager_obj); cager_obj <-
clusterCTSS(cager_obj); cager_obj <- identifyTSS(cager_obj);
write.table(getTSS(cager_obj), file = 'TSS.txt', sep = '\t', quote = FALSE,
row.names = FALSE)"
```

3'end-seq Analysis   Case 13.1: APA analysis based on 3'end-seq data

<table>
<tr><td>User's Inputs</td><td>Data and Description
SRR17422754.fastq.gz: Lexongen QuantSeq 3 prime-end RNA sequencing data from human cell line THLE2, healthy liver cell line replicate 1
SRR17422755.fastq.gz: Lexongen QuantSeq 3 prime-end RNA sequencing data from human cell line THLE2, healthy liver cell line replicate 2
SRR17422756.fastq.gz: Lexongen QuantSeq 3 prime-end RNA sequencing data from human cell line SNU398, liver cancer cell line replicate 1
SRR17422757.fastq.gz: Lexongen QuantSeq 3 prime-end RNA sequencing data from human cell line SNU398, liver cancer cell line replicate 2
hg38.fa: the human reference genome
gencode.v37.annotation.gtf: gene annotation
TruSeq3-SE.fa: trimming adapter

Goal
Identify polyadenylation sites and quantify alternative polyadenylation based on 3'-end sequencing data</td></tr>
<tr><td>Generated Plans by AutoBA</td><td>1. First, we will use the software 'Trim Galore' to trim the adapter sequences from the raw sequencing data.
2. Next, we will use the software 'Hisat2' to align the trimmed reads to the human reference genome.
3. After alignment, we will use the software 'StringTie' to assemble the aligned reads into transcripts.
4. Then, we will use the software 'DaPars' to identify the polyadenylation sites and quantify alternative polyadenylation.
5. Finally, we will use the software 'R' to perform statistical analysis and visualization of the results. The input file for this step is the output file from the previous step.</td></tr>
<tr><td>Generated Codes by AutoBA</td><td># step 1
source activate abc
Install Trim Galore
conda install -y trim-galore
Trim the adapter sequences
trim_galore --adapter ../example_data/TruSeq3-SE.fa --output_dir ../output/case9.2 ../example_data/case9.1/SRR17422754.fastq.gz
trim_galore --adapter ../example_data/TruSeq3-SE.fa --output_dir ../output/case9.2 ../example_data/case9.1/SRR17422755.fastq.gz
trim_galore --adapter ../example_data/TruSeq3-SE.fa --output_dir ../output/case9.2 ../example_data/case9.1/SRR17422756.fastq.gz
trim_galore --adapter ../example_data/TruSeq3-SE.fa --output_dir ../output/case9.2 ../example_data/case9.1/SRR17422757.fastq.gz
step 2
source activate abc
Install Hisat2
conda install -y hisat2
Align the trimmed reads to the human reference genome
hisat2 -x ../example_data/case9.1/hg38.fa -U ../output/case9.2/SRR17422754_trimmed.fq.gz -S ../output/case9.2/SRR17422754.sam</td></tr>
</table>

	hisat2 -x ../example_data/case9.1/hg38.fa -<br>U ../output/case9.2/SRR17422755_trimmed.fq.gz -<br>S ../output/case9.2/SRR17422755.sam<br>hisat2 -x ../example_data/case9.1/hg38.fa -<br>U ../output/case9.2/SRR17422756_trimmed.fq.gz -<br>S ../output/case9.2/SRR17422756.sam<br>hisat2 -x ../example_data/case9.1/hg38.fa -<br>U ../output/case9.2/SRR17422757_trimmed.fq.gz -<br>S ../output/case9.2/SRR17422757.sam<br>**# step 3**<br>source activate abc<br># Install StringTie<br>conda install -y stringtie<br># Assemble the aligned reads into transcripts<br>stringtie -G ../example_data/case9.1/gencode.v37.annotation.gtf -<br>o ../output/case9.2/SRR17422754.gtf ../output/case9.2/SRR17422754.sam<br>stringtie -G ../example_data/case9.1/gencode.v37.annotation.gtf -<br>o ../output/case9.2/SRR17422755.gtf ../output/case9.2/SRR17422755.sam<br>stringtie -G ../example_data/case9.1/gencode.v37.annotation.gtf -<br>o ../output/case9.2/SRR17422756.gtf ../output/case9.2/SRR17422756.sam<br>stringtie -G ../example_data/case9.1/gencode.v37.annotation.gtf -<br>o ../output/case9.2/SRR17422757.gtf ../output/case9.2/SRR17422757.sam<br><br>**# step 4**<br>source activate abc<br># Install DaPars<br>conda install -y dapars<br><br># Identify the polyadenylation sites and quantify alternative polyadenylation<br>DaPars -i ../output/case9.2/SRR17422754.gtf -<br>o ../output/case9.2/SRR17422754_DaPars_result.txt<br>DaPars -i ../output/case9.2/SRR17422755.gtf -<br>o ../output/case9.2/SRR17422755_DaPars_result.txt<br>DaPars -i ../output/case9.2/SRR17422756.gtf -<br>o ../output/case9.2/SRR17422756_DaPars_result.txt<br>DaPars -i ../output/case9.2/SRR17422757.gtf -<br>o ../output/case9.2/SRR17422757_DaPars_result.txt<br><br>**# step 5**<br>source activate abc<br><br># Install R<br>conda install -y r-base<br><br># Perform statistical analysis and visualization of the results<br>Rscript -e "setwd('../output/case9.2'); library(ggplot2); data1 <-<br>read.table('SRR17422754_DaPars_result.txt', header = TRUE); data2 <-<br>read.table('SRR17422755_DaPars_result.txt', header = TRUE); data3 <-<br>read.table('SRR17422756_DaPars_result.txt', header = TRUE); data4 <-<br>read.table('SRR17422757_DaPars_result.txt', header = TRUE);<br>pdf('SRR17422754_DaPars_result.pdf'); ggplot(data1, aes(x=variable,<br>y=value)) + geom_boxplot(); dev.off();<br>pdf('SRR17422755_DaPars_result.pdf'); ggplot(data2, aes(x=variable,

	y=value)) + geom_boxplot(); dev.off(); pdf('SRR17422756_DaPars_result.pdf'); ggplot(data3, aes(x=variable, y=value)) + geom_boxplot(); dev.off(); pdf('SRR17422757_DaPars_result.pdf'); ggplot(data4, aes(x=variable, y=value)) + geom_boxplot(); dev.off();"

ONT sequencing Analysis Case 14.1: isoform expression

<table>
<tr><td>User’s Inputs</td><td>Data and Description
RHL5569-T1_SAECK_shControl_replicate_1.ont.fastq.gz: ONT sequencing data from human cell line, control samples replicate 1
RHL5570-T1_SAECK_shControl_replicate_2.ont.fastq.gz: ONT sequencing data from human cell line, control samples replicate 2
RHL5571-T1_SAECK_shPARP4-1_replicate_1.ont.fastq.gz: ONT sequencing data from human cell line, treatment samples replicate 1
RHL5572-T1_SAECK_shPARP4-1_replicate_2.ont.fastq.gz: ONT sequencing data from human cell line, treatment samples replicate 2
hg38.fa: the human reference genome
gencode.v37.annotation.gtf: gene annotation

Goal
Quantify isoform expression in control and treatment samples</td></tr>
<tr><td>Generated Plans by AutoBA</td><td>1. Load the provided ONT sequencing data files and the human reference genome file into the workspace.
2. Use the software 'Minimap2' to align the ONT sequencing data to the human reference genome.
3. Convert the SAM files to BAM files using 'samtools'.
4. Sort the BAM files using 'samtools sort'.
5. Use the software 'StringTie' to assemble the transcripts for each sample.
6. Use 'StringTie' again to merge all the GTF files into a single file.
7. Use 'StringTie' to estimate the transcript abundances and generate the read count tables.
8. Use 'prepDE.py' script to extract the count matrix from the GTF files.
9. Use 'DESeq2' in R to perform differential expression analysis between control and treatment samples using the count matrix generated in the previous step.
10. Finally, the isoform expression in control and treatment samples will be quantified.</td></tr>
<tr><td>Generated Codes by AutoBA</td><td># step 1
source activate abc && conda install -y minimap2 && conda install -y samtools && conda install -y stringtie

step 2
source activate abc && minimap2 -ax map-ont ../example_data/case10.1/hg38.fa ../example_data/case10.1/RHL5569-T1_SAECK_shControl_replicate_1.ont.fastq.gz > ../output/case10.1/control_replicate_1.sam && minimap2 -ax map-ont ../example_data/case10.1/hg38.fa ../example_data/case10.1/RHL5570-T1_SAECK_shControl_replicate_2.ont.fastq.gz > ../output/case10.1/control_replicate_2.sam && minimap2 -ax map-ont ../example_data/case10.1/hg38.fa ../example_data/case10.1/RHL5571-T1_SAECK_shPARP4-1_replicate_1.ont.fastq.gz > ../output/case10.1/treatment_replicate_1.sam && minimap2 -ax map-ont ../example_data/case10.1/hg38.fa ../example_data/case10.1/RHL5572-T1_SAECK_shPARP4-1_replicate_2.ont.fastq.gz > ../output/case10.1/treatment_replicate_2.sam

step 3</td></tr>
</table>

source activate abc && samtools view -S -
b ../output/case10.1/control_replicate_1.sam > ../output/case10.1/control_re
plicate_1.bam && samtools view -S -
b ../output/case10.1/control_replicate_2.sam > ../output/case10.1/control_re
plicate_2.bam && samtools view -S -
b ../output/case10.1/treatment_replicate_1.sam > ../output/case10.1/treatm
ent_replicate_1.bam && samtools view -S -
b ../output/case10.1/treatment_replicate_2.sam > ../output/case10.1/treatm
ent_replicate_2.bam

# step 4
source activate abc && samtools
sort ../output/case10.1/control_replicate_1.bam -
o ../output/case10.1/control_replicate_1_sorted.bam && samtools
sort ../output/case10.1/control_replicate_2.bam -
o ../output/case10.1/control_replicate_2_sorted.bam && samtools
sort ../output/case10.1/treatment_replicate_1.bam -
o ../output/case10.1/treatment_replicate_1_sorted.bam && samtools
sort ../output/case10.1/treatment_replicate_2.bam -
o ../output/case10.1/treatment_replicate_2_sorted.bam

# step 5
source activate abc && stringtie -p 8 -
G ../example_data/case9.1/gencode.v37.annotation.gtf -
o ../output/case10.1/control_replicate_1.gtf ../output/case10.1/control_replic
ate_1_sorted.bam && stringtie -p 8 -
G ../example_data/case9.1/gencode.v37.annotation.gtf -
o ../output/case10.1/control_replicate_2.gtf ../output/case10.1/control_replic
ate_2_sorted.bam && stringtie -p 8 -
G ../example_data/case9.1/gencode.v37.annotation.gtf -
o ../output/case10.1/treatment_replicate_1.gtf ../output/case10.1/treatment_
replicate_1_sorted.bam && stringtie -p 8 -
G ../example_data/case9.1/gencode.v37.annotation.gtf -
o ../output/case10.1/treatment_replicate_2.gtf ../output/case10.1/treatment_
replicate_2_sorted.bam

# step 6
source activate abc && stringtie --merge -p 8 -
G ../example_data/case9.1/gencode.v37.annotation.gtf -
o ../output/case10.1/merged.gtf ../output/case10.1/control_replicate_1.gtf ../
output/case10.1/control_replicate_2.gtf ../output/case10.1/treatment_replica
te_1.gtf ../output/case10.1/treatment_replicate_2.gtf

# step 7
source activate abc && stringtie -e -B -p 8 -G ../output/case10.1/merged.gtf
-
o ../output/case10.1/control_replicate_1_abund.gtf ../output/case10.1/contro
l_replicate_1_sorted.bam && stringtie -e -B -p 8 -
G ../output/case10.1/merged.gtf -
o ../output/case10.1/control_replicate_2_abund.gtf ../output/case10.1/contro
l_replicate_2_sorted.bam && stringtie -e -B -p 8 -
G ../output/case10.1/merged.gtf -
o ../output/case10.1/treatment_replicate_1_abund.gtf ../output/case10.1/tre

	atment_replicate_1_sorted.bam && stringtie -e -B -p 8 -<br>G ../output/case10.1/merged.gtf -<br>o ../output/case10.1/treatment_replicate_2_abund.gtf ../output/case10.1/tre<br>atment_replicate_2_sorted.bam<br><br>**# step 8**<br>source activate abc && python prepDE.py -i ../output/case10.1 -<br>g ../output/case10.1/gene_count_matrix.csv -<br>t ../output/case10.1/transcript_count_matrix.csv<br><br>**# step 9**<br>source activate abc && conda install -y r && conda install -y -c bioconda<br>bioconductor-deseq2 && Rscript -e "library(DESeq2); countdata <-<br>read.csv('../output/case10.1/gene_count_matrix.csv', header=TRUE,<br>row.names=1); coldata <- data.frame(row.names=colnames(countdata),<br>condition=c('control', 'control', 'treatment', 'treatment')); dds <-<br>DESeqDataSetFromMatrix(countData = countdata, colData = coldata,<br>design = ~ condition); dds <- DESeq(dds); res <- results(dds); write.csv(res,<br>file='../output/case10.1/differential_expression_results.csv')"<br><br>**# step 10**<br>source activate abc && Rscript -e "library(DESeq2); countdata <-<br>read.csv('../output/case10.1/transcript_count_matrix.csv', header=TRUE,<br>row.names=1); coldata <- data.frame(row.names=colnames(countdata),<br>condition=c('control', 'control', 'treatment', 'treatment')); dds <-<br>DESeqDataSetFromMatrix(countData = countdata, colData = coldata,<br>design = ~ condition); dds <- DESeq(dds); res <- results(dds); write.csv(res,<br>file='../output/case10.1/isoform_expression_results.csv')"

PacBio iso-seq Analysis   Case 15.1: isoform analysis

<table>
<tr><td>User's Inputs</td><td>Data and Description
SRR19552218.fastq.gz: pacbio sequecning data from human cell line, cell line HCT-116
SRR19785215.fastq.gz: pacbio sequecning data from human cell line, cell line HepG2
hg38.fa: the human reference genome
gencode.v37.annotation.gtf: gene annotation

Goal
identify novel isoform and quantify isoform expression in two cell lines</td></tr>
<tr><td>Generated Plans by AutoBA</td><td>1. Load the provided PacBio sequencing data files for the HCT-116 and HepG2 cell lines.
2. Load the human reference genome file
3. Load the gene annotation file located
4. Use the 'Minimap2' software to align the sequencing data to the human reference genome. This step will generate a BAM file for each cell line.
5. Use the 'StringTie' software to assemble the aligned reads into transcripts for each cell line. This step will generate a GTF file for each cell line.
6. Use the 'Cuffcompare' software to compare the assembled transcripts with the gene annotation. This step will identify novel isoforms in each cell line.
7. Use the 'Cuffquant' software to quantify the expression of each isoform in each cell line. This step will generate a Cuffdiff output file for each cell line.
8. Use the 'Cuffdiff' software to compare the isoform expression between the two cell lines. This step will generate a final report that includes the novel isoforms and their expression in each cell line.</td></tr>
<tr><td>Generated Codes by AutoBA</td><td># step 1
source activate abc
Load the PacBio sequencing data files for the HCT-116 and HepG2 cell lines
zcat ../example_data/case10.1/SRR19552218.fastq.gz > HCT-116.fastq
zcat ../example_data/case10.1/SRR19785215.fastq.gz > HepG2.fastq

step 2
source activate abc
Load the human reference genome file
cp ../example_data/case10.1/hg38.fa .

step 3
source activate abc
Load the gene annotation file
cp ../example_data/case9.1/gencode.v37.annotation.gtf .

step 4
source activate abc
Install Minimap2 with conda
conda install -y minimap2
Align the HCT-116 sequencing data to the human reference genome using Minimap2
minimap2 -ax map-pb hg38.fa HCT-116.fastq > HCT-116.bam</td></tr>
</table>

	# Align the HepG2 sequencing data to the human reference genome using Minimap2<br>minimap2 -ax map-pb hg38.fa HepG2.fastq > HepG2.bam<br><br>**# step 5**<br>source activate abc<br># Install StringTie with conda<br>conda install -y stringtie<br># Assemble the aligned reads into transcripts for the HCT-116 cell line using StringTie<br>stringtie HCT-116.bam -o HCT-116.gtf<br># Assemble the aligned reads into transcripts for the HepG2 cell line using StringTie<br>stringtie HepG2.bam -o HepG2.gtf<br><br>**# step 6**<br>source activate abc<br># Install Cuffcompare with conda<br>conda install -y cufflinks<br># Compare the assembled transcripts with the gene annotation for the HCT-116 cell line using Cuffcompare<br>cuffcompare -r gencode.v37.annotation.gtf -o HCT-116_cuffcompare HCT-116.gtf<br><br># Compare the assembled transcripts with the gene annotation for the HepG2 cell line using Cuffcompare<br>cuffcompare -r gencode.v37.annotation.gtf -o HepG2_cuffcompare HepG2.gtf<br><br>**# step 7**<br>source activate abc<br># Install Cuffquant with conda<br>conda install -y cufflinks<br># Quantify the expression of each isoform in the HCT-116 cell line using Cuffquant<br>cuffquant -o ../output/case10.2/HCT-116_cuffquant HCT-116_cuffcompare<br># Quantify the expression of each isoform in the HepG2 cell line using Cuffquant<br>cuffquant -o ../output/case10.2/HepG2_cuffquant HepG2_cuffcompare<br><br>**# step 8**<br># Activate the conda environment<br>source activate abc<br># Install Cuffdiff with conda<br>conda install -y cufflinks<br># Compare the isoform expression between the two cell lines using Cuffdiff<br>cuffdiff -o ../output/case10.2/cuffdiff_output ../output/case10.2/HCT-116_cuffquant ../output/case10.2/HepG2_cuffquant

CLIP-seq Analysis Case 16.1: CLIP-seq data analysis

<table>
<tr><td>User's Inputs</td><td>Data and Description
ENCLB742AYH.fastq.gz: single end reads of eCLIP data from HepG2 cell line, SRSF1 targeted sample replicate 1
ENCLB770EDJ.fastq.gz: single end reads of eCLIP data from HepG2 cell line, SRSF1 targeted sample replicate 2
hg38.fa: the human reference genome
gencode.v37.annotation.gtf: gene annotation

Goal
identify SRSF1 protein-RNA crosslink sites based on CLIP sequencing data</td></tr>
<tr><td>Generated Plans by AutoBA</td><td>1. First, we will use the 'FastQC' software to perform quality control on the provided single end reads of CLIP sequencing data from the HepG2 cell line, SRSF1 targeted sample replicate 1 and 2.
2. Next, we will use the 'Cutadapt' software to trim the adapters from the reads.
3. After trimming, we will again use 'FastQC' to check the quality of the trimmed reads.
4. Then, we will use the 'Bowtie2' software to align the trimmed reads to the reference genome.
5. After alignment, we will use the 'Samtools' software to convert the alignment output to a sorted BAM file.
6. Next, we will use the 'BEDTools' software to convert the sorted BAM file to a BED file.
7. Then, we will use the 'BEDTools' software again to intersect the BED file with the gene annotation to identify the reads that overlap with genes.
8. Finally, we will use the 'Piranha' software to identify the SRSF1 protein-RNA crosslink sites based on the intersected reads.</td></tr>
<tr><td>Generated Codes by AutoBA</td><td># step 1
source activate abc
conda config --add channels conda-forge
conda config --add channels bioconda
conda install -y fastqc
fastqc ../example_data/case16/ENCLB742AYH.fastq.gz -o ./output/case16.2
fastqc ../example_data/case16/ENCLB770EDJ.fastq.gz -o ./output/case16.2

step 2
source activate abc
conda config --add channels conda-forge
conda config --add channels bioconda
conda install -y cutadapt
cutadapt -a file:../example_data/TruSeq3-SE.fa -o ./output/case16.2/ENCLB742AYH_trimmed.fastq.gz ../example_data/case16/ENCLB742AYH.fastq.gz
cutadapt -a file:../example_data/TruSeq3-SE.fa -o ./output/case16.2/ENCLB770EDJ_trimmed.fastq.gz ../example_data/case16/ENCLB770EDJ.fastq.gz

step 3
source activate abc
conda config --add channels conda-forge
conda config --add channels bioconda</td></tr>
</table>

```
conda install -y fastqc
fastqc ./output/case16.2/ENCLB742AYH_trimmed.fastq.gz -
o ./output/case16.2
fastqc ./output/case16.2/ENCLB770EDJ_trimmed.fastq.gz -
o ./output/case16.2

# step 4
source activate abc
conda config --add channels conda-forge
conda config --add channels bioconda
conda install -y bowtie2
bowtie2-build ../example_data/hg38.intg.virg.fa hg38
bowtie2 -x hg38 -U ./output/case16.2/ENCLB742AYH_trimmed.fastq.gz -
S ./output/case16.2/ENCLB742AYH.sam
bowtie2 -x hg38 -U ./output/case16.2/ENCLB770EDJ_trimmed.fastq.gz -
S ./output/case16.2/ENCLB770EDJ.sam

# step 5
source activate abc
conda config --add channels conda-forge
conda config --add channels bioconda
conda install -y samtools
samtools view -S -
b ./output/case16.2/ENCLB742AYH.sam > ./output/case16.2/ENCLB742AY
H.bam
samtools sort ./output/case16.2/ENCLB742AYH.bam -
o ./output/case16.2/ENCLB742AYH_sorted.bam
samtools view -S -
b ./output/case16.2/ENCLB770EDJ.sam > ./output/case16.2/ENCLB770ED
J.bam
samtools sort ./output/case16.2/ENCLB770EDJ.bam -
o ./output/case16.2/ENCLB770EDJ_sorted.bam

# step 6
source activate abc
conda config --add channels conda-forge
conda config --add channels bioconda
conda install -y bedtools
bedtools bamtobed -
i ./output/case16.2/ENCLB742AYH_sorted.bam > ./output/case16.2/ENCLB
742AYH.bed
bedtools bamtobed -
i ./output/case16.2/ENCLB770EDJ_sorted.bam > ./output/case16.2/ENCLB
770EDJ.bed

# step 7
source activate abc
conda config --add channels conda-forge
conda config --add channels bioconda
conda install -y bedtools
bedtools intersect -a ./output/case16.2/ENCLB742AYH.bed -
b ../example_data/gencode.v38.annotation.gtf > ./output/case16.2/ENCLB7
42AYH_intersected.bed
```

	bedtools intersect -a ./output/case16.2/ENCLB770EDJ.bed -<br>b ../example_data/gencode.v38.annotation.gtf > ./output/case16.2/ENCLB7<br>70EDJ_intersected.bed<br><br># step 8<br>source activate abc<br>conda config --add channels conda-forge<br>conda config --add channels bioconda<br>conda install -y piranha<br>Piranha -<br>b ./output/case16.2/ENCLB742AYH_intersected.bed > ./output/case16.2/E<br>NCLB742AYH_piranha.bed<br>Piranha -<br>b ./output/case16.2/ENCLB770EDJ_intersected.bed > ./output/case16.2/EN<br>CLB770EDJ_piranha.bed

RIP-Seq Analysis **Case 16.2: Identify enriched genes bounded by RPB**

**User's Inputs**	**Data and Description**<br>rip.rep1.fastq.gz: single end reads of RIP-seq data from HepG2 cell line, pulldown samples of SRSF1 replicate 1<br>rip.rep2.fastq.gz: single end reads of RIP-seq data from HepG2 cell line, pulldown samples of SRSF1 replicate 2<br>controll.rep1.fastq.gz: single end reads of RIP-seq daata from HepG2 cell line, control sample replicate 1<br>controll.rep2.fastq.gz: single end reads of RIP-seq daata from HepG2 cell line, control sample replicate 2<br>hg38.fa: the human reference genome<br>gencode.v37.annotation.gtf: gene annotation<br><br>Goal<br>identify genes enriched in pulldown samples over the control
**Generated Plans by AutoBA**	1. First, we will use the software 'FastQC' to perform quality control checks on the raw sequence data provided in the files 'rip.rep1.fastq.gz', 'rip.rep2.fastq.gz', 'controll.rep1.fastq.gz', and 'control2.rep2.fastq.gz'.<br>2. Next, we will use the software 'Trim Galore' to trim the low-quality bases and adapter sequences from the reads. The adapter sequences are provided in the file 'TruSeq3-SE.fa'.<br>3. After trimming, we will align the reads to the reference genome provided in the file 'hg38.intg.virg.fa' using the software 'HISAT2'.<br>4. Once the reads are aligned, we will use the software 'HTSeq' to count the number of reads mapped to each gene. The gene annotation is provided in the file 'gencode.v38.annotation.gtf'.<br>5. Finally, we will use the software 'DESeq2' to identify the genes that are differentially expressed between the pulldown samples and the control samples. The results will be a list of genes that are enriched in the pulldown samples over the control
**Generated Codes by AutoBA**	**# step 1**<br># Activate the conda environment<br>source activate abc<br>conda config --add channels conda-forge<br>conda config --add channels bioconda<br>conda install -y fastqc<br>fastqc ../example_data/case16/rip.rep1.fastq.gz -o ./output/case16.2<br>fastqc ../example_data/case16/rip.rep2.fastq.gz -o ./output/case16.2<br>fastqc ../example_data/case16/controll.rep1.fastq.gz -o ./output/case16.2<br>fastqc ../example_data/case16/control2.rep2.fastq.gz -o ./output/case16.2<br><br>**# step 2**<br>source activate abc<br>conda config --add channels conda-forge<br>conda config --add channels bioconda<br>conda install -y trim-galore<br>trim_galore --adapter ../example_data/TruSeq3-SE.fa ../example_data/case16/rip.rep1.fastq.gz --output_dir ./output/case16.2<br>trim_galore --adapter ../example_data/TruSeq3-SE.fa ../example_data/case16/rip.rep2.fastq.gz --output_dir ./output/case16.2

```
trim_galore --adapter ../example_data/TruSeq3-
SE.fa ../example_data/case16/controll.rep1.fastq.gz --
output_dir ./output/case16.2
trim_galore --adapter ../example_data/TruSeq3-
SE.fa ../example_data/case16/control2.rep2.fastq.gz --
output_dir ./output/case16.2

# step 3
source activate abc
conda config --add channels conda-forge
conda config --add channels bioconda
conda install -y hisat2
hisat2 -x ../example_data/hg38.intg.virg.fa -
U ./output/case16.2/rip.rep1_trimmed.fq.gz -
S ./output/case16.2/rip.rep1.sam
hisat2 -x ../example_data/hg38.intg.virg.fa -
U ./output/case16.2/rip.rep2_trimmed.fq.gz -
S ./output/case16.2/rip.rep2.sam
hisat2 -x ../example_data/hg38.intg.virg.fa -
U ./output/case16.2/controll.rep1_trimmed.fq.gz -
S ./output/case16.2/controll.rep1.sam
hisat2 -x ../example_data/hg38.intg.virg.fa -
U ./output/case16.2/control2.rep2_trimmed.fq.gz -
S ./output/case16.2/control2.rep2.sam

# step 4
source activate abc
conda config --add channels conda-forge
conda config --add channels bioconda
conda install -y htseq
htseq-count -f sam -s
no ./output/case16.2/rip.rep1.sam ../example_data/gencode.v38.annotation.
gtf > ./output/case16.2/rip.rep1.counts
htseq-count -f sam -s
no ./output/case16.2/rip.rep2.sam ../example_data/gencode.v38.annotation.
gtf > ./output/case16.2/rip.rep2.counts
htseq-count -f sam -s
no ./output/case16.2/controll.rep1.sam ../example_data/gencode.v38.annot
ation.gtf > ./output/case16.2/controll.rep1.counts
htseq-count -f sam -s
no ./output/case16.2/control2.rep2.sam ../example_data/gencode.v38.annot
ation.gtf > ./output/case16.2/control2.rep2.counts

# step 5
source activate abc
conda config --add channels conda-forge
conda config --add channels bioconda
conda install -y bioconductor-deseq2
Rscript -e "library(DESeq2); rip1 <-
read.table('./output/case16.2/rip.rep1.counts', header=TRUE,
row.names=1); rip2 <- read.table('./output/case16.2/rip.rep2.counts',
header=TRUE, row.names=1); control1 <-
read.table('./output/case16.2/controll.rep1.counts', header=TRUE,
```

	row.names=1); control2 <- read.table('./output/case16.2/control2.rep2.counts', header=TRUE, row.names=1); dds <- DESeqDataSetFromMatrix(countData = cbind(rip1, rip2, control1, control2), colData = data.frame(condition = factor(c('rip', 'rip', 'control', 'control'))), design = ~ condition); dds <- DESeq(dds); res <- results(dds); write.table(res, file='./output/case16.2/deseq2_results.txt')"}

Ribo-Seq Analysis   Case 17.1: Identify translated ORFs

**User's Inputs**	**Data and Description**<br>SRR12354645_1.fastq.gz: read 1 of paired-end ribo-seq data, read 1 of control samples replicate 1<br>SRR12354645_2.fastq.gz: read 2 of paired-end ribo-seq data, read 2 of control samples replicate 1<br>SRR12354646_1.fastq.gz: read 1 of paired-end ribo-seq data, read 1 of control samples replicate 2<br>SRR12354646_2.fastq.gz: read 2 of paired-end ribo-seq data, read 2 of control samples replicate 2<br>hg38.fa: the human reference genome<br>gencode.v37.annotation.gtf: gene annotation<br>TruSeq3-PE.fa: trimming adapter<br><br>**Goal**<br>Identify translated ORFs based on ribo-seq data
**Generated Plans by AutoBA**	1. First, we will use the software 'FastQC' to perform quality control checks on raw sequence data coming from high throughput sequencing pipelines.<br>2. Next, we will use the software 'Trim Galore!' to trim the low-quality ends from reads in addition to removing any technical sequences or adapters which may be present.<br>3. After trimming, we will use the software 'HISAT2' to align the trimmed reads to the reference genome '../example_data/hg38.fa'.<br>4. The aligned reads will then be sorted and indexed using the software 'SAMtools'.<br>5. We will use the software 'StringTie' to assemble the aligned reads into transcripts. The gene annotation file '../example_data/gencode.v38.annotation.gtf' will be used for guiding the assembly process.<br>6. Finally, we will use the software 'RiboTaper' to identify translated ORFs based on the assembled transcripts and the ribo-seq data.
**Generated Codes by AutoBA**	**# step 1**<br>source activate abc<br>conda config --add channels conda-forge<br>conda config --add channels bioconda<br>conda install -y fastqc<br>fastqc ../example_data/case16/SRR12354645_1.fastq.gz -<br>o ../output/case17.3.1<br>fastqc ../example_data/case16/SRR12354645_2.fastq.gz -<br>o ../output/case17.3.1<br>fastqc ../example_data/case16/SRR12354646_1.fastq.gz -<br>o ../output/case17.3.1<br>fastqc ../example_data/case16/SRR12354646_2.fastq.gz -<br>o ../output/case17.3.1<br><br>**# step 2**<br>source activate abc<br>conda config --add channels conda-forge<br>conda config --add channels bioconda<br>conda install -y trim-galore

```
trim_galore --paired --adapter ../example_data/TruSeq3-
PE.fa ../example_data/case16/SRR12354645_1.fastq.gz ../example_data/c
ase16/SRR12354645_2.fastq.gz -o ../output/case17.3.1
trim_galore --paired --adapter ../example_data/TruSeq3-
PE.fa ../example_data/case16/SRR12354646_1.fastq.gz ../example_data/c
ase16/SRR12354646_2.fastq.gz -o ../output/case17.3.1

# step 3
source activate abc
conda config --add channels conda-forge
conda config --add channels bioconda
conda install -y hisat2
hisat2-build ../example_data/hg38.fa ../output/case17.3.1/hg38
hisat2 -x ../output/case17.3.1/hg38 -
1 ../output/case17.3.1/SRR12354645_1_val_1.fq.gz -
2 ../output/case17.3.1/SRR12354645_2_val_2.fq.gz -
S ../output/case17.3.1/SRR12354645.sam
hisat2 -x ../output/case17.3.1/hg38 -
1 ../output/case17.3.1/SRR12354646_1_val_1.fq.gz -
2 ../output/case17.3.1/SRR12354646_2_val_2.fq.gz -
S ../output/case17.3.1/SRR12354646.sam

# step 4
source activate abc
conda config --add channels conda-forge
conda config --add channels bioconda
conda install -y samtools
samtools sort ../output/case17.3.1/SRR12354645.sam -
o ../output/case17.3.1/SRR12354645_sorted.bam
samtools index ../output/case17.3.1/SRR12354645_sorted.bam
samtools sort ../output/case17.3.1/SRR12354646.sam -
o ../output/case17.3.1/SRR12354646_sorted.bam
samtools index ../output/case17.3.1/SRR12354646_sorted.bam

# step 5
source activate abc
conda config --add channels conda-forge
conda config --add channels bioconda
conda install -y stringtie
stringtie ../output/case17.3.1/SRR12354645_sorted.bam -
G ../example_data/gencode.v38.annotation.gtf -
o ../output/case17.3.1/SRR12354645_transcripts.gtf
stringtie ../output/case17.3.1/SRR12354646_sorted.bam -
G ../example_data/gencode.v38.annotation.gtf -
o ../output/case17.3.1/SRR12354646_transcripts.gtf

# step 6
source activate abc
conda config --add channels conda-forge
conda config --add channels bioconda
conda install -y ribotaper
```

	RiboTaper -i ../output/case17.3.1/SRR12354645_sorted.bam -<br>a ../output/case17.3.1/SRR12354645_transcripts.gtf -<br>o ../output/case17.3.1/SRR12354645_ribotaper<br>RiboTaper -i ../output/case17.3.1/SRR12354646_sorted.bam -<br>a ../output/case17.3.1/SRR12354646_transcripts.gtf -<br>o ../output/case17.3.1/SRR12354646_ribotaper

scRNA-Seq Analysis Case 18.1: Cluster cell into groups based on raw fastq files

<table>
<tr><td>User’s Inputs</td><td>Data and Description
/pbmc_1k_v3_fastqs.tar: the packed fastq files from 10x single cell sequencing
refdata-gex-GRCh38-2020-A: directory contains transcriptome reference

Goal
cluster cells into different groups based on 10x single cell sequencing data</td></tr>
<tr><td>Generated Plans by AutoBA</td><td>1. Unpack the fastq files from '../example_data/case18/pbmc_1k_v3_fastqs.tar'. This contains the raw sequencing data that we will need to process.
2. Load the transcriptome reference from '../example_data/case18/refdata-gex-GRCh38-2020-A'.
3. Use the software 'Cell Ranger' to perform alignment, filtering, barcode counting, and UMI counting.
4. Import the output from 'Cell Ranger' into 'Seurat', a software for single cell RNA-seq data analysis. This will allow us to perform quality control, normalization, and scaling.
5. Use 'Seurat' to identify highly variable genes. These genes will be used for downstream analysis.
6. Perform principal component analysis (PCA) using 'Seurat'. This will reduce the dimensionality of our data and allow us to identify clusters of cells.
7. Use the 'FindClusters' function in 'Seurat' to cluster cells based on their PCA scores. This will group cells into different clusters based on their gene expression profiles.
8. Visualize the clusters using the 'RunUMAP' function in 'Seurat'. This will provide a 2D representation of the clusters, allowing us to visually inspect the results.
9. Interpret the results and write a report detailing the different cell clusters identified, their gene expression profiles, and potential biological significance.</td></tr>
<tr><td>Generated Codes by AutoBA</td><td># Step 1
source activate abc
conda install -y tar

tar -xvf ../example_data/case18/pbmc_1k_v3_fastqs.tar -
C ../output/case18.1.1

step 2
source activate abc
conda install -y cellranger

cellranger mkref --genome=GRCh38-2020-A --
fasta=../example_data/case18/refdata-gex-GRCh38-2020-
A/fasta/genome.fa --genes=../example_data/case18/refdata-gex-GRCh38-
2020-A/genes/genes.gtf

step 3
source activate abc
conda install -y cellranger</td></tr>
</table>

	cellranger count --id=sample --transcriptome=../example_data/case18/refdata-gex-GRCh38-2020-A --fastqs=../output/case18.1.1/pbmc_1k_v3_fastqs --sample=pbmc_1k_v3<br><br>**# Step 2-12**<br>Rscript -e "library(Seurat); sample <- Read10X(data.dir = '../output/case18.1.1/sample/outs/filtered_feature_bc_matrix'); seurat_obj <- CreateSeuratObject(counts = sample); seurat_obj <- NormalizeData(seurat_obj); seurat_obj <- FindVariableFeatures(seurat_obj); seurat_obj <- ScaleData(seurat_obj); seurat_obj <- RunPCA(seurat_obj, features = VariableFeatures(object = seurat_obj)); seurat_obj <- FindClusters(seurat_obj, resolution = 0.5); seurat_obj <- RunUMAP(seurat_obj, dims = 1:10); Idents(seurat_obj) <- 'seurat_clusters'; cluster1 <- subset(seurat_obj, idents = 1); cluster2 <- subset(seurat_obj, idents = 2); cluster1_genes <- FindAllMarkers(cluster1, only.pos = TRUE, min.pct = 0.25, logfc.threshold = 0.25); cluster2_genes <- FindAllMarkers(cluster2, only.pos = TRUE, min.pct = 0.25, logfc.threshold = 0.25); write.csv(cluster1_genes, file = '../output/case18.1.1/cluster1_genes.csv'); write.csv(cluster2_genes, file = '../output/case18.1.1/cluster2_genes.csv')".

scRNA-Seq Analysis Case 18.2: Clustering and Find the differentially expressed genes

<table>
<tr><td>User's Inputs</td><td>Data and Description
./examples/case2.1/data/filtered_gene_bc_matrices/hg19: path to 10x mtx data

Goal
Use scanpy to find the differentially expressed genes</td></tr>
<tr><td>Generated Plans by AutoBA</td><td>1. Load the 10x mtx data using the Scanpy's read_10x_mtx function. The file path is './examples/case2.1/data/filtered_gene_bc_matrices/hg19'.
2. Preprocess the data using the Scanpy's pp.filter_cells and pp.filter_genes functions to remove cells and genes with too few counts.
3. Normalize the data using the Scanpy's pp.normalize_total function.
4. Logarithmize the data using the Scanpy's pp.log1p function.
5. Identify highly-variable genes using the Scanpy's pp.highly_variable_genes function.
6. Scale the data to unit variance and zero mean using the Scanpy's pp.scale function.
7. Run PCA using the Scanpy's tl.pca function to reduce the dimensionality of the data.
8. Compute the neighborhood graph using the Scanpy's pp.neighbors function.
9. Run UMAP using the Scanpy's tl.umap function for visualization.
10. Cluster the cells using the Scanpy's tl.leiden function.
11. Find marker genes for each cluster using the Scanpy's tl.rank_genes_groups function.
12. Finally, identify the differentially expressed genes using the results from the rank_genes_groups function.</td></tr>
<tr><td>Generated Codes by AutoBA</td><td># Step 1
#!/bin/bash
Activate the conda environment
source activate abc
Install scanpy with conda
conda install -y scanpy
Load the 10x mtx data using the Scanpy's read_10x_mtx function
python -c "import scanpy as sc; adata = sc.read_10x_mtx('./examples/case2.1/data/filtered_gene_bc_matrices/hg19')"

Step 2-12
#!/bin/bash
Activate the conda environment
source activate abc
Identify the differentially expressed genes using the results from the rank_genes_groups function
python -c "import scanpy as sc; adata = sc.read_10x_mtx('./examples/case2.1/data/filtered_gene_bc_matrices/hg19'); sc.pp.filter_cells(adata, min_genes=200); sc.pp.filter_genes(adata, min_cells=3); sc.pp.normalize_total(adata, target_sum=1e4); sc.pp.log1p(adata); sc.pp.highly_variable_genes(adata); sc.pp.scale(adata); sc.tl.pca(adata); sc.pp.neighbors(adata); sc.tl.umap(adata); sc.tl.leiden(adata); sc.tl.rank_genes_groups(adata, 'leiden'); print(adata.uns['rank_genes_groups']['names'])"</td></tr>
</table>

scRNA-Seq Analysis Case 18.3: Identify Marker Genes

<table>
<tr><td>User’s Inputs</td><td>Data and Description
./examples/case2.1/data/filtered_gene_bc_matrices/hg19: path to 10x mtx data

Goal
Use scanpy to identify top5 marker genes.</td></tr>
<tr><td>Generated Plans by AutoBA</td><td>1. Load the 10x mtx data from the provided path '../example_data/case18/mtx/' using the 'read_10x_mtx' function in the 'scanpy' package.", "2. Normalize the data using the 'scanpy.pp.normalize_total' function.", "3. Logarithmize the data using the 'scanpy.pp.log1p' function.", "4. Identify highly variable genes using the 'scanpy.pp.highly_variable_genes' function.", "5. Scale the data using the 'scanpy.pp.scale' function.", "6. Run PCA on the data using the 'scanpy.tl.pca' function.", "7. Compute the neighborhood graph using the 'scanpy.pp.neighbors' function.", "8. Embed the data using the 'scanpy.tl.umap' function.", "9. Cluster the data using the 'scanpy.tl.leiden' function.", "10. Find marker genes for each cluster using the 'scanpy.tl.rank_genes_groups' function.", '11. Extract the top 5 marker genes from the result.</td></tr>
<tr><td>Generated Codes by AutoBA</td><td># Step 1
#!/bin/bash

Activate the conda environment
source activate abc

Install scanpy with conda
conda install -y scanpy

Load the 10x mtx data
python -c "import scanpy as sc; adata = sc.read_10x_mtx('../example_data/case18/mtx/')"

Step 2-11
#!/bin/bash

Activate the conda environment
source activate abc

Extract the top 5 marker genes from the result
python -c "import scanpy as sc; adata = sc.read_10x_mtx('../example_data/case18/mtx/'); sc.pp.normalize_total(adata); sc.pp.log1p(adata); sc.pp.highly_variable_genes(adata); sc.pp.scale(adata); sc.tl.pca(adata); sc.pp.neighbors(adata); sc.tl.umap(adata); sc.tl.leiden(adata); sc.tl.rank_genes_groups(adata, 'leiden'); top5_genes = adata.uns['rank_genes_groups']['names'][:, :5]; print(top5_genes)"</td></tr>
</table>

scRNA-Seq Analysis Case 18.4: Clustering and Visualization

**User's Inputs**	**Data and Description**<br>./examples/case2.1/data/filtered_gene_bc_matrices/hg19: path to 10x mtx data<br><br>**Goal**<br>Use scanpy to perform clustering and visualize the expression level of gene PPBP in the UMAP.
**Generated Plans by AutoBA**	1. Load the 10x mtx data from the provided file path './examples/case2.2/data/filtered_gene_bc_matrices/hg19' using the scanpy.read_10x_mtx function.<br>2. Normalize the data using the scanpy.pp.normalize_total function.<br>3. Logarithmize the data using the scanpy.pp.log1p function.<br>4. Identify highly-variable genes using the scanpy.pp.highly_variable_genes function.<br>5. Scale the data to unit variance and zero mean using the scanpy.pp.scale function.<br>6. Run PCA to reduce dimensionality using the scanpy.tl.pca function.<br>7. Compute the neighborhood graph using the scanpy.pp.neighbors function.<br>8. Embed the neighborhood graph using UMAP via the scanpy.tl.umap function.<br>9. Cluster cells using the Leiden algorithm via the scanpy.tl.leiden function.<br>10. Find marker genes for each cluster using the scanpy.tl.rank_genes_groups function.<br>11. Visualize the expression level of gene PPBP in the UMAP using the scanpy.pl.umap function.
**Generated Codes by AutoBA**	**# Step 1**<br># Activate the conda environment<br>source activate abc<br># Install scanpy<br>conda install -y scanpy<br># Load the 10x mtx data<br>python -c "import scanpy as sc; adata = sc.read_10x_mtx('./examples/case2.2/data/filtered_gene_bc_matrices/hg19')"<br><br>**# Step 2-11**<br>source activate abc<br># Visualize the expression level of gene PPBP in the UMAP<br>python -c "import scanpy as sc; adata = sc.read_10x_mtx('./examples/case2.2/data/filtered_gene_bc_matrices/hg19'); sc.pp.normalize_total(adata); sc.pp.log1p(adata); sc.pp.highly_variable_genes(adata); sc.pp.scale(adata); sc.tl.pca(adata); sc.pp.neighbors(adata); sc.tl.umap(adata); sc.tl.leiden(adata); sc.tl.rank_genes_groups(adata, 'leiden'); sc.pl.umap(adata, color='PPBP')"

Spatial Transcriptomics **Case 19.1: Neighborhood enrichment analysis**

**User's Inputs**	**Data and Description**<br>slice1.h5ad: spatial transcriptomics data for slice 1 in AnnData format<br><br>**Goal**<br>Use squidpy for neighborhood enrichment analysis
**Generated Plans by AutoBA**	1. Load the necessary libraries for the analysis. In this case, we will use 'squidpy' and 'anndata' for the analysis. Note that 'squidpy' is a powerful tool for spatial transcriptomics data analysis.<br>2. Load the spatial transcriptomics data for slice 1 using the 'anndata.read_h5ad' function. The file path is './examples/case4.1/data/slice1.h5ad'.<br>3. Perform initial data exploration to understand the structure and content of the data. This can be done using functions such as 'adata.obs' and 'adata.var' in 'anndata'.<br>4. Use the 'squidpy.gr.spatial_neighbors' function to compute a spatial graph based on the spatial coordinates of the data. This function will add a spatial neighbors graph to the AnnData object.<br>5. Use the 'squidpy.gr.nhood_enrichment' function to compute neighborhood enrichment. This function tests for each cluster, whether its spatial neighbors are randomly distributed.<br>6. Visualize the neighborhood enrichment results using the 'squidpy.pl.nhood_enrichment' function. This function plots the neighborhood enrichment test results.<br>7. Save and export the results for further analysis or presentation.
**Generated Codes by AutoBA**	**# Step 1**<br># Activate the conda environment<br>source activate abc<br># Install the necessary libraries<br>conda install -y squidpy<br>conda install -y anndata<br><br>**# Step 2-3**<br>source activate abc<br># Perform initial data exploration<br>python -c "import anndata; adata = anndata.read_h5ad('./examples/case4.1/data/slice1.h5ad'); print(adata.obs); print(adata.var)"<br><br>**# Step 4-7**<br>source activate abc<br># Compute a spatial graph based on the spatial coordinates of the data<br>python -c "import anndata; import squidpy as sq; adata = anndata.read_h5ad('./examples/case4.1/data/slice1.h5ad'); sq.gr.spatial_neighbors(adata); sq.gr.nhood_enrichment(adata); adata.write('./examples/case4.1/output/results.h5ad')"

Spatial Transcriptomics **Case 19.2: Single-cell mapping**

**User's Inputs**	**Data and Description**<br>slice1.h5ad: spatial transcriptomics data for slice 1 in AnnData format<br>sc.h5ad: single cell data in the same tissue for slice 1 in AnnData format<br><br>**Goal**<br>Use Tangram to map single-cell data to spatial transcriptomics data.
**Generated Plans by AutoBA**	1. Load the spatial transcriptomics data and single cell data using the 'anndata' Python library. The file paths are './experiments/case19.4/data/slice1.h5ad' and './experiments/case19.4/data/sc.h5ad' respectively.<br>2. Preprocess the data if necessary. This may include normalization, filtering, and identification of highly variable genes. This can be done using the 'scanpy' Python library.<br>3. Use the 'tangram' Python library to map the single cell data to the spatial transcriptomics data. This involves creating a mapping object, fitting the model, and then applying the mapping to the spatial data.<br>4. Save the mapped data for further analysis. This can be done using the 'anndata' library's write function.<br>5. Perform downstream analysis on the mapped data. This may include visualization, clustering, and differential expression analysis. This can be done using the 'scanpy' library.
**Generated Codes by AutoBA**	**# Step 1**<br>#!/bin/bash<br><br># Activate the conda environment<br>source activate abc<br><br># Add conda-forge and bioconda to the list of channels<br>conda config --add channels conda-forge<br>conda config --add channels bioconda<br><br># Install anndata library<br>conda install -y anndata<br><br># Load the spatial transcriptomics data<br>python -c "import anndata; adata1 = anndata.read_h5ad('./experiments/case19.4/data/slice1.h5ad')"<br><br># Load the single cell data<br>python -c "import anndata; adata2 = anndata.read_h5ad('./experiments/case19.4/data/sc.h5ad')"<br><br>**# Step 2**<br>#!/bin/bash<br><br># Activate the conda environment<br>source activate abc<br><br># Add conda-forge and bioconda to the list of channels<br>conda config --add channels conda-forge<br>conda config --add channels bioconda

	# Install scanpy library<br>conda install -y scanpy<br><br># Preprocess the spatial transcriptomics data<br>python -c "import scanpy as sc; sc.pp.normalize_total(adata1, target_sum=1e4); sc.pp.log1p(adata1); sc.pp.highly_variable_genes(adata1, min_mean=0.0125, max_mean=3, min_disp=0.5); adata1.raw = adata1"<br><br># Preprocess the single cell data<br>python -c "import scanpy as sc; sc.pp.normalize_total(adata2, target_sum=1e4); sc.pp.log1p(adata2); sc.pp.highly_variable_genes(adata2, min_mean=0.0125, max_mean=3, min_disp=0.5); adata2.raw = adata2"<br><br>**# Step 3**<br>#!/bin/bash<br><br># Activate the conda environment<br>source activate abc<br><br># Add conda-forge and bioconda to the list of channels<br>conda config --add channels conda-forge<br>conda config --add channels bioconda<br><br># Install tangram library<br>conda install -y tangram<br><br># Map the single cell data to the spatial transcriptomics data<br>python -c "import tangram as tg; import anndata; adata1 = anndata.read_h5ad('./experiments/case19.4/data/slice1.h5ad'); adata2 = anndata.read_h5ad('./experiments/case19.4/data/sc.h5ad'); mapper = tg.MapSpace(adata2, adata1, device='cpu'); mapper.train(); adata_map = mapper.map_cells()"<br><br>**# Step 4**<br>#!/bin/bash<br><br># Activate the conda environment<br>source activate abc<br><br># Add conda-forge and bioconda to the list of channels<br>conda config --add channels conda-forge<br>conda config --add channels bioconda<br><br># Save the mapped data<br>python -c "import anndata; adata_map.write('./experiments/case19.4/output/mapped_data.h5ad')"<br><br>**# Step 5**<br>#!/bin/bash<br><br># Activate the conda environment

	source activate abc<br><br># Add conda-forge and bioconda to the list of channels<br>conda config --add channels conda-forge<br>conda config --add channels bioconda<br><br># Perform downstream analysis on the mapped data<br>python -c "import scanpy as sc; import anndata; adata_map = anndata.read_h5ad('./experiments/case19.4/output/mapped_data.h5ad'); sc.pp.neighbors(adata_map); sc.tl.umap(adata_map); sc.tl.leiden(adata_map); sc.pl.umap(adata_map, color='leiden')"

Proteomics Case 20.1: protein expression quantification

<table>
<tr><td>User's Inputs</td><td>Data and Description
01CPTAC_COprospective_W_PNNL_20170123_B1S1_f01.mzML.gz: protein mass spectrometry data in mzML format, tumor samples fraction 1
01CPTAC_COprospective_W_PNNL_20170123_B1S1_f02.mzML.gz: protein mass spectrometry data in mzML format, tumor samples fraction 2
01CPTAC_COprospective_W_PNNL_20170123_B1S1_f03.mzML.gz: protein mass spectrometry data in mzML format, tumor samples fraction 3
98CPTAC_COprospective_W_VU_20160804_11CO047_f01.mzML.gz: protein mass spectrometry data in mzML format, normal samples fraction 1
98CPTAC_COprospective_W_VU_20160804_11CO047_f02.mzML.gz: protein mass spectrometry data in mzML format, normal samples fraction 2
98CPTAC_COprospective_W_VU_20160804_11CO047_f03.mzML.gz: protein mass spectrometry data in mzML format, normal samples fraction 3
UP000005640_9606.fasta: reference protein sequence from UniProt

Goal
identify proteins in tumor and normal samples based on mass spectrometry data</td></tr>
<tr><td>Generated Plans by AutoBA</td><td>1. Load the provided mass spectrometry data files using a software like 'ProteoWizard'.
2. Preprocess the data to remove noise and normalize the data using a software like 'OpenMS'.
3. Perform peak detection on the preprocessed data using a software like 'OpenMS'.
4. Perform protein identification by matching the detected peaks with the provided reference protein sequence file using a software like 'OpenMS'.
5. Repeat steps 1-4 for each of the provided mass spectrometry data files.
6. Compare the identified proteins in tumor and normal samples to identify differences.
7. Document the identified proteins and differences for further analysis.</td></tr>
<tr><td>Generated Codes by AutoBA</td><td># step 1 (output not used in the downstream analysis)
source activate abc

Install ProteoWizard
conda install -y proteowizard

Load the mass spectrometry data files
msconvert ./examples/case20.1/01CPTAC_COprospective_W_PNNL_20170123_B1S1_f01.mzML.gz
msconvert ./examples/case20.1/01CPTAC_COprospective_W_PNNL_20170123_B1S1_f02.mzML.gz
msconvert ./examples/case20.1/01CPTAC_COprospective_W_PNNL_20170123_B1S1_f03.mzML.gz
msconvert ./examples/case20.1/98CPTAC_COprospective_W_VU_20160804_11CO047_f01.mzML.gz
msconvert ./examples/case20.1/98CPTAC_COprospective_W_VU_20160804_11CO047_f02.mzML.gz
msconvert ./examples/case20.1/98CPTAC_COprospective_W_VU_20160804_11CO047_f03.mzML.gz

step 2</td></tr>
</table>

	# Activate the environment<br>source activate abc<br><br># Install OpenMS<br>conda install -y openms<br><br># Preprocess the data to remove noise and normalize the data<br>FileFilter -<br>in ./examples/case20.1/01CPTAC_COprospective_W_PNNL_20170123_B1S1_f01.mzML.gz -<br>out ../output/case20.1/01CPTAC_COprospective_W_PNNL_20170123_B1S1_f01_preprocessed.mzML<br>FileFilter -<br>in ./examples/case20.1/01CPTAC_COprospective_W_PNNL_20170123_B1S1_f02.mzML.gz -<br>out ../output/case20.1/01CPTAC_COprospective_W_PNNL_20170123_B1S1_f02_preprocessed.mzML<br>FileFilter -<br>in ./examples/case20.1/01CPTAC_COprospective_W_PNNL_20170123_B1S1_f03.mzML.gz -<br>out ../output/case20.1/01CPTAC_COprospective_W_PNNL_20170123_B1S1_f03_preprocessed.mzML<br>FileFilter -<br>in ./examples/case20.1/98CPTAC_COprospective_W_VU_20160804_11CO047_f01.mzML.gz -<br>out ../output/case20.1/98CPTAC_COprospective_W_VU_20160804_11CO047_f01_preprocessed.mzML<br>FileFilter -<br>in ./examples/case20.1/98CPTAC_COprospective_W_VU_20160804_11CO047_f02.mzML.gz -<br>out ../output/case20.1/98CPTAC_COprospective_W_VU_20160804_11CO047_f02_preprocessed.mzML<br>FileFilter -<br>in ./examples/case20.1/98CPTAC_COprospective_W_VU_20160804_11CO047_f03.mzML.gz -<br>out ../output/case20.1/98CPTAC_COprospective_W_VU_20160804_11CO047_f03_preprocessed.mzML<br><br>**# step 3**<br>source activate abc<br><br># Install OpenMS<br>conda install -y openms<br><br># Perform peak detection on the preprocessed data<br>PeakPickerHiRes -<br>in ../output/case20.1/01CPTAC_COprospective_W_PNNL_20170123_B1S1_f01_preprocessed.mzML -<br>out ../output/case20.1/01CPTAC_COprospective_W_PNNL_20170123_B1S1_f01_peaks.mzML<br>PeakPickerHiRes -<br>in ../output/case20.1/01CPTAC_COprospective_W_PNNL_20170123_B1S1_f02_preprocessed.mzML -

	out ../output/case20.1/01CPTAC_COprospective_W_PNNL_20170123_B1S1_f02_peaks.mzML<br>PeakPickerHiRes -<br>in ../output/case20.1/01CPTAC_COprospective_W_PNNL_20170123_B1S1_f03_preprocessed.mzML -<br>out ../output/case20.1/01CPTAC_COprospective_W_PNNL_20170123_B1S1_f03_peaks.mzML<br>PeakPickerHiRes -<br>in ../output/case20.1/98CPTAC_COprospective_W_VU_20160804_11CO047_f01_preprocessed.mzML -<br>out ../output/case20.1/98CPTAC_COprospective_W_VU_20160804_11CO047_f01_peaks.mzML<br>PeakPickerHiRes -<br>in ../output/case20.1/98CPTAC_COprospective_W_VU_20160804_11CO047_f02_preprocessed.mzML -<br>out ../output/case20.1/98CPTAC_COprospective_W_VU_20160804_11CO047_f02_peaks.mzML<br>PeakPickerHiRes -<br>in ../output/case20.1/98CPTAC_COprospective_W_VU_20160804_11CO047_f03_preprocessed.mzML -<br>out ../output/case20.1/98CPTAC_COprospective_W_VU_20160804_11CO047_f03_peaks.mzML<br><br>**# step 4**<br>source activate abc<br><br># Install OpenMS<br>conda install -y openms<br><br># Perform protein identification by matching the detected peaks with the provided reference protein sequence file<br>OMSSAAdapter -<br>in ../output/case20.1/01CPTAC_COprospective_W_PNNL_20170123_B1S1_f01_peaks.mzML -db ./examples/UP000005640_9606.fasta -<br>out ../output/case20.1/01CPTAC_COprospective_W_PNNL_20170123_B1S1_f01_identified.mzML<br>OMSSAAdapter -<br>in ../output/case20.1/01CPTAC_COprospective_W_PNNL_20170123_B1S1_f02_peaks.mzML -db ./examples/UP000005640_9606.fasta -<br>out ../output/case20.1/01CPTAC_COprospective_W_PNNL_20170123_B1S1_f02_identified.mzML<br>OMSSAAdapter -<br>in ../output/case20.1/01CPTAC_COprospective_W_PNNL_20170123_B1S1_f03_peaks.mzML -db ./examples/UP000005640_9606.fasta -<br>out ../output/case20.1/01CPTAC_COprospective_W_PNNL_20170123_B1S1_f03_identified.mzML<br>OMSSAAdapter -<br>in ../output/case20.1/98CPTAC_COprospective_W_VU_20160804_11CO047_f01_peaks.mzML -db ./examples/UP000005640_9606.fasta -<br>out ../output/case20.1/98CPTAC_COprospective_W_VU_20160804_11CO047_f01_identified.mzML<br>OMSSAAdapter -<br>in ../output/case20.1/98CPTAC_COprospective_W_VU_20160804_11CO0

	47_f02_peaks.mzML -db ./examples/UP000005640_9606.fasta -<br>out ../output/case20.1/98CPTAC_COprospective_W_VU_20160804_11CO<br>047_f02_identified.mzML<br>OMSSAAdapter -<br>in ../output/case20.1/98CPTAC_COprospective_W_VU_20160804_11CO0<br>47_f03_peaks.mzML -db ./examples/UP000005640_9606.fasta -<br>out ../output/case20.1/98CPTAC_COprospective_W_VU_20160804_11CO<br>047_f03_identified.mzML<br><br>**# step 5**<br>echo 'All steps have been performed for each of the provided mass spectrometry data files.'<br><br>**# step 6**<br>source activate abc<br><br># Install OpenMS<br>conda install -y openms<br><br># Compare the identified proteins in tumor and normal samples to identify differences<br>ProteinQuantifier -<br>in ../output/case20.1/01CPTAC_COprospective_W_PNNL_20170123_B1S<br>1_f01_identified.mzML -<br>out ../output/case20.1/01CPTAC_COprospective_W_PNNL_20170123_B1<br>S1_f01_quantified.csv<br>ProteinQuantifier -<br>in ../output/case20.1/01CPTAC_COprospective_W_PNNL_20170123_B1S<br>1_f02_identified.mzML -<br>out ../output/case20.1/01CPTAC_COprospective_W_PNNL_20170123_B1<br>S1_f02_quantified.csv<br>ProteinQuantifier -<br>in ../output/case20.1/01CPTAC_COprospective_W_PNNL_20170123_B1S<br>1_f03_identified.mzML -<br>out ../output/case20.1/01CPTAC_COprospective_W_PNNL_20170123_B1<br>S1_f03_quantified.csv<br>ProteinQuantifier -<br>in ../output/case20.1/98CPTAC_COprospective_W_VU_20160804_11CO0<br>47_f01_identified.mzML -<br>out ../output/case20.1/98CPTAC_COprospective_W_VU_20160804_11CO<br>047_f01_quantified.csv<br>ProteinQuantifier -<br>in ../output/case20.1/98CPTAC_COprospective_W_VU_20160804_11CO0<br>47_f02_identified.mzML -<br>out ../output/case20.1/98CPTAC_COprospective_W_VU_20160804_11CO<br>047_f02_quantified.csv<br>ProteinQuantifier -<br>in ../output/case20.1/98CPTAC_COprospective_W_VU_20160804_11CO0<br>47_f03_identified.mzML -<br>out ../output/case20.1/98CPTAC_COprospective_W_VU_20160804_11CO<br>047_f03_quantified.csv<br>**# step 7**<br>source activate abc

	# Install OpenMS<br>conda install -y openms<br><br># Document the identified proteins and differences for further analysis<br>TextExporter -<br>in ../output/case20.1/01CPTAC_COprospective_W_PNNL_20170123_B1S<br>1_f01_quantified.csv -<br>out ../output/case20.1/01CPTAC_COprospective_W_PNNL_20170123_B1<br>S1_f01_quantified.txt<br>TextExporter -<br>in ../output/case20.1/01CPTAC_COprospective_W_PNNL_20170123_B1S<br>1_f02_quantified.csv -<br>out ../output/case20.1/01CPTAC_COprospective_W_PNNL_20170123_B1<br>S1_f02_quantified.txt<br>TextExporter -<br>in ../output/case20.1/01CPTAC_COprospective_W_PNNL_20170123_B1S<br>1_f03_quantified.csv -<br>out ../output/case20.1/01CPTAC_COprospective_W_PNNL_20170123_B1<br>S1_f03_quantified.txt<br>TextExporter -<br>in ../output/case20.1/98CPTAC_COprospective_W_VU_20160804_11CO0<br>47_f01_quantified.csv -<br>out ../output/case20.1/98CPTAC_COprospective_W_VU_20160804_11CO<br>047_f01_quantified.txt<br>TextExporter -<br>in ../output/case20.1/98CPTAC_COprospective_W_VU_20160804_11CO0<br>47_f02_quantified.csv -<br>out ../output/case20.1/98CPTAC_COprospective_W_VU_20160804_11CO<br>047_f02_quantified.txt<br>TextExporter -<br>in ../output/case20.1/98CPTAC_COprospective_W_VU_20160804_11CO0<br>47_f03_quantified.csv -<br>out ../output/case20.1/98CPTAC_COprospective_W_VU_20160804_11CO<br>047_f03_quantified.txt

Metabolomics Case 21.1: metabolites concentration

**User's Inputs**	**Data and Description**<br>B1S1_f01.mzML.gz: LC-MS mass spectrometry data of metabolites in mzML format, tumor samples fraction 1<br>B1S1_f02.mzML.gz: LC-MS mass spectrometry data of metabolites in mzML format, tumor samples fraction 2<br>B1S1_f03.mzML.gz: LC-MS mass spectrometry data of metabolites in mzML format, tumor samples fraction 3<br>11CO047_f01.mzML.gz: LC-MS mass spectrometry data of metabolites in mzML format, normal samples fraction 1<br>11CO047_f02.mzML.gz: LC-MS mass spectrometry data of metabolites in mzML format, normal samples fraction 2<br>11CO047_f03.mzML.gz: LC-MS mass spectrometry data of metabolites in mzML format, normal samples fraction 3<br><br>**Goal**<br>calculate metabolites concentration in tumor and normal samples based on LC-MS mass spectrometry data
**Generated Plans by AutoBA**	1. Load the provided LC-MS mass spectrometry data files using a Python script with the help of the 'pymzml' library. This library is specifically designed for the parsing of mzML files, a common file format for mass spectrometry data.<br>2. For each file, extract the metabolites and their corresponding intensities. This can be done using the 'get_spectrum()' function in the 'pymzml' library.<br>3. Normalize the intensity values to obtain the relative concentration of each metabolite. This can be done using the 'normalize()' function in the 'numpy' library.<br>4. Group the data by sample type (tumor or normal) and calculate the average concentration of each metabolite in each group. This can be done using the 'groupby()' and 'mean()' functions in the 'pandas' library.<br>5. Compare the average concentrations of metabolites between tumor and normal samples. This can be done using the 'ttest_ind()' function in the 'scipy' library to perform a t-test, which will provide a p-value indicating whether the difference in concentrations is statistically significant.<br>6. Output the results in a tabular format, with rows representing metabolites and columns representing sample types (tumor and normal). Each cell in the table will contain the average concentration of a metabolite in a specific sample type. This can be done using the 'to_csv()' function in the 'pandas' library to export the table to a CSV file.
**Generated Codes by AutoBA**	# step 1-4<br>source activate abc<br>conda install -y pymzml<br><br>conda install -y numpy<br><br># Install pandas library<br>conda install -y pandas<br><br># Python script to group the data and calculate the average concentration<br>python << EOF<br>import pymzml<br>import numpy as np

```
import pandas as pd

# Load the LC-MS mass spectrometry data files
file1 = pymzml.run.Reader('./examples/case21.1/B1S1_f01.mzML.gz')
file2 = pymzml.run.Reader('./examples/case21.1/B1S1_f02.mzML.gz')
file3 = pymzml.run.Reader('./examples/case21.1/B1S1_f03.mzML.gz')
file4 = pymzml.run.Reader('./examples/case21.1/11CO047_f01.mzML.gz')
file5 = pymzml.run.Reader('./examples/case21.1/11CO047_f02.mzML.gz')
file6 = pymzml.run.Reader('./examples/case21.1/11CO047_f03.mzML.gz')

# Extract the metabolites and their corresponding intensities
metabolites1 = [spectrum['m/z array'] for spectrum in file1 if 'm/z array' in spectrum]
intensities1 = [spectrum['intensity array'] for spectrum in file1 if 'intensity array' in spectrum]

metabolites2 = [spectrum['m/z array'] for spectrum in file2 if 'm/z array' in spectrum]
intensities2 = [spectrum['intensity array'] for spectrum in file2 if 'intensity array' in spectrum]

metabolites3 = [spectrum['m/z array'] for spectrum in file3 if 'm/z array' in spectrum]
intensities3 = [spectrum['intensity array'] for spectrum in file3 if 'intensity array' in spectrum]

metabolites4 = [spectrum['m/z array'] for spectrum in file4 if 'm/z array' in spectrum]
intensities4 = [spectrum['intensity array'] for spectrum in file4 if 'intensity array' in spectrum]

metabolites5 = [spectrum['m/z array'] for spectrum in file5 if 'm/z array' in spectrum]
intensities5 = [spectrum['intensity array'] for spectrum in file5 if 'intensity array' in spectrum]

metabolites6 = [spectrum['m/z array'] for spectrum in file6 if 'm/z array' in spectrum]
intensities6 = [spectrum['intensity array'] for spectrum in file6 if 'intensity array' in spectrum]

# Normalize the intensity values
normalized_intensities1 = np.linalg.norm(intensities1)
normalized_intensities2 = np.linalg.norm(intensities2)
normalized_intensities3 = np.linalg.norm(intensities3)
normalized_intensities4 = np.linalg.norm(intensities4)
normalized_intensities5 = np.linalg.norm(intensities5)
normalized_intensities6 = np.linalg.norm(intensities6)

# Create a dataframe for each file
df1 = pd.DataFrame({'Metabolite': metabolites1, 'Intensity': normalized_intensities1, 'Sample': 'Tumor'})
```

	df2 = pd.DataFrame({'Metabolite': metabolites2, 'Intensity': normalized_intensities2, 'Sample': 'Tumor'})<br>df3 = pd.DataFrame({'Metabolite': metabolites3, 'Intensity': normalized_intensities3, 'Sample': 'Tumor'})<br>df4 = pd.DataFrame({'Metabolite': metabolites4, 'Intensity': normalized_intensities4, 'Sample': 'Normal'})<br>df5 = pd.DataFrame({'Metabolite': metabolites5, 'Intensity': normalized_intensities5, 'Sample': 'Normal'})<br>df6 = pd.DataFrame({'Metabolite': metabolites6, 'Intensity': normalized_intensities6, 'Sample': 'Normal'})<br><br># Concatenate the dataframes<br>df = pd.concat([df1, df2, df3, df4, df5, df6])<br><br># Group the data by sample type and calculate the average concentration<br>average_concentration = df.groupby(['Metabolite', 'Sample'])['Intensity'].mean()<br><br># Save the result to a file<br>average_concentration.to_csv('../output/case21.1/average_concentration.csv')<br><br>**# step 5**<br>source activate abc<br><br># Install scipy library<br>conda install -y scipy<br><br># Python script to perform a t-test<br>python << EOF<br>import pandas as pd<br>from scipy.stats import ttest_ind<br><br># Load the average concentration data<br>average_concentration = pd.read_csv('../output/case21.1/average_concentration.csv')<br><br># Split the data into tumor and normal samples<br>tumor = average_concentration[average_concentration['Sample'] == 'Tumor']['Intensity']<br>normal = average_concentration[average_concentration['Sample'] == 'Normal']['Intensity']<br><br># Perform a t-test<br>t_stat, p_value = ttest_ind(tumor, normal)<br><br># Save the result to a file<br>with open('../output/case21.1/t_test_result.txt', 'w') as f:<br>f.write('t-statistic: ' + str(t_stat) + '\np-value: ' + str(p_value))<br>EOF<br><br>**# step 6**<br>source activate abc

	# Python script to output the results in a tabular format<br>python << EOF<br>import pandas as pd<br><br># Load the average concentration data<br>average_concentration =<br>pd.read_csv('../output/case21.1/average_concentration.csv')<br><br># Pivot the table to have rows representing metabolites and columns representing sample types<br>pivot_table = average_concentration.pivot(index='Metabolite', columns='Sample', values='Intensity')<br><br># Export the table to a CSV file<br>pivot_table.to_csv('../output/case21.1/pivot_table.csv')<br>EOF

6. Results of AutoBA (local version)

WGS data analysis   Case 1.1: Genome assembly

**User's Inputs**	**Data and Description**<br>SRR1234567_R1.fastq.gz: Paired-end Illumina WGS reads, forward<br>SRR1234567_R2.fastq.gz: Paired-end Illumina WGS reads, reverse<br>TruSeq3-PE.fa: Adapter sequences for trimming<br>Reference.fasta: Reference genome for the organism under study in FASTA format<br><br>**Goal**<br>To perform genome assembly using paired-end WGS data
**Generated Plans by AutoBA**	"step 1: Trim the adapters from the paired-end WGS reads using the TruSeq3-PE.fa adapter sequences.",<br>"step 2: Merge the paired-end reads into a single file using a software such as FLASH.",<br>"step 3: Perform quality control and trimming of the merged reads using a software such as Trimmomatic.",<br>"step 4: Assemble the high-quality reads using a software such as SPAdes or Canu.",<br>"step 5: Evaluate the assembled contigs using a software such as Quast or Abyss.",<br>"step 6: Annotate the assembled contigs using a software such as Prokka or RAST.",<br>"step 7: Use the annotated contigs to identify potential functional genes and pathways using a software such as InterProScan or Gene Ontology.",<br>"step 8: Use the identified functional genes and pathways to infer the metabolic capabilities of the organism under study.",<br>"step 9: Use the assembled contigs to perform a phylogenetic analysis to identify the evolutionary relationships between the organism under study and other related species.",<br>"step 10: Use the assembled contigs to perform a genomic context analysis to identify potential regulatory elements and their target genes.",<br>"step 11: Use the identified regulatory elements and target genes to infer the gene regulation network of the organism under study."
**Generated Codes by AutoBA**	conda install -y trimmomatic<br>trimmomatic PE -threads<br>4 ./experiments/case1.1/data/SRR1234567_R1.fastq.gz ./experiments/case1.1/data/SRR1234567_R2.fastq.gz ./experiments/case1.1/data/TruSeq3-PE.fa ./experiments/case1.1/output/SRR1234567_R1_paired.fastq.gz ./experiments/case1.1/output/SRR1234567_R1_unpaired.fastq.gz ./experiments/case1.1/output/SRR1234567_R2_paired.fastq.gz ./experiments/case1.1/output/SRR1234567_R2_unpaired.fastq.gz<br><br>conda install -y spades<br>spades --only-assembler -<br>1 ./experiments/case1.1/output/SRR1234567_R1_paired.fastq.gz -<br>2 ./experiments/case1.1/output/SRR1234567_R2_paired.fastq.gz -<br>o ./experiments/case1.1/output/SRR1234567_assembled.fasta<br><br>conda install -y quast

	quast ./experiments/case1.1/output/SRR1234567_assembled.fasta<br><br>conda install -y prokka<br>prokka ./experiments/case1.1/output/SRR1234567_assembled.fasta -<br>o ./experiments/case1.1/output/SRR1234567_annotated.fasta<br><br>conda install -y interproscan<br>interproscan -i ./experiments/case1.1/output/SRR1234567_annotated.fasta<br>-o ./experiments/case1.1/output/SRR1234567_interproscan.tsv<br><br>conda install -y prokka<br>prokka ./experiments/case1.1/output/SRR1234567_assembled.fasta -<br>o ./experiments/case1.1/output/SRR1234567_annotated.fasta<br><br>conda install -y raxml<br>raxml -s ./experiments/case1.1/output/SRR1234567_assembled.fasta -m<br>GTRGAMMA -n SRR1234567 -p 12345 -<br>o ./experiments/case1.1/output/SRR1234567_raxml.tre<br><br>conda install -y genomiccontext<br>genomiccontext -<br>i ./experiments/case1.1/output/SRR1234567_assembled.fasta -<br>o ./experiments/case1.1/output/SRR1234567_genomiccontext.tsv<br><br>conda install -y genomiccontext<br>genomiccontext -<br>i ./experiments/case1.1/output/SRR1234567_assembled.fasta -<br>o ./experiments/case1.1/output/SRR1234567_genomiccontext.tsv

WGS/WES data analysis **Case 2.1 and Case 2.2 : Somatic SNV+indel calling and annotation**

**User's Inputs**	**Data and Description**<br>SRR23015874_R1.fastq.gz: paired end read1 from human whole genome sequencing, normal samples replicate 1<br>SRR23015874_R2.fastq.gz: paired end read2 from human whole genome sequencing, normal samples replicate 1<br>SRR23015876_R1.fastq.gz: paired end read1 from human whole genome sequencing, tumor samples replicate 1<br>SRR23015876_R2.fastq.gz: paired end read2 from human whole genome sequencing, tumor samples replicate 1<br>SRR23015877_R1.fastq.gz: paired end read1 from human whole genome sequencing, tumor samples replicate 2<br>SRR23015877_R2.fastq.gz: paired end read2 from human whole genome sequencing, tumor samples replicate 2<br>TruSeq3-PE.fa: trimming adapter<br>hg38.fa: human hg38 reference genome fasta<br><br>**Goal**<br>call somatic mutations in tumor samples over the matched normal samples and annotate the identified mutations
**Generated Plans by AutoBA**	"Step 1: Quality Control and Trimming of FastQC Files", "Use FastQC to evaluate the quality of the input FastQ files and remove any reads that do not meet the specified quality standards.", "Step 2: Adapter Removal", "Use Trimmomatic or Cutadapt to remove adapter sequences from the FastQ files.", "Step 3: Read Mapping", "Use BWA or Bowtie2 to map the trimmed reads to the human hg38 reference genome.", "Step 4: Variant Calling", "Use MuTect or Strelka to identify somatic mutations in the tumor samples over the matched normal samples.", "Step 5: Annotation", "Use Annovar or SnpEff to annotate the identified mutations with information such as functional impact, gene expression, and population frequency."
**Generated Codes by AutoBA**	conda activate abc<br>fastqc -<br>o ./experiments/case2.2/output ./experiments/case2.2/data/SRR23015874_R1.fastq.gz ./experiments/case2.2/data/SRR23015874_R2.fastq.gz ./experiments/case2.2/data/SRR23015876_R1.fastq.gz ./experiments/case2.2/data/SRR23015876_R2.fastq.gz ./experiments/case2.2/data/SRR23015877_R1.fastq.gz ./experiments/case2.2/data/SRR23015877_R2.fastq.gz<br><br>conda activate abc<br>fastqc -<br>o ./experiments/case2.2/output ./experiments/case2.2/data/SRR23015874_R1.fastq.gz ./experiments/case2.2/data/SRR23015874_R2.fastq.gz ./experiments/case2.2/data/SRR23015876_R1.fastq.gz ./experiments/case2.2/data/SRR23015876_R2.fastq.gz ./experiments/case2.2/data/SRR23015877_R1.fastq.gz ./experiments/case2.2/data/SRR23015877_R2.fastq.gz<br><br>conda activate abc<br>trimmomatic PE -threads 4 -<br>phred33 ./experiments/case2.2/data/SRR23015874_R1.fastq.gz ./experiments/case2.2/data/SRR23015874_R2.fastq.gz ./experiments/case2.2/data/SRR23015876_R1.fastq.gz ./experiments/case2.2/data/SRR23015876_R2.fastq.gz ./experiments/case2.2/data/SRR23015877_R1.fastq.gz ./experiment

	s/case2.2/data/SRR23015877_R2.fastq.gz ./experiments/case2.2/data/TruSeq3-PE.fa<br><br>conda activate abc<br>trimmomatic PE -threads 4 -phred33 ./experiments/case2.2/data/SRR23015874_R1.fastq.gz ./experiments/case2.2/data/SRR23015874_R2.fastq.gz ./experiments/case2.2/data/SRR23015876_R1.fastq.gz ./experiments/case2.2/data/SRR23015876_R2.fastq.gz ./experiments/case2.2/data/SRR23015877_R1.fastq.gz ./experiments/case2.2/data/SRR23015877_R2.fastq.gz ./experiments/case2.2/data/TruSeq3-PE.fa<br><br>bash code to finish the current task<br><br>conda activate abc<br>bwa mem -t 4 -R '@RG\tID:my-read-group\tSM:my-sample\tPL:illumina' ./experiments/case2.2/data/hg38.fa ./experiments/case2.2/data/SRR23015874_R1.fastq.gz ./experiments/case2.2/data/SRR23015874_R2.fastq.gz ./experiments/case2.2/data/SRR23015876_R1.fastq.gz ./experiments/case2.2/data/SRR23015876_R2.fastq.gz ./experiments/case2.2/data/SRR23015877_R1.fastq.gz ./experiments/case2.2/data/SRR23015877_R2.fastq.gz > ./experiments/case2.2/output/my-output.sam<br><br>conda activate abc<br>mutect -R ./experiments/case2.2/data/hg38.fa -I ./experiments/case2.2/data/SRR23015874_R1.fastq.gz ./experiments/case2.2/data/SRR23015874_R2.fastq.gz ./experiments/case2.2/data/SRR23015876_R1.fastq.gz ./experiments/case2.2/data/SRR23015876_R2.fastq.gz ./experiments/case2.2/data/SRR23015877_R1.fastq.gz ./experiments/case2.2/data/SRR23015877_R2.fastq.gz -o ./experiments/case2.2/output/my-output.vcf<br><br>conda activate abc<br>mutect -R ./experiments/case2.2/data/hg38.fa -I ./experiments/case2.2/data/SRR23015874_R1.fastq.gz ./experiments/case2.2/data/SRR23015874_R2.fastq.gz ./experiments/case2.2/data/SRR23015876_R1.fastq.gz ./experiments/case2.2/data/SRR23015876_R2.fastq.gz ./experiments/case2.2/data/SRR23015877_R1.fastq.gz ./experiments/case2.2/data/SRR23015877_R2.fastq.gz -o ./experiments/case2.2/output/my-output.vcf.<br><br>conda activate abc<br>annovar -out ./experiments/case2.2/output/my-output.vcf -buildver hg38 ./experiments/case2.2/output/my-output.vcf

WGS/WES data analysis **Case 2.3: Structure variation identification with normal**

**User's Inputs**	**Data and Description**<br>SRR23015874_R1.fastq.gz: paired end read1 from human whole genome sequencing, the matched normal samples replicate 1<br>SRR23015874_R2.fastq.gz: paired end read2 from human whole genome sequencing, the matched normal samples replicate 1<br>SRR23015876_R1.fastq.gz: paired end read1 from human whole genome sequencing, tumor samples replicate 1<br>SRR23015876_R2.fastq.gz: paired end read2 from human whole genome sequencing, tumor samples replicate 1<br>SRR23015877_R1.fastq.gz: paired end read1 from human whole genome sequencing, tumor samples replicate 2<br>SRR23015877_R2.fastq.gz: paired end read2 from human whole genome sequencing, tumor samples replicate 2<br>TruSeq3-PE.fa: trimming adapter<br>hg38.fa: human hg38 reference genome fasta<br><br>**Goal**<br>To identify structural variations, which can include large insertions, deletions, inversions, duplications, and translocations, by comparing the sequencing data from the tumor sample over the matched normal sample
**Generated Plans by AutoBA**	"Step 1: Trimming the adapters from the sequencing reads using TruSeq3-PE.fa", "Step 2: Mapping the trimmed reads to the human hg38 reference genome using STAR", "Step 3: Identifying structural variations using GATK's HaplotypeCaller", "Step 4: Filtering and sorting the structural variations using GATK's VariantFiltration and SortVariants", "Step 5: Visualizing the structural variations using a genome browser such as IGV"
**Generated Codes by AutoBA**	conda activate abc<br>trimmomatic PE -threads 4 -phred33 ./experiments/case2.2/data/SRR23015874_R1.fastq.gz ./experiments/case2.2/data/SRR23015874_R2.fastq.gz ./experiments/case2.2/data/SRR23015876_R1.fastq.gz ./experiments/case2.2/data/SRR23015876_R2.fastq.gz ./experiments/case2.2/data/SRR23015877_R1.fastq.gz ./experiments/case2.2/data/SRR23015877_R2.fastq.gz ./experiments/case2.2/data/TruSeq3-PE.fa ./experiments/case2.3/output/trimmed_reads<br><br>conda activate abc<br>STAR --runThreadN 4 --genomeDir ./experiments/case2.2/data/hg38.fa --readFilesIn ./experiments/case2.3/output/trimmed_reads/SRR23015874_R1.fastq.gz ./experiments/case2.3/output/trimmed_reads/SRR23015874_R2.fastq.gz ./experiments/case2.3/output/trimmed_reads/SRR23015876_R1.fastq.gz ./experiments/case2.3/output/trimmed_reads/SRR23015876_R2.fastq.gz ./experiments/case2.3/output/trimmed_reads/SRR23015877_R1.fastq.gz ./experiments/case2.3/output/trimmed_reads/SRR23015877_R2.fastq.gz --outFileNamePrefix ./experiments/case2.3/output/mapped_reads --outSAMtype BAM SortedByCoordinate --outSAMunmapped Within --outSAMattributes NH HI NM MD AS --outFilterMismatchNmax 999 --outFilterMatchNmin 10 --outFilterMultimapNmax 1 --outReadsUnmapped Fastx --outSAMstrandField intronMotif --limitBAMsortRAM 40000000000 --genomeLoad NoSharedMemory<br><br>conda activate abc

<table>
<tr>
<td></td>
<td>gatk HaplotypeCaller -R ./experiments/case2.2/data/hg38.fa -
I ./experiments/case2.3/output/mapped_reads/Aligned.sortedByCoord.out.b
am -O ./experiments/case2.3/output/structural_variations.vcf --emit-ref-
confidence GVCF --dbsnp ./experiments/case2.2/data/dbsnp_138.b37.vcf --
output-mode EMIT_VARIANTS_ONLY --max-reads-per-alignment-start
1000000 --create-output-variant-index true --standard-min-confidence-
threshold-for-calling 30.0 --standard-min-confidence-threshold-for-emitting
30.0

conda activate abc
gatk VariantFiltration -R ./experiments/case2.2/data/hg38.fa -
V ./experiments/case2.3/output/structural_variations.vcf -
O ./experiments/case2.3/output/filtered_structural_variations.vcf --filter-
name "GATKStandard" --filter-expression "QD < 2.0 || FS > 60.0 || MQ <
40.0 || MQRankSum < -12.5 || ReadPosRankSum < -8.0"
gatk SortVariants -R ./experiments/case2.2/data/hg38.fa -
V ./experiments/case2.3/output/filtered_structural_variations.vcf -
O ./experiments/case2.3/output/sorted_filtered_structural_variations.vcf

conda activate abc
IGV -
D ./experiments/case2.3/output/sorted_filtered_structural_variations.vcf</td>
</tr>
</table>

WGS/WES data analysis **Case 2.4: Structure variation identification without normal**

<table>
<tr><td>User's Inputs</td><td>Data and Description
SRR23015876_R1.fastq.gz: paired end read1 from human whole genome sequencing, tumor samples replicate 1
SRR23015876_R2.fastq.gz: paired end read2 from human whole genome sequencing, tumor samples replicate 1
SRR23015877_R1.fastq.gz: paired end read1 from human whole genome sequencing, tumor samples replicate 2
SRR23015877_R2.fastq.gz: paired end read2 from human whole genome sequencing, tumor samples replicate 2
TruSeq3-PE.fa: trimming adapter
hg38.fa: human hg38 reference genome fasta

Goal
To identify structural variations, which can include large insertions, deletions, inversions, duplications, and translocations based on the sequencing data from the tumor sample.</td></tr>
<tr><td>Generated Plans by AutoBA</td><td>"step 1: Trimming the adapters from the paired-end reads using TruSeq3-PE.fa", "step 2: Mapping the trimmed reads to the human hg38 reference genome using STAR", "step 3: Identifying structural variations using GATK and Annovar", "step 4: Filtering and prioritizing the structural variations using custom scripts and databases", "step 5: Visualizing and interpreting the results using a variety of tools and software"</td></tr>
<tr><td>Generated Codes by AutoBA</td><td>conda activate abc
conda install -y trimmomatic
trimmomatic PE -threads 4 ./experiments/case2.4/data/SRR23015876_R1.fastq.gz ./experiments/case2.4/data/SRR23015876_R2.fastq.gz ./experiments/case2.4/data/TruSeq3-PE.fa ./experiments/case2.4/data/SRR23015876_R1.fastq.gz ./experiments/case2.4/data/SRR23015876_R2.fastq.gz ./experiments/case2.4/data/TruSeq3-PE.fa

conda activate abc
conda install -y star
STAR --runThreadN 4 --genomeDir ./experiments/case2.4/data/hg38.fa --readFilesIn ./experiments/case2.4/data/SRR23015876_R1.fastq.gz ./experiments/case2.4/data/SRR23015876_R2.fastq.gz ./experiments/case2.4/data/SRR23015877_R1.fastq.gz ./experiments/case2.4/data/SRR23015877_R2.fastq.gz --outFileNamePrefix ./experiments/case2.4/output/ --outSAMtype BAM SortedByCoordinate --outSAMunmapped Within --outSAMattributes NH HI AS NM MD

conda activate abc
conda install -y gatk
gatk --java-options '-Xmx4g' HaplotypeCaller -R ./experiments/case2.4/data/hg38.fa -I ./experiments/case2.4/output/Aligned.sortedByCoord.out.bam -O ./experiments/case2.4/output/HaplotypeCaller.vcf

conda activate abc
conda install -y samtools</td></tr>
</table>

	samtools view -<br>b ./experiments/case2.4/output/Aligned.sortedByCoord.out.bam I samtools sort - ./experiments/case2.4/output/Aligned.sortedByCoord.out.bam.sorted<br>samtools<br>index ./experiments/case2.4/output/Aligned.sortedByCoord.out.bam.sorted<br><br><br>conda activate abc<br>conda install -y plotly<br>plotly --html ./experiments/case2.4/output/HaplotypeCaller.vcf.html --title 'Structural Variants' --xlabel 'Chromosome' --ylabel 'Position' --zlabel 'Allele Frequency' --color 'blue' --legend 'Structural Variants' --size 1000 1000 --output ./experiments/case2.4/output/HaplotypeCaller.vcf.png

ChIP-Seq Analysis   Case 3.1: Call Peaks

<table>
<tr><td>User's Inputs</td><td>Data and Description
SRR620204.fastq.gz: chip-seq data for Ring1B
SRR620205.fastq.gz: chip-seq data for cbx7
SRR620206.fastq.gz: chip-seq data for SUZ12
SRR620208.fastq.gz: chip-seq data for IgGold
mm39.ncbiRefSeq.gtf: genome annotations mouse
mm39.fa: mouse genome

Goal
Call peaks for protein cbx7 with IgGold as control</td></tr>
<tr><td>Generated Plans by AutoBA</td><td>"step 1: trim and filter the fastq files using Trimmomatic or Fastp", "step 2: align the filtered reads to the mouse genome using BWA or Bowtie", "step 3: sort and index the aligned reads using Samtools", "step 4: count the reads in the peaks using featureCounts from Subread", "step 5: normalize the read counts using TMM normalization from edgeR", "step 6: identify the differentially bound regions using DiffBind or HISAT2", "step 7: visualize the results using UCSC Genome Browser or Integrative Genomics Viewer", "step 8: interpret the results and draw conclusions about the binding of cbx7 and IgGold"</td></tr>
<tr><td>Generated Codes by AutoBA</td><td>conda activate abc
conda install trimmomatic -y
trimmomatic PE -threads
4 ./experiments/case3.1/data/SRR620204.fastq.gz ./experiments/case3.1/data/SRR620205.fastq.gz ./experiments/case3.1/data/SRR620206.fastq.gz ./experiments/case3.1/data/SRR620208.fastq.gz ./experiments/case3.1/data/mm39.ncbiRefSeq.gtf ./experiments/case3.1/data/mm39.fa ./experiments/case3.1/output

conda activate abc
conda install bwa -y
bwa mem -t
4 ./experiments/case3.1/data/SRR620204_1P.fastq.gz ./experiments/case3.1/data/SRR620205_1P.fastq.gz ./experiments/case3.1/data/SRR620206_1P.fastq.gz ./experiments/case3.1/data/SRR620208_1P.fastq.gz ./experiments/case3.1/data/mm39.ncbiRefSeq.gtf ./experiments/case3.1/data/mm39.fa ./experiments/case3.1/output

conda activate abc
conda install samtools -y
samtools sort -@
4 ./experiments/case3.1/data/SRR620204_1P.fastq.gz ./experiments/case3.1/data/SRR620205_1P.fastq.gz ./experiments/case3.1/data/SRR620206_1P.fastq.gz ./experiments/case3.1/data/SRR620208_1P.fastq.gz ./experiments/case3.1/data/mm39.ncbiRefSeq.gtf ./experiments/case3.1/data/mm39.fa ./experiments/case3.1/output

conda activate abc
conda install subread -y
featureCounts -p -T 4 -
a ./experiments/case3.1/data/SRR620204_1P.fastq.gz ./experiments/case3.1/data/SRR620205_1P.fastq.gz ./experiments/case3.1/data/SRR620206_1</td></tr>
</table>

	P.fastq.gz ./experiments/case3.1/data/SRR620208_1P.fastq.gz ./experiments/case3.1/data/mm39.ncbiRefSeq.gtf ./experiments/case3.1/data/mm39.fa ./experiments/case3.1/output<br><br>conda activate abc<br>conda install edgeR -y<br>edger count -t 4 -a ./experiments/case3.1/data/SRR620204_1P.fastq.gz ./experiments/case3.1/data/SRR620205_1P.fastq.gz ./experiments/case3.1/data/SRR620206_1P.fastq.gz ./experiments/case3.1/data/SRR620208_1P.fastq.gz ./experiments/case3.1/data/mm39.ncbiRefSeq.gtf ./experiments/case3.1/data/mm39.fa ./experiments/case3.1/output<br><br>conda activate abc<br>conda install diffbind -y<br>diffbind -p 4 -t ./experiments/case3.1/data/SRR620204_1P.fastq.gz ./experiments/case3.1/data/SRR620205_1P.fastq.gz ./experiments/case3.1/data/SRR620206_1P.fastq.gz ./experiments/case3.1/data/SRR620208_1P.fastq.gz ./experiments/case3.1/data/mm39.ncbiRefSeq.gtf ./experiments/case3.1/data/mm39.fa ./experiments/case3.1/output<br><br>conda activate abc<br>conda install igv -y<br>igv -b ./experiments/case3.1/data/SRR620204_1P.fastq.gz ./experiments/case3.1/data/SRR620205_1P.fastq.gz ./experiments/case3.1/data/SRR620206_1P.fastq.gz ./experiments/case3.1/data/SRR620208_1P.fastq.gz ./experiments/case3.1/data/mm39.ncbiRefSeq.gtf ./experiments/case3.1/data/mm39.fa ./experiments/case3.1/output<br><br>conda activate abc<br>conda install diffbind -y<br>diffbind -p 4 -t ./experiments/case3.1/data/SRR620204_1P.fastq.gz ./experiments/case3.1/data/SRR620205_1P.fastq.gz ./experiments/case3.1/data/SRR620206_1P.fastq.gz ./experiments/case3.1/data/SRR620208_1P.fastq.gz ./experiments/case3.1/data/mm39.ncbiRefSeq.gtf ./experiments/case3.1/data/mm39.fa ./experiments/case3.1/output

ChIP-Seq Analysis   Case 3.2: Motif Discovery

**User's Inputs**	**Data and Description**<br>SRR620204.fastq.gz: chip-seq data for Ring1B<br>SRR620205.fastq.gz: chip-seq data for cbx7<br>SRR620206.fastq.gz: chip-seq data for SUZ12<br>SRR620208.fastq.gz: chip-seq data for IgGold<br>mm39.ncbiRefSeq.gtf: genome annotations mouse<br>mm39.fa: mouse genome<br><br>**Goal**<br>Discover motifs within the peaks of protein SUZ12 with IgGold as control
**Generated Plans by AutoBA**	"Step 1: Preprocessing of the data", "Step 2: Mapping of the reads to the genome", "Step 3: Peak calling and annotation of the peaks", "Step 4: Motif discovery in the peaks of SUZ12 with IgGold as control", "Step 5: Visualization and interpretation of the results"
**Generated Codes by AutoBA**	conda activate abc<br>fastqc ./experiments/case3.2/data/SRR620204.fastq.gz ./experiments/case3.2/data/SRR620205.fastq.gz ./experiments/case3.2/data/SRR620206.fastq.gz ./experiments/case3.2/data/SRR620208.fastq.gz -o ./experiments/case3.2/output -t 4<br><br>conda activate abc<br>hisat2-build ./experiments/case3.2/data/mm39.fa ./experiments/case3.2/data/mm39.fa.hisat2<br>hisat2 -p 4 --dta -x ./experiments/case3.2/data/mm39.fa.hisat2 -1 ./experiments/case3.2/data/SRR620204.fastq.gz -2 ./experiments/case3.2/data/SRR620205.fastq.gz -3 ./experiments/case3.2/data/SRR620206.fastq.gz -4 ./experiments/case3.2/data/SRR620208.fastq.gz -S ./experiments/case3.2/output/hisat2.sam<br><br><br>conda activate abc<br>bedtools genomecov -ibam ./experiments/case3.2/output/hisat2.bam -bg > ./experiments/case3.2/output/hisat2.bedGraph<br>bedtools bamtobed -i ./experiments/case3.2/output/hisat2.bam > ./experiments/case3.2/output/hisat2.bed<br><br>conda activate abc<br>mkdir ./experiments/case3.2/output/meme<br>cd ./experiments/case3.2/output/meme<br>fasta-get-markov -m 0 -v ./experiments/case3.2/data/mm39.fa > ./experiments/case3.2/output/meme/background.txt<br>fasta-get-markov -m 0 -v ./experiments/case3.2/data/SRR620204.fastq.gz > ./experiments/case3.2/output/meme/SRR620204.txt<br>fasta-get-markov -m 0 -v ./experiments/case3.2/data/SRR620205.fastq.gz > ./experiments/case3.2/output/meme/SRR620205.txt

	fasta-get-markov -m 0 -<br>v ./experiments/case3.2/data/SRR620206.fastq.gz > ./experiments/case3.2/<br>output/meme/SRR620206.txt<br>fasta-get-markov -m 0 -<br>v ./experiments/case3.2/data/SRR620208.fastq.gz > ./experiments/case3.2/<br>output/meme/SRR620208.txt<br>MEME ./experiments/case3.2/output/meme/SRR620204.txt ./experiments/c<br>ase3.2/output/meme/SRR620205.txt ./experiments/case3.2/output/meme/S<br>RR620206.txt ./experiments/case3.2/output/meme/SRR620208.txt -<br>oc ./experiments/case3.2/output/meme/meme_output -<br>b ./experiments/case3.2/output/meme/background.txt -n 1 -m 0 -dna -<br>maxsize 14 -minw 5 -maxw 20 -objfunc classic -revcomp -nmotifs 3 -mod<br>zoops -o ./experiments/case3.2/output/meme/meme_output.html<br><br><br>conda activate abc<br>mkdir ./experiments/case3.2/output/meme<br>cd ./experiments/case3.2/output/meme<br>fasta-get-markov -m 0 -<br>v ./experiments/case3.2/data/mm39.fa > ./experiments/case3.2/output/mem<br>e/background.txt<br>fasta-get-markov -m 0 -<br>v ./experiments/case3.2/data/SRR620204.fastq.gz > ./experiments/case3.2/<br>output/meme/SRR620204.txt<br>fasta-get-markov -m 0 -<br>v ./experiments/case3.2/data/SRR620205.fastq.gz > ./experiments/case3.2/<br>output/meme/SRR620205.txt<br>fasta-get-markov -m 0 -<br>v ./experiments/case3.2/data/SRR620206.fastq.gz > ./experiments/case3.2/<br>output/meme/SRR620206.txt<br>fasta-get-markov -m 0 -<br>v ./experiments/case3.2/data/SRR620208.fastq.gz > ./experiments/case3.2/<br>output/meme/SRR620208.txt<br>MEME ./experiments/case3.2/output/meme/SRR620204.txt ./experiments/c<br>ase3.2/output/meme/SRR620205.txt ./experiments/case3.2/output/meme/S<br>RR620206.txt ./experiments/case3.2/output/meme/SRR620208.txt -<br>oc ./experiments/case3.2/output/meme/meme_output -<br>b ./experiments/case3.2/output/meme/background.txt -n 1 -m 0 -dna -<br>maxsize 14 -minw 5 -maxw 20 -objfunc classic -revcomp -nmotifs 3 -mod<br>zoops -o ./experiments/case3.2/output/meme/meme_output.html<br>.

ChIP-Seq Analysis Case 3.3: Functional Enrichment

**User's Inputs**	**Data and Description**<br>SRR620204.fastq.gz: chip-seq data for Ring1B<br>SRR620205.fastq.gz: chip-seq data for cbx7<br>SRR620206.fastq.gz: chip-seq data for SUZ12<br>SRR620208.fastq.gz: chip-seq data for IgGold<br>mm39.ncbiRefSeq.gtf: genome annotations mouse<br>mm39.fa: mouse genome<br><br>**Goal**<br>Perform functional enrichment for protein Ring1B, use protein IgGold as the control
**Generated Plans by AutoBA**	"step 1: Data Preprocessing", "step 2: Feature Extraction", "step 3: Functional Enrichment Analysis", "step 4: Results Interpretation"
**Generated Codes by AutoBA**	conda activate abc<br>conda install -y fastqc<br>fastqc ./experiments/case3.3/data/SRR620204.fastq.gz ./experiments/case3.3/data/SRR620205.fastq.gz ./experiments/case3.3/data/SRR620206.fastq.gz ./experiments/case3.3/data/SRR620208.fastq.gz<br>conda install -y trim-galore<br>trim_galore --<br>fastqc ./experiments/case3.3/data/SRR620204.fastq.gz ./experiments/case3.3/data/SRR620205.fastq.gz ./experiments/case3.3/data/SRR620206.fastq.gz ./experiments/case3.3/data/SRR620208.fastq.gz<br>conda install -y fastx-toolkit<br>fastx_quality_trimmer -i ./experiments/case3.3/data/SRR620204.fastq.gz -o ./experiments/case3.3/data/SRR620204_trimmed.fastq.gz<br>fastx_quality_trimmer -i ./experiments/case3.3/data/SRR620205.fastq.gz -o ./experiments/case3.3/data/SRR620205_trimmed.fastq.gz<br>fastx_quality_trimmer -i ./experiments/case3.3/data/SRR620206.fastq.gz -o ./experiments/case3.3/data/SRR620206_trimmed.fastq.gz<br>fastx_quality_trimmer -i ./experiments/case3.3/data/SRR620208.fastq.gz -o ./experiments/case3.3/data/SRR620208_trimmed.fastq.gz<br>conda install -y bwa<br>bwa<br>mem ./experiments/case3.3/data/mm39.fa ./experiments/case3.3/data/SRR620204_trimmed.fastq.gz ./experiments/case3.3/data/SRR620205_trimmed.fastq.gz ./experiments/case3.3/data/SRR620206_trimmed.fastq.gz ./experiments/case3.3/data/SRR620208_trimmed.fastq.gz > ./experiments/case3.3/data/SRR620204_SRR620205_SRR620206_SRR620208.sam<br>conda install -y samtools<br>samtools view -b ./experiments/case3.3/data/SRR620204_SRR620205_SRR620206_SRR620208.sam > ./experiments/case3.3/data/SRR620204_SRR620205_SRR620206_SRR620208.bam<br>conda install -y picard<br>java -jar picard.jar MarkDuplicates<br>INPUT=./experiments/case3.3/data/SRR620204_SRR620205_SRR620206_SRR620208.bam<br>OUTPUT=./experiments/case3.3/data/SRR620204_SRR620205_SRR620206_SRR620208_marked_duplicates.bam

	METRICS_FILE=./experiments/case3.3/data/SRR620204_SRR620205_SR<br>R620206_SRR620208_marked_duplicates_metrics.txt<br>conda install -y bedtools<br>bedtools genomecov -<br>ibam ./experiments/case3.3/data/SRR620204_SRR620205_SRR620206_S<br>RR620208_marked_duplicates.bam -<br>bg > ./experiments/case3.3/data/SRR620204_SRR620205_SRR620206_S<br>RR620208_marked_duplicates.bedgraph<br>conda install -y deepTools<br>deepTools bamCoverage -<br>b ./experiments/case3.3/data/SRR620204_SRR620205_SRR620206_SRR<br>620208_marked_duplicates.bam -<br>o ./experiments/case3.3/data/SRR620204_SRR620205_SRR620206_SRR<br>620208_marked_duplicates.bw<br>conda install -y bedtools<br>bedtools genomecov -<br>ibam ./experiments/case3.3/data/SRR620204_SRR620205_SRR620206_S<br>RR620208_marked_duplicates.bam -<br>bg > ./experiments/case3.3/data/SRR620204_SRR620205_SRR620206_S<br>RR620208_marked_duplicates.bedgraph<br>conda install -y deepTools<br>deepTools bamCoverage -<br>b ./experiments/case3.3/data/SRR620204_SRR620205_SRR620206_SRR<br>620208_marked_duplicates.bam -<br>o ./experiments/case3.3/data/SRR620204_SRR620205_SRR620206_SRR<br>620208_marked_duplicates.bw<br><br>bedtools genomecov -<br>ibam ./experiments/case3.3/data/SRR620204_SRR620205_SRR620206_S<br>RR620208_marked_duplicates.bam -<br>bg > ./experiments/case3.3/data/SRR620204_SRR620205_SRR620206_S<br>RR620208_marked_duplicates.bedgraph<br><br>conda install -y deepTools<br>deepTools bamCoverage -<br>b ./experiments/case3.3/data/SRR620204_SRR620205_SRR620206_SRR<br>620208_marked_duplicates.bam -<br>o ./experiments/case3.3/data/SRR620204_SRR620205_SRR620206_SRR<br>620208_marked_duplicates.bw<br><br><br>deepTools bamCoverage -<br>b ./experiments/case3.3/data/SRR620204_SRR620205_SRR620206_SRR<br>620208_marked_duplicates.bam -<br>o ./experiments/case3.3/data/SRR620204_SRR620205_SRR620206_SRR<br>620208_marked_duplicates.bw

Bisulfite-Seq data analysis Case 4.1: Identifying DNA methylation

**User's Inputs**	**Data and Description**<br>Sample_R1.fastq.gz: Raw sequencing reads from the bisulfite-converted DNA, typically paired-end reads. These reads contain information about the converted DNA, where unmethylated cytosines are converted to uracils (T) during library preparation.<br>Sample_R2.fastq.gz: Paired-end reads that complement the R1 reads. These are typically used for quality control and paired-end analysis.<br>TruSeq3-PE.fa: Adapter sequences for trimming<br>Reference.fasta: Reference genome for the organism under study in FASTA format, include bisulfite conversion.<br><br>**Goal**<br>To identify DNA methylation patterns in the genome based on the Bisulfite-Seq data.
**Generated Plans by AutoBA**	"step 1: Preprocessing of the raw sequencing data to remove adapter sequences and low-quality reads using the software 'Trimmomatic'.", "step 2: Align the trimmed reads to the reference genome using the software 'Bowtie2'.", "step 3: Sort and index the aligned reads using the software 'Samtools'.", "step 4: Call methylation sites and generate a methylation frequency plot using the software 'MethylDackel'.", "step 5: Visualize the methylation frequency plot using the software 'Integrative Genomics Viewer (IGV)'.", "step 6: Identify regions of the genome with significant differences in methylation between samples using the software 'Deseq2'.", "step 7: Annotate the differentially methylated regions using the software 'Annovar'.", "step 8: Functional enrichment analysis of the differentially methylated regions using the software 'DAVID'.", "step 9: Generate a report of the results, including the preprocessing methods, alignment statistics, methylation frequency plots, and functional enrichment analysis.", "step 10: Present the results in a clear and concise manner, highlighting the key findings and implications for further research."
**Generated Codes by AutoBA**	conda install trimmomatic -y && trimmomatic PE -phred33 ./experiments/case4.1/data/Sample_R1.fastq.gz ./experiments/case4.1/data/Sample_R2.fastq.gz ./experiments/case4.1/output/Sample_R1.fastq.gz ./experiments/case4.1/output/Sample_R2.fastq.gz ILLUMINACLIP:./experiments/case4.1/data/TruSeq3-PE.fa:2:30:10 LEADING:3 TRAILING:3 SLIDINGWINDOW:4:15 MINLEN:36<br><br>conda install bowtie2 -y && bowtie2-build ./experiments/case4.1/data/Reference.fasta ./experiments/case4.1/output/Reference.fasta && bowtie2 -x ./experiments/case4.1/output/Reference.fasta -1 ./experiments/case4.1/output/Sample_R1.fastq.gz -2 ./experiments/case4.1/output/Sample_R2.fastq.gz -S ./experiments/case4.1/output/Sample.sam<br><br>conda install samtools -y && samtools sort ./experiments/case4.1/output/Sample.sam -o ./experiments/case4.1/output/Sample.bam && samtools index ./experiments/case4.1/output/Sample.bam

	conda install methyldackel -y && methyldackel -b ./experiments/case4.1/output/Sample.bam -r ./experiments/case4.1/data/Reference.fasta -o ./experiments/case4.1/output/Sample.methylation.bed<br><br>conda install igv -y && igv ./experiments/case4.1/output/Sample.methylation.bed<br><br>conda install deseq2 -y && deseq2 -b ./experiments/case4.1/output/Sample.bam -r ./experiments/case4.1/data/Reference.fasta -o ./experiments/case4.1/output/Sample.methylation.bed<br><br>conda install annovar -y && annovar ./experiments/case4.1/output/Sample.methylation.bed ./experiments/case4.1/data/Reference.fasta ./experiments/case4.1/output/Sample.methylation.annotated.bed<br><br>conda install david -y && david ./experiments/case4.1/output/Sample.methylation.annotated.bed<br><br>methyldackel -b ./experiments/case4.1/output/Sample.bam -r ./experiments/case4.1/data/Reference.fasta -o ./experiments/case4.1/output/Sample.methylation.bed<br><br>methyldackel -b ./experiments/case4.1/output/Sample.bam -r ./experiments/case4.1/data/Reference.fasta -o ./experiments/case4.1/output/Sample.methylation.bed

ATAC-seq data analysis **Case 5.1: Identifying open chromatin regions**

**User's Inputs**	**Data and Description**<br>Sample_R1.fastq.gz: Raw sequencing reads from the ATAC-seq experiment. These are typically paired-end reads representing fragments of open chromatin regions.<br>Sample_R2.fastq.gz: Paired-end reads that complement the R1 reads.<br>TruSeq3-PE.fa: Adapter sequences for trimming<br>Reference.fasta: Reference genome for the organism under study in FASTA format. It matches the genome used for the ATAC-seq library preparation.<br><br>**Goal**<br>To identify open chromatin regions within the genome based on the ATAC-seq data.
**Generated Plans by AutoBA**	"step 1: Trim the raw sequencing reads using TruSeq3-PE.fa adapters", "step 2: Map the trimmed reads to the reference genome using a suitable aligner such as BWA", "step 3: Sort and index the mapped reads using SAMtools", "step 4: Identify open chromatin regions using a peak-calling algorithm such as MACS2 or HISAT2", "step 5: Annotate the identified peaks using a tool such as HISAT2 or DAM-IDLE", "step 6: Visualize the identified peaks using a tool such as UCSC Genome Browser or Integrative Genomics Viewer"
**Generated Codes by AutoBA**	conda activate abc<br>conda install -y trimmomatic<br>trimmomatic PE -threads<br>4 ./experiments/case5.1/data/Sample_R1.fastq.gz ./experiments/case5.1/data/Sample_R2.fastq.gz ./experiments/case5.1/data/TruSeq3-PE.fa ./experiments/case5.1/data/Sample_R1_paired.fastq.gz ./experiments/case5.1/data/Sample_R1_unpaired.fastq.gz ./experiments/case5.1/data/Sample_R2_paired.fastq.gz ./experiments/case5.1/data/Sample_R2_unpaired.fastq.gz<br><br>conda activate abc<br>conda install -y bwa<br>bwa mem -t<br>4 ./experiments/case5.1/data/Sample_R1_paired.fastq.gz ./experiments/case5.1/data/Sample_R2_paired.fastq.gz ./experiments/case5.1/data/Reference.fasta > ./experiments/case5.1/output/sample.sam<br><br>conda activate abc<br>conda install -y samtools<br>sort -<br>o ./experiments/case5.1/output/sample.bam ./experiments/case5.1/output/sample.sam<br><br><br>conda activate abc<br>conda install -y macs2<br>macs2 callpeak -t ./experiments/case5.1/data/Sample_R1_paired.fastq.gz -<br>c ./experiments/case5.1/data/Sample_R2_paired.fastq.gz -f BAM -g hs -<br>n ./experiments/case5.1/output/sample --<br>outdir ./experiments/case5.1/output

	conda activate abc<br>conda install -y hisat2<br>hisat2-<br>build ./experiments/case5.1/data/Reference.fasta ./experiments/case5.1/output/reference_genome<br>hisat2-splice-site-profiler ./experiments/case5.1/output/reference_genome<br>hisat2-inspect ./experiments/case5.1/output/reference_genome<br><br><br>conda activate abc<br>conda install -y ucsc-genome-browser<br>ucsc-genome-browser -db=./experiments/case5.1/data/Reference.fasta -<br>g=./experiments/case5.1/output/sample.bed -<br>o=./experiments/case5.1/output/sample.png

DNase-seq data analysis **Case 6.1: Identifying DnaseI hypersensitive site**

**User's Inputs**	**Data and Description**<br>Sample_R1.fastq.gz: Raw sequencing reads from the DNase-seq experiment.<br>Sample_R2.fastq.gz: Paired-end reads that complement the R1 reads, often used for quality control and paired-end analysis.<br>TruSeq3-PE.fa: Adapter sequences for trimming.<br>Reference.fasta: Reference genome for the organism under study in FASTA format<br><br>**Goal**<br>To identify DNase I hypersensitive sites (regions of open chromatin) within the genome based on the DNase-seq data.
**Generated Plans by AutoBA**	"step 1: Trim the raw sequencing reads using TruSeq3-PE.fa adapter sequences to remove low-quality reads and adapter sequences.", "step 2: Map the trimmed reads to the reference genome using software such as BWA or Bowtie.", "step 3: Count the number of reads that map to each genomic position to generate a signal track that represents the DNase I hypersensitivity of each position.", "step 4: Use the signal track to identify regions of open chromatin by identifying areas with high DNase I hypersensitivity.", "step 5: Annotate the identified regions of open chromatin using software such as Aiden or DREAM to identify functional elements such as promoters, enhancers, and other regulatory regions.", "step 6: Visualize the results using software such as UCSC Genome Browser or Integrative Genomics Viewer to identify patterns and trends in the distribution of DNase I hypersensitive sites and their overlap with functional elements."
**Generated Codes by AutoBA**	conda install trimmomatic -y && trimmomatic PE -phred33 ./experiments/case6.1/data/Sample_R1.fastq.gz ./experiments/case6.1/data/Sample_R2.fastq.gz ./experiments/case6.1/data/TruSeq3-PE.fa ./experiments/case6.1/output/Sample_R1_trimmed.fastq.gz ./experiments/case6.1/output/Sample_R1_trimmed.fastq.gz ILLUMINACLIP:./experiments/case6.1/data/TruSeq3-PE.fa:2:30:10 LEADING:3 TRAILING:3 SLIDINGWINDOW:4:15 MINLEN:36<br><br>conda install bwa -y && bwa mem -t 4 ./experiments/case6.1/data/Reference.fasta ./experiments/case6.1/output/Sample_R1_trimmed.fastq.gz ./experiments/case6.1/output/Sample_R2_trimmed.fastq.gz > ./experiments/case6.1/output/Sample.sam<br><br>conda install samtools -y && samtools view -bS ./experiments/case6.1/output/Sample.sam > ./experiments/case6.1/output/Sample.bam && samtools sort ./experiments/case6.1/output/Sample.bam -o ./experiments/case6.1/output/Sample_sorted.bam && samtools index ./experiments/case6.1/output/Sample_sorted.bam<br><br>conda install bedtools -y && bedtools genomecov -ibam ./experiments/case6.1/output/Sample_sorted.bam -bg > ./experiments/case6.1/output/Sample_sorted.bedGraph<br><br>conda install aiden -y && aiden annotate -i ./experiments/case6.1/output/Sample_sorted.bedGraph -

	o ./experiments/case6.1/output/Sample_annotated.bed -<br>r ./experiments/case6.1/data/Reference.fasta<br><br>conda install -c bioconda igv -y &&<br>igv ./experiments/case6.1/output/Sample_annotated.bed

4C-seq data analysis   Case 7.1: Find genomics interactions

<table>
<tr><td>User's Inputs</td><td>Data and Description
sample_4C-seq_data.fastq: Raw sequencing reads or aligned data from the 4C-seq experiment, depending on the starting point.
viewpoint_coordinates.bed: A file specifying the genomic coordinates of the viewpoint we want to study.
reference_genome.fasta: A reference genome in FASTA format for the organism under study. This should match the genome used for the 4C-seq library preparation.

Goal
To identify genomic interactions originating from a specific genomic viewpoint using 4C-seq data.</td></tr>
<tr><td>Generated Plans by AutoBA</td><td>"step 1: preprocessing of the raw sequencing reads or aligned data from the 4C-seq experiment using software such as fastqc and trimmomatic to remove low-quality reads and adapters.", "step 2: alignment of the preprocessed reads to the reference genome using software such as bowtie or hisat2 to identify the genomic coordinates of the aligned reads.", "step 3: calculation of the genomic interaction frequencies using software such as juicer or hiccups to identify the interactions between different genomic regions.", "step 4: visualization of the genomic interactions using software such as circos or ucsc genome browser to visualize the interactions in a circular layout or as a heatmap.", "step 5: statistical analysis of the genomic interactions using software such as R or python to identify the statistically significant interactions and their functional implications."</td></tr>
<tr><td>Generated Codes by AutoBA</td><td>conda install -y fastqc && fastqc ./experiments/case7.1/data/sample_4C-seq_data.fastq -o ./experiments/case7.1/output

conda install -y bowtie && bowtie -p 4 -v 1 ./experiments/case7.1/data/sample_4C-seq_data.fastq -S ./experiments/case7.1/output/sample_4C-seq_data.sam -f ./experiments/case7.1/data/reference_genome.fasta

conda install -y juicer && juicer -g ./experiments/case7.1/data/reference_genome.fasta -s ./experiments/case7.1/output/sample_4C-seq_data.sam -o ./experiments/case7.1/output/interactions.txt

conda install -y circos && circos -conf ./experiments/case7.1/output/interactions.txt -outputdir ./experiments/case7.1/output/circos_output

conda install -y r-base && Rscript -e "library(ggplot2); library(reshape2); df <- read.table('./experiments/case7.1/output/interactions.txt', header=TRUE); df_melt <- melt(df, id.vars=c('chrom1', 'chrom2')); ggplot(df_melt, aes(x=chrom1, y=chrom2, fill=value)) + geom_tile() + scale_fill_gradient(low='white', high='blue') + theme_void() + theme(legend.position='none') + labs(title='Genomic Interactions', x='Chromosome 1', y='Chromosome 2')"</td></tr>
</table>

Nanopore DNA sequencing data analysis Case 8.1: Genome assembly

**User's Inputs**	**Data and Description**<br>sample_nanopore_reads.fastq: Raw Nanopore sequencing data in fastq format.<br>reference_genome.fasta: A reference genome in FASTA format, if a closely related reference is available and used as a guide during the assembly<br><br>**Goal**<br>To assemble a genome from the Nanopore sequencing data.
**Generated Plans by AutoBA**	"step 1: Pre-processing of raw Nanopore sequencing data using the software 'NanoPlot' to remove low-quality reads and adapter sequences.", "step 2: Read trimming and error correction using the software 'NanoFilt' to remove reads with low quality scores and correct errors.", "step 3: Read mapping using the software 'NanoBLAST' to map the trimmed and error-corrected reads to the reference genome.", "step 4: Assembly of the genome using the software 'NanoSCOPE' to generate a draft assembly.", "step 5: Assembly evaluation and refinement using the software 'NanoPOLISH' to assess the quality of the draft assembly and refine it if necessary.", "step 6: Final assembly evaluation using the software 'NanoSCOPE' to assess the quality of the final assembly and verify that it meets the desired specifications."
**Generated Codes by AutoBA**	conda activate abc && conda install -y NanoPlot && NanoPlot ./experiments/case8.1/data/sample_nanopore_reads.fastq -o ./experiments/case8.1/output/sample_nanopore_reads_preprocessed.fastq<br><br>conda activate abc && conda install -y NanoFilt && NanoFilt ./experiments/case8.1/output/sample_nanopore_reads_preprocessed.fastq -o ./experiments/case8.1/output/sample_nanopore_reads_trimmed_and_corrected.fastq<br><br>conda activate abc && conda install -y NanoBLAST && NanoBLAST ./experiments/case8.1/output/sample_nanopore_reads_trimmed_and_corrected.fastq ./experiments/case8.1/data/reference_genome.fasta -o ./experiments/case8.1/output/sample_nanopore_reads_mapped_to_reference.sam<br><br>conda activate abc && conda install -y NanoSCOPE && NanoSCOPE ./experiments/case8.1/output/sample_nanopore_reads_mapped_to_reference.sam -o ./experiments/case8.1/output/draft_assembly.fasta<br><br>conda activate abc && conda install -y NanoPOLISH && NanoPOLISH ./experiments/case8.1/output/draft_assembly.fasta -o ./experiments/case8.1/output/draft_assembly_evaluated_and_refined.fasta<br><br>conda activate abc && conda install -y NanoSCOPE && NanoSCOPE ./experiments/case8.1/output/draft_assembly_evaluated_and_refined.fasta -o ./experiments/case8.1/output/final_assembly.fasta

Nanopore DNA sequencing data analysis   Case 8.2: Tandem repeats variation identification

<table>
<tr><td>User's Inputs</td><td>Data and Description
sample_nanopore_reads.fastq: Raw Nanopore sequencing data in fastq format.
reference_genome.fasta: A reference genome in FASTA format, if a closely related reference is available and used as a guide during the assembly

Goal
To identify tandem repeats.</td></tr>
<tr><td>Generated Plans by AutoBA</td><td>"Step 1: Preprocessing of Nanopore Sequencing Data", "Use a software such as `NanoPlot` to preprocess the raw nanopore sequencing data. This will include trimming the reads, removing low-quality reads, and filtering out adapter sequences.", "Step 2: Reference-based Assembly", "Use a software such as `Canu` or `Miniasm` to perform a reference-based assembly of the preprocessed nanopore sequencing data using the provided reference genome. This will generate a high-quality draft assembly of the genome.", "Step 3: Tandem Repeat Identification", "Use a software such as `Tandem Repeats Finder` or `TRF` to identify tandem repeats in the assembled genome. This will provide information on the location, size, and repeat content of the tandem repeats.", "Step 4: Genome Annotation", "Use a software such as `Prokka` to annotate the genome with features such as genes, promoters, and terminators. This will provide a better understanding of the function and organization of the genome.", "Step 5: Visualization and Interpretation", "Use a software such as `Integrative Genomics Viewer (IGV)` to visualize and interpret the results of the genome assembly and tandem repeat identification. This will provide a better understanding of the organization and function of the genome."</td></tr>
<tr><td>Generated Codes by AutoBA</td><td>source activate abc
conda install -y nano-plot
nano-plot --input ./experiments/case8.2/data/sample_nanopore_reads.fastq --output ./experiments/case8.2/output/sample_nanopore_reads.fastq.nano-plot

source activate abc
conda install -y nano-plot
nano-plot --input ./experiments/case8.2/data/sample_nanopore_reads.fastq --output ./experiments/case8.2/output/sample_nanopore_reads.fastq.nano-plot.

source activate abc
conda install -y canu
canu -d ./experiments/case8.2/output/canu -p assembly -genomeSize 1000000 -
nanopore ./experiments/case8.2/data/sample_nanopore_reads.fastq -correctedReads ./experiments/case8.2/output/sample_nanopore_reads.fastq.nano-plot.fastq

source activate abc
conda install -y canu
canu -d ./experiments/case8.2/output/canu -p assembly -genomeSize 1000000 -
nanopore ./experiments/case8.2/data/sample_nanopore_reads.fastq -</td></tr>
</table>

	correctedReads ./experiments/case8.2/output/sample_nanopore_reads.fastq.nano-plot.fastq<br><br>source activate abc<br>conda install -y trf<br>trf ./experiments/case8.2/output/assembly.fasta ./experiments/case8.2/output/trf.out<br><br><br>source activate abc<br>conda install -y trf<br>trf ./experiments/case8.2/output/assembly.fasta ./experiments/case8.2/output/trf.out<br><br>source activate abc<br>conda install -y prokka<br>prokka ./experiments/case8.2/output/assembly.fasta ./experiments/case8.2/output/prokka.out<br><br>source activate abc<br>conda install -y prokka<br>prokka ./experiments/case8.2/output/assembly.fasta ./experiments/case8.2/output/prokka.out<br><br>source activate abc<br>conda install -y igv<br>igv ./experiments/case8.2/output/assembly.fasta ./experiments/case8.2/output/trf.out ./experiments/case8.2/output/prokka.out<br><br>source activate abc<br>conda install -y igv<br>igv ./experiments/case8.2/output/assembly.fasta ./experiments/case8.2/output/trf.out ./experiments/case8.2/output/prokka.out

PacBio DNA sequencing data analysis **Case 9.1: Genome assembly**

<table>
<tr><td>User’s Inputs</td><td>Data and Description
sample_pacbio_reads.fastq: Raw PacBio sequencing data in fastq format.
eference_genome.fasta: A reference genome in FASTA format, if a closely related reference is available and used as a guide during the assembly

Goal
To assemble a genome from the PacBio sequencing data.</td></tr>
<tr><td>Generated Plans by AutoBA</td><td>"step 1: Pre-processing of raw PacBio sequencing data.", "step 2: Mapping of raw PacBio sequencing data to a reference genome.", "step 3: Assembly of PacBio sequencing data using a de novo assembly algorithm.", "step 4: Evaluation of the assembled genome using a benchmark dataset.", "step 5: Comparison of the assembled genome with the reference genome.", "step 6: Identification of variations between the assembled genome and the reference genome.", "step 7: Annotation of the variations using a gene prediction tool.", "step 8: Identification of potential functional effects of the variations using a variant effect prediction tool.", "step 9: Finalization of the genome assembly and preparation for downstream analyses."</td></tr>
<tr><td>Generated Codes by AutoBA</td><td>conda install -y -c bioconda pbcoretools
pbcoretools
fastq2sra ./experiments/case9.1/data/sample_pacbio_reads.fastq ./experiments/case9.1/output/sample_pacbio_reads.sra

conda install -y -c bioconda minimap2
minimap2 -ax
sr ./experiments/case9.1/data/sample_pacbio_reads.fastq ./experiments/case9.1/data/reference_genome.fasta > ./experiments/case9.1/output/sample_pacbio_reads.sam

conda install -y -c bioconda canu
canu -d ./experiments/case9.1/output/assembly -p canu genomeSize=5m -nanopore ./experiments/case9.1/output/sample_pacbio_reads.fastq

conda install -y -c bioconda canu
canu -d ./experiments/case9.1/output/assembly -p canu genomeSize=5m -nanopore ./experiments/case9.1/output/sample_pacbio_reads.fastq

conda install -y -c bioconda minimap2
minimap2 -ax
sr ./experiments/case9.1/output/sample_pacbio_reads.sra ./experiments/case9.1/data/reference_genome.fasta > ./experiments/case9.1/output/sample_pacbio_reads.sam

conda install -y -c bioconda bcftools
bcftools mpileup -
f ./experiments/case9.1/data/reference_genome.fasta ./experiments/case9.1/output/sample_pacbio_reads.sra | bcftools call -mv -Ob -
o ./experiments/case9.1/output/sample_pacbio_reads.vcf

conda install -y -c bioconda gffread</td></tr>
</table>

	gffread -<br>w ./experiments/case9.1/output/sample_pacbio_reads.gff ./experiments/case9.1/output/sample_pacbio_reads.vcf<br><br><br>conda install -y -c bioconda snpEff<br>snpEff ./experiments/case9.1/output/sample_pacbio_reads.vcf > ./experiments/case9.1/output/sample_pacbio_reads.eff.vcf<br><br>conda install -y -c bioconda canu<br>canu -d ./experiments/case9.1/output/assembly -p canu genomeSize=5m -nanopore ./experiments/case9.1/output/sample_pacbio_reads.fastq

RNA-Seq Analysis Case 10.1: Find differentially expressed genes

**User's Inputs**	**Data and Description**<br>SRR1374921.fastq.gz: single-end mouse rna-seq reads, replicate 1 in LoGlu group<br>SRR1374922.fastq.gz: single-end mouse rna-seq reads, replicate 2 in LoGlu group<br>SRR1374923.fastq.gz: single-end mouse rna-seq reads, replicate 1 in HiGlu group<br>SRR1374924.fastq.gz: single-end mouse rna-seq reads, replicate 2 in HiGlu group<br>TruSeq3-SE.fa: trimming adapter<br>mm39.fa: mouse mm39 genome fasta<br>mm39.ncbiRefSeq.gtf: mouse mm39 genome annotation<br><br>**Goal**<br>Find the differentially expressed genes
**Generated Plans by AutoBA**	"step 1: Trimming the adapters from the fastq files using Trimmomatic.", "step 2: Mapping the trimmed reads to the mouse genome using STAR.", "step 3: Counting the number of reads that map to each gene using featureCounts.", "step 4: Normalizing the read counts using TMM normalization.", "step 5: Identifying the differentially expressed genes using DESeq2.", "step 6: Visualizing the results using a volcano plot."
**Generated Codes by AutoBA**	conda install -y trimmomatic && trimmomatic SE -phred33 ./experiments/case10.1/data/SRR1374921.fastq.gz ./experiments/case10.1/data/SRR1374922.fastq.gz ./experiments/case10.1/data/SRR1374923.fastq.gz ./experiments/case10.1/data/SRR1374924.fastq.gz ILLUMINACLIP:./experiments/case10.1/data/TruSeq3-SE.fa:2:30:10 LEADING:3 TRAILING:3 SLIDINGWINDOW:4:15 MINLEN:36<br><br>conda install -y star && STAR --runMode alignReads --runThreadN 4 --genomeDir /path/to/mouse/genome/index --readFilesIn /path/to/trimmed/fastq/files --outFileNamePrefix /path/to/output/prefix --outSAMtype BAM SortedByCoordinate --outSAMunmapped Within --outSAMattributes NH HI NM MD AS --outReadsUnmapped Fastx --limitBAMsortRAM 10000000000 --outFilterMultimapNmax 1 --outFilterMismatchNmax 2 --outFilterMatchNmin 12<br><br>conda install -y subread && featureCounts -a /path/to/mouse/genome/index -o /path/to/output/prefix -g gene_id -t exon -T 4 /path/to/trimmed/fastq/files<br><br>conda install -y tmm && tmm normalization -i /path/to/featureCounts/output -o /path/to/normalized/output -t TMM<br><br>conda install -y DESeq2 && DESeq2 -i /path/to/normalized/output -o /path/to/output -t TMM -s LoGlu -c HiGlu -m -b 1000 -p 0.05<br><br>conda install -y R && Rscript -e 'library(ggplot2); library(DESeq2); df <- read.table('/path/to/DESeq2/output', header=TRUE, row.names=1); df <- df[,c(1,5,6)]; colnames(df) <- c('gene', 'baseMean', 'log2FoldChange'); df$log10pvalue <- -log10(df$padj); ggplot(df, aes(x=log2FoldChange, y=-log10pvalue, color=gene)) + geom_point() + theme_bw() + scale_color_brewer(type='qual', palette=1) + labs(x='log2 fold change', y='-

	log10 p-value', color='gene') + theme(legend.position='none') + coord_fixed()'

RNA-Seq Analysis   Case 10.2: Identify top5 down-regulated genes in HiGlu group

**User's Inputs**	**Data and Description**<br>SRR1374921.fastq.gz: single-end mouse rna-seq reads, replicate 1 in LoGlu group<br>SRR1374922.fastq.gz: single-end mouse rna-seq reads, replicate 2 in LoGlu group<br>SRR1374923.fastq.gz: single-end mouse rna-seq reads, replicate 1 in HiGlu group<br>SRR1374924.fastq.gz: single-end mouse rna-seq reads, replicate 2 in HiGlu group<br>TruSeq3-SE.fa: trimming adapter<br>mm39.fa: mouse mm39 genome fasta<br>mm39.ncbiRefSeq.gtf: mouse mm39 genome annotation<br><br>**Goal**<br>Identify top5 down-regulated genes in HiGlu group
**Generated Plans by AutoBA**	"step 1: Trimming the adapters from the fastq files using Trimmomatic.", "step 2: Mapping the trimmed reads to the mouse genome using STAR.", "step 3: Counting the number of reads that map to each gene using featureCounts.", "step 4: Normalizing the read counts using TMM normalization.", "step 5: Calculating the fold change between the HiGlu and LoGlu groups using DESeq2.", "step 6: Identifying the top 5 down-regulated genes in the HiGlu group using the fold change and adjusted p-value."
**Generated Codes by AutoBA**	conda install -y trimmomatic<br>trimmomatic SE -threads 4 -phred33 ./experiments/case10.2/data/SRR1374921.fastq.gz ./experiments/case10.2/data/SRR1374922.fastq.gz ./experiments/case10.2/data/SRR1374923.fastq.gz ./experiments/case10.2/data/SRR1374924.fastq.gz ./experiments/case10.2/data/TruSeq3-SE.fa ILLUMINACLIP:./experiments/case10.2/data/TruSeq3-SE.fa:2:30:10 LEADING:3 TRAILING:3 SLIDINGWINDOW:4:15 MINLEN:36<br><br>conda install -y star<br>star --runMode genomeGenerate --genomeDir ./experiments/case10.2/data/star_index --genomeFastaFiles ./experiments/case10.2/data/mm39.fa --sjdbGTFfile ./experiments/case10.2/data/mm39.ncbiRefSeq.gtf --sjdbOverhang 75 --runThreadN 4<br><br>conda install -y featureCounts<br>featureCounts -T 4 -p -t exon -g gene_id -o ./experiments/case10.2/output/counts.txt ./experiments/case10.2/data/SRR1374921.fastq.gz ./experiments/case10.2/data/SRR1374922.fastq.gz ./experiments/case10.2/data/SRR1374923.fastq.gz ./experiments/case10.2/data/SRR1374924.fastq.gz<br>samtools sort -o ./experiments/case10.2/output/sorted.bam ./experiments/case10.2/output/counts.txt<br>samtools index ./experiments/case10.2/output/sorted.bam<br>featureCounts -T 4 -p -t exon -g gene_id -o ./experiments/case10.2/output/sorted_counts.txt ./experiments/case10.2/output/sorted.bam

	Rscript -e "library(DESeq2); data <-<br>read.table('./experiments/case10.2/output/sorted_counts.txt',<br>header=TRUE); colnames(data) <- c('gene_id', 'counts'); design <-<br>data.frame(group=c('HiGlu', 'LoGlu'), replicate=c('1', '2')); contrasts(design)<br><- c(0, 1); fit <- DESeq2(data, design); results <- results(fit);<br>write.table(results, file='./experiments/case10.2/output/results.txt',<br>quote=FALSE, row.names=FALSE)"<br>Rscript -e "library(DESeq2); data <-<br>read.table('./experiments/case10.2/output/results.txt', header=TRUE); top5<br><- data[which(data$padj < 0.05 & data$log2FoldChange < 0), c('gene_id',<br>'log2FoldChange')]; write.table(top5,<br>file='./experiments/case10.2/output/top5.txt', quote=FALSE,<br>row.names=FALSE)"<br>conda create -y -n bioinformatics python=3.6 r-base=3.6.1 samtools=1.9<br>featureCounts=1.6.2 DESeq2=1.28.1<br>conda activate bioinformatics<br>conda deactivate<br><br>conda install -y tmm-normalization<br>tmm-normalization -i ./experiments/case10.2/output/sorted_counts.txt -<br>o ./experiments/case10.2/output/tmm_normalized_counts.txt<br><br>Rscript -e "library(DESeq2); data <-<br>read.table('./experiments/case10.2/output/sorted_counts.txt',<br>header=TRUE); colnames(data) <- c('gene_id', 'counts'); design <-<br>data.frame(group=c('HiGlu', 'LoGlu'), replicate=c('1', '2')); contrasts(design)<br><- c(0, 1); fit <- DESeq2(data, design); results <- results(fit);<br>write.table(results, file='./experiments/case10.2/output/results.txt',<br>quote=FALSE, row.names=FALSE)"<br><br>Rscript -e "library(DESeq2); data <-<br>read.table('./experiments/case10.2/output/results.txt', header=TRUE); top5<br><- data[which(data$padj < 0.05 & data$log2FoldChange < 0), c('gene_id',<br>'log2FoldChange')]; write.table(top5,<br>file='./experiments/case10.2/output/top5.txt', quote=FALSE,<br>row.names=FALSE)"

RNA-Seq Analysis   Case 10.3: Predict Fusion genes

**User's Inputs**	**Data and Description**<br>rnaseq_1.fastq.gz: RNA-Seq read 1 data (left read)<br>rnaseq_2.fastq.gz: RNA-Seq read 2 data (right read)<br>CTAT_HumanFusionLib.mini.dat.gz: a small fusion annotation library<br>minigenome.fa: small genome sequence consisting of ~750 genes<br>minigenome.gtf: transcript structure annotations for these genes<br><br>**Goal**<br>Predict Fusion genes
**Generated Plans by AutoBA**	"step 1: preprocessing of the RNA-Seq data using the software 'FASTQC' to ensure the quality of the reads.", "step 2: trimming of the adapters from the RNA-Seq reads using the software 'Trimmomatic'.", "step 3: mapping of the cleaned reads to the reference genome using the software 'Bowtie2'.", "step 4: identification of the fusion genes using the software 'FusionCatcher'."
**Generated Codes by AutoBA**	source activate abc && conda install -y fastqc && fastqc -<br>o ./experiments/case10.3/output ./experiments/case10.3/data/rnaseq_1.fast<br>q.gz ./experiments/case10.3/data/rnaseq_2.fastq.gz<br><br>source activate abc && conda install -y trimmomatic && trimmomatic -<br>o ./experiments/case10.3/output ./experiments/case10.3/data/rnaseq_1.fast<br>q.gz ./experiments/case10.3/data/rnaseq_2.fastq.gz<br><br>source activate abc && conda install -y bowtie2 && bowtie2-<br>build ./experiments/case10.3/data/minigenome.fa ./experiments/case10.3/o<br>utput/minigenome && bowtie2 -<br>x ./experiments/case10.3/output/minigenome -<br>1 ./experiments/case10.3/data/rnaseq_1.fastq.gz -<br>2 ./experiments/case10.3/data/rnaseq_2.fastq.gz -<br>S ./experiments/case10.3/output/rnaseq_1.sam<br><br>source activate abc && conda install -y fusioncatcher && fusioncatcher -<br>o ./experiments/case10.3/output -<br>t ./experiments/case10.3/data/CTAT_HumanFusionLib.mini.dat.gz -<br>g ./experiments/case10.3/data/minigenome.fa -<br>s ./experiments/case10.3/output/rnaseq_1.sam

RNA-Seq Analysis Case 10.4: isoform expression

<table>
<tr><td>User's Inputs</td><td>Data and Description
SRR14463651_1.fastq.gz: paired end RNA sequencing data from human lymphoma cell, read 1 of lymphoma samples replicate 1
SRR14463651_2.fastq.gz: paired end RNA sequencing data from human lymphoma cell, read 2 of lymphoma samples replicate 1
SRR14463652_1.fastq.gz: paired end RNA sequencing data from human lymphoma cell, read 1 of lymphoma samples replicate 2
SRR14463652_2.fastq.gz: paired end RNA sequencing data from human lymphoma cell, read 2 of lymphoma samples replicate 2
SRR14463680_1.fastq.gz: paired end RNA sequencing data from human lymphoma cell, read 1 of control samples replicate 1
SRR14463680_2.fastq.gz: paired end RNA sequencing data from human lymphoma cell, read 2 of control samples replicate 1
SRR14463681_1.fastq.gz: paired end RNA sequencing data from human lymphoma cell, read 1 of control samples replicate 2
SRR14463681_2.fastq.gz: paired end RNA sequencing data from human lymphoma cell, read 2 of control samples replicate 2
gencode.v44.transcripts.fa: transcriptome annotation
TruSeq3-PE.fa: trimming adapter
Goal
quantify RNA isoform expression based on transcriptome annotation</td></tr>
<tr><td>Generated Plans by AutoBA</td><td>"step 1: preprocessing the raw sequencing data by trimming the adapters and low-quality reads using the trimming adapter file and saving the output in a new file.", "step 2: mapping the trimmed reads to the transcriptome annotation using a software such as STAR, HISAT2, or TopHat, and saving the output in a new file.", "step 3: counting the number of reads that map to each transcript using a software such as featureCounts or RSEM, and saving the output in a new file.", "step 4: normalizing the read counts using a software such as TMM or DESeq2, and saving the output in a new file.", "step 5: visualizing the results using a software such as R or Python, and creating a plot that shows the expression levels of each transcript in each sample."</td></tr>
<tr><td>Generated Codes by AutoBA</td><td>conda install -y trimmomatic
trimmomatic PE -threads
4 ./experiments/case10.4/data/SRR14463651_1.fastq.gz ./experiments/case10.4/data/SRR14463651_2.fastq.gz ./experiments/case10.4/output/SRR14463651_1.trimmed.fastq.gz ./experiments/case10.4/output/SRR14463651_1.unpaired.fastq.gz ./experiments/case10.4/output/SRR14463651_2.trimmed.fastq.gz ./experiments/case10.4/output/SRR14463651_2.unpaired.fastq.gz ILLUMINACLIP:./experiments/case10.4/data/TruSeq3-PE.fa:2:30:10 LEADING:3 TRAILING:3 SLIDINGWINDOW:4:15 MINLEN:36

conda install -y hisat2
hisat2-build -p
4 ./experiments/case10.4/data/gencode.v44.transcripts.fa ./experiments/case10.4/output/gencode.v44.transcripts.fa.hisat2
hisat2 -x ./experiments/case10.4/output/gencode.v44.transcripts.fa.hisat2 -
1 ./experiments/case10.4/output/SRR14463651_1.trimmed.fastq.gz -
2 ./experiments/case10.4/output/SRR14463651_2.trimmed.fastq.gz -
S ./experiments/case10.4/output/SRR14463651.sam</td></tr>
</table>

	conda install -y subread<br>featureCounts -p -t exon -g gene_id -<br>a ./experiments/case10.4/output/gencode.v44.transcripts.fa.hisat2 -<br>o ./experiments/case10.4/output/SRR14463651.counts ./experiments/case1<br>0.4/output/SRR14463651.sam<br><br>conda install -y r-deseq2<br>DESeq2::estimateSizeFactors(countData<br>= ./experiments/case10.4/output/SRR14463651.counts, colData<br>= ./experiments/case10.4/output/SRR14463651.sam, outFile<br>= ./experiments/case10.4/output/SRR14463651.normalized.counts)<br><br>Rscript -e "library(ggplot2);<br>ggplot(data=./experiments/case10.4/output/SRR14463651.normalized.coun<br>ts, aes(x=sample, y=expression, fill=transcript)) + geom_bar(stat='identity',<br>position='dodge') + theme_bw() + labs(title='Expression Levels of Each<br>Transcript in Each Sample', x='Sample', y='Expression Level')"

RNA-seq Analysis Case 10.5: splicing analysis

<table>
<tr><td>User's Inputs</td><td>Data and Description
SRR14463651_1.fastq.gz: paired end RNA sequencing data from human lymphoma cell, read 1 of lymphoma samples replicate 1
SRR14463651_2.fastq.gz: paired end RNA sequencing data from human lymphoma cell, read 2 of lymphoma samples replicate 1
SRR14463652_1.fastq.gz: paired end RNA sequencing data from human lymphoma cell, read 1 of lymphoma samples replicate 2
SRR14463652_2.fastq.gz: paired end RNA sequencing data from human lymphoma cell, read 2 of lymphoma samples replicate 2
SRR14463680_1.fastq.gz: paired end RNA sequencing data from human lymphoma cell, read 1 of control samples replicate 1
SRR14463680_2.fastq.gz: paired end RNA sequencing data from human lymphoma cell, read 2 of control samples replicate 1
SRR14463681_1.fastq.gz: paired end RNA sequencing data from human lymphoma cell, read 1 of control samples replicate 2
SRR14463681_2.fastq.gz: paired end RNA sequencing data from human lymphoma cell, read 2 of control samples replicate 2
hg38.fa: reference genome
gencode.v38.annotation.gtf: gene annotation
TruSeq3-PE.fa: trimming adapter
Goal
identify alternative splicing events in lymphoma and control samples and compare them to get differentially spliced events</td></tr>
<tr><td>Generated Plans by AutoBA</td><td>"Step 1: Trimming and adapter removal", "Step 2: Mapping to the reference genome", "Step 3: Identifying differentially spliced events", "Step 4: Filtering and sorting the results", "Step 5: Visualizing the results"</td></tr>
<tr><td>Generated Codes by AutoBA</td><td>conda install -y trimmomatic && trimmomatic PE -threads 4 -phred33 ./experiments/case10.5/data/SRR14463651_1.fastq.gz ./experiments/case10.5/data/SRR14463651_2.fastq.gz ./experiments/case10.5/data/SRR14463652_1.fastq.gz ./experiments/case10.5/data/SRR14463652_2.fastq.gz ./experiments/case10.5/data/SRR14463680_1.fastq.gz ./experiments/case10.5/data/SRR14463680_2.fastq.gz ./experiments/case10.5/data/SRR14463681_1.fastq.gz ./experiments/case10.5/data/SRR14463681_2.fastq.gz TMPDIR=./experiments/case10.5/output/tmp ILLUMINACLIP=./experiments/case10.5/data/TruSeq3-PE.fa:2:30:10 LEADING=3 TRAILING=3 SLIDINGWINDOW=4:15 MINLEN=36

conda install -y hisat2 && hisat2-build ./experiments/case10.5/data/hg38.intg.virg.fa ./experiments/case10.5/data/hg38.intg.virg.fa.ht2 && hisat2 -p 4 -x ./experiments/case10.5/data/hg38.intg.virg.fa.ht2 -1 ./experiments/case10.5/output/SRR14463651_1.fastq.gz -2 ./experiments/case10.5/output/SRR14463651_2.fastq.gz -S ./experiments/case10.5/output/SRR14463651.sam && hisat2 -p 4 -x ./experiments/case10.5/data/hg38.intg.virg.fa.ht2 -1 ./experiments/case10.5/output/SRR14463652_1.fastq.gz -2 ./experiments/case10.5/output/SRR14463652_2.fastq.gz -S ./experiments/case10.5/output/SRR14463652.sam && hisat2 -p 4 -x ./experiments/case10.5/data/hg38.intg.virg.fa.ht2 -1 ./experiments/case10.5/output/SRR14463680_1.fastq.gz -2 ./experiments/case10.5/output/SRR14463680_2.fastq.gz -S ./experiments/case10.5/output/SRR14463680.sam && hisat2 -p 4 -</td></tr>
</table>

	x ./experiments/case10.5/data/hg38.intg.virg.fa.ht2 -<br>1 ./experiments/case10.5/output/SRR14463681_1.fastq.gz -<br>2 ./experiments/case10.5/output/SRR14463681_2.fastq.gz -<br>S ./experiments/case10.5/output/SRR14463681.sam<br><br>conda install -y stringtie &&<br>stringtie ./experiments/case10.5/output/SRR14463651.sam ./experiments/c<br>ase10.5/output/SRR14463652.sam ./experiments/case10.5/output/SRR144<br>63680.sam ./experiments/case10.5/output/SRR14463681.sam -p 4 -<br>o ./experiments/case10.5/output/stringtie.gtf -<br>G ./experiments/case10.5/data/gencode.v38.annotation.gtf<br><br>conda install -y stringtie &&<br>stringtie ./experiments/case10.5/output/SRR14463651.sam ./experiments/c<br>ase10.5/output/SRR14463652.sam ./experiments/case10.5/output/SRR144<br>63680.sam ./experiments/case10.5/output/SRR14463681.sam -p 4 -<br>o ./experiments/case10.5/output/stringtie.gtf -<br>G ./experiments/case10.5/data/gencode.v38.annotation.gtf<br><br>Rscript -e "library(ggplot2); ggplot(data, aes(x = lymphoma, y = control)) +<br>geom_point() + geom_smooth()"

RNA-seq Analysis   Case 10.6: APA analysis

**User's Inputs**	**Data and Description**<br>SRR14463651_1.fastq.gz: paired end RNA sequencing data from human lymphoma cell, read 1 of lymphoma samples replicate 1<br>SRR14463651_2.fastq.gz: paired end RNA sequencing data from human lymphoma cell, read 2 of lymphoma samples replicate 1<br>SRR14463652_1.fastq.gz: paired end RNA sequencing data from human lymphoma cell, read 1 of lymphoma samples replicate 2<br>SRR14463652_2.fastq.gz: paired end RNA sequencing data from human lymphoma cell, read 2 of lymphoma samples replicate 2<br>SRR14463680_1.fastq.gz: paired end RNA sequencing data from human lymphoma cell, read 1 of control samples replicate 1<br>SRR14463680_2.fastq.gz: paired end RNA sequencing data from human lymphoma cell, read 2 of control samples replicate 1<br>SRR14463681_1.fastq.gz: paired end RNA sequencing data from human lymphoma cell, read 1 of control samples replicate 2<br>SRR14463681_2.fastq.gz: paired end RNA sequencing data from human lymphoma cell, read 2 of control samples replicate 2<br>hg38.fa: reference genome<br>gencode.v38.annotation.gtf: gene annotation<br>TruSeq3-PE.fa: trimming adapter<br>**Goal**<br>identify and quantify alternative polyadenylation between lymphoma and control samples
**Generated Plans by AutoBA**	"Step 1: Trimming and adapter removal", "Use FastQC to evaluate the quality of the raw sequencing data and remove any low-quality reads.", "Use Trimmomatic to trim the reads based on the adapter sequence and any other applicable quality thresholds.", "Step 2: Mapping", "Use STAR or HISAT2 to map the trimmed reads to the reference genome.", "Step 3: Counting", "Use featureCounts or RSEM to count the number of reads that align to each gene.", "Step 4: Differential expression analysis", "Use DESeq2 or edgeR to perform a differential expression analysis between the lymphoma and control samples.", "Step 5: Visualization", "Use R or Python to visualize the results, such as a volcano plot or heatmap.", "Step 6: Annotation", "Use the gene annotation file to identify the biological meaning of the differentially expressed genes."
**Generated Codes by AutoBA**	conda install -y trimmomatic && trimmomatic PE -threads 4 ./experiments/case10.6/data/SRR14463651_1.fastq.gz ./experiments/case10.6/data/SRR14463651_2.fastq.gz ./experiments/case10.6/data/SRR14463652_1.fastq.gz ./experiments/case10.6/data/SRR14463652_2.fastq.gz ./experiments/case10.6/data/SRR14463680_1.fastq.gz ./experiments/case10.6/data/SRR14463680_2.fastq.gz ./experiments/case10.6/data/SRR14463681_1.fastq.gz ./experiments/case10.6/data/SRR14463681_2.fastq.gz ./experiments/case10.6/data/hg38.intg.virg.fa ./experiments/case10.6/data/gencode.v38.annotation.gtf ./experiments/case10.6/data/TruSeq3-PE.fa ./experiments/case10.6/output<br><br>conda install -y star && STAR --runThreadN 4 --genomeDir ./experiments/case10.6/data/hg38.intg.virg.fa --readFilesIn ./experiments/case10.6/output/SRR14463651_1_paired.fq.gz ./experiments/case10.6/output/SRR14463651_2_paired.fq.gz ./experiments/case10.6/output/SRR14463652_1_paired.fq.gz ./experiments/case10.6/output/SRR14463652_2_paired.fq.gz ./experiments/case10.6/output/SRR14463

	680_1_paired.fq.gz ./experiments/case10.6/output/SRR14463680_2_paired.fq.gz ./experiments/case10.6/output/SRR14463681_1_paired.fq.gz ./experiments/case10.6/output/SRR14463681_2_paired.fq.gz --outFileNamePrefix ./experiments/case10.6/output/ --outSAMtype BAM SortedByCoordinate --outReadsUnmapped Fastx --quantMode GeneCounts<br><br>conda install -y subread && featureCounts -T 4 -p -t exon -g gene_id -a ./experiments/case10.6/data/gencode.v38.annotation.gtf -o ./experiments/case10.6/output/counts.txt ./experiments/case10.6/output/Aligned.sortedByCoord.out.bam<br><br>conda install -y subread && featureCounts -T 4 -p -t exon -g gene_id -a ./experiments/case10.6/data/gencode.v38.annotation.gtf -o ./experiments/case10.6/output/counts.txt ./experiments/case10.6/output/Aligned.sortedByCoord.out.bam<br><br>conda install -y multiqc && multiqc ./experiments/case10.6/output/counts.txt -o ./experiments/case10.6/output/multiqc_report.html

RNA-seq Analysis   Case 10.7: RNA editing

**User's Inputs**	**Data and Description**<br>SRR14463651_1.fastq.gz: paired end RNA sequencing data from human lymphoma cell, read 1 of lymphoma samples replicate 1<br>SRR14463651_2.fastq.gz: paired end RNA sequencing data from human lymphoma cell, read 2 of lymphoma samples replicate 1<br>SRR14463652_1.fastq.gz: paired end RNA sequencing data from human lymphoma cell, read 1 of lymphoma samples replicate 2<br>SRR14463652_2.fastq.gz: paired end RNA sequencing data from human lymphoma cell, read 2 of lymphoma samples replicate 2<br>SRR14463680_1.fastq.gz: paired end RNA sequencing data from human lymphoma cell, read 1 of control samples replicate 1<br>SRR14463680_2.fastq.gz: paired end RNA sequencing data from human lymphoma cell, read 2 of control samples replicate 1<br>SRR14463681_1.fastq.gz: paired end RNA sequencing data from human lymphoma cell, read 1 of control samples replicate 2<br>SRR14463681_2.fastq.gz: paired end RNA sequencing data from human lymphoma cell, read 2 of control samples replicate 2<br>hg38.fa: reference genome<br>gencode.v38.annotation.gtf: gene annotation<br>TruSeq3-PE.fa: trimming adapter<br>**Goal**<br>identifying RNA edting events in lymphoma and control samples
**Generated Plans by AutoBA**	"Step 1: Quality Control and Trimming of FastQ files", "Step 2: Mapping of FastQ files to the Reference Genome", "Step 3: Identification of RNA Editing Events using the GATK toolkit", "Step 4: Filtering and Visualization of RNA Editing Events", "Step 5: Statistical Analysis and Interpretation of RNA Editing Events"
**Generated Codes by AutoBA**	conda activate abc<br>fastqc ./experiments/case10.7/data/SRR14463651_1.fastq.gz ./experiments/case10.7/data/SRR14463651_2.fastq.gz ./experiments/case10.7/data/SRR14463652_1.fastq.gz ./experiments/case10.7/data/SRR14463652_2.fastq.gz ./experiments/case10.7/data/SRR14463680_1.fastq.gz ./experiments/case10.7/data/SRR14463680_2.fastq.gz ./experiments/case10.7/data/SRR14463681_1.fastq.gz ./experiments/case10.7/data/SRR14463681_2.fastq.gz -o ./experiments/case10.7/output<br><br>conda activate abc<br>hisat2-build -p 16 ./experiments/case10.7/data/hg38.intg.virg.fa ./experiments/case10.7/data/hg38.intg.virg.fa.ht2<br>hisat2 -p 16 --dta -x ./experiments/case10.7/data/hg38.intg.virg.fa.ht2 -1 ./experiments/case10.7/data/SRR14463651_1.fastq.gz -2 ./experiments/case10.7/data/SRR14463651_2.fastq.gz -S ./experiments/case10.7/output/SRR14463651.sam<br>hisat2 -p 16 --dta -x ./experiments/case10.7/data/hg38.intg.virg.fa.ht2 -1 ./experiments/case10.7/data/SRR14463652_1.fastq.gz -2 ./experiments/case10.7/data/SRR14463652_2.fastq.gz -S ./experiments/case10.7/output/SRR14463652.sam<br>hisat2 -p 16 --dta -x ./experiments/case10.7/data/hg38.intg.virg.fa.ht2 -1 ./experiments/case10.7/data/SRR14463680_1.fastq.gz -2 ./experiments/case10.7/data/SRR14463680_2.fastq.gz -S ./experiments/case10.7/output/SRR14463680.sam

	hisat2 -p 16 --dta -x ./experiments/case10.7/data/hg38.intg.virg.fa.ht2 -<br>1 ./experiments/case10.7/data/SRR14463681_1.fastq.gz -<br>2 ./experiments/case10.7/data/SRR14463681_2.fastq.gz -<br>S ./experiments/case10.7/output/SRR14463681.sam<br><br><br>conda activate abc<br>gatk HaplotypeCaller -I ./experiments/case10.7/output/SRR14463651.sam -<br>I ./experiments/case10.7/output/SRR14463652.sam -<br>I ./experiments/case10.7/output/SRR14463680.sam -<br>I ./experiments/case10.7/output/SRR14463681.sam -<br>O ./experiments/case10.7/output/haplotypes.vcf<br><br>conda activate abc<br>gatk FilterRNAEditingSites -I ./experiments/case10.7/output/haplotypes.vcf -<br>O ./experiments/case10.7/output/filtered_haplotypes.vcf<br><br>conda activate abc<br>gatk FilterRNAEditingSites -I ./experiments/case10.7/output/haplotypes.vcf -<br>O ./experiments/case10.7/output/filtered_haplotypes.vcf

RNA-seq Analysis **Case 10.8: circular RNA analysis**

**User's Inputs**	**Data and Description**<br>**Data and Description**<br>SRR14463651_1.fastq.gz: paired end RNA sequencing data from human lymphoma cell, read 1 of lymphoma samples replicate 1<br>SRR14463651_2.fastq.gz: paired end RNA sequencing data from human lymphoma cell, read 2 of lymphoma samples replicate 1<br>SRR14463652_1.fastq.gz: paired end RNA sequencing data from human lymphoma cell, read 1 of lymphoma samples replicate 2<br>SRR14463652_2.fastq.gz: paired end RNA sequencing data from human lymphoma cell, read 2 of lymphoma samples replicate 2<br>SRR14463680_1.fastq.gz: paired end RNA sequencing data from human lymphoma cell, read 1 of control samples replicate 1<br>SRR14463680_2.fastq.gz: paired end RNA sequencing data from human lymphoma cell, read 2 of control samples replicate 1<br>SRR14463681_1.fastq.gz: paired end RNA sequencing data from human lymphoma cell, read 1 of control samples replicate 2<br>SRR14463681_2.fastq.gz: paired end RNA sequencing data from human lymphoma cell, read 2 of control samples replicate 2<br>hg38.fa: reference genome<br>gencode.v38.annotation.gtf: gene annotation<br>TruSeq3-PE.fa: trimming adapter<br>**Goal**<br>identify and quantify circular RNA in lymphoma and control samples
**Generated Plans by AutoBA**	"Step 1: Trimming and adapter removal", "Step 2: Mapping to the reference genome", "Step 3: Identifying circular RNA", "Step 4: Quantifying circular RNA abundance", "Step 5: Visualizing and interpreting results"
**Generated Codes by AutoBA**	conda install -y trimmomatic && trimmomatic PE -threads 4 -phred33 ./experiments/case10.8/data/SRR14463651_1.fastq.gz ./experiments/case10.8/data/SRR14463651_2.fastq.gz ./experiments/case10.8/data/SRR14463652_1.fastq.gz ./experiments/case10.8/data/SRR14463652_2.fastq.gz ./experiments/case10.8/data/SRR14463680_1.fastq.gz ./experiments/case10.8/data/SRR14463680_2.fastq.gz ./experiments/case10.8/data/SRR14463681_1.fastq.gz ./experiments/case10.8/data/SRR14463681_2.fastq.gz TMPDIR=./experiments/case10.8/output/tmp/ && rm -rf ./experiments/case10.8/output/tmp/<br><br>conda install -y star && STAR --runThreadN 4 --genomeDir ./experiments/case10.8/data/hg38.intg.virg.fa --readFilesIn ./experiments/case10.8/output/tmp/SRR14463651_1_paired.fq ./experiments/case10.8/output/tmp/SRR14463651_2_paired.fq ./experiments/case10.8/output/tmp/SRR14463652_1_paired.fq ./experiments/case10.8/output/tmp/SRR14463652_2_paired.fq ./experiments/case10.8/output/tmp/SRR14463680_1_paired.fq ./experiments/case10.8/output/tmp/SRR14463680_2_paired.fq ./experiments/case10.8/output/tmp/SRR14463681_1_paired.fq ./experiments/case10.8/output/tmp/SRR14463681_2_paired.fq --outSAMtype BAM SortedByCoordinate --outSAMunmapped Within --outSAMattributes NH HI NM MD AS --outSAMmode NoQS --outFileNamePrefix ./experiments/case10.8/output/SRR14463651_1.bam<br><br>conda install -y circRNA_identifier && circRNA_identifier -i ./experiments/case10.8/output/SRR14463651_1.bam -

	r ./experiments/case10.8/data/hg38.intg.virg.fa -<br>a ./experiments/case10.8/data/gencode.v38.annotation.gtf -<br>o ./experiments/case10.8/output/circular_RNA.txt<br><br>conda install -y featureCounts && featureCounts -T 4 -p -t exon -g gene_id -<br>a ./experiments/case10.8/data/gencode.v38.annotation.gtf -<br>o ./experiments/case10.8/output/counts.txt ./experiments/case10.8/output/S<br>RR14463651_1.bam ./experiments/case10.8/output/SRR14463651_2.bam .<br>/experiments/case10.8/output/SRR14463652_1.bam ./experiments/case10.<br>8/output/SRR14463652_2.bam ./experiments/case10.8/output/SRR144636<br>80_1.bam ./experiments/case10.8/output/SRR14463680_2.bam ./experime<br>nts/case10.8/output/SRR14463681_1.bam ./experiments/case10.8/output/S<br>RR14463681_2.bam<br><br>multiqc ./experiments/case10.8/output/counts.txt -<br>o ./experiments/case10.8/output/counts.pdf

Small RNA-seq Analysis **Case 11.1 and Case 11.2: miRNA analysis**

<table>
<tr><td>User’s Inputs</td><td>Data and Description
SRR10915820.fastq.gz: single end small RNA sequencing data from human cell line, control samples replicate 1
SRR10915821.fastq.gz: single end small RNA sequencing data from human cell line, control samples replicate 1
SRR10915822.fastq.gz: single end small RNA sequencing data from human cell line, control samples replicate 1
SRR10915823.fastq.gz: single end small RNA sequencing data from human cell line, control samples replicate 1
hsa.gff3: microRNA genomic coordinate from miRbase
TGGAATTCTCGGGTGCCAAGG: adaptor sequence
hg38.fa: the reference genome

Goal
quantify microRNA expression based on miRbase and predict novel microRNA</td></tr>
<tr><td>Generated Plans by AutoBA</td><td>"step 1: Data Preprocessing", "step 2: Read Mapping", "step 3: miRNA Quantification", "step 4: Novel miRNA Prediction", "step 5: Data Visualization"</td></tr>
<tr><td>Generated Codes by AutoBA</td><td>conda install -y cutadapt
cutadapt -a TGGAATTCTCGGGTGCCAAGG -o ./experiments/case11.1_2/output/SRR10915820.fastq.gz ./experiments/case11.1_2/data/SRR10915820.fastq.gz
cutadapt -a TGGAATTCTCGGGTGCCAAGG -o ./experiments/case11.1_2/output/SRR10915821.fastq.gz ./experiments/case11.1_2/data/SRR10915821.fastq.gz
cutadapt -a TGGAATTCTCGGGTGCCAAGG -o ./experiments/case11.1_2/output/SRR10915822.fastq.gz ./experiments/case11.1_2/data/SRR10915822.fastq.gz
cutadapt -a TGGAATTCTCGGGTGCCAAGG -o ./experiments/case11.1_2/output/SRR10915823.fastq.gz ./experiments/case11.1_2/data/SRR10915823.fastq.gz

STAR --runThreadN 8 --genomeDir ./experiments/case11.1_2/data/hg38.fa --readFilesIn ./experiments/case11.1_2/output/SRR10915820.fastq.gz ./experiments/case11.1_2/output/SRR10915821.fastq.gz ./experiments/case11.1_2/output/SRR10915822.fastq.gz ./experiments/case11.1_2/output/SRR10915823.fastq.gz --outSAMunmapped Within --outSAMtype BAM Unsorted --outSAMattributes NH HI NM MD --outFilterMultimapNmax 100000 --outFilterMismatchNmax 100000 --outFilterMatchNmin 100000 --outReadsUnmapped Fastx --outSAMstrandField intronMotif --outSAMmode Full --outSAMprimaryFlag AllBestScore --outSAMtype BAM SortedByCoordinate --limitBAMsortRAM 10000000000

salmon quant -i hsa.gff3 -l A -r SRR10915820.fastq.gz,SRR10915821.fastq.gz,SRR10915822.fastq.gz,SRR10915823.fastq.gz -o salmon_output

conda install -y miRDeep2
miRDeep2 -t ./experiments/case11.1_2/data/hsa.gff3 -r ./experiments/case11.1_2/output/SRR10915820.fastq.gz,./experiments/ca</td></tr>
</table>

	se11.1_2/output/SRR10915821.fastq.gz,./experiments/case11.1_2/output/SRR10915822.fastq.gz,./experiments/case11.1_2/output/SRR10915823.fastq.gz -o ./experiments/case11.1_2/output/novel_miRNA.fa<br><br>multiqc ./experiments/case11.1_2/output -o ./experiments/case11.1_2/output

CAGE-seq Analysis   Case 12.1: TSS analysis

**User's Inputs**	**Data and Description**<br>SRR11351697.fastq.gz: CAGE-seq data from human cell line, control samples replicate 1<br>SRR11351698.fastq.gz: CAGE-seq data from human cell line, control samples replicate 2<br>SRR11351700.fastq.gz: CAGE-seq data from human cell line, treatment samples replicate 1<br>SRR11351701.fastq.gz: CAGE-seq data from human cell line, treatment samples replicate 1<br>hg38.fa: the human reference genome<br>gencode.v37.annotation.gtf: gene annotation<br>TruSeq3-SE.fa: trimming adapter<br><br>**Goal**<br>Identify transcription start site (TSS) in the cell line based on CAGE-seq data
**Generated Plans by AutoBA**	'Step 1: Trimming and adapter removal', "Use software 'Trimmomatic' to remove low-quality reads and adapter sequences from the CAGE-seq data.", 'Step 2: Mapping to the reference genome', "Use software 'Bowtie2' to map the trimmed reads to the human reference genome (hg38).", 'Step 3: Filtering and sorting', "Use software 'Samtools' to filter and sort the mapped reads based on their quality and coordinates.", 'Step 4: Identifying TSS', "Use software 'HISAT2' to identify the transcription start sites (TSS) in the cell line based on the mapped reads.", 'Step 5: Annotation and visualization', "Use software 'R' to annotate and visualize the identified TSS with gene information from the gene annotation file (gencode.v37.annotation.gtf).
**Generated Codes by AutoBA**	conda install -y trimmomatic && trimmomatic PE -threads 4 ./experiments/case12.1/data/SRR11351697.fastq.gz ./experiments/case12.1/data/SRR11351698.fastq.gz ./experiments/case12.1/data/SRR11351700.fastq.gz ./experiments/case12.1/data/SRR11351701.fastq.gz ./experiments/case12.1/data/output/SRR11351697.trimmed.fastq.gz ./experiments/case12.1/data/output/SRR11351698.trimmed.fastq.gz ./experiments/case12.1/data/output/SRR11351700.trimmed.fastq.gz ./experiments/case12.1/data/output/SRR11351701.trimmed.fastq.gz<br><br>conda install -y bowtie2 && bowtie2-build ./experiments/case12.1/data/hg38.fa ./experiments/case12.1/data/hg38 && bowtie2 -x ./experiments/case12.1/data/hg38 -1 ./experiments/case12.1/data/SRR11351697.trimmed.fastq.gz -2 ./experiments/case12.1/data/SRR11351698.trimmed.fastq.gz -3 ./experiments/case12.1/data/SRR11351700.trimmed.fastq.gz -4 ./experiments/case12.1/data/SRR11351701.trimmed.fastq.gz -S ./experiments/case12.1/data/output/SRR11351697.trimmed.sam ./experiments/case12.1/data/output/SRR11351698.trimmed.sam ./experiments/case12.1/data/output/SRR11351700.trimmed.sam ./experiments/case12.1/data/output/SRR11351701.trimmed.sam<br><br>conda install -y samtools && samtools view -b -h ./experiments/case12.1/data/output/SRR11351697.trimmed.sam ./experi

	ments/case12.1/data/output/SRR11351698.trimmed.sam ./experiments/case12.1/data/output/SRR11351700.trimmed.sam ./experiments/case12.1/data/output/SRR11351701.trimmed.sam -o ./experiments/case12.1/data/output/SRR11351697.filtered.bam ./experiments/case12.1/data/output/SRR11351698.filtered.bam ./experiments/case12.1/data/output/SRR11351700.filtered.bam ./experiments/case12.1/data/output/SRR11351701.filtered.bam && samtools sort ./experiments/case12.1/data/output/SRR11351697.filtered.bam ./experiments/case12.1/data/output/SRR11351698.filtered.bam ./experiments/case12.1/data/output/SRR11351700.filtered.bam ./experiments/case12.1/data/output/SRR11351701.filtered.bam -o ./experiments/case12.1/data/output/SRR11351697.sorted.bam ./experiments/case12.1/data/output/SRR11351698.sorted.bam ./experiments/case12.1/data/output/SRR11351700.sorted.bam ./experiments/case12.1/data/output/SRR11351701.sorted.bam

3'end-seq Analysis   Case 13.1: APA analysis based on 3'end-seq data

**User's Inputs**	**Data and Description**<br>SRR17422754.fastq.gz: Lexongen QuantSeq 3 prime-end RNA sequencing data from human cell line THLE2, healthy liver cell line replicate 1<br>SRR17422755.fastq.gz: Lexongen QuantSeq 3 prime-end RNA sequencing data from human cell line THLE2, healthy liver cell line replicate 2<br>SRR17422756.fastq.gz: Lexongen QuantSeq 3 prime-end RNA sequencing data from human cell line SNU398, liver cancer cell line replicate 1<br>SRR17422757.fastq.gz: Lexongen QuantSeq 3 prime-end RNA sequencing data from human cell line SNU398, liver cancer cell line replicate 2<br>hg38.fa: the human reference genome<br>gencode.v37.annotation.gtf: gene annotation<br>TruSeq3-SE.fa: trimming adapter<br><br>**Goal**<br>Identify polyadenylation sites and quantify alternative polyadenylation based on 3'-end sequencing data
**Generated Plans by AutoBA**	"Step 1: Trimming and adapter removal", "Use the Trimmomatic software to remove low-quality base pairs and adapter sequences from the raw sequencing data. The input files are the fastq files and the output files are the trimmed and adapter-removed fastq files.", "Step 2: Mapping to the reference genome", "Use the STAR software to map the trimmed and adapter-removed fastq files to the human reference genome. The output files are the mapped reads in SAM format.", "Step 3: Converting SAM to BAM", "Use the samtools software to convert the SAM files to BAM format. The output files are the mapped reads in BAM format.", "Step 4: Sorting and indexing the BAM file", "Use the samtools software to sort and index the BAM file. The output files are the sorted and indexed BAM file.", "Step 5: Identifying polyadenylation sites", "Use the RSEM software to identify polyadenylation sites in the mapped reads. The output files are the identified polyadenylation sites in GTF format.", "Step 6: Quantifying alternative polyadenylation", "Use the RSEM software to quantify alternative polyadenylation in the mapped reads. The output files are the quantified alternative polyadenylation in GTF format.", "Step 7: Visualizing the results", "Use the UCSC genome browser to visualize the identified polyadenylation sites and the quantified alternative polyadenylation."
**Generated Codes by AutoBA**	conda activate abc<br>trimmomatic PE -threads<br>4 ./experiments/case13.1/data/SRR17422754.fastq.gz ./experiments/case13.1/data/SRR17422755.fastq.gz ./experiments/case13.1/data/SRR17422756.fastq.gz ./experiments/case13.1/data/SRR17422757.fastq.gz ./experiments/case13.1/data/TruSeq3-<br>SE.fa ./experiments/case13.1/output/SRR17422754_1.fastq.gz ./experiments/case13.1/output/SRR17422754_2.fastq.gz ./experiments/case13.1/output/SRR17422755_1.fastq.gz ./experiments/case13.1/output/SRR17422755_2.fastq.gz ./experiments/case13.1/output/SRR17422756_1.fastq.gz ./experiments/case13.1/output/SRR17422756_2.fastq.gz ./experiments/case13.1/output/SRR17422757_1.fastq.gz ./experiments/case13.1/output/SRR17422757_2.fastq.gz<br><br>conda activate abc<br>trimmomatic PE -threads<br>4 ./experiments/case13.1/data/SRR17422754.fastq.gz ./experiments/case1

	3.1/data/SRR17422755.fastq.gz ./experiments/case13.1/data/SRR17422756.fastq.gz ./experiments/case13.1/data/SRR17422757.fastq.gz ./experiments/case13.1/data/TruSeq3-SE.fa ./experiments/case13.1/output/SRR17422754_1.fastq.gz ./experiments/case13.1/output/SRR17422754_2.fastq.gz ./experiments/case13.1/output/SRR17422755_1.fastq.gz ./experiments/case13.1/output/SRR17422755_2.fastq.gz ./experiments/case13.1/output/SRR17422756_1.fastq.gz ./experiments/case13.1/output/SRR17422756_2.fastq.gz ./experiments/case13.1/output/SRR17422757_1.fastq.gz ./experiments/case13.1/output/SRR17422757_2.fastq.gz<br><br>conda activate abc<br>STAR --runThreadN 4 --genomeDir ./experiments/case13.1/data/hg38.fa --readFilesIn ./experiments/case13.1/output/SRR17422754_1.fastq.gz ./experiments/case13.1/output/SRR17422754_2.fastq.gz ./experiments/case13.1/output/SRR17422755_1.fastq.gz ./experiments/case13.1/output/SRR17422755_2.fastq.gz ./experiments/case13.1/output/SRR17422756_1.fastq.gz ./experiments/case13.1/output/SRR17422756_2.fastq.gz ./experiments/case13.1/output/SRR17422757_1.fastq.gz ./experiments/case13.1/output/SRR17422757_2.fastq.gz --outSAMtype BAM SortedByCoordinate --outSAMunmapped Within --outSAMattributes NH HI NM MD AS --outFileNamePrefix ./experiments/case13.1/output/SRR17422754_1.fastq.gz.sorted.bam<br><br><br>conda activate abc<br>STAR --runThreadN 4 --genomeDir ./experiments/case13.1/data/hg38.fa --readFilesIn ./experiments/case13.1/output/SRR17422754_1.fastq.gz ./experiments/case13.1/output/SRR17422754_2.fastq.gz ./experiments/case13.1/output/SRR17422755_1.fastq.gz ./experiments/case13.1/output/SRR17422755_2.fastq.gz ./experiments/case13.1/output/SRR17422756_1.fastq.gz ./experiments/case13.1/output/SRR17422756_2.fastq.gz ./experiments/case13.1/output/SRR17422757_1.fastq.gz ./experiments/case13.1/output/SRR17422757_2.fastq.gz --outSAMtype BAM SortedByCoordinate --outSAMunmapped Within --outSAMattributes NH HI NM MD AS --outFileNamePrefix ./experiments/case13.1/output/SRR17422754_1.fastq.gz.sorted.bam<br><br>conda activate abc<br>samtools view -bS ./experiments/case13.1/output/SRR17422754_1.fastq.gz.sorted.bam > ./experiments/case13.1/output/SRR17422754_1.fastq.gz.sorted.bam.bam<br>samtools view -bS ./experiments/case13.1/output/SRR17422754_2.fastq.gz.sorted.bam > ./experiments/case13.1/output/SRR17422754_2.fastq.gz.sorted.bam.bam<br>samtools view -bS ./experiments/case13.1/output/SRR17422755_1.fastq.gz.sorted.bam > ./experiments/case13.1/output/SRR17422755_1.fastq.gz.sorted.bam.bam<br>samtools view -bS ./experiments/case13.1/output/SRR17422755_2.fastq.gz.sorted.bam > ./experiments/case13.1/output/SRR17422755_2.fastq.gz.sorted.bam.bam

	samtools view -<br>bS ./experiments/case13.1/output/SRR17422756_1.fastq.gz.sorted.bam > ./<br>experiments/case13.1/output/SRR17422756_1.fastq.gz.sorted.bam.bam<br>samtools view -<br>bS ./experiments/case13.1/output/SRR17422756_2.fastq.gz.sorted.bam > ./<br>experiments/case13.1/output/SRR17422756_2.fastq.gz.sorted.bam.bam<br>samtools view -<br>bS ./experiments/case13.1/output/SRR17422757_1.fastq.gz.sorted.bam > ./<br>experiments/case13.1/output/SRR17422757_1.fastq.gz.sorted.bam.bam<br>samtools view -<br>bS ./experiments/case13.1/output/SRR17422757_2.fastq.gz.sorted.bam > ./<br>experiments/case13.1/output/SRR17422757_2.fastq.gz.sorted.bam.bam

ONT sequencing Analysis Case 14.1: isoform expression

**User's Inputs**	**Data and Description**<br>RHL5569-T1_SAECK_shControl_replicate_1.ont.fastq.gz: ONT sequencing data from human cell line, control samples replicate 1<br>RHL5570-T1_SAECK_shControl_replicate_2.ont.fastq.gz: ONT sequencing data from human cell line, control samples replicate 2<br>RHL5571-T1_SAECK_shPARP4-1_replicate_1.ont.fastq.gz: ONT sequencing data from human cell line, treatment samples replicate 1<br>RHL5572-T1_SAECK_shPARP4-1_replicate_2.ont.fastq.gz: ONT sequencing data from human cell line, treatment samples replicate 2<br>hg38.fa: the human reference genome<br>gencode.v37.annotation.gtf: gene annotation<br><br>**Goal**<br>Quantify isoform expression in control and treatment samples
**Generated Plans by AutoBA**	'step 1: preprocessing of the data', 'step 2: align the data to the reference genome using a software that is not STAR, java, perl, or annovar', 'step 3: quantify isoform expression in control and treatment samples using flair'
**Generated Codes by AutoBA**	flair --preprocess --fastq --input ./experiments/case14.1/data/RHL5569-T1_SAECK_shControl_replicate_1.ont.fastq.gz,./experiments/case14.1/data/RHL5570-T1_SAECK_shControl_replicate_2.ont.fastq.gz,./experiments/case14.1/data/RHL5571-T1_SAECK_shPARP4-1_replicate_1.ont.fastq.gz,./experiments/case14.1/data/RHL5572-T1_SAECK_shPARP4-1_replicate_2.ont.fastq.gz --output ./experiments/case14.1/data/output<br><br>flair --align --reference ./experiments/case14.1/data/hg38.fa --input ./experiments/case14.1/data/output/preprocessed_data.fastq --output ./experiments/case14.1/data/output/aligned_data.bam<br><br>flair --quantify --expression --input ./experiments/case14.1/data/output/aligned_data.bam --output ./experiments/case14.1/data/output/expression_data.txt

PacBio iso-seq Analysis **Case 15.1: isoform analysis**

<table>
<tr><td>User’s Inputs</td><td>Data and Description
SRR19552218.fastq.gz: pacbio sequecning data from human cell line, cell line HCT-116
SRR19785215.fastq.gz: pacbio sequecning data from human cell line, cell line HepG2
hg38.fa: the human reference genome
gencode.v37.annotation.gtf: gene annotation

Goal
identify novel isoform and quantify isoform expression in two cell lines</td></tr>
<tr><td>Generated Plans by AutoBA</td><td>"Step 1: Preprocessing of the sequencing data", "Use software such as fastp or trimmomatic to remove low-quality reads and adapters from the sequencing data.", "Step 2: Mapping of the sequencing data to the reference genome", "Use software such as minimap2 or bowtie to map the preprocessed sequencing data to the reference genome.", "Step 3: Identification of novel isoforms", "Use software such as HISAT2 or STAR to align the sequencing data to the reference genome and identify novel isoforms.", "Step 4: Quantification of isoform expression", "Use software such as featureCounts or RSEM to quantify the expression of identified isoforms in the two cell lines.", "Step 5: Visualization and interpretation of results", "Use software such as UCSC Genome Browser or Integrative Genomics Viewer to visualize and interpret the results, including the identification of differentially expressed isoforms between the two cell lines."</td></tr>
<tr><td>Generated Codes by AutoBA</td><td>fastp --in1 ./experiments/case15.1/data/SRR19552218.fastq.gz --in2 ./experiments/case15.1/data/SRR19785215.fastq.gz --out1 ./experiments/case15.1/data/SRR19552218_clean.fastq.gz --out2 ./experiments/case15.1/data/SRR19785215_clean.fastq.gz

fastp --in1 ./experiments/case15.1/data/SRR19552218.fastq.gz --in2 ./experiments/case15.1/data/SRR19785215.fastq.gz --out1 ./experiments/case15.1/data/SRR19552218_clean.fastq.gz --out2 ./experiments/case15.1/data/SRR19785215_clean.fastq.gz

minimap2 -ax map-ont ./experiments/case15.1/data/SRR19552218_clean.fastq.gz ./experiments/case15.1/data/SRR19785215_clean.fastq.gz ./experiments/case15.1/data/hg38.fa > ./experiments/case15.1/output/SRR19552218_SRR19785215.sam

minimap2 -ax map-ont ./experiments/case15.1/data/SRR19552218_clean.fastq.gz ./experiments/case15.1/data/SRR19785215_clean.fastq.gz ./experiments/case15.1/data/hg38.fa > ./experiments/case15.1/output/SRR19552218_SRR19785215.sam

hisat2-build -p 8 ./experiments/case15.1/data/hg38.fa ./experiments/case15.1/output/hg38_hisat2_index
hisat2 -p 8 --dta -x ./experiments/case15.1/output/hg38_hisat2_index -1 ./experiments/case15.1/data/SRR19552218_clean.fastq.gz -2 ./experiments/case15.1/data/SRR19785215_clean.fastq.gz -</td></tr>
</table>

	S ./experiments/case15.1/output/SRR19552218_SRR19785215_hisat2.sa<br>m<br><br>hisat2-build -p<br>8 ./experiments/case15.1/data/hg38.fa ./experiments/case15.1/output/hg38<br>_hisat2_index<br>hisat2 -p 8 --dta -x ./experiments/case15.1/output/hg38_hisat2_index -<br>1 ./experiments/case15.1/data/SRR19552218_clean.fastq.gz -<br>2 ./experiments/case15.1/data/SRR19785215_clean.fastq.gz -<br>S ./experiments/case15.1/output/SRR19552218_SRR19785215_hisat2.sa<br>m<br><br>featureCounts -a ./experiments/case15.1/data/gencode.v37.annotation.gtf -<br>o ./experiments/case15.1/output/featureCounts.txt ./experiments/case15.1/o<br>utput/SRR19552218_SRR19785215_hisat2.sam<br><br>conda activate abc<br>conda install -y featureCounts<br>featureCounts -a ./experiments/case15.1/data/gencode.v37.annotation.gtf -<br>o ./experiments/case15.1/output/featureCounts.txt ./experiments/case15.1/o<br>utput/SRR19552218_SRR19785215_hisat2.sam<br><br>conda activate abc<br>conda install -y featureCounts<br>featureCounts -a ./experiments/case15.1/data/gencode.v37.annotation.gtf -<br>o ./experiments/case15.1/output/featureCounts.txt ./experiments/case15.1/o<br>utput/SRR19552218_SRR19785215_hisat2.sam.<br><br><br>conda activate abc<br>conda install -y ucsc-genome-browser<br>ucsc-genome-browser -i ./experiments/case15.1/output/featureCounts.txt -<br>o ./experiments/case15.1/output/UCSC_Genome_Browser.png

CLIP-seq Analysis **Case 16.1: CLIP-seq data analysis**

**User's Inputs**	**Data and Description**<br>ENCLB742AYH.fastq.gz: single end reads of eCLIP data from HepG2 cell line, SRSF1 targeted sample replicate 1<br>ENCLB770EDJ.fastq.gz: single end reads of eCLIP data from HepG2 cell line, SRSF1 targeted sample replicate 2<br>hg38.fa: the human reference genome<br>gencode.v37.annotation.gtf: gene annotation<br><br>**Goal**<br>identify SRSF1 protein-RNA crosslink sites based on CLIP sequencing data
**Generated Plans by AutoBA**	'step 1: Data Preprocessing', 'step 2: Mapping', 'step 3: Peak Calling', 'step 4: Annotation', 'step 5: Visualization'
**Generated Codes by AutoBA**	conda install -y fastqc && fastqc -<br>o ./experiments/case16.1/data/output ./experiments/case16.1/data/ENCLB742AYH.fastq.gz ./experiments/case16.1/data/ENCLB770EDJ.fastq.gz ./experiments/case16.1/data/ENCLB053AXU.fastq.gz ./experiments/case16.1/data/ENCLB002KIQ.fastq.gz<br><br>conda install -y hisat2 && hisat2-build -p<br>16 ./experiments/case16.1/data/hg38.intg.virg.fa ./experiments/case16.1/data/hg38.intg.virg.fa.hisat2_index && hisat2 -p 16 --dta -<br>x ./experiments/case16.1/data/hg38.intg.virg.fa.hisat2_index -<br>1 ./experiments/case16.1/data/ENCLB742AYH.fastq.gz -<br>2 ./experiments/case16.1/data/ENCLB770EDJ.fastq.gz -<br>3 ./experiments/case16.1/data/ENCLB053AXU.fastq.gz -<br>4 ./experiments/case16.1/data/ENCLB002KIQ.fastq.gz -<br>S ./experiments/case16.1/data/output/alignment.sam<br><br>hisat2 -p 16 --dta -<br>x ./experiments/case16.1/data/hg38.intg.virg.fa.hisat2_index -<br>1 ./experiments/case16.1/data/ENCLB742AYH.fastq.gz -<br>2 ./experiments/case16.1/data/ENCLB770EDJ.fastq.gz -<br>3 ./experiments/case16.1/data/ENCLB053AXU.fastq.gz -<br>4 ./experiments/case16.1/data/ENCLB002KIQ.fastq.gz -<br>S ./experiments/case16.1/data/output/alignment.sam<br><br>conda install -y bedtools && bedtools intersect -<br>a ./experiments/case16.1/data/output/alignment.bed -<br>b ./experiments/case16.1/data/gencode.v38.annotation.gtf -c -s -split -f 0.5 -<br>r 0.5 > ./experiments/case16.1/data/output/annotated_peaks.bed<br><br>conda install -y igb<br>igb -g ./experiments/case16.1/data/hg38.intg.virg.fa -<br>b ./experiments/case16.1/data/output/annotated_peaks.bed

RIP-Seq Analysis **Case 16.2: Identify enriched genes bounded by RPB**

**User's Inputs**	**Data and Description**<br>rip.rep1.fastq.gz: single end reads of RIP-seq data from HepG2 cell line, pulldown samples of SRSF1 replicate 1<br>rip.rep2.fastq.gz: single end reads of RIP-seq data from HepG2 cell line, pulldown samples of SRSF1 replicate 2<br>controll.rep1.fastq.gz: single end reads of RIP-seq daata from HepG2 cell line, control sample replicate 1<br>controll.rep2.fastq.gz: single end reads of RIP-seq daata from HepG2 cell line, control sample replicate 2<br>hg38.fa: the human reference genome<br>gencode.v37.annotation.gtf: gene annotation<br><br>Goal<br>identify genes enriched in pulldown samples over the control
**Generated Plans by AutoBA**	"step 1: prepare the data", "step 2: trim the adapters", "step 3: map the reads to the reference genome", "step 4: count the reads per gene", "step 5: perform a statistical test to identify genes enriched in pulldown samples over the control", "step 6: generate a list of differentially expressed genes"
**Generated Codes by AutoBA**	conda create -n abc -y && conda activate abc && conda config --add channels conda-forge && conda config --add channels bioconda && conda install -y STAR && conda install -y java && conda install -y perl && conda install -y annovar<br><br>conda activate abc && trimmomatic PE -threads 4 ./experiments/case16.2/data/rip.rep1.fastq.gz ./experiments/case16.2/data/rip.rep1.fastq.gz ./experiments/case16.2/data/rip.rep1.fastq.gz ./experiments/case16.2/data/rip.rep1.fastq.gz ILLUMINACLIP:/experiments/case16.2/data/TruSeq3-SE.fa:2:30:10 LEADING:3 TRAILING:3 SLIDINGWINDOW:4:15 MINLEN:36<br><br>conda activate abc && STAR --runMode genomeGenerate --genomeDir /experiments/case16.2/data/hg38.intg.virg.fa --genomeFastaFiles /experiments/case16.2/data/hg38.intg.virg.fa --sjdbOverhang 100 --runThreadN 4<br><br>conda activate abc && featureCounts -p -T 4 -a /experiments/case16.2/data/gencode.v38.annotation.gtf -o /experiments/case16.2/output/featureCounts.txt /experiments/case16.2/data/rip.rep1.fastq.gz /experiments/case16.2/data/rip.rep2.fastq.gz /experiments/case16.2/data/controll.rep1.fastq.gz /experiments/case16.2/data/control2.rep2.fastq.gz<br><br>Rscript -e "library(edgeR); data <- read.table('/experiments/case16.2/output/featureCounts.txt'); design <- model.matrix(~sample); fit <- edgeR(data, design); degs <- topTags(fit, n=100); write.table(degs, '/experiments/case16.2/output/degs.txt', quote=FALSE, sep=' ')"<br><br>Rscript -e "library(edgeR); data <- read.table('/experiments/case16.2/output/featureCounts.txt'); design <-

	model.matrix(~sample); fit <- edgeR(data, design); degs <- topTags(fit, n=100); write.table(degs, '/experiments/case16.2/output/degs.txt', quote=FALSE, sep=' ')"

Ribo-Seq Analysis Case 17.1: Identify translated ORFs

<table>
<tr><td>User's Inputs</td><td>Data and Description
SRR12354645_1.fastq.gz: read 1 of paired-end ribo-seq data, read 1 of control samples replicate 1
SRR12354645_2.fastq.gz: read 2 of paired-end ribo-seq data, read 2 of control samples replicate 1
SRR12354646_1.fastq.gz: read 1 of paired-end ribo-seq data, read 1 of control samples replicate 2
SRR12354646_2.fastq.gz: read 2 of paired-end ribo-seq data, read 2 of control samples replicate 2
hg38.fa: the human reference genome
gencode.v37.annotation.gtf: gene annotation
TruSeq3-PE.fa: trimming adapter

Goal
Identify translated ORFs based on ribo-seq data</td></tr>
<tr><td>Generated Plans by AutoBA</td><td>"Step 1: Preprocessing of the ribo-seq data", "Step 2: Mapping of the ribo-seq data to the reference genome", "Step 3: Identification of the translated ORFs", "Step 4: Filtering of the translated ORFs based on quality and annotation", "Step 5: Visualization of the results"</td></tr>
<tr><td>Generated Codes by AutoBA</td><td>conda activate abc
conda install -y trimmomatic
trimmomatic PE -threads
4 ./experiments/case17.1/data/SRR12354645_1.fastq.gz ./experiments/case17.1/data/SRR12354645_2.fastq.gz ./experiments/case17.1/output/SRR12354645_1.fastq.gz ./experiments/case17.1/output/SRR12354645_1.fastq.gz ./experiments/case17.1/output/SRR12354645_2.fastq.gz ./experiments/case17.1/output/SRR12354645_2.fastq.gz
ILLUMINACLIP:/experiments/case17.1/data/TruSeq3-PE.fa:2:30:10
LEADING:3 TRAILING:3 SLIDINGWINDOW:4:15 MINLEN:36

conda activate abc
conda install -y hisat2
hisat2-build -p
4 ./experiments/case17.1/data/hg38.fa ./experiments/case17.1/output/hg38.fa
hisat2 -x ./experiments/case17.1/output/hg38.fa -
1 ./experiments/case17.1/output/SRR12354645_1.fastq.gz -
2 ./experiments/case17.1/output/SRR12354645_2.fastq.gz -
S ./experiments/case17.1/output/SRR12354645.sam

conda activate abc
conda install -y salmon
salmon quant -t ./experiments/case17.1/data/hg38.fa -l A -
1 ./experiments/case17.1/output/SRR12354645_1.fastq.gz -
2 ./experiments/case17.1/output/SRR12354645_2.fastq.gz -
o ./experiments/case17.1/output/salmon.quant

conda activate abc
conda install -y salmon
salmon quant -t ./experiments/case17.1/data/hg38.fa -l A -
1 ./experiments/case17.1/output/SRR12354645_1.fastq.gz -</td></tr>
</table>

	2 ./experiments/case17.1/output/SRR12354645_2.fastq.gz -<br>o ./experiments/case17.1/output/salmon.quant.<br><br><br>conda activate abc<br>salmon quant -t ./experiments/case17.1/data/hg38.fa -l A -<br>1 ./experiments/case17.1/output/SRR12354645_1.fastq.gz -<br>2 ./experiments/case17.1/output/SRR12354645_2.fastq.gz -<br>o ./experiments/case17.1/output/salmon.quant.

scRNA-Seq Analysis Case 18.1: Cluster cell into groups based on raw fastq files

**User’s Inputs**	**Data and Description**<br>/pbmc_1k_v3_fastqs.tar: the packed fastq files from 10x single cell sequencing<br>refdata-gex-GRCh38-2020-A: directory contains transcriptome reference<br><br>**Goal**<br>cluster cells into different groups based on 10x single cell sequencing data
**Generated Plans by AutoBA**	"step 1: preprocess the fastq files using the software 'cutadapt' to remove the low-quality reads and adapter sequences.", "step 2: align the preprocessed reads to the transcriptome reference using the software 'STAR' to generate the alignments.", "step 3: count the number of reads that map to each gene in the transcriptome reference using the software 'featureCounts' to generate the gene expression matrix.", "step 4: cluster the cells into different groups based on the gene expression matrix using the software 'Seurat' to identify the cell types and states.", "step 5: save the results to a file using the software 'R' to create a data frame and then export it to a csv file."
**Generated Codes by AutoBA**	cutadapt -a ADAPTER_SEQUENCE -o output.fastq input.fastq<br><br>STAR --runThreadN 4 --genomeDir /experiments/case18.1/data/refdata-gex-GRCh38-2020-A --readFilesIn ./experiments/case18.1/data/pbmc_1k_v3_fastqs.tar --readFilesCommand zcat --outSAMtype BAM Unsorted --outSAMunmapped Within --outSAMattributes NH HI NM MD AS --outSAMfilter Matched --outSAMstrandField intronMotif<br><br>featureCounts -a ./experiments/case18.1/data/refdata-gex-GRCh38-2020-A -o ./experiments/case18.1/output/featureCounts_output.txt ./experiments/case18.1/data/pbmc_1k_v3_fastqs.tar<br><br>seurat -i ./experiments/case18.1/output/featureCounts_output.txt -o ./experiments/case18.1/output/seurat_output.txt<br><br>Rscript -e "library(Seurat); seurat_obj <- Read10X(data.dir = './experiments/case18.1/data/pbmc_1k_v3_fastqs.tar'); seurat_obj <- SCTransform(seurat_obj); seurat_obj <- RunPCA(seurat_obj, features = rownames(seurat_obj)); seurat_obj <- FindNeighbors(seurat_obj); seurat_obj <- FindClusters(seurat_obj); saveRDS(seurat_obj, file = './experiments/case18.1/output/seurat_output.rds')"

scRNA-Seq Analysis Case 18.2: Clustering and Find the differentially expressed genes

**User's Inputs**	**Data and Description**<br>./examples/case2.1/data/filtered_gene_bc_matrices/hg19: path to 10x mtx data<br><br>**Goal**<br>Use scanpy to find the differentially expressed genes
**Generated Plans by AutoBA**	"Step 1: Load the 10x mtx data using scanpy.read_10x_mtx() function.", "Step 2: Normalize the data using scanpy.pp.normalize_total() function.", "Step 3: Filter out lowly expressed genes using scanpy.pp.filter_genes() function.", "Step 4: Perform dimensionality reduction using scanpy.pp.pca() function.", "Step 5: Cluster the cells using scanpy.pp.neighbors() and scanpy.tl.leiden() functions.", "Step 6: Find the differentially expressed genes between the clusters using scanpy.tl.rank_genes_groups() function.", "Step 7: Visualize the results using scanpy.pl.umap() and scanpy.pl.rank_genes_groups() functions."
**Generated Codes by AutoBA**	scanpy.read_10x_mtx('./experiments/case18.2/data/mtx/')<br><br>scanpy.pp.normalize_total(adata, target_sum=1, exclude_highly_expressed=True)<br><br>scanpy.pp.filter_genes(adata, min_counts=10, max_counts=500)<br><br>scanpy.pp.pca(adata, n_comps=50, svd_solver='arpack')<br><br>scanpy.pp.neighbors(adata, n_neighbors=10, n_pcs=50).<br>scanpy.tl.leiden(adata, resolution=1.0).<br><br><br>scanpy.tl.rank_genes_groups(adata, groups='leiden', n_genes=20, method='t-test')<br><br>scanpy.pl.umap(adata, color='leiden')<br>scanpy.pl.rank_genes_groups(adata, groups='leiden', n_genes=20, method='t-test')

scRNA-Seq Analysis Case 18.3: Identify Marker Genes

**User's Inputs**	**Data and Description**<br>./examples/case2.1/data/filtered_gene_bc_matrices/hg19: path to 10x mtx data<br><br>**Goal**<br>Use scanpy to identify top5 marker genes.
**Generated Plans by AutoBA**	"Step 1: Load the 10x mtx data using scanpy.read_10x_mtx() function. The function takes the path to the mtx data as input and returns a scanpy.AnnData object.", "Step 2: Preprocess the data by filtering out lowly expressed genes and selecting a subset of highly variable genes using scanpy.pp.filter_genes() and scanpy.pp.highly_variable_genes() functions.", "Step 3: Normalize the data using scanpy.pp.normalize_total() function to normalize the data by the total count per cell.", "Step 4: Scale the data using scanpy.pp.scale() function to scale the data to a common range.", "Step 5: Identify the top 5 marker genes for each cell type using scanpy.tl.rank_genes_groups() function. The function takes the AnnData object and a list of group labels as input and returns a pandas.DataFrame object containing the ranked genes for each group.", "Step 6: Visualize the results using scanpy.pl.violin() function to plot the distribution of the top 5 marker genes for each cell type."
**Generated Codes by AutoBA**	conda activate abc<br>scanpy.read_10x_mtx('./experiments/case18.3/data/mtx/')<br><br>conda activate abc<br>import scanpy as sc<br>data = sc.read_10x_mtx('./experiments/case18.3/data/mtx/')<br>filtered_data = sc.pp.filter_genes(data, min_cells=10)<br>highly_variable_genes = sc.pp.highly_variable_genes(filtered_data, n_top_genes=1000)<br>sc.pp.normalize_total(highly_variable_genes)<br>sc.pp.scale(highly_variable_genes)<br>marker_genes = sc.tl.rank_genes_groups(highly_variable_genes, groupby='cell_type')<br>sc.pl.violin(marker_genes, ['top5_gene1', 'top5_gene2', 'top5_gene3', 'top5_gene4', 'top5_gene5'])

scRNA-Seq Analysis Case 18.4: Clustering and Visualization

**User's Inputs**	**Data and Description**<br>./examples/case2.1/data/filtered_gene_bc_matrices/hg19: path to 10x mtx data<br><br>**Goal**<br>Use scanpy to perform clustering and visualize the expression level of gene PPBP in the UMAP.
**Generated Plans by AutoBA**	"Step 1: Load the 10x mtx data using scanpy.read_10x_mtx() and convert it into an AnnData object.", "Step 2: Perform clustering using the scanpy.tl.leiden() function.", "Step 3: Visualize the expression level of gene PPBP using the scanpy.pl.umap() function.", "Step 4: Save the visualization as a PDF file using the scanpy.pl.write_pdf() function."
**Generated Codes by AutoBA**	scanpy.read_10x_mtx('./experiments/case18.4/data/mtx/', './experiments/case18.4/output/')<br><br>scanpy.tl.leiden(adata, resolution=0.5, random_state=42)<br><br>scanpy.pl.umap(adata, color='PPBP', save='./experiments/case18.4/output/PPBP_umap.pdf')<br><br>scanpy.pl.write_pdf(adata, save='./experiments/case18.4/output/PPBP_umap.pdf')

Spatial Transcriptomics **Case 19.1: Neighborhood enrichment analysis**

**User's Inputs**	**Data and Description**<br>slice1.h5ad: spatial transcriptomics data for slice 1 in AnnData format<br><br>**Goal**<br>Use squidpy for neighborhood enrichment analysis
**Generated Plans by AutoBA**	"Step 1: Install and set up Tangram", "Step 2: Convert spatial transcriptomics data to a format compatible with Tangram", "Step 3: Load single-cell data into Tangram", "Step 4: Map single-cell data to spatial transcriptomics data using Tangram", "Step 5: Visualize and interpret the results"
**Generated Codes by AutoBA**	conda install -y -c bioconda -c conda-forge tangram<br>source activate abc<br>tangram --help<br><br>tangram convert -i ./experiments/case19.1/data/slice1.h5ad -<br>o ./experiments/case19.1/output/slice1.h5ad -f anndata<br><br>tangram load -i ./experiments/case19.1/data/slice1.h5ad -<br>o ./experiments/case19.1/output/slice1.h5ad<br><br>tangram map -i ./experiments/case19.1/output/slice1.h5ad -<br>o ./experiments/case19.1/output/slice1.mapped.h5ad<br><br>tangram visualize -i ./experiments/case19.1/output/slice1.mapped.h5ad -<br>o ./experiments/case19.1/output/slice1.mapped.pdf --color-by gene --<br>highlight-genes gene1,gene2

Spatial Transcriptomics **Case 19.2: Single-cell mapping**

**User's Inputs**	**Data and Description**<br>slice1.h5ad: spatial transcriptomics data for slice 1 in AnnData format<br>sc.h5ad: single cell data in the same tissue for slice 1 in AnnData format<br><br>**Goal**<br>Use Tangram to map single-cell data to spatial transcriptomics data.
**Generated Plans by AutoBA**	"step 1: Load the spatial transcriptomics data from './experiments/case19.2/data/slice1.h5ad' using the AnnData format.", "step 2: Load the single cell data from './experiments/case19.2/data/sc.h5ad' using the AnnData format.", "step 3: Preprocess the data by filtering genes and cells based on quality control metrics.", "step 4: Map the single cell data to the spatial transcriptomics data using Tangram.", "step 5: Visualize the mapped data to identify patterns and correlations between the single cell and spatial transcriptomics data.", "step 6: Use the mapped data to make predictions about the spatial distribution of gene expression in the tissue.", "step 7: Evaluate the performance of the Tangram mapping using metrics such as accuracy and precision.", "step 8: Refine the Tangram mapping by adjusting parameters and exploring different preprocessing techniques.", "step 9: Use the refined Tangram mapping to make predictions about the spatial distribution of gene expression in the tissue.", "step 10: Evaluate the performance of the refined Tangram mapping using metrics such as accuracy and precision."
**Generated Codes by AutoBA**	conda install -y anndata<br>import anndata<br>data = anndata.read_h5ad('./experiments/case19.2/data/slice1.h5ad')<br><br>conda install -y anndata<br>import anndata<br>data = anndata.read_h5ad('./experiments/case19.2/data/sc.h5ad')<br><br>conda install -y anndata<br>import anndata<br>data = anndata.read_h5ad('./experiments/case19.2/data/slice1.h5ad')<br>data = anndata.read_h5ad('./experiments/case19.2/data/sc.h5ad')<br>data.filter_genes(min_cells=3)<br>data.filter_cells(min_genes=200)<br>data.write_h5ad('./experiments/case19.2/output/filtered_data.h5ad')<br><br>conda install -y tangram<br>tangram --input ./experiments/case19.2/output/filtered_data.h5ad --<br>output ./experiments/case19.2/output/mapped_data.h5ad --<br>reference ./experiments/case19.2/data/slice1.h5ad --method<br>spatial_transcriptomics_mapping<br><br>conda install -y tangram<br>tangram --input ./experiments/case19.2/output/filtered_data.h5ad --<br>output ./experiments/case19.2/output/mapped_data.h5ad --<br>reference ./experiments/case19.2/data/slice1.h5ad --method<br>spatial_transcriptomics_mapping.<br><br>conda install -y tangram

	tangram --input ./experiments/case19.2/output/mapped_data.h5ad --output ./experiments/case19.2/output/predicted_data.h5ad --reference ./experiments/case19.2/data/slice1.h5ad --method spatial_transcriptomics_prediction.<br><br>conda install -y tangram<br>tangram --input ./experiments/case19.2/output/mapped_data.h5ad --output ./experiments/case19.2/output/evaluation_data.h5ad --reference ./experiments/case19.2/data/slice1.h5ad --method spatial_transcriptomics_evaluation --accuracy --precision<br><br>conda install -y tangram<br>tangram --input ./experiments/case19.2/output/mapped_data.h5ad --output ./experiments/case19.2/output/refined_data.h5ad --reference ./experiments/case19.2/data/slice1.h5ad --method spatial_transcriptomics_refinement --param1 value1 --param2 value2<br><br>conda install -y tangram<br>tangram --input ./experiments/case19.2/output/refined_data.h5ad --output ./experiments/case19.2/output/predicted_data.h5ad --reference ./experiments/case19.2/data/slice1.h5ad --method spatial_transcriptomics_prediction<br><br>conda install -y tangram<br>tangram --input ./experiments/case19.2/output/refined_data.h5ad --output ./experiments/case19.2/output/evaluation_data.h5ad --reference ./experiments/case19.2/data/slice1.h5ad --method spatial_transcriptomics_evaluation --accuracy --precision.

Proteomics Case 20.1: protein expression quantification

<table>
<tr><td>User's Inputs</td><td>Data and Description
01CPTAC_COprospective_W_PNNL_20170123_B1S1_f01.mzML.gz: protein mass spectrometry data in mzML format, tumor samples fraction 1
01CPTAC_COprospective_W_PNNL_20170123_B1S1_f02.mzML.gz: protein mass spectrometry data in mzML format, tumor samples fraction 2
01CPTAC_COprospective_W_PNNL_20170123_B1S1_f03.mzML.gz: protein mass spectrometry data in mzML format, tumor samples fraction 3
98CPTAC_COprospective_W_VU_20160804_11CO047_f01.mzML.gz: protein mass spectrometry data in mzML format, normal samples fraction 1
98CPTAC_COprospective_W_VU_20160804_11CO047_f02.mzML.gz: protein mass spectrometry data in mzML format, normal samples fraction 2
98CPTAC_COprospective_W_VU_20160804_11CO047_f03.mzML.gz: protein mass spectrometry data in mzML format, normal samples fraction 3
UP000005640_9606.fasta: reference protein sequence from UniProt

Goal
identify proteins in tumor and normal samples based on mass spectrometry data</td></tr>
<tr><td>Generated Plans by AutoBA</td><td>"step 1: Preprocessing of the mass spectrometry data using the software MS-Convert to convert the mzML files to the MGF format.", "step 2: Database search using the software MS-GF+ to identify the proteins in the tumor and normal samples.", "step 3: Protein inference using the software Percolator to combine the protein identification results from multiple samples and to correct for false discoveries.", "step 4: Protein functional annotation using the software InterProScan to identify the biological processes, cellular components, and molecular functions of the identified proteins.", "step 5: Data visualization and analysis using the software R to generate plots and statistics to summarize the results and to identify potential biomarkers for cancer diagnosis and treatment."</td></tr>
<tr><td>Generated Codes by AutoBA</td><td>conda install -y msconvert
msconvert ./experiments/case20.1/data/01CPTAC_COprospective_W_PNNL_20170123_B1S1_f01.mzML.gz ./experiments/case20.1/data/01CPTAC_COprospective_W_PNNL_20170123_B1S1_f02.mzML.gz ./experiments/case20.1/data/01CPTAC_COprospective_W_PNNL_20170123_B1S1_f03.mzML.gz ./experiments/case20.1/data/98CPTAC_COprospective_W_VU_20160804_11CO047_f01.mzML.gz ./experiments/case20.1/data/98CPTAC_COprospective_W_VU_20160804_11CO047_f02.mzML.gz ./experiments/case20.1/data/98CPTAC_COprospective_W_VU_20160804_11CO047_f03.mzML.gz -o ./experiments/case20.1/output/mgf

conda install -y msgfplus
msgfplus -d ./experiments/case20.1/data/UP000005640_9606.fasta -o ./experiments/case20.1/output/msgfplus.mzid ./experiments/case20.1/output/mgf/*.mgf

conda install -y percolator
percolator -i ./experiments/case20.1/output/msgfplus.mzid -o ./experiments/case20.1/output/percolator.mzid

conda install -y interproscan</td></tr>
</table>

	interproscan -i ./experiments/case20.1/output/percolator.mzid -o ./experiments/case20.1/output/interproscan.tsv<br><br>Rscript -e 'library(ggplot2); ggplot(data, aes(x = protein, y = expression)) + geom_point() + facet_grid(~ sample) + theme_bw()'

Metabolomics Case 21.1: metabolites concentration

**User's Inputs**	**Data and Description**<br>B1S1_f01.mzML.gz: LC-MS mass spectrometry data of metabolites in mzML format, tumor samples fraction 1<br>B1S1_f02.mzML.gz: LC-MS mass spectrometry data of metabolites in mzML format, tumor samples fraction 2<br>B1S1_f03.mzML.gz: LC-MS mass spectrometry data of metabolites in mzML format, tumor samples fraction 3<br>11CO047_f01.mzML.gz: LC-MS mass spectrometry data of metabolites in mzML format, normal samples fraction 1<br>11CO047_f02.mzML.gz: LC-MS mass spectrometry data of metabolites in mzML format, normal samples fraction 2<br>11CO047_f03.mzML.gz: LC-MS mass spectrometry data of metabolites in mzML format, normal samples fraction 3<br><br>**Goal**<br>calculate metabolites concentration in tumor and normal samples based on LC-MS mass spectrometry data
**Generated Plans by AutoBA**	"Step 1: Data Preprocessing", "Step 2: Feature Extraction", "Step 3: Data Transformation", "Step 4: Model Training", "Step 5: Model Evaluation", "Step 6: Metabolites Concentration Calculation"
**Generated Codes by AutoBA**	conda install -y -c bioconda msconvert<br><br>conda install -y -c bioconda msconvert && msconvert --mzML --outdir ./experiments/case21.1/output/mzML ./experiments/case21.1/data/*.mzML.gz<br><br>conda install -y -c bioconda msconvert && msconvert --mzML --outdir ./experiments/case21.1/output/mzML ./experiments/case21.1/data/*.mzML.gz<br><br>conda install -y -c bioconda msconvert && msconvert --mzML --outdir ./experiments/case21.1/output/mzML ./experiments/case21.1/data/*.mzML.gz<br><br>conda install -y -c bioconda msconvert && msconvert --mzML --outdir ./experiments/case21.1/output/mzML ./experiments/case21.1/data/*.mzML.gz && msconvert --mzML --outdir ./experiments/case21.1/output/mzML ./experiments/case21.1/data/*.mzML.gz<br><br>conda install -y -c bioconda msconvert && msconvert --mzML --outdir ./experiments/case21.1/output/mzML ./experiments/case21.1/data/*.mzML.gz && msconvert --mzML --outdir ./experiments/case21.1/output/mzML ./experiments/case21.1/data/*.mzML.gz

7. Results of AutoGPT

WGS data analysis   Case 1.1: Genome assembly

**User's Inputs**	You are a bioinformatician who specializes in writing code and performing analysis for a variety of bioinformatic data analysis tasks. Now I provide you with an Ubuntu 18.04 system where I save the following data, each line including the data path and data description.<br><br>SRR1234567_R1.fastq.gz: Paired-end Illumina WGS reads, forward<br>SRR1234567_R2.fastq.gz: Paired-end Illumina WGS reads, reverse<br>TruSeq3-PE.fa: Adapter sequences for trimming<br>Reference.fasta: Reference genome for the organism under study in FASTA format<br><br>I need you to help me plan what needs to be done in each step of the analysis process, and help me write the code for each step to install softwares, use tools and accomplish the following main goal:<br><br>To perform genome assembly using paired-end WGS data
**Generated Plans by AutoGPT**	Agent message<br>Based on the goal, here is the plan for performing genome assembly using paired-end WGS data:<br><br>1. **Install Required Software**:<br>- Install the necessary software for genome assembly, such as SPAdes or Velvet.<br>- Use the package manager (e.g., apt-get) to install the software on Ubuntu 18.04.<br><br>2. **Download and Prepare Data**:<br>- Download the paired-end Illumina WGS reads (SRR1234567_R1.fastq.gz and SRR1234567_R2.fastq.gz) and the adapter sequences (TruSeq3-PE.fa) to your Ubuntu system.<br>- Create a directory to store the data and move the downloaded files to that directory.<br>- Unzip the fastq.gz files if necessary.<br><br>3. **Quality Control and Trimming**:<br>- Perform quality control on the raw reads using tools like FastQC or Trimmomatic.<br>- Use the adapter sequences (TruSeq3-PE.fa) to trim the reads and remove any adapter contamination or low-quality regions.<br><br>4. **Genome Assembly**:<br>- Use the trimmed reads as input for the genome assembly software (SPAdes or Velvet).<br>- Specify the appropriate parameters for the assembly, such as read length, k-mer size, and coverage cutoffs.

- Run the assembly process and generate the assembled genome.

5. **Evaluate and Validate the Assembly**:
   - Assess the quality of the assembled genome using tools like QUAST or BUSCO.
   - Compare the assembled genome to the reference genome (Reference.fasta) to evaluate its accuracy and completeness.

6. **Documentation and Reporting**:
   - Document the entire analysis process, including the software used, parameters, and steps performed.
   - Generate a report summarizing the results, including statistics on the assembled genome and any identified issues or limitations.
   - Provide instructions on how to access and share the final assembled genome.

Please note that the above plan provides a general outline for the analysis process. The specific commands and code may vary depending on the software versions, data format, and specific requirements of your analysis.

The formatting requirements are as follows:

1. Organize the data description and data path in a clear and readable format.
2. Use a consistent naming convention for the data files and their descriptions.
3. Use proper punctuation and capitalization for clarity and readability.
4. Use a bulleted or numbered list to outline the steps of the analysis process.
5. Use code formatting or a monospaced font to distinguish code snippets or commands from regular text.
6. Clearly specify the main goal of the analysis process.

Here's an example of how the information could be formatted:

Data:
- SRR1234567_R1.fastq.gz: Paired-end Illumina WGS reads, forward
- SRR1234567_R2.fastq.gz: Paired-end Illumina WGS reads, reverse
- TruSeq3-PE.fa: Adapter sequences for trimming
- Reference.fasta: Reference genome for the organism under study in FASTA format

Analysis Process:
1. Install necessary software and tools:
   - [Software 1]
   - [Software 2]
   - [Tool 1]
   - [Tool 2]

2. Preprocess the data:
   - Trim adapter sequences using [Tool 1] and [TruSeq3-PE.fa]
   - Quality control and filtering of reads

	3. Perform genome assembly:<br>- Use [Tool 2] for genome assembly<br>- Input paired-end WGS data [SRR1234567_R1.fastq.gz] and [SRR1234567_R2.fastq.gz]<br>- Utilize [Reference.fasta] as the reference genome<br><br>Main Goal: Perform genome assembly using paired-end WGS data.

WGS/WES data analysis **Case 2.1 and Case 2.2 : Somatic SNV+indel calling and annotation**

**User's Inputs**	You are a bioinformatician who specializes in writing code and performing analysis for a variety of bioinformatic data analysis tasks. Now I provide you with an Ubuntu 18.04 system where I save the following data, each line including the data path and data description.<br><br>SRR23015874_R1.fastq.gz: paired end read1 from human whole genome sequencing, normal samples replicate 1<br>SRR23015874_R2.fastq.gz: paired end read2 from human whole genome sequencing, normal samples replicate 1<br>SRR23015876_R1.fastq.gz: paired end read1 from human whole genome sequencing, tumor samples replicate 1<br>SRR23015876_R2.fastq.gz: paired end read2 from human whole genome sequencing, tumor samples replicate 1<br>SRR23015877_R1.fastq.gz: paired end read1 from human whole genome sequencing, tumor samples replicate 2<br>SRR23015877_R2.fastq.gz: paired end read2 from human whole genome sequencing, tumor samples replicate 2<br>TruSeq3-PE.fa: trimming adapter<br>hg38.fa: human hg38 reference genome fasta<br><br>I need you to help me plan what needs to be done in each step of the analysis process, and help me write the code for each step to install softwares, use tools and accomplish the following main goal:<br><br>call somatic mutations in tumor samples over the matched normal samples and annotate the identified mutations
**Generated Plans by AutoGPT**	Based on the provided data and the main goal, here is the plan for each step of the analysis process:<br><br>1. **Data Preparation**:<br>- Install necessary software and tools for the analysis process.<br>- Organize the required data files.<br><br>2. **Quality Control and Preprocessing**:<br>- Perform quality control checks on the raw sequencing data.<br>- Trim adapter sequences from the reads.<br>- Perform read alignment to the reference genome (hg38.fa).<br><br>3. **Variant Calling**:<br>- Use a variant calling tool to identify variants in the tumor and normal samples.<br>- Perform variant calling separately for each sample replicate.<br>- Apply appropriate filters to remove low-quality variants and false positives.<br><br>4. **Somatic Mutation Calling**:<br>- Compare the variants called in the tumor samples with the variants in the matched normal samples.<br>- Identify somatic mutations by filtering out variants present in the normal samples.<br><br>5. **Variant Annotation**:

	- Annotate the identified somatic mutations with relevant information.<br>- Retrieve functional consequences, gene annotations, and other relevant information for each mutation.<br><br>6. **Result Analysis and Interpretation**:<br>- Analyze and interpret the annotated somatic mutations to understand their potential impact on genes and pathways.<br>- Perform downstream analysis such as pathway enrichment analysis or functional prediction of the mutated genes.<br><br>The specific software and tools used may vary depending on your preferences and the requirements of your analysis. The code for each step will depend on the chosen tools and programming language.<br><br>Formatting Requirements:<br><br>1. Organize the data description in a clear and structured manner, preferably in a list format.<br>2. Use a consistent naming convention for the data files, such as italicizing or bolding the file names.<br>3. Separate the data path and data description for each file using a colon or any other suitable delimiter.<br>4. Use a clear and concise language to describe the data, specifying relevant details such as paired-end reads, sample types, and replicates.<br>5. Use a consistent formatting style for the code, such as indentation and line breaks, to enhance readability.<br>6. Clearly label each step of the analysis process and code implementation.<br>7. Use appropriate formatting for file names, such as italicizing or bolding, to distinguish them from regular text.<br>8. Clearly state the main goal of the analysis process, such as calling somatic mutations and annotating them.<br>9. Use appropriate formatting for software names, such as italicizing or bolding, to distinguish them from regular text.<br>10. Clearly separate different sections or steps of the analysis process, such as using headings or numbering.

WGS/WES data analysis **Case 2.3: Structure variation identification with normal**

<table>
<tr><td>User's Inputs</td><td>You are a bioinformatician who specializes in writing code and performing analysis for a variety of bioinformatic data analysis tasks. Now I provide you with an Ubuntu 18.04 system where I save the following data, each line including the data path and data description.

SRR23015874_R1.fastq.gz: paired end read1 from human whole genome sequencing, the matched normal samples replicate 1
SRR23015874_R2.fastq.gz: paired end read2 from human whole genome sequencing, the matched normal samples replicate 1
SRR23015876_R1.fastq.gz: paired end read1 from human whole genome sequencing, tumor samples replicate 1
SRR23015876_R2.fastq.gz: paired end read2 from human whole genome sequencing, tumor samples replicate 1
SRR23015877_R1.fastq.gz: paired end read1 from human whole genome sequencing, tumor samples replicate 2
SRR23015877_R2.fastq.gz: paired end read2 from human whole genome sequencing, tumor samples replicate 2
TruSeq3-PE.fa: trimming adapter
hg38.fa: human hg38 reference genome fasta

I need you to help me plan what needs to be done in each step of the analysis process, and help me write the code for each step to install softwares, use tools and accomplish the following main goal:

To identify structural variations, which can include large insertions, deletions, inversions, duplications, and translocations, by comparing the sequencing data from the tumor sample over the matched normal sample</td></tr>
<tr><td>Generated Plans by AutoGPT</td><td>Agent message
Based on the goal, here is the research plan:

1. **Understand the Data**: The first step is to understand the data provided. The data includes paired end reads from human whole genome sequencing for both normal and tumor samples, trimming adapter, and human hg38 reference genome fasta.

2. **Identify Required Tools**: The next step is to identify the tools required for the analysis. This includes tools for quality control of sequencing data, alignment of sequencing reads to the reference genome, detection of structural variations, and data analysis and visualization.

3. **Install Required Tools**: Once the tools are identified, the next step is to install these tools on the Ubuntu 18.04 system.

4. **Preprocess the Data**: The data needs to be preprocessed before the analysis. This includes quality control checks on the sequencing data files and identification of any potential issues with the data.

5. **Align Sequencing Reads to the Reference Genome**: The next step is to align the sequencing reads from the tumor and matched normal samples to the reference genome.</td></tr>
</table>

	6. **Identify Structural Variations**: After the alignment, the next step is to identify structural variations by comparing the sequencing data from the tumor sample over the matched normal sample.<br><br>7. **Annotate and Visualize the Data**: The final step is to annotate and visualize the data. This includes intersecting the identified structural variations with known genomic features and analyzing and visualizing the structural variations.<br><br>The research will be conducted by performing web searches for each of these steps to gather the necessary information and resources. The results will be consolidated and presented in a clear and organized manner.

WGS/WES data analysis **Case 2.4: Structure variation identification without normal**

<table>
<tr>
<td>User’s Inputs</td>
<td>You are a bioinformatician who specializes in writing code and performing analysis for a variety of bioinformatic data analysis tasks. Now I provide you with an Ubuntu 18.04 system where I save the following data, each line including the data path and data description.

SRR23015876_R1.fastq.gz: paired end read1 from human whole genome sequencing, tumor samples replicate 1
SRR23015876_R2.fastq.gz: paired end read2 from human whole genome sequencing, tumor samples replicate 1
SRR23015877_R1.fastq.gz: paired end read1 from human whole genome sequencing, tumor samples replicate 2
SRR23015877_R2.fastq.gz: paired end read2 from human whole genome sequencing, tumor samples replicate 2
TruSeq3-PE.fa: trimming adapter
hg38.fa: human hg38 reference genome fasta

I need you to help me plan what needs to be done in each step of the analysis process, and help me write the code for each step to install softwares, use tools and accomplish the following main goal:

To identify structural variations, which can include large insertions, deletions, inversions, duplications, and translocations based on the sequencing data from the tumor sample.</td>
</tr>
<tr>
<td>Generated Plans by AutoGPT</td>
<td>## Step 1

Action executed:
Web search for 'best software for identifying structural variations in bioinformatics'
Function Call:
```javascript
web_search({
  "queries": [
    "best software for identifying structural variations in bioinformatics"
  ]
})
```
Result
```
Found the following result for the web search: 'best software for identifying structural variations in bioinformatics'

The software for identifying structural variations in bioinformatics mentioned in the text are:

1. BreakPoint surveyor
2. MAVIS
3. SoftSV
4. SvABA
5. Wham
6. GRIDSS
7. inGAP-sv</td>
</tr>
</table>

8. laSV
9. Lumpy
10. Manta
11. PBHoney-NGM
12. pbsv
13. PRISM
14. SVEngine (v1.0.0)
15. VISOR (v1.1)

The text does not provide information on which of these is the best.

```
## Step 2

### Action executed:
Web search for 'how to install bioinformatics software on Ubuntu 18.04'
### Function Call:
```javascript
web_search({
  "queries": [
    "how to install bioinformatics software on Ubuntu 18.04"
  ]
})
```
Result
```
Found the following result for the web search: 'how to install bioinformatics software on Ubuntu 18.04'
--------------
1. Use conda with the bioconda channel activated to install and manage your bioinformatics tools.

2. To install tools from the PAPPSO Ubuntu Bionic Beaver repository, add the official package repository to your system. Then, you can install any proteomic software from the repository with the command: sudo apt install xtpcpp masschroq-studio masschroq-gui.

3. Install JAVA if you don't have it already using the command: sudo apt install default-jre.

4. Download fastqc using the following commands:
   - mkdir -p ~/Downloads/bin
   - cd ~/Downloads/bin
   - wget
https://www.bioinformatics.babraham.ac.uk/projects/fastqc/fastqc_v0.11.8.zip
   - unzip fastqc_v0.11.8.zip
   - rm fastqc_v0.11.8.zip
   - sudo chmod 770 FastQC/fastqc

5. To install BWA, download and extract the source code using the following commands:
  - cd ~/workspace/bin
  - wget https://cytranet.dl.sourceforge.net/project/bio-bwa/bwa-0.7.17.tar.bz2
  - tar --bzip2 -xvf bwa-0.7.17.tar.bz2
  - cd  bwa-0.7.17
  - make
  - ln -s ~/workspace/bin/bwa-0.7.17/bwa ~/workspace/bin/bwa

6. To check the installation, use the command: ~/workspace/bin/bwa.

(Note: The information about installing GATK 4 is incomplete.)

--------------

```
Step 3

Action executed:
Web search for 'how to preprocess sequencing data in bioinformatics'
Function Call:
```javascript
web_search({
  "queries": [
    "how to preprocess sequencing data in bioinformatics"
  ]
})
```
Result
```
Found the following result for the web search: 'how to preprocess sequencing data in bioinformatics'
--------------
1. Preprocessing of Next Generation Sequencing data involves several steps, including read alignment, transcript quantification, and differential expression analysis. This ensures that the data is ready for downstream analyses, such as variant calling, differential expression analysis, and functional annotation.

2. Data preprocessing can be affected by various factors such as PCR or sample contamination, sequencing quality, data contamination caused by index hopping when splitting data, or alignment software or specific parameter adjustment of downstream personalized analysis.

3. Data preprocessing software and algorithms such as Cutadapt, FastP, and Trimmomatic have shown excellent results in obtaining high-quality clean data for downstream analysis. However, the choice of software and algorithms needs to be made carefully, and the data preprocessing algorithms need to be further improved according to actual data features.

4. Tools like Trimmomatic and Cutadapt can be used to remove low-quality reads based on quality score thresholds.

5. The FASTQ Preprocessing tool uses the well-known preprocessing software Trimmomatic, which is a fast, multithreaded command line tool that can be used to trim and crop sequencing data as well as to remove adapters.

6. Sequence Quality Trimming is a common step in preprocessing to cope with lower quality data. Typically, lower quality bases from the ends of reads are removed.

7. Quality Check and preprocessing are necessary to avoid bioinformatic procedures being compromised by low-quality sequences, sequence artifacts, and sequence contamination, which can lead to misassembly and erroneous conclusions.

(Note: The information is incomplete as it does not provide detailed steps or parameters for each preprocessing step.)

--------------

```
Step 4

Action executed:
Web search for 'how to align sequencing data to reference genome'
Function Call:
```javascript
web_search({
  "queries": [
    "how to align sequencing data to reference genome"
  ]
})
```
Result
```
Found the following result for the web search: 'how to align sequencing data to reference genome'
--------------
1. Upon receiving next generation sequencing data, the first phase of bioinformatic analysis involves aligning or mapping the reads to the reference genome.

2. Before aligning sequences to a reference genome, it is necessary to index the genome using `bwa`. This only needs to be done once as the process creates an index that is stored on the hard drive alongside the genome. The syntax for this is: `bwa index path-to-genome`. For example: `bwa index genome/GCA_002872995.1_Otsh_v1.0_genomic.fna.gz`.

3. The indexing process allows for an aligner to work more efficiently by finding all exact matches to a sequenced read using a single lookup in the given data structure and not scanning the entire reference genome from start to finish for each read.

4. The alignment process includes building a large index database from a reference genome and/or the set of reads. Global positioning is performed to determine the potential positions of each read in the reference genome. Alignment algorithms use the prepared index to determine one or more possible regions of the reference genome that are likely to be similar to each read sequence. Lastly, pairwise alignment is performed between the read and the reference sequence.

5. The global positioning step uses the generated genome index to retrieve the locations (in the genome) of various seeds extracted from the sequencing reads. The read alignment algorithm uses the determined seed locations to reduce the search space from the entire reference genome to only the neighborhood region of each seed location.

6. There are over 60 different mappers, and their number is growing. In this tutorial, Bowtie2 is used, a fast and memory-efficient open-source tool particularly good at aligning sequencing reads of about 50 up to 1,000s.

7. Sequencing produces a collection of sequences without genomic context. Mapping the reads of an experiment to a reference genome is a key step in modern genomic data analysis. With the mapping the reads are assigned to a specific location in the genome and insights like the expression level of genes can be gained. The short reads do not come with position information, so we do not know what part of the genome they came from. We need to use the sequence of the read to find its origin in the genome.

Note: The information is incomplete as it does not provide detailed steps on how to perform the alignment using specific tools or software.

--------------

```
Step 5

Action executed:
Web search for 'how to detect structural variations from aligned data'
Function Call:
```javascript
web_search({
  "queries": [
    "how to detect structural variations from aligned data"
  ]
})
```
Result
```
Found the following result for the web search: 'how to detect structural variations from aligned data'
--------------
To detect structural variations (SVs) from aligned data, the following steps can be taken:

1. De novo-assembled sequences can be aligned to a reference or other assembly. The alterations between the two can be systematically identified by comparing each position in one genome to its corresponding position in the other genome. This should allow the identification of all forms of variations.

2. Use of specialized long-read alignment algorithms. However, there is no best practice for which aligner should be used when performing SV detection.

3. Combinatorial algorithms can be used for structural variation detection in high-throughput sequenced genomes. How these algorithms integrate read-alignment patterns influences their ability to detect specific variant classes.

4. Structural variation, including large deletions, duplications, inversions, translocations, and other rearrangements, is common in human and cancer genomes. A number of methods have been developed to identify structural variants from Illumina short-read sequencing data. However, reliable identification of structural variants remains challenging because many variants have breakpoints in repetitive regions of the genome and thus are difficult to identify with short reads.

5. The recently developed linked-read sequencing technology from 10X Genomics can be used. For cuteSV, NGMLR alignment results can be used as it was benchmarked in its research. Since the default parameters of the two software programs were not optimized for low-dept, to increase sensitivity, multiple combinations of parameters can be tried.

Note: The information does not provide specific decimal precision as requested.

--------------

```
Step 6

Action executed:
Web search for 'how to analyze and interpret detected structural variations'
Function Call:
```javascript
web_search({
  "queries": [
    "how to analyze and interpret detected structural variations"
  ]
})
```
Result
```
Found the following result for the web search: 'how to analyze and interpret detected structural variations'
--------------

| | |
|---|---|
| | 1. Structural variation is a common form of interindividual genetic variation. Balanced structural variation poses bigger challenges and has been less studied than unbalanced forms.<br>2. Structural variants are mostly discovered through array-CGH, intensity analysis from SNP arrays and read-pair and RD analysis of high-throughput sequencing data.<br>3. The most well-studied types of structural variants are those involving change in copy number of genetic material, as those are easier to detect with current technologies.<br>4. Detection of balanced structural variants, aside from those large enough to be visible on a karyotype, generally involves the analysis of paired-end NGS data and the application of complex algorithms.<br>5. In 2004, the field of SV analysis was opened thanks to the development of array-CGH, which remains a robust method for discovery of CNV.<br>6. To detect SVs, de novo-assembled sequences can be aligned to a reference or other assembly, and the alterations between the two can be systematically identified: the comparison of each position in one genome to its corresponding position in the other genome should allow the identification of all forms of variations.<br>7. SVs are associated with diverse diseases and are a notable hallmark of cancer genomes. Long-reads, linked-reads, Hi-C, and optical mapping resolve structures that short-reads struggle to detect in the majority of cancers such as inter-and-intra chromosomal translocations, complex rearrangements.<br>8. Accurate detection of complex structural variations can be achieved using single-molecule sequencing.<br><br>Note: The information is incomplete as it does not provide specific steps or methods for analyzing and interpreting detected structural variations.<br><br>-------------- |

**ChIP-Seq Analysis Case 3.1: Call Peaks**

| | |
|---|---|
| **User's Inputs** | You are a bioinformatician who specializes in writing code and performing analysis for a variety of bioinformatic data analysis tasks. Now I provide you with an Ubuntu 18.04 system where I save the following data, each line including the data path and data description.<br><br>SRR620204.fastq.gz: chip-seq data for Ring1B<br>SRR620205.fastq.gz: chip-seq data for cbx7<br>SRR620206.fastq.gz: chip-seq data for SUZ12<br>SRR620208.fastq.gz: chip-seq data for IgGold<br>mm39.ncbiRefSeq.gtf: genome annotations mouse<br>mm39.fa: mouse genome<br><br>I need you to help me plan what needs to be done in each step of the analysis process, and help me write the code for each step to install softwares, use tools and accomplish the following main goal:<br><br>Call peaks for protein cbx7 with IgGold as control |

| | |
|---|---|
| **Generated Plans by AutoGPT** | ## Step 1<br><br>### Action executed:<br>Agent message<br>Based on the goal, here is the plan for the analysis process:<br><br>1. **Install necessary software**: Install the required software for chip-seq analysis, such as Bowtie, MACS2, and BEDTools. You can use package managers like apt or conda to install these tools.<br><br>2. **Download and prepare the reference genome and annotations**: Download the mouse genome (mm39.fa) and genome annotations (mm39.ncbiRefSeq.gtf) provided. Index the genome using Bowtie or a similar tool to create a reference index for alignment.<br><br>3. **Align the chip-seq data for cbx7 and IgGold to the reference genome**: Use Bowtie or a similar aligner to align the chip-seq data (SRR620205.fastq.gz for cbx7 and SRR620208.fastq.gz for IgGold) to the indexed mouse genome. Convert the aligned reads to BAM format for further analysis.<br><br>4. **Call peaks for cbx7 using IgGold as the control**: Use MACS2 or a similar peak-calling tool to identify peaks for cbx7 using IgGold as the control. Adjust the parameters of the peak-calling tool based on the specific experiment and data quality. Generate peak files in BED format for downstream analysis.<br><br>Please note that the provided plan is a general outline, and you may need to adapt it based on your specific requirements and preferences.<br><br>The formatting requirements are as follows:<br><br>1. **Data Path and Description**: Each line should include the data path and a brief description of the data. The format should be as follows: [Data Path]: [Data Description]<br><br>2. **Code Steps**: The code steps should be clearly outlined and organized. Each step should be numbered or bulleted for easy reference.<br><br>3. **Software Installation**: If any software needs to be installed, it should be mentioned in the code steps along with the necessary commands or instructions.<br><br>4. **Tool Usage**: If any specific tools need to be used for the analysis, they should be mentioned in the code steps along with the necessary commands or instructions.<br><br>5. **Main Goal**: The main goal should be clearly stated, specifying the protein (cbx7) and the control (IgGold) to be used for calling peaks. |

**ChIP-Seq Analysis      Case 3.2: Motif Discovery**

**User's Inputs**

You are a bioinformatician who specializes in writing code and performing analysis for a variety of bioinformatic data analysis tasks. Now I provide you with an Ubuntu 18.04 system where I save the following data, each line including the data path and data description.

SRR620204.fastq.gz: chip-seq data for Ring1B
SRR620205.fastq.gz: chip-seq data for cbx7
SRR620206.fastq.gz: chip-seq data for SUZ12
SRR620208.fastq.gz: chip-seq data for IgGold
mm39.ncbiRefSeq.gtf: genome annotations mouse
mm39.fa: mouse genome

I need you to help me plan what needs to be done in each step of the analysis process, and help me write the code for each step to install softwares, use tools and accomplish the following main goal:

Discover motifs within the peaks of protein SUZ12 with IgGold as control

**Generated Plans by AutoGPT**

## Step 1

### Action executed:
Web search for 'how to install bedtools on Ubuntu 18.04'
### Function Call:
```javascript
web_search({
  "queries": [
    "how to install bedtools on Ubuntu 18.04"
  ]
})
```
Result
```
Found the following result for the web search: 'how to install bedtools on Ubuntu 18.04'
--------------
To install bedtools on Ubuntu 18.04, follow these steps:

1. Update your APT package manager by entering the following command in the terminal: `sudo apt update`
2. Install bedtools by entering the following command in the terminal: `sudo apt install bedtools`

Please note that the information provided does not specify decimal precision as it is not applicable in this context.

--------------

```
Step 2

Action executed:
Web search for 'how to install MACS2 on Ubuntu 18.04'
Function Call:

```javascript
web_search({
  "queries": [
    "how to install MACS2 on Ubuntu 18.04"
  ]
})
```
Result
```
Found the following result for the web search: 'how to install MACS2 on Ubuntu 18.04'
--------------
To install MACS2 on Ubuntu 18.04, you can follow these steps:

1. You need to have root permission to install software with pip, unless you have installed a different Python distribution by your own. You may want to check the solution of 'virtual environment' to install software to your own user space.

2. Under command line, type: `$ pip install MACS2`. PyPI will install Numpy automatically if they are absent.

3. To upgrade, type: `$ pip install -U MACS2`.

4. Alternatively, you could try installing with `pip install MACS2 --user`.

5. You could create a virtual environment and install MACS2 in that with `pip install MACS2`.

6. Download and install `conda` (which you can do into your home dir) and then install using `conda` from the `bioconda` channel.

Please note that MACS2 requires python 2.

--------------

```
Step 3

Action executed:
Web search for 'how to install MEME Suite on Ubuntu 18.04'
Function Call:
```javascript
web_search({
  "queries": [
    "how to install MEME Suite on Ubuntu 18.04"
  ]
})
```
Result
```
Found the following result for the web search: 'how to install MEME Suite on Ubuntu 18.04'
```

To install MEME Suite on Ubuntu 18.04, follow these steps:

1. Download the software from https://meme-suite.org/doc/download.html

2. Unzip and untar the distribution using the command: `tar zxf meme-.tar.gz`

3. Navigate to the meme directory using the command: `cd meme-`

4. Configure the installation using the command: `./configure --prefix=$HOME/meme --enable-build-libxml2 --enable-build-libxslt`

5. Build MEME Suite using the command: `make`

6. Test the MEME Suite build by using the command: `make test`

7. Install MEME Suite using the command: `make install`

8. Edit your shell configuration file to add `$HOME/meme/bin` and `$HOME/meme/libexec/meme-version #` to your PATH. For bash, you can add the following line to your `.profile` file: `export PATH=your install path/bin:$PATH`

Please note that the information is incomplete as the exact version of MEME Suite to be downloaded is not specified, and the exact path to be added to the `.profile` file is not provided.

```
## Step 4

### Action executed:
Web search for 'how to use FastQC for quality control'
### Function Call:
```javascript
web_search({
  "queries": [
    "how to use FastQC for quality control"
  ]
})
```
Result
```
Found the following result for the web search: 'how to use FastQC for quality control'
--------------
To use FastQC for quality control, follow these steps:

1. Import data from BAM, SAM, or FastQ files.
2. FastQC provides a quick overview to identify potential problems in the data.

3. It generates summary graphs and tables for quick assessment of the data.
4. The results are exported to an HTML-based report for easy viewing.

Note: Before running FastQC, ensure you are on a compute node in an interactive session. If not, run the `srun` command.

FastQC can also be run with a graphical interface, but for didactic purposes, it is often run in the terminal.

After running FastQC, you can use MultiQC to aggregate bioinformatics analysis results from many samples into a single report. Set the files generated by FastQC as input, and the MultiQC result will be stored in `1-qualityControl_beforeTrimming/outputMultiqc`.

Please note that the information provided does not include specific commands or code to run FastQC or MultiQC.

--------------

```
Step 5

Action executed:
Web search for 'how to use Trimmomatic for read trimming'
Function Call:
```javascript
web_search({
  "queries": [
    "how to use Trimmomatic for read trimming"
  ]
})
```
Result
```
Found the following result for the web search: 'how to use Trimmomatic for read trimming'
--------------
To use Trimmomatic for read trimming, follow these steps:

1. Download and install the binary version of Trimmomatic from the Usadel lab website.

2. Create a shortcut to call the command by typing `trimmomatic` rather than remembering the more complicated java command line every time. You can do this by adding a line like this to your bash profile: `alias trimmomatic='java -jar /Users/name/local/bin/trimmomatic-0.XX.jar'`.

3. Load the Trimmomatic module using the command `$ module load Trimmomatic`.

4. Run the command `$ trimmomatic` to see some of your options.

5. A complete command for Trimmomatic will look something like the command below. This command is an example and will not work, as we do not have the files it refers to:

```bash
$ trimmomatic PE -threads 4 SRR_1056_1.fastq SRR_1056_2.fastq  \
SRR_1056_1.trimmed.fastq SRR_1056_1un.trimmed.fastq \
SRR_1056_2.trimmed.fastq SRR_1056_2un.trimmed.fastq \
ILLUMINACLIP:SRR_adapters.fa SLIDINGWINDOW:4:20
```

In this example, we have told Trimmomatic:

- `PE` that it is paired end data.
- `-threads 4` to use 4 threads.
- `SRR_1056_1.fastq` and `SRR_1056_2.fastq` are the input files.
- `SRR_1056_1.trimmed.fastq`, `SRR_1056_1un.trimmed.fastq`, `SRR_1056_2.trimmed.fastq`, `SRR_1056_2un.trimmed.fastq` are the output files.
- `ILLUMINACLIP:SRR_adapters.fa` to clip the Illumina adapters.
- `SLIDINGWINDOW:4:20` to use a sliding window approach to trim the reads.

Note: The flags are followed by positional arguments, meaning the order in which you specify them is important. In paired end mode, Trimmomatic expects the two input files, and then the names of the output files. In single end mode, Trimmomatic will expect 1 file as input, after which you can enter the output file names.

```
## Step 6

### Action executed:
Web search for 'how to align reads with Bowtie'
### Function Call:
```javascript
web_search({
  "queries": [
    "how to align reads with Bowtie"
  ]
})
```
Result
```
Found the following result for the web search: 'how to align reads with Bowtie'
--------------
1. Install Bowtie and change to the directory containing Bowtie. For instance, if Bowtie is installed in a directory named /Users/joe/software/bowtie, type: cd /Users/joe/software/bowtie.

2. Align the reads to the _E. coli_ genome with Bowtie and save the SAM output to a temporary file: ./bowtie –S indexes/e_coli reads/e_coli_10000snp.fq > /tmp/e_coli.sam.
3. Convert the SAM output to BAM format: samtools view –bS –o /tmp/e_coli.bam /tmp/e_coli.sam.
4. Bowtie can output SAM (using the -S/--sam option), and SAM can be converted to BAM using SAMtools.
5. Bowtie2 will perform a global end-to-end read alignment, which aligns from the first to the last base of the read. This alignment is best for reads that have already been trimmed for quality and adapters.
6. Bowtie2 also has a local alignment mode, which ignores portions of the read that do not align well.
7. To align reads to an indexed genome/sequence using `bowtie`, use the command: $ bowtie --no-unal --threads n --sam index_name -1 reads_1.fastq -2 reads_2.fastq output.sam.
8. The first argument to bowtie is the basename of the index for the genome to be searched. The second argument is the name of a FASTQ file containing the reads.
9. The `--no-unal` is an optional argument, meaning reads that do not align to the reference sequence will not be written to the output.
10. The Bowtie package enables ultrafast and memory-efficient alignment of large sets of sequencing reads to a reference sequence, such as the human genome. The package contains tools for building indexes of reference genomes and for aligning short reads using the index as a guide.
11. When you select the Tools ‣ Align to reference ‣ Align short reads item in the main menu, the Align Sequencing Reads dialog appears. Set value of the Align short reads method parameter to Bowtie 2. Specify the Reference sequence — DNA sequence to align short reads to. This parameter is required. Specify the Result file name — file in SAM format to write the result of the alignment into. This parameter is required.

Note: The information is incomplete as it does not provide detailed steps on how to install Bowtie, how to build indexes of reference genomes, and how to handle errors during the process.

--------------

```

Step 7

Action executed:
Web search for 'how to call peaks with MACS2'
Function Call:
```javascript
web_search({
  "queries": [
    "how to call peaks with MACS2"
  ]
})
```
Result
```

Found the following result for the web search: 'how to call peaks with MACS2'

To call peaks with MACS2, you can use the `callpeak` function. This is the main function in MACS2 and can be invoked by typing `macs2 callpeak`. If you type this command without parameters, you will see a full description of command-line options.

Here are some commonly used options:

- `-t`: The IP data file (this is the only REQUIRED parameter for MACS)
- `-c`: The control or mock data file

For example, for regular peak calling, you can use the following command: `macs2 callpeak -t ChIP.bam -c Control.bam -f BAM -g hs -n test -B -q 0.01`.

For broad peak calling, you can use the following command: `macs2 callpeak -t ChIP.bam -c Control.bam --broad -g hs --broad-cutoff 0.1`.

Please note that the information might be incomplete and you might need to refer to the MACS2 documentation or use `macs2 COMMAND -h` to find out more.

```
## Step 8

### Action executed:
Web search for 'how to use bedtools for peak intersection'
### Function Call:
```javascript
web_search({
  "queries": [
    "how to use bedtools for peak intersection"
  ]
})
```
Result
```
Found the following result for the web search: 'how to use bedtools for peak intersection'
--------------
To use bedtools for peak intersection, follow these steps:

1. Use the `bedtools intersect` command. This command evaluates file A and finds regions that overlap in file B. It can be used for filtering of peaks and assessing the overlap of peaks.

Command: `$ bedtools intersect -h`

2. If you have multiple bed files and you want to retain peaks in at least two out of three files, you can use `bedtools intersect` to retain the peaks in the general file that overlap with peaks in the smaller files.

Command: `bedtools intersect -a general_file.bed -b smaller_file1.bed smaller_file2.bed smaller_file3.bed > output.bed`

3. If you want to get the intersection of peaks into a new file, you can use the following command:

Command: `bedtools intersect -a file1.bed -b file2.bed file3.bed file4.bed file5.bed -wo > intersected.bed`

4. To obtain the peak union (common peaks + unique peaks) from all the samples, concatenate all the BED files of peaks to a single file and then do a bedtools merge to get the peak union.

Command: `cat file1.bed file2.bed file3.bed > union.bed`
`bedtools merge -i union.bed > merged.bed`

5. To obtain the peak intersection, create a list of BED files and use a for loop over this list of BED files to do intersectBed between the peak union and each sample specific BED file.

6. If you want to use `bedtools intersect` with multiple files after `-b`, it performs pairwise intersections between `-a` and each file in `-b`. It does not intersect all files simultaneously.

Note: The information provided does not include decimal precision as it is not applicable in this context.

--------------

```
Step 9

Action executed:
Web search for 'how to use MEME Suite for motif discovery'
Function Call:
```javascript
web_search({
  "queries": [
    "how to use MEME Suite for motif discovery"
  ]
})
```
Result
```
Found the following result for the web search: 'how to use MEME Suite for motif discovery'
--------------
To use MEME Suite for motif discovery, follow these steps:

1. The MEME Suite allows the discovery of novel motifs in collections of unaligned nucleotide or protein sequences. It supports motif-based analysis of DNA, RNA, and protein sequences.

2. The Suite provides motif discovery algorithms using both probabilistic (MEME) and discrete models (STREME), which have complementary strengths. It also allows discovery of motifs with arbitrary insertions and deletions (GLAM2).

3. The MEME Suite provides three tools for motif enrichment analysis--measuring the enrichment of known motifs in sets of sequences. The motif enrichment may be anywhere in the sequences (SEA, AME), or concentrated in the central regions of the sequences (CentriMo).

4. The Suite also provides an algorithm for scanning sequences for matches to motifs (FIMO, MAST, and GLAM2Scan), scanning for clusters of motifs (MCAST), finding preferred spacings between motifs (SpaMo), predicting the biological roles of motifs (GOMo), and predicting the regulatory targets of transcription factors.

5. Not all the tools are available as web services, so to get the full power of the MEME Suite you will need to download and install a local copy of the software.

6. The web-based version of the MEME Suite comprises an integrated set of tools and databases for performing motif-based sequence analyses. The core of the suite is the meme motif discovery algorithm, which finds motifs in unaligned sequences.

7. The user interfaces are consistent and flexible. You can input sequences required by the first three groupings either by selecting a file for upload or by typing (or cut-and-paste). All the tools that accept motifs as input (for enrichment, scanning, or comparison) allow you to upload them by selecting a file name or by typing or cutting-and-pasting one or more motifs in any of a number of different formats.

8. The MEME Suite is freely available for academic use at http://meme-suite.org, and source code is also available for download and local installation.

Please note that the information is incomplete and does not provide a step-by-step guide on how to use the software.

--------------

```
Step 10

Action executed:
Web search for 'how to validate motifs with JASPAR or TRANSFAC'
Function Call:
```javascript
web_search({

"queries": [
  "how to validate motifs with JASPAR or TRANSFAC"
]
})
```
Result
```
Found the following result for the web search: 'how to validate motifs with JASPAR or TRANSFAC'
--------------
To validate motifs with JASPAR or TRANSFAC, the following steps can be followed:

1. The motifs in JASPAR are collected in two ways: Internally, by analyzing ChIP-seq/-exo sequences using a custom motif discovery pipeline, and externally, motifs taken directly from other publications and/or resources.

2. For TRANSFAC, the annotation of Xie et al. is used. For comparing to the JASPAR CORE matrices, the Pearson Correlation Coefficient (PCC) is used to define matrix similarity.

3. All PHYLOFACTS matrices are scanned against the TRANSFAC (public version) motif that has the best similarity score when compared to this model. Only hits with a similarity score over 0.8 are considered.

4. The motif conservation score (MCS) is calculated. Only motifs with an MCS>6 are retained.

5. The count matrices for these motifs are extracted from the downloaded alignments. They are further annotated according to their resemblance with TRANSFAC and JASPAR CORE motifs.

6. The database contains 292 motifs, and so the entire procedure yields a total of 291,000 motif P values. The uniformity of these P values is then measured.

7. The motifs are then used to define i-cisTarget regulatory regions. There is no threshold for comparing the best score in input regions vs. background regions.

8. The motifs are validated using the source article (always Nature 434, 338-345 (2005)).

Please note that the information is incomplete and may require further research or context to fully understand the validation process.

--------------

**ChIP-Seq Analysis Case 3.3: Functional Enrichment**

| | |
|---|---|
| **User's Inputs** | You are a bioinformatician who specializes in writing code and performing analysis for a variety of bioinformatic data analysis tasks. Now I provide you with an Ubuntu 18.04 system where I save the following data, each line including the data path and data description.<br><br>SRR620204.fastq.gz: chip-seq data for Ring1B<br>SRR620205.fastq.gz: chip-seq data for cbx7<br>SRR620206.fastq.gz: chip-seq data for SUZ12<br>SRR620208.fastq.gz: chip-seq data for IgGold<br>mm39.ncbiRefSeq.gtf: genome annotations mouse<br>mm39.fa: mouse genome<br><br>I need you to help me plan what needs to be done in each step of the analysis process, and help me write the code for each step to install softwares, use tools and accomplish the following main goal:<br><br>Perform functional enrichment for protein Ring1B, use protein IgGold as the control |
| **Generated Plans by AutoGPT** | ## Step 1<br><br>### Action executed:<br>Agent message<br>Based on the goal, here is the research plan:<br><br>1. **Understand the Goal**: The main goal is to perform functional enrichment for protein Ring1B, using protein IgGold as the control. The data provided includes chip-seq data for Ring1B and IgGold, genome annotations, and the mouse genome.<br><br>2. **Identify Necessary Tools and Software**: Research the best bioinformatics tools and software for chip-seq data analysis and functional enrichment. This may include tools for quality control, adapter trimming, read alignment, peak calling, and functional enrichment analysis.<br><br>3. **Plan the Analysis Process**: Outline the steps needed to achieve the goal. This will likely include preprocessing the chip-seq data, performing functional enrichment analysis, and comparing the results for Ring1B and IgGold.<br><br>4. **Write the Code**: Once the necessary tools and analysis steps have been identified, write the code for each step. This will include code to install the necessary software, preprocess the data, perform the analysis, and compare the results.<br><br>5. **Verify the Results**: After the analysis is complete, verify that the results are accurate and complete. If any data is missing or the results are not as expected, additional research and analysis may be needed.<br><br>6. **Format the Results**: Ensure that the results are presented in a clear and understandable format. This may include providing a brief description of |

| | |
|---|---|
| | each step, clearly labeling the data, and organizing the code in a logical and readable manner.<br><br>The above function result was too large, so it was stored in the variable "${plan_webResearch_1}".<br>### Prediction:<br>-> The best next step for the assistant would be to provide a plan outlining the steps needed to accomplish the main goal of performing functional enrichment for protein Ring1B using protein IgGold as the control. The plan should include the following steps:<br><br>1. Install the necessary software and tools for the analysis process.<br>2. Download and prepare the required data files, including the chip-seq data for Ring1B (SRR620204.fastq.gz) and IgGold (SRR620208.fastq.gz), as well as the genome annotations (mm39.ncbiRefSeq.gtf) and mouse genome (mm39.fa).<br>3. Perform quality control on the chip-seq data to ensure data integrity and reliability.<br>4. Map the chip-seq data to the mouse genome using a suitable alignment tool.<br>5. Perform peak calling to identify the enriched regions in the chip-seq data for Ring1B and IgGold.<br>6. Perform differential peak analysis to compare the enriched regions between Ring1B and IgGold.<br>7. Perform functional enrichment analysis on the differentially enriched regions using a suitable tool or database.<br>8. Analyze and interpret the results of the functional enrichment analysis.<br><br>The assistant should also provide the code snippets for each step to guide the user in implementing the analysis process. |

**Bisulfite-Seq data analysis** **Case 4.1: Identifying DNA methylation**

| | |
|---|---|
| **User's Inputs** | You are a bioinformatician who specializes in writing code and performing analysis for a variety of bioinformatic data analysis tasks. Now I provide you with an Ubuntu 18.04 system where I save the following data, each line including the data path and data description.<br><br>Sample_R1.fastq.gz: Raw sequencing reads from the bisulfite-converted DNA, typically paired-end reads. These reads contain information about the converted DNA, where unmethylated cytosines are converted to uracils (T) during library preparation.<br>Sample_R2.fastq.gz: Paired-end reads that complement the R1 reads. These are typically used for quality control and paired-end analysis.<br>TruSeq3-PE.fa: Adapter sequences for trimming<br>Reference.fasta: Reference genome for the organism under study in FASTA format, include bisulfite conversion.<br><br>I need you to help me plan what needs to be done in each step of the analysis process, and help me write the code for each step to install softwares, use tools and accomplish the following main goal:<br><br>To identify DNA methylation patterns in the genome based on the Bisulfite-Seq data. |
| **Generated Plans by AutoGPT** | ## Step 1<br><br>### Action executed:<br>Web search for 'how to install FastQC on Ubuntu 18.04'<br>### Function Call:<br>```javascript<br>web_search({<br>"queries": [<br>"how to install FastQC on Ubuntu 18.04"<br>]<br>})<br>```<br>### Result<br>```<br>Found the following result for the web search: 'how to install FastQC on Ubuntu 18.04'<br>--------------<br>To install FastQC on Ubuntu 18.04, follow these steps:<br><br>1. Install JAVA if you don't have it already:<br>```<br>sudo apt install default-jre<br>```<br><br>2. Download FastQC:<br>```<br>mkdir -p ~/Downloads/bin<br>cd ~/Downloads/bin |

  wget
https://www.bioinformatics.babraham.ac.uk/projects/fastqc/fastqc_v0.11.8.zi
p
  unzip fastqc_v0.11.8.zip
  rm fastqc_v0.11.8.zip
  ```

3. Make sure that FastQC is executable:
  ```
  sudo chmod 770 FastQC/fastqc
  ```

Alternatively, you can install FastQC using the apt package manager:

1. Update your package list:
  ```
  sudo apt update
  ```

2. Install FastQC:
  ```
  sudo apt install fastqc
  ```

Please note that the version of FastQC installed via the apt package manager may not be the latest version. If you need a specific version, it's recommended to download it directly from the FastQC website.

```
## Step 2

### Action executed:
Web search for 'how to install Bismark on Ubuntu 18.04'
### Function Call:
```javascript
web_search({
  "queries": [
    "how to install Bismark on Ubuntu 18.04"
  ]
})
```
Result
```
Found the following result for the web search: 'how to install Bismark on Ubuntu 18.04'
--------------
To install Bismark on Ubuntu 18.04, you can follow these steps:

1. Download the Bismark image which is set up with Ubuntu 18.04, samtools v1.9.0, bowtie v2.3.5, and bismark v0.21.0.
```

2. Run Bismark using the following command:
```
$ docker run --rm -v $(pwd):/home/in -w /in chrishah/bismark:v0.21.0 b
```
This command will mount the directory `/in` of the container to the current working directory on your local machine, and allow you to access files in this directory and any sub-directories.

Alternatively, you can install Bismark by following these steps:

1. Download the Bismark tar file (bismark_v0.X.Y.tar.gz).

2. Copy the tar file into a Bismark installation folder.

3. Extract all files by typing:
```
tar xzf bismark_v0.X.Y.tar.gz
```
4. Place the extracted folder (Bismark-0.22.3) in a folder named Bismark at /usr/bin.

5. Run the command:
```
bismark_genome_preparation --path_to_aligner /usr/bin/bowtie2/ --verbose /data/genomes/homo_sapiens/GRCh38/
```
Note: Replace `/full/path/to/` with the actual path to the Bismark installation.

Please note that Perl and bowtie2 need to be installed and located at /usr/bin/ for Bismark to work.

You can also install Bismark using conda with the following command:
```
conda install -c bioconda bismark
```

Please note that the information provided might be incomplete and additional steps might be required.

```
## Step 3

### Action executed:
Web search for 'how to install Samtools on Ubuntu 18.04'
### Function Call:
```javascript
web_search({
  "queries": [
    "how to install Samtools on Ubuntu 18.04"
  ]
})

```
Result
```
Found the following result for the web search: 'how to install Samtools on Ubuntu 18.04'
--------------
To install Samtools on Ubuntu 18.04, follow these steps:

1. Update your package list: `sudo apt-get update -y`
2. Install the Samtools package: `sudo apt install samtools`

Alternatively, you can use `aptitude` to install Samtools:

1. Update your apt database: `sudo aptitude update`
2. Install the Samtools package: `sudo aptitude -y install samtools`

If you want to compile Samtools, you can do so with the following commands:

1. Navigate to your Downloads directory: `cd ~/Downloads`
2. Download the Samtools source code: `wget https://vorboss.dl.sourceforge.net/project/samtools/samtools/0.1.19/samtools-0.1.19.tar.bz2`
3. Extract the downloaded file: `tar -xf samtools-0.1.19.tar.bz2`
4. Navigate to the extracted directory: `cd samtools-0.1.19`
5. Compile the source code: `make`

Please note that the version of Samtools available in the Ubuntu 18.04 repositories is 1.7-1. If you specifically need version 0.1.19, you will need to compile it from source as described above.

--------------

```
Step 4

Action executed:
Web search for 'how to install BEDTools on Ubuntu 18.04'
Function Call:
```javascript
web_search({
  "queries": [
    "how to install BEDTools on Ubuntu 18.04"
  ]
})
```
Result
```
Found the following result for the web search: 'how to install BEDTools on Ubuntu 18.04'
--------------
To install BEDTools on Ubuntu 18.04, follow these steps:

1. Update the apt database by running the following command in the terminal: `sudo apt update`
2. Install bedtools by running the following command: `sudo apt install bedtools`

--------------

```
Step 5

Action executed:
Web search for 'how to use FastQC for quality control of sequencing reads'
Function Call:
```javascript
web_search({
  "queries": [
    "how to use FastQC for quality control of sequencing reads"
  ]
})
```
Result
```
Found the following result for the web search: 'how to use FastQC for quality control of sequencing reads'
--------------
To use FastQC for quality control of sequencing reads, follow these steps:

1. Import data from BAM, SAM, or FastQ files (any variant).
2. FastQC provides a quick overview to tell you in which areas there may be problems.
3. It provides summary graphs and tables to quickly assess your data.
4. FastQC exports results to an HTML-based report for easy viewing and sharing.
5. To generate a report about the quality of sequencing reads, type the program's name in the terminal followed by the flag -h. This will display the instructions for using the program.
6. FastQC can be used to investigate the quality of your sequences using a nice graphical summary output.

Please note that FastQC requires a suitable Java Runtime Environment and the Picard BAM/SAM Libraries to function. The information on how to specify decimal precision in FastQC is not provided in the given text.

--------------

```
Step 6

Action executed:
Web search for 'how to use Bismark for aligning bisulfite-converted reads to the reference genome'
Function Call:
```javascript

web_search({
  "queries": [
    "how to use Bismark for aligning bisulfite-converted reads to the reference genome"
  ]
})
```
Result
```
Found the following result for the web search: 'how to use Bismark for aligning bisulfite-converted reads to the reference genome'
--------------
To use Bismark for aligning bisulfite-converted reads to the reference genome, follow these steps:

1. Sequence reads are first transformed into fully bisulfite-converted forward (C->T) and reverse read (G->A conversion of the forward strand) versions.

2. These transformed reads are then aligned to similarly converted versions of the genome (also C->T and G->A converted).

3. Bismark runs four alignment processes against the bisulfite genomes in parallel to find a unique best alignment.

4. The aligned sequence reads are then compared to the normal genomic sequence to determine the methylation state of all cytosine positions.

5. Bismark uses four parallel instances of the short read aligner Bowtie for the alignment process.

6. Bismark can handle BS-Seq data from both directional and non-directional libraries.

7. The output of Bismark can be easily imported into a genome viewer, such as SeqMonk, for further analysis.

Note: The information provided does not specify any decimal precision requirements in the process of using Bismark for aligning bisulfite-converted reads to the reference genome.

--------------

```
Step 7

Action executed:
Web search for 'how to use Bismark Methylation Extractor to extract DNA methylation information'
Function Call:
```javascript
web_search({
  "queries": [

"how to use Bismark Methylation Extractor to extract DNA methylation information"
  ]
})
```
Result
```
Found the following result for the web search: 'how to use Bismark Methylation Extractor to extract DNA methylation information'
--------------
To use Bismark Methylation Extractor to extract DNA methylation information, follow these steps:

1. Ensure the script from the methylation extractor (which is now included in the Bismark package) resides in the same folder as the 'bismark_methylation_extractor' itself.

2. The methylation extractor automatically detects whether Bismark alignment file(s) were run in single-end or paired-end mode. This automatic detection can be overridden by manually specifying -s or -p. This option is only available for SAM/BAM files.

3. When run in stand-alone mode, the coverage file will replace 'bedGraph' as the file ending with 'bismark.cov'. If the output filename is anything other than 'bedGraph', '.bismark.cov' will be appended to the filename.

4. The final step in the processing pipeline uses Bismark to perform methylation extraction. The principle is straightforward: assign a methylated call when a cytosine is observed at a position showing a cytosine in the reference genome, and assign an unmethylated call when a thymine is observed at a position showing a cytosine in the reference genome.

5. After alignment with the Bismark aligner, call methylation sites using the Bismark methylation extractor.

Note: The efficiency of cytosine (bisulfite) conversion in the sample is estimated to be 99.8%.

The information is incomplete as it does not provide detailed step-by-step instructions or command-line examples for using the Bismark Methylation Extractor.

--------------

```
Step 8

Action executed:
Web search for 'how to use Samtools and BEDTools to analyze DNA methylation patterns'
Function Call:
```javascript
web_search({

  "queries": [
    "how to use Samtools and BEDTools to analyze DNA methylation patterns"
  ]
})
```
Result
```
Found the following result for the web search: 'how to use Samtools and BEDTools to analyze DNA methylation patterns'
--------------
To analyze DNA methylation patterns using Samtools and BEDTools, follow these steps:

1. Ensure a high quality of the sequencing reads to get a good alignment and correct methylation scores. Prior to the alignment step, incorrectly converted reads should be discarded and reads with adapter sequences must be found and adapters trimmed.

2. Use Samtools and BEDTools to extract information in BED format. These BED files are supplied to MEDIPS that estimates the reproducibility of the genome-wide DNA methylation profile with respect to the total number of given short reads and to the size of the reference genome. MEDIPS also analyzes the coverage of genome-wide DNA sequence patterns (e.g. CpGs) by the given reads, and calculates a CpG enrichment factor as a quality control for the immunoprecipitation.

3. Use the following commands to perform these tasks:
   ```
   cd /share/workshop/epigenetics_workshop/$USER/chipseq_example/HTS_testing
   module load samtools
   samtools view -F 2048 -f 2 -q 30 -b JLDY037E.streamed.bam | samtools sort -o JLDY037E.streamed_filtered.bam
   samtools flagstat JLDY037E.streamed.bam
   samtools flagstat JLDY037E.streamed_filtered.bam
   ```

4. Use BEDTools to study genome methylation. BEDTools consists of a set of sub-commands (tools) that are invoked as follows from the terminal of InsideDNA platform:
   ```
   idna_submit.py [wrapper parameters] ‘idna_[tool name] [bedtools options]’
   ```
   One of the main tools available in the BEDTools suite is intersectBed, which returns overlapping features between two BED files.

Note: The information provided is incomplete and may require additional steps or parameters for a complete analysis.

```
## Step 9

### Action executed:
Web search for 'how to interpret DNA methylation patterns'
### Function Call:
```javascript
web_search({
  "queries": [
    "how to interpret DNA methylation patterns"
  ]
})
```
Result
```
Found the following result for the web search: 'how to interpret DNA methylation patterns'
--------------
1. DNA methylation sequencing data analysis involves basic principles and steps that have been used for mammalian genomes. Computational pipelines are used to analyze such data.

2. Interpretation of DNA methylation and hydroxymethylation results depends highly on the experimental setup. Computational tools can be helpful to explore the results. DMPs and DMRs are usually being associated with genes, based on their location relative to gene promoter or body.

3. To interpret DNA methylation, data must be processed, visualized, and statistically analyzed.

4. DNA methylation levels, general profiling, the identification of DMRs and the visualization of the methylome are part of the interpretation process. Array-based data, such as that from Illumina’s HM450K, are fluorescence intensities that quantify the relative abundance of methylated and unmethylated loci.

5. Profiling approaches vary in many aspects, such as DNA input, resolution, genomic region coverage, and bioinformatics analysis. Knowledge of these methods is required for selection of a feasible method.

6. Genome-wide methylation level are calculated and plotted, and DMRs are determined. To perform an integrative analysis, DNA methylation data are coupled with gene expression, e.g., differentially expressed genes (DEGs), to delineate the regulatory role of DNA methylation.

7. The bisulfite sequencing data are generally processed with several steps, including adapter trimming.

8. The advent of next-generation DNA sequencing has provided powerful tools to examine the genome-wide DNA methylation pattern with single-nucleotide resolution.

| | |
|---|---|
| | (Note: The information provided does not include any specific decimal precision as requested. The text does not provide numerical data or statistics that can be represented with decimal precision.)<br><br>-------------- |

**ATAC-seq data analysis** **Case 5.1: Identifying open chromatin regions**

<table>
<tr><td>User's Inputs</td><td>You are a bioinformatician who specializes in writing code and performing analysis for a variety of bioinformatic data analysis tasks. Now I provide you with an Ubuntu 18.04 system where I save the following data, each line including the data path and data description.<br><br>Sample_R1.fastq.gz: Raw sequencing reads from the ATAC-seq experiment. These are typically paired-end reads representing fragments of open chromatin regions.<br>Sample_R2.fastq.gz: Paired-end reads that complement the R1 reads.<br>TruSeq3-PE.fa: Adapter sequences for trimming<br>Reference.fasta: Reference genome for the organism under study in FASTA format. It matches the genome used for the ATAC-seq library preparation.<br><br>I need you to help me plan what needs to be done in each step of the analysis process, and help me write the code for each step to install softwares, use tools and accomplish the following main goal:<br><br>To identify open chromatin regions within the genome based on the ATAC-seq data.</td></tr>
<tr><td>Generated Plans by AutoGPT</td><td>## Step 1<br><br>### Action executed:<br>Agent message<br>Based on the goal, here is the plan for the bioinformatic data analysis:<br><br>**Step 1: Install Required Software**<br>- Install Ubuntu 18.04 system on the computer.<br>- Install necessary software packages for bioinformatic analysis, such as:<br>  - FastQC: Quality control of sequencing reads.<br>  - Trimmomatic: Trimming adapter sequences from reads.<br>  - Bowtie2: Aligning reads to the reference genome.<br>  - MACS2: Identifying open chromatin regions.<br><br>**Step 2: Download and Organize Data**<br>- Download the required data files:<br>  - Sample_R1.fastq.gz<br>  - Sample_R2.fastq.gz<br>  - TruSeq3-PE.fa<br>  - Reference.fasta<br>- Create a project directory and organize the data files within it.<br><br>**Step 3: Quality Control of Sequencing Reads**<br>- Run FastQC on Sample_R1.fastq.gz and Sample_R2.fastq.gz to assess the quality of the reads.<br>- Analyze the FastQC reports to identify any potential issues with the sequencing data.<br><br>**Step 4: Trimming Adapter Sequences**<br>- Use Trimmomatic to remove adapter sequences from the reads.<br>- Provide the path to TruSeq3-PE.fa as the adapter file.</td></tr>
</table>

| | |
|---|---|
| | - Trim both Sample_R1.fastq.gz and Sample_R2.fastq.gz.<br><br>**Step 5: Align Reads to the Reference Genome**<br>- Use Bowtie2 to align the trimmed reads to the Reference.fasta genome.<br>- Generate a sorted and indexed BAM file as the output.<br><br>**Step 6: Identify Open Chromatin Regions**<br>- Use MACS2 to identify open chromatin regions based on the aligned reads.<br>- Provide the sorted and indexed BAM file as input.<br>- Adjust MACS2 parameters as needed for peak calling.<br><br>**Step 7: Analyze and Interpret Results**<br>- Analyze the output files from MACS2 to identify significant open chromatin regions.<br>- Visualize the identified regions using genome browsers or other visualization tools.<br>- Perform downstream analysis or integrate the results with other datasets if required.<br><br>**Step 8: Documentation and Reporting**<br>- Document the analysis steps, including software versions and parameters used.<br>- Prepare a report summarizing the findings and methodology.<br>- Include any code snippets or scripts used during the analysis.<br><br>Note: The above steps provide a general outline for the analysis process. The specific commands and code required may vary depending on the software versions, data format, and specific analysis requirements.<br>### Prediction:<br>-> The best next step for the assistant would be to ask the user for more specific information about the analysis process and the desired output. This will help the assistant understand the user's requirements and provide more accurate guidance and code for each step of the analysis. |

**DNase-seq data analysis** **Case 6.1: Identifying DnaseI hypersensitive site**

| | |
|---|---|
| **User's Inputs** | You are a bioinformatician who specializes in writing code and performing analysis for a variety of bioinformatic data analysis tasks. Now I provide you with an Ubuntu 18.04 system where I save the following data, each line including the data path and data description.<br><br>Sample_R1.fastq.gz: Raw sequencing reads from the DNase-seq experiment.<br>Sample_R2.fastq.gz: Paired-end reads that complement the R1 reads, often used for quality control and paired-end analysis.<br>TruSeq3-PE.fa: Adapter sequences for trimming.<br>Reference.fasta: Reference genome for the organism under study in FASTA format<br><br>I need you to help me plan what needs to be done in each step of the analysis process, and help me write the code for each step to install softwares, use tools and accomplish the following main goal:<br><br>To identify DNase I hypersensitive sites (regions of open chromatin) within the genome based on the DNase-seq data. |
| **Generated Plans by AutoGPT** | ## Step 1<br><br>### Action executed:<br>Agent message<br>Based on the goal, here is the plan for the bioinformatic data analysis:<br><br>**Step 1: Install Required Software**<br>- Install Ubuntu 18.04 system on the computer.<br>- Install necessary software packages for bioinformatic analysis, such as:<br>- FastQC: Quality control of sequencing reads.<br>- Trimmomatic: Trimming adapter sequences.<br>- Bowtie2: Aligning reads to the reference genome.<br>- MACS2: Identifying DNase I hypersensitive sites.<br><br>**Step 2: Download and Organize Data**<br>- Download the required data files:<br>- Sample_R1.fastq.gz: Raw sequencing reads from the DNase-seq experiment.<br>- Sample_R2.fastq.gz: Paired-end reads for quality control and analysis.<br>- TruSeq3-PE.fa: Adapter sequences for trimming.<br>- Reference.fasta: Reference genome in FASTA format.<br>- Create a project directory and organize the data files within it.<br><br>**Step 3: Quality Control and Trimming**<br>- Run FastQC on Sample_R1.fastq.gz and Sample_R2.fastq.gz to assess read quality.<br>- Use Trimmomatic to trim adapter sequences from the reads:<br>- Input: Sample_R1.fastq.gz and Sample_R2.fastq.gz<br>- Output: Trimmed_R1.fastq.gz and Trimmed_R2.fastq.gz<br><br>**Step 4: Read Alignment**<br>- Index the reference genome (Reference.fasta) using Bowtie2. |

- Align the trimmed reads (Trimmed_R1.fastq.gz and Trimmed_R2.fastq.gz) to the reference genome using Bowtie2:
  - Output: Aligned reads in SAM/BAM format.

**Step 5: Identifying DNase I Hypersensitive Sites**
- Use MACS2 to identify DNase I hypersensitive sites based on the aligned reads:
  - Input: Aligned reads in BAM format.
  - Output: DNase I hypersensitive sites in BED format.

**Step 6: Code Implementation**
- Write code to automate the above steps using appropriate programming language (e.g., Python, Bash).
- Use command-line tools and libraries to execute the necessary commands and perform the analysis.
- Ensure proper error handling and logging for each step.

Note: This plan provides a high-level overview of the analysis process. Detailed code implementation may vary depending on specific requirements, software versions, and data characteristics.

**Formatting Requirements:**
1. Data Description: Each line should include the data path and a brief description of the data. The format for each line should be as follows:
   - [Data Path]: [Data Description]
2. Code Steps: The code steps should be clearly outlined and organized. Each step should be numbered or labeled, and the code for each step should be provided.
3. Software Installation: If any software needs to be installed, it should be clearly mentioned. The instructions for installing the software should be provided in a clear and concise manner.
4. Tool Usage: If any specific tools need to be used for the analysis, they should be mentioned. The code for using each tool should be provided, along with any necessary parameters or options.
5. Main Goal: The main goal of the analysis should be clearly stated. In this case, it is to identify DNase I hypersensitive sites based on the DNase-seq data.

Overall, the formatting should be clear, organized, and easy to follow, ensuring that the user can understand and replicate the analysis process.

**4C-seq data analysis   Case 7.1: Find genomics interactions**

| | |
|---|---|
| **User's Inputs** | You are a bioinformatician who specializes in writing code and performing analysis for a variety of bioinformatic data analysis tasks. Now I provide you with an Ubuntu 18.04 system where I save the following data, each line including the data path and data description.<br><br>sample_4C-seq_data.fastq: Raw sequencing reads or aligned data from the 4C-seq experiment, depending on the starting point.<br>viewpoint_coordinates.bed: A file specifying the genomic coordinates of the viewpoint we want to study.<br>reference_genome.fasta: A reference genome in FASTA format for the organism under study. This should match the genome used for the 4C-seq library preparation.<br><br>I need you to help me plan what needs to be done in each step of the analysis process, and help me write the code for each step to install softwares, use tools and accomplish the following main goal:<br><br>To identify genomic interactions originating from a specific genomic viewpoint using 4C-seq data. |
| **Generated Plans by AutoGPT** | ## Step 1<br><br>### Action executed:<br>Agent message<br>Based on your requirements, here is a plan for the analysis process and the code for each step:<br><br>1. **Install Required Software:**<br>- Install Ubuntu 18.04 system on your machine.<br>- Install necessary bioinformatics software packages such as Bowtie, SAMtools, BEDtools, and R.<br><br>```bash<br>sudo apt-get update<br>sudo apt-get install bowtie samtools bedtools r-base<br>```<br><br>2. **Prepare the Data:**<br>- Download or transfer the following data files to your Ubuntu system:<br>- sample_4C-seq_data.fastq: Raw sequencing reads or aligned data from the 4C-seq experiment.<br>- viewpoint_coordinates.bed: A file specifying the genomic coordinates of the viewpoint we want to study.<br>- reference_genome.fasta: A reference genome in FASTA format for the organism under study.<br><br>3. **Quality Control and Preprocessing:**<br>- Perform quality control checks on the 4C-seq data using tools like FastQC.<br>- Trim or filter the reads if necessary to remove low-quality or adapter sequences.<br>- Align the reads to the reference genome using Bowtie or a similar alignment tool. |

| | |
|---|---|
| | - Use SAMtools to convert the alignment file to BAM format and sort it. |

```bash
fastqc sample_4C-seq_data.fastq
bowtie2 -x reference_genome -U sample_4C-seq_data.fastq -S aligned_reads.sam
samtools view -S -b aligned_reads.sam > aligned_reads.bam
samtools sort aligned_reads.bam -o sorted_reads.bam
```

4. **Identify Genomic Interactions:**
   - Use BEDtools to extract reads that overlap with the genomic coordinates specified in the viewpoint_coordinates.bed file.
   - Perform peak calling or identify significant interactions using tools like HiCExplorer or CHiCAGO.
   - Analyze and visualize the identified interactions using R or other plotting libraries.

```bash
bedtools intersect -a sorted_reads.bam -b viewpoint_coordinates.bed > intersected_reads.bam
```

5. **Interpret and Validate Results:**
   - Analyze the identified interactions to understand their biological significance.
   - Validate the results using experimental techniques such as PCR or ChIP-qPCR.
   - Perform downstream analysis or integrate the results with other genomic datasets if required.

Please note that the above steps provide a general outline of the analysis process. The specific tools and parameters may vary depending on your specific requirements and the nature of your data.

Nanopore DNA sequencing data analysis   Case 8.1: Genome assembly

<table>
<tr><td>User's Inputs</td><td>You are a bioinformatician who specializes in writing code and performing analysis for a variety of bioinformatic data analysis tasks. Now I provide you with an Ubuntu 18.04 system where I save the following data, each line including the data path and data description.

sample_nanopore_reads.fastq: Raw Nanopore sequencing data in fastq format.
eference_genome.fasta: A reference genome in FASTA format, if a closely related reference is available and used as a guide during the assembly

I need you to help me plan what needs to be done in each step of the analysis process, and help me write the code for each step to install softwares, use tools and accomplish the following main goal:

To assemble a genome from the Nanopore sequencing data.</td></tr>
<tr><td>Generated Plans by AutoGPT</td><td>## Step 1

Action executed:
Agent message
Based on the goal, here is the plan for the analysis process and the code for each step:

1. **Preprocessing the raw Nanopore sequencing data**: This involves quality control, trimming adapters, and filtering out low-quality reads. We can use software like Porechop or Poretools for this step.

Code for preprocessing with Porechop:
```
Install Porechop
pip install porechop

Preprocess the raw Nanopore sequencing data
porechop -i sample_nanopore_reads.fastq -o preprocessed_reads.fastq
```

2. **Aligning the preprocessed reads to the reference genome**: This can be done using tools like minimap2 or BWA-MEM.

Code for alignment with minimap2:
```
Install minimap2
git clone https://github.com/lh3/minimap2
cd minimap2
make

Align the preprocessed reads to the reference genome
./minimap2 -ax map-ont reference_genome.fasta
preprocessed_reads.fastq > aligned_reads.sam
```

3. **Assembling the genome**: Once the reads are aligned, we can use a genome assembler like Canu, Flye, or Unicycler to assemble the genome.</td></tr>
</table>

These assemblers utilize the aligned reads and the reference genome (if available) to generate a consensus sequence.

Code for genome assembly with Canu:
```
# Install Canu
git clone https://github.com/marbl/canu
cd canu/src
make -j

# Assemble the genome
./canu -p assembly -d assembly_dir genomeSize=5m -nanopore-raw
aligned_reads.sam
```

4. **Evaluating the quality of the assembled genome**: Tools like QUAST or BUSCO can be used to evaluate the completeness and accuracy of the assembly.

Code for evaluation with QUAST:
```
# Install QUAST
pip install quast

# Evaluate the quality of the assembly
quast.py assembly_dir/assembly.contigs.fasta
```

5. **Annotating the assembled genome**: The assembled genome can be annotated using tools like Prokka or MAKER to identify genes, regulatory elements, and other genomic features.

Code for annotation with Prokka:
```
# Install Prokka
conda install -c conda-forge -c bioconda prokka

# Annotate the assembled genome
prokka --outdir annotation --prefix genome_annotation
assembly_dir/assembly.contigs.fasta
```

Please note that you may need to adjust the commands based on your specific setup and requirements.

Nanopore DNA sequencing data analysis Case 8.2: Tandem repeats variation identification

<table>
<tr><td>User's Inputs</td><td>You are a bioinformatician who specializes in writing code and performing analysis for a variety of bioinformatic data analysis tasks. Now I provide you with an Ubuntu 18.04 system where I save the following data, each line including the data path and data description.

sample_nanopore_reads.fastq: Raw Nanopore sequencing data in fastq format.
eference_genome.fasta: A reference genome in FASTA format, if a closely related reference is available and used as a guide during the assembly

I need you to help me plan what needs to be done in each step of the analysis process, and help me write the code for each step to install softwares, use tools and accomplish the following main goal:

To identify tandem repeats.</td></tr>
<tr><td>Generated Plans by AutoGPT</td><td>## Step 1

Action executed:
Agent message
Based on the goal, here is the plan for the bioinformatic data analysis:

1. **Install Required Software**: Check if the necessary software is already installed on the Ubuntu 18.04 system. If not, install the following software:
- Nanopore sequencing data analysis software (e.g., MinKNOW, Guppy)
- Genome assembly software (e.g., Canu, Flye)
- Tandem repeat identification software (e.g., TRF, Tandem Repeats Finder)

2. **Preprocess Raw Nanopore Sequencing Data**: Use the installed Nanopore sequencing data analysis software to preprocess the raw fastq data (sample_nanopore_reads.fastq). Perform quality control and filtering to remove low-quality reads, adapters, and contaminants. Generate cleaned and trimmed fastq files for downstream analysis.

3. **Genome Assembly**: Use the cleaned and trimmed fastq files from Step 2 for genome assembly. Utilize the installed genome assembly software (e.g., Canu, Flye) to assemble the genome. Provide the reference genome (reference_genome.fasta) as a guide during the assembly process if available.

4. **Tandem Repeat Identification**: Once the genome assembly is completed, use the installed tandem repeat identification software (e.g., TRF, Tandem Repeats Finder) to identify tandem repeats in the assembled genome. Adjust the parameters of the software according to the specific requirements of the analysis. Extract the identified tandem repeat sequences and their locations in the genome.

5. **Code Implementation**: Write code to automate the above steps and perform the necessary installations, data preprocessing, genome assembly, and tandem repeat identification. Utilize programming languages such as Python or Bash scripting to write the code. Ensure the code is well-documented, modular, and efficient.</td></tr>
</table>

6. **Result Analysis and Visualization**: Analyze the identified tandem repeat sequences and their locations in the assembled genome. Perform statistical analysis if required to understand the distribution and characteristics of the tandem repeats. Visualize the results using appropriate bioinformatics tools or libraries (e.g., matplotlib, ggplot) to generate plots, graphs, or visual representations.

7. **Documentation and Reporting**: Document the entire analysis process, including the steps performed, software used, parameters, and code implementation details. Prepare a comprehensive report summarizing the findings, including the identified tandem repeats, their characteristics, and any relevant insights. Include the code developed in the report or as a separate file for reproducibility.

Note: The specific software and tools mentioned in the plan are just examples. You may choose alternative software based on your expertise and the specific requirements of the analysis.

As for the formatting requirements:

1. **Data Path and Description**: Each line should include the data path and a brief description of the data. For example:
   - `sample_nanopore_reads.fastq`: Raw Nanopore sequencing data in fastq format.
   - `reference_genome.fasta`: A reference genome in FASTA format.

2. **Code and Software Installation**: The code and software installation steps should be clearly mentioned and formatted separately. For example:
   - Code:
```
# Code for installing required software
sudo apt-get install <software_name>
```

3. **Analysis Process Steps**: Each step of the analysis process should be listed and formatted separately. For example:
   - Step 1: Preprocessing
```
# Code for preprocessing step
python preprocess.py --input <input_file> --output <output_file>
```

4. **Main Goal**: The main goal should be clearly stated and formatted separately. For example:
   - Main Goal: To identify tandem repeats.

By following these formatting requirements, the instructions and data provided will be organized and easy to understand for the bioinformatician.

PacBio DNA sequencing data analysis **Case 9.1: Genome assembly**

**User's Inputs**	You are a bioinformatician who specializes in writing code and performing analysis for a variety of bioinformatic data analysis tasks. Now I provide you with an Ubuntu 18.04 system where I save the following data, each line including the data path and data description.<br><br>sample_pacbio_reads.fastq: Raw PacBio sequencing data in fastq format.<br>eference_genome.fasta: A reference genome in FASTA format, if a closely related reference is available and used as a guide during the assembly<br><br>I need you to help me plan what needs to be done in each step of the analysis process, and help me write the code for each step to install softwares, use tools and accomplish the following main goal:<br><br>To assemble a genome from the PacBio sequencing data.
**Generated Plans by AutoGPT**	-> The best next step for the assistant would be to gather more information about the specific requirements and constraints of the genome assembly task. This includes understanding the desired output, any specific software or tools that need to be used, and any additional data or resources that may be required.<br><br>The assistant can start by asking the user for more details about the PacBio sequencing data, such as the size of the dataset, the expected coverage, and any quality control steps that have already been performed. Additionally, the assistant can inquire about the available computational resources, such as the amount of memory and processing power available.<br><br>Based on the information gathered, the assistant can then provide a detailed plan outlining the necessary steps for genome assembly. This plan may include steps such as data preprocessing, quality control, reference-guided assembly, and post-assembly analysis. The assistant can also provide code snippets or recommendations for specific software or tools that can be used for each step.<br><br>Overall, the assistant should aim to understand the user's requirements and provide a comprehensive plan that addresses all the necessary steps for genome assembly.

RNA-Seq Analysis **Case 10.1: Find differentially expressed genes**

<table>
<tr><td>User's Inputs</td><td>You are a bioinformatician who specializes in writing code and performing analysis for a variety of bioinformatic data analysis tasks. Now I provide you with an Ubuntu 18.04 system where I save the following data, each line including the data path and data description.

SRR1374921.fastq.gz: single-end mouse rna-seq reads, replicate 1 in LoGlu group
SRR1374922.fastq.gz: single-end mouse rna-seq reads, replicate 2 in LoGlu group
SRR1374923.fastq.gz: single-end mouse rna-seq reads, replicate 1 in HiGlu group
SRR1374924.fastq.gz: single-end mouse rna-seq reads, replicate 2 in HiGlu group
TruSeq3-SE.fa: trimming adapter
mm39.fa: mouse mm39 genome fasta
mm39.ncbiRefSeq.gtf: mouse mm39 genome annotation

I need you to help me plan what needs to be done in each step of the analysis process, and help me write the code for each step to install softwares, use tools and accomplish the following main goal:

Find the differentially expressed genes</td></tr>
<tr><td>Generated Plans by AutoGPT</td><td>## Step 1

Action executed:
Agent message
Based on the goal, here is the plan for the analysis process and the code for each step:

1. **Install necessary software and tools**:
- FastQC: For quality control checks on raw sequence data.
- Trimmomatic: For trimming Illumina (FASTQ) data.
- HISAT2: For aligning sequencing reads to a population of human genomes.
- StringTie: For assembling RNA-Seq alignments into potential transcripts.
- DESeq2: For differential gene expression analysis based on the negative binomial distribution.

2. **Preprocess the RNA-seq reads**:
- Use FastQC for quality control checks on raw sequence data.
- Use Trimmomatic with TruSeq3-SE.fa to trim adapters from the RNA-seq reads.

3. **Align the reads to the mouse genome**:
- Use HISAT2 with mm39.fa to align the trimmed reads to the mouse genome.

4. **Quantify gene expression levels**:</td></tr>
</table>

	- Use StringTie with mm39.ncbiRefSeq.gtf to quantify gene expression levels.<br><br>5. **Perform statistical analysis to identify differentially expressed genes**:<br>- Use DESeq2 for statistical analysis to identify differentially expressed genes.<br><br>The code for each step will be provided in the next steps.

RNA-Seq Analysis **Case 10.2: Identify top5 down-regulated genes in HiGlu group**

**User's Inputs**	You are a bioinformatician who specializes in writing code and performing analysis for a variety of bioinformatic data analysis tasks. Now I provide you with an Ubuntu 18.04 system where I save the following data, each line including the data path and data description.<br><br>SRR1374921.fastq.gz: single-end mouse rna-seq reads, replicate 1 in LoGlu group<br>SRR1374922.fastq.gz: single-end mouse rna-seq reads, replicate 2 in LoGlu group<br>SRR1374923.fastq.gz: single-end mouse rna-seq reads, replicate 1 in HiGlu group<br>SRR1374924.fastq.gz: single-end mouse rna-seq reads, replicate 2 in HiGlu group<br>TruSeq3-SE.fa: trimming adapter<br>mm39.fa: mouse mm39 genome fasta<br>mm39.ncbiRefSeq.gtf: mouse mm39 genome annotation<br><br>I need you to help me plan what needs to be done in each step of the analysis process, and help me write the code for each step to install softwares, use tools and accomplish the following main goal:<br><br>Identify top5 down-regulated genes in HiGlu group
**Generated Plans by AutoGPT**	-> The best next step for the assistant would be to start by installing the necessary software and tools required for the analysis process. Based on the provided information, the following software and tools are required:<br><br>1. Trimming adapter: TruSeq3-SE.fa<br>2. Mouse genome fasta: mm39.fa<br>3. Mouse genome annotation: mm39.ncbiRefSeq.gtf<br><br>The assistant should guide the user on how to install these software and tools on their Ubuntu 18.04 system. Additionally, the assistant can provide instructions on how to download and save the provided data files in the appropriate directory.<br><br>Once the software and tools are installed, the assistant can proceed to the next step of the analysis process, which would involve using the installed tools to perform the necessary analysis tasks.

RNA-Seq Analysis   Case 10.3: Predict Fusion genes

**User’s Inputs**	You are a bioinformatician who specializes in writing code and performing analysis for a variety of bioinformatic data analysis tasks. Now I provide you with an Ubuntu 18.04 system where I save the following data, each line including the data path and data description.<br><br>rnaseq_1.fastq.gz: RNA-Seq read 1 data (left read)<br>rnaseq_2.fastq.gz: RNA-Seq read 2 data (right read)<br>CTAT_HumanFusionLib.mini.dat.gz: a small fusion annotation library<br>minigenome.fa: small genome sequence consisting of ~750 genes<br>minigenome.gtf: transcript structure annotations for these genes<br><br>I need you to help me plan what needs to be done in each step of the analysis process, and help me write the code for each step to install softwares, use tools and accomplish the following main goal:<br><br>Predict Fusion genes
**Generated Plans by AutoGPT**	## Step 1<br><br>### Action executed:<br>Web search for 'How to install bioinformatics tools on Ubuntu 18.04'<br>### Function Call:<br>```javascript<br>web_search({<br>"queries": [<br>"How to install bioinformatics tools on Ubuntu 18.04"<br>]<br>})<br>```<br>### Result<br>```<br>Found the following result for the web search: 'How to install bioinformatics tools on Ubuntu 18.04'<br>--------------<br>1. Install RDPTools on Ubuntu: The text does not provide specific steps for this installation.<br><br>2. Install some alignment software on Ubuntu 18.04 & 20.04: The text does not provide specific steps for this installation.<br><br>3. Install samtools, tabix, and bcftools: You can install these straight from apt. The commands are:<br>- sudo apt install samtools<br>- sudo apt install tabix<br>- sudo apt install bcftools<br><br>4. Install bioinformatics tools using conda / bioconda:<br>- Download miniconda using the command: wget https://repo.continuum.io/miniconda/Miniconda3-latest-Linux-x86_64.sh -O ~/miniconda.sh<br>- Install miniconda using the command: bash miniconda.sh -b -p miniconda

- Add miniconda to your PATH using the command: export PATH=$PWD/miniconda/bin:$PATH
- Update conda using the command: conda update

5. Install tools from the PAPPSO Ubuntu Bionic Beaver repository: The text does not provide specific steps for this installation.

6. Install JAVA, fastqc, and trim galore on Ubuntu 18.04:
   - Install JAVA using the command: sudo apt install default-jre
   - Download fastqc using the commands:
     - mkdir -p ~/Downloads/bin
     - cd ~/Downloads/bin
     - wget https://www.bioinformatics.babraham.ac.uk/projects/fastqc/fastqc_v0.11.8.zip
     - unzip fastqc_v0.11.8.zip
     - rm fastqc_v0.11.8.zip
     - Make sure that fastqc is executable using the command: sudo chmod 770 FastQC/fastqc
   - The text does not provide specific steps for downloading trim galore.

7. Install a large number of different bioinformatics tools for a workshop: The text does not provide specific steps for these installations.

```
## Step 2

### Action executed:
Web search for 'How to preprocess RNA-Seq data'
### Function Call:
```javascript
web_search({
  "queries": [
    "How to preprocess RNA-Seq data"
  ]
})
```
Result
```
Found the following result for the web search: 'How to preprocess RNA-Seq data'
--------------
1. Quality Check: The overall quality of the sequence information is checked. This is the first step in preprocessing of raw reads for RNA-Seq data.

2. Removal of Contaminations: Contaminations from other organisms are removed.

3. Quality Control Run: Another quality control run is performed to confirm that the sequence data is now acceptable.

4. Use of Tools: Tools like FastQC are used for sequence data quality control. However, there is no comprehensive tool that covers all preprocessing steps commonly used on RNA-seq data.

5. Preprocessing Steps: The most time demanding step in the analysis of RNA-seq data is preprocessing the raw sequence data, such as running quality control and adapter, contamination and quality trimming.

6. Use of Alignment Files: Some tools only take alignment files (BAM) as input, which renders them inappropriate when working with alignment-free transcript counters such as kallisto and salmon.

7. Tutorial: The Delhomme et al. Epigenesys protocol can be followed for RNA sequencing data preprocessing.

8. Specialized Bioinformatic Expertise: The specialized bioinformatic expertise required to successfully preprocess and analyze RNA-seq data can pose a significant barrier for some research groups. Researchers must find relevant studies, collect the necessary metadata, quantify read mapping, calculate the DEGs, perform Gene Ontology (GO) enrichment, and compute significant signaling pathways.

Note: The information is incomplete as it does not provide detailed steps or methods for each of the preprocessing steps.

--------------

```
Step 3

Action executed:
Web search for 'How to align RNA-Seq reads to the reference genome'
Function Call:
```javascript
web_search({
  "queries": [
    "How to align RNA-Seq reads to the reference genome"
  ]
})
```
Result
```
Found the following result for the web search: 'How to align RNA-Seq reads to the reference genome'
--------------
1. Choose an appropriate reference genome to map the reads against.
2. Use splice-aware alignment tools such as STAR or HISAT2 for the alignment process.
3. With a tool like STAR, you can "project" genomic alignments directly to transcriptomic coordinates.
4. RSEM, Salmon, and eXpress are capable of processing these alignments.

5. Perform spliced read alignment against the genome using STAR.
6. Align the original reads against the assembly using Bowtie2 and quantify with Salmon.
7. Use TopHat for RNA-seq mapping, which aligns reads in two steps: unspliced reads are mapped to locate exons with Bowtie, and unmapped reads are then split and aligned independently to identify exon junctions.
8. Depending on the availability of a genome sequence, you can infer which transcripts are expressed by mapping the reads to the reference genome (genome mapping) or transcriptome (transcriptome mapping).
9. The goal of the read alignment is to map short sequencing reads efficiently to a large reference genome to identify the 'correct' genomic loci from which the read originated whilst taking into account errors in the sequence reads.
10. Two alignment strategies are available: STAR, a gapped RNA-Seq read mapper with support for splice-junction and fusion read detection, and BWA.
11. The process includes quality control of the reads, alignment of the reads to the reference genome, conversion of the files to raw counts, analysis of the counts with DeSeq2, and finally annotation of the reads using Biomart.

--------------

```
Step 4

Action executed:
Web search for 'How to identify fusion genes using fusion annotation library'
Function Call:
```javascript
web_search({
  "queries": [
    "How to identify fusion genes using fusion annotation library"
  ]
})
```
Result
```
Found the following result for the web search: 'How to identify fusion genes using fusion annotation library'
--------------
To identify fusion genes using a fusion annotation library, follow these steps:

1. Use the STAR aligner to map junction reads and spanning reads to a reference annotation set.
2. Run fusion detection by selecting the Chimeric results data node and choosing STAR-Fusion from the Variant analysis menu in the toolbox.
3. Choose the STAR-Fusion annotation from the drop-down list. Automatic downloads of the plug-n-play libraries are provided by Trinity Cancer Transcriptome.
4. The IDP-fusion tool can be used to detect and annotate fusion genes with novel genes and novel exons involved.

5. An analytic tool is needed to find fusion genes (i.e. the pair of involved genes), determine the fusion sites between the paired genes, and identify the expressed isoforms.
6. Validation of targeted RNAseq for fusion gene detection can be done by comparing the abundance of fusion reads from targeted and canonical RNASeq libraries.
7. FusionGDB 2.0 is a database that systematically annotates the function of fusion genes across pan-cancer aided by deep learning approaches. It provides information on Gene Sequence, Fusion Gene PPI analysis, Related Drugs, and Related Diseases.

Please note that the information might be incomplete as it is extracted from different webpages and might lack some context.

--------------

```
Step 5

Action executed:
Web search for 'How to analyze and interpret fusion genes'
Function Call:
```javascript
web_search({
  "queries": [
    "How to analyze and interpret fusion genes"
  ]
})
```
Result
```
Found the following result for the web search: 'How to analyze and interpret fusion genes'
--------------
To analyze and interpret fusion genes, the following steps are required:

1. Find fusion genes: This involves identifying the pair of genes involved in the fusion. This is the foundation of all subsequent downstream research.

2. Determine the fusion sites: This involves identifying the specific locations where the paired genes have fused.

3. Identify the expressed isoforms: This involves identifying the different versions of the gene that are being expressed.

Analytic tools such as Second Generation Sequencing (SGS) and algorithms like deFuse and FusionMap are used to accomplish these steps. These tools are based on alignments of SGS short reads.

In addition to these, the analysis of fusion genes also involves the consideration of ambiguously aligned paired-end reads. Tools like deFuse are capable of identifying gene fusions with boundaries between known exons as well as between intronic or intergenic sequences.

| | |
|---|---|
| | For validation of fusions, several confidence measures estimating the likelihood of the fusion are used. The resulting groups and subgroups are predicted as fusion genes.<br><br>The analysis of fusion genes can also provide insights into the functions of their constituent domains and help in assigning accurate functional annotations.<br><br>Please note that the information provided is incomplete and may require further research for a comprehensive understanding of the process.<br><br>-------------- |

**RNA-Seq Analysis** **Case 10.4: isoform expression**

| | |
|---|---|
| **User's Inputs** | You are a bioinformatician who specializes in writing code and performing analysis for a variety of bioinformatic data analysis tasks. Now I provide you with an Ubuntu 18.04 system where I save the following data, each line including the data path and data description.<br><br>SRR14463651_1.fastq.gz: paired end RNA sequencing data from human lymphoma cell, read 1 of lymphoma samples replicate 1<br>SRR14463651_2.fastq.gz: paired end RNA sequencing data from human lymphoma cell, read 2 of lymphoma samples replicate 1<br>SRR14463652_1.fastq.gz: paired end RNA sequencing data from human lymphoma cell, read 1 of lymphoma samples replicate 2<br>SRR14463652_2.fastq.gz: paired end RNA sequencing data from human lymphoma cell, read 2 of lymphoma samples replicate 2<br>SRR14463680_1.fastq.gz: paired end RNA sequencing data from human lymphoma cell, read 1 of control samples replicate 1<br>SRR14463680_2.fastq.gz: paired end RNA sequencing data from human lymphoma cell, read 2 of control samples replicate 1<br>SRR14463681_1.fastq.gz: paired end RNA sequencing data from human lymphoma cell, read 1 of control samples replicate 2<br>SRR14463681_2.fastq.gz: paired end RNA sequencing data from human lymphoma cell, read 2 of control samples replicate 2<br>gencode.v44.transcripts.fa: transcriptome annotation<br>TruSeq3-PE.fa: trimming adapter<br><br>I need you to help me plan what needs to be done in each step of the analysis process, and help me write the code for each step to install softwares, use tools and accomplish the following main goal:<br><br>quantify RNA isoform expression based on transcriptome annotation |
| **Generated Plans by AutoGPT** | -> The best next step for the assistant would be to gather more information about the specific analysis process and the tools/software required to accomplish the goal of quantifying RNA isoform expression based on the transcriptome annotation. This information is necessary to plan the steps and write the code effectively.<br><br>### Selected agent:<br>-> Researcher<br><br>## Step 1<br><br>### Action executed:<br>Agent message<br>Based on the goal and the data provided, here is the plan for the analysis process:<br><br>1. **Install Required Software**: We need to ensure that the necessary software packages for bioinformatic analysis are installed on the Ubuntu 18.04 system. These include:<br>- FastQC: for quality control of sequencing data.<br>- Trimmomatic: for adapter trimming and quality filtering of sequencing data. |

- HISAT2: for aligning RNA-seq reads to the reference transcriptome.
- StringTie: for quantifying transcript abundance.
- Samtools: for manipulating and analyzing aligned sequencing data.
- R or Python: for further analysis and visualization.

2. **Download and Prepare Data**: The provided data files need to be downloaded to the Ubuntu system. A directory structure should be created to organize the data. The compressed fastq.gz files need to be unzipped using the appropriate command. The integrity and quality of the sequencing data should be verified using FastQC.

3. **Preprocess the Data**: The sequencing data should be preprocessed by trimming adapter sequences and performing quality filtering using Trimmomatic. Quality control checks on the trimmed data should be performed using FastQC.

4. **Align Reads to the Reference Transcriptome**: An index of the reference transcriptome should be built using HISAT2. The trimmed reads should be aligned to the reference transcriptome using HISAT2. The alignment output should be converted to BAM format using Samtools.

5. **Quantify Transcript Abundance**: StringTie should be used to estimate transcript abundance levels based on the aligned reads. A transcript abundance table should be generated for further analysis.

6. **Perform Differential Expression Analysis**: If applicable, the transcript abundance between different conditions (e.g., lymphoma samples vs. control samples) should be compared using statistical methods such as DESeq2 or edgeR. Differentially expressed isoforms and genes should be identified.

7. **Visualization and Interpretation**: R or Python should be used to visualize the results, such as creating plots, heatmaps, or interactive visualizations. The findings should be interpreted and conclusions drawn based on the analysis.

The specific code for each step will depend on the software versions, file paths, and specific analysis requirements. It is recommended to consult the documentation of each software/tool for detailed instructions on installation and usage.
### Prediction:
-> The best next step for the assistant would be to gather more information about the specific analysis process and the tools/software required to accomplish the goal of quantifying RNA isoform expression based on the transcriptome annotation. This information is necessary to plan the steps and write the code effectively.

**RNA-seq Analysis** **Case 10.5: splicing analysis**

<table>
<tr><td>User's Inputs</td><td>You are a bioinformatician who specializes in writing code and performing analysis for a variety of bioinformatic data analysis tasks. Now I provide you with an Ubuntu 18.04 system where I save the following data, each line including the data path and data description.<br><br>SRR14463651_1.fastq.gz: paired end RNA sequencing data from human lymphoma cell, read 1 of lymphoma samples replicate 1<br>SRR14463651_2.fastq.gz: paired end RNA sequencing data from human lymphoma cell, read 2 of lymphoma samples replicate 1<br>SRR14463652_1.fastq.gz: paired end RNA sequencing data from human lymphoma cell, read 1 of lymphoma samples replicate 2<br>SRR14463652_2.fastq.gz: paired end RNA sequencing data from human lymphoma cell, read 2 of lymphoma samples replicate 2<br>SRR14463680_1.fastq.gz: paired end RNA sequencing data from human lymphoma cell, read 1 of control samples replicate 1<br>SRR14463680_2.fastq.gz: paired end RNA sequencing data from human lymphoma cell, read 2 of control samples replicate 1<br>SRR14463681_1.fastq.gz: paired end RNA sequencing data from human lymphoma cell, read 1 of control samples replicate 2<br>SRR14463681_2.fastq.gz: paired end RNA sequencing data from human lymphoma cell, read 2 of control samples replicate 2<br>hg38.fa: reference genome<br>gencode.v38.annotation.gtf: gene annotation<br>TruSeq3-PE.fa: trimming adapter<br><br>I need you to help me plan what needs to be done in each step of the analysis process, and help me write the code for each step to install softwares, use tools and accomplish the following main goal:<br><br>identify alternative splicing events in lymphoma and control samples and compare them to get differentially spliced events</td></tr>
<tr><td>Generated Plans by AutoGPT</td><td>-> The best next step for the assistant would be to gather more information about the specific analysis process and the tools/software required to identify alternative splicing events in the given RNA sequencing data. This can be done by asking the user for more details about the analysis process, such as the specific steps involved, any preferred tools or software, and any specific requirements or considerations. Additionally, the assistant can inquire about the user's familiarity with bioinformatics analysis and their preferred programming language for writing the code.<br><br>### Selected agent:<br>-> Researcher<br><br>## Step 1<br><br>### Action executed:<br>Agent message<br>Based on the goal, here is the plan for the analysis process:</td></tr>
</table>

| | |
|---|---|
| | 1. **Install Required Software and Tools**: Install necessary bioinformatics software and tools such as FastQC, HISAT2, StringTie, and DESeq2 on your Ubuntu 18.04 system.<br><br>2. **Download and Prepare Data**: Download the required data files and organize them in a project directory.<br><br>3. **Quality Control and Preprocessing**: Run FastQC on the raw sequencing data to assess the quality. Trim the adapter sequences and perform quality filtering and trimming of the reads.<br><br>4. **Read Alignment**: Use HISAT2 to align the preprocessed reads to the reference genome (hg38.fa) and generate alignment files for each sample.<br><br>5. **Transcript Assembly and Quantification**: Use StringTie to assemble transcripts and estimate their abundances for each sample, using the gene annotation file to guide the assembly process.<br><br>6. **Differential Splicing Analysis**: Use DESeq2 to perform differential splicing analysis between lymphoma and control samples. Prepare the count matrix from the transcript abundances obtained in the previous step and perform statistical analysis to identify differentially spliced events.<br><br>7. **Interpretation and Visualization**: Analyze and interpret the results obtained from the differential splicing analysis. Visualize the differentially spliced events using tools like R or Python libraries.<br><br>8. **Documentation and Reporting**: Document the analysis steps, including the software versions used and parameter settings. Prepare a report summarizing the findings, including the identified differentially spliced events and their biological implications.<br><br>The code for each step will depend on the specific software and tools you choose to use. It is recommended to refer to the documentation and user guides of the respective software for detailed instructions on how to execute each step.<br><br>For data and code formatting, each line should include the data path and data description. The code for each step should be provided and properly formatted for readability. The main goal of identifying alternative splicing events and comparing them should be clearly stated. The formatting should be clear, organized, and easy to follow. |

**RNA-seq Analysis** **Case 10.6: APA analysis**

<table>
<tr><td>User's Inputs</td><td>You are a bioinformatician who specializes in writing code and performing analysis for a variety of bioinformatic data analysis tasks. Now I provide you with an Ubuntu 18.04 system where I save the following data, each line including the data path and data description.<br><br>SRR14463651_1.fastq.gz: paired end RNA sequencing data from human lymphoma cell, read 1 of lymphoma samples replicate 1<br>SRR14463651_2.fastq.gz: paired end RNA sequencing data from human lymphoma cell, read 2 of lymphoma samples replicate 1<br>SRR14463652_1.fastq.gz: paired end RNA sequencing data from human lymphoma cell, read 1 of lymphoma samples replicate 2<br>SRR14463652_2.fastq.gz: paired end RNA sequencing data from human lymphoma cell, read 2 of lymphoma samples replicate 2<br>SRR14463680_1.fastq.gz: paired end RNA sequencing data from human lymphoma cell, read 1 of control samples replicate 1<br>SRR14463680_2.fastq.gz: paired end RNA sequencing data from human lymphoma cell, read 2 of control samples replicate 1<br>SRR14463681_1.fastq.gz: paired end RNA sequencing data from human lymphoma cell, read 1 of control samples replicate 2<br>SRR14463681_2.fastq.gz: paired end RNA sequencing data from human lymphoma cell, read 2 of control samples replicate 2<br>hg38.fa: reference genome<br>gencode.v38.annotation.gtf: gene annotation<br>TruSeq3-PE.fa: trimming adapter<br><br>I need you to help me plan what needs to be done in each step of the analysis process, and help me write the code for each step to install softwares, use tools and accomplish the following main goal:<br><br>identify and quantify alternative polyadenylation between lymphoma and control samples</td></tr>
<tr><td>Generated Plans by AutoGPT</td><td>## Step 1<br><br>### Action executed:<br>Agent message<br>Based on the goal, here is the plan for the analysis process:<br><br>1. **Install Required Software and Tools**: Install necessary bioinformatics software and tools on the Ubuntu 18.04 system. These include FastQC for quality control of sequencing data, Trimmomatic for trimming adapter sequences, HISAT2 for aligning RNA-seq reads to the reference genome, StringTie for quantifying gene expression levels, and DESeq2 for differential gene expression analysis.<br><br>2. **Download Reference Data**: Download the reference genome (hg38.fa) and gene annotation file (gencode.v38.annotation.gtf) from their respective sources or databases. Store the downloaded files in a specific directory.<br><br>3. **Preprocess the RNA-seq Data**: Use Trimmomatic to remove adapter sequences and low-quality reads from the RNA-seq data. Perform quality control using FastQC to ensure the data quality after preprocessing.</td></tr>
</table>

| | |
|---|---|
| | 4. **Align RNA-seq Reads to the Reference Genome**: Use HISAT2 to align the preprocessed RNA-seq reads to the reference genome. Generate alignment files (SAM/BAM format) for further analysis.<br><br>5. **Quantify Gene Expression Levels**: Use StringTie to estimate transcript abundances and generate expression tables for each sample. Merge the expression tables into a single matrix for downstream analysis.<br><br>6. **Perform Differential Gene Expression Analysis**: Use DESeq2 to identify and quantify alternative polyadenylation between lymphoma and control samples. Perform statistical analysis to identify significantly differentially expressed genes. Generate plots and tables summarizing the results.<br><br>7. **Interpret and Visualize the Results**: Analyze the differential expression results to identify genes with alternative polyadenylation between lymphoma and control samples. Visualize the results using appropriate plots, such as volcano plots or heatmaps. Interpret the findings and draw conclusions based on the analysis.<br><br>The code for each step will depend on the specific software versions and command-line options. It is recommended to refer to the documentation of each software/tool for detailed instructions on how to use them.<br><br>In terms of formatting, each line should include the data path and data description. The data paths should be clearly specified for each file. The data description should provide relevant information about the data, such as the type of sequencing data, sample type, replicate number, etc. The data paths and descriptions should be organized in a clear and readable format, such as using bullet points or numbered lists.<br><br>The code for each step should be provided and properly formatted and indented for readability. Relevant comments should be included to explain the purpose and functionality of the code. If there are multiple code blocks or sections, they should be clearly separated and labeled. The formatting should be organized, clear, and easy to follow, allowing the reader to understand the data and code structure easily. |

**RNA-seq Analysis        Case 10.7: RNA editing**

<table>
<tr>
<td>User's Inputs</td>
<td>You are a bioinformatician who specializes in writing code and performing analysis for a variety of bioinformatic data analysis tasks. Now I provide you with an Ubuntu 18.04 system where I save the following data, each line including the data path and data description.<br><br>SRR14463651_1.fastq.gz: paired end RNA sequencing data from human lymphoma cell, read 1 of lymphoma samples replicate 1<br>SRR14463651_2.fastq.gz: paired end RNA sequencing data from human lymphoma cell, read 2 of lymphoma samples replicate 1<br>SRR14463652_1.fastq.gz: paired end RNA sequencing data from human lymphoma cell, read 1 of lymphoma samples replicate 2<br>SRR14463652_2.fastq.gz: paired end RNA sequencing data from human lymphoma cell, read 2 of lymphoma samples replicate 2<br>SRR14463680_1.fastq.gz: paired end RNA sequencing data from human lymphoma cell, read 1 of control samples replicate 1<br>SRR14463680_2.fastq.gz: paired end RNA sequencing data from human lymphoma cell, read 2 of control samples replicate 1<br>SRR14463681_1.fastq.gz: paired end RNA sequencing data from human lymphoma cell, read 1 of control samples replicate 2<br>SRR14463681_2.fastq.gz: paired end RNA sequencing data from human lymphoma cell, read 2 of control samples replicate 2<br>hg38.fa: reference genome<br>gencode.v38.annotation.gtf: gene annotation<br>TruSeq3-PE.fa: trimming adapter<br><br>I need you to help me plan what needs to be done in each step of the analysis process, and help me write the code for each step to install softwares, use tools and accomplish the following main goal:<br><br>identifying RNA edting events in lymphoma and control samples</td>
</tr>
<tr>
<td>Generated Plans by AutoGPT</td>
<td>## Step 1<br><br>### Action executed:<br>Web search for 'how to install FastQC on Ubuntu 18.04'<br>### Function Call:<br>```javascript<br>web_search({<br>  "queries": [<br>    "how to install FastQC on Ubuntu 18.04"<br>  ]<br>})<br>```<br>### Result<br>```<br>Found the following result for the web search: 'how to install FastQC on Ubuntu 18.04'<br>--------------<br>To install FastQC on Ubuntu 18.04, follow these steps:<br><br>1. Install JAVA if you don't have it already:<br>   ```<br>   sudo apt install default-jre</td>
</tr>
</table>

```

2. Download FastQC:
```
mkdir -p ~/Downloads/bin
cd ~/Downloads/bin
wget
https://www.bioinformatics.babraham.ac.uk/projects/fastqc/fastqc_v0.11.8.zip
unzip fastqc_v0.11.8.zip
rm fastqc_v0.11.8.zip
```

3. Make sure that FastQC is executable:
```
sudo chmod 770 FastQC/fastqc
```

Alternatively, you can install FastQC using the apt package manager:

1. Update your package list:
```
sudo apt update
```

2. Install FastQC:
```
sudo apt install fastqc
```

Please note that the version of FastQC installed via the apt package manager may not be the latest version. If you need a specific version, it's recommended to download it directly from the FastQC website as shown in the first method.

```
## Step 2

### Action executed:
Web search for 'how to install Trimmomatic on Ubuntu 18.04'
### Function Call:
```javascript
web_search({
  "queries": [
    "how to install Trimmomatic on Ubuntu 18.04"
  ]
})
```
Result
```
```

Found the following result for the web search: 'how to install Trimmomatic on Ubuntu 18.04'

To install Trimmomatic on Ubuntu 18.04, follow these steps:

1. Update the package repositories and get the latest package information by running the command: `sudo apt-get update -y` or `sudo apt update`

2. Install the Trimmomatic package by running the command: `sudo apt-get install -y trimmomatic` or `sudo apt install trimmomatic`

3. Check the system logs to confirm that there are no related errors. You can use tools like ZoomAdmin to check the logs.

```
## Step 3

### Action executed:
Web search for 'how to install HISAT2 on Ubuntu 18.04'
### Function Call:
```javascript
web_search({
  "queries": [
    "how to install HISAT2 on Ubuntu 18.04"
  ]
})
```
Result
```
Found the following result for the web search: 'how to install HISAT2 on Ubuntu 18.04'
--------------
To install HISAT2 on Ubuntu 18.04, follow these steps:

1. Update package repositories and get the latest package information by running the command: `sudo apt-get update -y`

2. Install the packages and dependencies with the command: `sudo apt-get install -y hisat2`

Alternatively, you can download and extract the binary executable:

1. Navigate to your workspace/bin directory with the command: `cd ~/workspace/bin`

2. Download the binary files with the command: `wget ftp://ftp.ccb.jhu.edu/pub/infphilo/hisat2/downloads/hisat2-2.0.4-Linux_x86_64.zip`

3. Extract the downloaded file with the command: `unzip hisat2-2.0.4-Linux_x86_64.zip`

Please note that the information might be incomplete as it's extracted from different webpages.

--------------

```
Step 4

Action executed:
Web search for 'how to install StringTie on Ubuntu 18.04'
Function Call:
```javascript
web_search({
  "queries": [
    "how to install StringTie on Ubuntu 18.04"
  ]
})
```
Result
```
Found the following result for the web search: 'how to install StringTie on Ubuntu 18.04'
--------------
To install StringTie on Ubuntu 18.04, you can follow these steps:

Method 1: Using apt-get or apt
1. Update apt database with the following command: `sudo apt-get update`
2. Install StringTie using the following command: `sudo apt-get -y install stringtie`

Method 2: Using aptitude
1. Update apt database with the following command: `sudo aptitude update`
2. Install StringTie using the following command: `sudo aptitude -y install stringtie`

Method 3: Downloading and extracting the binary distribution
1. Navigate to your workspace directory: `cd ~/workspace/bin`
2. Download the binary distribution: `wget http://ccb.jhu.edu/software/stringtie/dl/stringtie-2.2.1.Linux_x86_64.tar.gz`
3. Extract the downloaded file: `tar -xzvf stringtie-2.2.1.Linux_x86_64.tar.gz`
4. Make a symlink to make it easier to find: `ln -s ~/workspace/bin/stringtie-2.2.1.Linux_x86_64/stringtie ~/workspace/bin/stringtie`
5. Test the installation: `~/workspace/bin/stringtie -h`

Please note that the version of StringTie may vary, so replace "2.2.1" with the version you are installing.

--------------

```
## Step 5
```

Action executed:
Web search for 'how to install DESeq2 on Ubuntu 18.04'
Function Call:
```javascript
web_search({
  "queries": [
    "how to install DESeq2 on Ubuntu 18.04"
  ]
})
```
Result
```
Found the following result for the web search: 'how to install DESeq2 on Ubuntu 18.04'
--------------
To install DESeq2 on Ubuntu 18.04, you need to have R version 3.5.1 installed. Here are the steps:

1. Check your R version by typing `R.version` in your R console. The output should show `R version 3.5.1`.

2. Install the Bioconductor package manager if it's not already installed. You can do this by running the following commands in your R console:
   ```
   if (!requireNamespace("BiocManager", quietly = TRUE))
   install.packages("BiocManager")
   ```

3. Install DESeq2 using the Bioconductor package manager by running the following command in your R console:
   ```
   BiocManager::install("DESeq2")
   ```

Please note that the installation might fail if certain dependencies are not met. If you encounter errors, you might need to install additional packages or resolve other issues based on the error messages.

The information provided does not specify how to handle decimal precision during the installation process.

--------------

```
Step 6

Action executed:
Web search for 'how to preprocess RNA-seq data'
Function Call:
```javascript
web_search({
  "queries": [
    "how to preprocess RNA-seq data"
```

]
})
```
### Result
```
Found the following result for the web search: 'how to preprocess RNA-seq data'
----
1. Quality Check: The overall quality of the sequence information is checked. This is the first step in preprocessing of raw reads for RNA-seq data.

2. Removal of Contaminations: Contaminations from other organisms are removed. This is done after the quality check.

3. Second Quality Control: Another quality control run is performed to confirm that the sequence data is now acceptable. This is done after the removal of contaminations.

4. Use of Tools: Tools like FastQC are used for sequence data quality control and for filtering or trimming.

5. Preprocessing: The most time demanding step in the analysis of RNA-seq data is preprocessing the raw sequence data, such as running quality control and adapter, contamination and quality filtering.

6. RNA-seq Analysis: To complete an RNA-seq analysis, researchers must find relevant studies, collect the necessary metadata, quantify read mapping, calculate the DEGs, perform Gene Ontology (GO) enrichment, and compute significant signaling pathways.

Note: The information is incomplete as it does not provide specific details on how to perform each step or the tools used for each step.

```
## Step 7

### Action executed:
Web search for 'how to align RNA-seq reads to the reference genome'
### Function Call:
```javascript
web_search({
  "queries": [
    "how to align RNA-seq reads to the reference genome"
  ]
})
```
Result
```
Found the following result for the web search: 'how to align RNA-seq reads to the reference genome'

--------------
1. Choose an appropriate reference genome to map the RNA-seq reads against.
2. Use splice-aware alignment tools such as STAR or HISAT2 for the alignment process.
3. With a tool like STAR, you can "project" genomic alignments directly to transcriptomic coordinates.
4. RSEM, Salmon, and eXpress are capable of processing these alignments.
5. The original reads can be aligned against the assembly using Bowtie2 and quantified with Salmon.
6. STAR has the ability to project the aligned reads to transcriptomic coordinates, allowing for a consistent quantification method.
7. TopHat is another popular RNA-seq mapper that aligns reads in two steps: unspliced reads are mapped to locate exons with Bowtie, and unmapped reads are then split and aligned independently to identify exon junctions.
8. The goal of the read alignment is to map short sequencing reads efficiently to a large reference genome to identify the 'correct' genomic loci from which the read originated while taking into account errors in the sequence reads.
9. Two alignment strategies are available: STAR, a gapped RNA-Seq read mapper with support for splice-junction and fusion read detection, and BWA, a software package for mapping low-divergent sequences against a large reference genome.
10. The RNA-seq data analysis process includes quality control of the reads, alignment of the reads to the reference genome, conversion of the files to raw counts, analysis of the counts with DeSeq2, and finally annotation of the reads using Biomart.

--------------

```

Step 8

Action executed:
Web search for 'how to quantify gene expression levels'
Function Call:
```javascript
web_search({
  "queries": [
    "how to quantify gene expression levels"
  ]
})
```
Result
```
Found the following result for the web search: 'how to quantify gene expression levels'
--------------
1. Gene expression levels can be quantified using a method called multiplex qPCR. This approach allows for the detection of the levels of RNA from multiple genes in the same qPCR reaction.

2. Gene expression level can be obtained in quantitative form using microarrays and RNA-seq.

3. Microarrays allow the analysis of the expression levels of thousands of preselected genes in one experiment. The basic principle behind microarrays is the hybridization of complementary DNA fragments.

4. Another technique for studying the expression of multiple genes simultaneously is SAGE (serial analysis of gene expression).

5. The simplest approach to quantifying gene expression by RNA-seq is to count the number of reads that map (i.e. align) to each gene (read count) using programs such as HTSeq-count. This gene-level quantification approach utilises a gene transfer format (GTF) file containing gene models, with each model representing the structure of transcripts produced by a given gene.

6. Expression levels can be measured relative to other genes (relative quantification) or against a standard (absolute quantification). Real-time PCR is the gold standard in nucleic acid quantification because of its accuracy and sensitivity. Real-time PCR can be used to quantitate mRNA or miRNA expression following conversion to cDNA or to quantitate genomic DNA directly to investigate transcriptional activity.

7. The relative amounts of mRNA are a relative measure of the rate of synthesis of proteins, if one assumes that the rate of synthesis of each protein is similarly proportional to the amount of its mRNA.

Note: The information is incomplete as it does not provide specific steps or procedures for quantifying gene expression levels.

--------------

```
Step 9

Action executed:
Web search for 'how to perform differential gene expression analysis'
Function Call:
```javascript
web_search({
  "queries": [
    "how to perform differential gene expression analysis"
  ]
})
```
Result
```
Found the following result for the web search: 'how to perform differential gene expression analysis'
--------------
To perform differential gene expression analysis:

1. Normalize the count data: This is necessary to make accurate comparisons of gene expression between samples. The counts of mapped reads for each gene is proportional to the amount of RNA that was present in the original sample.

2. Perform exploratory data analysis: This is done for quality assessment and to explore the relationship between samples.

3. Perform differential expression analysis: This is done to determine which genes are expressed at different levels between conditions.

4. Visually explore the results: This is done prior to performing downstream functional analysis.

5. Filter and Sort: Keep the genes that have an adjusted p-value of less or equal to 0.05.

Note: The differential expression analysis and any downstream functional analysis are generally performed in R using R packages specifically designed for the complex statistical analyses required to determine whether genes are differentially expressed.

--------------

```
Step 10

Action executed:
Web search for 'how to identify and quantify alternative polyadenylation'
Function Call:
```javascript
web_search({
  "queries": [
    "how to identify and quantify alternative polyadenylation"
  ]
})
```
Result
```
Found the following result for the web search: 'how to identify and quantify alternative polyadenylation'
--------------
The identification and quantification of alternative polyadenylation sites can be achieved through various methods. One such method is the use of RNA-seq data. A survey on this topic was conducted by Chen M., Ji G., Fu H., Lin Q., Ye C., Ye W., Su Y., & Wu X. in 2020, and it was published in Briefings in Bioinformatics, volume 21, issue 4, pages 1261–1276 (DOI: 10.1093/bib/bbz068).

Another tool mentioned for the identification and quantification of alternative polyadenylation sites from RNA-seq data is APAtrap. This tool was

| | |
|---|---|
| | discussed in a publication in Bioinformatics in 2018, volume 34, pages 1841-1849 (DOI: 10.1093/bioinformatics/bty029).<br><br>Additionally, 3′-Seq and Iso-Seq are methods that can identify and quantify the usage of polyadenylation sites more reliably than computational tools that take short-read RNA-seq as input. However, a tool called QAPA, when run with a set of polyadenylation site annotations derived from small quantities of 3′-Seq or Iso-Seq, can reliably quantify variation in APA across conditions, such as across genotypes.<br><br>Unfortunately, the text does not provide any decimal precision data related to the identification and quantification of alternative polyadenylation.<br><br>-------------- |

**RNA-seq Analysis    Case 10.8: circular RNA analysis**

<table>
<tr><td>User's Inputs</td><td>You are a bioinformatician who specializes in writing code and performing analysis for a variety of bioinformatic data analysis tasks. Now I provide you with an Ubuntu 18.04 system where I save the following data, each line including the data path and data description.<br><br>SRR14463651_1.fastq.gz: paired end RNA sequencing data from human lymphoma cell, read 1 of lymphoma samples replicate 1<br>SRR14463651_2.fastq.gz: paired end RNA sequencing data from human lymphoma cell, read 2 of lymphoma samples replicate 1<br>SRR14463652_1.fastq.gz: paired end RNA sequencing data from human lymphoma cell, read 1 of lymphoma samples replicate 2<br>SRR14463652_2.fastq.gz: paired end RNA sequencing data from human lymphoma cell, read 2 of lymphoma samples replicate 2<br>SRR14463680_1.fastq.gz: paired end RNA sequencing data from human lymphoma cell, read 1 of control samples replicate 1<br>SRR14463680_2.fastq.gz: paired end RNA sequencing data from human lymphoma cell, read 2 of control samples replicate 1<br>SRR14463681_1.fastq.gz: paired end RNA sequencing data from human lymphoma cell, read 1 of control samples replicate 2<br>SRR14463681_2.fastq.gz: paired end RNA sequencing data from human lymphoma cell, read 2 of control samples replicate 2<br>hg38.fa: reference genome<br>gencode.v38.annotation.gtf: gene annotation<br>TruSeq3-PE.fa: trimming adapter<br><br>I need you to help me plan what needs to be done in each step of the analysis process, and help me write the code for each step to install softwares, use tools and accomplish the following main goal:<br><br>identify and quantify circular RNA in lymphoma and control samples</td></tr>
<tr><td>Generated Plans by AutoGPT</td><td>## Step 1<br><br>### Action executed:<br>Agent message<br>Based on the goal, here is the plan for the analysis process and the code for each step:<br><br>1. **Install Required Software and Tools**<br>- Update the system packages:<br>```<br>sudo apt-get update<br>sudo apt-get upgrade<br>```<br>- Install FastQC for quality control of sequencing data:<br>```<br>sudo apt-get install fastqc<br>```<br>- Install HISAT2 for aligning RNA-seq reads to the reference genome:<br>```<br>sudo apt-get install hisat2<br>```<br>- Install StringTie for transcript assembly and quantification:</td></tr>
</table>

```
sudo apt-get install stringtie
```

- Install CIRI2 for circular RNA identification and quantification:

```
wget http://sourceforge.net/projects/ciri/files/CIRI2/CIRI_v2.0.6.zip
unzip CIRI_v2.0.6.zip
cd CIRI_v2.0.6
perl Makefile.PL
make
make install
```

2. **Preprocess the RNA-seq Data**
   - Use FastQC to assess the quality of the raw sequencing data:

```
fastqc SRR14463651_1.fastq.gz SRR14463651_2.fastq.gz
SRR14463652_1.fastq.gz SRR14463652_2.fastq.gz
SRR14463680_1.fastq.gz SRR14463680_2.fastq.gz
SRR14463681_1.fastq.gz SRR14463681_2.fastq.gz
```

   - Trim the adapter sequences from the RNA-seq reads using TruSeq3-PE.fa as the adapter file. You can use a tool like Trimmomatic for this:

```
java -jar trimmomatic-0.36.jar PE -phred33 input_forward.fq.gz
input_reverse.fq.gz output_forward_paired.fq.gz
output_forward_unpaired.fq.gz output_reverse_paired.fq.gz
output_reverse_unpaired.fq.gz ILLUMINACLIP:TruSeq3-PE.fa:2:30:10
LEADING:3 TRAILING:3 SLIDINGWINDOW:4:15 MINLEN:36
```

   - Perform quality control checks again using FastQC on the trimmed reads.

3. **Align RNA-seq Reads to the Reference Genome**
   - Use HISAT2 to align the trimmed RNA-seq reads to the reference genome (hg38.fa):

```
hisat2 -p 8 --dta -x hg38 -1 trimmed_paired_1.fq -2 trimmed_paired_2.fq
-S aligned.sam
```

4. **Transcript Assembly and Quantification**
   - Use StringTie to assemble transcripts and estimate their abundances for each sample:

```
stringtie -p 8 -G gencode.v38.annotation.gtf -o out.gtf -l sample
aligned.bam
```

5. **Circular RNA Identification and Quantification**
   - Use CIRI2 to identify and quantify circular RNA from the aligned reads:

```
```

	perl CIRI2.pl -I aligned.bam -O circRNA_identified -F hg38.fa -A gencode.v38.annotation.gtf<br>```<br><br>6. **Statistical Analysis and Visualization**<br>- Perform statistical analysis (e.g., differential expression analysis) on the identified circular RNA. You can use a tool like DESeq2 for this. This step will require R programming skills.<br><br>7. **Interpretation and Reporting**<br>- Analyze the results and interpret the findings in the context of lymphoma.<br>- Prepare a report summarizing the analysis steps, results, and conclusions. Include relevant figures and tables to support the findings.<br><br>Please note that the specific commands and code required for each step may vary depending on the software versions, data formats, and specific analysis requirements.

Small RNA-seq Analysis **Case 11.1 and Case 11.2: miRNA analysis**

<table>
<tr><td>User's Inputs</td><td>You are a bioinformatician who specializes in writing code and performing analysis for a variety of bioinformatic data analysis tasks. Now I provide you with an Ubuntu 18.04 system where I save the following data, each line including the data path and data description.

SRR10915820.fastq.gz: single end small RNA sequencing data from human cell line, control samples replicate 1
SRR10915821.fastq.gz: single end small RNA sequencing data from human cell line, control samples replicate 1
SRR10915822.fastq.gz: single end small RNA sequencing data from human cell line, control samples replicate 1
SRR10915823.fastq.gz: single end small RNA sequencing data from human cell line, control samples replicate 1
hsa.gff3: microRNA genomic coordinate from miRbase
TGGAATTCTCGGGTGCCAAGG: adaptor sequence
hg38.fa: the reference genome

I need you to help me plan what needs to be done in each step of the analysis process, and help me write the code for each step to install softwares, use tools and accomplish the following main goal:

quantify microRNA expression based on miRbase and predict novel microRNA</td></tr>
<tr><td>Generated Plans by AutoGPT</td><td>## Step 1

Action executed:
Agent message
Based on the goal, the following steps need to be taken:

1. **Install necessary software and tools for the analysis**:
- Install bioinformatics tools such as Bowtie, featureCounts, and miRDeep2.

2. **Preprocess the sequencing data**:
- Perform quality control checks and trimming of adapter sequences (e.g., using FastQC and Cutadapt).

3. **Align the preprocessed data to the reference genome**:
- Use Bowtie to align the preprocessed data to the reference genome hg38.fa.

4. **Extract microRNA genomic coordinates from miRbase**:
- Parse the hsa.gff3 file to extract the genomic coordinates of known microRNAs.

5. **Quantify microRNA expression using the aligned data and miRbase coordinates**:
- Use featureCounts to count the number of reads mapping to each microRNA.

6. **Predict novel microRNAs based on the analysis results**:</td></tr>
</table>

	- Utilize miRDeep2 to predict novel microRNAs based on the aligned data and known microRNA expression.<br><br>The specific code for each step will depend on the software and tools being used, and the programming language of choice.<br><br>Formatting Requirements:<br><br>1. Organize the data description in a list format, with each line including the data path and data description.<br><br>2. Use proper punctuation and capitalization for clarity and readability.<br><br>3. Use a consistent and clear naming convention for the data files and descriptions.<br><br>4. Use code formatting for any code snippets or commands provided.<br><br>5. Clearly separate each step of the analysis process and code snippets for each step.<br><br>6. Use appropriate indentation and spacing for code readability.<br><br>7. Use appropriate headings or subheadings to distinguish different sections or steps of the analysis process.<br><br>8. Use a clear and concise language to describe the steps and code.<br><br>9. Use appropriate syntax highlighting or formatting for code snippets, if applicable.<br><br>10. Provide any additional instructions or explanations in a clear and organized manner.<br><br>Remember to consider the specific requirements and preferences of the intended audience while formatting the information.

CAGE-seq Analysis **Case 12.1: TSS analysis**

<table>
<tr><td>User's Inputs</td><td>You are a bioinformatician who specializes in writing code and performing analysis for a variety of bioinformatic data analysis tasks. Now I provide you with an Ubuntu 18.04 system where I save the following data, each line including the data path and data description.

SRR11351697.fastq.gz: CAGE-seq data from human cell line, control samples replicate 1
SRR11351698.fastq.gz: CAGE-seq data from human cell line, control samples replicate 2
SRR11351700.fastq.gz: CAGE-seq data from human cell line, treatment samples replicate 1
SRR11351701.fastq.gz: CAGE-seq data from human cell line, treatment samples replicate 1
hg38.fa: the human reference genome
gencode.v37.annotation.gtf: gene annotation
TruSeq3-SE.fa: trimming adapter

I need you to help me plan what needs to be done in each step of the analysis process, and help me write the code for each step to install softwares, use tools and accomplish the following main goal:

Identify transcription start site (TSS) in the cell line based on CAGE-seq data</td></tr>
<tr><td>Generated Plans by AutoGPT</td><td>## Step 1

Action executed:
Agent message
Based on the goal, here is the plan for the analysis process:

1. **Install Required Software**:
   - Install Ubuntu 18.04 system on your machine.
   - Install necessary software packages such as Python, R, and bioinformatics tools like BEDTools, SAMtools, and HISAT2.

2. **Download and Prepare Data**:
   - Download the provided data files (SRR11351697.fastq.gz, SRR11351698.fastq.gz, SRR11351700.fastq.gz, SRR11351701.fastq.gz, hg38.fa, gencode.v37.annotation.gtf, TruSeq3-SE.fa) to your local system.
   - Create a project directory and organize the data files within it.

3. **Quality Control and Preprocessing**:
   - Perform quality control checks on the CAGE-seq data using tools like FastQC.
   - Trim adapter sequences from the CAGE-seq data using TruSeq3-SE.fa and tools like Cutadapt or Trimmomatic.
   - Perform additional preprocessing steps like read filtering, quality trimming, and read alignment using HISAT2 and the human reference genome (hg38.fa).

4. **Peak Calling and TSS Identification**:</td></tr>
</table>

	- Use a peak calling algorithm such as MACS2 or HOMER to identify peaks or enriched regions in the aligned CAGE-seq data.<br>- Filter the identified peaks based on statistical significance and other criteria.<br>- Use the gene annotation file (gencode.v37.annotation.gtf) to associate the identified peaks with nearby genes.<br>- Identify the TSS regions based on the peak locations and gene annotations.<br><br>5. **Data Analysis and Visualization**:<br>- Perform downstream analysis on the identified TSS regions, such as differential expression analysis, motif enrichment analysis, or functional annotation.<br>- Use bioinformatics tools and programming languages like Python or R to analyze and visualize the results.<br>- Generate plots, graphs, and summary statistics to interpret and present the findings.<br><br>The data should be formatted as follows:<br><br>- SRR11351697.fastq.gz: CAGE-seq data from human cell line, control samples replicate 1<br>- SRR11351698.fastq.gz: CAGE-seq data from human cell line, control samples replicate 2<br>- SRR11351700.fastq.gz: CAGE-seq data from human cell line, treatment samples replicate 1<br>- SRR11351701.fastq.gz: CAGE-seq data from human cell line, treatment samples replicate 1<br>- hg38.fa: the human reference genome<br>- gencode.v37.annotation.gtf: gene annotation<br>- TruSeq3-SE.fa: trimming adapter<br><br>Please note that this is just one possible way to format the data. The actual formatting can vary based on personal preference or specific requirements.

3’end-seq Analysis Case 13.1: APA analysis based on 3’end-seq data

<table>
<tr><td>User’s Inputs</td><td>You are a bioinformatician who specializes in writing code and performing analysis for a variety of bioinformatic data analysis tasks. Now I provide you with an Ubuntu 18.04 system where I save the following data, each line including the data path and data description.

SRR17422754.fastq.gz: Lexongen QuantSeq 3 prime-end RNA sequencing data from human cell line THLE2, healthy liver cell line replicate 1
SRR17422755.fastq.gz: Lexongen QuantSeq 3 prime-end RNA sequencing data from human cell line THLE2, healthy liver cell line replicate 2
SRR17422756.fastq.gz: Lexongen QuantSeq 3 prime-end RNA sequencing data from human cell line SNU398, liver cancer cell line replicate 1
SRR17422757.fastq.gz: Lexongen QuantSeq 3 prime-end RNA sequencing data from human cell line SNU398, liver cancer cell line replicate 2
hg38.fa: the human reference genome
gencode.v37.annotation.gtf: gene annotation
TruSeq3-SE.fa: trimming adapter

I need you to help me plan what needs to be done in each step of the analysis process, and help me write the code for each step to install softwares, use tools and accomplish the following main goal:

Identify polyadenylation sites and quantify alternative polyadenylation based on 3'-end sequencing data</td></tr>
<tr><td>Generated Plans by AutoGPT</td><td>## Step 1

Action executed:
Agent message
Based on the goal, here is the plan for the analysis process and the code for each step:

1. **Install Required Software**:
  - Install Ubuntu 18.04 system on your machine.
  - Install necessary software packages for bioinformatics analysis, such as:
    - FastQC: for quality control of sequencing data.
    - Trimmomatic: for trimming adapter sequences from the reads.
    - STAR: for aligning the reads to the reference genome.
    - BEDTools: for manipulating genomic intervals.
    - SAMtools: for manipulating sequence alignment files.
    - R: for data analysis and visualization.

2. **Download and Prepare Data**:
  - Download the required data files:
    - SRR11351697.fastq.gz
    - SRR11351698.fastq.gz
    - SRR11351700.fastq.gz
    - SRR11351701.fastq.gz
    - hg38.fa
    - gencode.v37.annotation.gtf
    - TruSeq3-SE.fa
  - Create a project directory and organize the data files within it.

3. **Quality Control and Trimming**:</td></tr>
</table>

	- Run FastQC on the CAGE-seq data files to assess the quality of the sequencing reads.<br>- Use Trimmomatic to remove adapter sequences and low-quality bases from the reads.<br>- Generate trimmed and filtered FASTQ files for each sample.<br><br>4. **Genome Alignment**:<br>- Index the reference genome (hg38.fa) using STAR.<br>- Align the trimmed CAGE-seq reads to the indexed genome using STAR.<br>- Generate sorted and indexed BAM files for each sample.<br><br>5. **Peak Calling and TSS Identification**:<br>- Use a peak calling algorithm, such as MACS2 or HOMER, to identify enriched regions representing potential TSS.<br>- Convert the identified peaks to a BED file format.<br>- Use BEDTools to intersect the identified peaks with the gene annotation file (gencode.v37.annotation.gtf) to assign TSS to specific genes.<br>- Generate a final list of TSS coordinates and associated gene information.<br><br>6. **Data Analysis and Visualization**:<br>- Use R or any preferred programming language to analyze and visualize the TSS data.<br>- Perform statistical analysis, such as differential expression analysis, if applicable.<br>- Generate plots, heatmaps, or other visualizations to represent the TSS data.<br><br>Please note that the above steps provide a general outline of the analysis process. The specific commands and code required for each step may vary depending on the software versions, data format, and specific analysis goals. It is recommended to refer to the documentation and tutorials of the individual software tools for detailed instructions on their usage and parameters.

ONT sequencing Analysis   Case 14.1: isoform expression

<table>
<tr><td>User's Inputs</td><td>You are a bioinformatician who specializes in writing code and performing analysis for a variety of bioinformatic data analysis tasks. Now I provide you with an Ubuntu 18.04 system where I save the following data, each line including the data path and data description.

RHL5569-T1_SAECK_shControl_replicate_1.ont.fastq.gz: ONT sequencing data from human cell line, control samples replicate 1
RHL5570-T1_SAECK_shControl_replicate_2.ont.fastq.gz: ONT sequencing data from human cell line, control samples replicate 2
RHL5571-T1_SAECK_shPARP4-1_replicate_1.ont.fastq.gz: ONT sequencing data from human cell line, treatment samples replicate 1
RHL5572-T1_SAECK_shPARP4-1_replicate_2.ont.fastq.gz: ONT sequencing data from human cell line, treatment samples replicate 2
hg38.fa: the human reference genome
gencode.v37.annotation.gtf: gene annotation

I need you to help me plan what needs to be done in each step of the analysis process, and help me write the code for each step to install softwares, use tools and accomplish the following main goal:

Quantify isoform expression in control and treatment samples</td></tr>
<tr><td>Generated Plans by AutoGPT</td><td>Agent message
Based on the goal, the following steps are planned for the analysis process:

1. **Install Required Software**: Install the necessary software for isoform expression quantification, such as FastQC for quality control of sequencing data, HISAT2 for aligning sequencing reads to the reference genome, and StringTie for quantifying isoform expression levels.

2. **Download and Prepare Data**: Download the required data files and perform quality control on the sequencing data using FastQC. Trim adapters and low-quality bases from the sequencing reads if necessary.

3. **Align Sequencing Reads to the Reference Genome**: Use HISAT2 to align the trimmed sequencing reads to the human reference genome (hg38.fa). Generate alignment files (SAM/BAM format) for each sample.

4. **Quantify Isoform Expression**: Use StringTie to quantify isoform expression levels in the control and treatment samples. Provide the gene annotation file (gencode.v37.annotation.gtf) to guide the isoform quantification process. Generate expression values (e.g., FPKM or TPM) for each isoform in each sample.

5. **Statistical Analysis**: Perform statistical analysis to identify differentially expressed isoforms between control and treatment samples. Use appropriate statistical methods (e.g., DESeq2 or edgeR) to compare expression levels and identify significant differences.

6. **Visualization and Interpretation**: Visualize the results using plots, such as volcano plots or heatmaps, to identify the most significant differentially</td></tr>
</table>

	expressed isoforms. Interpret the results and provide insights into the impact of the treatment on isoform expression.<br><br>The formatting requirements are as follows:<br><br>1. Organize the provided data in a clear and readable format.<br>2. Use a consistent format for presenting the data path and description.<br>3. Clearly distinguish between different data files and their corresponding descriptions.<br>4. Use proper punctuation and capitalization for clarity and readability.<br>5. Use a consistent format for presenting the main goal.<br><br>Here is an example of how the provided data can be formatted:<br><br>Data:<br>- RHL5569-T1_SAECK_shControl_replicate_1.ont.fastq.gz: ONT sequencing data from human cell line, control samples replicate 1<br>- RHL5570-T1_SAECK_shControl_replicate_2.ont.fastq.gz: ONT sequencing data from human cell line, control samples replicate 2<br>- RHL5571-T1_SAECK_shPARP4-1_replicate_1.ont.fastq.gz: ONT sequencing data from human cell line, treatment samples replicate 1<br>- RHL5572-T1_SAECK_shPARP4-1_replicate_2.ont.fastq.gz: ONT sequencing data from human cell line, treatment samples replicate 2<br>- hg38.fa: the human reference genome<br>- gencode.v37.annotation.gtf: gene annotation<br><br>Main Goal:<br>Quantify isoform expression in control and treatment samples<br><br>Please note that this is just one possible formatting style, and you can adjust it according to your preferences or any specific formatting guidelines provided by your organization.

PacBio iso-seq Analysis **Case 15.1: isoform analysis**

<table>
<tr><td>User's Inputs</td><td>You are a bioinformatician who specializes in writing code and performing analysis for a variety of bioinformatic data analysis tasks. Now I provide you with an Ubuntu 18.04 system where I save the following data, each line including the data path and data description.

SRR19552218.fastq.gz: pacbio sequecning data from human cell line, cell line HCT-116
SRR19785215.fastq.gz: pacbio sequecning data from human cell line, cell line HepG2
hg38.fa: the human reference genome
gencode.v37.annotation.gtf: gene annotation

I need you to help me plan what needs to be done in each step of the analysis process, and help me write the code for each step to install softwares, use tools and accomplish the following main goal:

identify novel isoform and quantify isoform expression in two cell lines</td></tr>
<tr><td>Generated Plans by AutoGPT</td><td>## Step 1

Action executed:
Agent message
Based on the goal, here is the plan for the analysis process and the code for each step:

1. **Install required software and tools**:
- Minimap2: For aligning the sequencing data to the reference genome.
- SAMtools: For manipulating aligned data.
- StringTie: For assembling transcripts and estimating their abundances.
- GffCompare: For comparing identified transcripts to the reference annotation.

Code:
```bash
# Update the system
sudo apt-get update -y

# Install Minimap2
sudo apt-get install -y minimap2

# Install SAMtools
sudo apt-get install -y samtools

Install StringTie
wget http://ccb.jhu.edu/software/stringtie/dl/stringtie-2.1.4.Linux_x86_64.tar.gz
tar -xzvf stringtie-2.1.4.Linux_x86_64.tar.gz
export PATH=$PATH:$(pwd)/stringtie-2.1.4.Linux_x86_64

Install GffCompare
wget http://ccb.jhu.edu/software/stringtie/dl/gffcompare-0.11.6.Linux_x86_64.tar.gz</td></tr>
</table>

```
tar -xzvf gffcompare-0.11.6.Linux_x86_64.tar.gz
export PATH=$PATH:$(pwd)/gffcompare-0.11.6.Linux_x86_64
```

2. **Align the sequencing data to the reference genome**:
   - Use Minimap2 for alignment.

   Code:
   ```bash
   # Align SRR19552218.fastq.gz to hg38.fa
   minimap2 -ax map-pb hg38.fa SRR19552218.fastq.gz | samtools sort -o
SRR19552218.bam

   # Align SRR19785215.fastq.gz to hg38.fa
   minimap2 -ax map-pb hg38.fa SRR19785215.fastq.gz | samtools sort -o
SRR19785215.bam
   ```

3. **Assemble transcripts and estimate their abundances**:
   - Use StringTie for assembling transcripts and estimating their abundances.

   Code:
   ```bash
   # Assemble and quantify transcripts for SRR19552218.bam
   stringtie SRR19552218.bam -G gencode.v37.annotation.gtf -o
SRR19552218.gtf -B

   # Assemble and quantify transcripts for SRR19785215.bam
   stringtie SRR19785215.bam -G gencode.v37.annotation.gtf -o
SRR19785215.gtf -B
   ```

4. **Identify novel isoforms**:
   - Use GffCompare to compare identified transcripts to the reference annotation.

   Code:
   ```bash
   # Identify novel isoforms for SRR19552218.gtf
   gffcompare -r gencode.v37.annotation.gtf -G -o SRR19552218
SRR19552218.gtf

   # Identify novel isoforms for SRR19785215.gtf
   gffcompare -r gencode.v37.annotation.gtf -G -o SRR19785215
SRR19785215.gtf
   ```

5. **Compare isoform expression between the two cell lines**:
   - The output of StringTie includes estimated expression levels of each transcript. You can use these outputs to compare isoform expression between the two cell lines. This step may require additional scripting or use

	of downstream analysis tools depending on the specific comparison and visualization methods you want to use.<br><br>Please note that this is a basic plan and may need to be adjusted based on the specific requirements of your analysis. For example, you may need to add steps for quality control of the raw sequencing data, or you may need to use different tools or parameters based on the characteristics of your data.

CLIP-seq Analysis **Case 16.1: CLIP-seq data analysis**

**User's Inputs**	You are a bioinformatician who specializes in writing code and performing analysis for a variety of bioinformatic data analysis tasks. Now I provide you with an Ubuntu 18.04 system where I save the following data, each line including the data path and data description.<br><br>ENCLB742AYH.fastq.gz: single end reads of eCLIP data from HepG2 cell line, SRSF1 targeted sample replicate 1<br>ENCLB770EDJ.fastq.gz: single end reads of eCLIP data from HepG2 cell line, SRSF1 targeted sample replicate 2<br>hg38.fa: the human reference genome<br>gencode.v37.annotation.gtf: gene annotation<br><br>I need you to help me plan what needs to be done in each step of the analysis process, and help me write the code for each step to install softwares, use tools and accomplish the following main goal:<br><br>identify SRSF1 protein-RNA crosslink sites based on CLIP sequencing data
**Generated Plans by AutoGPT**	## Step 1<br><br>### Action executed:<br>Agent message<br>Based on the goal, here is the plan for the analysis process and the code for each step:<br><br>1. **Data Preparation**:<br>- Install necessary software and tools for bioinformatic analysis on the Ubuntu 18.04 system.<br>- Download the required data files:<br>- ENCLB742AYH.fastq.gz: single-end reads of eCLIP data from HepG2 cell line, SRSF1 targeted sample replicate 1.<br>- ENCLB770EDJ.fastq.gz: single-end reads of eCLIP data from HepG2 cell line, SRSF1 targeted sample replicate 2.<br>- hg38.fa: the human reference genome.<br>- gencode.v37.annotation.gtf: gene annotation file.<br><br>```bash<br># Install necessary software and tools<br># Example: Install FastQC, Trimmomatic, Bowtie, and peak calling tool (Piranha)<br>sudo apt-get install fastqc trimmomatic bowtie piranha<br><br># Download the data files<br># Example: Using wget command<br>wget <data_file_url><br><br># Repeat the above command for each data file<br>```<br><br>2. **Quality Control and Preprocessing**:<br>- Perform quality control checks on the eCLIP data using tools like FastQC.

	- Trim adapter sequences and low-quality bases from the reads using tools like Trimmomatic or Cutadapt.<br>- Perform quality filtering to remove low-quality reads.<br><br>3. **Alignment**:<br>- Align the preprocessed reads to the human reference genome (hg38.fa) using a read aligner like Bowtie, BWA, or STAR.<br>- Generate alignment files (BAM format) for each replicate.<br><br>4. **Peak Calling**:<br>- Identify protein-RNA crosslink sites (peaks) using peak calling algorithms specifically designed for CLIP-seq data, such as Piranha, PARalyzer, or CLIPper.<br>- Use the aligned reads (BAM files) and gene annotation file (gencode.v37.annotation.gtf) as input for peak calling.<br>- Obtain a list of SRSF1 protein-RNA crosslink sites (peaks) based on the CLIP sequencing data.<br><br>5. **Peak Annotation and Analysis**:<br>- Annotate the identified peaks with gene information using the gene annotation file (gencode.v37.annotation.gtf).<br>- Perform downstream analysis on the annotated peaks, such as motif analysis, enrichment analysis, or visualization.<br><br>Please note that the specific software and tools used may vary depending on your preferences and the specific requirements of your analysis.

RIP-Seq Analysis **Case 16.2: Identify enriched genes bounded by RPB**

**User's Inputs**	You are a bioinformatician who specializes in writing code and performing analysis for a variety of bioinformatic data analysis tasks. Now I provide you with an Ubuntu 18.04 system where I save the following data, each line including the data path and data description.<br><br>rip.rep1.fastq.gz: single end reads of RIP-seq data from HepG2 cell line, pulldown samples of SRSF1 replicate 1<br>rip.rep2.fastq.gz: single end reads of RIP-seq data from HepG2 cell line, pulldown samples of SRSF1 replicate 2<br>controll.rep1.fastq.gz: single end reads of RIP-seq daata from HepG2 cell line, control sample replicate 1<br>controll.rep2.fastq.gz: single end reads of RIP-seq daata from HepG2 cell line, control sample replicate 2<br>hg38.fa: the human reference genome<br>gencode.v37.annotation.gtf: gene annotation<br><br>I need you to help me plan what needs to be done in each step of the analysis process, and help me write the code for each step to install softwares, use tools and accomplish the following main goal:<br><br>identify genes enriched in pulldown samples over the control
**Generated Plans by AutoGPT**	## Step 1<br><br>### Action executed:<br>Agent message<br>Based on the provided information and the goal to identify genes enriched in pulldown samples over the control, here is the plan for the analysis process:<br><br>1. **Data Preparation**:<br>- Install necessary software and tools for the analysis process.<br>- Download and save the required data files (rip.rep1.fastq.gz, rip.rep2.fastq.gz, control.rep1.fastq.gz, control.rep2.fastq.gz, hg38.fa, gencode.v37.annotation.gtf) to a specific directory on the Ubuntu 18.04 system.<br><br>2. **Quality Control and Preprocessing**:<br>- Perform quality control checks on the raw sequencing data (rip.rep1.fastq.gz, rip.rep2.fastq.gz, control.rep1.fastq.gz, control.rep2.fastq.gz) using appropriate tools (e.g., FastQC).<br>- Preprocess the data by trimming adapters, removing low-quality reads, and filtering out any contaminants or artifacts using tools like Trimmomatic or Cutadapt.<br><br>3. **Alignment and Mapping**:<br>- Index the human reference genome (hg38.fa) using a tool like Bowtie or BWA.<br>- Map the preprocessed reads (from step 2) to the indexed reference genome using a read aligner such as Bowtie or HISAT2.<br>- Generate alignment files (BAM or SAM format) for each sample.

	4. **Read Counting and Differential Expression Analysis**:<br>- Use the gene annotation file (gencode.v37.annotation.gtf) to assign reads to genes and generate count tables for each sample using tools like featureCounts or HTSeq.<br>- Normalize the count tables to account for differences in library size and gene length using methods like TPM (Transcripts Per Million) or FPKM (Fragments Per Kilobase Million).<br>- Perform differential expression analysis between the pulldown samples (rip.rep1 and rip.rep2) and control samples (control.rep1 and control.rep2) using tools like DESeq2 or edgeR.<br>- Identify genes that are significantly enriched in the pulldown samples compared to the control samples based on statistical analysis results.<br><br>5. **Gene Annotation and Functional Analysis**:<br>- Annotate the identified enriched genes with additional information such as gene symbols, gene descriptions, and functional annotations using tools like biomaRt or DAVID.<br>- Perform functional enrichment analysis to identify enriched gene ontology (GO) terms, pathways, or biological processes associated with the identified genes using tools like clusterProfiler or Enrichr.<br><br>6. **Visualization and Reporting**:<br>- Generate visualizations such as heatmaps, volcano plots, or gene expression profiles to visualize the results of the analysis.<br>- Prepare a report summarizing the analysis process, including the identified enriched genes, their functional annotations, and any significant findings.<br><br>Please note that the specific software, tools, and code required for each step may vary depending on your preferences and the specific requirements of your analysis.

Ribo-Seq Analysis   Case 17.1: Identify translated ORFs

<table>
<tr><td>User's Inputs</td><td>You are a bioinformatician who specializes in writing code and performing analysis for a variety of bioinformatic data analysis tasks. Now I provide you with an Ubuntu 18.04 system where I save the following data, each line including the data path and data description.

SRR12354645_1.fastq.gz: read 1 of paired-end ribo-seq data, read 1 of control samples replicate 1
SRR12354645_2.fastq.gz: read 2 of paired-end ribo-seq data, read 2 of control samples replicate 1
SRR12354646_1.fastq.gz: read 1 of paired-end ribo-seq data, read 1 of control samples replicate 2
SRR12354646_2.fastq.gz: read 2 of paired-end ribo-seq data, read 2 of control samples replicate 2
hg38.fa: the human reference genome
gencode.v37.annotation.gtf: gene annotation
TruSeq3-PE.fa: trimming adapter

I need you to help me plan what needs to be done in each step of the analysis process, and help me write the code for each step to install softwares, use tools and accomplish the following main goal:

Identify translated ORFs based on ribo-seq data</td></tr>
<tr><td>Generated Plans by AutoGPT</td><td>## Step 1

Action executed:
Agent message
Based on the goal, here is the plan for the analysis process and the code for each step:

1. **Install Required Software**:
  - Install the necessary software packages for bioinformatic analysis, such as:
    - FastQC: for quality control of sequencing data.
    - Cutadapt: for adapter trimming.
    - STAR: for aligning reads to the reference genome.
    - RiboTaper: for identifying translated ORFs.
  - Use the package manager (e.g., apt-get) to install these tools on Ubuntu 18.04.

2. **Download Reference Data**:
  - Download the required reference data files:
    - hg38.fa: the human reference genome.
    - gencode.v37.annotation.gtf: gene annotation file.
    - TruSeq3-PE.fa: adapter sequences for trimming.
  - Store these files in a specific directory on the Ubuntu system.

3. **Preprocess Ribo-seq Data**:
  - Use Cutadapt to trim adapter sequences from the ribo-seq data.
  - Run FastQC to assess the quality of the trimmed data.
  - Perform any necessary quality filtering or trimming based on FastQC results.</td></tr>
</table>

	4. **Align Trimmed Data to Reference Genome**:<br>- Use STAR to align the trimmed ribo-seq data to the human reference genome.<br>- Generate alignment files (e.g., BAM files) for downstream analysis.<br><br>5. **Identify Translated ORFs**:<br>- Utilize RiboTaper to identify translated ORFs from the aligned ribo-seq data.<br>- RiboTaper uses ribosome profiling data to detect ribosome-protected fragments (RPFs) and infer translated ORFs.<br>- Adjust RiboTaper parameters as needed for your specific analysis.<br><br>6. **Analyze and Interpret Results**:<br>- Analyze the output from RiboTaper to identify translated ORFs.<br>- Explore differentially translated ORFs between control and experimental conditions.<br>- Perform statistical analysis and visualization to interpret the results.<br><br>Please note that the code for each step may vary depending on the specific software versions and command-line options. It is recommended to consult the documentation of each tool for detailed instructions on installation and usage.<br>### Prediction:

scRNA-Seq Analysis Case 18.1: Cluster cell into groups based on raw fastq files

**User's Inputs**	You are a bioinformatician who specializes in writing code and performing analysis for a variety of bioinformatic data analysis tasks. Now I provide you with an Ubuntu 18.04 system where I save the following data, each line including the data path and data description.<br><br>/pbmc_1k_v3_fastqs.tar: the packed fastq files from 10x single cell sequencing<br>refdata-gex-GRCh38-2020-A: directory contains transcriptome reference<br><br>I need you to help me plan what needs to be done in each step of the analysis process, and help me write the code for each step to install softwares, use tools and accomplish the following main goal:<br><br>cluster cells into different groups based on 10x single cell sequencing data
**Generated Plans by AutoGPT**	## Step 1<br><br>### Action executed:<br>Failed to sanitize function fs_readDirectory with args {<br>"path": "/pbmc_1k_v3_fastqs.tar"<br>}. Error: Function fs_readDirectory does not exist. Only use the functions you have been provided with<br>Function fs_readDirectory does not exist. Only use the functions you have been provided with<br>### Prediction:<br>-> The best next step for the assistant would be to analyze the provided data and plan the steps required for clustering cells based on the 10x single cell sequencing data. This would involve installing necessary software, using appropriate tools, and implementing the code for each step of the analysis process.<br><br>### Selected agent:<br>-> CsvAnalyst<br><br>## Step 2<br><br>### Action executed:<br>Agent message<br>I apologize for the confusion. I don't have the capability to read directories or files directly. However, based on the information provided, here's a general plan for the analysis process:<br><br>1. **Data Preparation**: Unpack the fastq files from '/pbmc_1k_v3_fastqs.tar'. This can be done using the `tar` command in Ubuntu.<br><br>2. **Quality Control**: Perform quality control on the unpacked fastq files. This can be done using tools like FastQC.<br><br>3. **Read Alignment**: Align the reads to the reference genome. The reference genome is provided in the 'refdata-gex-GRCh38-2020-A' directory. This can be done using tools like STAR.

	4. **Quantification**: Quantify the gene expressions. This can be done using tools like featureCounts.<br><br>5. **Normalization**: Normalize the gene expression data. This can be done using tools like DESeq2.<br><br>6. **Clustering**: Cluster the cells into different groups based on the normalized gene expression data. This can be done using tools like Seurat.<br><br>Please note that each of these steps requires specific bioinformatics tools and they need to be installed on the Ubuntu system. The installation process varies for each tool. Also, each step may require specific parameters that need to be adjusted based on the data and the specific research question.

scRNA-Seq Analysis Case 18.2: Clustering and Find the differentially expressed genes

**User's Inputs**	You are a bioinformatician who specializes in writing code and performing analysis for a variety of bioinformatic data analysis tasks. Now I provide you with an Ubuntu 18.04 system where I save the following data, each line including the data path and data description.<br><br>./examples/case2.1/data/filtered_gene_bc_matrices/hg19: path to 10x mtx data<br><br>I need you to help me plan what needs to be done in each step of the analysis process, and help me write the code for each step to install softwares, use tools and accomplish the following main goal:<br><br>Use scanpy to find the differentially expressed genes
**Generated Plans by AutoGPT**	## Step 1<br><br>### Action executed:<br>Web search for 'How to install scanpy on Ubuntu 18.04'<br>### Function Call:<br>```javascript<br>web_search({<br>"queries": [<br>"How to install scanpy on Ubuntu 18.04"<br>]<br>})<br>```<br>### Result<br>```<br>Found the following result for the web search: 'How to install scanpy on Ubuntu 18.04'<br>--------------<br>To install scanpy on Ubuntu 18.04, you can use either pip or conda. Here are the steps:<br><br>1. Using pip:<br>- Run the command `pip install scanpy` in your terminal. If you prefer to use Python 3, consider using `pip3` instead of `pip`.<br>- If you want to install additional packages that are needed for popular parts of scanpy but aren't requirements, you can run `pip install 'scanpy[leiden]'`. This will install igraph and leiden.<br><br>2. Using conda:<br>- If you do not have a working installation of Python 3.6 (or later), consider installing Miniconda.<br>- After installing Miniconda, run the command `conda install -c conda-forge scanpy python-igraph leidenalg`.<br><br>After installation, you can check if scanpy is installed correctly by opening Python 3 interpreter from the terminal by running `python` and then `import scanpy as sc`.<br><br>Please note that the information might be incomplete as it's extracted from different webpages and some information might not be properly formatted.

```
## Step 2

### Action executed:
Web search for 'How to load 10x mtx data into scanpy'
### Function Call:
```javascript
web_search({
  "queries": [
    "How to load 10x mtx data into scanpy"
  ]
})
```
Result
```
Found the following result for the web search: 'How to load 10x mtx data into scanpy'
--------------
To load 10x mtx data into Scanpy, you can use the function `scanpy.read_10x_mtx`. The function takes several parameters:

- `path`: The path to the directory containing the .mtx file.
- `var_names`: This can be set to 'gene_symbols'.
- `make_unique`: This can be set to True.
- `cache`: This can be set to False.
- `cache_compression`: This can be left empty.
- `gex_only`: This can be set to True.
- `prefix`: This can be left as None.

Please note that the information is incomplete and may require additional parameters or steps.

--------------

```
Step 3

Action executed:
Web search for 'How to perform differential expression analysis with scanpy'
Function Call:
```javascript
web_search({
  "queries": [
    "How to perform differential expression analysis with scanpy"
  ]
})
```
Result
```

Found the following result for the web search: 'How to perform differential expression analysis with scanpy'

To perform differential expression analysis with Scanpy, the function `rank_genes_group` is used. The default method to compute differential expression is the `t-test_overestim_var`. Other implemented methods include `logreg`, `t-test`, and `wilcoxon`. By default, the .raw attribute of AnnData is used if it has been initialized, but this can be changed by setting `use_raw=False`.

In addition to these methods, `diffxpy` is also used for differential expression testing in Scanpy.

The clustering with resolution 0.6 is suggested to give a reasonable number of clusters for the analysis.

Scanpy is a scalable toolkit for analyzing single-cell gene expression data. It includes methods for preprocessing, visualization, clustering, trajectory inference, and differential expression testing. The Python-based implementation can efficiently deal with datasets of more than one million cells.

Please note that this information might be incomplete as it is based on the provided text chunks.

```
## Step 4

### Action executed:
Web search for 'How to visualize differentially expressed genes in scanpy'
### Function Call:
```javascript
web_search({
  "queries": [
    "How to visualize differentially expressed genes in scanpy"
  ]
})
```
Result
```
Found the following result for the web search: 'How to visualize differentially expressed genes in scanpy'
--------------
To visualize differentially expressed genes in Scanpy, you can use various methods such as dotplots, violin plots, heatmaps, and tracksplots.

1. Dotplots: This visualization is useful to get an overview of the genes that show differential expression. You can use `sc.pl.rank_genes_groups_dotplot(pbmc, n_genes=4)` to show only the top 4 scoring genes. You can also plot log fold changes instead of gene expression to get a better representation.

| | |
|---|---|
| | 2. Matrixplot: You can visualize marker genes using matrixplot. The raw gene expression is scaled and the color map is changed from the default to 'Blues' using `sc.pl.rank_genes_groups_matrixplot(pbmc, n_genes=3, standard_scale='var', cmap='Blues')`.<br><br>3. Tracksplots: The track plot shows the same information as the heatmap, but, instead of a color scale, the gene expression is represented by height. You can use `sc.pl.tracksplot(ad,marker_genes, groupby='louvain')` to visualize the data.<br><br>4. Heatmap and Volcano plot: Each row of a heatmap corresponds to a gene and each column to a single-cell. The brighter the color is the higher is the expression of that gene in a particular cell. Volcano plots show the change in expression on the x-axis (log-fold change) and statistical significance on the y-axis (FDR-corrected p-values). You can color code the genes that have FDR-corrected p-value under 0.01 and log-fold change of over 1.5.<br><br>Before plotting the heatmaps, normalize the data to see the differences in expression between two conditions better.<br><br>Note: The information is incomplete as it does not provide the exact code for creating a heatmap and volcano plot.<br><br>-------------- |

**scRNA-Seq Analysis Case 18.3: Identify Marker Genes**

| | |
|---|---|
| **User’s Inputs** | You are a bioinformatician who specializes in writing code and performing analysis for a variety of bioinformatic data analysis tasks. Now I provide you with an Ubuntu 18.04 system where I save the following data, each line including the data path and data description.<br><br>./examples/case2.1/data/filtered_gene_bc_matrices/hg19: path to 10x mtx data<br><br>I need you to help me plan what needs to be done in each step of the analysis process, and help me write the code for each step to install softwares, use tools and accomplish the following main goal:<br><br>Use scanpy to identify top5 marker genes. |
| **Generated Plans by AutoGPT** | # Step 1<br><br>### Action executed:<br>Web search for 'how to install Ubuntu 18.04'<br>### Function Call:<br>```javascript<br>web_search({<br>"queries": [<br>"how to install Ubuntu 18.04"<br>]<br>})<br>```<br>### Result<br>```<br>Found the following result for the web search: 'how to install Ubuntu 18.04'<br>--------------<br>1. Boot the system from the USB. A Welcome screen will appear with two options: Try Ubuntu directly from the CD, without installing or Install Ubuntu.<br>2. Click the Install Ubuntu button to start the installation wizard.<br>3. Choose a language for the keyboard layout and click on Continue.<br>4. Type in the required information, such as username and password for the root user, then hit Continue.<br>5. The official installation begins.<br>6. Once the installation is complete, remove the USB and restart the system.<br>7. If the Ubuntu screen appears after restarting, the installation was successful.<br><br>Note: The text does not provide information on how to boot the system from the USB, nor does it detail the steps between choosing the keyboard layout and entering the root user information.<br><br>--------------<br><br>```<br>## Step 2<br><br>### Action executed:<br>Web search for 'how to install Python on Ubuntu 18.04' |

### Function Call:
```javascript
web_search({
  "queries": [
    "how to install Python on Ubuntu 18.04"
  ]
})
```
Result
```
Found the following result for the web search: 'how to install Python on Ubuntu 18.04'
--------------
To install Python on Ubuntu 18.04, follow these steps:

1. Make sure your system is running Ubuntu 18.04 and you have a user account with sudo privileges. Access to a terminal window/command-line (Ctrl–Alt–T) is also required.

2. Update your system and refresh repository lists by running the command: `sudo apt update`.

3. Install Python 3.8.3 using the apt package manager. The Python 3.8.3 package is available through a PPA. To add the PPA and make apt aware of the new package, run the following command: `add-apt-repository ppa:deadsnakes/ppa -y`.

4. Now, to install Python 3.8.3, run this command: `apt install python3.8 -y`.

5. Verify the installation by running a command to drop into a Python shell and observe the version reported.

Please note that the information might be incomplete as the command to verify the installation was not fully provided.

--------------

```
Step 3

Action executed:
Web search for 'how to install scanpy on Ubuntu 18.04'
Function Call:
```javascript
web_search({
  "queries": [
    "how to install scanpy on Ubuntu 18.04"
  ]
})
```
Result
```
```

Found the following result for the web search: 'how to install scanpy on Ubuntu 18.04'

To install scanpy on Ubuntu 18.04, you can use either pip or conda. Here are the steps:

1. Using pip:
   - Run the command: `pip install scanpy`
   - If you prefer to use PyPI exclusively, run: `pip install 'scanpy[leiden]'`. The extra `[leiden]` installs two packages that are needed for popular parts of scanpy but aren’t requirements: igraph and leiden.

2. Using conda:
   - If you do not have a working installation of Python 3.6 (or later), consider installing Miniconda.
   - Then run: `conda install -c conda-forge scanpy python-igraph leidenalg`

After installation, you can check if scanpy is installed by opening Python 3 interpreter from the terminal by running `python` and then `import scanpy as sc`.

Please note that the information might be incomplete as it's extracted from different webpages and might not cover all the steps or requirements for the installation.

```
## Step 4

### Action executed:
Web search for 'how to load 10x mtx data into scanpy'
### Function Call:
```javascript
web_search({
  "queries": [
    "how to load 10x mtx data into scanpy"
  ]
})
```
Result
```
Found the following result for the web search: 'how to load 10x mtx data into scanpy'
--------------
To load 10x mtx data into Scanpy, you can use the function `scanpy.read_10x_mtx`. The function takes several parameters:

- `path`: The path to the directory containing the .mtx file.
- `var_names`: This can be set to 'gene_symbols' to use gene symbols for variable names.
- `make_unique`: If set to True, it makes the variable names unique.
- `cache`: If set to False, it does not cache the data.

- `cache_compression`: This can be set to an empty token if no compression is desired.
- `gex_only`: If set to True, it only considers 'Gene Expression' features.
- `prefix`: This can be set to None if no prefix is desired.

Here is an example of how to use the function:

```python
rnaseq1 = sc.read_10x_mtx("GSE145328_RAW")
```

In this example, "GSE145328_RAW" is the path to the directory containing the .mtx file.

Please note that the information provided might be incomplete as it is based on the given chunks of text.

```
## Step 5

### Action executed:
Web search for 'data preprocessing steps in scanpy'
### Function Call:
```javascript
web_search({
  "queries": [
    "data preprocessing steps in scanpy"
  ]
})
```
Result
```
Found the following result for the web search: 'data preprocessing steps in scanpy'
--------------
1. Variable genes: Scanpy performs a basic transformation on the data matrix to filter highly-variable genes. This is part of the preprocessing steps in Scanpy.

2. Batch-effect correction: Scanpy includes a preprocessing step for batch-effect correction. This is a transformation of the data matrix that is not a tool.

3. Per-cell normalization: Scanpy performs per-cell normalization as part of its preprocessing steps. This is a basic transformation on the data matrix.

4. Preprocessing recipes: Scanpy uses preprocessing recipes as part of its data preprocessing steps. These recipes perform basic transformations on the data matrix.

5. Visual quality control: Scanpy uses `highest_expr_genes()` and `filter_genes_dispersion()` for visual quality control in its basic preprocessing steps.

6. Selection and filtration of cells and genes based on quality metrics: This is a preprocessing step in Scanpy where cells and genes are selected and filtered based on quality metrics.

7. Data normalization: This is a preprocessing step in Scanpy where the data is normalized.

8. Identification of highly variable genes: This is a preprocessing step in Scanpy where highly variable genes are identified.

9. Log transformation: This is a preprocessing step in Scanpy where a log transformation is applied to the data.

10. Cluster annotation: This is an optional preprocessing step in Scanpy where clusters are annotated.

11. Subset cells: This is an optional preprocessing step in Scanpy where cells are subset.

12. Save processed data: This is a preprocessing step in Scanpy where the processed data is saved.

Note: The decimal precision requirement is not applicable as the extracted information does not involve numerical data.

--------------

```
Step 6

Action executed:
Web search for 'exploratory data analysis in scanpy'
Function Call:
```javascript
web_search({
  "queries": [
    "exploratory data analysis in scanpy"
  ]
})
```
Result
```
Found the following result for the web search: 'exploratory data analysis in scanpy'
--------------
Scanpy is a Python package used for exploratory data analysis, particularly for large-scale single-cell gene expression data. It is designed for interactive analyses and is often used with Jupyter Notebooks. Scanpy's implementation is based on Python data science and machine learning

libraries, allowing it to scale to millions of cells. The analysis process with Scanpy is interactive, with decisions and analysis steps depending on the results of previous steps and potentially input from experimental partners.

Scanpy's API is divided into datasets, preprocessing (pp), tools (tl), and corresponding plotting (pl) functions. An example of its use involves a dataset of 2700 peripheral blood mononuclear cells of a healthy donor sequenced on the Illumina NextSeq 500.

Scanpy can be used in conjunction with other tools like 'besca', which is developed by the Bioinformatics and Exploratory Data Analysis (BEDA) team at Roche Pharma Research and Early Development. Besca offers additional useful data structures and Python functions.

Scanpy also allows for label transfers between an annotated scRNA-seq dataset and an unannotated Visium dataset, leveraging the data integration performances of Scanorama for exploratory analysis. However, for the label transfer task, more principled approaches based on cell-type deconvolution are advised for more accurate and interpretable results.

Scanpy provides efficient reimplementations of pre-existing statistical methods and analytical workflows, which can be used to perform general exploratory data analysis and special inferences. It is designed to exploit powerful analytical methods using modern high-performance computing architectures, such as servers and clusters with large amounts of memory, multiple central and graphical processing unit cores, and solid-state drives (SSD) with high-speed data access.

The information provided does not include specific decimal precision data related to Scanpy's exploratory data analysis.

--------------

```
Step 7

Action executed:
Web search for 'how to use tl.rank_genes_groups() in scanpy'
Function Call:
```javascript
web_search({
  "queries": [
    "how to use tl.rank_genes_groups() in scanpy"
  ]
})
```
Result
```
Found the following result for the web search: 'how to use tl.rank_genes_groups() in scanpy'
--------------
The function `tl.rank_genes_groups()` in Scanpy is used to rank genes for characterizing groups. Here is an example of how to use it:

```python
import scanpy as sc
adata = sc.datasets.pbmc68k_reduced()
sc.tl.rank_genes_groups(adata, 'bulk_labels', method='wilcoxon')
sc.pl.rank_genes_groups(adata)
```

The function `tl.rank_genes_groups()` has the following parameters:

- `adata`: The annotated data matrix.
- `groupby`: The key of the observations grouping to consider.
- `use_raw`: Whether to use raw attribute or not.
- `groups`: Subset of groups, e.g. `['g1', 'g2', 'g3']`, to which comparison shall be restricted, or `'all'`.
- `reference`: If `'rest'`, compare each group to the union of the rest of the group. If `'all'`, compare each group to all other groups. If a group identifier, compare with respect to this group.
- `n_genes`: The number of genes to consider.
- `rankby_abs`: Whether to rank by absolute values for ranking.
- `pts`: Whether to compute fraction of points in each group.
- `key_added`: If a key is added, adata.uns[‘key_added’] is a structured array that stores the scores, names, and categories.
- `copy`: Whether to return a copy instead of writing to adata.
- `method`: The method used to compute test statistic. If `'t-test'`, uses t-test, if `'wilcoxon'`, uses Wilcoxon-rank-sum. If `'logreg'`, uses logistic regression.
- `corr_method`: The multiple testing correction method.

You can also specify groups and reference arguments. For example:

```python
sc.tl.rank_genes_groups(adata, 'louvain', groups=['1', '2'], reference='rest')
```

In this case, the reference would include cells from category '3'.

```
## Step 8

### Action executed:
Web search for 'how to extract top 5 marker genes in scanpy'
### Function Call:
```javascript
web_search({
  "queries": [
    "how to extract top 5 marker genes in scanpy"
  ]
})
```
Result
```

```
Found the following result for the web search: 'how to extract top 5 marker genes in scanpy'
--------------
1. Use the `sc.tl.rank_genes_groups` function to rank the genes. For example: `sc.tl.rank_genes_groups(WT_Donuts, 'leiden', method='t-test', use_raw=False)`. This function is used to get the marker genes from clusters.

2. To visualize the top 5 genes, use the `sc.pl.rank_genes_groups` function. For example: `sc.pl.rank_genes_groups(WT_Donuts, n_genes=5, sharey=False)`.

3. If you want to get the top 5 genes that are up-regulated in 'mut' compared to 'ctrl', you can use the following code: `sc.tl.rank_genes_groups(WT_Donuts_clust1, 'condition', groups=['mut'], reference='ctrl', method='wilcoxon', key='mut_up_ctrl_down')` and `sc.pl.rank_genes_groups(WT_Donuts_clust1, n_genes=5, key='mut_up_ctrl_down')`.

4. You can apply more stringent marker gene filters using the `sc.tl.filter_rank_genes_groups` function. For example: `sc.tl.filter_rank_genes_groups(pbmc, min_in_group_fraction=0.5, max_out_group_fraction=0.3, min_fold_change=1.5)`.

5. To visualize the marker genes using matrixplot, use the `sc.pl.rank_genes_groups_matrixplot` function. For example: `axs = sc.pl.rank_genes_groups_matrixplot(pbmc, n_genes=3, standard_scale='var', cmap='Blues')`.

Note: The information provided assumes that you have `adata.obs['condition']` which stores the categories `'mut'` and `'ctrl'`, and that you are interested in `adata.obs['leiden'] == '1'`. Change these values to match your data.

--------------

```
Step 9

Action executed:
Web search for 'how to visualize marker genes in scanpy'
Function Call:
```javascript
web_search({
  "queries": [
    "how to visualize marker genes in scanpy"
  ]
})
```
Result
```
```

Found the following result for the web search: 'how to visualize marker genes in scanpy'

To visualize marker genes in Scanpy, you can use different methods such as dotplots, violin plots, heatmaps, and tracksplot. Here are the steps:

1. Set up a dictionary with the marker genes. This will allow Scanpy to automatically label the groups of genes. For example:
```python
marker_genes_dict = {
'B-cell': ['CD79A', 'MS4A1'],
'Dendritic': ['FCER1A', 'CST3']
}
```
2. To identify differentially expressed genes, run `sc.tl.rank_genes_groups`. This function will compare the distribution of each gene in a group against the distribution in other groups.

3. To visualize the groups of cells that express the marker genes, use the `sc.pl.umap` function. For example:
```python
sc.pl.umap(pbmc, color=['CD79A', 'MS4A1', 'IGJ', 'CD3D', 'FCER1A', 'FCGR3A', 'n_counts', 'bulk_labels'], s=50, frameon=False, ncols=4, vmax='p99')
```
4. To visualize the marker genes after annotating the cell types, use the `sc.pl.dotplot` function. For example:
```python
sc.pl.dotplot(adata, marker_genes, groupby='leiden');
```
5. For a compact violin plot, use the `sc.pl.stacked_violin` function. For example:
```python
sc.pl.stacked_violin(adata, marker_genes, groupby='leiden', rotation=90);
```
6. To visualize the co-expression of two marker genes in single cells on a UMAP, use the `sc.tl.score_genes` and `sc.pl.umap` functions. For example:
```python
gene_list = ['MS4A1', 'CD79A']
sc.tl.score_genes(adata, gene_list, score_name='MS4A1_CD79A')
sc.pl.umap(adata, color='MS4A1_CD79A')
```

Note: Scanpy draws all plots by setting `use_raw=True`. This might not be the best method if the raw data was not regressed out with mitochondrial genes, gene counts, cell cycle scores, etc.

```

## Step 10
```

Action executed:
Web search for 'how to write reproducible code in Python'
Function Call:
```javascript
web_search({
  "queries": [
    "how to write reproducible code in Python"
  ]
})
```
Result
```
Found the following result for the web search: 'how to write reproducible code in Python'
--------------
1. Organize your Python code properly.
2. Package your code with setuptools.
3. Perform unit testing with unittest/pytest.
4. Ensure code quality with flake8.
5. Document your code with Sphinx.
6. Automate processes with Tox.
7. Use continuous integration with Travis-CI.
8. Host your documentation with ReadTheDocs.
9. Implement command line interfaces using Click.
10. Set up Numpy random seed to 150 to ensure consistent results.
11. Write reproducible and maintainable Jupyter notebooks.
12. Prioritize decimal precision, aim for answers with 3 decimal places, if possible; if not settle for 2 decimal places. Only take 1 decimal or rounded numbers when absolutely necessary.

(Note: Some information might be missing, such as how to specifically organize Python code, how to use the mentioned tools, etc.)

--------------
```

scRNA-Seq Analysis   Case 18.4: Clustering and Visualization

**User's Inputs**	You are a bioinformatician who specializes in writing code and performing analysis for a variety of bioinformatic data analysis tasks. Now I provide you with an Ubuntu 18.04 system where I save the following data, each line including the data path and data description.<br><br>./examples/case2.1/data/filtered_gene_bc_matrices/hg19: path to 10x mtx data<br><br>I need you to help me plan what needs to be done in each step of the analysis process, and help me write the code for each step to install softwares, use tools and accomplish the following main goal:<br><br>Use scanpy to perform clustering and visualize the expression level of gene PPBP in the UMAP.
**Generated Plans by AutoGPT**	Error: Executing commands is not supported in this application environment.

<table>
<tr>
<td>User’s Inputs</td>
<td>You are a bioinformatician who specializes in writing code and performing analysis for a variety of bioinformatic data analysis tasks. Now I provide you with an Ubuntu 18.04 system where I save the following data, each line including the data path and data description.

slice1.h5ad: spatial transcriptomics data for slice 1 in AnnData format

I need you to help me plan what needs to be done in each step of the analysis process, and help me write the code for each step to install softwares, use tools and accomplish the following main goal:

Use squidpy for neighborhood enrichment analysis</td>
</tr>
<tr>
<td>Generated Plans by AutoGPT</td>
<td>## Step 1

Action executed:
Web search for 'how to install python and pip on Ubuntu 18.04'
Function Call:
```javascript
web_search({
  "queries": [
    "how to install python and pip on Ubuntu 18.04"
  ]
})
```
Result
```
Found the following result for the web search: 'how to install python and pip on Ubuntu 18.04'

To install Python 2 and pip for Python 2 on Ubuntu 18.04, follow these steps:

1. Update the package index by running the following command: `sudo apt update`
2. Install pip for Python 2 with: `sudo apt install python-pip`. This command will also install Python2 and all the dependencies required for building Python modules.
3. Verify the installation by printing the pip version number: `pip --version`

To install Python 3 and pip for Python 3 on Ubuntu 18.04, follow these steps:

1. Start by updating the package list using the following command: `sudo apt update`
2. Use the following command to install pip for Python 3: `sudo apt install python3-pip`. This command will also install all the dependencies required for building Python modules.
3. Once the installation is complete, verify the installation by checking the pip version with: `pip3 --version`

Note: The version number may vary.</td>
</tr>
</table>

```
## Step 2

### Action executed:
Web search for 'how to install numpy, pandas, scanpy, and squidpy using pip'
### Function Call:
```javascript
web_search({
  "queries": [
    "how to install numpy, pandas, scanpy, and squidpy using pip"
  ]
})
```
Result
```
Found the following result for the web search: 'how to install numpy, pandas, scanpy, and squidpy using pip'
--------------
To install numpy, pandas, scanpy, and squidpy using pip, you can use the following commands:

1. Numpy and Pandas are not explicitly mentioned in the provided text. However, they are typically installed using the following commands:
   - `pip install numpy`
   - `pip install pandas`

2. For Scanpy:
   - `pip install scanpy`
   - Alternatively, for additional packages: `pip install 'scanpy[leiden]'`

3. For Squidpy:
   - `pip install squidpy`
   - Alternatively, for interactive features: `pip install 'squidpy[interactive]'`
   - To install Squidpy from GitHub: `pip install git+https://github.com/scverse/squidpy@main`

Please note that the installation of numpy and pandas is assumed based on common practices as they were not mentioned in the provided text.

--------------

```
Step 3

Action executed:
Web search for 'how to load spatial transcriptomics data in Python'
Function Call:
```javascript
web_search({
  "queries": [

    "how to load spatial transcriptomics data in Python"
  ]
})
```
Result
```
Found the following result for the web search: 'how to load spatial transcriptomics data in Python'
--------------
To load spatial transcriptomics data in Python, you can use the Scanpy library. Here are the steps:

1. Import the necessary libraries:
```python
import scanpy as sc
import pandas as pd
import matplotlib.pyplot as plt
import seaborn as sns
```

2. Set the logging and figure parameters:
```python
sc.logging.print_versions()
sc.set_figure_params(facecolor="white", figsize=(8, 8))
sc.settings.verbosity = 3
```

3. Load the data. The example given uses a Visium spatial transcriptomics dataset of the human lymphnode, which is publicly available from the 10x genomics website. The exact method to load the data is not provided in the given text.

Please note that the information provided is incomplete and does not provide the exact method to load the data.

```
## Step 4

### Action executed:
Web search for 'how to perform neighborhood enrichment analysis using squidpy'
### Function Call:
```javascript
web_search({
  "queries": [
    "how to perform neighborhood enrichment analysis using squidpy"
  ]
})
```
Result
```

Found the following result for the web search: 'how to perform neighborhood enrichment analysis using squidpy'

To perform neighborhood enrichment analysis using Squidpy, follow these steps:

1. Import Squidpy:
```python
import squidpy as sq
```

2. Load your dataset. In this example, the dataset is loaded using the `sq.datasets.visium_flu` function:
```python
adata = sq.datasets.visium_flu
```

3. Visualize cluster annotation in spatial context with `squidpy.pl.spatial_scatter()`. This step is optional but can help you understand your data better:
```python
sq.pl.spatial_scatter(adata, shape=None, color="cluster", size=1)
```

4. Compute the neighborhood enrichment score using the `squidpy.gr.nhood_enrichment()` function. The `cluster_key` parameter should be set to the key of the clusters in your data:
```python
sq.gr.nhood_enrichment(adata, cluster_key="leiden")
```

5. Visualize the results with `squidpy.pl.nhood_enrichment()`. This step is optional but can help you understand the results better:
```python
fig, ax = plt.subplots(1, 2, figsize=(13, 7))
sq.pl.nhood_enrichment(
  adata,
  cluster_key="leiden",
  figsize=(8, 8),
  title="Neighborhood enrichment adata",
  ax=ax[0],
)
sq.pl.spatial_scatter(adata_subsample, color="leiden", shape=None,
size=2, ax=ax[1])
```

Please note that the `cluster_key` parameter should be replaced with the key of the clusters in your data. The `adata_subsample` in the last line should be replaced with your subsampled data.

```
```

Step 5

Action executed:
Web search for 'how to visualize results of neighborhood enrichment analysis in Python'
Function Call:
```javascript
web_search({
  "queries": [
    "how to visualize results of neighborhood enrichment analysis in Python"
  ]
})
```
Result
```
Found the following result for the web search: 'how to visualize results of neighborhood enrichment analysis in Python'
--------------
To visualize the results of neighborhood enrichment analysis in Python, you can use the `squidpy` library. Here are the steps:

1. Import the library: `import squidpy as sq`
2. Load your data: `adata = sq.datasets.visium_flu`
3. Calculate the neighborhood enrichment score: `sq.gr.nhood_enrichment(adata, cluster_key="leiden")`
4. Visualize the results using the following code:

```python
fig, ax = plt.subplots(1, 2, figsize=(13, 7))
sq.pl.nhood_enrichment(
  adata,
  cluster_key="leiden",
  figsize=(8, 8),
  title="Neighborhood enrichment adata",
  ax=ax[0],
)
sq.pl.spatial_scatter(adata_subsample, color="leiden", shape=None, size=2, ax=ax[1])
```

Please note that the dataset should contain cell type annotations in `anndata.Anndata.obs` which are used for the calculation of the neighborhood enrichment.

```

Spatial Transcriptomics **Case 19.2: Single-cell mapping**

**User's Inputs**	You are a bioinformatician who specializes in writing code and performing analysis for a variety of bioinformatic data analysis tasks. Now I provide you with an Ubuntu 18.04 system where I save the following data, each line including the data path and data description.<br><br>slice1.h5ad: spatial transcriptomics data for slice 1 in AnnData format<br>sc.h5ad: single cell data in the same tissue for slice 1 in AnnData format<br><br>I need you to help me plan what needs to be done in each step of the analysis process, and help me write the code for each step to install softwares, use tools and accomplish the following main goal:<br><br>Use Tangram to map single-cell data to spatial transcriptomics data.
**Generated Plans by AutoGPT**	## Step 1<br><br>### Action executed:<br>Agent message<br>Based on the goal, here is the plan and the code snippets for each step:<br><br>**Step 1: Install Required Software**<br>- Install Ubuntu 18.04 system (if not already installed)<br>- Install necessary dependencies for Tangram and other required tools<br><br>```bash<br># Update the system<br>sudo apt-get update<br><br># Install pip for Python 3<br>sudo apt-get install python3-pip<br><br># Install necessary Python packages<br>pip3 install anndata pandas numpy scipy matplotlib seaborn scanpy tangram<br>```<br><br>**Step 2: Download and Prepare Data**<br>- Download the "slice1.h5ad" file containing spatial transcriptomics data<br>- Download the "sc.h5ad" file containing single-cell data for the same tissue<br>- Load the data files into the analysis environment<br><br>```python<br># Import necessary packages<br>import anndata<br><br># Load the data<br>adata_slice1 = anndata.read_h5ad('path_to_your_data/slice1.h5ad')<br>adata_sc = anndata.read_h5ad('path_to_your_data/sc.h5ad')<br>```<br><br>**Step 3: Data Preprocessing and Quality Control**<br>- Perform quality control on the single-cell data and spatial transcriptomics data

- Filter out low-quality cells and genes
- Normalize the data to account for differences in sequencing depth

```python
# Import necessary packages
import scanpy as sc

# Perform quality control and normalization
sc.pp.filter_cells(adata_sc, min_genes=200)
sc.pp.filter_genes(adata_sc, min_cells=3)
sc.pp.normalize_total(adata_sc, target_sum=1e4)
sc.pp.log1p(adata_sc)
sc.pp.highly_variable_genes(adata_sc, min_mean=0.0125, max_mean=3,
min_disp=0.5)
adata_sc.raw = adata_sc
adata_sc = adata_sc[:, adata_sc.var.highly_variable]
```

Step 4: Data Integration
- Use Tangram or other suitable tools to integrate the single-cell data with the spatial transcriptomics data
- Map the single-cell data onto the spatial transcriptomics data based on spatial coordinates

```python
# Import necessary packages
import tangram as tg

# Map single-cell data to spatial transcriptomics data
mapper = tg.MapCells(adata_sc, adata_slice1)
mapper.map_cells()
```

Step 5: Visualization and Analysis
- Visualize the integrated data to explore the spatial distribution of gene expression in the tissue
- Perform downstream analysis such as clustering, differential expression analysis, or spatial gene expression analysis

```python
# Visualize the mapped data
mapper.plot_spatial_transformation()
```

Step 6: Interpretation and Reporting
- Interpret the results of the analysis and draw conclusions
- Generate visualizations and reports to communicate the findings

Please note that the code snippets provided are placeholders and may need to be modified based on the specific tools and programming languages you are using for your bioinformatic analysis.

Proteomics   Case 20.1: protein expression quantification

<table>
<tr>
<td>User's Inputs</td>
<td>You are a bioinformatician who specializes in writing code and performing analysis for a variety of bioinformatic data analysis tasks. Now I provide you with an Ubuntu 18.04 system where I save the following data, each line including the data path and data description.

01CPTAC_COprospective_W_PNNL_20170123_B1S1_f01.mzML.gz: protein mass spectrometry data in mzML format, tumor samples fraction 1
01CPTAC_COprospective_W_PNNL_20170123_B1S1_f02.mzML.gz: protein mass spectrometry data in mzML format, tumor samples fraction 2
01CPTAC_COprospective_W_PNNL_20170123_B1S1_f03.mzML.gz: protein mass spectrometry data in mzML format, tumor samples fraction 3
98CPTAC_COprospective_W_VU_20160804_11CO047_f01.mzML.gz: protein mass spectrometry data in mzML format, normal samples fraction 1
98CPTAC_COprospective_W_VU_20160804_11CO047_f02.mzML.gz: protein mass spectrometry data in mzML format, normal samples fraction 2
98CPTAC_COprospective_W_VU_20160804_11CO047_f03.mzML.gz: protein mass spectrometry data in mzML format, normal samples fraction 3
UP000005640_9606.fasta: reference protein sequence from UniProt

I need you to help me plan what needs to be done in each step of the analysis process, and help me write the code for each step to install softwares, use tools and accomplish the following main goal:

identify proteins in tumor and normal samples based on mass spectrometry data</td>
</tr>
<tr>
<td>Generated Plans by AutoGPT</td>
<td>## Step 1

Action executed:
Web search for 'software for protein identification from mass spectrometry data'
Function Call:
```javascript
web_search({
  "queries": [
    "software for protein identification from mass spectrometry data"
  ]
})
```
Result
```
Found the following result for the web search: 'software for protein identification from mass spectrometry data'
----
1. "Mass-Up" - An open-source utility for proteomics designed to support the preprocessing and analysis of MALDI-TOF mass spectrometry data. It loads data from mzML, mzXML, and CSV files and allows users to apply baseline correction, normalization, smoothing, peak detection, and peak matching.</td>
</tr>
</table>

2. "PIQMIe" - A web-based software for proteomics identifications/quantitations data management. It includes peptide mapping, error tolerant search, and disulfide analysis.

3. "Spectromania" - A proprietary software for analysis and visualization of mass spectrometric data.

4. "StavroX" - A freeware software to identify cross-linked peptides from mass spectrometric data. It can be used for a wide variety of cross linkers and proteases used in the cross-linking MS experiment.

5. "MassMatrix" - A freeware database search algorithm for tandem mass spectrometric data. It uses a mass accuracy sensitive probabilistic scoring model to rank peptide and protein matches.

6. "MassWiz" - An open-source search algorithm developed at the Institute of Genomics and Integrative Biology.

7. "Proteome Discoverer" - A software recommended for iterative search strategies, multi-search engine comparison, TMT SPS MS3, and glycopeptide identification and quantification. It helps identify and quantify proteins in complex biological samples.

8. "ProteoWizard toolkit" - A set of libraries and applications for proteomics research. It includes tools like Skyline for Multiple Reaction Monitoring methods and analysis of mass spectrometer data, idConvert, msaccess, and msconvert.

9. "idconvert" - A tool to convert between various file formats.

10. "LA-iMageS" - A tool to produce 2D and 3D images. (Incomplete information)

Note: The information about some software is incomplete, such as their specific functionalities and features.

```
## Step 2

### Action executed:
Web search for 'how to preprocess mass spectrometry data'
### Function Call:
```javascript
web_search({
  "queries": [
    "how to preprocess mass spectrometry data"
  ]
})
```
Result
```

Found the following result for the web search: 'how to preprocess mass spectrometry data'
----
1. Loading the Data: Mass spectrometry data can be stored in different formats. If the data is stored in text files with two columns (the mass/charge (M/Z) ratios and the corresponding intensity values), you can use one of the following MATLAB® I/O functions: `importdata`, `dlmread`, or `textscan`. Alternatively, if the data is stored in JCAMP-DX formatted files, you can use the function `jcampread`. If the data is contained in a spreadsheet of an Excel® workbook, you can use the function `xlsread`. If the data is stored in mzXML format, you can use appropriate functions to read it.

2. Reading raw data: Load raw mass/charge and ion intensity data from comma-separated-value (CSV) files, or read a JCAMP-DX-formatted file with mass spectrometry data into the MATLAB® environment. You can also have data in TXT files and use the `importdata` function.

3. Preprocessing raw data: Resample high-resolution data to a lower resolution where the extra data points are not needed. Correct the baseline of the data.

4. LC-MS data preprocessing: This includes steps of noise filtering, deisotoping, peak detection, RT alignment, peak matching, and normalization. These data preprocessing steps generate a list of detected peaks characterized by their RTs, m/z values, and intensities. The preprocessed data can be used in subsequent analysis, e.g., identification of significant differences between groups.

Note: The information is incomplete as it does not provide detailed steps or methods for each preprocessing step.

```
## Step 3

### Action executed:
Web search for 'database search tools for mass spectrometry data'
### Function Call:

```javascript
web_search({
  "queries": [
    "database search tools for mass spectrometry data"
  ]
})
```

Result
```
Found the following result for the web search: 'database search tools for mass spectrometry data'
--------------
1. Database Search Tool: QUEST
2. Database Search Tool: MS-GF+

3. Database Search Tool: ProXL - Analyze, Visualize, & Share Protein Cross-linking Data
4. Database Search Tool: Scaffold - download free viewer to read Scaffold (.sfd) files
5. Database Search Tool: Skyline – open source software for analysis of SRM/MRM/PRM/DIA/SWATH data
6. Database Search Tool: XlinkDB
7. Database Search Tool: BLAST - NCBI Basic Local Alignment Search Tool
8. Database Search Tool: ChemCalc - molecular mass calculator
9. Database Search Tool: Comet - an open source tandem mass spectrometry (MS/MS) sequence database search tool
10. Database Search Tool: Delta Mass - database of protein post-translational modifications
11. Database Search Tool: Entrez - NCBI database search and retrieval system
12. Database Search Tool: ExPASY - proteomics tools
13. Database Search Tool: IonSource - mass spectrometry tools and resources
14. Database Search Tool: NEWT - taxonomy database browser
15. Database Search Tool: PepNovo - de novo sequencing of low precision MS/MS data
16. Database Search Tool: Proteomics Tools
17. Database Search Tool: ProteomicsTools - Software for the post-analysis of MASCOT, SEQUEST, Comet, XTandem, PFind, PeptidePhophet, MyriMatch, MSGF, OMSSA, MSAmanda or Percolator database search result
18. Database Search Tool: ProteoWizard - Link library and tools that are a set of modular and extensible open-source, cross-platform tools and software libraries that facilitate proteomics data analysis
19. Database Search Tool: ProteoWorker
20. Database Search Tool: MSGF+ - for analysis of mass spectrometry data
21. Database Search Tool: MSPLIT - spectral library search
22. Database Search Tool: MODa - open modification search
23. Database Search Tool: Maestro - spectral networks search
24. Database Search Tool: MSPLIT-DIA.

--------------

```
Step 4

Action executed:
Web search for 'how to quantify proteins from mass spectrometry data'
Function Call:
```javascript
web_search({
  "queries": [
    "how to quantify proteins from mass spectrometry data"
  ]
})
```
```

Result
```
Found the following result for the web search: 'how to quantify proteins from mass spectrometry data'
--------------
1. Quantification of proteins from mass spectrometry data can be done by monitoring ion currents derived from the intact, protonated, peptide ions. Product ions generated by MS-MS can also be used for quantification, although this increases selectivity at the expense of sensitivity.

2. MALDI (Matrix-Assisted Laser Desorption/Ionization) is a precise and accurate approach to protein quantification. The mass spectrometric immunoassay, which combines immunoaffinity column capture with MALDI detection, is a widely adopted method.

3. Isotope-labeled versions of the target proteolytic peptides are typically added as an internal standard for quantification. Monitoring more than one MS-MS transition for each target species provides a powerful approach to quantify a predetermined set of proteins for multiple samples.

4. The targeted quantitative strategy consists of three major components: generation of stable isotope labeled peptides/proteins as references for the corresponding targeted protein; an effective sample preparation protocol to reduce sample complexity and enrich the targeted surrogates; a data-driven highly specific mass spectrometry platform for candidate-based analysis.

5. Quantification of intermediate-abundance proteins in serum can be done by multiple reaction monitoring mass spectrometry in a single-quadrupole ion trap.

6. MaxQuant software can be used to identify and quantify proteins from the DDA (Data Dependent Acquisition) data. It reports an intensity-based absolute quantification (iBAQ) value, a measure of protein abundance. The relative iBAQ (riBAQ), which is the iBAQ for a protein or protein group divided by all non-contaminant, non-redundant proteins, can also be calculated.

7. The output of the quantification process is a matrix with a list of proteins and their corresponding abundances in the respective samples, filtered using false-discovery rate cut-offs.

Note: The information is incomplete as it does not provide specific steps or procedures for quantifying proteins from mass spectrometry data.

--------------

```
Step 5

Action executed:
Web search for 'data analysis tools for mass spectrometry data'
Function Call:
```javascript
```

	web_search({<br>"queries": [<br>"data analysis tools for mass spectrometry data"<br>]<br>})<br>```<br>### Result<br>```<br>Found the following result for the web search: 'data analysis tools for mass spectrometry data'<br>--------------<br>1. OpenMS: A set of 185 tools and workflows for mass spectrometric data processing tasks. It provides a well-defined application programming interface in C++ and Python and uses standardized open data formats.<br><br>2. Mass-Up: An open-source utility for proteomics designed to support the preprocessing and analysis of MALDI-TOF mass spectrometry data. It loads data from mzML, mzXML, and CSV files and allows users to apply baseline correction, normalization, smoothing, peak detection, and peak matching.<br><br>3. Spectronaut: A proprietary commercial software for quantitative proteomics developed by Biognosys AG. It allows the targeted analysis of data independent acquisition (DIA) data sets for label-free peptide quantitation, also called SWATH acquisition.<br><br>4. OpenChrom: An open-source software for chromatography and mass spectrometry that can be extended using plug-ins. It has converters for the native access of various data files, including mzXML, netCDF, Agilent, Finnigan, and Varian file formats.<br><br>5. ORIGAMI: An open-source software suite for the analysis of mass spectrometry data.<br><br>6. mineXpert: An open-source software for mass spectral data visualization/mining. It supports ion mobility mass spectrometry.<br><br>7. mMass: An open-source multi-platform package of tools for mass spectrometric data analysis and interpretation written in Python.<br><br>8. MassWiz: A tool for peptide identification and FDR calculation.<br><br>9. MS Amanda: An algorithm optimized for high accuracy tandem mass spectra.<br><br>10. MSFragger: A tool for ultrafast database search for peptides.<br><br>11. Thermo Scientific Proteomics Software: A suite of software programs specifically designed to mine complex, high-resolution accurate-mass (HRAM) Orbitrap data.<br><br>12. Compound Discoverer: A software platform that relies on descriptive statistics, data reduction, and data visualization for data mining analysis.

13. patRoon: A software platform that uses descriptive statistics, data reduction, and data visualization for data mining analysis.

14. MetFrag: A platform for further annotation of processed data.

15. SIRIUS: A platform for further annotation of processed data.

Note: Some information might be incomplete.

```
## Step 6

### Action executed:
Web search for 'how to visualize mass spectrometry data'
### Function Call:
```javascript
web_search({
  "queries": [
    "how to visualize mass spectrometry data"
  ]
})
```
Result
```
Found the following result for the web search: 'how to visualize mass spectrometry data'
--------------
1. A set of tools has been developed that includes a database and a visualization component to collect and monitor LC-MS/MS data. This toolkit can be used to increase quality control for complex LCMS assays.

2. The toolkit includes a parsing tool built with Python and standard libraries to parse XML files after each clinical run. The parsed information is stored in a database comprised of three distinct tables.

3. The toolkit also includes an interactive data visualization tool that uses the data from the database. There are 5 different visualizations that present the data based on interchangeable parameters.

4. BatMass is another mass-spectrometry data visualization tool, with a focus on being fast and interactive. It supports the open standard mzML and mzXML mass spectrometry data types and offers viewer synchronization.

5. BatMass allows for viewing MS1 data in one viewer and MS2 data in another. Clicking on a point on the chromatogram brings up a graphical representation of the selected MS1 spectrum and raw file metadata associated with it.

6. vMS-Share is a web application that offers versatile frameworks for data visualization and mining. It enables direct and facile analysis of raw mass spectra and manual evaluation of significant findings.

7. vMS-Share provides interactive and user-friendly visualizations to the user via a variety of easily accessible search filters.

8. For viewing identifications within the experiment, hovering with the mouse pointer above a point on the chromatogram will bring up a graphical representation of the selected MS1 spectrum and raw file metadata associated with it.

9. In case of MS2 data, the selected MS1 spectrum will also contain a tab for viewing MS2 spectra associated with it. The MS2 spectra tab contains a table ordered by retention times which are used as links to view each MS2 spectrum and the corresponding raw file metadata.

10. The experiment image consists of an interactive chromatogram viewer with flagged identification points coupled with an MS spectrum viewer and a table of selected identifications. Chromatogram and spectrum viewers offer zoom in/out capabilities.

Note: The information is incomplete and does not provide specific steps on how to visualize the data.

--------------

```
```

Step 7

Action executed:
Web search for 'how to document and report mass spectrometry data analysis'
Function Call:
```javascript
web_search({
  "queries": [
    "how to document and report mass spectrometry data analysis"
  ]
})
```
Result
```
Found the following result for the web search: 'how to document and report mass spectrometry data analysis'
--------------
1. Use the MIAPE-MSI (Minimum Information About a Proteomics Experiment - Mass Spectrometry Informatics) to document and report mass spectrometry data analysis. This includes a checklist of information about mass spectrometry according to the type of analysis performed (e.g. MS, MS/MS, LC-MS).
```

	2. Spectra data are usually stored in a text file managed by file systems, and meta information is generally treated in separate files. However, there is a need for a uniform and widely accepted access and manipulation strategy for such large datasets, especially when information from a single experiment on a specified MS platform has to be shared and validated among different laboratories.<br><br>3. Companies that produce software tools for MS analysis provide modules designed to support all experimental phases.<br><br>4. The PSI (Proteomics Standards Initiative) is developing the mzQuantML and mzTab formats to support SRM (Selected Reaction Monitoring) results in addition to the results of many other kinds of quantitative proteomics analyses.<br><br>5. A new data type that has emerged is one that can contain a set of previously identified spectra. In a typical workflow, the identification results from a shotgun analysis are combined into consensus spectra and those spectra stored with their peak annotations and other related information.<br><br>6. It is common to convert the binary mass spectrometer output files into simple text files containing only the MS/MS spectra. One of the first such formats was the simple dta format wherein each spectrum was written to a separate file containing one header line for the known or assumed charge and the mass of the precursor peptide ion, calculated from the measured m/z and the charge.<br><br>7. It is encouraged that analyses be performed and reported using all the PSI formats: TraML for transition lists or inclusion lists, mzML for mass spectrometer output results, mzIdentML for encoding the results of database searching and validation, and mzQuantML for encoding the quantitative results of the analysis.<br><br>8. The mass spectrometry functions preprocess and classify raw data from SELDI-TOF and MALDI-TOF spectrometers and use statistical learning functions to identify patterns. Load raw mass/charge and ion intensity data from comma-separated-value files.<br><br>9. The solution to make integration or comparison of mass spectrometer output data easier is to create open file formats that can encode the information in these output files in XML (Extensible Markup Language) and then write software to read and write these formats. A unified format called mzML emerged.<br><br>10. The processing of mass spectrometry data may involve semi-automatic computational analysis of the recorded mass spectra and sometimes also of the associated metadata (e.g., elution characteristics if the instrument is coupled to a chromatography system). The result of the processing can be assigned a score, rank or confidence measure.<br><br>--------------

Metabolomics Case 21.1: metabolites concentration

<table>
<tr><td>User's Inputs</td><td>You are a bioinformatician who specializes in writing code and performing analysis for a variety of bioinformatic data analysis tasks. Now I provide you with an Ubuntu 18.04 system where I save the following data, each line including the data path and data description.

B1S1_f01.mzML.gz: LC-MS mass spectrometry data of metabolites in mzML format, tumor samples fraction 1
B1S1_f02.mzML.gz: LC-MS mass spectrometry data of metabolites in mzML format, tumor samples fraction 2
B1S1_f03.mzML.gz: LC-MS mass spectrometry data of metabolites in mzML format, tumor samples fraction 3
11CO047_f01.mzML.gz: LC-MS mass spectrometry data of metabolites in mzML format, normal samples fraction 1
11CO047_f02.mzML.gz: LC-MS mass spectrometry data of metabolites in mzML format, normal samples fraction 2
11CO047_f03.mzML.gz: LC-MS mass spectrometry data of metabolites in mzML format, normal samples fraction 3

I need you to help me plan what needs to be done in each step of the analysis process, and help me write the code for each step to install softwares, use tools and accomplish the following main goal:

calculate metabolites concentration in tumor and normal samples based on LC-MS mass spectrometry data</td></tr>
<tr><td>Generated Plans by AutoGPT</td><td>## Step 1

Action executed:
Web search for 'how to install OpenMS on Ubuntu 18.04'
Function Call:
```javascript
web_search({
  "queries": [
    "how to install OpenMS on Ubuntu 18.04"
  ]
})
```
Result
```
Found the following result for the web search: 'how to install OpenMS on Ubuntu 18.04'
----
1. Update your system to the latest version by running the following commands:
   ```
   apt-get update -y
   apt-get upgrade -y
   ```
   Once your server is updated, restart it to apply the changes.

2. Install Java as OpenNMS is written in Java. You can install Java JDK by running the following command:</td></tr>
</table>

```
apt install default-jdk
```

3. Download the OpenNMS GPG key and add it using the following command:
```
wget -O - http://debian.opennms.org/OPENNMS-GPG-KEY | apt-key add -
```

4. Add the OpenNMS repository to the APT source list file with the following command:
```
nano /etc/apt/sources.list
```
Add the following lines to the end of the file:
```
deb http://debian.opennms.org stable main
deb-src http://debian.opennms.org stable main
```

5. Install PostgreSQL as OpenNMS uses the PostgreSQL database to store its data. PostgreSQL is available by default in the Ubuntu 18.04 repository. You can install it by simply running the following command:
```
apt-get install postgresql -y
```

6. Install OpenNMS and all other required packages by running the command below:
```
apt install opennms
```

7. During the installation, you will receive a notification that in order to complete the install and setup of OpenNMS, you need to configure the OpenNMS database.

8. After the installation, OpenNMS will be available on HTTP port 8980 by default. Open your favorite browser and navigate to `http://your-domain.com:8980` or `http://server-ip-address:8980`. The default username and password is **admin**/**admin**.

Note: The information about how to configure the OpenNMS database is missing.

```
## Step 2

### Action executed:
```

Web search for 'how to install XCMS on Ubuntu 18.04'
Function Call:
```javascript
web_search({
  "queries": [
    "how to install XCMS on Ubuntu 18.04"
  ]
})
```
Result
```
Found the following result for the web search: 'how to install XCMS on Ubuntu 18.04'
--------------
To install XCMS on Ubuntu 18.04, follow these steps:

1. Install the necessary dependencies by typing the following in a bash shell:
   ```
   sudo apt-get install libnetcdf-dev build-essential
   ```
2. Download the latest xcms_*.tar.gz file from http://www.bioconductor.org/packages/release/bioc/html/xcms.html
3. Navigate to the directory where you downloaded the file (e.g., type `cd ~/Downloads` if that is where you saved it)
4. Run R as root by typing `sudo R`
5. At the R prompt, type: `install.packages("./xcms_1.23.1.tar.gz")`

Alternatively, if you are using R version "4.3", you can install XCMS using Bioconductor:

1. Start R and enter the following:
   ```
   if (!require("BiocManager", quietly = TRUE))
   install.packages("BiocManager")

   BiocManager::install("xcms")
   ```

Please note that the version of the xcms package (e.g., "xcms_1.23.1.tar.gz") may vary depending on the latest release. Always check the official website for the most recent version.

--------------

```
Step 3

Action executed:
Web search for 'how to install mzR on Ubuntu 18.04'
Function Call:
```javascript
web_search({
```

  "queries": [
    "how to install mzR on Ubuntu 18.04"
  ]
})
```
### Result
```
Found the following result for the web search: 'how to install mzR on Ubuntu 18.04'

To install mzR on Ubuntu 18.04, follow these steps:

1. Open R and use the following commands:
   ```
   > source("http://bioconductor.org/biocLite.R")
   > biocLite("mzR")
   ```
2. If you encounter an error like 'shared object ‘mzR.so’ not found', you can try to install your package from the command line like so:
   ```
   R CMD INSTALL --no-multiarch /path/to/yourPackage
   ```
3. Note that `mzR` requires a more recent version of `R` and Bioconductor; `mzR` has been available since BioC 2.9 (R-2.14). In general, it is advised to use the latest `R` for Bioc usage.

Please note that the information might be incomplete as the chunks provided do not provide a full guide on how to install mzR on Ubuntu 18.04.

```
## Step 4

### Action executed:
Web search for 'how to uncompress mzML.gz files'
### Function Call:
```javascript
web_search({
  "queries": [
    "how to uncompress mzML.gz files"
  ]
})
```
Result
```
Found the following result for the web search: 'how to uncompress mzML.gz files'
--------------
To uncompress mzML.gz files, you can use the following steps:

1. Download and install MZmine2. You can find it on the MZmine2 website.

2. Open MZmine2 and import your .mzML.gz files. You can do this by going to the Raw data methods in the top menu and selecting the Raw data import method from the dropdown menu.
3. After importing, you can sort the files alphabetically if you wish. To do this, shift click the multiple imported raw files to select them all, then right-click (or control-click on MacOS), and select Sort Alphabetically from the popup menu.
4. You can then proceed to process the data in MZmine2 as needed.

Please note that this information is incomplete and may not cover all the necessary steps or potential issues you may encounter when uncompressing mzML.gz files.

--------------

```

Step 5

Action executed:
Web search for 'how to convert mzML to mzXML or mzData'
Function Call:
```javascript
web_search({
  "queries": [
    "how to convert mzML to mzXML or mzData"
  ]
})
```
Result
```
Found the following result for the web search: 'how to convert mzML to mzXML or mzData'
--------------
1. Use MSConvert (ProteoWizard) to convert mzML to mzXML or mzData. Choose an Output Directory, under Options, select mzML or mzXML for output format, 32-bit for binary encoding precision and uncheck Use zlib compression. Under filters, choose Peak Picking with Vendor checked, to centroid the data. Indicate MS-Levels 1-2. Click Add to add the filter. Make sure the PeakPicking filter is the first filter in the list, otherwise, the conversion will not be centroided.

2. For Bruker recalibrated data, it is recommended to perform export analysis to mzXML using the Bruker's Data Analysis software prior to importing to MZmine.

3. MSConvert supports the conversion of AB SCIEX, Agilent, Bruker, Shimadzu, Thermo Scientific, and Waters raw data.

4. MZmine supports both open (e.g., .mzML, .mzXML, .imzML, .netCDF, and .aird) and proprietary formats from Thermo Scientific .raw, and Bruker Daltonics .d and .tdf/tsf. All other raw data files from other vendors must be converted into an open format prior to the import.

5. MyriMatch accepts MS/MS data in the mzData format. However, it doesn't support mzData anymore because ProteoWizard doesn't which is what MM uses for I/O. It now supports all the formats that pwiz does, including the vendor formats on Windows. The recommended format is mzML.

6. You can use msconvert to convert some mzML files to an mzXML file. The 'xml' files from PRIDE come generally in the mzData format.

Note: The information is incomplete as it does not provide detailed step-by-step instructions for each conversion process.

--------------

```
Step 6

Action executed:
Web search for 'LC-MS data preprocessing steps'
Function Call:
```javascript
web_search({
  "queries": [
    "LC-MS data preprocessing steps"
  ]
})
```
Result
```
Found the following result for the web search: 'LC-MS data preprocessing steps'
--------------
1. Noise Filtering: This step involves the removal of unwanted signals or disturbances from the LC-MS data.

2. Deisotoping: This process involves the removal of isotopic peaks from the mass spectra, leaving only the monoisotopic peaks.

3. Peak Detection: This step involves identifying and quantifying the peaks in the LC-MS data.

4. RT Alignment: This step involves aligning the retention times (RTs) of the detected peaks across different LC-MS runs.

5. Peak Matching: This step involves matching the detected peaks across different LC-MS runs based on their m/z values and RTs.

6. Normalization: This step involves adjusting the intensities of the detected peaks to minimize the effects of systematic variations in the LC-MS data.

7. MS/MS Identification: This step involves associating the detected peaks with peptides/proteins through tandem mass spectrometry (MS/MS) identification.

8. Chromatographic Peak Detection: This step involves identifying the chromatographic peaks in the LC-MS data.

9. Sample Alignment: This step involves aligning the samples in the LC-MS data.

10. Peak Correspondence: This step involves establishing correspondence between the detected peaks across different LC-MS runs.

Note: The information is incomplete as it does not provide specific details about how each of these steps is performed.

--------------

```
Step 7

Action executed:
Web search for 'feature detection in LC-MS data'
Function Call:
```javascript
web_search({
  "queries": [
    "feature detection in LC-MS data"
  ]
})
```
Result
```
Found the following result for the web search: 'feature detection in LC-MS data'
--------------
1. Feature detection is a crucial step in the LC/MS data processing pipeline. It should be reliable, reporting as many "real" features as possible while keeping the false positive rate low. The challenge for the algorithms is to detect features of low intensity induced by compounds with low abundance and to avoid feature-like signals caused by chemical noise.

2. Liquid chromatography coupled to mass spectrometry (LC/MS) is an important analytical technology for metabolomics experiments. Determining the boundaries, centres and intensities of the two-dimensional signals in the LC/MS raw data is called feature detection. For the subsequent analysis of complex samples such as plant extracts, a reliable feature detection is mandatory.

3. A new feature detection algorithm _centWave_ for high-resolution LC/MS data sets has been developed. It collects regions of interest (partial mass traces) in the raw-data, and applies continuous wavelet transformation and optionally Gauss-fitting in the chromatographic domain.

4. A novel feature detection method based on deep learning named S is introduced. This method is crucial for high-resolution liquid chromatography-mass spectrometry (LC-MS) data analysis.

5. Feature detection is a critical step in the preprocessing of Liquid Chromatography – Mass Spectrometry (LC-MS) metabolomics data. The predominant approach is to detect features using noise filters and peak shape models based on the data at hand alone.

6. A hybrid feature detection approach is described. Once the database is available, the method employs a four-step procedure to detect features from a new batch of LC-MS profiles.

7. The method analyzes the data using a traditional unsupervised approach with relatively stringent criteria for peak calling. A feature list is generated. The newly found features are compared with those in the database. If a feature found in the new data matches a known feature in the database, it is added to the final feature list.

(Note: The information is incomplete as it does not provide specific details about the performance, precision, or recall of the feature detection methods mentioned.)

--------------

```
Step 8

Action executed:
Web search for 'how to match LC-MS data against metabolite database'
Function Call:
```javascript
web_search({
  "queries": [
    "how to match LC-MS data against metabolite database"
  ]
})
```
Result
```
Found the following result for the web search: 'how to match LC-MS data against metabolite database'
--------------
1. Use an appropriate scoring function to measure the similarity between two MS/MS spectra. Spectra with high similarity are considered to represent the same metabolites.

2. Use computational tools to reduce the number of putative identifications and prioritize them for further verification.

3. Use the measured peak mass to search a database to produce a list of mass-matches. Other parameters such as retention time (RT), MS/MS

spectrum, and/or ion mobility collision cross-section (CCI) can be used to narrow down the list to one unique ID or a few possible structures.

4. Match tolerances of MS1 m/z and RT matches should be set as 15 ppm and 30%, respectively. MS2 spectra of qualified peaks should be further matched against the surrogated MS2 spectra from seed metabolites. The qualified peaks with dot product score larger than 0.5 or matched fragments more than 4 should be linked to the seed metabolites, and their putative structures should be assigned from the reaction-paired neighbor metabolites.

5. Use freely available search algorithms such as the NIST MS Search GUI (graphical user interface), NIST MS PepSearch or MS-DIAL for peak detection and mass spectral deconvolution.

6. Use software tools like XCMS, MS-DIAL, and MZmine for processing, visualizing, and analyzing mass spectrometry-based molecular profile data.

7. For seed metabolites annotation, set the match tolerances as MS1 match, 15 ppm; RT match, 20 s; MS2 match, 0.8 (dot product score). The adducts of protonation and deprotonation should be used in seed annotation in positive and negative modes, respectively.

Note: The information is incomplete as it does not provide a step-by-step guide on how to match LC-MS data against a metabolite database.

--------------

```
Step 9

Action executed:
Web search for 'how to calculate metabolite concentration from LC-MS data'
Function Call:
```javascript
web_search({
  "queries": [
    "how to calculate metabolite concentration from LC-MS data"
  ]
})
```
Result
```
Found the following result for the web search: 'how to calculate metabolite concentration from LC-MS data'
--------------
To calculate metabolite concentration from LC-MS data, the following steps can be followed:

1. Generate a standard curve with the metabolite versus the internal standard. This is considered the best practice. However, in cases where metabolite synthesis is difficult, standard curves are sometimes prepared

using the substrate, assuming that the signal for substrate and the metabolite will be equivalent.

2. The ratio of the slopes of the standard curves can vary up to 4-fold. If the ratio is a fraction, it is inverted and subtracted from 1. For example, if the slope ratio is 0.3, inverting this number gives 3.3, which has an absolute error from perfect agreement of 2.3.

3. The unsigned average difference of the substrate/metabolite ratios from 1 indicates the error. For normal LC-MS, the error is 1.2, whereas for captive spray LC-MS it is 0.8.

4. Using the substrate to construct the standard curve can yield a predicted concentration of the metabolite up to 4-fold lower than the actual concentration to approximately 2-fold higher than the actual metabolite concentration.

5. The goal of data processing is to report a quantitative value per metabolite feature per sample, which is a proxy for its biological concentration.

Please note that the signal varies depending on the compound and the method used. Also, the use of computational and informatics tools can accelerate the identification process and reduce the cost.

The information is incomplete as it does not provide a detailed step-by-step process or formula for calculating the metabolite concentration from LC-MS data.

--------------

```
Step 10

Action executed:
Web search for 'statistical analysis of metabolite concentrations'
Function Call:
```javascript
web_search({
  "queries": [
    "statistical analysis of metabolite concentrations"
  ]
})
```
Result
```
Found the following result for the web search: 'statistical analysis of metabolite concentrations'
--------------
1. The German KORA F4 cohort study involved 1764 blood serum samples and 218 measured metabolites covering various pathways. The Independent Component Analysis (ICA) method was used for the metabolomics data and it outperformed k-means clustering and standard

PCA in terms of a biologically more sound result. However, the specific statistical results or metabolite concentrations are not provided.

2. In a study involving red blood cells, the concentration profile of 84 out of 91 (92%) measured metabolites was accurately predicted using only measurements of five biomarkers. The median of prediction errors (symmetric mean absolute percent error) across all metabolites was 13%. However, the specific metabolite concentrations or the names of the five biomarkers are not provided.

3. In metabolomic analysis, data normalization is required to remove undesired systematic biases. The two most commonly used methods for this are the use of endogenous stable metabolites (like creatinine in urine) and the use of the total spectral area (i.e., area under the curve (AUC)). However, the specific statistical results or metabolite concentrations are not provided.

4. In studies involving LC-MS or GC-MS methods, semi-quantitative results are usually yielded, i.e., the content of metabolites in the experimental groups relative to the control groups. However, the specific statistical results or metabolite concentrations are not provided.

5. Single-sample profiling (SSP) analysis uses an input list of normalized metabolite concentrations in a common biofluid, tissue, or cell type and a database with the normal concentration ranges of these metabolites in the sample. However, the specific statistical results or metabolite concentrations are not provided.

--------------

```

Step 11

Action executed:
Web search for 'how to visualize metabolite concentrations'
Function Call:

```javascript
web_search({
  "queries": [
    "how to visualize metabolite concentrations"
  ]
})
```

Result

```
Found the following result for the web search: 'how to visualize metabolite concentrations'
--------------
1. The method GEM-Vis (Genome-Scale Metabolic model Visualization) is used for the visualization and contextualization of longitudinal metabolomic data in metabolic networks. It offers three different graphical representations of metabolic concentration that allow for different
```
```

	interpretations of metabolomic data through a smooth animation. This method is implemented in the freely available software SBMLsimulator.<br><br>2. Metabolite AutoPlotter, a web-based application, is used for the analysis of metabolite concentrations. It can process metabolites up to M+30. During data processing, the isotopic information is extracted from the metabolite names and all isotopologues are grouped and shown as stacked bars for each condition. These plots can be shown either in absolute intensities (peak areas) or in relative intensities.<br><br>3. Viime offers an interactive volcano plot option to visualize the magnitude of the change in a metabolite along with the statistical significance of that change. The horizontal axis displays the Log2 Fold change while the vertical axis displays the –log10 of the p-value.<br><br>4. Viime also includes a fully interactive heatmap with row and column dendrograms. Selected metabolites are highlighted in orange on the left. Sample groups are colored along the bottom to provide additional context.<br><br>5. Viime has a metabolite correlation network diagram for a cleaner visualization of selected metabolite groups. Hovering over nodes or edges brings up the metabolite identification information and the strength of the correlations respectively.<br><br>6. A Google-map style network visualization system is used to present the results of a given metabolomic study. It supports intuitive and interactive data exploration through point-and-click, dragging and lossless zooming. Additional features include a comprehensive compound library for metabolite name conversion, automatic generation of analysis report, as well as the implementation of various univariate statistical procedures that can be accessed when users click on any metabolite.<br><br>The information is incomplete as it does not provide specific steps or procedures on how to visualize metabolite concentrations using the mentioned methods and tools.<br><br>--------------

8. **Results of ChatGPT-3.5**

WGS data analysis   Case 1.1: Genome assembly

<table>
<tr><td>User's Inputs</td><td>You are a bioinformatician who specializes in writing code and performing analysis for a variety of bioinformatic data analysis tasks. Now I provide you with an Ubuntu 18.04 system where I save the following data, each line including the data path and data description.

SRR1234567_R1.fastq.gz: Paired-end Illumina WGS reads, forward
SRR1234567_R2.fastq.gz: Paired-end Illumina WGS reads, reverse
TruSeq3-PE.fa: Adapter sequences for trimming
Reference.fasta: Reference genome for the organism under study in FASTA format

I need you to help me plan what needs to be done in each step of the analysis process, and help me write the code for each step to install softwares, use tools and accomplish the following main goal:

To perform genome assembly using paired-end WGS data</td></tr>
<tr><td>Generated Results by ChatGPT-3.5</td><td>Agent message

First, you would need to set up the system and install necessary software for the analysis process. Here's a plan broken down into steps along with the code for each step:

Step 1: Install necessary software
```bash
# Update system
sudo apt update && sudo apt upgrade

# Install required tools
sudo apt install trimmomatic
sudo apt install spades
```

Step 2: Quality control and adapter trimming
```bash
trimmomatic PE SRR1234567_R1.fastq.gz SRR1234567_R2.fastq.gz \
    SRR1234567_R1_trimmed.fastq.gz unpaired_R1.fastq.gz \
    SRR1234567_R2_trimmed.fastq.gz unpaired_R2.fastq.gz \
    ILLUMINACLIP:TruSeq3-PE.fa:2:30:10 LEADING:3 TRAILING:3 SLIDINGWINDOW:4:15 MINLEN:36
```

Step 3: Genome assembly
```bash
spades.py -1 SRR1234567_R1_trimmed.fastq.gz -2 SRR1234567_R2_trimmed.fastq.gz -o genome_assembly -t 4
```</td></tr>
</table>

	In the above steps, trimmomatic is used for quality control and adapter trimming, and SPAdes is used for genome assembly. The example provided assumes that the necessary tools and data are located within the same directory or in the system's default search paths. Adjust the commands according to the actual location of the data and software on your system. Additionally, this is a simplified example assuming default settings for the software. There may be additional parameters that you might want to consider based on the specific requirements of your data and analysis.

WGS/WES data analysis **Case 2.1 and Case 2.2 : Somatic SNV+indel calling and annotation**

**User's Inputs**	You are a bioinformatician who specializes in writing code and performing analysis for a variety of bioinformatic data analysis tasks. Now I provide you with an Ubuntu 18.04 system where I save the following data, each line including the data path and data description.<br><br>SRR23015874_R1.fastq.gz: paired end read1 from human whole genome sequencing, normal samples replicate 1<br>SRR23015874_R2.fastq.gz: paired end read2 from human whole genome sequencing, normal samples replicate 1<br>SRR23015876_R1.fastq.gz: paired end read1 from human whole genome sequencing, tumor samples replicate 1<br>SRR23015876_R2.fastq.gz: paired end read2 from human whole genome sequencing, tumor samples replicate 1<br>SRR23015877_R1.fastq.gz: paired end read1 from human whole genome sequencing, tumor samples replicate 2<br>SRR23015877_R2.fastq.gz: paired end read2 from human whole genome sequencing, tumor samples replicate 2<br>TruSeq3-PE.fa: trimming adapter<br>hg38.fa: human hg38 reference genome fasta<br><br>I need you to help me plan what needs to be done in each step of the analysis process, and help me write the code for each step to install softwares, use tools and accomplish the following main goal:<br><br>call somatic mutations in tumor samples over the matched normal samples and annotate the identified mutations
**Generated Results by ChatGPT-3.5**	For this analysis, you'd need to prepare the system, preprocess the data, align the sequences, call somatic mutations, and annotate the identified mutations. Here's a plan broken down into steps along with the code for each step:<br><br>Step 1: Install necessary software<br>```bash<br># Update system<br>sudo apt update && sudo apt upgrade<br><br># Install required tools<br>sudo apt install bwa<br>sudo apt install samtools<br>sudo apt install gatk4 # Genome Analysis Toolkit<br>sudo apt install bcftools<br>```<br><br>Step 2: Data preprocessing - Adapter trimming and quality control<br>```bash<br># Assuming Trimmomatic is installed<br>java -jar trimmomatic PE SRR23015874_R1.fastq.gz SRR23015874_R2.fastq.gz \\<br>SRR23015874_R1_trimmed.fastq.gz unpaired_R1.fastq.gz \\<br>SRR23015874_R2_trimmed.fastq.gz unpaired_R2.fastq.gz \\<br>ILLUMINACLIP:TruSeq3-PE.fa:2:30:10 LEADING:3 TRAILING:3 SLIDINGWINDOW:4:15 MINLEN:36

```
# Repeat for the other samples
```

Step 3: Align the sequences to the reference genome
```bash
# Align the sequences
bwa mem hg38.fa SRR23015874_R1_trimmed.fastq.gz
SRR23015874_R2_trimmed.fastq.gz > normal_rep1.sam
bwa mem hg38.fa SRR23015876_R1_trimmed.fastq.gz
SRR23015876_R2_trimmed.fastq.gz > tumor_rep1.sam
bwa mem hg38.fa SRR23015877_R1_trimmed.fastq.gz
SRR23015877_R2_trimmed.fastq.gz > tumor_rep2.sam
```

Step 4: Convert and sort SAM to BAM, and index
```bash
samtools view -S -b normal_rep1.sam > normal_rep1.bam
samtools view -S -b tumor_rep1.sam > tumor_rep1.bam
samtools view -S -b tumor_rep2.sam > tumor_rep2.bam

samtools sort normal_rep1.bam -o normal_rep1_sorted.bam
samtools index normal_rep1_sorted.bam

samtools sort tumor_rep1.bam -o tumor_rep1_sorted.bam
samtools index tumor_rep1_sorted.bam

samtools sort tumor_rep2.bam -o tumor_rep2_sorted.bam
samtools index tumor_rep2_sorted.bam
```

Step 5: Call somatic mutations using GATK4
```bash
gatk Mutect2 -R hg38.fa -I normal_rep1_sorted.bam -I
tumor_rep1_sorted.bam -tumor tumor_rep1 -normal normal_rep1 -O
tumor_vs_normal_1.vcf.gz
gatk Mutect2 -R hg38.fa -I normal_rep1_sorted.bam -I
tumor_rep2_sorted.bam -tumor tumor_rep2 -normal normal_rep1 -O
tumor_vs_normal_2.vcf.gz
```

Step 6: Merge and annotate mutations
```bash
bcftools merge tumor_vs_normal_1.vcf.gz tumor_vs_normal_2.vcf.gz -O z -
o merged_tumor_vs_normal.vcf.gz

# Annotate the VCF file using a tool like VEP (Variant Effect Predictor) or
ANNOVAR
# Example for VEP:
vep -i merged_tumor_vs_normal.vcf.gz -o annotated_variants.txt
```

	These steps should set you up for the analysis of calling somatic mutations in tumor samples over matched normal samples and annotating the identified mutations using the provided data. Adjust the commands according to the actual location of the data and software on your system. Additionally, some tools may require additional setup and configuration steps.

WGS/WES data analysis **Case 2.3: Structure variation identification with normal**

**User's Inputs**	You are a bioinformatician who specializes in writing code and performing analysis for a variety of bioinformatic data analysis tasks. Now I provide you with an Ubuntu 18.04 system where I save the following data, each line including the data path and data description.<br><br>SRR23015874_R1.fastq.gz: paired end read1 from human whole genome sequencing, the matched normal samples replicate 1<br>SRR23015874_R2.fastq.gz: paired end read2 from human whole genome sequencing, the matched normal samples replicate 1<br>SRR23015876_R1.fastq.gz: paired end read1 from human whole genome sequencing, tumor samples replicate 1<br>SRR23015876_R2.fastq.gz: paired end read2 from human whole genome sequencing, tumor samples replicate 1<br>SRR23015877_R1.fastq.gz: paired end read1 from human whole genome sequencing, tumor samples replicate 2<br>SRR23015877_R2.fastq.gz: paired end read2 from human whole genome sequencing, tumor samples replicate 2<br>TruSeq3-PE.fa: trimming adapter<br>hg38.fa: human hg38 reference genome fasta<br><br>I need you to help me plan what needs to be done in each step of the analysis process, and help me write the code for each step to install softwares, use tools and accomplish the following main goal:<br><br>To identify structural variations, which can include large insertions, deletions, inversions, duplications, and translocations, by comparing the sequencing data from the tumor sample over the matched normal sample
**Generated Results by ChatGPT-3.5**	Certainly! Here's the breakdown of steps and the corresponding code to achieve your goal:<br><br>Step 1: Install necessary software<br>```bash<br># Update the system<br>sudo apt update && sudo apt upgrade<br><br># Install required tools<br>sudo apt install bwa<br>sudo apt install samtools<br>sudo apt install minimap2<br>sudo apt install lumpy-sv<br>```<br><br>Step 2: Data preprocessing - Adapter trimming and quality control<br>```bash<br># Assuming Trimmomatic is installed<br>java -jar trimmomatic PE SRR23015874_R1.fastq.gz SRR23015874_R2.fastq.gz \\<br>SRR23015874_R1_trimmed.fastq.gz unpaired_R1.fastq.gz \\<br>SRR23015874_R2_trimmed.fastq.gz unpaired_R2.fastq.gz \\<br>ILLUMINACLIP:TruSeq3-PE.fa:2:30:10 LEADING:3 TRAILING:3 SLIDINGWINDOW:4:15 MINLEN:36

Repeat for the tumor samples
```

Step 3: Align the sequences to the reference genome
```bash
# Align the sequences
bwa mem hg38.fa SRR23015874_R1_trimmed.fastq.gz
SRR23015874_R2_trimmed.fastq.gz > normal_rep1.sam
bwa mem hg38.fa SRR23015876_R1.fastq.gz
SRR23015876_R2.fastq.gz > tumor_rep1.sam
bwa mem hg38.fa SRR23015877_R1.fastq.gz
SRR23015877_R2.fastq.gz > tumor_rep2.sam
```

Step 4: Convert and sort SAM to BAM, and index
```bash
samtools view -S -b normal_rep1.sam > normal_rep1.bam
samtools view -S -b tumor_rep1.sam > tumor_rep1.bam
samtools view -S -b tumor_rep2.sam > tumor_rep2.bam

samtools sort normal_rep1.bam -o normal_rep1_sorted.bam
samtools index normal_rep1_sorted.bam

samtools sort tumor_rep1.bam -o tumor_rep1_sorted.bam
samtools index tumor_rep1_sorted.bam

samtools sort tumor_rep2.bam -o tumor_rep2_sorted.bam
samtools index tumor_rep2_sorted.bam
```

Step 5: Detect structural variations using Lumpy
```bash
lumpyexpress -B
normal_rep1_sorted.bam,tumor_rep1_sorted.bam,tumor_rep2_sorted.bam
-S somatic_sv.vcf
```

Step 6: Annotate the identified structural variations
```bash
# Annotate the structural variations using a tool like ANNOVAR or VEP
# Example for ANNOVAR:
./annotate_variation.pl -downdb -webfrom annovar -build hg38
somatic_sv.vcf humandb/
```

These steps should help you identify structural variations, such as large insertions, deletions, inversions, duplications, and translocations, by comparing the sequencing data from the tumor sample over the matched normal sample. Adjust the commands according to the actual location of the data and software on your system. Additionally, some tools may require additional setup and configuration steps.
```

WGS/WES data analysis **Case 2.4: Structure variation identification without normal**

<table>
<tr><td>User's Inputs</td><td>You are a bioinformatician who specializes in writing code and performing analysis for a variety of bioinformatic data analysis tasks. Now I provide you with an Ubuntu 18.04 system where I save the following data, each line including the data path and data description.

SRR23015876_R1.fastq.gz: paired end read1 from human whole genome sequencing, tumor samples replicate 1
SRR23015876_R2.fastq.gz: paired end read2 from human whole genome sequencing, tumor samples replicate 1
SRR23015877_R1.fastq.gz: paired end read1 from human whole genome sequencing, tumor samples replicate 2
SRR23015877_R2.fastq.gz: paired end read2 from human whole genome sequencing, tumor samples replicate 2
TruSeq3-PE.fa: trimming adapter
hg38.fa: human hg38 reference genome fasta

I need you to help me plan what needs to be done in each step of the analysis process, and help me write the code for each step to install softwares, use tools and accomplish the following main goal:

To identify structural variations, which can include large insertions, deletions, inversions, duplications, and translocations based on the sequencing data from the tumor sample.</td></tr>
<tr><td>Generated Results by ChatGPT-3.5</td><td>To identify structural variations using the provided data, you'd need to install necessary software, preprocess the data, align the sequences, and finally detect structural variations. Here's a plan broken down into steps along with the code for each step:

Step 1: Install necessary software
```bash
# Update system
sudo apt update && sudo apt upgrade

# Install required tools
sudo apt install bwa
sudo apt install samtools
sudo apt install bcftools
sudo apt install minimap2
sudo apt install lumpy-sv
```

Step 2: Data preprocessing - Adapter trimming and quality control
```bash
Assuming Trimmomatic is installed
java -jar trimmomatic PE SRR23015876_R1.fastq.gz SRR23015876_R2.fastq.gz \
    SRR23015876_R1_trimmed.fastq.gz unpaired_R1.fastq.gz \
    SRR23015876_R2_trimmed.fastq.gz unpaired_R2.fastq.gz \
    ILLUMINACLIP:TruSeq3-PE.fa:2:30:10 LEADING:3 TRAILING:3 SLIDINGWINDOW:4:15 MINLEN:36</td></tr>
</table>

```
java -jar trimmomatic PE SRR23015877_R1.fastq.gz
SRR23015877_R2.fastq.gz \
    SRR23015877_R1_trimmed.fastq.gz unpaired_R1.fastq.gz \
    SRR23015877_R2_trimmed.fastq.gz unpaired_R2.fastq.gz \
    ILLUMINACLIP:TruSeq3-PE.fa:2:30:10 LEADING:3 TRAILING:3
SLIDINGWINDOW:4:15 MINLEN:36
```

Step 3: Align the sequences to the reference genome
```bash
# Align the sequences using BWA or Minimap2
bwa mem hg38.fa SRR23015876_R1_trimmed.fastq.gz
SRR23015876_R2_trimmed.fastq.gz > tumor_rep1.sam
bwa mem hg38.fa SRR23015877_R1_trimmed.fastq.gz
SRR23015877_R2_trimmed.fastq.gz > tumor_rep2.sam
```

Step 4: Convert and sort SAM to BAM, and index
```bash
samtools view -S -b tumor_rep1.sam > tumor_rep1.bam
samtools view -S -b tumor_rep2.sam > tumor_rep2.bam

samtools sort tumor_rep1.bam -o tumor_rep1_sorted.bam
samtools index tumor_rep1_sorted.bam

samtools sort tumor_rep2.bam -o tumor_rep2_sorted.bam
samtools index tumor_rep2_sorted.bam
```

Step 5: Detect structural variations using Lumpy
```bash
lumpyexpress -B tumor_rep1_sorted.bam,tumor_rep2_sorted.bam -S
tumor_sv.vcf
```

These steps should help you identify structural variations, such as large insertions, deletions, inversions, duplications, and translocations, based on the sequencing data from the tumor sample. Always adjust the commands according to the actual location of the data and software on your system. Additionally, some tools may require additional setup and configuration steps.

ChIP-Seq Analysis   Case 3.1: Call Peaks

<table>
<tr><td>User's Inputs</td><td>You are a bioinformatician who specializes in writing code and performing analysis for a variety of bioinformatic data analysis tasks. Now I provide you with an Ubuntu 18.04 system where I save the following data, each line including the data path and data description.

SRR620204.fastq.gz: chip-seq data for Ring1B
SRR620205.fastq.gz: chip-seq data for cbx7
SRR620206.fastq.gz: chip-seq data for SUZ12
SRR620208.fastq.gz: chip-seq data for IgGold
mm39.ncbiRefSeq.gtf: genome annotations mouse
mm39.fa: mouse genome

I need you to help me plan what needs to be done in each step of the analysis process, and help me write the code for each step to install softwares, use tools and accomplish the following main goal:

Call peaks for protein cbx7 with IgGold as control</td></tr>
<tr><td>Generated Results by ChatGPT-3.5</td><td>To call peaks for the protein cbx7 using IgGold as the control, the following steps need to be performed:

Step 1: Install necessary software
```bash
# Update system
sudo apt update && sudo apt upgrade

# Install required tools such as Bowtie, Bowtie2, SAMtools, MACS2
sudo apt install bowtie
sudo apt install bowtie2
sudo apt install samtools
pip install MACS2
```

Step 2: Preprocess data – Align the sequences
```bash
Assuming mm39.fa is the mouse genome file
bowtie2 -x mm39 -U SRR620205.fastq.gz -S cbx7_alignment.sam
bowtie2 -x mm39 -U SRR620208.fastq.gz -S IgGold_alignment.sam
```

Step 3: Convert and sort SAM to BAM
```bash
samtools view -S -b cbx7_alignment.sam > cbx7_alignment.bam
samtools sort cbx7_alignment.bam -o cbx7_sorted.bam
samtools index cbx7_sorted.bam

samtools view -S -b IgGold_alignment.sam > IgGold_alignment.bam
samtools sort IgGold_alignment.bam -o IgGold_sorted.bam
samtools index IgGold_sorted.bam</td></tr>
</table>

	```<br><br>Step 4: Call peaks using MACS2<br>```bash<br>macs2 callpeak -t cbx7_sorted.bam -c IgGold_sorted.bam -f BAM -g mm --outdir cbx7_peaks<br>```<br><br>These steps should help you call peaks for the protein cbx7 using IgGold as the control. Always adjust the commands according to the actual location of the data and software on your system. Additionally, some tools may require additional setup and configuration steps.

ChIP-Seq Analysis Case 3.2: Motif Discovery

<table>
<tr><td>User's Inputs</td><td>You are a bioinformatician who specializes in writing code and performing analysis for a variety of bioinformatic data analysis tasks. Now I provide you with an Ubuntu 18.04 system where I save the following data, each line including the data path and data description.

SRR620204.fastq.gz: chip-seq data for Ring1B
SRR620205.fastq.gz: chip-seq data for cbx7
SRR620206.fastq.gz: chip-seq data for SUZ12
SRR620208.fastq.gz: chip-seq data for IgGold
mm39.ncbiRefSeq.gtf: genome annotations mouse
mm39.fa: mouse genome

I need you to help me plan what needs to be done in each step of the analysis process, and help me write the code for each step to install softwares, use tools and accomplish the following main goal:

Discover motifs within the peaks of protein SUZ12 with IgGold as control</td></tr>
<tr><td>Generated Results by ChatGPT-3.5</td><td>To discover motifs within the peaks of protein SUZ12 with IgGold as the control, the following steps can be performed:

Step 1: Install necessary software
```bash
# Update system
sudo apt update && sudo apt upgrade

# Install required tools such as Bowtie, Bowtie2, SAMtools, MACS2, MEME Suite
sudo apt install bowtie
sudo apt install bowtie2
sudo apt install samtools
pip install MACS2
sudo apt install meme
```

Step 2: Preprocess data – Align the sequences
```bash
Assuming mm39.fa is the mouse genome file
bowtie2 -x mm39 -U SRR620206.fastq.gz -S SUZ12_alignment.sam
bowtie2 -x mm39 -U SRR620208.fastq.gz -S IgGold_alignment.sam
```

Step 3: Convert and sort SAM to BAM
```bash
samtools view -S -b SUZ12_alignment.sam > SUZ12_alignment.bam
samtools sort SUZ12_alignment.bam -o SUZ12_sorted.bam
samtools index SUZ12_sorted.bam

samtools view -S -b IgGold_alignment.sam > IgGold_alignment.bam
samtools sort IgGold_alignment.bam -o IgGold_sorted.bam
samtools index IgGold_sorted.bam
```</td></tr>
</table>

	Step 4: Call peaks using MACS2<br>```bash<br>macs2 callpeak -t SUZ12_sorted.bam -c IgGold_sorted.bam -f BAM -g mm --outdir SUZ12_peaks<br>```<br><br>Step 5: Discover motifs within the peaks using MEME Suite<br>```bash<br>meme-chip -db mm39.ncbiRefSeq.gtf -meme-mod zoops -meme-nmotifs 3 -meme-minw 6 -meme-maxw 12 -meme SUZ12_peaks/NA_peaks.narrowPeak<br>```<br><br>These steps should help you discover motifs within the peaks of protein SUZ12 with IgGold as the control. Always adjust the commands according to the actual location of the data and software on your system. Additionally, some tools may require additional setup and configuration steps.

ChIP-Seq Analysis   Case 3.3: Functional Enrichment

**User's Inputs**	You are a bioinformatician who specializes in writing code and performing analysis for a variety of bioinformatic data analysis tasks. Now I provide you with an Ubuntu 18.04 system where I save the following data, each line including the data path and data description.<br><br>SRR620204.fastq.gz: chip-seq data for Ring1B<br>SRR620205.fastq.gz: chip-seq data for cbx7<br>SRR620206.fastq.gz: chip-seq data for SUZ12<br>SRR620208.fastq.gz: chip-seq data for IgGold<br>mm39.ncbiRefSeq.gtf: genome annotations mouse<br>mm39.fa: mouse genome<br><br>I need you to help me plan what needs to be done in each step of the analysis process, and help me write the code for each step to install softwares, use tools and accomplish the following main goal:<br><br>Perform functional enrichment for protein Ring1B, use protein IgGold as the control
**Generated Results by ChatGPT-3.5**	Performing functional enrichment involves comparing the identified gene sets (e.g., from ChIP-seq peaks) to functional annotations, such as gene ontology terms or pathways, to identify overrepresented functions associated with those genes.<br><br>Here's a general outline of the steps and codes for performing functional enrichment using ChIP-seq data for protein Ring1B with IgGold as the control:<br><br>Step 1: Install necessary software<br>```bash<br># Update system<br>sudo apt update && sudo apt upgrade<br><br># Install required tools such as Bowtie, Bowtie2, SAMtools, MACS2, BEDTools, HOMER<br>sudo apt install bowtie<br>sudo apt install bowtie2<br>sudo apt install samtools<br>pip install MACS2<br>sudo apt install bedtools<br># Install HOMER (http://homer.ucsd.edu/homer/download.html)<br>```<br><br>Step 2: Preprocess data – Align the sequences<br>```bash<br># Assuming mm39.fa is the mouse genome file<br>bowtie2 -x mm39 -U SRR620204.fastq.gz -S Ring1B_alignment.sam<br>bowtie2 -x mm39 -U SRR620208.fastq.gz -S IgGold_alignment.sam<br>```<br><br>Step 3: Convert and sort SAM to BAM

```bash
samtools view -S -b Ring1B_alignment.sam > Ring1B_alignment.bam
samtools sort Ring1B_alignment.bam -o Ring1B_sorted.bam
samtools index Ring1B_sorted.bam

samtools view -S -b IgGold_alignment.sam > IgGold_alignment.bam
samtools sort IgGold_alignment.bam -o IgGold_sorted.bam
samtools index IgGold_sorted.bam
```

Step 4: Call peaks using MACS2

```bash
macs2 callpeak -t Ring1B_sorted.bam -c IgGold_sorted.bam -f BAM -g mm
--outdir Ring1B_peaks
```

Step 5: Perform functional enrichment using HOMER

```bash
# Use HOMER to perform motif and/or functional enrichment analysis
findMotifsGenome.pl Ring1B_peaks/NA_peaks.narrowPeak mm39 -size
given
```

Always adjust the commands according to the actual location of the data and software on your system. Additionally, some tools may require additional setup and configuration steps.

Bisulfite-Seq data analysis Case 4.1: Identifying DNA methylation

**User's Inputs**	You are a bioinformatician who specializes in writing code and performing analysis for a variety of bioinformatic data analysis tasks. Now I provide you with an Ubuntu 18.04 system where I save the following data, each line including the data path and data description.<br><br>Sample_R1.fastq.gz: Raw sequencing reads from the bisulfite-converted DNA, typically paired-end reads. These reads contain information about the converted DNA, where unmethylated cytosines are converted to uracils (T) during library preparation.<br>Sample_R2.fastq.gz: Paired-end reads that complement the R1 reads. These are typically used for quality control and paired-end analysis.<br>TruSeq3-PE.fa: Adapter sequences for trimming<br>Reference.fasta: Reference genome for the organism under study in FASTA format, include bisulfite conversion.<br><br>I need you to help me plan what needs to be done in each step of the analysis process, and help me write the code for each step to install softwares, use tools and accomplish the following main goal:<br><br>To identify DNA methylation patterns in the genome based on the Bisulfite-Seq data.
**Generated Results by ChatGPT-3.5**	To identify DNA methylation patterns in the genome based on the Bisulfite-Seq data, the following steps can be performed:<br><br>Step 1: Install necessary software<br>```bash<br># Update system<br>sudo apt update && sudo apt upgrade<br><br># Install required tools such as Bismark, Bowtie2, Samtools, and others<br># Bismark is commonly used for Bisulfite-Seq analysis<br># Samtools is used for manipulating SAM and BAM files<br># Other tools or languages might be necessary for downstream analysis, such as R for data visualization and analysis<br>```<br><br>Step 2: Data preprocessing – Adapter trimming, quality control, and alignment<br>```bash<br># Assuming Trimmomatic and Bismark are installed<br>java -jar trimmomatic PE Sample_R1.fastq.gz Sample_R2.fastq.gz \\<br>Sample_R1_trimmed.fastq.gz unpaired_R1.fastq.gz \\<br>Sample_R2_trimmed.fastq.gz unpaired_R2.fastq.gz \\<br>ILLUMINACLIP:TruSeq3-PE.fa:2:30:10 LEADING:3 TRAILING:3 SLIDINGWINDOW:4:15 MINLEN:36<br><br># Align the sequences (example using Bismark)<br>bismark --genome Reference.fasta -1 Sample_R1_trimmed.fastq.gz -2 Sample_R2_trimmed.fastq.gz

	```<br><br>Step 3: Deduplicate and sort the BAM file<br>```bash<br>bismark_methylation_extractor --paired-end --bedGraph --comprehensive aligned_reads.bam<br>sort -k1,1 -k2,2n aligned_reads.cov > aligned_reads_sorted.cov<br>```<br><br>Step 4: Calculate DNA methylation levels<br>```bash<br>bismark2bedGraph --CX aligned_reads.bam<br>coverage2cytosine --genome_folder Reference.fasta --CX aligned_reads.bismark.cov<br>```<br><br>Step 5: Visualize and analyze DNA methylation data<br>```bash<br># Use a genome browser or tools like IGV, Integrative Genomics Viewer, or Bismark tools for visualization and further statistical analysis<br>```<br><br>These steps should help you identify DNA methylation patterns in the genome based on the Bisulfite-Seq data. Always adjust the commands according to the actual location of the data and software on your system. Additionally, some tools may require additional setup and configuration steps.

ATAC-seq data analysis **Case 5.1: Identifying open chromatin regions**

**User's Inputs**	You are a bioinformatician who specializes in writing code and performing analysis for a variety of bioinformatic data analysis tasks. Now I provide you with an Ubuntu 18.04 system where I save the following data, each line including the data path and data description.<br><br>Sample_R1.fastq.gz: Raw sequencing reads from the ATAC-seq experiment. These are typically paired-end reads representing fragments of open chromatin regions.<br>Sample_R2.fastq.gz: Paired-end reads that complement the R1 reads.<br>TruSeq3-PE.fa: Adapter sequences for trimming<br>Reference.fasta: Reference genome for the organism under study in FASTA format. It matches the genome used for the ATAC-seq library preparation.<br><br>I need you to help me plan what needs to be done in each step of the analysis process, and help me write the code for each step to install softwares, use tools and accomplish the following main goal:<br><br>To identify open chromatin regions within the genome based on the ATAC-seq data.
**Generated Results by ChatGPT-3.5**	To identify open chromatin regions within the genome based on the ATAC-seq data, the following steps can be performed:<br><br>Step 1: Install necessary software<br>```bash<br># Update system<br>sudo apt update && sudo apt upgrade<br><br># Install required tools such as Bowtie2, Samtools, Picard, BEDTools, and MACS2<br>sudo apt install bowtie2<br>sudo apt install samtools<br>sudo apt install picard-tools<br>sudo apt install bedtools<br>pip install MACS2<br>```<br><br>Step 2: Data preprocessing – Adapter trimming and alignment<br>```bash<br># Assuming Trimmomatic and Bowtie2 are installed<br>java -jar trimmomatic PE Sample_R1.fastq.gz Sample_R2.fastq.gz \\<br>Sample_R1_trimmed.fastq.gz unpaired_R1.fastq.gz \\<br>Sample_R2_trimmed.fastq.gz unpaired_R2.fastq.gz \\<br>ILLUMINACLIP:TruSeq3-PE.fa:2:30:10 LEADING:3 TRAILING:3 SLIDINGWINDOW:4:15 MINLEN:36<br><br># Align the sequences using Bowtie2 to the reference genome<br>bowtie2 -x Reference.fasta -1 Sample_R1_trimmed.fastq.gz -2 Sample_R2_trimmed.fastq.gz -S aligned_reads.sam<br>```

	Step 3: Convert, sort, and index SAM to BAM<br>```bash<br>samtools view -S -b aligned_reads.sam > aligned_reads.bam<br>samtools sort aligned_reads.bam -o aligned_reads_sorted.bam<br>samtools index aligned_reads_sorted.bam<br>```<br><br>Step 4: Call peaks using MACS2<br>```bash<br>macs2 callpeak -t aligned_reads_sorted.bam -f BAM -g hs --outdir open_chromatin_regions<br>```<br><br>Step 5: Visualize and analyze open chromatin regions<br>```bash<br># Use genomic browsers, like IGV (Integrative Genomics Viewer), or downstream analysis tools for interpretation and downstream analysis<br>```<br><br>These steps should help you identify open chromatin regions within the genome based on the ATAC-seq data. Always adjust the commands according to the actual location of the data and software on your system. Additionally, some tools may require additional setup and configuration steps.

DNase-seq data analysis **Case 6.1: Identifying DnaseI hypersensitive site**

<table>
<tr><td>User's Inputs</td><td>You are a bioinformatician who specializes in writing code and performing analysis for a variety of bioinformatic data analysis tasks. Now I provide you with an Ubuntu 18.04 system where I save the following data, each line including the data path and data description.

Sample_R1.fastq.gz: Raw sequencing reads from the DNase-seq experiment.
Sample_R2.fastq.gz: Paired-end reads that complement the R1 reads, often used for quality control and paired-end analysis.
TruSeq3-PE.fa: Adapter sequences for trimming.
Reference.fasta: Reference genome for the organism under study in FASTA format

I need you to help me plan what needs to be done in each step of the analysis process, and help me write the code for each step to install softwares, use tools and accomplish the following main goal:

To identify DNase I hypersensitive sites (regions of open chromatin) within the genome based on the DNase-seq data.</td></tr>
<tr><td>Generated Results by ChatGPT-3.5</td><td>To identify DNase I hypersensitive sites (DHS) within the genome based on the DNase-seq data, the following steps can be performed:

Step 1: Install necessary software
```bash
# Update system
sudo apt update && sudo apt upgrade

# Install required tools such as Bowtie2, Samtools, BedTools, and MACS2
sudo apt-get install bowtie2
sudo apt-get install samtools
sudo apt-get install bedtools
pip install MACS2
```

Step 2: Data preprocessing – Adapter trimming and alignment
```bash
Assuming Trimmomatic and Bowtie2 are installed
java -jar trimmomatic PE Sample_R1.fastq.gz Sample_R2.fastq.gz \
    Sample_R1_trimmed.fastq.gz unpaired_R1.fastq.gz \
    Sample_R2_trimmed.fastq.gz unpaired_R2.fastq.gz \
    ILLUMINACLIP:TruSeq3-PE.fa:2:30:10 LEADING:3 TRAILING:3 SLIDINGWINDOW:4:15 MINLEN:36

Align the sequences using Bowtie2 to the reference genome
bowtie2 -x Reference.fasta -1 Sample_R1_trimmed.fastq.gz -2 Sample_R2_trimmed.fastq.gz -S aligned_reads.sam
```

Step 3: Convert, sort, and index SAM to BAM</td></tr>
</table>

	```bash<br>samtools view -S -b aligned_reads.sam > aligned_reads.bam<br>samtools sort aligned_reads.bam -o aligned_reads_sorted.bam<br>samtools index aligned_reads_sorted.bam<br>```<br><br>Step 4: Call peaks using MACS2<br>```bash<br>macs2 callpeak -t aligned_reads_sorted.bam -f BAM -g hs --outdir dhs_sites<br>```<br><br>Step 5: Visualize and analyze DNase I hypersensitive sites<br>```bash<br># Use genomic browsers, like IGV (Integrative Genomics Viewer), or downstream analysis tools for interpretation and downstream analysis<br>```<br><br>These steps should help you identify DNase I hypersensitive sites within the genome based on the DNase-seq data. Always adjust the commands according to the actual location of the data and software on your system. Additionally, some tools may require additional setup and configuration steps.

4C-seq data analysis   Case 7.1: Find genomics interactions

**User's Inputs**	You are a bioinformatician who specializes in writing code and performing analysis for a variety of bioinformatic data analysis tasks. Now I provide you with an Ubuntu 18.04 system where I save the following data, each line including the data path and data description.<br><br>sample_4C-seq_data.fastq: Raw sequencing reads or aligned data from the 4C-seq experiment, depending on the starting point.<br>viewpoint_coordinates.bed: A file specifying the genomic coordinates of the viewpoint we want to study.<br>reference_genome.fasta: A reference genome in FASTA format for the organism under study. This should match the genome used for the 4C-seq library preparation.<br><br>I need you to help me plan what needs to be done in each step of the analysis process, and help me write the code for each step to install softwares, use tools and accomplish the following main goal:<br><br>To identify genomic interactions originating from a specific genomic viewpoint using 4C-seq data.
**Generated Results by ChatGPT-3.5**	To identify genomic interactions originating from a specific genomic viewpoint using 4C-seq data, the following steps can be performed:<br><br>Step 1: Install necessary software<br>```bash<br># Update system<br>sudo apt update && sudo apt upgrade<br><br># Install required tools such as Bowtie2, Samtools, BedTools, and HiC-Pro<br>sudo apt-get install bowtie2<br>sudo apt-get install samtools<br>sudo apt-get install bedtools<br># Install HiC-Pro from source or using a package manager<br>```<br><br>Step 2: Process the 4C-seq data and align to the reference genome<br>```bash<br># Assuming you have raw sequencing reads, quality control, adapter trimming, and alignment can be performed using Bowtie2<br>bowtie2 -x reference_genome.fasta -U sample_4C-seq_data.fastq -S aligned_reads.sam<br>samtools view -S -b aligned_reads.sam > aligned_reads.bam<br>samtools sort aligned_reads.bam -o aligned_reads_sorted.bam<br>samtools index aligned_reads_sorted.bam<br>```<br><br>Step 3: Identify interactions using HiC-Pro<br>After installing HiC-Pro, follow the necessary steps to configure and process 4C-seq data, specifying the viewpoint coordinates using the viewpoint_coordinates.bed file, and perform the interaction calling and analysis according to the HiC-Pro documentation. Additionally, it's crucial to configure HiC-Pro to handle 4C-seq data specifically.

	Step 4: Visualize and analyze 4C-seq interactions<br>After obtaining the interactions, visualize and analyze the data using appropriate tools or packages such as Juicebox, HiGlass, or custom scripts for downstream analysis and interpretation.<br><br>These steps should help you identify genomic interactions originating from a specific genomic viewpoint using 4C-seq data. Always adjust the commands according to the actual location of the data and software on your system. Additionally, some tools may require additional setup and configuration steps.

Nanopore DNA sequencing data analysis Case 8.1: Genome assembly

**User's Inputs**	You are a bioinformatician who specializes in writing code and performing analysis for a variety of bioinformatic data analysis tasks. Now I provide you with an Ubuntu 18.04 system where I save the following data, each line including the data path and data description.<br><br>sample_nanopore_reads.fastq: Raw Nanopore sequencing data in fastq format.<br>eference_genome.fasta: A reference genome in FASTA format, if a closely related reference is available and used as a guide during the assembly<br><br>I need you to help me plan what needs to be done in each step of the analysis process, and help me write the code for each step to install softwares, use tools and accomplish the following main goal:<br><br>To assemble a genome from the Nanopore sequencing data.
**Generated Results by ChatGPT-3.5**	To assemble a genome from Nanopore sequencing data, the following steps can be performed:<br><br>Step 1: Install necessary software<br>```bash<br># Update system<br>sudo apt update && sudo apt upgrade<br><br># Install required tools such as Minimap2, Samtools, and Canu<br>sudo apt install minimap2<br>sudo apt install samtools<br># Download and install Canu from the official website:<br>https://canu.readthedocs.io/en/latest/quick-start.html#installation<br>```<br><br>Step 2: Preprocess the Nanopore reads and map to the reference genome (if available)<br>```bash<br># Map to a closely related reference genome (if available) using minimap2<br>minimap2 -ax map-ont reference_genome.fasta<br>sample_nanopore_reads.fastq > aligned_reads.sam<br>samtools view -S -b aligned_reads.sam > aligned_reads.bam<br>samtools sort aligned_reads.bam -o aligned_reads_sorted.bam<br>samtools index aligned_reads_sorted.bam<br>```<br><br>Step 3: Assemble the genome using Canu<br>```bash<br># Assuming Canu is installed, use the following command to assemble the genome<br>canu -p genome -d genome_assembly -nanopore-raw<br>sample_nanopore_reads.fastq<br># This will produce the assembly in the "genome_assembly" directory<br>```<br><br>Step 4 (Optional): Further Polishing

	You can further polish the assembly using tools like Racon or Medaka if necessary, using the assembled genome as input.<br><br>Always adjust the commands according to the actual location of the data and software on your system. Additionally, some tools may require additional setup and configuration steps or specific options based on the characteristics of the data and the nature of the genome being assembled.

Nanopore DNA sequencing data analysis **Case 8.2: Tandem repeats variation identification**

<table>
<tr><td>User's Inputs</td><td>You are a bioinformatician who specializes in writing code and performing analysis for a variety of bioinformatic data analysis tasks. Now I provide you with an Ubuntu 18.04 system where I save the following data, each line including the data path and data description.

sample_nanopore_reads.fastq: Raw Nanopore sequencing data in fastq format.
eference_genome.fasta: A reference genome in FASTA format, if a closely related reference is available and used as a guide during the assembly

I need you to help me plan what needs to be done in each step of the analysis process, and help me write the code for each step to install softwares, use tools and accomplish the following main goal:

To identify tandem repeats.</td></tr>
<tr><td>Generated Results by ChatGPT-3.5</td><td>To identify tandem repeats, you can follow the steps outlined below:

Step 1: Install necessary software
```bash
# Update system
sudo apt update && sudo apt upgrade

# Install required tools such as Tandem Repeats Finder (TRF) and Samtools
# Download TRF from the official website: https://tandem.bu.edu/trf/trf.download.html
# Samtools can be installed using:
sudo apt install samtools
```

Step 2: Preprocess the Nanopore reads (if necessary)
If the data needs preprocessing, you can employ quality control tools such as Porechop or Guppy to trim adapters and low-quality bases.

Step 3: Run Tandem Repeats Finder (TRF)
```bash
Run TRF on the Nanopore reads to detect tandem repeats
/path/to/trf sample_nanopore_reads.fastq 2 7 7 80 10 50 500 -l 7 -d -h
This command runs TRF with default parameters. You may modify the parameters based on your preferences and the nature of the data.
```

Step 4: Interpret the TRF output
The TRF output file contains information about identified tandem repeats. Additional post-processing, visualization, and downstream analysis can be performed as needed based on the specific research goals.

Always adjust the commands according to the actual location of the data and software on your system. Additionally, some tools may require additional setup and configuration steps.</td></tr>
</table>

PacBio DNA sequencing data analysis **Case 9.1: Genome assembly**

<table>
<tr><td>User's Inputs</td><td>You are a bioinformatician who specializes in writing code and performing analysis for a variety of bioinformatic data analysis tasks. Now I provide you with an Ubuntu 18.04 system where I save the following data, each line including the data path and data description.

sample_pacbio_reads.fastq: Raw PacBio sequencing data in fastq format.
eference_genome.fasta: A reference genome in FASTA format, if a closely related reference is available and used as a guide during the assembly

I need you to help me plan what needs to be done in each step of the analysis process, and help me write the code for each step to install softwares, use tools and accomplish the following main goal:

To assemble a genome from the PacBio sequencing data.</td></tr>
<tr><td>Generated Results by ChatGPT-3.5</td><td>To assemble a genome from PacBio sequencing data, you can follow the steps below:

Step 1: Install necessary software
```bash
# Update system
sudo apt update && sudo apt upgrade

# Install required tools such as Minimap2, Samtools, and Canu
sudo apt install minimap2
sudo apt install samtools
# Download and install Canu from the official website:
https://canu.readthedocs.io/en/latest/quick-start.html#installation
```

Step 2: Preprocess the PacBio reads and map to the reference genome (if available)
```bash
Map to a closely related reference genome (if available) using minimap2
minimap2 -ax map-pb reference_genome.fasta
sample_pacbio_reads.fastq > aligned_reads.sam
samtools view -S -b aligned_reads.sam > aligned_reads.bam
samtools sort aligned_reads.bam -o aligned_reads_sorted.bam
samtools index aligned_reads_sorted.bam
```

Step 3: Assemble the genome using Canu
```bash
Assuming Canu is installed, use the following command to assemble the genome
canu -p genome -d genome_assembly -pacbio-raw
sample_pacbio_reads.fastq
This will produce the assembly in the "genome_assembly" directory
```

Step 4 (Optional): Further Polishing</td></tr>
</table>

	You can further polish the assembly using tools like Arrow, Pilón, or Racon if necessary.<br><br>After performing these steps, you would have assembled a genome from PacBio sequencing data. Adjust the commands according to the actual location of the data and software on your system. Additionally, some tools may require additional setup and configuration steps or specific options based on the characteristics of the data and the nature of the genome being assembled.

RNA-Seq Analysis Case 10.1: Find differentially expressed genes

<table>
<tr>
<td>User's Inputs</td>
<td>You are a bioinformatician who specializes in writing code and performing analysis for a variety of bioinformatic data analysis tasks. Now I provide you with an Ubuntu 18.04 system where I save the following data, each line including the data path and data description.

SRR1374921.fastq.gz: single-end mouse rna-seq reads, replicate 1 in LoGlu group
SRR1374922.fastq.gz: single-end mouse rna-seq reads, replicate 2 in LoGlu group
SRR1374923.fastq.gz: single-end mouse rna-seq reads, replicate 1 in HiGlu group
SRR1374924.fastq.gz: single-end mouse rna-seq reads, replicate 2 in HiGlu group
TruSeq3-SE.fa: trimming adapter
mm39.fa: mouse mm39 genome fasta
mm39.ncbiRefSeq.gtf: mouse mm39 genome annotation

I need you to help me plan what needs to be done in each step of the analysis process, and help me write the code for each step to install softwares, use tools and accomplish the following main goal:

Find the differentially expressed genes</td>
</tr>
<tr>
<td>Generated Results by ChatGPT-3.5</td>
<td>To find differentially expressed genes from the RNA-seq data, you can follow the steps below:

Step 1: Install necessary software
```bash
# Update system
sudo apt update && sudo apt upgrade

Install required tools such as HISAT2, StringTie, and DESeq2 using conda
Assuming conda is installed, you could create and activate a new environment while installing the necessary tools using:
conda create -n rna_seq_env hisat2 stringtie samtools subread bioconductor-deseq2
conda activate rna_seq_env
```

Step 2: Preprocess and align RNA-seq reads
```bash
Index the reference genome using HISAT2
hisat2-build mm39.fa mm39_index

Align reads to the reference genome
hisat2 -x mm39_index -U SRR1374921.fastq.gz -S SRR1374921_aligned.sam</td>
</tr>
</table>

hisat2 -x mm39_index -U SRR1374922.fastq.gz -S
SRR1374922_aligned.sam
hisat2 -x mm39_index -U SRR1374923.fastq.gz -S
SRR1374923_aligned.sam
hisat2 -x mm39_index -U SRR1374924.fastq.gz -S
SRR1374924_aligned.sam
```

Step 3: Assemble transcripts and estimate their abundance
```bash
# Convert SAM to BAM and sort
samtools view -S -b SRR1374921_aligned.sam >
SRR1374921_aligned.bam
samtools sort SRR1374921_aligned.bam -o SRR1374921_sorted.bam
samtools index SRR1374921_sorted.bam

# Use StringTie to assemble transcripts and estimate their abundance
stringtie SRR1374921_sorted.bam -G mm39.ncbiRefSeq.gtf -o
SRR1374921.gtf -l SRR1374921
stringtie SRR1374922_sorted.bam -G mm39.ncbiRefSeq.gtf -o
SRR1374922.gtf -l SRR1374922
stringtie SRR1374923_sorted.bam -G mm39.ncbiRefSeq.gtf -o
SRR1374923.gtf -l SRR1374923
stringtie SRR1374924_sorted.bam -G mm39.ncbiRefSeq.gtf -o
SRR1374924.gtf -l SRR1374924
```

Step 4: Differential expression analysis using DESeq2
```bash
R
# Inside R environment
library("DESeq2")
# Read count data
data <- as.data.frame(counts(cbind(SRR1374921, SRR1374922,
SRR1374923, SRR1374924)))
# Create a metadata table
metadata <- data.frame(row.names=colnames(data), condition=c("LoGlu",
"LoGlu", "HiGlu", "HiGlu"))
# Create DESeqDataSet
dds <- DESeqDataSetFromMatrix(countData = data, colData = metadata,
design = ~condition)
# Run differential expression analysis
dds <- DESeq(dds)
# Extract differentially expressed genes
res <- results(dds)
```

After performing these steps, you will obtain a list of differentially expressed genes from your RNA-seq data. Always adjust the commands according to the actual location of the data and software on your system. Additionally, some tools may require additional setup and configuration steps.
```

RNA-Seq Analysis Case 10.2: Identify top5 down-regulated genes in HiGlu group

<table>
<tr><td>User's Inputs</td><td>You are a bioinformatician who specializes in writing code and performing analysis for a variety of bioinformatic data analysis tasks. Now I provide you with an Ubuntu 18.04 system where I save the following data, each line including the data path and data description.

SRR1374921.fastq.gz: single-end mouse rna-seq reads, replicate 1 in LoGlu group
SRR1374922.fastq.gz: single-end mouse rna-seq reads, replicate 2 in LoGlu group
SRR1374923.fastq.gz: single-end mouse rna-seq reads, replicate 1 in HiGlu group
SRR1374924.fastq.gz: single-end mouse rna-seq reads, replicate 2 in HiGlu group
TruSeq3-SE.fa: trimming adapter
mm39.fa: mouse mm39 genome fasta
mm39.ncbiRefSeq.gtf: mouse mm39 genome annotation

I need you to help me plan what needs to be done in each step of the analysis process, and help me write the code for each step to install softwares, use tools and accomplish the following main goal:

Identify top5 down-regulated genes in HiGlu group</td></tr>
<tr><td>Generated Results by ChatGPT-3.5</td><td>To identify the top 5 down-regulated genes in the HiGlu group, you can follow the steps below using a popular toolset for RNA-seq analysis:

Step 1: Install necessary software

First, ensure you have the required tools like HISAT2, StringTie, Samtools, and R with DESeq2.

```bash
# Update system
sudo apt update && sudo apt upgrade

# Install required tools such as HISAT2, StringTie, Samtools, and R with DESeq2
sudo apt install hisat2
sudo apt install samtools
# Install R: https://cran.r-project.org/
# Install R packages DESeq2 and Bioconductor: https://bioconductor.org/packages/release/bioc/html/DESeq2.html
```

Step 2: Preprocess and align RNA-seq reads

Use HISAT2 to align the reads to the reference genome, MM39.

```bash
Index the reference genome using HISAT2
hisat2-build mm39.fa mm39_index

Align reads to the reference genome</td></tr>
</table>

```
hisat2 -x mm39_index -U SRR1374923.fastq.gz -S
SRR1374923_aligned.sam
hisat2 -x mm39_index -U SRR1374924.fastq.gz -S
SRR1374924_aligned.sam
```

Step 3: Assemble transcripts and estimate their abundance

Use StringTie to assemble transcripts and estimate their abundance.

```bash
# Convert SAM to BAM and sort
samtools view -S -b SRR1374923_aligned.sam >
SRR1374923_aligned.bam
samtools sort SRR1374923_aligned.bam -o SRR1374923_sorted.bam
samtools index SRR1374923_sorted.bam

samtools view -S -b SRR1374924_aligned.sam >
SRR1374924_aligned.bam
samtools sort SRR1374924_aligned.bam -o SRR1374924_sorted.bam
samtools index SRR1374924_sorted.bam

# Use StringTie to assemble transcripts and estimate their abundance
stringtie SRR1374923_sorted.bam -G mm39.ncbiRefSeq.gtf -o
SRR1374923.gtf -l SRR1374923
stringtie SRR1374924_sorted.bam -G mm39.ncbiRefSeq.gtf -o
SRR1374924.gtf -l SRR1374924
```

Step 4: Differential expression analysis using DESeq2 in R

```R
# Run R
R

# Inside R environment
library("DESeq2")

# Assuming you have prepared count tables for HiGlu and LoGlu, perform
the following in R
# Read count data
data <- as.data.frame(counts(cbind(SRR1374923, SRR1374924)))

# Create a metadata table
metadata <- data.frame(row.names=colnames(data), group=c("HiGlu",
"HiGlu"))

# Create DESeqDataSet
dds <- DESeqDataSetFromMatrix(countData = data, colData = metadata,
design = ~group)

# Run differential expression analysis
dds <- DESeq(dds)
```

	# Extract differentially expressed genes<br>res <- results(dds)<br><br># Get top 5 down-regulated genes in the HiGlu group<br>top_downregulated_genes <- head(res[order(res$log2FoldChange),], n=5)<br>```<br><br>After performing these steps in R, you'll have identified the top 5 down-regulated genes in the HiGlu group. Always adjust the commands according to the actual location of the data and software on your system. Additionally, some tools may require additional setup and configuration steps, and R packages may need to be installed before running the analysis.

RNA-Seq Analysis   Case 10.3: Predict Fusion genes

**User's Inputs**	You are a bioinformatician who specializes in writing code and performing analysis for a variety of bioinformatic data analysis tasks. Now I provide you with an Ubuntu 18.04 system where I save the following data, each line including the data path and data description.<br><br>rnaseq_1.fastq.gz: RNA-Seq read 1 data (left read)<br>rnaseq_2.fastq.gz: RNA-Seq read 2 data (right read)<br>CTAT_HumanFusionLib.mini.dat.gz: a small fusion annotation library<br>minigenome.fa: small genome sequence consisting of ~750 genes<br>minigenome.gtf: transcript structure annotations for these genes<br><br>I need you to help me plan what needs to be done in each step of the analysis process, and help me write the code for each step to install softwares, use tools and accomplish the following main goal:<br><br>Predict Fusion genes
**Generated Results by ChatGPT-3.5**	To predict fusion genes from the provided data, you can follow the steps below using FusionMap, a popular tool for fusion gene detection.<br><br>Step 1: Install necessary software<br><br>First, ensure you have the required tools like FusionMap.<br><br>```bash<br># Update system<br>sudo apt update && sudo apt upgrade<br><br># Install required tools such as FusionMap<br># You can download FusionMap from its official website: http://omics.bjcancer.org/download/FusionMap/<br># After downloading, install and set up FusionMap as per the provided installation instructions<br>```<br><br>Step 2: Preprocess RNA-Seq reads<br><br>For FusionMap, the RNA-Seq reads need to be preprocessed and aligned.<br><br>```bash<br># Assuming your reads are paired-end, trim adapter sequences using Cutadapt or Trimmomatic<br># Align the preprocessed reads to the small genome using a suitable aligner like HISAT2 or STAR<br># Convert the aligned reads to BAM format if not already in BAM format<br>```<br><br>Step 3: Run FusionMap<br><br>```bash<br># Assuming FusionMap is set up correctly, run the fusion gene detection analysis

	/path/to/fusionmap -1 rnaseq_1.fastq.gz -2 rnaseq_2.fastq.gz -d CTAT_HumanFusionLib.mini.dat.gz -C minigenome.fa -G minigenome.gtf -O fusion_results_output_directory<br>```<br><br>After performing these steps, FusionMap will identify potential fusion genes. Ensure to adjust commands according to the actual location of the data and software on your system. Additionally, some tools may require additional setup and configuration. Also, ensure that the aligner and FusionMap are appropriately configured based on the specific characteristics of your data and research goals.

RNA-Seq Analysis **Case 10.4: isoform expression**

<table>
<tr>
<td>User's Inputs</td>
<td>You are a bioinformatician who specializes in writing code and performing analysis for a variety of bioinformatic data analysis tasks. Now I provide you with an Ubuntu 18.04 system where I save the following data, each line including the data path and data description.

SRR14463651_1.fastq.gz: paired end RNA sequencing data from human lymphoma cell, read 1 of lymphoma samples replicate 1
SRR14463651_2.fastq.gz: paired end RNA sequencing data from human lymphoma cell, read 2 of lymphoma samples replicate 1
SRR14463652_1.fastq.gz: paired end RNA sequencing data from human lymphoma cell, read 1 of lymphoma samples replicate 2
SRR14463652_2.fastq.gz: paired end RNA sequencing data from human lymphoma cell, read 2 of lymphoma samples replicate 2
SRR14463680_1.fastq.gz: paired end RNA sequencing data from human lymphoma cell, read 1 of control samples replicate 1
SRR14463680_2.fastq.gz: paired end RNA sequencing data from human lymphoma cell, read 2 of control samples replicate 1
SRR14463681_1.fastq.gz: paired end RNA sequencing data from human lymphoma cell, read 1 of control samples replicate 2
SRR14463681_2.fastq.gz: paired end RNA sequencing data from human lymphoma cell, read 2 of control samples replicate 2
gencode.v44.transcripts.fa: transcriptome annotation
TruSeq3-PE.fa: trimming adapter

I need you to help me plan what needs to be done in each step of the analysis process, and help me write the code for each step to install softwares, use tools and accomplish the following main goal:

quantify RNA isoform expression based on transcriptome annotation</td>
</tr>
<tr>
<td>Generated Results by ChatGPT-3.5</td>
<td>To quantify RNA isoform expression based on the provided transcriptome annotation and RNA sequencing data, you can follow the steps below using widely used tools such as HISAT2, StringTie, and Ballgown.

Step 1: Install necessary software

Ensure you have the required tools such as HISAT2, StringTie, and Ballgown. Also, install other tools if necessary using package managers like apt or installation provided by the tool's official website.

```bash
# Update system
sudo apt update && sudo apt upgrade

# Install required tools such as HISAT2, StringTie, and SAMtools
sudo apt install hisat2
sudo apt install samtools
# Install R: https://cran.r-project.org/
# Install required R packages for RNA-seq analysis including Ballgown
```</td>
</tr>
</table>

Step 2: Preprocess RNA-Seq reads – align reads to the transcriptome

Align the RNA-Seq reads to the transcriptome using HISAT2.

```bash
# Index the transcriptome using HISAT2
hisat2-build gencode.v44.transcripts.fa gencode_index

# Align the reads from each sample to the transcriptome
hisat2 -x gencode_index -1 SRR14463651_1.fastq.gz -2 SRR14463651_2.fastq.gz -S sample1.sam
hisat2 -x gencode_index -1 SRR14463652_1.fastq.gz -2 SRR14463652_2.fastq.gz -S sample2.sam
hisat2 -x gencode_index -1 SRR14463680_1.fastq.gz -2 SRR14463680_2.fastq.gz -S control1.sam
hisat2 -x gencode_index -1 SRR14463681_1.fastq.gz -2 SRR14463681_2.fastq.gz -S control2.sam
```

Step 3: Assemble transcripts and quantify their expression

Use StringTie to assemble the transcripts and quantify their expression.

```bash
# Convert SAM to BAM, sort, and index
samtools view -S -b sample1.sam > sample1.bam
samtools sort sample1.bam -o sample1_sorted.bam
samtools index sample1_sorted.bam

samtools view -S -b sample2.sam > sample2.bam
samtools sort sample2.bam -o sample2_sorted.bam
samtools index sample2_sorted.bam

samtools view -S -b control1.sam > control1.bam
samtools sort control1.bam -o control1_sorted.bam
samtools index control1_sorted.bam

samtools view -S -b control2.sam > control2.bam
samtools sort control2.bam -o control2_sorted.bam
samtools index control2_sorted.bam

# Use StringTie to assemble transcripts and quantify their expression
stringtie sample1_sorted.bam -G gencode.v44.transcripts.fa -o sample1.gtf -l sample1
stringtie sample2_sorted.bam -G gencode.v44.transcripts.fa -o sample2.gtf -l sample2
stringtie control1_sorted.bam -G gencode.v44.transcripts.fa -o control1.gtf -l control1
stringtie control2_sorted.bam -G gencode.v44.transcripts.fa -o control2.gtf -l control2
```

Step 4: Perform differential isoform expression analysis

Using Ballgown in R, perform differential isoform expression analysis.

```R
# Run R
R

# Inside R environment
library("ballgown")

# Load data into ballgown
myDir <- "path_to_directory_containing_sample_and_control_gtf_files"
myFiles <- list.files(path=myDir, pattern="\\.gtf$")
bg = ballgown(dataDir=myDir, samplePattern='sample', pData=pheno_data)

# Test for differential expression
results_splicing = stattest(bg, feature="transcript", meas="Sashimi")

# Extract significant isoform expression changes based on testing
significant_results = subset(results_splicing, qval < 0.05)
```

After following these steps, you'll have quantified RNA isoform expression based on the transcriptome annotation provided. Adjust the commands according to the actual location of the data and software on your system. Additionally, be sure to install any required R packages and set up the R environment appropriately before using Ballgown.

RNA-seq Analysis   Case 10.5: splicing analysis

<table>
<tr><td>User's Inputs</td><td>You are a bioinformatician who specializes in writing code and performing analysis for a variety of bioinformatic data analysis tasks. Now I provide you with an Ubuntu 18.04 system where I save the following data, each line including the data path and data description.

SRR14463651_1.fastq.gz: paired end RNA sequencing data from human lymphoma cell, read 1 of lymphoma samples replicate 1
SRR14463651_2.fastq.gz: paired end RNA sequencing data from human lymphoma cell, read 2 of lymphoma samples replicate 1
SRR14463652_1.fastq.gz: paired end RNA sequencing data from human lymphoma cell, read 1 of lymphoma samples replicate 2
SRR14463652_2.fastq.gz: paired end RNA sequencing data from human lymphoma cell, read 2 of lymphoma samples replicate 2
SRR14463680_1.fastq.gz: paired end RNA sequencing data from human lymphoma cell, read 1 of control samples replicate 1
SRR14463680_2.fastq.gz: paired end RNA sequencing data from human lymphoma cell, read 2 of control samples replicate 1
SRR14463681_1.fastq.gz: paired end RNA sequencing data from human lymphoma cell, read 1 of control samples replicate 2
SRR14463681_2.fastq.gz: paired end RNA sequencing data from human lymphoma cell, read 2 of control samples replicate 2
hg38.fa: reference genome
gencode.v38.annotation.gtf: gene annotation
TruSeq3-PE.fa: trimming adapter

I need you to help me plan what needs to be done in each step of the analysis process, and help me write the code for each step to install softwares, use tools and accomplish the following main goal:

identify alternative splicing events in lymphoma and control samples and compare them to get differentially spliced events</td></tr>
<tr><td>Generated Results by ChatGPT-3.5</td><td>To identify alternative splicing events in lymphoma and control samples and compare them to obtain differentially spliced events, you can follow the steps below using tools like HISAT2, StringTie, and rMATS.

Step 1: Install necessary software

First, ensure you have the required tools such as HISAT2, StringTie, rMATS, and other necessary tools. You can install these tools using package managers or by following installation instructions on their official websites.

```bash
# Update system
sudo apt update && sudo apt upgrade

# Install required tools such as HISAT2, StringTie, and rMATS
sudo apt install hisat2
sudo apt install stringtie
# Install rMATS from its official website: https://rnaseq-mats.sourceforge.net/</td></tr>
</table>

```

Step 2: Preprocess RNA-Seq reads – align reads to the reference genome

Align the RNA-Seq reads to the reference genome using HISAT2.

```bash
# Index the reference genome using HISAT2
hisat2-build hg38.fa hg38_index

# Align the reads from each sample to the reference genome
hisat2 -x hg38_index -1 SRR14463651_1.fastq.gz -2
SRR14463651_2.fastq.gz -S lymphoma_replicate1.sam
hisat2 -x hg38_index -1 SRR14463652_1.fastq.gz -2
SRR14463652_2.fastq.gz -S lymphoma_replicate2.sam
hisat2 -x hg38_index -1 SRR14463680_1.fastq.gz -2
SRR14463680_2.fastq.gz -S control_replicate1.sam
hisat2 -x hg38_index -1 SRR14463681_1.fastq.gz -2
SRR14463681_2.fastq.gz -S control_replicate2.sam
```

Step 3: Assemble and quantitate transcripts using StringTie

Use StringTie to assemble and quantitate transcripts for each sample.

```bash
stringtie lymphoma_replicate1.sam -G gencode.v38.annotation.gtf -o
lymphoma_replicate1.gtf
stringtie lymphoma_replicate2.sam -G gencode.v38.annotation.gtf -o
lymphoma_replicate2.gtf
stringtie control_replicate1.sam -G gencode.v38.annotation.gtf -o
control_replicate1.gtf
stringtie control_replicate2.sam -G gencode.v38.annotation.gtf -o
control_replicate2.gtf
```

Step 4: Identify differentially spliced events using rMATS

```bash
# Assuming rMATS is installed and set up properly
python /path/to/rMATS/rMATS.py \
  --b1 lymphoma_replicate1.bam lymphoma_replicate2.bam \
  --b2 control_replicate1.bam control_replicate2.bam \
  --gtf gencode.v38.annotation.gtf \
  --od differential_splicing_results
```

After performing these steps, you will obtain differentially spliced events. Adjust the commands according to the actual location of the data and software on your system. Additionally, some tools may require additional setup and configuration steps.
```

RNA-seq Analysis Case 10.6: APA analysis

<table>
<tr><td>User's Inputs</td><td>You are a bioinformatician who specializes in writing code and performing analysis for a variety of bioinformatic data analysis tasks. Now I provide you with an Ubuntu 18.04 system where I save the following data, each line including the data path and data description.

SRR14463651_1.fastq.gz: paired end RNA sequencing data from human lymphoma cell, read 1 of lymphoma samples replicate 1
SRR14463651_2.fastq.gz: paired end RNA sequencing data from human lymphoma cell, read 2 of lymphoma samples replicate 1
SRR14463652_1.fastq.gz: paired end RNA sequencing data from human lymphoma cell, read 1 of lymphoma samples replicate 2
SRR14463652_2.fastq.gz: paired end RNA sequencing data from human lymphoma cell, read 2 of lymphoma samples replicate 2
SRR14463680_1.fastq.gz: paired end RNA sequencing data from human lymphoma cell, read 1 of control samples replicate 1
SRR14463680_2.fastq.gz: paired end RNA sequencing data from human lymphoma cell, read 2 of control samples replicate 1
SRR14463681_1.fastq.gz: paired end RNA sequencing data from human lymphoma cell, read 1 of control samples replicate 2
SRR14463681_2.fastq.gz: paired end RNA sequencing data from human lymphoma cell, read 2 of control samples replicate 2
hg38.fa: reference genome
gencode.v38.annotation.gtf: gene annotation
TruSeq3-PE.fa: trimming adapter

I need you to help me plan what needs to be done in each step of the analysis process, and help me write the code for each step to install softwares, use tools and accomplish the following main goal:

identify and quantify alternative polyadenylation between lymphoma and control samples</td></tr>
<tr><td>Generated Results by ChatGPT-3.5</td><td>To identify and quantify alternative polyadenylation (APA) between lymphoma and control samples, follow these steps using tools like STAR, RSEM, and APAtrap.

Step 1: Install necessary software

Ensure you have the required tools such as STAR, RSEM, and APAtrap. You can install these tools using package managers or by following installation instructions on their official websites.

```bash
# Update system
sudo apt update && sudo apt upgrade

Install required tools such as STAR, RSEM, APAtrap
STAR and RSEM details: https://github.com/alexdobin/STAR, https://deweylab.github.io/RSEM/
APAtrap details: https://github.com/lulab/APAtrap
```</td></tr>
</table>

Step 2: Preprocess RNA-Seq reads – align reads to the reference genome

Align the RNA-Seq reads to the reference genome using STAR.

```bash
# Index the reference genome using STAR
STAR --runMode genomeGenerate --genomeDir
/path_to_genome_directory --genomeFastaFiles hg38.fa --sjdbGTFfile
gencode.v38.annotation.gtf

# Align the reads from each sample to the reference genome
STAR --runThreadN 4 --genomeDir /path_to_genome_directory --
readFilesIn SRR14463651_1.fastq.gz SRR14463651_2.fastq.gz --
outSAMtype BAM SortedByCoordinate --quantMode TranscriptomeSAM --
outFileNamePrefix lymphoma_rep1
STAR --runThreadN 4 --genomeDir /path_to_genome_directory --
readFilesIn SRR14463652_1.fastq.gz SRR14463652_2.fastq.gz --
outSAMtype BAM SortedByCoordinate --quantMode TranscriptomeSAM --
outFileNamePrefix lymphoma_rep2
STAR --runThreadN 4 --genomeDir /path_to_genome_directory --
readFilesIn SRR14463680_1.fastq.gz SRR14463680_2.fastq.gz --
outSAMtype BAM SortedByCoordinate --quantMode TranscriptomeSAM --
outFileNamePrefix control_rep1
STAR --runThreadN 4 --genomeDir /path_to_genome_directory --
readFilesIn SRR14463681_1.fastq.gz SRR14463681_2.fastq.gz --
outSAMtype BAM SortedByCoordinate --quantMode TranscriptomeSAM --
outFileNamePrefix control_rep2
```

Step 3: Quantify gene and isoform expression using RSEM

Quantify gene and isoform expression using RSEM.

```bash
rsem-calculate-expression --paired-end
lymphoma_rep1Aligned.toTranscriptome.out.bam
/path_to_genome_directory gencode.v38.annotation.gtf lymphoma_rep1
rsem-calculate-expression --paired-end
lymphoma_rep2Aligned.toTranscriptome.out.bam
/path_to_genome_directory gencode.v38.annotation.gtf lymphoma_rep2
rsem-calculate-expression --paired-end
control_rep1Aligned.toTranscriptome.out.bam /path_to_genome_directory
gencode.v38.annotation.gtf control_rep1
rsem-calculate-expression --paired-end
control_rep2Aligned.toTranscriptome.out.bam /path_to_genome_directory
gencode.v38.annotation.gtf control_rep2
```

Step 4: Identify and quantify alternative polyadenylation sites using APAtrap

```bash
# Assuming APAtrap is installed and set up properly
```

	APATrap --minCoverage 10 --condition1 lymphoma_rep1.isoforms.results lymphoma_rep2.isoforms.results --condition2 control_rep1.isoforms.results control_rep2.isoforms.results --output-dir APAtrap_output<br>```<br><br>After performing these steps, you will have identified and quantified alternative polyadenylation between lymphoma and control samples using APAtrap. Always adjust the commands according to the actual location of the data and software on your system. Additionally, some tools may require additional setup and configuration steps.

RNA-seq Analysis Case 10.7: RNA editing

<table>
<tr><td>User's Inputs</td><td>You are a bioinformatician who specializes in writing code and performing analysis for a variety of bioinformatic data analysis tasks. Now I provide you with an Ubuntu 18.04 system where I save the following data, each line including the data path and data description.

SRR14463651_1.fastq.gz: paired end RNA sequencing data from human lymphoma cell, read 1 of lymphoma samples replicate 1
SRR14463651_2.fastq.gz: paired end RNA sequencing data from human lymphoma cell, read 2 of lymphoma samples replicate 1
SRR14463652_1.fastq.gz: paired end RNA sequencing data from human lymphoma cell, read 1 of lymphoma samples replicate 2
SRR14463652_2.fastq.gz: paired end RNA sequencing data from human lymphoma cell, read 2 of lymphoma samples replicate 2
SRR14463680_1.fastq.gz: paired end RNA sequencing data from human lymphoma cell, read 1 of control samples replicate 1
SRR14463680_2.fastq.gz: paired end RNA sequencing data from human lymphoma cell, read 2 of control samples replicate 1
SRR14463681_1.fastq.gz: paired end RNA sequencing data from human lymphoma cell, read 1 of control samples replicate 2
SRR14463681_2.fastq.gz: paired end RNA sequencing data from human lymphoma cell, read 2 of control samples replicate 2
hg38.fa: reference genome
gencode.v38.annotation.gtf: gene annotation
TruSeq3-PE.fa: trimming adapter

I need you to help me plan what needs to be done in each step of the analysis process, and help me write the code for each step to install softwares, use tools and accomplish the following main goal:

identifying RNA edting events in lymphoma and control samples</td></tr>
<tr><td>Generated Results by ChatGPT-3.5</td><td>To identify RNA editing events in lymphoma and control samples, you can follow these steps using tools like HISAT2, SAMtools, GATK, and Varscan.

Step 1: Install necessary software

Ensure that you have the required tools such as HISAT2, SAMtools, GATK, and Varscan. You can install these tools using package managers or following the installation instructions provided on their official websites.

```bash
# Update system
sudo apt update && sudo apt upgrade

# Install required tools such as HISAT2, SAMtools, GATK, and Varscan
sudo apt install hisat2
sudo apt install samtools
sudo apt install default-jre # Install Java Runtime Environment (if not already installed)
# Install GATK: https://gatk.broadinstitute.org/hc/en-us/articles/360037562611-Getting-started-with-GATK4
# Install Varscan: http://varscan.sourceforge.net
```</td></tr>
</table>

Step 2: Preprocess RNA-Seq reads – align reads to the reference genome

Align the RNA-Seq reads to the reference genome using HISAT2 and process the alignments using SAMtools.

```bash
# Index the reference genome using HISAT2
hisat2-build hg38.fa hg38_index

# Align the reads from each sample to the reference genome
hisat2 -x hg38_index -1 SRR14463651_1.fastq.gz -2 SRR14463651_2.fastq.gz -S lymphoma_replicate1.sam
hisat2 -x hg38_index -1 SRR14463652_1.fastq.gz -2 SRR14463652_2.fastq.gz -S lymphoma_replicate2.sam
hisat2 -x hg38_index -1 SRR14463680_1.fastq.gz -2 SRR14463680_2.fastq.gz -S control_replicate1.sam
hisat2 -x hg38_index -1 SRR14463681_1.fastq.gz -2 SRR14463681_2.fastq.gz -S control_replicate2.sam

# Convert SAM to BAM, sort, and index
samtools view -S -b lymphoma_replicate1.sam > lymphoma_replicate1.bam
samtools sort lymphoma_replicate1.bam -o lymphoma_replicate1_sorted.bam
samtools index lymphoma_replicate1_sorted.bam

samtools view -S -b lymphoma_replicate2.sam > lymphoma_replicate2.bam
samtools sort lymphoma_replicate2.bam -o lymphoma_replicate2_sorted.bam
samtools index lymphoma_replicate2_sorted.bam

samtools view -S -b control_replicate1.sam > control_replicate1.bam
samtools sort control_replicate1.bam -o control_replicate1_sorted.bam
samtools index control_replicate1_sorted.bam

samtools view -S -b control_replicate2.sam > control_replicate2.bam
samtools sort control_replicate2.bam -o control_replicate2_sorted.bam
samtools index control_replicate2_sorted.bam
```

Step 3: Identify RNA editing events using GATK and Varscan

Use GATK and Varscan to identify RNA editing events.

```bash
# Assuming that RNA editing calling is treated as SNV calling using GATK HaplotypeCaller
gatk HaplotypeCaller -R hg38.fa -I lymphoma_replicate1_sorted.bam -O lymphoma_replicate1_variants.vcf
gatk HaplotypeCaller -R hg38.fa -I lymphoma_replicate2_sorted.bam -O lymphoma_replicate2_variants.vcf
gatk HaplotypeCaller -R hg38.fa -I control_replicate1_sorted.bam -O control_replicate1_variants.vcf
```

	gatk HaplotypeCaller -R hg38.fa -I control_replicate2_sorted.bam -O control_replicate2_variants.vcf<br><br># Using Varscan to identify RNA editing events<br>java -jar VarScan.jar mpileup2snp lymphoma_replicate1_sorted.bam --output-vcf 1 --vcf-sample lymphoma_replicate1 > lymphoma_replicate1_editing.vcf<br>java -jar VarScan.jar mpileup2snp lymphoma_replicate2_sorted.bam --output-vcf 1 --vcf-sample lymphoma_replicate2 > lymphoma_replicate2_editing.vcf<br>java -jar VarScan.jar mpileup2snp control_replicate1_sorted.bam --output-vcf 1 --vcf-sample control_replicate1 > control_replicate1_editing.vcf<br>java -jar VarScan.jar mpileup2snp control_replicate2_sorted.bam --output-vcf 1 --vcf-sample control_replicate2 > control_replicate2_editing.vcf<br>```<br><br>After executing these steps, you will have identified RNA editing events in lymphoma and control samples using GATK and Varscan. Always adjust the commands according to the actual location of the data and software on your system. Additionally, some tools may require additional setup and configuration steps.

RNA-seq Analysis   Case 10.8: circular RNA analysis

<table>
<tr>
<td>User's Inputs</td>
<td>You are a bioinformatician who specializes in writing code and performing analysis for a variety of bioinformatic data analysis tasks. Now I provide you with an Ubuntu 18.04 system where I save the following data, each line including the data path and data description.

SRR14463651_1.fastq.gz: paired end RNA sequencing data from human lymphoma cell, read 1 of lymphoma samples replicate 1
SRR14463651_2.fastq.gz: paired end RNA sequencing data from human lymphoma cell, read 2 of lymphoma samples replicate 1
SRR14463652_1.fastq.gz: paired end RNA sequencing data from human lymphoma cell, read 1 of lymphoma samples replicate 2
SRR14463652_2.fastq.gz: paired end RNA sequencing data from human lymphoma cell, read 2 of lymphoma samples replicate 2
SRR14463680_1.fastq.gz: paired end RNA sequencing data from human lymphoma cell, read 1 of control samples replicate 1
SRR14463680_2.fastq.gz: paired end RNA sequencing data from human lymphoma cell, read 2 of control samples replicate 1
SRR14463681_1.fastq.gz: paired end RNA sequencing data from human lymphoma cell, read 1 of control samples replicate 2
SRR14463681_2.fastq.gz: paired end RNA sequencing data from human lymphoma cell, read 2 of control samples replicate 2
hg38.fa: reference genome
gencode.v38.annotation.gtf: gene annotation
TruSeq3-PE.fa: trimming adapter

I need you to help me plan what needs to be done in each step of the analysis process, and help me write the code for each step to install softwares, use tools and accomplish the following main goal:

identify and quantify circular RNA in lymphoma and control samples</td>
</tr>
<tr>
<td>Generated Results by ChatGPT-3.5</td>
<td>To identify and quantify circular RNA in lymphoma and control samples, you can follow these steps using tools such as STAR, DCC, and CIRI2.

Step 1: Install necessary software

Ensure that you have the required tools such as STAR, DCC (Detection of circRNA using CIRI), and CIRI2. You can install these tools using package managers or by following installation instructions provided on their official websites.

```bash
# Update system
sudo apt update && sudo apt upgrade

# Install required tools such as STAR
sudo apt install STAR

Install other tools such as DCC and CIRI2 as per their respective documentation
```

Step 2: Preprocess RNA-Seq reads – align reads to the reference genome</td>
</tr>
</table>

Align the RNA-Seq reads to the reference genome using STAR.

```bash
# Index the reference genome using STAR
STAR --runMode genomeGenerate --genomeDir
/path_to_genome_directory --genomeFastaFiles hg38.fa --sjdbGTFfile
gencode.v38.annotation.gtf

# Align the reads from each sample to the reference genome
STAR --runThreadN 4 --genomeDir /path_to_genome_directory --
readFilesIn SRR14463651_1.fastq.gz SRR14463651_2.fastq.gz --
outSAMtype BAM SortedByCoordinate --outFileNamePrefix
lymphoma_rep1
STAR --runThreadN 4 --genomeDir /path_to_genome_directory --
readFilesIn SRR14463652_1.fastq.gz SRR14463652_2.fastq.gz --
outSAMtype BAM SortedByCoordinate --outFileNamePrefix
lymphoma_rep2
STAR --runThreadN 4 --genomeDir /path_to_genome_directory --
readFilesIn SRR14463680_1.fastq.gz SRR14463680_2.fastq.gz --
outSAMtype BAM SortedByCoordinate --outFileNamePrefix control_rep1
STAR --runThreadN 4 --genomeDir /path_to_genome_directory --
readFilesIn SRR14463681_1.fastq.gz SRR14463681_2.fastq.gz --
outSAMtype BAM SortedByCoordinate --outFileNamePrefix control_rep2
```

Step 3: Identify circular RNA using CIRI2

```bash
# Assuming CIRI2 is installed and set up properly
CIRI2 -I lymphoma_rep1Aligned.sortedByCoord.out.bam -O
lymphoma_rep1_circRNA.txt
CIRI2 -I lymphoma_rep2Aligned.sortedByCoord.out.bam -O
lymphoma_rep2_circRNA.txt
CIRI2 -I control_rep1Aligned.sortedByCoord.out.bam -O
control_rep1_circRNA.txt
CIRI2 -I control_rep2Aligned.sortedByCoord.out.bam -O
control_rep2_circRNA.txt
```

Step 4: Quantify circular RNA using DCC

Quantify the circRNA expression and perform differential circular RNA analysis using DCC.

```bash
# Assuming DCC is installed and set up properly
dcc quantify -l lymphoma_rep1_circRNA.txt -w control_rep1_circRNA.txt -o
quantification_l1vsC1
dcc quantify -l lymphoma_rep2_circRNA.txt -w control_rep2_circRNA.txt -o
quantification_l2vsC2
```

	After performing these steps, you will have identified and quantified circular RNA in lymphoma and control samples using CIRI2 and DCC. Always adjust the commands according to the actual location of the data and software on your system. Additionally, some tools may require additional setup and configuration steps.

Small RNA-seq Analysis **Case 11.1 and Case 11.2: miRNA analysis**

**User's Inputs**	You are a bioinformatician who specializes in writing code and performing analysis for a variety of bioinformatic data analysis tasks. Now I provide you with an Ubuntu 18.04 system where I save the following data, each line including the data path and data description.<br><br>SRR10915820.fastq.gz: single end small RNA sequencing data from human cell line, control samples replicate 1<br>SRR10915821.fastq.gz: single end small RNA sequencing data from human cell line, control samples replicate 1<br>SRR10915822.fastq.gz: single end small RNA sequencing data from human cell line, control samples replicate 1<br>SRR10915823.fastq.gz: single end small RNA sequencing data from human cell line, control samples replicate 1<br>hsa.gff3: microRNA genomic coordinate from miRbase<br>TGGAATTCTCGGGTGCCAAGG: adaptor sequence<br>hg38.fa: the reference genome<br><br>I need you to help me plan what needs to be done in each step of the analysis process, and help me write the code for each step to install softwares, use tools and accomplish the following main goal:<br><br>quantify microRNA expression based on miRbase and predict novel microRNA
**Generated Results by ChatGPT-3.5**	Certainly! To achieve your main goal of quantifying microRNA expression based on miRbase and predicting novel microRNAs, several steps are involved. Below is a plan for the analysis process and the corresponding code for each step.<br><br>Step 1: Data preprocessing<br>- Install necessary software and tools like FastQC, Cutadapt, and Bowtie for quality control and adapter trimming.<br>```bash<br># Install FastQC<br>sudo apt install fastqc<br><br># Install Cutadapt<br>sudo apt install cutadapt<br><br># Install Bowtie<br>sudo apt install bowtie<br>```<br><br>Step 2: Preprocessing small RNA data<br>- Perform quality control using FastQC on all FASTQ files.<br>```bash<br># Run FastQC on all files<br>fastqc SRR10915820.fastq.gz SRR10915821.fastq.gz SRR10915822.fastq.gz SRR10915823.fastq.gz<br>```<br><br>- Trim adapters and filter low-quality bases using Cutadapt.<br>```bash

	# Trim adapters and filter low-quality bases<br>cutadapt -a TGGAATTCTCGGGTGCCAAGG -o<br>trimmed_SRR10915820.fastq.gz SRR10915820.fastq.gz<br># Repeat the command for each file, changing the input and output file names<br>```<br><br>Step 3: Mapping to the reference genome<br>- Index the reference genome with Bowtie.<br>```bash<br># Index the reference genome<br>bowtie-build hg38.fa hg38_index<br>```<br><br>- Map preprocessed reads to the indexed reference genome.<br>```bash<br># Map reads to the indexed reference genome<br>bowtie -v 1 hg38_index -q trimmed_SRR10915820.fastq.gz<br>SRR10915820_hits.sam<br># Repeat the command for each file, changing the input and output file names<br>```<br><br>Step 4: MicroRNA quantification<br>- Use software like mirdeep2 or miRge for microRNA quantification and prediction.<br>```bash<br># Install mirdeep2<br>wget https://www.mdc-berlin.de/8551903/en/releasemirdeep2tgz<br>tar -zxvf miRDeep2v0.0.8.tar.gz<br>cd miRDeep2/<br>chmod +x install.pl<br>./install.pl<br><br># Quantify microRNAs<br>miRDeep2.pl trimmed_SRR10915820.fastq.gz hg38.fa<br>SRR10915820_hits.sam hsa.gff3 none none 2>log.txt >output.txt<br># Repeat the command for each file, changing the input and output file names<br>```<br><br>Step 5: Analysis and prediction of novel microRNAs<br>- Interpret the output to quantify known microRNAs and predict novel microRNAs based on expression and genomic coordinates.<br><br>Remember to adapt the above code and steps further according to the specific requirements and the data you are working with.

CAGE-seq Analysis Case 12.1: TSS analysis

<table>
<tr>
<td>User’s Inputs</td>
<td>You are a bioinformatician who specializes in writing code and performing analysis for a variety of bioinformatic data analysis tasks. Now I provide you with an Ubuntu 18.04 system where I save the following data, each line including the data path and data description.

SRR11351697.fastq.gz: CAGE-seq data from human cell line, control samples replicate 1
SRR11351698.fastq.gz: CAGE-seq data from human cell line, control samples replicate 2
SRR11351700.fastq.gz: CAGE-seq data from human cell line, treatment samples replicate 1
SRR11351701.fastq.gz: CAGE-seq data from human cell line, treatment samples replicate 1
hg38.fa: the human reference genome
gencode.v37.annotation.gtf: gene annotation
TruSeq3-SE.fa: trimming adapter

I need you to help me plan what needs to be done in each step of the analysis process, and help me write the code for each step to install softwares, use tools and accomplish the following main goal:

Identify transcription start site (TSS) in the cell line based on CAGE-seq data</td>
</tr>
<tr>
<td>Generated Results by ChatGPT-3.5</td>
<td>To identify transcription start sites (TSS) based on CAGE-seq data, several steps are required. Below is a plan for the analysis process and the corresponding code for each step.

Step 1: Data preprocessing
- Install necessary software and tools such as FastQC and Cutadapt for quality control and adapter trimming.
```bash
# Install FastQC
sudo apt install fastqc

# Install Cutadapt
sudo apt install cutadapt
```

Step 2: Preprocessing CAGE-seq data
- Perform quality control using FastQC on all FASTQ files.
```bash
Run FastQC on all files
fastqc SRR11351697.fastq.gz SRR11351698.fastq.gz SRR11351700.fastq.gz SRR11351701.fastq.gz
```

- Trim adapters and filter low-quality bases using Cutadapt.
```bash
Trim adapters and filter low-quality bases</td>
</tr>
</table>

cutadapt -a TruSeq3-SE.fa -o trimmed_SRR11351697.fastq.gz SRR11351697.fastq.gz
Repeat the command for each file, changing the input and output file names
```

Step 3: Mapping to the reference genome
- Use a tool like STAR or HISAT2 to align trimmed reads to the human reference genome.
```bash
# Install STAR
sudo apt install star

# Index the reference genome with STAR
STAR --runThreadN 4 --runMode genomeGenerate --genomeDir genome_index --genomeFastaFiles hg38.fa --sjdbGTFfile gencode.v37.annotation.gtf

# Map preprocessed reads to the indexed reference genome
STAR --runThreadN 4 --genomeDir genome_index --readFilesIn trimmed_SRR11351697.fastq.gz --outFileNamePrefix SRR11351697_
# Repeat the command for each file, changing the input and output file names
```

Step 4: Identifying TSS using CAGE-seq data
- Use tools like Paraclu or FANTOM for identifying TSS based on the aligned CAGE-seq data.
```bash
# Install FANTOM
# Download and install FANTOM5 CAGEfightR pipeline from https://github.com/thecodinglizard/CAGEfightR

# Identify TSS using CAGE-seq data
Rscript CAGEfightR_pipeline.R -i SRR11351697_Aligned.sortedByCoord.out.bam -g gencode.v37.annotation.gtf -o TSS_identification_output
# Repeat the command for each file, changing the input and output file names
```

Make sure to adapt the above code and steps further according to the specific requirements and the data you are working with.
```

3’end-seq Analysis   Case 13.1: APA analysis based on 3’end-seq data

**User’s Inputs**	You are a bioinformatician who specializes in writing code and performing analysis for a variety of bioinformatic data analysis tasks. Now I provide you with an Ubuntu 18.04 system where I save the following data, each line including the data path and data description.<br><br>SRR17422754.fastq.gz: Lexongen QuantSeq 3 prime-end RNA sequencing data from human cell line THLE2, healthy liver cell line replicate 1<br>SRR17422755.fastq.gz: Lexongen QuantSeq 3 prime-end RNA sequencing data from human cell line THLE2, healthy liver cell line replicate 2<br>SRR17422756.fastq.gz: Lexongen QuantSeq 3 prime-end RNA sequencing data from human cell line SNU398, liver cancer cell line replicate 1<br>SRR17422757.fastq.gz: Lexongen QuantSeq 3 prime-end RNA sequencing data from human cell line SNU398, liver cancer cell line replicate 2<br>hg38.fa: the human reference genome<br>gencode.v37.annotation.gtf: gene annotation<br>TruSeq3-SE.fa: trimming adapter<br><br>I need you to help me plan what needs to be done in each step of the analysis process, and help me write the code for each step to install softwares, use tools and accomplish the following main goal:<br><br>Identify polyadenylation sites and quantify alternative polyadenylation based on 3'-end sequencing data
**Generated Results by ChatGPT-3.5**	To identify polyadenylation sites and quantify alternative polyadenylation based on 3'-end sequencing data, you would need to perform a series of analysis steps. Below is a plan for the analysis process and the corresponding code for each step.<br><br>Step 1: Data preprocessing<br>Install necessary software and tools such as FastQC and Cutadapt for quality control and adapter trimming.<br>```bash<br># Install FastQC<br>sudo apt install fastqc<br><br># Install Cutadapt<br>sudo apt install cutadapt<br>```<br><br>Step 2: Preprocessing RNA-seq data<br>Perform quality control using FastQC on all FASTQ files.<br>```bash<br># Run FastQC on all files<br>fastqc SRR17422754.fastq.gz SRR17422755.fastq.gz SRR17422756.fastq.gz SRR17422757.fastq.gz<br>```<br><br>Trim adapters and filter low-quality bases using Cutadapt.<br>```bash<br># Trim adapters and filter low-quality bases<br>cutadapt -a TruSeq3-SE.fa -o trimmed_SRR17422754.fastq.gz SRR17422754.fastq.gz

```
# Repeat the command for each file, changing the input and output file names
```

Step 3: Mapping to the reference genome
Use a tool like STAR or HISAT2 to align trimmed reads to the human reference genome.

```bash
# Install STAR
sudo apt install star

# Index the reference genome with STAR
STAR --runThreadN 4 --runMode genomeGenerate --genomeDir genome_index --genomeFastaFiles hg38.fa --sjdbGTFfile gencode.v37.annotation.gtf

# Map preprocessed reads to the indexed reference genome
STAR --runThreadN 4 --genomeDir genome_index --readFilesIn trimmed_SRR17422754.fastq.gz --outFileNamePrefix SRR17422754_
# Repeat the command for each file, changing the input and output file names
```

Step 4: Identifying polyadenylation sites and quantifying alternative polyadenylation
Use tools like DaPars, QAPA, or APAtrap to identify polyadenylation sites and quantify alternative polyadenylation based on the aligned 3'-end sequencing data.

```bash
# Install DaPars, QAPA, or APAtrap according to the software of your choice

# Run the selected tool to identify polyadenylation sites and quantify alternative polyadenylation
# Replace "tool_name" with the actual tool command and parameters
tool_name -i SRR17422754_Aligned.sortedByCoord.out.bam -g gencode.v37.annotation.gtf -o polyadenylation_output
# Repeat the command for each file, changing the input and output file names
```

Always adapt the code and steps based on the specific analysis requirements and the data being used.

ONT sequencing Analysis Case 14.1: isoform expression

<table>
<tr><td>User's Inputs</td><td>You are a bioinformatician who specializes in writing code and performing analysis for a variety of bioinformatic data analysis tasks. Now I provide you with an Ubuntu 18.04 system where I save the following data, each line including the data path and data description.

RHL5569-T1_SAECK_shControl_replicate_1.ont.fastq.gz: ONT sequencing data from human cell line, control samples replicate 1
RHL5570-T1_SAECK_shControl_replicate_2.ont.fastq.gz: ONT sequencing data from human cell line, control samples replicate 2
RHL5571-T1_SAECK_shPARP4-1_replicate_1.ont.fastq.gz: ONT sequencing data from human cell line, treatment samples replicate 1
RHL5572-T1_SAECK_shPARP4-1_replicate_2.ont.fastq.gz: ONT sequencing data from human cell line, treatment samples replicate 2
hg38.fa: the human reference genome
gencode.v37.annotation.gtf: gene annotation

I need you to help me plan what needs to be done in each step of the analysis process, and help me write the code for each step to install softwares, use tools and accomplish the following main goal:

Quantify isoform expression in control and treatment samples</td></tr>
<tr><td>Generated Results by ChatGPT-3.5</td><td>To quantify isoform expression in control and treatment samples using ONT sequencing data, several steps are involved. Below is a plan for the analysis process and the corresponding code for each step.

Step 1: Data preprocessing
Install necessary software and tools for quality control and preprocessing of ONT sequencing data. Commonly used tools include Porechop for adapter trimming and Minimap2 for aligning long reads to a reference genome.
```bash
Install Porechop
pip install Porechop

# Install Minimap2
sudo apt install minimap2
```

Step 2: Preprocessing ONT sequencing data
Perform quality control and adapter trimming using Porechop or similar tools.
```bash
Trim adapters and filter low-quality reads using Porechop
porechop -i RHL5569-T1_SAECK_shControl_replicate_1.ont.fastq.gz -o trimmed_RHL5569-T1_SAECK_shControl_replicate_1.ont.fastq.gz
Repeat the command for each file, changing the input and output file names
```

Step 3: Mapping to the reference genome and isoform quantification</td></tr>
</table>

Use Minimap2 to align the preprocessed ONT reads to the human reference genome and then employ tools like Salmon, Kallisto, or StringTie to quantify isoform expression.

```bash
# Index the reference genome with Minimap2
minimap2 -d hg38.mmi hg38.fa

# Map preprocessed ONT reads to the indexed reference genome using Minimap2
minimap2 -ax map-ont hg38.mmi trimmed_RHL5569-T1_SAECK_shControl_replicate_1.ont.fastq.gz > RHL5569-T1_SAECK_shControl_replicate_1.sam
# Repeat the command for each file, changing the input and output file names

# Quantify isoform expression using Salmon
# Install Salmon
wget https://github.com/COMBINE-lab/salmon/releases/download/v1.4.0/salmon-1.4.0_linux_x86_64.tar.gz
tar -zxvf salmon-1.4.0_linux_x86_64.tar.gz
export PATH=$PATH:/path_to_salmon/salmon-1.4.0_linux_x86_64
salmon index -t gencode.v37.annotation.gtf -i salmon_index
salmon quant -i salmon_index -l A -1 RHL5569-T1_SAECK_shControl_replicate_1.ont.fastq.gz -2 RHL5570-T1_SAECK_shControl_replicate_2.ont.fastq.gz -o control_samples_quant
salmon quant -i salmon_index -l A -1 RHL5571-T1_SAECK_shPARP4-1_replicate_1.ont.fastq.gz -2 RHL5572-T1_SAECK_shPARP4-1_replicate_2.ont.fastq.gz -o treatment_samples_quant
```

Always adapt the code and steps based on the specific analysis requirements and the data being used. Make sure to appropriately account for the specific characteristics of Oxford Nanopore sequencing data in your analysis.

PacBio iso-seq Analysis **Case 15.1: isoform analysis**

<table>
<tr><td>User’s Inputs</td><td>You are a bioinformatician who specializes in writing code and performing analysis for a variety of bioinformatic data analysis tasks. Now I provide you with an Ubuntu 18.04 system where I save the following data, each line including the data path and data description.

SRR19552218.fastq.gz: pacbio sequecning data from human cell line, cell line HCT-116
SRR19785215.fastq.gz: pacbio sequecning data from human cell line, cell line HepG2
hg38.fa: the human reference genome
gencode.v37.annotation.gtf: gene annotation

I need you to help me plan what needs to be done in each step of the analysis process, and help me write the code for each step to install softwares, use tools and accomplish the following main goal:

identify novel isoform and quantify isoform expression in two cell lines</td></tr>
<tr><td>Generated Results by ChatGPT-3.5</td><td>To identify novel isoforms and quantify isoform expression in the two cell lines using PacBio sequencing data, follow the plan and code for each step provided below:

Step 1: Data preprocessing
Install necessary software and tools for quality control and preprocessing of PacBio sequencing data. Consider tools like minimap2 for aligning long reads to a reference genome.

```bash
# Install Minimap2
sudo apt install minimap2
```

Step 2: Preprocessing PacBio sequencing data
Perform quality control on all FASTQ files. Since PacBio data usually doesn't require adapter trimming, focus on quality assessment.

```bash
Perform quality control using FastQC
fastqc SRR19552218.fastq.gz
fastqc SRR19785215.fastq.gz
```

Step 3: Mapping to the reference genome and isoform identification
Align the PacBio reads to the reference genome and identify novel isoforms using tools such as minimap2 for alignment and StringTie or SUPPA for isoform identification.

```bash
Index the human reference genome with minimap2
minimap2 -d hg38.mmi hg38.fa</td></tr>
</table>

```
# Map PacBio reads to the indexed reference genome using minimap2
minimap2 -ax map-pb hg38.mmi SRR19552218.fastq.gz >
SRR19552218.sam
minimap2 -ax map-pb hg38.mmi SRR19785215.fastq.gz >
SRR19785215.sam

# Use StringTie for novel isoform identification
stringtie SRR19552218.sam -G gencode.v37.annotation.gtf -o
SRR19552218_transcripts.gtf
stringtie SRR19785215.sam -G gencode.v37.annotation.gtf -o
SRR19785215_transcripts.gtf
```

Step 4: Quantify isoform expression
Use tools like StringTie, Salmon, or Kallisto to quantify isoform expression.

```bash
# Quantify isoform expression using StringTie
stringtie SRR19552218.sam -G SRR19552218_transcripts.gtf -e -B -A
SRR19552218_abundance.txt
stringtie SRR19785215.sam -G SRR19785215_transcripts.gtf -e -B -A
SRR19785215_abundance.txt
```

Always adapt the code and steps based on the specific analysis requirements and the data being used, especially considering the characteristics of PacBio sequencing data in your analysis.

CLIP-seq Analysis **Case 16.1: CLIP-seq data analysis**

<table>
<tr><td>User's Inputs</td><td>You are a bioinformatician who specializes in writing code and performing analysis for a variety of bioinformatic data analysis tasks. Now I provide you with an Ubuntu 18.04 system where I save the following data, each line including the data path and data description.

ENCLB742AYH.fastq.gz: single end reads of eCLIP data from HepG2 cell line, SRSF1 targeted sample replicate 1
ENCLB770EDJ.fastq.gz: single end reads of eCLIP data from HepG2 cell line, SRSF1 targeted sample replicate 2
hg38.fa: the human reference genome
gencode.v37.annotation.gtf: gene annotation

I need you to help me plan what needs to be done in each step of the analysis process, and help me write the code for each step to install softwares, use tools and accomplish the following main goal:

identify SRSF1 protein-RNA crosslink sites based on CLIP sequencing data</td></tr>
<tr><td>Generated Results by ChatGPT-3.5</td><td>To identify SRSF1 protein-RNA crosslink sites based on eCLIP sequencing data, you will need to perform a series of analyses. Below is a plan for the analysis process and the corresponding code for each step:

Step 1: Data preprocessing
Install necessary software and tools for quality control and preprocessing of eCLIP sequencing data. FastQC is commonly used for quality control.

```bash
# Install FastQC
sudo apt install fastqc
```

Step 2: Preprocessing eCLIP sequencing data
Perform quality control on all FASTQ files.

```bash
Run FastQC on all files
fastqc ENCLB742AYH.fastq.gz
fastqc ENCLB770EDJ.fastq.gz
```

Step 3: Alignment to the reference genome
Use a tool like STAR or HISAT2 to align the eCLIP reads to the human reference genome.

```bash
# Install STAR
sudo apt install star

Index the reference genome with STAR
STAR --runThreadN 4 --runMode genomeGenerate --genomeDir genome_index --genomeFastaFiles hg38.fa --sjdbGTFfile gencode.v37.annotation.gtf</td></tr>
</table>

```
# Map eCLIP reads to the indexed reference genome
STAR --runThreadN 4 --genomeDir genome_index --readFilesIn
ENCLB742AYH.fastq.gz --outFileNamePrefix SRSF1_replicate1_
STAR --runThreadN 4 --genomeDir genome_index --readFilesIn
ENCLB770EDJ.fastq.gz --outFileNamePrefix SRSF1_replicate2_
```

Step 4: Identification of protein-RNA crosslink sites
Finally, use tools like Piranha, CLIPper, or findPeaks (part of Homer suite) to identify protein-RNA crosslink sites based on the aligned eCLIP data.

```bash
# Install the required tool (e.g., Piranha, CLIPper, findPeaks)

# Use the tool to identify protein-RNA crosslink sites
# Replace "tool_command" with the actual command for the selected tool
tool_command -i SRSF1_replicate1.bam -g gencode.v37.annotation.gtf -o
SRSF1_replicate1_crosslink_sites.bed
tool_command -i SRSF1_replicate2.bam -g gencode.v37.annotation.gtf -o
SRSF1_replicate2_crosslink_sites.bed
```

These steps should help you identify SRSF1 protein-RNA crosslink sites based on eCLIP sequencing data. Always adjust the code and steps according to specific analysis needs and the data being used.

RIP-Seq Analysis **Case 16.2: Identify enriched genes bounded by RPB**

<table>
<tr>
<td>User's Inputs</td>
<td>You are a bioinformatician who specializes in writing code and performing analysis for a variety of bioinformatic data analysis tasks. Now I provide you with an Ubuntu 18.04 system where I save the following data, each line including the data path and data description.

rip.rep1.fastq.gz: single end reads of RIP-seq data from HepG2 cell line, pulldown samples of SRSF1 replicate 1
rip.rep2.fastq.gz: single end reads of RIP-seq data from HepG2 cell line, pulldown samples of SRSF1 replicate 2
controll.rep1.fastq.gz: single end reads of RIP-seq daata from HepG2 cell line, control sample replicate 1
controll.rep2.fastq.gz: single end reads of RIP-seq daata from HepG2 cell line, control sample replicate 2
hg38.fa: the human reference genome
gencode.v37.annotation.gtf: gene annotation

I need you to help me plan what needs to be done in each step of the analysis process, and help me write the code for each step to install softwares, use tools and accomplish the following main goal:

identify genes enriched in pulldown samples over the control</td>
</tr>
<tr>
<td>Generated Results by ChatGPT-3.5</td>
<td>To identify genes enriched in pulldown samples over the control in the context of RIP-seq data, you will need to follow a series of analyses. Below is a plan for the analysis process and the corresponding code for each step:

Step 1: Data preprocessing
Install necessary software and tools for quality control and preprocessing of RIP-seq data. FastQC is commonly used for quality control.

```bash
# Install FastQC
sudo apt install fastqc
```

Step 2: Preprocessing RIP-seq data
Perform quality control on all FASTQ files.

```bash
Run FastQC on all files
fastqc rip.rep1.fastq.gz
fastqc rip.rep2.fastq.gz
fastqc control.rep1.fastq.gz
fastqc control.rep2.fastq.gz
```

Step 3: Alignment to the reference genome
Use a tool like STAR or HISAT2 to align the RIP-seq reads to the human reference genome.

```bash</td>
</tr>
</table>

# Install STAR
sudo apt install star

Index the reference genome with STAR
STAR --runThreadN 4 --runMode genomeGenerate --genomeDir genome_index --genomeFastaFiles hg38.fa --sjdbGTFfile gencode.v37.annotation.gtf

Map RIP-seq reads to the indexed reference genome
STAR --runThreadN 4 --genomeDir genome_index --readFilesIn rip.rep1.fastq.gz --outFileNamePrefix SRSF1_pulldown_replicate1_
STAR --runThreadN 4 --genomeDir genome_index --readFilesIn rip.rep2.fastq.gz --outFileNamePrefix SRSF1_pulldown_replicate2_
STAR --runThreadN 4 --genomeDir genome_index --readFilesIn control.rep1.fastq.gz --outFileNamePrefix control_replicate1_
STAR --runThreadN 4 --genomeDir genome_index --readFilesIn control.rep2.fastq.gz --outFileNamePrefix control_replicate2_
```

Step 4: Identifying enriched genes
Use tools like DESeq2, edgeR, or limma to identify differentially enriched genes between pulldown and control samples based on the aligned RIP-seq data.

```bash
# Install the required tool (e.g., DESeq2, edgeR, limma)

# Use the tool to identify differentially enriched genes
# Replace "tool_command" with the actual command for the selected tool
tool_command -i SRSF1_pulldown_replicate1.bam, SRSF1_pulldown_replicate2.bam -c control_replicate1.bam, control_replicate2.bam -g gencode.v37.annotation.gtf -o enriched_genes_output.txt
```

These steps should help you identify genes enriched in pulldown samples over the control based on RIP-seq data. Always adjust the code and steps according to specific analysis needs and the data being used.
```

Ribo-Seq Analysis **Case 17.1: Identify translated ORFs**

<table>
<tr><td>User's Inputs</td><td>You are a bioinformatician who specializes in writing code and performing analysis for a variety of bioinformatic data analysis tasks. Now I provide you with an Ubuntu 18.04 system where I save the following data, each line including the data path and data description.

SRR12354645_1.fastq.gz: read 1 of paired-end ribo-seq data, read 1 of control samples replicate 1
SRR12354645_2.fastq.gz: read 2 of paired-end ribo-seq data, read 2 of control samples replicate 1
SRR12354646_1.fastq.gz: read 1 of paired-end ribo-seq data, read 1 of control samples replicate 2
SRR12354646_2.fastq.gz: read 2 of paired-end ribo-seq data, read 2 of control samples replicate 2
hg38.fa: the human reference genome
gencode.v37.annotation.gtf: gene annotation
TruSeq3-PE.fa: trimming adapter

I need you to help me plan what needs to be done in each step of the analysis process, and help me write the code for each step to install softwares, use tools and accomplish the following main goal:

Identify translated ORFs based on ribo-seq data</td></tr>
<tr><td>Generated Results by ChatGPT-3.5</td><td>To identify translated ORFs based on ribo-seq data, you will need to carry out several steps, from preprocessing the data to analyzing the results. Here's a plan for the analysis process and the corresponding code for each step:

Step 1: Data preprocessing
Install and use software tools for quality control and adapter trimming. FastQC and Cutadapt are typically used for these tasks.

```bash
# Install FastQC
sudo apt install fastqc

# Install Cutadapt
sudo apt install cutadapt
```

Step 2: Preprocessing ribo-seq data
Perform quality control using FastQC, trim adapters, and filter low-quality bases using Cutadapt.

```bash
Run FastQC on all files
fastqc SRR12354645_1.fastq.gz SRR12354645_2.fastq.gz SRR12354646_1.fastq.gz SRR12354646_2.fastq.gz

Trim adapters and filter low-quality bases using Cutadapt</td></tr>
</table>

```
cutadapt -a file:TruSeq3-PE.fa -o trimmed_SRR12354645_1.fastq.gz -p
trimmed_SRR12354645_2.fastq.gz SRR12354645_1.fastq.gz
SRR12354645_2.fastq.gz
cutadapt -a file:TruSeq3-PE.fa -o trimmed_SRR12354646_1.fastq.gz -p
trimmed_SRR12354646_2.fastq.gz SRR12354646_1.fastq.gz
SRR12354646_2.fastq.gz
```

Step 3: Mapping to the reference genome
Use a tool like STAR or HISAT2 to align the trimmed ribo-seq reads to the human reference genome.

```bash
# Install STAR
sudo apt install star

# Index the reference genome with STAR
STAR --runThreadN 4 --runMode genomeGenerate --genomeDir
genome_index --genomeFastaFiles hg38.fa --sjdbGTFfile
gencode.v37.annotation.gtf

# Map preprocessed reads to the indexed reference genome
STAR --runThreadN 4 --genomeDir genome_index --readFilesIn
trimmed_SRR12354645_1.fastq.gz trimmed_SRR12354645_2.fastq.gz --
outFileNamePrefix SRR12354645_
STAR --runThreadN 4 --genomeDir genome_index --readFilesIn
trimmed_SRR12354646_1.fastq.gz trimmed_SRR12354646_2.fastq.gz --
outFileNamePrefix SRR12354646_
```

Step 4: Identify translated ORFs
Use tools such as Ribo-seq pipeline, ORF-RATER, or RiboTaper to identify translated ORFs based on the aligned ribo-seq data.

```bash
# Use the selected tool to identify translated ORFs
# Replace "tool_command" with the actual command for the chosen tool
tool_command -i SRR12354645_Aligned.sortedByCoord.out.bam -g
gencode.v37.annotation.gtf -o translated_ORFs_rep1.bed
tool_command -i SRR12354646_Aligned.sortedByCoord.out.bam -g
gencode.v37.annotation.gtf -o translated_ORFs_rep2.bed
```

Always adapt the code and steps based on the specific analysis requirements and the data being used. Ensure that the software and tools chosen align with the characteristics of ribo-seq data.

scRNA-Seq Analysis Case 18.1: Cluster cell into groups based on raw fastq files

**User's Inputs**	You are a bioinformatician who specializes in writing code and performing analysis for a variety of bioinformatic data analysis tasks. Now I provide you with an Ubuntu 18.04 system where I save the following data, each line including the data path and data description.<br><br>/pbmc_1k_v3_fastqs.tar: the packed fastq files from 10x single cell sequencing<br>refdata-gex-GRCh38-2020-A: directory contains transcriptome reference<br><br>I need you to help me plan what needs to be done in each step of the analysis process, and help me write the code for each step to install softwares, use tools and accomplish the following main goal:<br><br>cluster cells into different groups based on 10x single cell sequencing data
**Generated Results by ChatGPT-3.5**	To cluster cells based on 10x single-cell sequencing data, you will need to carry out several steps, from preprocessing the data to running cell clustering algorithms. Here's a high-level overview of the process and the corresponding code for each step:<br><br>Step 1: Data extraction<br>Extract the 10x single-cell sequencing fastq files from the provided tar archive.<br><br>```bash<br># Extract the contents of the tar file<br>tar -xvf pbmc_1k_v3_fastqs.tar<br>```<br><br>Step 2: Preprocessing and alignment<br>Install and use software tools for quality control, preprocessing, and alignment of the single-cell sequencing data. Commonly used tools include Cell Ranger for preprocessing and alignment.<br><br>```bash<br># Install Cell Ranger<br># Download Cell Ranger from the 10x Genomics website and follow installation instructions<br># For example:<br>wget "https://cf.10xgenomics.com/releases/cell-exp/cellranger-6.0.0.tar.gz"<br>tar -zxvf cellranger-6.0.0.tar.gz<br>export PATH=$PATH:/path_to_cellranger/cellranger-6.0.0<br>```<br><br>Step 3: Preprocessing and alignment<br>Use Cell Ranger to preprocess and align the single-cell sequencing data to the transcriptome reference.<br><br>```bash<br># Run Cell Ranger to preprocess and align the data<br>cellranger count --id=pbmc_1k --transcriptome=refdata-gex-GRCh38-2020-A --fastqs=pbmc_1k_v3_fastqs

````
```
````

Step 4: Cell clustering
Use Seurat, Scanpy, or another tool to cluster cells based on the aligned single-cell sequencing data.

```R
# Assuming you have R and Seurat installed
# Install Seurat if you haven't already
install.packages("Seurat")

# Run cell clustering with Seurat
library(Seurat)
pbmc_1k <- Read10X(data.dir = "pbmc_1k/outs/filtered_feature_bc_matrix")
# Read in the count matrix
pbmc_1k <- CreateSeuratObject(counts = pbmc_1k, project = "pbmc_1k",
min.cells = 3, min.features = 200)
pbmc_1k <- NormalizeData(pbmc_1k)
pbmc_1k <- FindVariableFeatures(pbmc_1k, selection.method = "vst",
nfeatures = 2000)
pbmc_1k <- ScaleData(pbmc_1k, features = rownames(pbmc_1k))
pbmc_1k <- RunPCA(pbmc_1k, features = VariableFeatures(object =
pbmc_1k))
pbmc_1k <- FindNeighbors(pbmc_1k, dims = 1:10)
pbmc_1k <- FindClusters(pbmc_1k, resolution = 0.6)
```

In the above code, the assumption is that the data is RNA-seq-based single-cell data. Adjust the tools and code based on the specific type of single-cell sequencing data you are working with, given that different types may require different preprocessing and clustering approaches. Always ensure that software and tools are compatible with your analysis needs and the data provided.
```

scRNA-Seq Analysis Case 18.2: Clustering and Find the differentially expressed genes

<table>
<tr><td>User's Inputs</td><td>You are a bioinformatician who specializes in writing code and performing analysis for a variety of bioinformatic data analysis tasks. Now I provide you with an Ubuntu 18.04 system where I save the following data, each line including the data path and data description.

./examples/case2.1/data/filtered_gene_bc_matrices/hg19: path to 10x mtx data

I need you to help me plan what needs to be done in each step of the analysis process, and help me write the code for each step to install softwares, use tools and accomplish the following main goal:

Use scanpy to find the differentially expressed genes</td></tr>
<tr><td>Generated Results by ChatGPT-3.5</td><td>To use Scanpy to find differentially expressed genes, you will need to follow a series of steps, from setting up the required software to running the analysis. Below is a plan for the analysis process and the corresponding code for each step:

Step 1: Environment Setup
Set up a Python environment with the required packages. You can use pip or conda to install Scanpy and its dependencies.

```bash
Create and activate a virtual environment (optional but recommended)
This step assumes you have Miniconda or Anaconda installed
conda create -n scanpy_env python=3.8
conda activate scanpy_env

Install Scanpy and its dependencies
conda install -c conda-forge scanpy
```

Step 2: Load and preprocess the single-cell data
Load the single-cell data from the provided 10x mtx directory and perform basic preprocessing steps such as quality control, normalization, and gene filtering.

```python
import scanpy as sc

Load the single-cell data from the provided directory
adata =
sc.read_10x_mtx('./examples/case2.1/data/filtered_gene_bc_matrices/hg19
', var_names='gene_symbols', cache=True)

Preprocess the data (quality control, normalization, filtering)
sc.pp.filter_genes(adata, min_cells=3) # Filter genes expressed in less than 3 cells
sc.pp.normalize_total(adata) # Normalize counts per cell
sc.pp.log1p(adata) # Logarithmize the data
sc.pp.scale(adata) # Scale (z-score) the data</td></tr>
</table>

	```<br><br>Step 3: Find differentially expressed genes<br>Use Scanpy to find differentially expressed genes between experimental conditions or cell clusters.<br><br>```python<br># Perform differential expression analysis<br>sc.tl.rank_genes_groups(adata, 'group_variable') # Replace 'group_variable' with the variable that identifies the groups in your data<br>result = adata.uns['rank_genes_groups']<br><br># Visualize the results (volcano plot, etc.)<br>sc.pl.rank_genes_groups(adata, group='group_variable', n_genes=25) # Visualize the top 25 DE genes<br>```<br><br>Always adapt the code and steps based on the specific analysis requirements and the data being used. Ensure that the software and tools chosen align with the characteristics of your single-cell data.

scRNA-Seq Analysis Case 18.3: Identify Marker Genes

<table>
<tr><td>User's Inputs</td><td>You are a bioinformatician who specializes in writing code and performing analysis for a variety of bioinformatic data analysis tasks. Now I provide you with an Ubuntu 18.04 system where I save the following data, each line including the data path and data description.

./examples/case2.1/data/filtered_gene_bc_matrices/hg19: path to 10x mtx data

I need you to help me plan what needs to be done in each step of the analysis process, and help me write the code for each step to install softwares, use tools and accomplish the following main goal:

Use scanpy to identify top5 marker genes.</td></tr>
<tr><td>Generated Results by ChatGPT-3.5</td><td>To use Scanpy to identify the top 5 marker genes from your 10x mtx data, you will need to set up the environment, load and preprocess the data, then perform cluster identification and marker gene identification. Below is the plan for the analysis process and the corresponding code for each step:

Step 1: Environment Setup
Set up a Python environment with the required packages. You can use pip or conda to install Scanpy and its dependencies.

```bash
Create and activate a virtual environment (optional but recommended)
This step assumes you have Miniconda or Anaconda installed
conda create -n scanpy_env python=3.8
conda activate scanpy_env

Install Scanpy and its dependencies
conda install -c conda-forge scanpy
```

Step 2: Load and preprocess the single-cell data
Load the single-cell data from the provided 10x mtx directory and perform basic preprocessing steps such as quality control, normalization, and gene filtering.

```python
import scanpy as sc

Load the single-cell data from the provided directory
adata =
sc.read_10x_mtx('./examples/case2.1/data/filtered_gene_bc_matrices/hg19
', var_names='gene_symbols', cache=True)

Preprocess the data (quality control, normalization, filtering)
sc.pp.filter_genes(adata, min_cells=3) # Filter genes expressed in less than 3 cells
sc.pp.normalize_total(adata) # Normalize counts per cell
sc.pp.log1p(adata) # Logarithmize the data
sc.pp.scale(adata) # Scale (z-score) the data</td></tr>
</table>

<table>
<tr>
<td></td>
<td>```

Step 3: Identify top 5 marker genes
Use Scanpy to identify the top 5 marker genes that distinguish different clusters or groups of cells.

```python
# Perform clustering to identify cell clusters (if not already done)
sc.tl.leiden(adata)

# Identify marker genes using the Wilcoxon rank-sum test
sc.tl.rank_genes_groups(adata, groupby='leiden', method='wilcoxon')

# Visualize the top 5 marker genes per cluster or group
sc.pl.rank_genes_groups(adata, n_genes=5)
```

Always adapt the code and steps based on the specific analysis requirements and the data being used. Ensure that the software and tools chosen align with the characteristics of your single-cell data.</td>
</tr>
</table>

scRNA-Seq Analysis Case 18.4: Clustering and Visualization

<table>
<tr><td>User's Inputs</td><td>You are a bioinformatician who specializes in writing code and performing analysis for a variety of bioinformatic data analysis tasks. Now I provide you with an Ubuntu 18.04 system where I save the following data, each line including the data path and data description.

./examples/case2.1/data/filtered_gene_bc_matrices/hg19: path to 10x mtx data

I need you to help me plan what needs to be done in each step of the analysis process, and help me write the code for each step to install softwares, use tools and accomplish the following main goal:

Use scanpy to perform clustering and visualize the expression level of gene PPBP in the UMAP.</td></tr>
<tr><td>Generated Results by ChatGPT-3.5</td><td>To use Scanpy to perform clustering and visualize the expression level of gene PPBP in the UMAP, you will need to set up the environment, load and preprocess the data, perform clustering, and visualize gene expression. Below is the plan for the analysis process and the corresponding code for each step:

Step 1: Environment Setup
Set up a Python environment with the required packages. You can use pip or conda to install Scanpy and its dependencies.

```bash
Create and activate a virtual environment (optional but recommended)
This step assumes you have Miniconda or Anaconda installed
conda create -n scanpy_env python=3.8
conda activate scanpy_env

Install Scanpy and its dependencies
conda install -c conda-forge scanpy
```

Step 2: Load and preprocess the single-cell data
Load the single-cell data from the provided 10x mtx directory and perform basic preprocessing steps such as quality control, normalization, and gene filtering.

```python
import scanpy as sc

Load the single-cell data from the provided directory
adata =
sc.read_10x_mtx('./examples/case2.1/data/filtered_gene_bc_matrices/hg19
', var_names='gene_symbols', cache=True)

Preprocess the data (quality control, normalization, filtering)
sc.pp.filter_genes(adata, min_cells=3) # Filter genes expressed in less than 3 cells
sc.pp.normalize_total(adata) # Normalize counts per cell</td></tr>
</table>

	sc.pp.log1p(adata) # Logarithmize the data<br>sc.pp.scale(adata) # Scale (z-score) the data<br>```<br><br>Step 3: Perform clustering using UMAP<br>Use Scanpy to perform clustering and visualize the gene expression level of PPBP in UMAP.<br><br>```python<br># Perform clustering and UMAP visualization<br>sc.pp.neighbors(adata, n_neighbors=10, n_pcs=30)<br>sc.tl.umap(adata)<br>sc.tl.leiden(adata) # Perform clustering<br><br># Visualize the expression level of gene PPBP in the UMAP<br>sc.pl.umap(adata, color=['PPBP'], use_raw=False) # Visualize PPBP gene expression in the UMAP<br>```<br><br>Always adapt the code and steps based on the specific analysis requirements and the data being used. Ensure that the software and tools chosen align with the characteristics of your single-cell data.

Spatial Transcriptomics **Case 19.1: Neighborhood enrichment analysis**

User's Inputs

You are a bioinformatician who specializes in writing code and performing analysis for a variety of bioinformatic data analysis tasks. Now I provide you with an Ubuntu 18.04 system where I save the following data, each line including the data path and data description.

slice1.h5ad: spatial transcriptomics data for slice 1 in AnnData format

I need you to help me plan what needs to be done in each step of the analysis process, and help me write the code for each step to install softwares, use tools and accomplish the following main goal:

Use squidpy for neighborhood enrichment analysis

Generated Results by ChatGPT-3.5

To use Squidpy for neighborhood enrichment analysis, you will need to complete several steps, including environment setup, loading and preprocessing the spatial transcriptomics data, and performing the neighborhood enrichment analysis. Below is the plan for the analysis process and the corresponding code for each step:

Step 1: Environment Setup
Set up a Python environment with the required packages, including Squidpy and dependencies. You can use pip or conda to install Squidpy and its dependencies.

```bash
# Create and activate a virtual environment (optional but recommended)
# This step assumes you have Miniconda or Anaconda installed
conda create -n squidpy_env python=3.8
conda activate squidpy_env

# Install Squidpy and its dependencies
conda install -c conda-forge squidpy
```

Step 2: Load and preprocess the spatial transcriptomics data
Load the spatial transcriptomics data from the provided H5AD file, adjust preprocessing steps based on the specific data, and explore its basic characteristics.

```python
import scanpy as sc
import squidpy as sq

# Load the spatial transcriptomics data from the provided file
adata = sc.read_h5ad('slice1.h5ad')

# Preprocess the data as needed (quality control, normalization, filtering,
etc.)
# e.g., running basic preprocessing steps for quality control, normalization,
and filtering
sc.pp.filter_genes(adata, min_counts=1)  # Example gene filtering based on
counts
```

sc.pp.normalize_total(adata) # Normalize counts per cell
sc.pp.log1p(adata) # Logarithmize the data
sc.pp.highly_variable_genes(adata, flavor='seurat', n_top_genes=2000) # Identify highly variable genes
sc.pp.scale(adata, max_value=10) # Scale (z-score) the data

# Explore the data to understand its basic characteristics
print(adata)
```

Step 3: Perform neighborhood enrichment analysis using Squidpy
Use Squidpy to perform neighborhood enrichment analysis on the spatial transcriptomics data.

```python
# Perform neighborhood enrichment analysis
# This step assumes you have spatial coordinates information in adata.obsm['spatial'] and have defined neighborhoods
sq.gr.nhood_enrichment(adata, cluster_key='leiden', mode='dot_product', n_perms=1000)

# Visualize the neighborhood enrichment results
sq.pl.nhood_enrichment(adata, cluster_key='leiden')
```

Always adjust the code and steps based on the specific analysis requirements and the data being used. Ensure that the software and tools chosen align with the characteristics of your spatial transcriptomics data.
```

Spatial Transcriptomics **Case 19.2: Single-cell mapping**

<table>
<tr><td>User's Inputs</td><td>You are a bioinformatician who specializes in writing code and performing analysis for a variety of bioinformatic data analysis tasks. Now I provide you with an Ubuntu 18.04 system where I save the following data, each line including the data path and data description.

slice1.h5ad: spatial transcriptomics data for slice 1 in AnnData format
sc.h5ad: single cell data in the same tissue for slice 1 in AnnData format

I need you to help me plan what needs to be done in each step of the analysis process, and help me write the code for each step to install softwares, use tools and accomplish the following main goal:

Use Tangram to map single-cell data to spatial transcriptomics data.</td></tr>
<tr><td>Generated Results by ChatGPT-3.5</td><td>To use Tangram to map single-cell data to spatial transcriptomics data, you will need to set up an environment, load and preprocess the spatial transcriptomics and single-cell data, and then use Tangram for spatial mapping. Below is the plan for the analysis process and the corresponding code for each step:

Step 1: Environment Setup
Set up a Python environment with the required packages, including Tangram and its dependencies. You can use pip or conda to install Tangram and its dependencies.

```bash
Create and activate a virtual environment (optional but recommended)
This step assumes you have Miniconda or Anaconda installed
conda create -n tangram_env python=3.8
conda activate tangram_env

Install Tangram and its dependencies
Please check the specific installation instructions for Tangram
For example (not real commands, please consult the Tangram documentation):
pip install tangram
```

Step 2: Load and preprocess the spatial transcriptomics and single-cell data
Load the spatial transcriptomics data and single-cell data from the provided H5AD files, and preprocess the data as needed (quality control, normalization, filtering, etc.).

```python
import scanpy as sc
import anndata
import tangram as tg

# Load the spatial transcriptomics data from the provided file
spatial_data = anndata.read_h5ad('slice1.h5ad')

Load the single-cell data from the provided file</td></tr>
</table>

single_cell_data = anndata.read_h5ad('sc.h5ad')

# Preprocess the data as needed for both datasets
# Example: Preprocess both spatial and single cell data, which may include quality control, normalization, and filtering
# For instance:
sc.pp.normalize_total(single_cell_data, target_sum=1e4)
sc.pp.log1p(single_cell_data)

sc.pp.normalize_total(spatial_data, target_sum=1e4)
sc.pp.log1p(spatial_data)
```

Step 3: Use Tangram for spatial mapping
Utilize Tangram to map the single-cell data to the spatial transcriptomics data.

```python
# Map single-cell data to spatial transcriptomics data using Tangram
tangram_model = tg.tangram(spatial_data, single_cell_data)

# Add spatial information to single-cell data
mapped_single_cell_data = tg.predict(tangram_model, single_cell_data)

# You can then use the mapped_single_cell_data for downstream analysis or visualization
```

Always adapt the code and steps based on the specific analysis requirements and the data being used. Ensure that the software and tools chosen align with your specific data types and experimental design.
```

Proteomics Case 20.1: protein expression quantification

<table>
<tr><td>User’s Inputs</td><td>You are a bioinformatician who specializes in writing code and performing analysis for a variety of bioinformatic data analysis tasks. Now I provide you with an Ubuntu 18.04 system where I save the following data, each line including the data path and data description.

01CPTAC_COprospective_W_PNNL_20170123_B1S1_f01.mzML.gz: protein mass spectrometry data in mzML format, tumor samples fraction 1
01CPTAC_COprospective_W_PNNL_20170123_B1S1_f02.mzML.gz: protein mass spectrometry data in mzML format, tumor samples fraction 2
01CPTAC_COprospective_W_PNNL_20170123_B1S1_f03.mzML.gz: protein mass spectrometry data in mzML format, tumor samples fraction 3
98CPTAC_COprospective_W_VU_20160804_11CO047_f01.mzML.gz: protein mass spectrometry data in mzML format, normal samples fraction 1
98CPTAC_COprospective_W_VU_20160804_11CO047_f02.mzML.gz: protein mass spectrometry data in mzML format, normal samples fraction 2
98CPTAC_COprospective_W_VU_20160804_11CO047_f03.mzML.gz: protein mass spectrometry data in mzML format, normal samples fraction 3
UP000005640_9606.fasta: reference protein sequence from UniProt

I need you to help me plan what needs to be done in each step of the analysis process, and help me write the code for each step to install softwares, use tools and accomplish the following main goal:

identify proteins in tumor and normal samples based on mass spectrometry data</td></tr>
<tr><td>Generated Results by ChatGPT-3.5</td><td>To identify proteins in tumor and normal samples based on mass spectrometry data, you'll need to perform several steps which include setting up the environment, preprocessing, data processing, and finally protein identification. Below are the plan and corresponding code for each step:

Step 1: Environment Setup
Set up a Python environment with the required packages, including Proteomics tools such as MaxQuant, Proteome Discoverer, or other similar tools that could help you process the mass spectrometry data. Additionally, you might need to install the specialized proteomics search engines such as Mascot, Sequest, or Comet to identify proteins from the mass spectrometry data.

Proteomics software such as MaxQuant and Proteome Discoverer are often proprietary tools that are not directly available through package managers. However, some open-source proteomics analysis tools like OpenMS or Philosopher can be installed through different methods such as conda or pip.

For this example, let's assume you are using the OpenMS proteomics software suite, which is available through conda.

```bash
Create and activate a virtual environment (optional but recommended)</td></tr>
</table>

This step assumes you have Miniconda or Anaconda installed
conda create -n proteomics_env python=3.8
conda activate proteomics_env

Install OpenMS using conda
conda install -c conda-forge -c bioconda openms
```

Step 2: Data Preprocessing
Preprocess the mzML files to convert them into a format suitable for downstream analysis, for example into mzXML or mgf format. This step might also involve peak picking, noise filtering, and the conversion into a format suitable for protein identification.

```bash
# Convert mzML files to mzXML using OpenMS
FileConverter -in
01CPTAC_COprospective_W_PNNL_20170123_B1S1_f01.mzML.gz -out
01CPTAC_COprospective_W_PNNL_20170123_B1S1_f01.mzXML
# Repeat the above command for other mzML files

# Convert mzML files to mgf using OpenMS (alternative format for
downstream analysis)
FileConverter -in
01CPTAC_COprospective_W_PNNL_20170123_B1S1_f01.mzML.gz -out
01CPTAC_COprospective_W_PNNL_20170123_B1S1_f01.mgf
# Repeat the above command for other mzML files
```

Step 3: Protein Identification
Use a proteomics search engine like Mascot, Sequest, or Comet to identify proteins from the mass spectrometry data. You will use the reference protein sequence file in UniProt (UP000005640_9606.fasta) as the database for the search.

The following command uses Mascot as an example to perform protein identification based on the mzXML files:

```bash
mascot_cmd -s
01CPTAC_COprospective_W_PNNL_20170123_B1S1_f01.mzXML -d
UP000005640_9606.fasta -o identified_proteins_tumor.csv
```

Repeat the above procedure for each of the mzXML files representing tumor samples, as well as the mzXML files representing normal samples.

Always adapt the code and steps based on the specific tools you are using for your mass spectrometry data analysis. Ensure that the software and tools chosen align with your data characteristics and experimental design. This process may vary based on the specific software used and the format of the mass spectrometry data you are working with.
```

Metabolomics Case 21.1: metabolites concentration

<table>
<tr><td>User's Inputs</td><td>You are a bioinformatician who specializes in writing code and performing analysis for a variety of bioinformatic data analysis tasks. Now I provide you with an Ubuntu 18.04 system where I save the following data, each line including the data path and data description.

B1S1_f01.mzML.gz: LC-MS mass spectrometry data of metabolites in mzML format, tumor samples fraction 1
B1S1_f02.mzML.gz: LC-MS mass spectrometry data of metabolites in mzML format, tumor samples fraction 2
B1S1_f03.mzML.gz: LC-MS mass spectrometry data of metabolites in mzML format, tumor samples fraction 3
11CO047_f01.mzML.gz: LC-MS mass spectrometry data of metabolites in mzML format, normal samples fraction 1
11CO047_f02.mzML.gz: LC-MS mass spectrometry data of metabolites in mzML format, normal samples fraction 2
11CO047_f03.mzML.gz: LC-MS mass spectrometry data of metabolites in mzML format, normal samples fraction 3

I need you to help me plan what needs to be done in each step of the analysis process, and help me write the code for each step to install softwares, use tools and accomplish the following main goal:

calculate metabolites concentration in tumor and normal samples based on LC-MS mass spectrometry data</td></tr>
<tr><td>Generated Results by ChatGPT-3.5</td><td>To calculate metabolite concentrations in tumor and normal samples based on LC-MS mass spectrometry data, you'll need to perform several steps, including environment setup, data preprocessing, feature extraction, and concentration calculation. Below is the plan for the analysis and the corresponding code for each step:

Step 1: Environment Setup
Set up a Python environment with the required packages for LC-MS data processing and metabolomics analysis. You may want to install software such as XCMS, MZmine, or other tools suitable for LC-MS data processing.

For this example, let's use XCMS as a representative tool. XCMS is a widely used package for preprocessing and feature extraction from mass spectrometry data and it can be installed using R.

```bash
# Install R
sudo apt update
sudo apt install r-base

Start R console
R</td></tr>
</table>

```
# Install XCMS package within R
if (!requireNamespace("BiocManager", quietly = TRUE))
    install.packages("BiocManager")
BiocManager::install("xcms")
```

Step 2: Data Preprocessing
Preprocess the mzML files to extract and align features such as peaks representing metabolites. This step might include peak picking, retention time correction, noise filtering, and other preprocessing steps.

```R
library(xcms)

# Read and preprocessing mzML files
# Use the following code as a base and adjust parameters as needed
filepaths <- c("B1S1_f01.mzML.gz", "B1S1_f02.mzML.gz",
"B1S1_f03.mzML.gz", "11CO047_f01.mzML.gz", "11CO047_f02.mzML.gz",
"11CO047_f03.mzML.gz")
raw_data <- readMSData2(filepaths, centroided = TRUE)
centWaveSettings <- CentWaveParam(snthresh=5, noise=1000)
features <- findChromPeaks(raw_data, param=centWaveSettings)
aligned_features <- groupChromPeaks(features)
```

Step 3: Feature Extraction and Quantification
Extract features representing metabolites and calculate their concentrations in tumor and normal samples. This step might involve peak integration, feature alignment, and normalization.

```R
# Extract and quantify features in the samples
feature_values <- quantifyFeatures(aligned_features, raw_data)
normalized_values <- retcor(feature_values)
```

These steps provide a basic outline for processing LC-MS data and calculating metabolite concentrations in tumor and normal samples. The code provided serves as a starting point, and specific parameters and methods may need to be tailored based on the characteristics of your data and the specific tools used for metabolite quantification. Always ensure that the software and tools chosen align with the characteristics of your LC-MS data and experimental design.

9. Results of ChatGPT-4

WGS data analysis   Case 1.1: Genome assembly

<table>
<tr>
<td>User's Inputs</td>
<td>You are a bioinformatician who specializes in writing code and performing analysis for a variety of bioinformatic data analysis tasks. Now I provide you with an Ubuntu 18.04 system where I save the following data, each line including the data path and data description.

SRR1234567_R1.fastq.gz: Paired-end Illumina WGS reads, forward
SRR1234567_R2.fastq.gz: Paired-end Illumina WGS reads, reverse
TruSeq3-PE.fa: Adapter sequences for trimming
Reference.fasta: Reference genome for the organism under study in FASTA format

I need you to help me plan what needs to be done in each step of the analysis process, and help me write the code for each step to install softwares, use tools and accomplish the following main goal:

To perform genome assembly using paired-end WGS data</td>
</tr>
<tr>
<td>Generated Results by ChatGPT-4</td>
<td>Sure, here is an example of a basic pipeline for genome assembly using paired-end whole genome sequencing data:

Step 1: Preprocessing of Raw Reads

Firstly, trim adapters and low-quality bases from the reads using **Trimmomatic**. This step removes any adaptor sequences and improves the overall quality of the reads.

Before running trimmomatic, install it:

```bash
sudo apt update
sudo apt install -y default-jre
wget
http://www.usadellab.org/cms/uploads/supplementary/Trimmomatic/Trimmomatic-0.39.zip
unzip Trimmomatic-0.39.zip
```

Then, run trimmomatic:

```bash
java -jar Trimmomatic-0.39/trimmomatic-0.39.jar PE \
    -phred33 \
    SRR1234567_R1.fastq.gz SRR1234567_R2.fastq.gz \
    SRR1234567_R1_trimmed.fastq.gz SRR1234567_R1_unpaired.fastq.gz \
    SRR1234567_R2_trimmed.fastq.gz SRR1234567_R2_unpaired.fastq.gz \
    ILLUMINACLIP:TruSeq3-PE.fa:2:30:10 \</td>
</tr>
</table>

    LEADING:3 TRAILING:3 SLIDINGWINDOW:4:15 MINLEN:36
```

### Step 2: Genome Assembly

There are many tools available for genome assembly: **SPAdes**, **MaSuRCA**, **Velvet**, etc. Here, we'll use **SPAdes**.

First, you need to install it:

```bash
wget http://cab.spbu.ru/files/release3.15.2/SPAdes-3.15.2-Linux.tar.gz
tar -xvzf SPAdes-3.15.2-Linux.tar.gz
export PATH=$PATH:$PWD/SPAdes-3.15.2-Linux/bin
```

Run SPAdes for the genome assembly:

```bash
spades.py --pe1-1 SRR1234567_R1_trimmed.fastq.gz \
    --pe1-2 SRR1234567_R2_trimmed.fastq.gz \
    --cov-cutoff auto -t 8 -m 16 -o spades_output
```

Step 3: Validate Assembly Results

After assembly, you can validate your results using a tool like **QUAST**.

Install it:

```bash
wget https://downloads.sourceforge.net/project/quast/quast-5.0.2.tar.gz
tar -xzf quast-5.0.2.tar.gz
cd quast-5.0.2
sudo ./setup.py install
cd ..
```

Then run QUAST:

```bash
quast.py spades_output/contigs.fasta -o quast_report
```

This pipeline is a simplification and there might be more steps involved, including error correction, scaffolding, or gap closing. Sometimes, additional validation steps, like checking for contamination, might be needed. This pipeline also assumes that the input data is of sufficient coverage and quality to produce a high-quality genome assembly.
```

WGS/WES data analysis **Case 2.1 and Case 2.2 : Somatic SNV+indel calling and annotation**

<table>
<tr><td>User's Inputs</td><td>You are a bioinformatician who specializes in writing code and performing analysis for a variety of bioinformatic data analysis tasks. Now I provide you with an Ubuntu 18.04 system where I save the following data, each line including the data path and data description.

SRR23015874_R1.fastq.gz: paired end read1 from human whole genome sequencing, normal samples replicate 1
SRR23015874_R2.fastq.gz: paired end read2 from human whole genome sequencing, normal samples replicate 1
SRR23015876_R1.fastq.gz: paired end read1 from human whole genome sequencing, tumor samples replicate 1
SRR23015876_R2.fastq.gz: paired end read2 from human whole genome sequencing, tumor samples replicate 1
SRR23015877_R1.fastq.gz: paired end read1 from human whole genome sequencing, tumor samples replicate 2
SRR23015877_R2.fastq.gz: paired end read2 from human whole genome sequencing, tumor samples replicate 2
TruSeq3-PE.fa: trimming adapter
hg38.fa: human hg38 reference genome fasta

I need you to help me plan what needs to be done in each step of the analysis process, and help me write the code for each step to install softwares, use tools and accomplish the following main goal:

call somatic mutations in tumor samples over the matched normal samples and annotate the identified mutations</td></tr>
<tr><td>Generated Results by ChatGPT-4</td><td>Calling somatic mutations in tumor samples against matched normal samples is a common task in cancer genomics. The specific steps can vary, but a general pipeline could be:

1. Adapter and quality trimming
2. Alignment of the reads to the reference genome
3. Sort and index the BAM files
4. Marking duplicates
5. Realignment around indels
6. Base recalibration
7. Variant calling
8. Variant annotation

Step 1: Adapter and Quality Trimming

We'll use **Trimmomatic** for this. The process was previously described, and the command line will look the same for each pair of the sequencing reads. Let's assume `Trimmomatic-0.39` directory is in the your PATH.

```bash
java -jar Trimmomatic-0.39/trimmomatic-0.39.jar PE \
    -phred33 \
    SRR23015874_R1.fastq.gz SRR23015874_R2.fastq.gz \
    SRR23015874_R1_trimmed.fastq.gz \
    SRR23015874_R1_unpaired.fastq.gz \</td></tr>
</table>

    SRR23015874_R2_trimmed.fastq.gz \
    SRR23015874_R2_unpaired.fastq.gz \
    ILLUMINACLIP:TruSeq3-PE.fa:2:30:10 \
    LEADING:3 TRAILING:3 SLIDINGWINDOW:4:15 MINLEN:36
```
Repeat this for all sample pairs.

### Step 2: Alignment of the reads to the reference genome

We'll use **BWA-MEM** for this. First install BWA:

```bash
sudo apt-get update
sudo apt-get install bwa
```
Index the reference genome:

```bash
bwa index hg38.fa
```
Then, align each pair of the trimmed reads to the reference genome:

```bash
bwa mem hg38.fa SRR23015874_R1_trimmed.fastq.gz
SRR23015874_R2_trimmed.fastq.gz > aligned_SRR23015874.sam
```
Repeat this for other samples.

Step 3: Sort and Index the BAM files

We'll use **SAMtools** for this. Install it via:

```bash
sudo apt-get install samtools
```
Convert the SAM file to BAM, sort, and index it:

```bash
samtools view -bS aligned_SRR23015874.sam | samtools sort - -o
sorted_SRR23015874.bam
samtools index sorted_SRR23015874.bam
```
Repeat this for other samples.

Step 4: Marking duplicates

We'll use **Picard Tools** for this. Let's assume `picard.jar` is in your PATH.

```bash
java -jar picard.jar MarkDuplicates \
    INPUT=sorted_SRR23015874.bam \
    OUTPUT=marked_duplicates_SRR23015874.bam \

    METRICS_FILE=metrics_SRR23015874.txt
```
Repeat this for other samples.

Step 5: Realignment around indels

We'll use **GATK3** for this. It's a bit complex and has two steps: creating a table of possible indels and then realigning the indels.

```bash
java -jar GenomeAnalysisTK.jar \
    -T RealignerTargetCreator \
    -R hg38.fa \
    -I marked_duplicates_SRR23015874.bam \
    -o SRR23015874.intervals

java -jar GenomeAnalysisTK.jar \
    -T IndelRealigner \
    -R hg38.fa \
    -I marked_duplicates_SRR23015874.bam \
    -targetIntervals SRR23015874.intervals \
    -o realigned_SRR23015874.bam
```
Repeat this for other samples.

Step 6: Base recalibration

This step adjusts the quality scores of the reads, which can improve the accuracy of variant calls. This also uses **GATK3**.

```bash
java -jar GenomeAnalysisTK.jar \
    -T BaseRecalibrator \
    -R hg38.fa \
    -I realigned_SRR23015874.bam \
    -knownSites snp.vcf \
    -o recal_SRR23015874.table

java -jar GenomeAnalysisTK.jar \
    -T PrintReads \
    -R hg38.fa \
    -I realigned_SRR23015874.bam \
    -BQSR recal_SRR23015874.table \
    -o recalibrated_SRR23015874.bam
```

Step 7: Variant Calling

We'll use **Mutect2** from GATK for improved performance on somatic variation:

```bash
gatk Mutect2 \

| | |
|---|---|
| | -R hg38.fa \<br>-I recalibrated_SRR23015876.bam \<br>-I recalibrated_SRR23015877.bam \<br>-normal SRR23015874 \<br>-O unfiltered.vcf \<br>-tumor SRR23015876 SRR23015877<br>```<br><br>### Step 8: Variant Annotation<br><br>We'll use **ANNOVAR** for this.<br><br>```bash<br>table_annovar.pl unfiltered.vcf humandb/ -buildver hg38 -out annotated -remove -protocol refGene,cytoBand,exac03,avsnp147,dbnsfp30a -operation g,r,f,f,f -nastring . -vcfinput -polish -xreffile example/gene_xref.txt<br>```<br><br>Please remember, these are all examples and depending on the quality of your sequencing data, and the specific aims of your analyses, these parameters might need to be adjusted. You also might need to perform quality checks at each step to ensure everything is going as expected.<br><br>(Note: gatk, picard, Mutect need appropriate licences for usage and not mentioned their installation steps, please follow the user manuals to install them.) |

**WGS/WES data analysis** **Case 2.3: Structure variation identification with normal**

<table>
<tr><td>User's Inputs</td><td>You are a bioinformatician who specializes in writing code and performing analysis for a variety of bioinformatic data analysis tasks. Now I provide you with an Ubuntu 18.04 system where I save the following data, each line including the data path and data description.<br><br>SRR23015874_R1.fastq.gz: paired end read1 from human whole genome sequencing, the matched normal samples replicate 1<br>SRR23015874_R2.fastq.gz: paired end read2 from human whole genome sequencing, the matched normal samples replicate 1<br>SRR23015876_R1.fastq.gz: paired end read1 from human whole genome sequencing, tumor samples replicate 1<br>SRR23015876_R2.fastq.gz: paired end read2 from human whole genome sequencing, tumor samples replicate 1<br>SRR23015877_R1.fastq.gz: paired end read1 from human whole genome sequencing, tumor samples replicate 2<br>SRR23015877_R2.fastq.gz: paired end read2 from human whole genome sequencing, tumor samples replicate 2<br>TruSeq3-PE.fa: trimming adapter<br>hg38.fa: human hg38 reference genome fasta<br><br>I need you to help me plan what needs to be done in each step of the analysis process, and help me write the code for each step to install softwares, use tools and accomplish the following main goal:<br><br>To identify structural variations, which can include large insertions, deletions, inversions, duplications, and translocations, by comparing the sequencing data from the tumor sample over the matched normal sample</td></tr>
<tr><td>Generated Results by ChatGPT-4</td><td>Analysis of structural variations (SVs) usually involves the following steps:<br><br>1. Data Preprocessing<br>2. Alignment<br>3. Structural Variation Calling<br>4. Structural Variation Annotation<br><br>Here's a common pipeline performed on human data to identify structural variations.<br><br>### Step 1: Data Preprocessing<br><br>First, we need to trim the adapters and remove low-quality bases from the sequencing reads. As you've done before, you can use Trimmomatic for this.<br><br>```bash<br>java -jar Trimmomatic-0.39/trimmomatic-0.39.jar PE \<br>    -phred33 \<br>    SRR23015874_R1.fastq.gz SRR23015874_R2.fastq.gz \<br>    SRR23015874_R1_trimmed.fastq.gz<br>SRR23015874_R1_unpaired.fastq.gz \</td></tr>
</table>

SRR23015874_R2_trimmed.fastq.gz
SRR23015874_R2_unpaired.fastq.gz \
ILLUMINACLIP:TruSeq3-PE.fa:2:30:10 \
LEADING:3 TRAILING:3 SLIDINGWINDOW:4:15 MINLEN:36
```

Step 2: Alignment

Align the sequencing reads to the reference genome. We'll use BWA-MEM for this because it is great for structural variation calling since it properly assigns split read mappings, a valuable signal for structural variant calling.

```bash
bwa mem -t 4 -K 100000000 -Y hg38.fa SRR23015874_R1.fastq.gz
SRR23015874_R2.fastq.gz > aligned_reads.sam
```

Step 3: Structural Variation Calling

Next, we'll use a tool like **LUMPY** to perform our structural variant calling. LUMPY takes aligned reads in SAM/BAM format, which we just generated, as input.

First, let's sort our SAM file and convert it to BAM:

```bash
samtools sort -@ 4 -o sorted_reads.bam aligned_reads.sam
samtools index sorted_reads.bam
```

Then, we can install and run LUMPY:

```bash
# Download and install
git clone --recursive https://github.com/arq5x/lumpy-sv.git
cd lumpy-sv
make

# Run LUMPY
lumpyexpress -B sorted_reads.bam -S sample.splitters.bam -D
sample.discordants.bam -o variants.vcf
```

Step 4: Structural Variation Annotation

Finally, we want to annotate our found variants. **AnnotSV** is a great tool for structural variant annotation. You can annotate your found variants like so:

```bash
AnnotSV -SVinputFile variants.vcf -outputFile annotated_variants.tsv
```
```

	These steps will give you a basic pipeline for structural variant calling. Keep in mind that SV calling is a complex task and there might be additional steps or tools necessary for your specific project. SV calling usually requires careful filtering and manual review to validate the results. Also, make sure to follow the user manuals to install the tools correctly.

WGS/WES data analysis **Case 2.4: Structure variation identification without normal**

<table>
<tr><td>User's Inputs</td><td>You are a bioinformatician who specializes in writing code and performing analysis for a variety of bioinformatic data analysis tasks. Now I provide you with an Ubuntu 18.04 system where I save the following data, each line including the data path and data description.

SRR23015876_R1.fastq.gz: paired end read1 from human whole genome sequencing, tumor samples replicate 1
SRR23015876_R2.fastq.gz: paired end read2 from human whole genome sequencing, tumor samples replicate 1
SRR23015877_R1.fastq.gz: paired end read1 from human whole genome sequencing, tumor samples replicate 2
SRR23015877_R2.fastq.gz: paired end read2 from human whole genome sequencing, tumor samples replicate 2
TruSeq3-PE.fa: trimming adapter
hg38.fa: human hg38 reference genome fasta

I need you to help me plan what needs to be done in each step of the analysis process, and help me write the code for each step to install softwares, use tools and accomplish the following main goal:

To identify structural variations, which can include large insertions, deletions, inversions, duplications, and translocations based on the sequencing data from the tumor sample.</td></tr>
<tr><td>Generated Results by ChatGPT-4</td><td>Sure, the pipelines to identify structural variations (SVs) in tumor samples could include the following steps:

1. Data Preprocessing
2. Alignment to Reference
3. SV Detection
4. SV Annotation

Below is an example of code snippets to perform each step:

Step 1: Data Preprocessing
You can use Trimmomatic to trim adapters and remove low-quality bases from the sequencing reads.

Install Trimmomatic:

```bash
sudo apt-get update
sudo apt-get install trimmomatic
```

Run Trimmomatic to trim your data:

```bash
java -jar /usr/share/java/trimmomatic-0.36.jar PE -phred33 \
    SRR23015876_R1.fastq.gz SRR23015876_R2.fastq.gz \
    output_forward_paired.fq.gz output_forward_unpaired.fq.gz \</td></tr>
</table>

output_reverse_paired.fq.gz output_reverse_unpaired.fq.gz \
ILLUMINACLIP:TruSeq3-PE.fa:2:30:10 SLIDINGWINDOW:4:15
MINLEN:36
```

### Step 2: Alignment to Reference
Align the sequencing reads to the reference human genome (hg38 in your case) using BWA for SV calling as it properly assigns split read mappings, a valuable signal for structural variant calling.

Install BWA:

```bash
sudo apt-get install bwa
```

Index the reference genome and align the trimmed reads to the reference genome:

```bash
bwa index hg38.fa
bwa mem -t 8 hg38.fa output_forward_paired.fq.gz
output_reverse_paired.fq.gz > aln_reads.sam
```

Step 3: SV Detection
For SV detection, LUMPY can be applied. It is sensitive to all types of SVs.

First, convert the SAM file to sorted BAM:

```bash
samtools sort -@ 4 -o sorted_reads.bam aln_reads.sam
samtools index sorted_reads.bam
```

Then, to install LUMPY:

```bash
git clone --recursive https://github.com/arq5x/lumpy-sv.git
cd lumpy-sv
make
```

For running LUMPY:

```bash
./lumpyexpress -B sorted_reads.bam -S sample.splitters.bam -D
sample.discordants.bam -o variants.vcf
```

Step 4: SV Annotation
Finally, structural variant annotation can be performed with a tool such as AnnotSV.
```

	You will need to download and install AnnotSV following instructions from their [website](https://lbgi.fr/AnnotSV/). Then, you can annotate your found variants:<br><br>```bash<br>AnnotSV -SVinputFile variants.vcf -outputFile annotated_variants.tsv<br>```<br><br>This basic pipeline should help you identify structural variants in your tumor samples. Be aware that SV calling often requires careful filtering and additional validation steps.

ChIP-Seq Analysis   Case 3.1: Call Peaks

<table>
<tr>
<td>User’s Inputs</td>
<td>You are a bioinformatician who specializes in writing code and performing analysis for a variety of bioinformatic data analysis tasks. Now I provide you with an Ubuntu 18.04 system where I save the following data, each line including the data path and data description.

SRR620204.fastq.gz: chip-seq data for Ring1B
SRR620205.fastq.gz: chip-seq data for cbx7
SRR620206.fastq.gz: chip-seq data for SUZ12
SRR620208.fastq.gz: chip-seq data for IgGold
mm39.ncbiRefSeq.gtf: genome annotations mouse
mm39.fa: mouse genome

I need you to help me plan what needs to be done in each step of the analysis process, and help me write the code for each step to install softwares, use tools and accomplish the following main goal:

Call peaks for protein cbx7 with IgGold as control</td>
</tr>
<tr>
<td>Generated Results by ChatGPT-4</td>
<td>Let's plan the analysis process for peak calling. Here are the necessary steps:

1. Quality Control of Raw Reads
2. Alignment to Reference Genome
3. Sort and Index BAM Files
4. Peak Calling

Step 1: Quality Control of Raw Reads
Check the quality of reads with FastQC:

```bash
sudo apt-get install fastqc
fastqc SRR620205.fastq.gz SRR620208.fastq.gz
```

Step 2: Alignment to Reference Genome

Align the reads to the mouse genome using Bowtie2.

First, build a bowtie index for the mouse genome:

```bash
sudo apt-get install bowtie2
bowtie2-build mm39.fa mm39
```

Then, align the reads:

```bash
bowtie2 -x mm39 -U SRR620205.fastq.gz -S aligned_cbx7.sam
bowtie2 -x mm39 -U SRR620208.fastq.gz -S aligned_IgGold.sam
```</td>
</tr>
</table>

Step 3: Sort and Indexing BAM Files

BAM files need to be sorted and indexed using SAMtools:

```bash
sudo apt-get install samtools
samtools view -bS aligned_cbx7.sam | samtools sort - -o sorted_cbx7.bam
samtools view -bS aligned_IgGold.sam | samtools sort - -o
sorted_IgGold.bam
samtools index sorted_cbx7.bam
samtools index sorted_IgGold.bam
```

Step 4: Peak Calling

For peak calling, use MACS2. Before using MACS2, you need to install it along with its dependencies:

```bash
sudo apt-get install python3 python3-pip
pip3 install numpy scipy
pip3 install macs2
```

Then, perform peak calling for cbx7 with IgGold as a negative control:

```bash
macs2 callpeak -t sorted_cbx7.bam -c sorted_IgGold.bam -f BAM -g mm -n
cbx7_peaks -B -q 0.01
```

The result will be written to the file `cbx7_peaks_peaks.narrowPeak`, which contains the identified peak locations.

This pipeline provides a basic process to call peaks for ChIP-seq data. Depending on the quality and nature of the_INPUT, additional steps such as PCR duplicate removal, blacklisting regions, or downstream analysis such as motif analysis might be necessary.

ChIP-Seq Analysis Case 3.2: Motif Discovery

<table>
<tr>
<td>User’s Inputs</td>
<td>You are a bioinformatician who specializes in writing code and performing analysis for a variety of bioinformatic data analysis tasks. Now I provide you with an Ubuntu 18.04 system where I save the following data, each line including the data path and data description.

SRR620204.fastq.gz: chip-seq data for Ring1B
SRR620205.fastq.gz: chip-seq data for cbx7
SRR620206.fastq.gz: chip-seq data for SUZ12
SRR620208.fastq.gz: chip-seq data for IgGold
mm39.ncbiRefSeq.gtf: genome annotations mouse
mm39.fa: mouse genome

I need you to help me plan what needs to be done in each step of the analysis process, and help me write the code for each step to install softwares, use tools and accomplish the following main goal:

Discover motifs within the peaks of protein SUZ12 with IgGold as control</td>
</tr>
<tr>
<td>Generated Results by ChatGPT-4</td>
<td>In order to identify the DNA sequence motifs within the peaks of protein "SUZ12", we may follow these steps:

1. Quality control of raw reads
2. Alignment to reference genome
3. Sort and index BAM files
4. Peak calling
5. Extract peak sequences
6. Motif discovery

Below are the codes for each step:

Step 1: Quality control of raw reads
FastQC can be used to perform quality checks on raw sequence data:

```bash
sudo apt-get install fastqc
fastqc SRR620206.fastq.gz SRR620208.fastq.gz
```

Step 2: Alignment to reference genome

Align the reads to the mouse genome using Bowtie2:

```bash
sudo apt-get install bowtie2
bowtie2-build mm39.fa mm39
bowtie2 -x mm39 -U SRR620206.fastq.gz -S aligned_SUZ12.sam
bowtie2 -x mm39 -U SRR620208.fastq.gz -S aligned_IgGold.sam
```

Step 3: Sort and index BAM files

BAM files need to be sorted and indexed using SAMtools:</td>
</tr>
</table>

```bash
sudo apt-get install samtools
samtools view -bS aligned_SUZ12.sam | samtools sort - -o sorted_SUZ12.bam
samtools view -bS aligned_IgGold.sam | samtools sort - -o sorted_IgGold.bam
samtools index sorted_SUZ12.bam
samtools index sorted_IgGold.bam
```

Step 4: Peak calling

Use MACS2 for peak calling. You can install it and perform peak calling for SUZ12 with IgGold as control:

```bash
sudo apt-get install python3 python3-pip
pip3 install numpy scipy
pip3 install macs2

macs2 callpeak -t sorted_SUZ12.bam -c sorted_IgGold.bam -f BAM -g mm -n SUZ12_peaks -B -q 0.01
```

Step 5: Extract peak sequences

Convert the peak coordinates into FASTA sequences using bedtools:

```bash
sudo apt-get install bedtools
bedtools getfasta -fi mm39.fa -bed SUZ12_peaks_peaks.narrowPeak > peak_sequences.fa
```

Step 6: Motif discovery

A tool like MEME can be used to discover motifs in the sequences:

```bash
sudo apt-get install meme
meme peak_sequences.fa -maxsites 2000 -nmotifs 10 -dna
```

These steps should help you discover motifs within the peaks of protein SUZ12. Always remember to interpret these results carefully and possibly consider further downstream analyses to validate the findings. Different parameters and tools might also be necessary depending on the specific characteristics of your data.

ChIP-Seq Analysis   Case 3.3: Functional Enrichment

**User's Inputs**	You are a bioinformatician who specializes in writing code and performing analysis for a variety of bioinformatic data analysis tasks. Now I provide you with an Ubuntu 18.04 system where I save the following data, each line including the data path and data description.<br><br>SRR620204.fastq.gz: chip-seq data for Ring1B<br>SRR620205.fastq.gz: chip-seq data for cbx7<br>SRR620206.fastq.gz: chip-seq data for SUZ12<br>SRR620208.fastq.gz: chip-seq data for IgGold<br>mm39.ncbiRefSeq.gtf: genome annotations mouse<br>mm39.fa: mouse genome<br><br>I need you to help me plan what needs to be done in each step of the analysis process, and help me write the code for each step to install softwares, use tools and accomplish the following main goal:<br><br>Perform functional enrichment for protein Ring1B, use protein IgGold as the control
**Generated Results by ChatGPT-4**	In order to perform functional enrichment for the protein "Ring1B", you would generally follow these steps:<br><br>1. Quality control of raw reads<br>2. Alignment to reference genome<br>3. Sort and index BAM files<br>4. Peak Calling<br>5. Annotating peaks<br>6. Functional enrichment analysis<br><br>Please note that the annotation and functional enrichment steps often require further manual review and interpretation. Here's how you might tackle each step:<br><br>### Step 1: Quality control of raw reads<br>FastQC can be used to perform quality checks on raw sequence data:<br><br>```bash<br>sudo apt-get install fastqc<br>fastqc SRR620204.fastq.gz SRR620208.fastq.gz<br>```<br><br>### Step 2: Alignment to reference genome<br><br>Bowtie2 is commonly used for alignment:<br><br>```bash<br>sudo apt-get install bowtie2<br>bowtie2-build mm39.fa mm39<br>bowtie2 -x mm39 -U SRR620204.fastq.gz -S aligned_Ring1B.sam<br>bowtie2 -x mm39 -U SRR620208.fastq.gz -S aligned_IgGold.sam<br>```

Step 3: Sort and index BAM files

SAMtools can be used to sort and index BAM files:

```bash
sudo apt-get install samtools
samtools view -bS aligned_Ring1B.sam | samtools sort - -o
sorted_Ring1B.bam
samtools view -bS aligned_IgGold.sam | samtools sort - -o
sorted_IgGold.bam
samtools index sorted_Ring1B.bam
samtools index sorted_IgGold.bam
```

Step 4: Peak Calling

Use MACS2 for peak calling:

```bash
sudo apt-get install python3 python3-pip
pip3 install numpy scipy
pip3 install macs2

macs2 callpeak -t sorted_Ring1B.bam -c sorted_IgGold.bam -f BAM -g mm
-n Ring1B_peaks -B -q 0.01
```

Step 5: Annotating peaks

You can use BEDTools and a gene annotation file (like the provided GTF file) to annotate the peaks:

```bash
sudo apt-get install bedtools
bedtools intersect -wa -wb -a Ring1B_peaks_filterdup_peaks.narrowPeak -
b mm39.ncbiRefSeq.gtf > annotated_peaks.txt
```

Step 6: Functional enrichment analysis

To perform the functional enrichment analysis, you will likely want to use an R package like clusterProfiler or a web tool such as the Database for Annotation, Visualization, and Integrated Discovery (DAVID). These tools will help you identify which Gene Ontology (GO) terms, pathways, or other functional categories are overrepresented in the genes near your ChIP-seq peaks.

Please note you would need to extract the gene symbols from the annotated peaks file and prepare a gene list as input for these tools. This last step would most likely be performed manually or with a custom script depending on the specifics of the output file from the annotation step.

Bisulfite-Seq data analysis   Case 4.1: Identifying DNA methylation

<table>
<tr>
<td>User's Inputs</td>
<td>You are a bioinformatician who specializes in writing code and performing analysis for a variety of bioinformatic data analysis tasks. Now I provide you with an Ubuntu 18.04 system where I save the following data, each line including the data path and data description.

Sample_R1.fastq.gz: Raw sequencing reads from the bisulfite-converted DNA, typically paired-end reads. These reads contain information about the converted DNA, where unmethylated cytosines are converted to uracils (T) during library preparation.
Sample_R2.fastq.gz: Paired-end reads that complement the R1 reads. These are typically used for quality control and paired-end analysis.
TruSeq3-PE.fa: Adapter sequences for trimming
Reference.fasta: Reference genome for the organism under study in FASTA format, include bisulfite conversion.

I need you to help me plan what needs to be done in each step of the analysis process, and help me write the code for each step to install softwares, use tools and accomplish the following main goal:

To identify DNA methylation patterns in the genome based on the Bisulfite-Seq data.</td>
</tr>
<tr>
<td>Generated Results by ChatGPT-4</td>
<td>The usual steps for a Bisulfite-Seq data analysis include the following:

1. Quality Control of Raw Reads
2. Trimming Adapters and Low Quality Bases
3. Alignment to Reference Genome
4. Methylation Extraction
5. Methylation Analysis

Here is how you can go through each step:

Step 1: Quality Control of Raw Reads
Inspect the quality of the reads using FastQC:

```bash
sudo apt-get install fastqc
fastqc Sample_R1.fastq.gz Sample_R2.fastq.gz
```

Step 2: Trimming Adapters and Low Quality Bases
Trim adapters and low quality bases using Trimmomatic:

```bash
sudo apt-get install trimmomatic
java -jar /usr/share/java/trimmomatic.jar PE -phred33 \
    Sample_R1.fastq.gz Sample_R2.fastq.gz \
    output_forward_paired.fq.gz output_forward_unpaired.fq.gz \
    output_reverse_paired.fq.gz output_reverse_unpaired.fq.gz \
    ILLUMINACLIP:TruSeq3-PE.fa:2:30:10 SLIDINGWINDOW:4:15
MINLEN:36</td>
</tr>
</table>

```

### Step 3: Alignment to Reference Genome
For Bisulfite-Seq, Bismark is widely used because it can handle converted genomes. First, you'll need to prepare the genome:

```bash
bismark_genome_preparation --path_to_aligner /usr/bin/bowtie2/ --verbose
/path_to_genome_folder/
```

Then, perform the alignment:

```bash
bismark --path_to_bowtie /usr/bin/bowtie2/ --multicore 2 --paired-end \
    /path_to_genome_folder/ \
    output_forward_paired.fq.gz output_reverse_paired.fq.gz
```

Step 4: Methylation Extraction
Next, extract methylation data with Bismark methylation extractor:

```bash
bismark_methylation_extractor --gzip --multicore 2 \
    --bedGraph --counts --report --buffer_size 10G --output \
    /path_to_output/ \
    aligned_sequence.bam
```

Step 5: Methylation Analysis
Afterwards, you can use the methylation information obtained in the form of a bedGraph or other format to detect differential methylation or visualize data with tools like IGV.

Remember that interpretation of methylation can be complex and heavily dependent on biological context, i.e., the function of regions that show differential methylation and whether these changes occur in a tissue or developmental stage-specific manner. Also biological replicates are often required to derive meaningful conclusions from the data.
```

ATAC-seq data analysis **Case 5.1: Identifying open chromatin regions**

**User's Inputs**	You are a bioinformatician who specializes in writing code and performing analysis for a variety of bioinformatic data analysis tasks. Now I provide you with an Ubuntu 18.04 system where I save the following data, each line including the data path and data description.<br><br>Sample_R1.fastq.gz: Raw sequencing reads from the ATAC-seq experiment. These are typically paired-end reads representing fragments of open chromatin regions.<br>Sample_R2.fastq.gz: Paired-end reads that complement the R1 reads.<br>TruSeq3-PE.fa: Adapter sequences for trimming<br>Reference.fasta: Reference genome for the organism under study in FASTA format. It matches the genome used for the ATAC-seq library preparation.<br><br>I need you to help me plan what needs to be done in each step of the analysis process, and help me write the code for each step to install softwares, use tools and accomplish the following main goal:<br><br>To identify open chromatin regions within the genome based on the ATAC-seq data.
**Generated Results by ChatGPT-4**	The steps to analyze ATAC-seq data are as follows:<br><br>1. Raw Data Quality Control<br>2. Adapter Trimming and Quality Filtering<br>3. Aligning Reads to a Reference Genome<br>4. Removing Duplicates<br>5. Peak Calling<br><br>You can follow the following steps:<br><br>### Step 1: Raw Data Quality Control<br>FastQC can be used to check the quality of the raw data:<br><br>```bash<br>sudo apt-get install fastqc<br>fastqc Sample_R1.fastq.gz Sample_R2.fastq.gz<br>```<br><br>### Step 2: Adapter Trimming and Quality Filtering<br>Trim adapters and filter low quality reads using Trimmomatic:<br><br>```bash<br>sudo apt-get install trimmomatic<br>java -jar /usr/share/java/trimmomatic.jar PE -phred33 \\<br>Sample_R1.fastq.gz Sample_R2.fastq.gz \\<br>trimmed_R1.fastq.gz unpaired_R1.fastq.gz \\<br>trimmed_R2.fastq.gz unpaired_R2.fastq.gz \\<br>ILLUMINACLIP:TruSeq3-PE.fa:2:30:10 LEADING:20 TRAILING:20 SLIDINGWINDOW:4:30 MINLEN:36<br>```

Step 3: Aligning Reads to a Reference Genome
Bowtie2 is a commonly used tool for alignment. Be sure you've indexed your reference genome before running the alignment:

```bash
sudo apt-get install bowtie2
bowtie2-build Reference.fasta Reference
bowtie2 -x Reference -1 trimmed_R1.fastq.gz -2 trimmed_R2.fastq.gz -S aligned.sam
```

Step 4: Removing Duplicates
Duplicates are removed from aligned reads to account for PCR bias. Samtools can be used in this process:

```bash
sudo apt-get install samtools
samtools view -bS aligned.sam > aligned.bam
samtools sort aligned.bam -o aligned_sorted.bam
samtools rmdup aligned_sorted.bam aligned_sorted_rmdup.bam
```

Step 5: Peak Calling
MACS2 is widely used for peak calling:

```bash
pip3 install macs2
macs2 callpeak -t aligned_sorted_rmdup.bam -f BAMPE -n ATACseq -g hs -q 0.05 --nomodel --shift -100 --extsize 200
```

Bedgraph files from MACS2 can be visualized in e.g., IGV.

For downstream analysis, you might want to use the narrowPeak or summit files generated by MACS2 and conduct motif analysis, peak annotation, differential openness analysis, etc., according to your specific research question. Note that the parameters for all commands, particularly in the MACS2 command can be altered to fit the needs of your specific experiment and data.

DNase-seq data analysis **Case 6.1: Identifying DnaseI hypersensitive site**

<table>
<tr><td>User's Inputs</td><td>You are a bioinformatician who specializes in writing code and performing analysis for a variety of bioinformatic data analysis tasks. Now I provide you with an Ubuntu 18.04 system where I save the following data, each line including the data path and data description.

Sample_R1.fastq.gz: Raw sequencing reads from the DNase-seq experiment.
Sample_R2.fastq.gz: Paired-end reads that complement the R1 reads, often used for quality control and paired-end analysis.
TruSeq3-PE.fa: Adapter sequences for trimming.
Reference.fasta: Reference genome for the organism under study in FASTA format

I need you to help me plan what needs to be done in each step of the analysis process, and help me write the code for each step to install softwares, use tools and accomplish the following main goal:

To identify DNase I hypersensitive sites (regions of open chromatin) within the genome based on the DNase-seq data.</td></tr>
<tr><td>Generated Results by ChatGPT-4</td><td>Sure, here are the typical steps to analyze the DNase-seq data and identify DNase I hypersensitive sites:

1. Quality Control of Raw Data
2. Trimming Adapters
3. Aligning Reads to a Reference Genome
4. Marking Duplicates
5. Peak Calling

Step 1: Quality Control of Raw Data
FastQC can be used to check the quality of the raw reads.

```bash
sudo apt-get install fastqc
fastqc Sample_R1.fastq.gz Sample_R2.fastq.gz
```

Step 2: Trimming Adapters
Trimmomatic can be used to remove the adapter sequences and perform quality Trimmming.

```bash
sudo apt-get install trimmomatic
java -jar /usr/share/java/trimmomatic.jar PE -phred33 Sample_R1.fastq.gz Sample_R2.fastq.gz \
ILUMINACLIP:TruSeq3-PE.fa:2:30:10 LEADING:3 TRAILING:3 SLIDINGWINDOW:4:15 MINLEN:36
```

Step 3: Aligning Reads to a Reference Genome
Align the reads to the reference genome using BWA.</td></tr>
</table>

```bash
sudo apt-get install bwa
bwa index Reference.fasta
bwa mem Reference.fasta trimmed_R1.fastq.gz trimmed_R2.fastq.gz > aligned_reads.sam
```

Step 4: Marking Duplicates
Mark duplicate reads (likely arising from PCR duplication) using Picard.

```bash
sudo apt-get install picard-tools
java -jar picard.jar MarkDuplicates INPUT=aligned_reads.sam OUTPUT=marked_duplicates.bam METRICS_FILE=metrics.txt
```

Step 5: Peak Calling
Peak calling can be performed using MACS2.

```bash
pip3 install macs2
macs2 callpeak -t marked_duplicates.bam -f BAMPE -n DNase -g hs -B -q 0.05
```

The output from peak calling can be used to identify DNase I hypersensitive sites across the genome. Further downstream analysis might involve, for example, investigating the genomic features (like promoters, enhancers) that are associated with these sites, or the biological pathways and processes that are associated with the genes near these sites. Make sure the parameters used in each step fit the specific requirements for your experiment and dataset.

4C-seq data analysis   Case 7.1: Find genomics interactions

<table>
<tr><td>User's Inputs</td><td>You are a bioinformatician who specializes in writing code and performing analysis for a variety of bioinformatic data analysis tasks. Now I provide you with an Ubuntu 18.04 system where I save the following data, each line including the data path and data description.

sample_4C-seq_data.fastq: Raw sequencing reads or aligned data from the 4C-seq experiment, depending on the starting point.
viewpoint_coordinates.bed: A file specifying the genomic coordinates of the viewpoint we want to study.
reference_genome.fasta: A reference genome in FASTA format for the organism under study. This should match the genome used for the 4C-seq library preparation.

I need you to help me plan what needs to be done in each step of the analysis process, and help me write the code for each step to install softwares, use tools and accomplish the following main goal:

To identify genomic interactions originating from a specific genomic viewpoint using 4C-seq data.</td></tr>
<tr><td>Generated Results by ChatGPT-4</td><td>The general steps to analyze the 4C-seq data usually include:

- Quality Control of Raw Reads
- Alignment of Reads to a Reference Genome
- Filtering and Collapsing Reads
- Normalization
- Interaction Calling

Here's how you might perform each step:

Step 1: Quality Control of Raw Reads

You can use FastQC for quality control:

```bash
sudo apt-get install fastqc
fastqc sample_4C-seq_data.fastq
```

Step 2: Alignment of Reads to a Reference Genome

Align the reads to the reference genome. This can be done using tools like Bowtie2:

```bash
sudo apt-get install bowtie2
bowtie2-build reference_genome.fasta reference
bowtie2 -x reference -U sample_4C-seq_data.fastq -S aligned.sam
```

Step 3: Filtering and Collapsing Reads</td></tr>
</table>

Depending on the specific 4C-seq protocol and the sequencing depth, you might need to filter and collapse reads. This can be done using custom scripts or tools like 'vcf', 'bam' files using Samtools.

```bash
sudo apt-get install samtools
samtools view -bS aligned.sam > aligned.bam
samtools sort aligned.bam -o sorted.bam
samtools rmdup sorted.bam filtered.bam
```

Step 4: Normalization

You would use a tool specifically designed for 4C-seq data, such as `FourCSeq`, which is an R package that implements multiple normalization methods:

```R
if (!requireNamespace("BiocManager", quietly = TRUE))
   install.packages("BiocManager")
BiocManager::install("FourCSeq")

library(FourCSeq)
# Assume that you've customer data and/or read from .bed and .bam files.
# The data preparation process can be specific depending on your
experiment
# So here we skip data preparation details by assuming 'fs' as FourCSeq
object
normCounts(fs) <- normVST(normCounts(fs),
                   blind = FALSE,
                   parametric = TRUE)
```

Step 5: Interaction Calling

Interaction calling can be performed using `FourCSeq` as well to identify regions differentially interacting with the viewpoint.

```R
res <- diffInteraction(fs)
res <- lapply(res, rbind, do.call(rbind, res))
```

4C-seq analysis can be complex and can require fine-tuning of parameters, adjustments for multiple comparisons, and careful interpretation of results, particularly when identifying differential interactions.

Nanopore DNA sequencing data analysis   Case 8.1: Genome assembly

**User's Inputs**	You are a bioinformatician who specializes in writing code and performing analysis for a variety of bioinformatic data analysis tasks. Now I provide you with an Ubuntu 18.04 system where I save the following data, each line including the data path and data description.<br><br>sample_nanopore_reads.fastq: Raw Nanopore sequencing data in fastq format.<br>eference_genome.fasta: A reference genome in FASTA format, if a closely related reference is available and used as a guide during the assembly<br><br>I need you to help me plan what needs to be done in each step of the analysis process, and help me write the code for each step to install softwares, use tools and accomplish the following main goal:<br><br>To assemble a genome from the Nanopore sequencing data.
**Generated Results by ChatGPT-4**	Here are the steps typically involved in the assembly of a genome from Nanopore sequencing data:<br><br>1. Quality Control of Raw Reads<br>2. Genome Assembly<br>3. Assembly Polishing<br>4. Assembly Evaluation<br><br>Below is how you perform each step:<br><br>### Step 1: Quality Control of Raw Reads<br>FastQC can be used to assess the quality of the reads.<br><br>```bash<br>sudo apt-get install fastqc<br>fastqc sample_nanopore_reads.fastq<br>```<br><br>### Step 2: Genome Assembly<br>The long reads from Nanopore sequencing data can be assembled using tools like Canu, Flye, or wtdbg2. Here is an example using Flye.<br><br>First, install Flye using conda:<br><br>```bash<br>conda install -c bioconda flye<br>```<br><br>Then run Flye to assemble the genome:<br><br>```bash<br>flye --nano-raw sample_nanopore_reads.fastq --out-dir assembly --genome-size 5m<br>```

Replace '5m' with an estimate of your own genome size (you can denote size in k, m, g for kilobases, megabases, and gigabases, respectively).

Step 3: Assembly Polishing
Polishing consists of error correction steps made after the initial assembly, this can greatly increase the quality of the assembly. First, map the reads back to the assembled contigs:

```bash
minimap2 -ax map-ont assembly/assembly.fasta
sample_nanopore_reads.fastq > aligned_reads.sam
```

Convert SAM to BAM:

```bash
samtools view -b aligned_reads.sam | samtools sort - -o aligned_reads.bam
samtools index aligned_reads.bam
```

Then run Racon for the first round of polishing:

```bash
racon sample_nanopore_reads.fastq aligned_reads.sam
assembly/assembly.fasta > polished_assembly.fasta
```

After this, you may want to repeat the alignment and do further rounds of polishing with Racon, or use another tool like Medaka or Nanopolish for subsequent rounds.

Step 4: Assembly Evaluation
One option to assess the quality of the assembly is with QUAST.

```bash
quast polished_assembly.fasta -o report
```

That's the overview of the process. Please note that parameters (such as genome size in Flye) may need to be adjusted to fit the dictates of your particular data and project.

Nanopore DNA sequencing data analysis   Case 8.2: Tandem repeats variation identification

<table>
<tr><td>User's Inputs</td><td>You are a bioinformatician who specializes in writing code and performing analysis for a variety of bioinformatic data analysis tasks. Now I provide you with an Ubuntu 18.04 system where I save the following data, each line including the data path and data description.

sample_nanopore_reads.fastq: Raw Nanopore sequencing data in fastq format.
eference_genome.fasta: A reference genome in FASTA format, if a closely related reference is available and used as a guide during the assembly

I need you to help me plan what needs to be done in each step of the analysis process, and help me write the code for each step to install softwares, use tools and accomplish the following main goal:

To identify tandem repeats.</td></tr>
<tr><td>Generated Results by ChatGPT-4</td><td>Sure, the steps to identify tandem repeats from the Nanopore sequencing data often involve:

1. Quality Control of Raw Reads
2. Reads Alignment to Reference Genome
3. Repeat Identification

Step 1: Quality Control of Raw Reads

Qualify the raw reads using FastQC:

```bash
sudo apt-get install fastqc
fastqc sample_nanopore_reads.fastq
```

Step 2: Reads Alignment to Reference Genome

Next, you'll need to align the reads to the reference genome. Here I use minimap2:

```bash
Install minimap2
conda install -c bioconda minimap2

Align the reads
minimap2 -ax map-ont reference_genome.fasta
sample_nanopore_reads.fastq > aligned.sam
```

Step 3: Repeat Identification

For tandem repeat identification you can use Tandem Repeat Finder (TRF) or similar softwares. Here's how to use TRF:

```bash</td></tr>
</table>

	# Download and extract (visit the TRF site for the most recent version)<br>wget https://tandem.bu.edu/trf/downloads/trf409.linux64<br>chmod 755 trf409.linux64<br><br># Run TRF<br>./trf409.linux64 reference_genome.fasta 2 7 7 80 10 50 500 -d -h<br>```<br><br>This will output a `.dat` file that contains the identified tandem repeats.<br><br>Parameters should be adapted to best suit your specific data. In the TRF example above, we used `2 7 7 80 10 50 500`, which are the default parameters suggested by the TRF manual (match, mismatch, delta, PM, PI, minscore, and max period). For more details, refer to the TRF manual.

PacBio DNA sequencing data analysis **Case 9.1: Genome assembly**

User's Inputs

You are a bioinformatician who specializes in writing code and performing analysis for a variety of bioinformatic data analysis tasks. Now I provide you with an Ubuntu 18.04 system where I save the following data, each line including the data path and data description.

sample_pacbio_reads.fastq: Raw PacBio sequencing data in fastq format.
eference_genome.fasta: A reference genome in FASTA format, if a closely related reference is available and used as a guide during the assembly

I need you to help me plan what needs to be done in each step of the analysis process, and help me write the code for each step to install softwares, use tools and accomplish the following main goal:

To assemble a genome from the PacBio sequencing data.

Generated Results by ChatGPT-4

The raw data quality check, assembly, polishing, and assembly evaluation are the general steps when assembling a genome from sequencing data.

Step 1: Quality Control of Raw Reads

FastQC should handle this:

```bash
sudo apt-get install fastqc
fastqc sample_pacbio_reads.fastq
```

Step 2: Genome Assembly

The PacBio (now PacBio Sequel) data assembly requires specific software. One popular choice is Canu, which is a fork of the Celera Assembler designed for long reads.

```bash
# Install Canu via conda
conda install -c bioconda canu

# Run Canu
canu -p assembly -d assembly_dir genomeSize=<estimate your genome size> -pacbio-raw sample_pacbio_reads.fastq
```

Please replace `<estimate your genome size>` with the approximate size of your reference genome (e.g., 4.6m for a 4.6 Mb genome).

Step 3: Assembly Polishing

Incorporating PacBio's tools might further improve assembly. Arrow (part of GenomicConsensus package from PacBio) is a choice:

```bash
# Assume that you have installed blasr and pbalign for read alignment
# Align raw reads to the assembly
```

	pbalign --nproc 10 sample_pacbio_reads.fastq assembly/assembly.fasta aligned_reads.bam<br><br># Create reference fasta index file<br>samtools faidx assembly.fasta<br><br># Genome consensus calling utilizing Arrow<br>arrow aligned_reads.bam -r assembly.fasta -o variants.gff -o consensus.fasta -j 20<br>```<br><br>### Step 4: Assembly Evaluation<br><br>Tools like QUAST can evaluate the assembly:<br><br>```bash<br># Install and run QUAST<br>conda install -c bioconda quast<br>quast.py consensus.fasta -o quast_report<br>```<br><br>The codes might slightly vary due to the complexity and variability of bioinformatic data and their analyses. Ensure to validate every step and adjust parameters when necessary.

RNA-Seq Analysis Case 10.1: Find differentially expressed genes

**User's Inputs**	You are a bioinformatician who specializes in writing code and performing analysis for a variety of bioinformatic data analysis tasks. Now I provide you with an Ubuntu 18.04 system where I save the following data, each line including the data path and data description.<br><br>SRR1374921.fastq.gz: single-end mouse rna-seq reads, replicate 1 in LoGlu group<br>SRR1374922.fastq.gz: single-end mouse rna-seq reads, replicate 2 in LoGlu group<br>SRR1374923.fastq.gz: single-end mouse rna-seq reads, replicate 1 in HiGlu group<br>SRR1374924.fastq.gz: single-end mouse rna-seq reads, replicate 2 in HiGlu group<br>TruSeq3-SE.fa: trimming adapter<br>mm39.fa: mouse mm39 genome fasta<br>mm39.ncbiRefSeq.gtf: mouse mm39 genome annotation<br><br>I need you to help me plan what needs to be done in each step of the analysis process, and help me write the code for each step to install softwares, use tools and accomplish the following main goal:<br><br>Find the differentially expressed genes
**Generated Results by ChatGPT-4**	To find differentially expressed genes with RNA-seq, several approaches can be performed including the QC of raw reads, trimming adapters, aligning reads, counting transcripts, and differential expression. Here is the suggested path:<br><br>### Step 1: Quality Control of Raw Reads (using FastQC)<br>```bash<br>sudo apt-get install fastqc<br>fastqc *.fastq.gz<br>```<br><br>### Step 2: Trimming Adapters (using Trimmomatic for Single-End)<br>```bash<br>sudo apt-get install trimmomatic<br>for sampleName in 'SRR1374921' 'SRR1374922' 'SRR1374923' 'SRR1374924'<br>do<br>java -jar /usr/share/java/trimmomatic.jar SE -phred33 \<br>${sampleName}.fastq.gz \<br>${sampleName}_trimmed.fastq.gz \<br>ILLUMINACLIP:TruSeq3-SE.fa:2:30:10 LEADING:3 TRAILING:3 SLIDINGWINDOW:4:15 MINLEN:36<br>done<br>```

Step 3: Aligning Reads to the Reference Genome (using Hisat2)
```bash
sudo apt-get install hisat2
hisat2-build mm39.fa mm39_index
for sampleName in 'SRR1374921' 'SRR1374922' 'SRR1374923'
'SRR1374924'
do
    hisat2 -x mm39_index -U ${sampleName}_trimmed.fastq.gz -S
${sampleName}.sam
done
```

Step 4: Convert SAM to BAM and Sort (via Samtools)
```bash
sudo apt-get install samtools
for sampleName in 'SRR1374921' 'SRR1374922' 'SRR1374923'
'SRR1374924'
do
    samtools view -S -b ${sampleName}.sam > ${sampleName}.bam
    samtools sort ${sampleName}.bam -o ${sampleName}_sorted.bam
    samtools index ${sampleName}_sorted.bam
done
```

Step 5: Counting Transcripts (using HTSeq-count)
```bash
sudo apt-get install htseq
for sampleName in 'SRR1374921' 'SRR1374922' 'SRR1374923'
'SRR1374924'
do
    htseq-count --stranded=no -f bam -r pos ${sampleName}_sorted.bam
mm39.ncbiRefSeq.gtf > ${sampleName}_counts.txt
done
```

Step 6: Differential Expression (with DESeq2)
Assuming you have R configured with DESeq2 installed:
```R
library(DESeq2)

# Load data from HTSeq
count_files <- list.files(pattern="_counts.txt$")
countdata <- sapply(count_files, function(path)
                data.frame(counts=as.integer(read.table(path, header=F)$V2),
check.names=F), simplify='data.frame')
rownames(countdata) <- read.table(count_files[1], header = FALSE)$V1

# Specify condition of each sample
condition <- factor(c("LoGlu", "LoGlu", "HiGlu", "HiGlu"))

# Create a DESeq dataset
coldata <- data.frame(row.names = colnames(countdata), condition)
```

	dds <- DESeqDataSetFromMatrix(countData = countdata, colData = coldata, design = ~ condition)<br><br># Perform differential expression<br>dds <- DESeq(dds)<br>res <- results(dds)<br>```<br><br>Adjoining each step could help determine parameters to fit specific requirements for your experiment/dataset. Please examine data after each critical step to ensure quality. If results appear unexpected, it may be that quality control measures were overlooked before these steps, so it's important to check data before differential expression analysis.

RNA-Seq Analysis   Case 10.2: Identify top5 down-regulated genes in HiGlu group

User's Inputs

You are a bioinformatician who specializes in writing code and performing analysis for a variety of bioinformatic data analysis tasks. Now I provide you with an Ubuntu 18.04 system where I save the following data, each line including the data path and data description.

SRR1374921.fastq.gz: single-end mouse rna-seq reads, replicate 1 in LoGlu group
SRR1374922.fastq.gz: single-end mouse rna-seq reads, replicate 2 in LoGlu group
SRR1374923.fastq.gz: single-end mouse rna-seq reads, replicate 1 in HiGlu group
SRR1374924.fastq.gz: single-end mouse rna-seq reads, replicate 2 in HiGlu group
TruSeq3-SE.fa: trimming adapter
mm39.fa: mouse mm39 genome fasta
mm39.ncbiRefSeq.gtf: mouse mm39 genome annotation

I need you to help me plan what needs to be done in each step of the analysis process, and help me write the code for each step to install softwares, use tools and accomplish the following main goal:

Identify top5 down-regulated genes in HiGlu group

Generated Results by ChatGPT-4

To identify downregulated genes, we generally follow these steps:

1. Quality Control of Raw Reads
2. Reads Trimming
3. Aligning Reads to the Reference Genome
4. Read Counting for Genomic Features
5. Differential Expression Analysis

Here are the commands for each step:

Step 1: Quality Control of Raw Reads

Using FastQC:

```bash
sudo apt-get install fastqc
fastqc *.fastq.gz
```

Step 2: Trimming Adapters
Utilizing Trimmomatic:

```bash
sudo apt-get install trimmomatic
for sampleName in 'SRR1374921' 'SRR1374922' 'SRR1374923' 'SRR1374924'
do
    java -jar /usr/share/java/trimmomatic.jar SE -phred33 \
    ${sampleName}.fastq.gz \
```

    ${sampleName}_trimmed.fastq.gz \
    ILLUMINACLIP:TruSeq3-SE.fa:2:30:10 LEADING:3 TRAILING:3
SLIDINGWINDOW:4:15 MINLEN:36
done
```

### Step 3: Aligning Reads to the Reference Genome
Making use of Hisat2:

```bash
sudo apt-get install hisat2
hisat2-build mm39.fa mm39_index
for sampleName in 'SRR1374921' 'SRR1374922' 'SRR1374923'
'SRR1374924'
do
    hisat2 -x mm39_index -U ${sampleName}_trimmed.fastq.gz -S
${sampleName}.sam
done
```

Step 4: Read Counting for Genomic Features
Applying HTSeq:

```bash
sudo apt-get install htseq
for sampleName in 'SRR1374921' 'SRR1374922' 'SRR1374923'
'SRR1374924'
do
    htseq-count -s no -f sam ${sampleName}.sam mm39.ncbiRefSeq.gtf >
${sampleName}_counts.txt
done
```

Step 5: Differential Expression Analysis
Finally, perform DESeq2 in an R environment:

```R
library(DESeq2)

# prepare a metadata dataframe for DESeqDataSetFromHTSeqCount
function
samplesInfo <- as.data.frame(matrix(ncol = 2, nrow = 4))
rownames(samplesInfo) <- c('SRR1374921', 'SRR1374922', 'SRR1374923',
'SRR1374924')
colnames(samplesInfo) <- c('condition', 'type')
samplesInfo$condition <- c('LoGlu', 'LoGlu', 'HiGlu', 'HiGlu')
samplesInfo$type <- rep('single-end', 4)

ddsHTSeq <- DESeqDataSetFromHTSeqCount(sampleTable =
samplesInfo,
                                    directory = "/path/to/your/count/files/",
                                    design = ~ condition)

| | |
|---|---|
| | dds <- DESeq(ddsHTSeq)<br>res <- results(dds, contrast=c("condition","HiGlu","LoGlu"))<br>resOrdered <- res[order(res$log2FoldChange),]  #sort by log fold change<br>head(resOrdered, 5)  #print top 5 down-regulated genes<br>```<br>Please replace "/path/to/your/count/files/" with your actual directory path.<br><br>This program provides the top 5 downregulated genes in the HiGLu group. Note that the results can vary slightly based on the precise parameters used in the above steps. |

**RNA-Seq Analysis        Case 10.3: Predict Fusion genes**

<table>
<tr><td>User's Inputs</td><td>You are a bioinformatician who specializes in writing code and performing analysis for a variety of bioinformatic data analysis tasks. Now I provide you with an Ubuntu 18.04 system where I save the following data, each line including the data path and data description.<br><br>rnaseq_1.fastq.gz: RNA-Seq read 1 data (left read)<br>rnaseq_2.fastq.gz: RNA-Seq read 2 data (right read)<br>CTAT_HumanFusionLib.mini.dat.gz: a small fusion annotation library<br>minigenome.fa: small genome sequence consisting of ~750 genes<br>minigenome.gtf: transcript structure annotations for these genes<br><br>I need you to help me plan what needs to be done in each step of the analysis process, and help me write the code for each step to install softwares, use tools and accomplish the following main goal:<br><br>Predict Fusion genes</td></tr>
<tr><td>Generated Results by ChatGPT-4</td><td>To predict fusion genes from RNA-Seq data, we usually perform these steps:<br><br>1. Quality Control of Raw Reads<br>2. Fusion Gene Prediction<br><br>### Step 1: Quality Control of Raw Reads<br><br>FastQC can be used for this:<br><br>```bash<br>sudo apt-get install fastqc<br>fastqc rnaseq_1.fastq.gz rnaseq_2.fastq.gz<br>```<br><br>### Step 2: Fusion Gene Prediction<br><br>Many tools can predict fusion genes, such as STAR-Fusion. We'll use STAR-Fusion in this sample workflow:<br><br>First, install STAR and STAR-Fusion:<br><br>```bash<br># STAR<br>wget https://github.com/alexdobin/STAR/archive/2.7.9a.tar.gz<br>tar -xzf 2.7.9a.tar.gz<br>export PATH=$PATH:`pwd`/STAR-2.7.9a/bin/Linux_x86_64/<br><br># STAR-Fusion<br>wget https://github.com/STAR-Fusion/STAR-Fusion/releases/download/STAR-Fusion_v1.10.0/STAR-Fusion.v1.10.0.tar.gz<br>tar -xzf STAR-Fusion.v1.10.0.tar.gz<br>export PATH=$PATH:`pwd`/STAR-Fusion.v1.10.0/<br>```</td></tr>
</table>

Second, build STAR genome index:

```bash
mkdir STAR_index
STAR --runMode genomeGenerate --genomeDir STAR_index --
genomeFastaFiles minigenome.fa --sjdbGTFfile minigenome.gtf --
runThreadN <Number_of_threads>
```

Replace `<Number_of_threads>` with the number of threads you wish to use.

Finally, run STAR-Fusion:

```bash
STAR --genomeDir STAR_index --readFilesIn rnaseq_1.fastq.gz
rnaseq_2.fastq.gz --outSAMtype BAM SortedByCoordinate --
outReadsUnmapped None --chimSegmentMin 20 --
chimJunctionOverhangMin 20 --alignSJDBoverhangMin 10 --
alignMatesGapMax 200000 --alignIntronMax 200000 --
chimSegmentReadGapMax 3 --alignSJstitchMismatchNmax 5 -1 5 5 --
runThreadN <Number_of_threads> --readFilesCommand zcat --
outFileNamePrefix star

STAR-Fusion --genomeDir STAR_index --out_dir star_fusion_outdir --left_fq
rnaseq_1.fastq.gz --right_fq rnaseq_2.fastq.gz --chimeric_out_junction
starChimeric.out.junction --CPU <Number_of_threads>
```

Replace `<Number_of_threads>` with the number of threads you wish to use.

The predicted fusion genes will be in a file named `star_fusion_outdir/star-fusion.fusion_predictions.tsv`

Please note that fusion identification requires significant computational resources, thus parameters above and tools chosen should best suit your specific data and project.

RNA-Seq Analysis   Case 10.4: isoform expression

**User's Inputs**	You are a bioinformatician who specializes in writing code and performing analysis for a variety of bioinformatic data analysis tasks. Now I provide you with an Ubuntu 18.04 system where I save the following data, each line including the data path and data description.<br><br>SRR14463651_1.fastq.gz: paired end RNA sequencing data from human lymphoma cell, read 1 of lymphoma samples replicate 1<br>SRR14463651_2.fastq.gz: paired end RNA sequencing data from human lymphoma cell, read 2 of lymphoma samples replicate 1<br>SRR14463652_1.fastq.gz: paired end RNA sequencing data from human lymphoma cell, read 1 of lymphoma samples replicate 2<br>SRR14463652_2.fastq.gz: paired end RNA sequencing data from human lymphoma cell, read 2 of lymphoma samples replicate 2<br>SRR14463680_1.fastq.gz: paired end RNA sequencing data from human lymphoma cell, read 1 of control samples replicate 1<br>SRR14463680_2.fastq.gz: paired end RNA sequencing data from human lymphoma cell, read 2 of control samples replicate 1<br>SRR14463681_1.fastq.gz: paired end RNA sequencing data from human lymphoma cell, read 1 of control samples replicate 2<br>SRR14463681_2.fastq.gz: paired end RNA sequencing data from human lymphoma cell, read 2 of control samples replicate 2<br>gencode.v44.transcripts.fa: transcriptome annotation<br>TruSeq3-PE.fa: trimming adapter<br><br>I need you to help me plan what needs to be done in each step of the analysis process, and help me write the code for each step to install softwares, use tools and accomplish the following main goal:<br><br>quantify RNA isoform expression based on transcriptome annotation
**Generated Results by ChatGPT-4**	Quantifying RNA isoform expression from RNA sequencing data can involve the following steps:<br><br>1. Raw Read Quality Control<br>2. Trimming<br>3. Alignment to Reference Transcriptome<br>4. Quantifying Transcripts<br><br>### Step 1: Raw Read Quality Control<br>FastQC can perform quality checks on raw sequence data:<br><br>```bash<br>sudo apt-get install fastqc<br>fastqc *.fastq.gz<br>```<br><br>### Step 2: Trimming<br>Trimmomatic can be used for removing adapters and trimming low quality bases:<br><br>```bash

	sudo apt-get install trimmomatic<br><br>for sampleName in 'SRR14463651_1' 'SRR14463651_2' 'SRR14463652_1' 'SRR14463652_2' 'SRR14463680_1' 'SRR14463680_2' 'SRR14463681_1' 'SRR14463681_2'<br>do<br>java -jar /path_to_trimmomatic/trimmomatic-0.39.jar PE \\<br>${sampleName}_1.fastq.gz \\<br>${sampleName}_2.fastq.gz \\<br>${sampleName}_1_paired.fastq.gz \\<br>${sampleName}_1_unpaired.fastq.gz \\<br>${sampleName}_2_paired.fastq.gz \\<br>${sampleName}_2_unpaired.fastq.gz \\<br>ILLUMINACLIP:TruSeq3-PE.fa:2:30:10 LEADING:3 TRAILING:3 SLIDINGWINDOW:4:15 MINLEN:36<br>done<br>```<br><br>Please replace "/path_to_trimmomatic" with the path where trimmomatic-0.39.jar is located on your system.<br><br>### Step 3: Alignment to Reference Transcriptome<br>Salmon can be used for alignment-based transcript quantification:<br><br>```bash<br># Install salmon<br>conda install -c bioconda salmon<br><br># build Salmon transcript index<br>salmon index -t gencode.v44.transcripts.fa -i gencode.v44.transcripts.index<br><br># process samples for quantification<br>for sampleName in 'SRR14463651' 'SRR14463652' 'SRR14463680' 'SRR14463681'<br>do<br>salmon quant -i gencode.v44.transcripts.index -l A \\<br>-1 ${sampleName}_1_paired.fastq.gz \\<br>-2 ${sampleName}_2_paired.fastq.gz \\<br>-p 8 --validateMappings -o ${sampleName}_quant<br>done<br>```<br><br>### Step 4: Quantifying Transcripts<br>The Salmon output will contain a file `quant.sf` in each *_quant output folder, which contains the transcript abundance estimates. The 'NumReads' column in this file will provide the quantified expressions.<br><br>Remember that downstream analyses may require additional steps such as differential expression analysis, depending on your specific questions and goals.

RNA-seq Analysis **Case 10.5: splicing analysis**

<table>
<tr><td>User's Inputs</td><td>You are a bioinformatician who specializes in writing code and performing analysis for a variety of bioinformatic data analysis tasks. Now I provide you with an Ubuntu 18.04 system where I save the following data, each line including the data path and data description.

SRR14463651_1.fastq.gz: paired end RNA sequencing data from human lymphoma cell, read 1 of lymphoma samples replicate 1
SRR14463651_2.fastq.gz: paired end RNA sequencing data from human lymphoma cell, read 2 of lymphoma samples replicate 1
SRR14463652_1.fastq.gz: paired end RNA sequencing data from human lymphoma cell, read 1 of lymphoma samples replicate 2
SRR14463652_2.fastq.gz: paired end RNA sequencing data from human lymphoma cell, read 2 of lymphoma samples replicate 2
SRR14463680_1.fastq.gz: paired end RNA sequencing data from human lymphoma cell, read 1 of control samples replicate 1
SRR14463680_2.fastq.gz: paired end RNA sequencing data from human lymphoma cell, read 2 of control samples replicate 1
SRR14463681_1.fastq.gz: paired end RNA sequencing data from human lymphoma cell, read 1 of control samples replicate 2
SRR14463681_2.fastq.gz: paired end RNA sequencing data from human lymphoma cell, read 2 of control samples replicate 2
hg38.fa: reference genome
gencode.v38.annotation.gtf: gene annotation
TruSeq3-PE.fa: trimming adapter

I need you to help me plan what needs to be done in each step of the analysis process, and help me write the code for each step to install softwares, use tools and accomplish the following main goal:

identify alternative splicing events in lymphoma and control samples and compare them to get differentially spliced events</td></tr>
<tr><td>Generated Results by ChatGPT-4</td><td>To identify and compare alternative splicing events between your samples, you could follow these steps:

1. Quality Control of Raw Reads
2. Trimming Reads
3. Alignment to the Reference Genome
4. Identify Alternative Splicing Events
5. Differentially Spliced Events Analysis

Here is how you perform each step:

Step 1: Quality Control of Raw Reads
FastQC provides quality checks on raw sequence data.

```bash
sudo apt-get install fastqc
fastqc *.fastq.gz
```

Step 2: Trimming Reads</td></tr>
</table>

You can use Trimmomatic to remove adapter sequences from reads.

```bash
sudo apt-get install trimmomatic

for sampleName in 'SRR14463651' 'SRR14463652' 'SRR14463680'
'SRR14463681'
do
    java -jar /path_to_trimmomatic/trimmomatic-0.39.jar PE \
        ${sampleName}_1.fastq.gz \
        ${sampleName}_2.fastq.gz \
        ${sampleName}_1_paired_trimmed.fastq.gz \
        ${sampleName}_1_unpaired_trimmed.fastq.gz \
        ${sampleName}_2_paired_trimmed.fastq.gz \
        ${sampleName}_2_unpaired_trimmed.fastq.gz \
        ILLUMINACLIP:TruSeq3-PE.fa:2:30:10 LEADING:3 TRAILING:3
MINLEN:36
done
```

Step 3: Alignment to the Reference Genome
Use STAR aligner to map reads to the reference genome.

```bash
wget https://github.com/alexdobin/STAR/archive/2.7.9a.tar.gz
tar -xzf 2.7.9a.tar.gz
export PATH=$PATH:`pwd`/STAR-2.7.9a/bin/Linux_x86_64/

mkdir STAR_index
STAR --runMode genomeGenerate --genomeDir STAR_index --
genomeFastaFiles hg38.fa --sjdbGTFfile gencode.v38.annotation.gtf --
runThreadN 4

for sampleName in 'SRR14463651' 'SRR14463652' 'SRR14463680'
'SRR14463681'
do
    STAR --genomeDir STAR_index --readFilesIn
${sampleName}_1_paired_trimmed.fastq.gz
${sampleName}_2_paired_trimmed.fastq.gz --runThreadN 4 --outSAMtype
BAM SortedByCoordinate --outFileNamePrefix ${sampleName}_
done
```
Adjust `--runThreadN` according to your available processor cores.

Step 4: Identify Alternative Splicing Events
Use rMATS for this, which is an automatic tool to detect alternative splicing events.

```bash
# Docker might be the easiest way to install rMATS
docker pull computomics/rmats:4.1.0

# Run rMATS with docker
```

	docker run -v $(pwd):/data computomics/rmats:4.1.0 rmats.py --b1 SRR14463651_.Aligned.sortedByCoord.out.bam,SRR14463652_.Aligned.sortedByCoord.out.bam --b2 SRR14463680_.Aligned.sortedByCoord.out.bam,SRR14463681_.Aligned.sortedByCoord.out.bam --gtf gencode.v38.annotation.gtf --od /data/rmats_out --tmp /data/tmp -t paired --readLength 50<br>```<br><br>### Step 5: Differentially Spliced Events Analysis<br>rMATS also produces files for each splicing event type with inclusion levels (`*.MATS.JunctionCountOnly.txt`).<br><br>It may benefit you to perform statistical analysis to find differentially spliced events. Due to the complexity, this can be done in a programming environment such as R, using the statistical methodology appropriate for your experimental design.<br><br>Please notice that the above code might need some adjustments according to your computational environment settings and the specific parameters of your samples. Always ensure to validate at each step and adjust parameters when necessary.

RNA-seq Analysis   Case 10.6: APA analysis

**User's Inputs**	You are a bioinformatician who specializes in writing code and performing analysis for a variety of bioinformatic data analysis tasks. Now I provide you with an Ubuntu 18.04 system where I save the following data, each line including the data path and data description.<br><br>SRR14463651_1.fastq.gz: paired end RNA sequencing data from human lymphoma cell, read 1 of lymphoma samples replicate 1<br>SRR14463651_2.fastq.gz: paired end RNA sequencing data from human lymphoma cell, read 2 of lymphoma samples replicate 1<br>SRR14463652_1.fastq.gz: paired end RNA sequencing data from human lymphoma cell, read 1 of lymphoma samples replicate 2<br>SRR14463652_2.fastq.gz: paired end RNA sequencing data from human lymphoma cell, read 2 of lymphoma samples replicate 2<br>SRR14463680_1.fastq.gz: paired end RNA sequencing data from human lymphoma cell, read 1 of control samples replicate 1<br>SRR14463680_2.fastq.gz: paired end RNA sequencing data from human lymphoma cell, read 2 of control samples replicate 1<br>SRR14463681_1.fastq.gz: paired end RNA sequencing data from human lymphoma cell, read 1 of control samples replicate 2<br>SRR14463681_2.fastq.gz: paired end RNA sequencing data from human lymphoma cell, read 2 of control samples replicate 2<br>hg38.fa: reference genome<br>gencode.v38.annotation.gtf: gene annotation<br>TruSeq3-PE.fa: trimming adapter<br><br>I need you to help me plan what needs to be done in each step of the analysis process, and help me write the code for each step to install softwares, use tools and accomplish the following main goal:<br><br>identify and quantify alternative polyadenylation between lymphoma and control samples
**Generated Results by ChatGPT-4**	To identify and quantify alternative polyadenylation between different sample sets from RNA-seq data, you can follow these steps:<br><br>1. Raw Read Quality Control (FastQC)<br>2. Trimming (Trimmomatic)<br>3. Alignment to Reference Genome (STAR)<br>4. Identification and Quantification of Polyadenylation Sites (QAPA or DaPars)<br><br>Please note that the quantification of alternative polyadenylation events is a specialized analysis task and requires specific tools like APAtrap, DaPars, or QAPA. In this guide, we will use DaPars, a Python-based tool:<br><br>### Step 1: Raw Read Quality Control<br>FastQC provides quality checks on raw sequence data.<br><br>```bash<br>sudo apt-get install fastqc<br>fastqc *.fastq.gz<br>```

Step 2: Trimming
To remove the adapter sequences, use Trimmomatic.

```bash
sudo apt-get install trimmomatic

for sampleName in 'SRR14463651' 'SRR14463652' 'SRR14463680'
'SRR14463681'
do
    java -jar /path_to_trimmomatic/trimmomatic-0.39.jar PE \
    ${sampleName}_1.fastq.gz \
    ${sampleName}_2.fastq.gz \
    ${sampleName}_1_trimmed.fastq.gz
${sampleName}_1_unpaired.fastq.gz \
    ${sampleName}_2_trimmed.fastq.gz
${sampleName}_2_unpaired.fastq.gz \
    ILLUMINACLIP:TruSeq3-PE.fa:2:30:10 SLIDINGWINDOW:4:20
MINLEN:25
done
```

Step 3: Alignment to Reference Genome
Align your cleaned reads to the reference genome using STAR with included gene annotations.

```bash
# Download and install STAR
wget https://github.com/alexdobin/STAR/archive/2.7.9a.tar.gz
tar -xzf 2.7.9a.tar.gz
sudo cp STAR-2.7.9a/bin/Linux_x86_64/STAR /usr/local/bin

# Generate STAR genome index
mkdir STAR_index
STAR --runMode genomeGenerate --genomeDir STAR_index --
genomeFastaFiles hg38.fa --sjdbGTFfile gencode.v38.annotation.gtf --
runThreadN <Number_of_threads>

# Align paired end reads
for sampleName in 'SRR14463651' 'SRR14463652' 'SRR14463680'
'SRR14463681'
do
    STAR --genomeDir STAR_index \
    --readFilesIn ${sampleName}_1_trimmed.fastq.gz
${sampleName}_2_trimmed.fastq.gz \
    --runThreadN <Number_of_threads> --alignIntronMax 500000 \
    --outSAMtype BAM SortedByCoordinate --readFilesCommand zcat \
    --outFileNamePrefix ${sampleName}_
done
```

Replace `<Number_of_threads>` with the number of threads you wish to use.

Step 4: Identification and Quantification of Polyadenylation Sites
This can be done using DaPars, a Python-based package that identifies and quantifies alternative polyadenylation (APA) from standard RNA-seq data.

First, you will install DaPars:

```bash
pip install Cython numpy
pip install DaPars
```

Then, you can run DaPars to detect APA events. You will have to make a configuration file for your samples. An example of the configuration file could look something like this:

```bash
[Files]
hg38.Reference_gene_model = gencode.v38.annotation.gtf
hg38.chrom_size = hg38.genome.fa.fai

[Options]
Num_least_in_group1 = 2
Num_least_in_group2 = 2
Coverage_cutoff = 30
FDR_cutoff = 0.05
PDUI_cutoff = 0.2

[Samples]
Control = control_samples_replicate1.bed, control_samples_replicate2.bed
Lymphoma = lymphoma_samples_replicate1.bed,
lymphoma_samples_replicate2.bed
```

Once the configuration file is set up, you can then run DaPars:

```bash
DaPars_main.py example_configure_file.txt
```

This will output the differential polyadenylation sites between Lymphoma and control samples.

Please replace "example_configure_file.txt" with your actual configuration file path. In addition, remember to adjust parameters based on your specific data and project needs.

RNA-seq Analysis **Case 10.7: RNA editing**

User's Inputs

You are a bioinformatician who specializes in writing code and performing analysis for a variety of bioinformatic data analysis tasks. Now I provide you with an Ubuntu 18.04 system where I save the following data, each line including the data path and data description.

SRR14463651_1.fastq.gz: paired end RNA sequencing data from human lymphoma cell, read 1 of lymphoma samples replicate 1
SRR14463651_2.fastq.gz: paired end RNA sequencing data from human lymphoma cell, read 2 of lymphoma samples replicate 1
SRR14463652_1.fastq.gz: paired end RNA sequencing data from human lymphoma cell, read 1 of lymphoma samples replicate 2
SRR14463652_2.fastq.gz: paired end RNA sequencing data from human lymphoma cell, read 2 of lymphoma samples replicate 2
SRR14463680_1.fastq.gz: paired end RNA sequencing data from human lymphoma cell, read 1 of control samples replicate 1
SRR14463680_2.fastq.gz: paired end RNA sequencing data from human lymphoma cell, read 2 of control samples replicate 1
SRR14463681_1.fastq.gz: paired end RNA sequencing data from human lymphoma cell, read 1 of control samples replicate 2
SRR14463681_2.fastq.gz: paired end RNA sequencing data from human lymphoma cell, read 2 of control samples replicate 2
hg38.fa: reference genome
gencode.v38.annotation.gtf: gene annotation
TruSeq3-PE.fa: trimming adapter

I need you to help me plan what needs to be done in each step of the analysis process, and help me write the code for each step to install softwares, use tools and accomplish the following main goal:

identifying RNA edting events in lymphoma and control samples

Generated Results by ChatGPT-4

Identifying RNA editing events from RNA-seq data involves several steps:

1. Raw Read Quality Control
2. Trimming of Reads
3. Alignment to Reference Genome
4. Variant Calling
5. Identification of RNA Editing Sites

Step 1: Raw Read Quality Control

To check the quality of your raw reads sequence data, use FastQC.

```bash
sudo apt-get install fastqc
fastqc *.fastq.gz
```

Step 2: Trimming of Reads

You can use Trim Galore, a wrapper tool around Cutadapt and FastQC, to remove adapter sequences and perform quality trimming from high-throughput sequence data.

```bash
# First, install Trim Galore
sudo apt-get install trim-galore

for sampleName in 'SRR14463651' 'SRR14463652' 'SRR14463680' 'SRR14463681'
do
    trim_galore --paired ${sampleName}_1.fastq.gz ${sampleName}_2.fastq.gz
done
```

Step 3: Alignment to Reference Genome

For alignment, you will use a splice-aware aligner such as STAR.

```bash
# Install STAR
sudo apt-get install star

# Generate STAR genome index
mkdir STAR_index
STAR --runMode genomeGenerate --outTmpDir STAR_index --genomeFastaFiles hg38.fa --sjdbGTFfile gencode.v38.annotation.gtf

# Alignment using STAR
for sampleName in 'SRR14463651' 'SRR14463652' 'SRR14463680' 'SRR14463681'
do
    STAR --genomeDir STAR_index --readFilesIn ${sampleName}_1_val_1.fq.gz ${sampleName}_2_val_2.fq.gz --outFileNamePrefix ${sampleName}_trimmed --outSAMtype BAM SortedByCoordinate
done
```

Step 4: Variant Calling

You will use this sorted Bam file for variant calling. An appropriate tool is GATK's HaplotypeCaller.

```bash
# Install necessary tools
sudo apt-get install samtools
conda install -c bioconda gatk4

# Convert BAM to SAM and generate vcf files
for sampleName in 'SRR14463651' 'SRR14463652' 'SRR14463680' 'SRR14463681'
```

```
do
    samtools view -h
${sampleName}_trimmedAligned.sortedByCoord.out.bam >
${sampleName}_trimmedAligned.sortedByCoord.out.sam
    gatk HaplotypeCaller -R hg38.fa -I
${sampleName}_trimmedAligned.sortedByCoord.out.bam -O
${sampleName}.vcf
done
```

Step 5: Identification of RNA Editing Sites

You can use a tool called "REDItools" to identify the RNA editing sites.

```bash
# Install REDItools
conda install -c bioconda reditools

# Analyze .vcf files to recognize RNA editing events
for sampleName in 'SRR14463651' 'SRR14463652' 'SRR14463680'
'SRR14463681'
do
    REDItoolDnaRna.py -i
${sampleName}_trimmedAligned.sortedByCoord.out.bam -f hg38.fa -o
${sampleName}_RNA_editing_events.txt -t -e -d 5 -l 20 -D
${sampleName}.vcf
done
```

This will carry out the analysis and create files with the identified RNA editing sites. Ensure to explore more criteria and filters that suit the demands of your particular project using these tools. Note that RNA-seq based methods have limited capacity to distinguish RNA-editing events from genomic DNA mutations and aligner artifacts; hence orthogonal validations are needed for critical findings.

RNA-seq Analysis **Case 10.8: circular RNA analysis**

<table>
<tr><td>User's Inputs</td><td>You are a bioinformatician who specializes in writing code and performing analysis for a variety of bioinformatic data analysis tasks. Now I provide you with an Ubuntu 18.04 system where I save the following data, each line including the data path and data description.

SRR14463651_1.fastq.gz: paired end RNA sequencing data from human lymphoma cell, read 1 of lymphoma samples replicate 1
SRR14463651_2.fastq.gz: paired end RNA sequencing data from human lymphoma cell, read 2 of lymphoma samples replicate 1
SRR14463652_1.fastq.gz: paired end RNA sequencing data from human lymphoma cell, read 1 of lymphoma samples replicate 2
SRR14463652_2.fastq.gz: paired end RNA sequencing data from human lymphoma cell, read 2 of lymphoma samples replicate 2
SRR14463680_1.fastq.gz: paired end RNA sequencing data from human lymphoma cell, read 1 of control samples replicate 1
SRR14463680_2.fastq.gz: paired end RNA sequencing data from human lymphoma cell, read 2 of control samples replicate 1
SRR14463681_1.fastq.gz: paired end RNA sequencing data from human lymphoma cell, read 1 of control samples replicate 2
SRR14463681_2.fastq.gz: paired end RNA sequencing data from human lymphoma cell, read 2 of control samples replicate 2
hg38.fa: reference genome
gencode.v38.annotation.gtf: gene annotation
TruSeq3-PE.fa: trimming adapter

I need you to help me plan what needs to be done in each step of the analysis process, and help me write the code for each step to install softwares, use tools and accomplish the following main goal:

identify and quantify circular RNA in lymphoma and control samples</td></tr>
<tr><td>Generated Results by ChatGPT-4</td><td>Here are the steps you need to follow to identify and quantify circular RNAs (circRNAs) from your RNA-seq data:

1. Quality Control of Raw Reads
2. Trimming/Filtering of Sequences
3. Alignment to the Reference Genome
4. Circular RNA Prediction
5. Quantification of Circular RNAs

Step 1: Quality Control of Raw Reads

FastQC is widely used for quality control checks on raw sequence data.

```bash
sudo apt-get install fastqc
fastqc *.fastq.gz
```

Step 2: Trimming/Filtering of Sequences

Trimmomatic can be used to trim the adapters and low-quality ends:</td></tr>
</table>

```bash
sudo apt-get install trimmomatic

for sampleName in 'SRR14463651' 'SRR14463652' 'SRR14463680'
'SRR14463681'
do
    java -jar /path_to_trimmomatic/trimmomatic-0.39.jar PE \
        ${sampleName}_1.fastq.gz \
        ${sampleName}_2.fastq.gz \
        ${sampleName}_1_paired.fastq.gz \
        ${sampleName}_1_unpaired.fastq.gz \
        ${sampleName}_2_paired.fastq.gz \
        ${sampleName}_2_unpaired.fastq.gz \
        ILLUMINACLIP:TruSeq3-PE.fa:2:30:10 SLIDINGWINDOW:4:15
MINLEN:36
done
```

Step 3: Alignment to the Reference Genome

Star is commonly used for reporting alignments suited for circRNA detection. It generates Chimeric.out.junction.out file that contains the alignment info of potential backspliced junction reads.

```bash
# Download, extract, and move STAR to PATH
wget https://github.com/alexdobin/STAR/archive/2.7.9a.tar.gz
tar -xzf 2.7.9a.tar.gz
export PATH=$PATH:`pwd`/STAR-2.7.9a/bin/Linux_x86_64/

# Generate STAR genome index
mkdir STAR_index
STAR --runMode genomeGenerate --genomeDir STAR_index --
genomeFastaFiles hg38.fa --sjdbGTFfile gencode.v38.annotation.gtf --
runThreadN <Number_of_threads>

# Align reads using STAR
for sampleName in 'SRR14463651' 'SRR14463652' 'SRR14463680'
'SRR14463681'
do
    STAR --genomeDir STAR_index --readFilesIn
${sampleName}_1_paired.fastq.gz ${sampleName}_2_paired.fastq.gz --
outFileNamePrefix ${sampleName}_ --chimOutType WithinBAM --
chimSegmentMin 20 --chimJunctionOverhangMin 15 --runThreadN
<Number_of_threads>
done
```

Replace `<Number_of_threads>` with the number of threads you wish to use.

Step 4: Circular RNA Prediction

CIRCexplorer2 is a robust and efficient tool for circular RNA prediction:

```bash
# Install CIRCexplorer2
sudo apt-get install python-dev python-pip
sudo pip install CIRCexplorer2

# Run CIRCexplorer2
for sampleName in 'SRR14463651' 'SRR14463652' 'SRR14463680'
'SRR14463681'
do
   CIRCexplorer2 parse -t STAR ${sampleName}_Chimeric.out.junction >
${sampleName}_Chimeric.out.junction.ce2
   CIRCexplorer2 annotate -r hg38_ref.txt -g hg38.fa -b
${sampleName}_back_spliced_junction.bed -j
${sampleName}_Chimeric.out.junction.ce2
done
```

You should obtain a back_spliced_junction.bed file that includes all detected circRNAs.

Step 5: Quantification of Circular RNAs

CIRCexplorer2 also provides quantification function based on back-splicing reads:

```bash
for sampleName in 'SRR14463651' 'SRR14463652' 'SRR14463680'
'SRR14463681'
do
   CIRCexplorer2 count -f STAR -r circRNA_known.txt -b
${sampleName}_back_spliced_junction.bed -j
${sampleName}_Chimeric.out.junction >
${sampleName}_circRNA_count.txt
done
```

This will generate a *_circRNA_count.txt file for each sample, which contains circRNA quantification results.

Note that each analysis might require further manual check and verification, and fine-tuning of parameters is often necessary to attain optimal results.

Small RNA-seq Analysis **Case 11.1 and Case 11.2: miRNA analysis**

**User's Inputs**	You are a bioinformatician who specializes in writing code and performing analysis for a variety of bioinformatic data analysis tasks. Now I provide you with an Ubuntu 18.04 system where I save the following data, each line including the data path and data description.<br><br>SRR10915820.fastq.gz: single end small RNA sequencing data from human cell line, control samples replicate 1<br>SRR10915821.fastq.gz: single end small RNA sequencing data from human cell line, control samples replicate 1<br>SRR10915822.fastq.gz: single end small RNA sequencing data from human cell line, control samples replicate 1<br>SRR10915823.fastq.gz: single end small RNA sequencing data from human cell line, control samples replicate 1<br>hsa.gff3: microRNA genomic coordinate from miRbase<br>TGGAATTCTCGGGTGCCAAGG: adaptor sequence<br>hg38.fa: the reference genome<br><br>I need you to help me plan what needs to be done in each step of the analysis process, and help me write the code for each step to install softwares, use tools and accomplish the following main goal:<br><br>quantify microRNA expression based on miRbase and predict novel microRNA
**Generated Results by ChatGPT-4**	Identification and quantification of known microRNAs (miRNAs), as well as prediction of novel miRNAs, typically involve the following steps:<br><br>1. Quality Control of Raw Reads<br>2. Adapter Trimming<br>3. Alignment to Reference Genome<br>4. Quantification of Known miRNAs<br>5. Prediction of Novel miRNAs.<br><br>Before you start, make sure you install necessary tools like fastQC, cutadapt, and miRDeep2. miRDeep2 is a comprehensive toolset serving both of our last steps.<br><br>Here are the detailed steps:<br><br>### Step 1: Quality Control of Raw Reads<br><br>FastQC can be used to check the quality of your raw fastq data.<br><br>```bash<br>sudo apt-get install fastqc<br>fastqc *.fastq.gz<br>```<br><br>### Step 2: Adapter Trimming<br><br>Cutadapt can be used to trim adapter sequences.

```bash
# Install Cutadapt
sudo apt-get install cutadapt

# Trim 3' adapter
for sampleName in 'SRR10915820' 'SRR10915821' 'SRR10915822' 'SRR10915823'
do
    cutadapt -a TGGAATTCTCGGGTGCCAAGG -m 18 -o ${sampleName}_trimmed.fastq.gz ${sampleName}.fastq.gz
done
```

Step 3: Alignment to the Reference Genome

For the alignment, the mapper.pl script of miRDeep2 suite can be used.

```bash
# Assuming miRDeep2 is installed and its path included in PATH environment variable

# Convert fastq to fasta as needed by miRDeep2 mapper.pl script
for sampleName in 'SRR10915820' 'SRR10915821' 'SRR10915822' 'SRR10915823'
do
    mapper.pl ${sampleName}_trimmed.fastq.gz -e -h -j -l 18 -m -s ${sampleName}_reads.fa -t ${sampleName}_mapping.arf -v -n -i -p hg38.fa
done
```
This will create both collapsed reads suitable for miRDeep2 and .arf alignment files, containing information about each mapped read.

Step 4: Quantification of Known miRNAs

miRDeep2 comes with a quantifier script that can be used to quantify miRNAs:

```bash
# Run miRDeep2 quantifier.pl
for sampleName in 'SRR10915820' 'SRR10915821' 'SRR10915822' 'SRR10915823'
do
    quantifier.pl -p hsa.gff3 -r ${sampleName}_reads.fa -m ${sampleName}_mapping.arf -t hsa
done
```
It takes a file with the genomic positions of known miRNAs and outputs the number of reads mapping to each miRNA.

Step 5: Prediction of Novel miRNAs

For the prediction of novel miRNAs, the miRDeep2.pl script can be used. Here we are running it on the first file, adjust accordingly for other files:

```bash
# Run miRDeep2.pl
miRDeep2.pl SRR10915820_reads.fa hg38.fa SRR10915820_mapping.arf none none SRR10915820_miRNAs 2> report.log
```
This generates a number of output files including SRR10915820_miRNAs.html, which contains the main results of the miRDeep2.pl run. It also lists potential novel miRNAs.

Please remember to adjust the parameters of each command to best suit your specific data and project requirements.

CAGE-seq Analysis Case 12.1: TSS analysis

**User's Inputs**	You are a bioinformatician who specializes in writing code and performing analysis for a variety of bioinformatic data analysis tasks. Now I provide you with an Ubuntu 18.04 system where I save the following data, each line including the data path and data description.<br><br>SRR11351697.fastq.gz: CAGE-seq data from human cell line, control samples replicate 1<br>SRR11351698.fastq.gz: CAGE-seq data from human cell line, control samples replicate 2<br>SRR11351700.fastq.gz: CAGE-seq data from human cell line, treatment samples replicate 1<br>SRR11351701.fastq.gz: CAGE-seq data from human cell line, treatment samples replicate 1<br>hg38.fa: the human reference genome<br>gencode.v37.annotation.gtf: gene annotation<br>TruSeq3-SE.fa: trimming adapter<br><br>I need you to help me plan what needs to be done in each step of the analysis process, and help me write the code for each step to install softwares, use tools and accomplish the following main goal:<br><br>Identify transcription start site (TSS) in the cell line based on CAGE-seq data
**Generated Results by ChatGPT-4**	The basic processing pipeline for your CAGE-Seq data to identify transcription start sites (TSS), is as follows:<br><br>1. Quality Control (QC) of Raw Reads<br>2. Trimming of Adapters and Low-Quality Bases<br>3. Alignment to the Reference Genome<br>4. TSS Identification<br><br>### Step 1: Quality Control (QC) of Raw Reads<br>You can use FastQC to perform Quality Control checks on raw sequence data.<br><br>```bash<br>sudo apt-get install fastqc<br>fastqc *.fastq.gz<br>```<br><br>### Step 2: Trimming of Adapters and Low-Quality Bases<br>Cutadapt can be used to trim the adapters and low-quality bases.<br><br>```bash<br># Install Cutadapt<br>pip install --user --upgrade cutadapt<br><br>for FILE in SRR11351697 SRR11351698 SRR11351700 SRR11351701<br>do

    cutadapt -a file:TruSeq3-SE.fa -o ${FILE}_trimmed.fastq.gz
${FILE}.fastq.gz
done
```

### Step 3: Alignment to the Reference Genome
You would then use an aligner such as HISAT2, to map your reads to the reference genome:

```bash
# Download, extract and install HISAT2
wget https://cloud.biohpc.swmed.edu/index.php/s/hisat2-220-
source/download -O hisat2-2.2.0-source.zip
unzip hisat2-2.2.0-source.zip
cd hisat2-2.2.0
make
export PATH=$PATH:`pwd`

# Build the genome index
hisat2-build hg38.fa hg38_index

# Alignment
for FILE in SRR11351697 SRR11351698 SRR11351700 SRR11351701
do
    hisat2 -x hg38_index -U ${FILE}_trimmed.fastq.gz -S ${FILE}.sam
done
```

Step 4: TSS Identification
Programs like Tfit (from CAGEr package in R) or HOMER have been used to predict TSSs from CAGE-Seq data. However these are not command line and for simplicity, let's use `paraclu`, which performs clustering to identify TSSs.

```bash
# Install BEDTools for BAM to BED conversion
sudo apt-get install bedtools

# Convert SAM to BED format
for FILE in SRR11351697 SRR11351698 SRR11351700 SRR11351701
do
    bedtools bamtobed -i ${FILE}.sam > ${FILE}.bed
done

# Download and compile paraclu
wget https://github.com/Kingsford-
Group/paraclu/archive/refs/heads/master.zip -O paraclu.zip
unzip paraclu.zip
cd paraclu-master
make
cp paraclu /usr/local/bin/

# Run paraclu

<table>
<tr><td></td><td>for FILE in SRR11351697 SRR11351698 SRR11351700 SRR11351701<br>do<br>    sort -k1,1 -k2,2n ${FILE}.bed | paraclu 10 > ${FILE}_clusters.bed<br>done<br>```<br>This might be enough to give you the clusters of transcription start sites. Remember that you'll need to adjust this pipeline to assemble replicates and treatments, filter clusters by length and read count, and adjust parameters to suit your specific project. Use a genome browser like IGV to visualize clusters makes the interpretation much easier.</td></tr>
</table>

**3’end-seq Analysis  Case 13.1: APA analysis based on 3’end-seq data**

| | |
|---|---|
| **User’s Inputs** | You are a bioinformatician who specializes in writing code and performing analysis for a variety of bioinformatic data analysis tasks. Now I provide you with an Ubuntu 18.04 system where I save the following data, each line including the data path and data description.<br><br>SRR17422754.fastq.gz: Lexongen QuantSeq 3 prime-end RNA sequencing data from human cell line THLE2, healthy liver cell line replicate 1<br>SRR17422755.fastq.gz: Lexongen QuantSeq 3 prime-end RNA sequencing data from human cell line THLE2, healthy liver cell line replicate 2<br>SRR17422756.fastq.gz: Lexongen QuantSeq 3 prime-end RNA sequencing data from human cell line SNU398, liver cancer cell line replicate 1<br>SRR17422757.fastq.gz: Lexongen QuantSeq 3 prime-end RNA sequencing data from human cell line SNU398, liver cancer cell line replicate 2<br>hg38.fa: the human reference genome<br>gencode.v37.annotation.gtf: gene annotation<br>TruSeq3-SE.fa: trimming adapter<br><br>I need you to help me plan what needs to be done in each step of the analysis process, and help me write the code for each step to install softwares, use tools and accomplish the following main goal:<br><br>Identify polyadenylation sites and quantify alternative polyadenylation based on 3'-end sequencing data |
| **Generated Results by ChatGPT-4** | Analysis of 3'-end sequencing data, such as Lexogen QuantSeq, typically involves the following steps:<br><br>1. Quality Control of Raw Reads<br>2. Adapter Trimming<br>3. Alignment to the Reference Genome<br>4. Identification and Quantification of Polyadenylation Sites<br><br>To achieve your goal, you will especially need a tool or strategy to detect poly(A) sites. Here, we can use a dedicated tool such as FLAIR (Full-Length Alternative Isoform analysis of RNA) for poly(A) site identification.<br><br>### Step 1: Quality Control of Raw Reads<br>FastQC can be used to check the quality of your raw Fastq data.<br><br>```bash<br>sudo apt-get install fastqc<br>fastqc *.fastq.gz<br>```<br><br>### Step 2: Adapter Trimming<br>The reads can be trimmed using Trim Galore!:<br><br>```bash<br># First, install Trim Galore!<br>sudo apt-get install trim-galore |

for sampleName in 'SRR17422754' 'SRR17422755' 'SRR17422756' 'SRR17422757'
do
    trim_galore -a TruSeq3-SE.fa -o ${sampleName}_trimmed.fastq.gz ${sampleName}.fastq.gz
done
```

Step 3: Alignment to the Reference Genome
For alignment, using a splice-aware aligner such as STAR is recommended:

```bash
# Download, extract, and move STAR to PATH
wget https://github.com/alexdobin/STAR/archive/2.7.9a.tar.gz
tar -xzf 2.7.9a.tar.gz
export PATH=$PATH:`pwd`/STAR-2.7.9a/bin/Linux_x86_64/

# Generate STAR genome index
STAR --runMode genomeGenerate --genomeDir STAR_index --genomeFastaFiles hg38.fa --sjdbGTFfile gencode.v37.annotation.gtf --runThreadN <Number_of_threads>

# Align using STAR
for sampleName in 'SRR17422754' 'SRR17422755' 'SRR17422756' 'SRR17422757'
do
    STAR --genomeDir STAR_index --readFilesIn ${sampleName}_trimmed.fastq.gz --outFileNamePrefix ${sampleName}_
done
```

Step 4: Identification and Quantification of Polyadenylation Sites
FLAIR can be used to identify the poly(A) sites:

```bash
# First, install FLAIR
git clone https://github.com/BrooksLabUCSC/flair
cd flair
python3 setup.py install

# Run FLAIR
for sampleName in 'SRR17422754' 'SRR17422755' 'SRR17422756' 'SRR17422757'
do
    python flair.py align -g hg38.fa -r ${sampleName}.fastq.gz
    python flair.py correct -q ${sampleName}.sam -g hg38.fa
    python flair.py collapse -g hg38.fa -r ${sampleName}.bed
    python flair.py quantify -i ${sampleName}.bed -r hg38.fa -f gencode.v37.annotation.gtf
done
```
```

	Replace `<Number_of_threads>` with the number of threads you wish to allocate.<br><br>These steps will generate a bed file and a tsv file where each mapped read includes information about poly(A) sites and quantifications of alternative polyadenylation. Different poly(A) sites within individual genes can be compared for differential usage. It is important to visualize the results and validate some of the predicted poly(A) sites with existing databases or through experimental validation.

ONT sequencing Analysis   Case 14.1: isoform expression

**User's Inputs**	You are a bioinformatician who specializes in writing code and performing analysis for a variety of bioinformatic data analysis tasks. Now I provide you with an Ubuntu 18.04 system where I save the following data, each line including the data path and data description.<br><br>RHL5569-T1_SAECK_shControl_replicate_1.ont.fastq.gz: ONT sequencing data from human cell line, control samples replicate 1<br>RHL5570-T1_SAECK_shControl_replicate_2.ont.fastq.gz: ONT sequencing data from human cell line, control samples replicate 2<br>RHL5571-T1_SAECK_shPARP4-1_replicate_1.ont.fastq.gz: ONT sequencing data from human cell line, treatment samples replicate 1<br>RHL5572-T1_SAECK_shPARP4-1_replicate_2.ont.fastq.gz: ONT sequencing data from human cell line, treatment samples replicate 2<br>hg38.fa: the human reference genome<br>gencode.v37.annotation.gtf: gene annotation<br><br>I need you to help me plan what needs to be done in each step of the analysis process, and help me write the code for each step to install softwares, use tools and accomplish the following main goal:<br><br>Quantify isoform expression in control and treatment samples
**Generated Results by ChatGPT-4**	Quantification of isoform expression from Oxford Nanopore sequencing data typically requires several steps:<br><br>1. Quality Control of Raw Reads<br>2. Aligning Reads to the Reference Genome<br>3. Isoform Annotation and Quantification<br><br>Before commencing the steps, consider installing necessary tools: MinKNOW for basecalling (if raw data is in fast5 format), pycoQC for quality control, Minimap2 for alignment, and StringTie for isoform annotation and quantification.<br><br>### Step 1: Quality Control of Raw Reads<br><br>Use pycoQC for quality control checks on raw sequence data.<br><br>First, install pycoQC (note that pycoQC requires python >3.6 and pip):<br><br>```bash<br>pip install pycoQC<br>```<br><br>Then, generate a sequencing summary file through MinKNOW or Guppy, which is required by pycoQC.<br><br>Once you have the sequencing summary file (e.g., sequencing_summary.txt), run:<br><br>```bash

pycoQC -f sequencing_summary.txt -o pycoQC_output.html
```

Examine the HTML report to assess sequencing quality.

### Step 2: Aligning Reads to the Reference Genome

Use Minimap2 for aligning sequences to the reference genome. Minimap2 is efficient for long read alignments:

First, install Minimap2:

```bash
conda install -c bioconda minimap2
```

Execute Minimap2 to align reads:

```bash
for sample in 'RHL5569-T1_SAECK_shControl_replicate_1' 'RHL5570-T1_SAECK_shControl_replicate_2' 'RHL5571-T1_SAECK_shPARP4-1_replicate_1' 'RHL5572-T1_SAECK_shPARP4-1_replicate_2'
do
    minimap2 -ax splice -uf --secondary=no -t 8 hg38.fa ${sample}.ont.fastq.gz | samtools sort -o ${sample}.ont.sorted.bam
    samtools index ${sample}.ont.sorted.bam
done
```

Step 3: Isoform Annotation and Quantification

You can use StringTie for isoform annotation and quantification:

First, install StringTie:

```bash
conda install -c bioconda stringtie
```

Use StringTie for isoform expression levels:

```bash
for sample in 'RHL5569-T1_SAECK_shControl_replicate_1' 'RHL5570-T1_SAECK_shControl_replicate_2' 'RHL5571-T1_SAECK_shPARP4-1_replicate_1' 'RHL5572-T1_SAECK_shPARP4-1_replicate_2'
do
    stringtie ${sample}.ont.sorted.bam -o ${sample}.ont.gtf -G gencode.v37.annotation.gtf -A gene_abundances.tsv
done
```

This will provide a GTF file for each sample with estimated transcript abundances. To perform differential expression analysis between control
```

	and treatment samples, you can use the output from this step in conjunction with tools like DESeq2 or edgeR.<br><br>Please adjust the number of threads ("-t 8") in the minimap2 command and other parameters to match the specifications of your system and to fit your specific project requirements.

PacBio iso-seq Analysis **Case 15.1: isoform analysis**

<table>
<tr>
<td>User’s Inputs</td>
<td>You are a bioinformatician who specializes in writing code and performing analysis for a variety of bioinformatic data analysis tasks. Now I provide you with an Ubuntu 18.04 system where I save the following data, each line including the data path and data description.

SRR19552218.fastq.gz: pacbio sequecning data from human cell line, cell line HCT-116
SRR19785215.fastq.gz: pacbio sequecning data from human cell line, cell line HepG2
hg38.fa: the human reference genome
gencode.v37.annotation.gtf: gene annotation

I need you to help me plan what needs to be done in each step of the analysis process, and help me write the code for each step to install softwares, use tools and accomplish the following main goal:

identify novel isoform and quantify isoform expression in two cell lines</td>
</tr>
<tr>
<td>Generated Results by ChatGPT-4</td>
<td>Since you're working with PacBio sequencing data to identify and quantify isoform expression, a suitable analysis pipeline might be:

1. Quality Control of Raw Reads
2. Read Alignment to the Reference Genome
3. Isoform Identification
4. Isoform Quantification

This will require software packages such as `fastqc`, `minimap2`, and `cufflinks`.

Before you start these steps, please install all the necessary tools. Depending on your sequencing technology (PacBio RS II, Sequel, Sequel II), you might need to process raw reads before further analysis.

Step 1: Quality Control of Raw Reads

FastQC can be used to perform quality control checks on raw sequence data.

```bash
sudo apt-get install fastqc
fastqc *.fastq.gz
```

This will generate a QC report for each fastq file, which can be viewed in a web browser.

Step 2: Read Alignment to the Reference Genome

Since you're working with long reads, Minimap2, an alignment tool offering good performance with long sequences, is an appropriate choice.</td>
</tr>
</table>

First, install Minimap2:

```bash
conda install -c bioconda minimap2
conda install -c bioconda samtools  # required for format conversion
```

Then, perform the alignments:

```bash
for sample in 'SRR19552218' 'SRR19785215'; do
    minimap2 -ax splice -uf --secondary=no hg38.fa ${sample}.fastq.gz |
samtools sort -o ${sample}_sorted.bam
    samtools index ${sample}_sorted.bam
done
```

Step 3: Isoform Identification

Cufflinks can be used to assemble individual transcripts from RNA-seq reads that have been aligned to the genome:

```bash
conda install -c bioconda cufflinks  # install Cufflinks

for sample in 'SRR19552218' 'SRR19785215'; do
    cufflinks -p 8 -o ${sample}_cufflinks -G gencode.v37.annotation.gtf
${sample}_sorted.bam
done
```

Step 4: Isoform Quantification

Cufflinks generates a file called `transcripts.gtf`, which you can use for isoform quantification using Cuffquant and Cuffnorm:

```bash
for sample in 'SRR19552218' 'SRR19785215'; do
    cuffquant -p 8 gencode.v37.annotation.gtf ${sample}_sorted.bam -o
${sample}_cuffquant
done

cuffnorm -o cuffnorm_output -L SRR19552218,SRR19785215
gencode.v37.annotation.gtf \
SRR19552218_cuffquant/abundances.cxb \
SRR19785215_cuffquant/abundances.cxb
```

Please adjust the number of threads ("-p 8") in the minimap2 and cufflinks commands as per your system's capabilities.

With some post-processing to filter out lowly-expressed isoforms or ones present in only one of the cell lines, this pipeline should help you achieve

	your research goals. It's also recommended to validate these isoforms using RT-qPCR or other experimental methods.

CLIP-seq Analysis **Case 16.1: CLIP-seq data analysis**

<table>
<tr>
<td>User’s Inputs</td>
<td>You are a bioinformatician who specializes in writing code and performing analysis for a variety of bioinformatic data analysis tasks. Now I provide you with an Ubuntu 18.04 system where I save the following data, each line including the data path and data description.

ENCLB742AYH.fastq.gz: single end reads of eCLIP data from HepG2 cell line, SRSF1 targeted sample replicate 1
ENCLB770EDJ.fastq.gz: single end reads of eCLIP data from HepG2 cell line, SRSF1 targeted sample replicate 2
hg38.fa: the human reference genome
gencode.v37.annotation.gtf: gene annotation

I need you to help me plan what needs to be done in each step of the analysis process, and help me write the code for each step to install softwares, use tools and accomplish the following main goal:

identify SRSF1 protein-RNA crosslink sites based on CLIP sequencing data</td>
</tr>
<tr>
<td>Generated Results by ChatGPT-4</td>
<td>To identify protein-RNA crosslink sites using eCLIP sequencing data, you would typically perform tasks in this order:

1. Quality Control of Raw Reads
2. Adapter Trimming
3. Aligning Reads to the Reference Genome
4. Peak Calling

The final step is crucial to identify the protein-RNA crosslink sites, you can use tools like CLIPper or Piranha.

Before you start, ensure necessary tools like fastqc, cutadapt, STAR, samtools, and CLIPper are installed on your system.

Step 1: Quality Control of Raw Reads

Use fastQC for a preliminary check on your data quality:

```bash
sudo apt-get install fastqc
fastqc ENCLB742AYH.fastq.gz ENCLB770EDJ.fastq.gz
```

Step 2: Adapter Trimming

Use cutadapt to trim the adapters:

```bash
Install Cutadapt
pip install --user --upgrade cutadapt

Trim Adapter (specify the appropriate adapter sequence used in your experiment)
for sampleName in 'ENCLB742AYH' 'ENCLB770EDJ'</td>
</tr>
</table>

```
do
    cutadapt -a "AGATCGGAAGAGC" -o ${sampleName}_trimmed.fastq.gz ${sampleName}.fastq.gz
done
```

Step 3: Aligning Reads to the Reference Genome

Choose an aligner suitable for RNA sequencing data like STAR:

```bash
# Install STAR
# assuming directory with STAR source code is named STAR-2.7.9a
cd STAR-2.7.9a/source
make STAR
export PATH=$PATH:$(pwd)

# Generate genome index
STAR --runThreadN <number_of_threads> --runMode genomeGenerate --genomeDir hg38_STARindex --genomeFastaFiles hg38.fa --sjdbGTFfile gencode.v37.annotation.gtf --sjdbOverhang 100

# Align reads
for sampleName in 'ENCLB742AYH' 'ENCLB770EDJ'
do
    STAR --genomeDir hg38_STARindex --readFilesIn ${sampleName}_trimmed.fastq.gz --outFileNamePrefix {$sampleName} --runThreadN <number_of_threads> --outSAMtype BAM SortedByCoordinate
done
```

Replace `<number_of_threads>` with appropriate thread number.

Step 4: Peak Calling

Peak calling can be performed using the CLIPper software:

```bash
# Assuming that you have CLIPper and its dependencies (scipy, numpy, matplotlib, pybedtools) installed

for sampleName in 'ENCLB742AYH' 'ENCLB770EDJ'
do
    clipper -b ${sampleName}Aligned.sortedByCoord.out.bam -s hg38 -o FileName
done
```

These commands will generate a BED file with peak regions denoting potential protein-RNA crosslink sites. Be sure to check the parameters in each command to suit your specific experiment and data.

RIP-Seq Analysis **Case 16.2: Identify enriched genes bounded by RPB**

**User's Inputs**	You are a bioinformatician who specializes in writing code and performing analysis for a variety of bioinformatic data analysis tasks. Now I provide you with an Ubuntu 18.04 system where I save the following data, each line including the data path and data description.<br><br>rip.rep1.fastq.gz: single end reads of RIP-seq data from HepG2 cell line, pulldown samples of SRSF1 replicate 1<br>rip.rep2.fastq.gz: single end reads of RIP-seq data from HepG2 cell line, pulldown samples of SRSF1 replicate 2<br>controll.rep1.fastq.gz: single end reads of RIP-seq daata from HepG2 cell line, control sample replicate 1<br>controll.rep2.fastq.gz: single end reads of RIP-seq daata from HepG2 cell line, control sample replicate 2<br>hg38.fa: the human reference genome<br>gencode.v37.annotation.gtf: gene annotation<br><br>I need you to help me plan what needs to be done in each step of the analysis process, and help me write the code for each step to install softwares, use tools and accomplish the following main goal:<br><br>identify genes enriched in pulldown samples over the control
**Generated Results by ChatGPT-4**	To determine which genes are enriched in your pulldown samples relative to the control, you'll need to perform a number of bioinformatics analyses. Here's a general step-by-step plan:<br><br>1. Install necessary software.<br><br>2. Quality control of your input data.<br><br>3. Map sequencing reads to the reference genome.<br><br>4. Perform quality control on the mapped data.<br><br>5. Estimate gene expression levels from the mapped reads.<br><br>6. Do differential gene expression analysis to identify genes that are more highly expressed in pulldown samples compared to controls.<br><br>For the installation of the necessary software, we'll use FastQC, Cutadapt, Bowtie2, SAMtools, R, DESeq2, and Python. Here are the commands to install them on Ubuntu:<br><br>```bash<br>sudo apt-get update<br>sudo apt-get install fastqc cutadapt bowtie2 samtools r-base python3-pip<br>pip3 install multiqc<br>sudo R<br>```<br><br>Inside R shell, install the package:

```R
if (!requireNamespace("BiocManager", quietly = TRUE))
    install.packages("BiocManager")
BiocManager::install("DESeq2")
```

To leave the R shell, you can type:

```R
quit()
```

Now, let's break down each step:

1. Quality control of your input data.

Use FastQC and Cutadapt to check and clean your reads.

```bash
fastqc rip.rep1.fastq.gz rip.rep2.fastq.gz control.rep1.fastq.gz
control.rep2.fastq.gz

cutadapt -q 20 -o rip.rep1.trimmed.fastq.gz rip.rep1.fastq.gz
cutadapt -q 20 -o rip.rep2.trimmed.fastq.gz rip.rep2.fastq.gz
cutadapt -q 20 -o control.rep1.trimmed.fastq.gz control.rep1.fastq.gz
cutadapt -q 20 -o control.rep2.trimmed.fastq.gz control.rep2.fastq.gz
```

2. Map trimmed reads to reference genome with Bowtie2.

First, index your reference genome:

```bash
bowtie2-build hg38.fa hg38
```

Next, map the reads:

```bash
bowtie2 -x hg38 -U rip.rep1.trimmed.fastq.gz -S rip.rep1.sam
bowtie2 -x hg38 -U rip.rep2.trimmed.fastq.gz -S rip.rep2.sam
bowtie2 -x hg38 -U control.rep1.trimmed.fastq.gz -S control.rep1.sam
bowtie2 -x hg38 -U control.rep2.trimmed.fastq.gz -S control.rep2.sam
```

3. Quality control on mapped data and conversion to required formats.

You can use SAMtools for this:

```bash
samtools view -S -b rip.rep1.sam > rip.rep1.bam
samtools view -S -b rip.rep2.sam > rip.rep2.bam
```

```
samtools view -S -b control.rep1.sam > control.rep1.bam
samtools view -S -b control.rep2.sam > control.rep2.bam

samtools sort rip.rep1.bam > rip.rep1.sorted.bam
samtools sort rip.rep2.bam > rip.rep2.sorted.bam
samtools sort control.rep1.bam > control.rep1.sorted.bam
samtools sort control.rep2.bam > control.rep2.sorted.bam
```

4. Estimate gene expression levels:

Use featureCounts to quantify gene expression. First, install subread:

```bash
sudo apt-get install subread
```

Then, run featureCounts:

```bash
featureCounts -a gencode.v37.annotation.gtf -o counts.txt
rip.rep1.sorted.bam rip.rep2.sorted.bam control.rep1.sorted.bam
control.rep2.sorted.bam
```

5. Differential Expression Analysis:

At this step, you will use the DESeq2 package in R to carry out differential gene expression analysis.

```R
library(DESeq2)

# Read in data, here 'counts.txt' is result from featureCounts
countdata <- read.csv("counts.txt", sep="\t", row.names=1)
condition <- factor(c("pulldown","pulldown", "control", "control"))
coldata <- DataFrame(condition)
dds <- DESeqDataSetFromMatrix(countData = countdata, colData =
coldata, design = ~ condition)
dds <- DESeq(dds)
res <- results(dds)
res <- res[order(res$padj),]
write.csv(res, file="differential_expression_results.csv")
```

This will provide you with a table of genes and their corresponding log2 fold changes, p-values, and adjusted p-values. Sorting by adjusting p-value (or padj in the R script) ensures we first see genes with strong statistical evidence for differential expression. The genes at the top of this list are those which are differentially expressed between your conditions.

Remember to interpret your results respecting the biological relevance and statistical significance.

	Please note these codes haven't been tested and are just for giving you an idea. Each specific analysis situation may require adjustments and interpretational caution is advised.

Ribo-Seq Analysis **Case 17.1: Identify translated ORFs**

User's Inputs

You are a bioinformatician who specializes in writing code and performing analysis for a variety of bioinformatic data analysis tasks. Now I provide you with an Ubuntu 18.04 system where I save the following data, each line including the data path and data description.

SRR12354645_1.fastq.gz: read 1 of paired-end ribo-seq data, read 1 of control samples replicate 1
SRR12354645_2.fastq.gz: read 2 of paired-end ribo-seq data, read 2 of control samples replicate 1
SRR12354646_1.fastq.gz: read 1 of paired-end ribo-seq data, read 1 of control samples replicate 2
SRR12354646_2.fastq.gz: read 2 of paired-end ribo-seq data, read 2 of control samples replicate 2
hg38.fa: the human reference genome
gencode.v37.annotation.gtf: gene annotation
TruSeq3-PE.fa: trimming adapter

I need you to help me plan what needs to be done in each step of the analysis process, and help me write the code for each step to install softwares, use tools and accomplish the following main goal:

Identify translated ORFs based on ribo-seq data

Generated Results by ChatGPT-4

Translating Ribosome Affinity Purification (Ribo-Seq) data can be used to profile ribosome footprints and, from there, locate translated Open Reading Frames (ORFs). Your analysis will require several steps:

1. Quality check and trim your reads.
2. Map your trimmed reads to the reference genome.
3. Perform footprint profiling.
4. Identify translated ORFs.

Here's an overview of how you may accomplish this:

1. Quality check and trimming

FastQC and Cutadapt can be used here. Install these with the following commands:

```bash
sudo apt-get update
sudo apt-get install fastqc python3-pip
pip3 install cutadapt multiqc
```

Get a quality check on your files:

```bash
fastqc *.fastq.gz
```

Trim adapters using Cutadapt (if necessary):

```bash
cutadapt -a file:TruSeq3-PE.fa -o SRR12354645_1_trimmed.fastq.gz
SRR12354645_1.fastq.gz
cutadapt -a file:TruSeq3-PE.fa -o SRR12354645_2_trimmed.fastq.gz
SRR12354645_2.fastq.gz
cutadapt -a file:TruSeq3-PE.fa -o SRR12354646_1_trimmed.fastq.gz
SRR12354646_1.fastq.gz
cutadapt -a file:TruSeq3-PE.fa -o SRR12354646_2_trimmed.fastq.gz
SRR12354646_2.fastq.gz
```

2. Map your trimmed reads to the reference genome

To map your trimmed reads, you can use HISAT2. First, you need to install it.

```bash
sudo apt-get install hisat2
```

Construct an index using your reference genome:

```bash
hisat2-build hg38.fa hg38
```

Then you map the reads using the built index:

```bash
hisat2 -x hg38 -1 SRR12354645_1_trimmed.fastq.gz -2
SRR12354645_2_trimmed.fastq.gz -S SRR12354645.sam
hisat2 -x hg38 -1 SRR12354646_1_trimmed.fastq.gz -2
SRR12354646_2_trimmed.fastq.gz -S SRR12354646.sam
```

3. Perform footprint profiling

Ribo-seq strives to capture the positions of translating ribosomes on mRNAs. This step involves analyzing the positions of ribosome protected fragments (RPFs) with ribo-seq data. A tool for this can be RiboProfiling, an R package. Start by installing it:

```R
if (!requireNamespace("BiocManager", quietly = TRUE))
   install.packages("BiocManager")
BiocManager::install("RiboProfiling")
```

4. Identify Translated ORFs

	To identify ORFs, we're going to use RiboTaper, a highly effective tool. Unfortunately, RiboTaper does not have a terminal command installer. You can download and install it manually from their website: https://ohlerlab.mdc-berlin.de/software/RiboTaper_147/<br>Once you have RiboTaper installed and your data ready, you will need to prepare your data in the structure that RiboTaper needs for analysis. Following the instructions provided in the README or user guide is suggested.<br><br>The example of these steps is a very generalized workflow. Different analysis scenarios might require adjustments. Check the respective tool manuals for in-depth explanations and possible options. Also, these codes are not tested, so they may require minor adjustments as per your data. Note that identifying ORFs is a complex task and interpretation of the results should be done carefully.

scRNA-Seq Analysis Case 18.1: Cluster cell into groups based on raw fastq files

<table>
<tr><td>User's Inputs</td><td>You are a bioinformatician who specializes in writing code and performing analysis for a variety of bioinformatic data analysis tasks. Now I provide you with an Ubuntu 18.04 system where I save the following data, each line including the data path and data description.

/pbmc_1k_v3_fastqs.tar: the packed fastq files from 10x single cell sequencing
refdata-gex-GRCh38-2020-A: directory contains transcriptome reference

I need you to help me plan what needs to be done in each step of the analysis process, and help me write the code for each step to install softwares, use tools and accomplish the following main goal:

cluster cells into different groups based on 10x single cell sequencing data</td></tr>
<tr><td>Generated Results by ChatGPT-4</td><td>To accomplish your goal, you will need to perform a Single-cell RNA-seq (scRNA-seq) analysis. There are several methods to perform scRNA-seq analysis, here we will use the 10X Genomics Cell Ranger pipeline and R package Seurat. Here's the step-by-step plan:

1. Install necessary software.
2. Extract the fastq files.
3. Run the Cell Ranger pipeline.
4. Post analysis using Seurat.

1. Installing Necessary Software

The major software you will need for this analysis are:

- Cell Ranger: follow the instructions on the 10X Genomics website to download and install Cell Ranger.

- R: you may need to additionally install a suite of packages including "Seurat" for downstream analysis.

You can install R using following command:

```
sudo apt-get install r-base
```

Then you can go into the R shell by just typing `R` in terminal and install Seurat:

```
install.packages('Seurat')
```

Also, the analysis requires pandas Python library, you can install it by:

```
pip3 install pandas</td></tr>
</table>

```

### 2. Extract the Fastq Files

First, decompress fastq files:

```bash
tar -xvf pbmc_1k_v3_fastqs.tar
```

3. Run the Cell Ranger pipeline

Check the path to your transcriptome and correct it if necessary. Then execute the following command:

```bash
cellranger count --id=sample1 \
        --transcriptome=refdata-gex-GRCh38-2020-A \
        --fastqs=path_to_fastq \
        --sample=sample_name \
        --expect-cells=1000
```

Replace "path_to_fastq" and "sample_name" with the appropriate values. The `--expect-cells` option is used to give Cell Ranger a hint about the number of expected cells.

4. Post Analysis Using Seurat

After Cell Ranger completes, you will have a directory (named "sample1" above) that contains several subdirectories. The one we care about is /outs, which contains the file 'filtered_gene_bc_matrices'.

```R
library(Seurat)
# Load the data
matrix_dir = "/path/to/sample1/outs/filtered_feature_bc_matrix"
pbmc.data <- Read10X(data.dir = matrix_dir)
# Initialize the Seurat object with raw (non-normalized data).
pbmc <- CreateSeuratObject(counts = pbmc.data, project = "sample1",
min.cells = 3, min.features = 200)
pbmc[["percent.mt"]] <- PercentageFeatureSet(pbmc, pattern = "^MT-")
# Standard log-normalization
pbmc <- NormalizeData(pbmc, normalization.method = "LogNormalize",
scale.factor = 10000)
pbmc <- FindVariableFeatures(pbmc, selection.method = "vst", nfeatures =
2000)
# Scale the data
pbmc <- ScaleData(pbmc, verbose = FALSE)
pbmc <- RunPCA(pbmc, verbose = FALSE)
pbmc <- FindNeighbors(pbmc, dims = 1:10)
pbmc <- FindClusters(pbmc, resolution = 0.5)
# Run UMAP for visualization

| | |
|---|---|
| | pbmc <- RunUMAP(pbmc, dims = 1:10)<br>DimPlot(pbmc, group.by = "seurat_clusters")<br>```<br><br>The Seurat commands will normalize the data, find variable features, scale data, run PCA, find neighbors, find clusters, and run UMAP for visualization.<br><br>Remember, copy and replace "/path/to/sample1" with your folder path and "sample1" with your sample name.<br><br>This is a basic analysis to cluster your data based on the 10x single cell sequencing data. You can add more steps according to your specific goals. |

**scRNA-Seq Analysis Case 18.2: Clustering and Find the differentially expressed genes**

<table>
<tr><td>User's Inputs</td><td>You are a bioinformatician who specializes in writing code and performing analysis for a variety of bioinformatic data analysis tasks. Now I provide you with an Ubuntu 18.04 system where I save the following data, each line including the data path and data description.<br><br>./examples/case2.1/data/filtered_gene_bc_matrices/hg19: path to 10x mtx data<br><br>I need you to help me plan what needs to be done in each step of the analysis process, and help me write the code for each step to install softwares, use tools and accomplish the following main goal:<br><br>Use scanpy to find the differentially expressed genes</td></tr>
<tr><td>Generated Results by ChatGPT-4</td><td>To identify differentially expressed genes using the Python bioinformatics package "scanpy," you'll need to follow these steps:<br><br>1. Install necessary software.<br>2. Load the data into an AnnData object (scanpy's data format).<br>3. Preprocess your data.<br>4. Identify differentially expressed genes.<br><br>Here's the detailed plan and the accompanying code snippets:<br><br>### 1. Installing Necessary Software<br><br>If you have not yet installed scanpy, you can install it with the following command:<br><br>```bash<br>pip3 install scanpy<br>```<br><br>Additional packages like matplotlib and seaborn for data visualization, and pandas for data manipulation might also be useful. These can be installed similarly:<br><br>```bash<br>pip3 install matplotlib seaborn pandas<br>```<br><br>### 2. Load The Data Into An AnnData Object<br><br>You can load the 10x mtx data into a scanpy AnnData object:<br><br>```python<br>import scanpy as sc<br><br>adata = sc.read_10x_mtx(<br>    './examples/case2.1/data/filtered_gene_bc_matrices/hg19',  # the directory with the `.mtx` file</td></tr>
</table>

    var_names='gene_symbols',                # use gene symbols for the variable names (variables-axis index)
                                             )
```

3. Preprocess Your Data

You'll want to normalize, log-transform your data, and identify highly variable genes:

```python
sc.pp.normalize_total(adata, target_sum=1e4)
sc.pp.log1p(adata)
adata.raw = adata
sc.pp.highly_variable_genes(adata, min_mean=0.0125, max_mean=3, min_disp=0.5)
adata = adata[:, adata.var.highly_variable]
```

4. Identify Differentially Expressed Genes

To identify differentially expressed genes, you will first run a PCA (Principal Component Analysis), construct a neighborhood graph of cells, cluster the graph, and find markers for each cluster:

```python
sc.tl.pca(adata, svd_solver='arpack')
sc.pp.neighbors(adata, n_neighbors=10, n_pcs=40)
sc.tl.leiden(adata)
sc.tl.rank_genes_groups(adata, 'leiden')
```

After these steps, you can view the differentially expressed genes in each cluster:

```python
sc.pl.rank_genes_groups(adata, n_genes=25, sharey=False)
```

The code listed here illustrates the basic steps in a scanpy analysis. Additions and modifications may be needed in order to address the specifics of any given use case. For more detailed walkthroughs, refer to the scanpy tutorials available at: https://scanpy.readthedocs.io/

Please replace `./examples/case2.1/data/filtered_gene_bc_matrices/hg19` with your actual data directory.
```

scRNA-Seq Analysis Case 18.3: Identify Marker Genes

User's Inputs

You are a bioinformatician who specializes in writing code and performing analysis for a variety of bioinformatic data analysis tasks. Now I provide you with an Ubuntu 18.04 system where I save the following data, each line including the data path and data description.

./examples/case2.1/data/filtered_gene_bc_matrices/hg19: path to 10x mtx data

I need you to help me plan what needs to be done in each step of the analysis process, and help me write the code for each step to install softwares, use tools and accomplish the following main goal:

Use scanpy to identify top5 marker genes.

Generated Results by ChatGPT-4

To identify top marker genes using the Python bioinformatics package "scanpy," you'll need to follow these steps:

1. Install necessary software.
2. Load the data into an AnnData object (scanpy's data format).
3. Preprocess your data.
4. Perform clustering
5. Identify marker genes.

Here's the detailed plan and the accompanying code snippets:

1. Installing Necessary Software
If you have not yet installed scanpy, you can install it with the following command:

```bash
pip3 install scanpy
```

2. Load The Data Into An AnnData Object
You can load the 10x mtx data into a scanpy AnnData object:

```python
import scanpy as sc

adata = sc.read_10x_mtx(
    './examples/case2.1/data/filtered_gene_bc_matrices/hg19',  # directory with the .mtx file
    var_names='gene_symbols',  # use gene symbols for the variable names (variables-axis index)
)
```
3. Preprocess Your Data
You'll want to normalize, log-transform your data, and identify highly variable genes:

```python
sc.pp.normalize_total(adata, target_sum=1e4)
```

sc.pp.log1p(adata)
adata.raw = adata
sc.pp.highly_variable_genes(adata, min_mean=0.0125, max_mean=3, min_disp=0.5)
adata = adata[:, adata.var.highly_variable]
```
### 4. Perform Clustering
To perform clustering so you can identify marker genes for each cluster, first run a PCA (Principal Component Analysis), construct a neighborhood graph of cells, and cluster the graph:

```python
sc.tl.pca(adata, svd_solver='arpack')
sc.pp.neighbors(adata, n_neighbors=10, n_pcs=40)
sc.tl.leiden(adata)
```
5. Identify Marker Genes
To identify marker genes, you will find markers for each cluster.

```python
sc.tl.rank_genes_groups(adata, 'leiden')
```

After these steps, the top marker genes can be accessed as follows:

```python
sc.pl.rank_genes_groups(adata, n_genes=5, sharey=False)
```

The code listed here illustrates the basic steps in a scanpy analysis. Additions and modifications may be needed in order to address the specifics of any given use case. For more detailed walkthroughs, refer to the scanpy tutorials available at: https://scanpy.readthedocs.io/

Please replace `./examples/case2.1/data/filtered_gene_bc_matrices/hg19` with your actual data directory.
```

scRNA-Seq Analysis   Case 18.4: Clustering and Visualization

<table>
<tr>
<td>User's Inputs</td>
<td>You are a bioinformatician who specializes in writing code and performing analysis for a variety of bioinformatic data analysis tasks. Now I provide you with an Ubuntu 18.04 system where I save the following data, each line including the data path and data description.

./examples/case2.1/data/filtered_gene_bc_matrices/hg19: path to 10x mtx data

I need you to help me plan what needs to be done in each step of the analysis process, and help me write the code for each step to install softwares, use tools and accomplish the following main goal:

Use scanpy to perform clustering and visualize the expression level of gene PPBP in the UMAP.</td>
</tr>
<tr>
<td>Generated Results by ChatGPT-4</td>
<td>To visualize the expression level of a specific gene across clusters, you would need to follow these steps:

1. Install necessary software.
2. Load the data into an AnnData object (scanpy's data format).
3. Preprocess your data.
4. Perform clustering
5. Visualize gene expression using UMAP.

Here's the detailed plan and the accompanying code snippets:

1. Installing Necessary Software
If you have not yet installed scanpy, you can install it with the following command:

```bash
pip3 install scanpy
```

2. Load The Data Into An AnnData Object
You can load the 10x mtx data into a scanpy AnnData object:

```python
import scanpy as sc

# Correct the path to your data directory
adata = sc.read_10x_mtx(
    './examples/case2.1/data/filtered_gene_bc_matrices/hg19', # directory with the .mtx file
    var_names='gene_symbols', # use gene symbols for the variable names (variables-axis index)
)
```
3. Preprocess Your Data
Normalize, log-transform your data, and identify highly variable genes:

```python</td>
</tr>
</table>

sc.pp.normalize_total(adata, target_sum=1e4)
sc.pp.log1p(adata)
adata.raw = adata
sc.pp.highly_variable_genes(adata, min_mean=0.0125, max_mean=3, min_disp=0.5)
adata = adata[:, adata.var.highly_variable]
```

### 4. Perform Clustering
Perform clustering which will be used for the visualization, first run a PCA (Principal Component Analysis), construct a neighborhood graph of cells, and cluster the graph.

```python
sc.tl.pca(adata, svd_solver='arpack')
sc.pp.neighbors(adata, n_neighbors=10, n_pcs=40)
sc.tl.leiden(adata)
```

5. Visualize gene expression using UMAP.

To visualize the gene expression of "PPBP" across the different clusters, compute a UMAP reduction and then visualize it:

```python
sc.tl.umap(adata)
sc.pl.umap(adata, color='PPBP')
```

Ensure you replace `PPBP` with the gene of interest if it is different or you can add more genes to color by providing a list of genes. Also, replace `'./examples/case2.1/data/filtered_gene_bc_matrices/hg19'` with your actual data directory.

The presented option and steps provide a simplified starting point, further steps might be needed to refine your specific analysis. For more detailed walkthroughs, refer to the scanpy tutorials available at: https://scanpy.readthedocs.io/
```

Spatial Transcriptomics **Case 19.1: Neighborhood enrichment analysis**

User's Inputs

You are a bioinformatician who specializes in writing code and performing analysis for a variety of bioinformatic data analysis tasks. Now I provide you with an Ubuntu 18.04 system where I save the following data, each line including the data path and data description.

slice1.h5ad: spatial transcriptomics data for slice 1 in AnnData format

I need you to help me plan what needs to be done in each step of the analysis process, and help me write the code for each step to install softwares, use tools and accomplish the following main goal:

Use squidpy for neighborhood enrichment analysis

Generated Results by ChatGPT-4

For conducting a neighborhood enrichment analysis using Squidpy, you'll proceed through these stages:

1. Install the necessary software.
2. Load the spatial transcriptomics data.
3. Preprocess the data.
4. Compute a connectivity matrix.
5. Perform neighborhood enrichment analysis.

Here is a detailed breakdown of all the steps, along with code snippets to help you along:

1. Install Squidpy
If Squidpy is not already installed on your system, you'll need to install it. You can use the following command:

```bash
pip3 install squidpy
```
You may also want to install Scanpy because Squidpy is designed to work seamlessly with Scanpy objects.

```bash
pip3 install scanpy
```

2. Load the Spatial Transcriptomics Data
Next, you'll load your spatial transcriptomics data. If it's stored as an h5ad file, you can use the Scanpy read function as follows:

```python
import scanpy as sc
adata = sc.read('slice1.h5ad')
```

3. Preprocess the Data
Data preprocessing can contain different steps including normalization, scaling, and handling spatial coordinates etc. Here I assume that as your

data is already in .h5ad format (AnnData), these preprocessing steps might have been performed already.

If further preprocessing is necessary, refer to Scanpy and Squidpy documentation to perform necessary steps.

4. Compute a connectivity matrix
To perform neighborhood enrichment analysis, you first need to compute a spatial connectivity matrix with the `sq.gr.spatial_neighbors()` function.

```python
import squidpy as sq
sq.gr.spatial_neighbors(adata)
```

5. Perform Neighborhood Enrichment Analysis
Now, with the spatial connectivity matrix computed, you can execute the neighborhood enrichment analysis:

```python
sq.gr.nhood_enrichment(adata, cluster_key='clusters')
```
Replace `'clusters'` with the name of the column in `adata.obs` that contains your cluster annotations.

You can visualize the results with the following command:

```python
sq.pl.nhood_enrichment(adata, cluster_key='clusters')
```

Again, replace `'clusters'` with the name of the column in `adata.obs` that contains your cluster annotations.

Remember to replace `'slice1.h5ad'` with the actual path to your file. Also, Scanpy and Squidpy functions are highly customizable, be sure to read through their documentation to understand all the available options so you can adjust them to suit your specific needs.

This is a very generalized workflow and different analysis scenarios might require adjustments. Check the respective tool manuals for in-depth explanations and possible options.

Spatial Transcriptomics **Case 19.2: Single-cell mapping**

User's Inputs

You are a bioinformatician who specializes in writing code and performing analysis for a variety of bioinformatic data analysis tasks. Now I provide you with an Ubuntu 18.04 system where I save the following data, each line including the data path and data description.

slice1.h5ad: spatial transcriptomics data for slice 1 in AnnData format
sc.h5ad: single cell data in the same tissue for slice 1 in AnnData format

I need you to help me plan what needs to be done in each step of the analysis process, and help me write the code for each step to install softwares, use tools and accomplish the following main goal:

Use Tangram to map single-cell data to spatial transcriptomics data.

Generated Results by ChatGPT-4

Tangram is a Python library that maps single-cell RNA-seq data (scRNA-seq) onto spatially resolved transcriptomics data. These are the steps you would typically follow:

1. Install required software.
2. Load the single-cell and spatial transcriptomics data.
3. Preprocess data for Tangram.
4. Run Tangram mapping.
5. Visualize the mapping.

Here's the step-by-step process along with the code snippets:

1. Install required Software

First, install Tangram by using the following command:

```bash
pip3 install git+https://github.com/broadinstitute/Tangram.git
```

2. Load the single-cell and spatial transcriptomics data

Use Scanpy to load your AnnData format data as follows:

```python
import scanpy as sc

# Correct the path to your data directory
sc_adata = sc.read('sc.h5ad')
sp_adata = sc.read('slice1.h5ad')
```

3. Preprocess data for Tangram

Make sure that the single-cell and spatial data are processed properly before proceeding with Tangram. Mainly, it's important to ensure that the gene names between the two AnnData objects are consistent.

4. Run Tangram mapping

Perform the mapping using Tangram:

```python
import numpy as np
import tangram as tg

# Select source and target genes
source_genes = np.intersect1d(sc_adata.var_names, sp_adata.var_names)
source_adata = sc_adata[:, source_genes]
target_adata = sp_adata[:, source_genes]

# Compute Tangram mapping matrix
mapping_matrix = tg.map_cells_to_space(source_adata, target_adata, device='cpu', n_epochs=500)
```

5. Visualize the mapping

Visualize the mapping of a specific cell type or state:

```python
cell_type = 'cell_type1'  # replace with your relevant cell type.
sc_adata.obs[cell_type] = (sc_adata.obs[cell_type] > 0.5)  # Update as per your condition.
tg.plotting.plot_probabilities(target_adata.obsm['spatial'], mapping_matrix, source_adata, cell_type)
```

Replace `'cell_type1'` and the condition with the cell type or state you want to visualize. And replace 'slice1.h5ad' and 'sc.h5ad' with your actual data file paths.

These codes just provide a basic workflow for mapping scRNA-seq data onto spatial transcriptomics with Tangram, additional preprocessing steps (filtering, normalization, batch effect correction, dimensionality reduction, etc.) might be required for both the spatial and scRNA-seq data. Please consult the respective reference manuals and adjust the procedure to meet your needs.

Proteomics Case 20.1: protein expression quantification

<table>
<tr><td>User’s Inputs</td><td>You are a bioinformatician who specializes in writing code and performing analysis for a variety of bioinformatic data analysis tasks. Now I provide you with an Ubuntu 18.04 system where I save the following data, each line including the data path and data description.

01CPTAC_COprospective_W_PNNL_20170123_B1S1_f01.mzML.gz: protein mass spectrometry data in mzML format, tumor samples fraction 1
01CPTAC_COprospective_W_PNNL_20170123_B1S1_f02.mzML.gz: protein mass spectrometry data in mzML format, tumor samples fraction 2
01CPTAC_COprospective_W_PNNL_20170123_B1S1_f03.mzML.gz: protein mass spectrometry data in mzML format, tumor samples fraction 3
98CPTAC_COprospective_W_VU_20160804_11CO047_f01.mzML.gz: protein mass spectrometry data in mzML format, normal samples fraction 1
98CPTAC_COprospective_W_VU_20160804_11CO047_f02.mzML.gz: protein mass spectrometry data in mzML format, normal samples fraction 2
98CPTAC_COprospective_W_VU_20160804_11CO047_f03.mzML.gz: protein mass spectrometry data in mzML format, normal samples fraction 3
UP000005640_9606.fasta: reference protein sequence from UniProt

I need you to help me plan what needs to be done in each step of the analysis process, and help me write the code for each step to install softwares, use tools and accomplish the following main goal:

identify proteins in tumor and normal samples based on mass spectrometry data</td></tr>
<tr><td>Generated Results by ChatGPT-4</td><td>To identify proteins in tumor and normal samples based on mass spectrometry data, you'll need to perform the following steps:

1. Install necessary software.
2. Perform database search.
3. Filter, group and annotate results.

Here's the detailed plan and the accompanying code snippets. In the context of this guide, we are using OpenMS, which is a powerful open-source software used for mass spectrometry data analysis.

1. Install Necessary Software

It's assumed that package management software `conda` is installed. If not, install it first via the Miniconda distribution (https://docs.conda.io/en/latest/miniconda.html). The following commands install the OpenMS package on your Ubuntu system:

```bash
conda create -n openms
conda activate openms
conda install -c bioconda openms
```

2. Perform Database Search</td></tr>
</table>

The database search can be performed by multiple search engines. Here we use OMSSA search engine, but the process is similar with other tools. First, convert the raw files to the mzML format which is readable in OpenMS, and then run the database search via `OMSSAAdapter`.

```bash
# Convert the raw files to mzML
for raw_file in *.mzML.gz
do
   ThermoRawFileConverter -in $raw_file -out ${raw_file%.mzML.gz}.mzML
done

# Run the database search. Batch mode for multiple files.
for file in *.mzML
do
   OMSSAAdapter -in $file -out ${file%.mzML}.idXML -db
UP000005640_9606.fasta -enzyme_trypticity full -fragment_method CID -
precursor_mass_tolerance 10.0 -fragment_mass_tolerance 0.5
done
```

Replace `UP000005640_9606.fasta` with your actual database path. This code assumes that digesting enzyme is trypsin, fragment method is CID, and the tolerance for precursor and fragment ions is 10 PPM and 0.5 Da, respectively.

3. Filter, Group and Annotate Results

After database search, the results need to be processed, filtered, and grouped for reliable identification results.

```bash
# False Discovery Rate filtering and protein grouping
for id_file in *.idXML
do
   IDFilter -in $id_file -out ${id_file%.idXML}_filtered.idXML -score:pep 0.01
   IDPosteriorErrorProbability -in ${id_file%.idXML}_filtered.idXML -out
${id_file%.idXML}_scored.idXML
   FalseDiscoveryRate -in ${id_file%.idXML}_scored.idXML -out
${id_file%.idXML}_fdr.idXML -q_value_threshold 0.01
   IDMapper -in ${id_file%.idXML}_fdr.idXML -out
${id_file%.idXML}_mapped.idXML -spectrum_reference ms_run[1] -
id:peptides_threshold_score 0.01
   ProteinInference -in ${id_file%.idXML}_mapped.idXML -out
${id_file%.idXML}_proteins.idXML
done
```

This filters PSMs with a PEP cutoff (=0.01), followed by False Discovery Rate estimation for FDR-controlled protein inference. We also group the proteins with shared identified peptides.

	You may need to adjust or extend the given protocols to better suit your analytical requirements. Also, replace `.mzML.gz` and `.mzML` file paths with the actual data directories.<br><br>Note: This workflow is a basic representation and can be a start of a more comprehensive mass spectrometry proteomics analysis. Other procedures like quantitation, statistical testing, and downstream bioinformatics analysis can be included based on your study goal.

Metabolomics Case 21.1: metabolites concentration

**User's Inputs**	You are a bioinformatician who specializes in writing code and performing analysis for a variety of bioinformatic data analysis tasks. Now I provide you with an Ubuntu 18.04 system where I save the following data, each line including the data path and data description.<br><br>B1S1_f01.mzML.gz: LC-MS mass spectrometry data of metabolites in mzML format, tumor samples fraction 1<br>B1S1_f02.mzML.gz: LC-MS mass spectrometry data of metabolites in mzML format, tumor samples fraction 2<br>B1S1_f03.mzML.gz: LC-MS mass spectrometry data of metabolites in mzML format, tumor samples fraction 3<br>11CO047_f01.mzML.gz: LC-MS mass spectrometry data of metabolites in mzML format, normal samples fraction 1<br>11CO047_f02.mzML.gz: LC-MS mass spectrometry data of metabolites in mzML format, normal samples fraction 2<br>11CO047_f03.mzML.gz: LC-MS mass spectrometry data of metabolites in mzML format, normal samples fraction 3<br><br>I need you to help me plan what needs to be done in each step of the analysis process, and help me write the code for each step to install softwares, use tools and accomplish the following main goal:<br><br>calculate metabolites concentration in tumor and normal samples based on LC-MS mass spectrometry data
**Generated Results by ChatGPT-4**	Quantifying metabolites from LC-MS data involves the following steps:<br><br>1. Install necessary software.<br>2. Convert the raw mzML files to an OpenMS-compatible format (if they're not already).<br>3. Perform peak picking.<br>4. Perform feature detection.<br>5. Align features to a consensus metabolite map.<br>6. Use the consensus map to quantify metabolites.<br><br>Below, we focus on a workflow using OpenMS, an open-source software for LC-MS data analysis. The workflow may vary depending on the characteristics of your samples and the nature of your experiment.<br><br>### 1. Install Required Software<br><br>You need to install OpenMS:<br><br>```bash<br>conda create -n openms<br>conda activate openms<br>conda install -c bioconda openms<br>```<br><br>### 2. Convert mzML Files (If Needed)

If the original mzML data compressed (gzipped), you would first need to decompress it. Here we use the `gzip` command in Linux:

```bash
gunzip *.mzML.gz
```

3. Perform Peak Picking

The initial stage in processing involves peak picking using a tool like the OpenMS `PeakPickerHiRes` tool.

```bash
for file in *.mzML
do
  PeakPickerHiRes -in $file -out picked-$file
done
```

4. Perform Feature Detection

Feature detection groups peaks across different scans (from the same sample) into features representing the same analyte (e.g., metabolite ion). An example feature detection tool provided by OpenMS is `FeatureFinderMetabo`.

```bash
for file in picked-*.mzML
do
  FeatureFinderMetabo -in $file -out feature-$file
done
```

5. Align Features to a Consensus Metabolite Map

After feature detection, you need to align features from different LC-MS runs. `FeatureLinkerUnlabeledQT` is one tool in OpenMS that can achieve this.

```bash
FeatureLinkerUnlabeledQT -in feature-*.mzML -out consensus.xml
```

6. Use the Consensus Map to Quantify Metabolites

Once you obtain the consensus map, you can use it as a reference to quantify each metabolite in individual LC-MS run. In OpenMS, you can use `FeatureFinderIdentification` to do this:

```bash
for file in picked-*.mzML
do
  FeatureFinderIdentification -in $file -id consensus.xml -out quant-$file
```

	done<br>```<br><br>These are the basic steps. This workflow assumes that the LC-MS data is unlabeled (no stable isotope labeling was used). If you have labeled data or if you want to perform identification of the metabolites, you would require additional steps and potentially additional data (e.g., tandem MS data), so the workflow would need adjustments.<br><br>Please replace `*.mzML.gz` and `*.mzML` with your actual data directories. Additional adjustments may be needed depending on the specifics of your project, the particular analytical technique used for LC-MS, and the setup of your mass spectrometer. Be sure to thoroughly check your data at each stage to ensure the quality and accuracy of the results. More detailed information can be found in the OpenMS documentation: https://abibuilder.informatik.uni-tuebingen.de/archive/openms/Documentation/release/2.6.0/html/index.html

10. Results of CodeLlama-34B-Instruct

WGS data analysis Case 1.1: Genome assembly

**User's Inputs**	You are a bioinformatician who specializes in writing code and performing analysis for a variety of bioinformatic data analysis tasks. Now I provide you with an Ubuntu 18.04 system where I save the following data, each line including the data path and data description.<br><br>SRR1234567_R1.fastq.gz: Paired-end Illumina WGS reads, forward<br>SRR1234567_R2.fastq.gz: Paired-end Illumina WGS reads, reverse<br>TruSeq3-PE.fa: Adapter sequences for trimming<br>Reference.fasta: Reference genome for the organism under study in FASTA format<br><br>I need you to help me plan what needs to be done in each step of the analysis process, and help me write the code for each step to install softwares, use tools and accomplish the following main goal:<br><br>To perform genome assembly using paired-end WGS data
**Generated Results by CodeLlama-34B-Instruct**	Agent message<br>Based on the goal, here is the plan for performing genome assembly using paired-end WGS data:<br><br>1. **Install Required Software**:<br>- Install the necessary software for genome assembly, such as SPAdes or Velvet.<br>- Use the package manager (e.g., apt-get) to install the software on Ubuntu 18.04.<br><br>2. **Download and Prepare Data**:<br>- Download the paired-end Illumina WGS reads (SRR1234567_R1.fastq.gz and SRR1234567_R2.fastq.gz) and the adapter sequences (TruSeq3-PE.fa) to your Ubuntu system.<br>- Create a directory to store the data and move the downloaded files to that directory.<br>- Unzip the fastq.gz files if necessary.<br><br>3. **Quality Control and Trimming**:<br>- Perform quality control on the raw reads using tools like FastQC or Trimmomatic.<br>- Use the adapter sequences (TruSeq3-PE.fa) to trim the reads and remove any adapter contamination or low-quality regions.<br><br>4. **Genome Assembly**:<br>- Use the trimmed reads as input for the genome assembly software (SPAdes or Velvet).<br>- Specify the appropriate parameters for the assembly, such as read length, k-mer size, and coverage cutoffs.<br>- Run the assembly process and generate the assembled genome.<br><br>5. **Evaluate and Validate the Assembly**:

	- Assess the quality of the assembled genome using tools like QUAST or BUSCO.<br>- Compare the assembled genome to the reference genome (Reference.fasta) to evaluate its accuracy and completeness.<br><br>6. **Documentation and Reporting**:<br>- Document the entire analysis process, including the software used, parameters, and steps performed.<br>- Generate a report summarizing the results, including statistics on the assembled genome and any identified issues or limitations.<br>- Provide instructions on how to access and share the final assembled genome.<br><br>Please note that the above plan provides a general outline for the analysis process. The specific commands and code may vary depending on the software versions, data format, and specific requirements of your analysis.<br><br>The formatting requirements are as follows:<br><br>1. Organize the data description and data path in a clear and readable format.<br>2. Use a consistent naming convention for the data files and their descriptions.<br>3. Use proper punctuation and capitalization for clarity and readability.<br>4. Use a bulleted or numbered list to outline the steps of the analysis process.<br>5. Use code formatting or a monospaced font to distinguish code snippets or commands from regular text.<br>6. Clearly specify the main goal of the analysis process.<br><br>Here's an example of how the information could be formatted:<br><br>Data:<br>- SRR1234567_R1.fastq.gz: Paired-end Illumina WGS reads, forward<br>- SRR1234567_R2.fastq.gz: Paired-end Illumina WGS reads, reverse<br>- TruSeq3-PE.fa: Adapter sequences for trimming<br>- Reference.fasta: Reference genome for the organism under study in FASTA format<br><br>Analysis Process:<br>1. Install necessary software and tools:<br>- [Software 1]<br>- [Software 2]<br>- [Tool 1]<br>- [Tool 2]<br><br>2. Preprocess the data:<br>- Trim adapter sequences using [Tool 1] and [TruSeq3-PE.fa]<br>- Quality control and filtering of reads<br><br>3. Perform genome assembly:<br>- Use [Tool 2] for genome assembly

	- Input paired-end WGS data [SRR1234567_R1.fastq.gz] and [SRR1234567_R2.fastq.gz]<br>- Utilize [Reference.fasta] as the reference genome<br><br>Main Goal: Perform genome assembly using paired-end WGS data.

WGS/WES data analysis **Case 2.1 and Case 2.2 : Somatic SNV+indel calling and annotation**

**User's Inputs**	You are a bioinformatician who specializes in writing code and performing analysis for a variety of bioinformatic data analysis tasks. Now I provide you with an Ubuntu 18.04 system where I save the following data, each line including the data path and data description.<br><br>SRR23015874_R1.fastq.gz: paired end read1 from human whole genome sequencing, normal samples replicate 1<br>SRR23015874_R2.fastq.gz: paired end read2 from human whole genome sequencing, normal samples replicate 1<br>SRR23015876_R1.fastq.gz: paired end read1 from human whole genome sequencing, tumor samples replicate 1<br>SRR23015876_R2.fastq.gz: paired end read2 from human whole genome sequencing, tumor samples replicate 1<br>SRR23015877_R1.fastq.gz: paired end read1 from human whole genome sequencing, tumor samples replicate 2<br>SRR23015877_R2.fastq.gz: paired end read2 from human whole genome sequencing, tumor samples replicate 2<br>TruSeq3-PE.fa: trimming adapter<br>hg38.fa: human hg38 reference genome fasta<br><br>I need you to help me plan what needs to be done in each step of the analysis process, and help me write the code for each step to install softwares, use tools and accomplish the following main goal:<br><br>call somatic mutations in tumor samples over the matched normal samples and annotate the identified mutations
**Generated Results by CodeLlama-34B-Instruct**	> Assistant: Sure, I can help you with that. Here's a high-level plan for the analysis process:<br><br>1. Install necessary software:<br>* FastQC: for quality control of the raw sequencing data<br>* Trimmomatic: for adapter and quality trimming of the raw sequencing data<br>* BWA: for aligning the trimmed sequencing data to the reference genome<br>* Samtools: for sorting, indexing, and viewing the aligned sequencing data<br>* GATK: for variant calling and annotation of the identified mutations<br>2. Quality control:<br>* Use FastQC to assess the quality of the raw sequencing data<br>* Use Trimmomatic to trim the raw sequencing data and remove low-quality reads<br>3. Alignment:<br>* Use BWA to align the trimmed sequencing data to the reference genome<br>* Use Samtools to sort, index, and view the aligned sequencing data<br>4. Variant calling:<br>* Use GATK to call variants from the aligned sequencing data<br>* Use GATK to annotate the identified mutations with information such as functional impact, gene expression levels, and mutational load

5. Annotation:
* Use GATK to annotate the identified mutations with information such as functional impact, gene expression levels, and mutational load
6. Filtering:
* Use GATK to filter the identified mutations based on various criteria such as read depth, allele frequency, and functional impact
7. Visualization:
* Use GATK to visualize the identified mutations in a genomic browser such as IGV

Here's an example code for each step:

1. Install necessary software:
```bash
sudo apt-get update
sudo apt-get install fastqc trimmomatic bwa samtools gatk
```
2. Quality control:
```bash
fastqc SRR23015874_R1.fastq.gz SRR23015874_R2.fastq.gz
SRR23015876_R1.fastq.gz SRR23015876_R2.fastq.gz
SRR23015877_R1.fastq.gz SRR23015877_R2.fastq.gz
```
3. Alignment:
```bash
bwa mem -t 4 hg38.fa SRR23015874_R1.fastq.gz
SRR23015874_R2.fastq.gz SRR23015876_R1.fastq.gz
SRR23015876_R2.fastq.gz SRR23015877_R1.fastq.gz
SRR23015877_R2.fastq.gz | samtools view -bS - | samtools sort -o
aligned.bam -
```
4. Variant calling:
```bash
gatk HaplotypeCaller -R hg38.fa -I aligned.bam -O variants.vcf
```
5. Annotation:
```bash
gatk AnnotateVariants -R hg38.fa -V variants.vcf -O annotated.vcf
```
6. Filtering:
```bash
gatk VariantFiltration -R hg38.fa -V annotated.vcf -O filtered.vcf
```
7. Visualization:
```bash
igv -b aligned.bam -g hg38.fa -v filtered.vcf
```
Note that this is just an example code and may need to be modified based on the specific requirements of your analysis. Additionally, this code assumes that you have the necessary software and data files in the correct locations.

WGS/WES data analysis **Case 2.3: Structure variation identification with normal**

**User's Inputs**	You are a bioinformatician who specializes in writing code and performing analysis for a variety of bioinformatic data analysis tasks. Now I provide you with an Ubuntu 18.04 system where I save the following data, each line including the data path and data description.<br><br>SRR23015874_R1.fastq.gz: paired end read1 from human whole genome sequencing, the matched normal samples replicate 1<br>SRR23015874_R2.fastq.gz: paired end read2 from human whole genome sequencing, the matched normal samples replicate 1<br>SRR23015876_R1.fastq.gz: paired end read1 from human whole genome sequencing, tumor samples replicate 1<br>SRR23015876_R2.fastq.gz: paired end read2 from human whole genome sequencing, tumor samples replicate 1<br>SRR23015877_R1.fastq.gz: paired end read1 from human whole genome sequencing, tumor samples replicate 2<br>SRR23015877_R2.fastq.gz: paired end read2 from human whole genome sequencing, tumor samples replicate 2<br>TruSeq3-PE.fa: trimming adapter<br>hg38.fa: human hg38 reference genome fasta<br><br>I need you to help me plan what needs to be done in each step of the analysis process, and help me write the code for each step to install softwares, use tools and accomplish the following main goal:<br><br>To identify structural variations, which can include large insertions, deletions, inversions, duplications, and translocations, by comparing the sequencing data from the tumor sample over the matched normal sample
**Generated Results by CodeLlama-34B-Instruct**	> Assistant: Sure, I can help you with that. Here's a high-level overview of the steps involved in the analysis process:<br><br>1. Quality Control:<br>* Trim the adapters from the sequencing reads using Trimmomatic.<br>* Remove low-quality reads and reads with low mapping quality using Trimmomatic.<br>* Filter out reads with high levels of noise or errors using Trimmomatic.<br>2. Mapping:<br>* Map the trimmed and filtered reads to the human hg38 reference genome using BWA.<br>* Sort the mapped reads by coordinate using Samtools.<br>3. Variant Calling:<br>* Identify potential structural variations using a tool such as BreakDancer or Delly.<br>* Filter the identified variants based on quality and evidence.<br>4. Genotyping:<br>* Genotype the identified variants using a tool such as GATK.<br>* Filter the genotypes based on quality and evidence.<br>5. Annotation:<br>* Annotate the genotypes with information about the structural variations, such as the type of variation (insertion, deletion, inversion, etc.), the size of the variation, and the location of the variation in the genome.

6. Visualization:
* Visualize the genotypes and annotations using a tool such as Integrative Genomics Viewer (IGV) or UCSC Genome Browser.

Here's an example of how you could write code for each step of the analysis process:

1. Quality Control:
```bash
# Trim adapters from reads
trimmomatic PE -threads 4 SRR23015874_R1.fastq.gz
SRR23015874_R2.fastq.gz SRR23015876_R1.fastq.gz
SRR23015876_R2.fastq.gz SRR23015877_R1.fastq.gz
SRR23015877_R2.fastq.gz -adapter TruSeq3-PE.fa -o
trimmed_reads.fastq.gz

# Remove low-quality reads and reads with low mapping quality
trimmomatic PE -threads 4 trimmed_reads.fastq.gz -o
filtered_reads.fastq.gz -quality 30 -mapq 30

# Filter out reads with high levels of noise or errors
trimmomatic PE -threads 4 filtered_reads.fastq.gz -o
filtered_reads_no_noise.fastq.gz -noise 0.05
```
2. Mapping:
```bash
# Map reads to reference genome
bwa mem -t 4 hg38.fa filtered_reads_no_noise.fastq.gz >
mapped_reads.sam

# Sort mapped reads by coordinate
samtools sort -o sorted_mapped_reads.bam mapped_reads.sam
```
3. Variant Calling:
```bash
# Identify potential structural variations using BreakDancer
breakdancer -t 4 -o breakdancer_output.vcf sorted_mapped_reads.bam

# Filter BreakDancer output based on quality and evidence
breakdancer_filter -t 4 -o filtered_breakdancer_output.vcf
breakdancer_output.vcf
```
4. Genotyping:
```bash
# Genotype the identified variants using GATK
gatk GenotypeGVCFs -R hg38.fa -V breakdancer_output.vcf -o
genotyped_breakdancer_output.vcf

# Filter the genotypes based on quality and evidence
gatk VariantFiltration -R hg38.fa -V genotyped_breakdancer_output.vcf -o
filtered_genotyped_breakdancer_output.vcf
```
5. Annotation:

	```bash<br># Annotate the genotypes with information about the structural variations<br>gatk AnnotateVariants -R hg38.fa -V<br>filtered_genotyped_breakdancer_output.vcf -o annotated_genotypes.vcf<br>```<br>6. Visualization:<br>```bash<br># Visualize the genotypes and annotations using IGV<br>igv -b annotated_genotypes.vcf -g hg38.fa<br>```<br>Note that this is just an example and the specific commands and parameters may need to be adjusted based on the specific requirements of your analysis. Additionally, this is a high-level overview of the steps involved in the analysis process and there may be additional steps or variations depending on the specific requirements of your analysis.

WGS/WES data analysis **Case 2.4: Structure variation identification without normal**

<table>
<tr><td>User's Inputs</td><td>You are a bioinformatician who specializes in writing code and performing analysis for a variety of bioinformatic data analysis tasks. Now I provide you with an Ubuntu 18.04 system where I save the following data, each line including the data path and data description.

SRR23015876_R1.fastq.gz: paired end read1 from human whole genome sequencing, tumor samples replicate 1
SRR23015876_R2.fastq.gz: paired end read2 from human whole genome sequencing, tumor samples replicate 1
SRR23015877_R1.fastq.gz: paired end read1 from human whole genome sequencing, tumor samples replicate 2
SRR23015877_R2.fastq.gz: paired end read2 from human whole genome sequencing, tumor samples replicate 2
TruSeq3-PE.fa: trimming adapter
hg38.fa: human hg38 reference genome fasta

I need you to help me plan what needs to be done in each step of the analysis process, and help me write the code for each step to install softwares, use tools and accomplish the following main goal:

To identify structural variations, which can include large insertions, deletions, inversions, duplications, and translocations based on the sequencing data from the tumor sample.</td></tr>
<tr><td>Generated Results by CodeLlama-34B-Instruct</td><td>Sure, I can help you with that. Here's a high-level plan for the analysis process:

1. Install necessary software and tools:
* FastQC: for quality control of the sequencing data
* Trimmomatic: for adapter trimming
* BWA: for aligning the trimmed reads to the reference genome
* Samtools: for sorting and indexing the aligned reads
* GATK: for identifying structural variations
2. Quality control of the sequencing data:
* Run FastQC on the sequencing data to assess the quality of the reads
* Check for any issues with the data, such as low quality scores or adapter contamination
3. Adapter trimming:
* Use Trimmomatic to remove adapters from the sequencing data
* This will improve the accuracy of the alignment and reduce the computational requirements
4. Alignment of the trimmed reads to the reference genome:
* Use BWA to align the trimmed reads to the reference genome
* This will allow us to identify the location of the structural variations in the genome
5. Sorting and indexing the aligned reads:
* Use Samtools to sort and index the aligned reads
* This will allow us to quickly access and manipulate the data
6. Identification of structural variations:
* Use GATK to identify structural variations, such as large insertions, deletions, inversions, duplications, and translocations</td></tr>
</table>

* This will allow us to identify the genetic changes that are driving the tumor growth
7. Visualization and interpretation of the results:
* Use a visualization tool, such as Integrative Genomics Viewer (IGV), to visualize the structural variations and interpret the results

Here's some sample code for each step:

1. Install necessary software and tools:
```bash
sudo apt-get update
sudo apt-get install fastqc trimmomatic bwa samtools gatk
```
2. Quality control of the sequencing data:
```bash
fastqc SRR23015876_R1.fastq.gz SRR23015876_R2.fastq.gz
SRR23015877_R1.fastq.gz SRR23015877_R2.fastq.gz
```
3. Adapter trimming:
```bash
trimmomatic PE -threads 4 SRR23015876_R1.fastq.gz
SRR23015876_R2.fastq.gz SRR23015877_R1.fastq.gz
SRR23015877_R2.fastq.gz
```
4. Alignment of the trimmed reads to the reference genome:
```bash
bwa mem -t 4 hg38.fa SRR23015876_R1.fastq.gz
SRR23015876_R2.fastq.gz SRR23015877_R1.fastq.gz
SRR23015877_R2.fastq.gz | samtools view -bS - | samtools sort -o
aligned.bam
```
5. Sorting and indexing the aligned reads:
```bash
samtools sort aligned.bam -o sorted.bam
samtools index sorted.bam
```
6. Identification of structural variations:
```bash
gatk --java-options "-Xmx4g" HaplotypeCaller -R hg38.fa -I sorted.bam -O
output.vcf
```
7. Visualization and interpretation of the results:
```bash
igv -b output.vcf -g hg38.fa
```
Note that this is just a high-level plan and the specific commands and parameters may need to be adjusted based on the specific requirements of the analysis. Additionally, this plan assumes that the data is already in the correct format and that the necessary software and tools are installed.--------------

ChIP-Seq Analysis Case 3.1: Call Peaks

<table>
<tr><td>User's Inputs</td><td>You are a bioinformatician who specializes in writing code and performing analysis for a variety of bioinformatic data analysis tasks. Now I provide you with an Ubuntu 18.04 system where I save the following data, each line including the data path and data description.

SRR620204.fastq.gz: chip-seq data for Ring1B
SRR620205.fastq.gz: chip-seq data for cbx7
SRR620206.fastq.gz: chip-seq data for SUZ12
SRR620208.fastq.gz: chip-seq data for IgGold
mm39.ncbiRefSeq.gtf: genome annotations mouse
mm39.fa: mouse genome

I need you to help me plan what needs to be done in each step of the analysis process, and help me write the code for each step to install softwares, use tools and accomplish the following main goal:

Call peaks for protein cbx7 with IgGold as control</td></tr>
<tr><td>Generated Results by CodeLlama-34B-Instruct</td><td>Assistant: Sure, I can help you with that! Here's a high-level plan for the analysis process:

1. Install necessary software:
* FastQC: for quality control of raw sequencing data
* Trimmomatic: for adapter and quality trimming of raw sequencing data
* Bowtie2: for aligning trimmed sequencing reads to the reference genome
* MACS2: for peak calling
* Bedtools: for manipulating and analyzing genomic intervals
2. Quality control and trimming of raw sequencing data:
* Use FastQC to assess the quality of the raw sequencing data
* Use Trimmomatic to trim the raw sequencing data for adapter and quality control
3. Alignment of trimmed sequencing reads to the reference genome:
* Use Bowtie2 to align the trimmed sequencing reads to the reference genome
4. Peak calling for protein cbx7 with IgGold as control:
* Use MACS2 to call peaks for protein cbx7 with IgGold as control
* Use Bedtools to manipulate and analyze the genomic intervals
5. Visualization and interpretation of peak calls:
* Use a genome browser such as UCSC Genome Browser or Integrative Genomics Viewer (IGV) to visualize the peak calls
* Interpret the peak calls in the context of the genome and the biological question being asked

Here's some sample code for each step:

1. Installing necessary software:
```bash
sudo apt-get update
sudo apt-get install fastqc trimmomatic bowtie2 macs2 bedtools
```
2. Quality control and trimming of raw sequencing data:</td></tr>
</table>

```bash
fastqc SRR620204.fastq.gz SRR620205.fastq.gz SRR620206.fastq.gz SRR620208.fastq.gz
trimmomatic SRR620204.fastq.gz SRR620205.fastq.gz SRR620206.fastq.gz SRR620208.fastq.gz
```

3. Alignment of trimmed sequencing reads to the reference genome:

```bash
bowtie2-build mm39.fa mm39
bowtie2 -x mm39 -1 SRR620204_1.fastq.gz -2 SRR620204_2.fastq.gz -S SRR620204.sam
bowtie2 -x mm39 -1 SRR620205_1.fastq.gz -2 SRR620205_2.fastq.gz -S SRR620205.sam
bowtie2 -x mm39 -1 SRR620206_1.fastq.gz -2 SRR620206_2.fastq.gz -S SRR620206.sam
bowtie2 -x mm39 -1 SRR620208_1.fastq.gz -2 SRR620208_2.fastq.gz -S SRR620208.sam
```

4. Peak calling for protein cbx7 with IgGold as control:

```bash
macs2 callpeak -t SRR620204.fastq.gz -c SRR620205.fastq.gz -f BAM -g mm39.fa -n cbx7_IgGold
```

5. Visualization and interpretation of peak calls:

```bash
bedtools genomecov -ibam SRR620204.bam -bg SRR620204.bedGraph
bedtools genomecov -ibam SRR620205.bam -bg SRR620205.bedGraph
bedtools genomecov -ibam SRR620206.bam -bg SRR620206.bedGraph
bedtools genomecov -ibam SRR620208.bam -bg SRR620208.bedGraph
```

Note that this is just a high-level plan and you may need to modify the code and parameters based on your specific needs and the characteristics of your data. Additionally, you may need to install additional software or tools to perform certain tasks.

ChIP-Seq Analysis   Case 3.2: Motif Discovery

<table>
<tr><td>User's Inputs</td><td>You are a bioinformatician who specializes in writing code and performing analysis for a variety of bioinformatic data analysis tasks. Now I provide you with an Ubuntu 18.04 system where I save the following data, each line including the data path and data description.

SRR620204.fastq.gz: chip-seq data for Ring1B
SRR620205.fastq.gz: chip-seq data for cbx7
SRR620206.fastq.gz: chip-seq data for SUZ12
SRR620208.fastq.gz: chip-seq data for IgGold
mm39.ncbiRefSeq.gtf: genome annotations mouse
mm39.fa: mouse genome

I need you to help me plan what needs to be done in each step of the analysis process, and help me write the code for each step to install softwares, use tools and accomplish the following main goal:

Discover motifs within the peaks of protein SUZ12 with IgGold as control</td></tr>
<tr><td>Generated Results by CodeLlama-34B-Instruct</td><td>Assistant: Sure, I can help you with that! Here's a high-level plan for the analysis process:

1. Install necessary software:
* FastQC: for quality control of raw sequencing data
* Trimmomatic: for adapter and quality trimming of raw sequencing data
* Bowtie2: for aligning trimmed sequencing reads to the reference genome
* HISAT2: for aligning trimmed sequencing reads to the reference genome
* Samtools: for sorting and indexing aligned sequencing reads
* Bedtools: for peak calling and motif discovery
* MEME: for motif discovery
2. Preprocess the data:
* Use FastQC to assess the quality of the raw sequencing data
* Use Trimmomatic to trim the raw sequencing data for adapter and quality control
* Use Bowtie2 or HISAT2 to align the trimmed sequencing reads to the reference genome
* Use Samtools to sort and index the aligned sequencing reads
3. Identify peaks:
* Use Bedtools to identify peaks in the aligned sequencing reads
* Use the peaks to create a peak set for each sample (SUZ12 and IgGold)
4. Discover motifs:
* Use MEME to discover motifs within the peaks of SUZ12
* Use the motifs to create a motif set for SUZ12
5. Compare motifs:
* Use the motif set for SUZ12 to compare with the motif set for IgGold
* Use a statistical test to determine if the motifs are significantly different between the two samples
6. Interpret results:</td></tr>
</table>

* Based on the results of the statistical test, determine if there are any significant differences in the motifs between SUZ12 and IgGold
* If there are significant differences, interpret the results in the context of the biological question being asked

Here's some example code for each step of the analysis process:

1. Install necessary software:
```bash
sudo apt-get update
sudo apt-get install fastqc trimmomatic bowtie2 hisat2 samtools bedtools meme
```
2. Preprocess the data:
```bash
# FastQC
fastqc SRR620204.fastq.gz SRR620205.fastq.gz SRR620206.fastq.gz SRR620208.fastq.gz

# Trimmomatic
trimmomatic PE -threads 4 SRR620204.fastq.gz SRR620205.fastq.gz SRR620206.fastq.gz SRR620208.fastq.gz

# Bowtie2 or HISAT2
bowtie2 -p 4 -x mm39 -U SRR620204.fastq.gz SRR620205.fastq.gz SRR620206.fastq.gz SRR620208.fastq.gz
hisat2 -p 4 -x mm39 -U SRR620204.fastq.gz SRR620205.fastq.gz SRR620206.fastq.gz SRR620208.fastq.gz

# Samtools
samtools view -bS SRR620204.fastq.gz SRR620205.fastq.gz SRR620206.fastq.gz SRR620208.fastq.gz | samtools sort -o SRR620204.bam SRR620205.bam SRR620206.bam SRR620208.bam
```
3. Identify peaks:
```bash
# Bedtools
bedtools genomecov -ibam SRR620204.bam -bg SRR620204.bedgraph
bedtools genomecov -ibam SRR620205.bam -bg SRR620205.bedgraph
bedtools genomecov -ibam SRR620206.bam -bg SRR620206.bedgraph
bedtools genomecov -ibam SRR620208.bam -bg SRR620208.bedgraph

# Create peak sets
bedtools intersect -a SRR620204.bedgraph -b SRR620205.bedgraph -c > SRR620204_vs_SRR620205.bed
bedtools intersect -a SRR620204.bedgraph -b SRR620206.bedgraph -c > SRR620204_vs_SRR620206.bed
bedtools intersect -a SRR620204.bedgraph -b SRR620208.bedgraph -c > SRR620204_vs_SRR620208.bed
```
4. Discover motifs:
```bash
# MEME
```

meme -oc SRR620204_vs_SRR620205.meme
SRR620204_vs_SRR620205.bed
meme -oc SRR620204_vs_SRR620206.meme
SRR620204_vs_SRR620206.bed
meme -oc SRR620204_vs_SRR620208.meme
SRR620204_vs_SRR620208.bed
```

5. Compare motifs:

```bash
# Compare motifs
memecompare -oc SRR620204_vs_SRR620205.meme
SRR620204_vs_SRR620205.meme SRR620204_vs_SRR620206.meme
SRR620204_vs_SRR620208.meme
```

6. Interpret results:

```bash
# Interpret results
# ...
```

Note that this is just an example code and may need to be modified based on the specific requirements of your analysis. Additionally, this code assumes that you have the necessary software installed and that the data is in the correct format.

```

ChIP-Seq Analysis   Case 3.3: Functional Enrichment

<table>
<tr><td>User's Inputs</td><td>You are a bioinformatician who specializes in writing code and performing analysis for a variety of bioinformatic data analysis tasks. Now I provide you with an Ubuntu 18.04 system where I save the following data, each line including the data path and data description.

SRR620204.fastq.gz: chip-seq data for Ring1B
SRR620205.fastq.gz: chip-seq data for cbx7
SRR620206.fastq.gz: chip-seq data for SUZ12
SRR620208.fastq.gz: chip-seq data for IgGold
mm39.ncbiRefSeq.gtf: genome annotations mouse
mm39.fa: mouse genome

I need you to help me plan what needs to be done in each step of the analysis process, and help me write the code for each step to install softwares, use tools and accomplish the following main goal:

Perform functional enrichment for protein Ring1B, use protein IgGold as the control</td></tr>
<tr><td>Generated Results by CodeLlama-34B-Instruct</td><td>Assistant: Sure, I can help you with that. Here's a step-by-step plan for the analysis process:

1. Install necessary software:
* FastQC: a quality control tool for high-throughput sequencing data.
* Trimmomatic: a tool for trimming adapters and low-quality bases from FastQ files.
* HISAT2: a spliced aligner for RNA-seq data.
* StringTie: a tool for quantifying gene expression and splicing events from RNA-seq data.
* Ballgown: a tool for differential gene expression analysis.
* GSEA: a tool for gene set enrichment analysis.
2. Preprocess the data:
* Use FastQC to assess the quality of the raw FastQ files.
* Use Trimmomatic to trim adapters and low-quality bases from the FastQ files.
* Use HISAT2 to align the trimmed FastQ files to the mouse genome.
* Use StringTie to quantify gene expression and splicing events from the aligned data.
3. Perform functional enrichment analysis:
* Use Ballgown to calculate the differential gene expression between Ring1B and IgGold.
* Use GSEA to perform gene set enrichment analysis for the differentially expressed genes.
4. Interpret the results:
* Analyze the results of the functional enrichment analysis to identify overrepresented gene ontology (GO) terms that are enriched in the differentially expressed genes.
* Use the results to infer the biological functions that are associated with the differentially expressed genes.</td></tr>
</table>

Here's some sample code for each step:

1. Installing necessary software:
```bash
sudo apt-get update
sudo apt-get install fastqc trimmomatic hisat2 stringtie ballgown gsea
```
2. Preprocessing the data:
```bash
# FastQC
fastqc SRR620204.fastq.gz SRR620205.fastq.gz SRR620206.fastq.gz
SRR620208.fastq.gz

# Trimmomatic
trimmomatic PE -threads 4 SRR620204.fastq.gz SRR620205.fastq.gz
SRR620206.fastq.gz SRR620208.fastq.gz

# HISAT2
hisat2 -p 4 -x mm39.fa -1 SRR620204.fastq.gz -2 SRR620205.fastq.gz -S
SRR620206.fastq.gz -U SRR620208.fastq.gz

# StringTie
stringtie -p 4 -G mm39.ncbiRefSeq.gtf -o SRR620204.gtf SRR620205.gtf
SRR620206.gtf SRR620208.gtf
```
3. Performing functional enrichment analysis:
```bash
# Ballgown
ballgown count -p 4 -o SRR620204.ballgown SRR620204.gtf
SRR620205.gtf SRR620206.gtf SRR620208.gtf

# GSEA
gsea -p 4 -o SRR620204.gsea SRR620204.ballgown SRR620205.ballgown
SRR620206.ballgown SRR620208.ballgown
```
4. Interpreting the results:
```bash
# Analyze the results of the functional enrichment analysis
gsea -p 4 -o SRR620204.gsea SRR620204.ballgown SRR620205.ballgown
SRR620206.ballgown SRR620208.ballgown

# Use the results to infer the biological functions that are associated with
the differentially expressed genes
gsea -p 4 -o SRR620204.gsea SRR620204.ballgown SRR620205.ballgown
SRR620206.ballgown SRR620208.ballgown | less
```
Note that this is just a sample code, and you may need to modify it based on your specific needs and the version of the software you are using. Additionally, you may need to install additional software or libraries to perform the analysis.

Bisulfite-Seq data analysis   Case 4.1: Identifying DNA methylation

**User's Inputs**	You are a bioinformatician who specializes in writing code and performing analysis for a variety of bioinformatic data analysis tasks. Now I provide you with an Ubuntu 18.04 system where I save the following data, each line including the data path and data description.<br><br>Sample_R1.fastq.gz: Raw sequencing reads from the bisulfite-converted DNA, typically paired-end reads. These reads contain information about the converted DNA, where unmethylated cytosines are converted to uracils (T) during library preparation.<br>Sample_R2.fastq.gz: Paired-end reads that complement the R1 reads. These are typically used for quality control and paired-end analysis.<br>TruSeq3-PE.fa: Adapter sequences for trimming<br>Reference.fasta: Reference genome for the organism under study in FASTA format, include bisulfite conversion.<br><br>I need you to help me plan what needs to be done in each step of the analysis process, and help me write the code for each step to install softwares, use tools and accomplish the following main goal:<br><br>To identify DNA methylation patterns in the genome based on the Bisulfite-Seq data.
**Generated Results by CodeLlama-34B-Instruct**	Assistant: Sure, I can help you with that! Here's a high-level plan for the analysis process:<br><br>1. Install necessary software and tools:<br>* FastQC: A quality control tool for high-throughput sequencing data.<br>* Trimmomatic: A tool for trimming adapters and low-quality bases from sequencing reads.<br>* Bismark: A tool for mapping bisulfite-converted DNA reads to a reference genome.<br>* Samtools: A tool for aligning and sorting sequencing reads.<br>* Bedtools: A tool for working with genomic intervals and features.<br>* DeepTools: A tool for visualizing and analyzing genomic data.<br>2. Preprocessing:<br>* Use FastQC to assess the quality of the raw sequencing reads.<br>* Use Trimmomatic to trim adapters and low-quality bases from the reads.<br>* Use Bismark to map the trimmed reads to the reference genome.<br>* Use Samtools to align and sort the mapped reads.<br>* Use Bedtools to convert the aligned reads to a bedGraph format.<br>3. Methylation calling:<br>* Use DeepTools to call methylation sites and generate a methylation profile.<br>* Use the methylation profile to identify regions of the genome that are differentially methylated.<br>4. Visualization and interpretation:<br>* Use DeepTools to visualize the methylation profile and identify regions of the genome that are differentially methylated.

* Use other tools such as UCSC Genome Browser or Integrative Genomics Viewer (IGV) to visualize the methylation profile and identify regions of the genome that are differentially methylated.

Here's some sample code for each step of the analysis process:

1. Install necessary software and tools:

```bash
sudo apt-get install fastqc trimmomatic bismark samtools bedtools deeptools
```

2. Preprocessing:

```bash
# FastQC
fastqc Sample_R1.fastq.gz Sample_R2.fastq.gz

# Trimmomatic
trimmomatic PE -phred33 Sample_R1.fastq.gz Sample_R2.fastq.gz \
  ILLUMINACLIP:TruSeq3-PE.fa:2:30:10 \
  LEADING:3 TRAILING:3 SLIDINGWINDOW:4:15 MINLEN:36

# Bismark
bismark --bowtie2 --bam --output-dir bismark_output --basename
Sample_R1_R2 \
  --unmapped-reads Sample_R1.fastq.gz Sample_R2.fastq.gz

# Samtools
samtools view -bS bismark_output/Sample_R1_R2.bam | \
  samtools sort -o bismark_output/Sample_R1_R2.sorted.bam

# Bedtools
bedtools bamtobed -i bismark_output/Sample_R1_R2.sorted.bam > \
  bismark_output/Sample_R1_R2.bed
```

3. Methylation calling:

```bash
# DeepTools
deeptools methylation_profile -b bismark_output/Sample_R1_R2.bed \
  -r Reference.fasta -o methylation_profile.txt

# Call methylation sites
deeptools methylation_sites -b bismark_output/Sample_R1_R2.bed \
  -r Reference.fasta -o methylation_sites.bed
```

4. Visualization and interpretation:

```bash
# Visualize methylation profile
deeptools plot_profile -b methylation_profile.txt -o methylation_profile.png

# Visualize methylation sites
deeptools plot_sites -b methylation_sites.bed -o methylation_sites.png
```

	Note that this is just a high-level plan and you may need to modify the code and parameters based on your specific needs and the characteristics of your data. Additionally, you may need to install additional software and tools to perform the analysis.<br>--------------

ATAC-seq data analysis **Case 5.1: Identifying open chromatin regions**

<table>
<tr><td>User's Inputs</td><td>You are a bioinformatician who specializes in writing code and performing analysis for a variety of bioinformatic data analysis tasks. Now I provide you with an Ubuntu 18.04 system where I save the following data, each line including the data path and data description.

Sample_R1.fastq.gz: Raw sequencing reads from the ATAC-seq experiment. These are typically paired-end reads representing fragments of open chromatin regions.
Sample_R2.fastq.gz: Paired-end reads that complement the R1 reads.
TruSeq3-PE.fa: Adapter sequences for trimming
Reference.fasta: Reference genome for the organism under study in FASTA format. It matches the genome used for the ATAC-seq library preparation.

I need you to help me plan what needs to be done in each step of the analysis process, and help me write the code for each step to install softwares, use tools and accomplish the following main goal:

To identify open chromatin regions within the genome based on the ATAC-seq data.</td></tr>
<tr><td>Generated Results by CodeLlama-34B-Instruct</td><td>Assistant: Sure, I can help you with that! Here's a high-level plan for the analysis process:

1. Quality Control:
* Trim the raw sequencing reads using TruSeq3-PE.fa adapters to remove adapter sequences and low-quality bases.
* Assess the quality of the trimmed reads using FastQC to identify any remaining issues.
2. Mapping:
* Map the trimmed reads to the reference genome using a suitable aligner (e.g. BWA, Bowtie2, STAR).
* Sort and index the mapped reads using Samtools.
3. Peak Calling:
* Use a peak calling tool (e.g. MACS2, HISAT2, SICER) to identify regions of the genome that are enriched for ATAC-seq signal.
* Filter the peaks to remove any low-quality or redundant peaks.
4. Annotation:
* Annotate the peaks with information about the genomic features they overlap, such as gene promoters, enhancers, or other regulatory elements.
* Use a tool such as Bedtools or Pybedtools to perform the annotation.
5. Visualization:
* Visualize the peaks and their annotations using a suitable tool (e.g. UCSC Genome Browser, Integrative Genomics Viewer, Circos).

Here's some sample code for each step:

1. Quality Control:
```bash
Trim the raw sequencing reads using TruSeq3-PE.fa adapters</td></tr>
</table>

```
cutadapt -a TruSeq3-PE.fa -o Sample_R1_trimmed.fastq.gz
Sample_R1.fastq.gz
cutadapt -a TruSeq3-PE.fa -o Sample_R2_trimmed.fastq.gz
Sample_R2.fastq.gz

# Assess the quality of the trimmed reads using FastQC
fastqc Sample_R1_trimmed.fastq.gz Sample_R2_trimmed.fastq.gz
```
2. Mapping:
```bash
# Map the trimmed reads to the reference genome using BWA
bwa mem -t 4 -M -R '@RG\tID:Sample\tSM:Sample' Reference.fasta
Sample_R1_trimmed.fastq.gz Sample_R2_trimmed.fastq.gz | samtools
view -bS - | samtools sort -o Sample.bam -

# Sort and index the mapped reads using Samtools
samtools sort Sample.bam -o Sample.sorted.bam
samtools index Sample.sorted.bam
```
3. Peak Calling:
```bash
# Use MACS2 to identify regions of the genome that are enriched for ATAC-
seq signal
macs2 callpeak -t Sample_R1_trimmed.fastq.gz -c
Sample_R2_trimmed.fastq.gz -f BAM -g hs -n Sample

# Filter the peaks to remove any low-quality or redundant peaks
macs2 filterdup -i Sample_peaks.narrowPeak -o
Sample_peaks.filtered.narrowPeak
```
4. Annotation:
```bash
# Annotate the peaks with information about the genomic features they
overlap
bedtools intersect -a Sample_peaks.filtered.narrowPeak -b Reference.gff -
wa -wb | awk '{print $1,$2,$3,$4,$5,$6,$7,$8,$9,$10}' >
Sample_peaks.annotated.bed
```
5. Visualization:
```bash
# Visualize the peaks and their annotations using UCSC Genome Browser
bedtools genomecov -ibam Sample.bam -bg > Sample.bedGraph
wigToBigWig Sample.bedGraph /path/to/chrom.sizes Sample.bw
```
Note that this is just a high-level plan and the specific tools and parameters used may vary depending on the specific requirements of your analysis.

DNase-seq data analysis **Case 6.1: Identifying DnaseI hypersensitive site**

**User's Inputs**	You are a bioinformatician who specializes in writing code and performing analysis for a variety of bioinformatic data analysis tasks. Now I provide you with an Ubuntu 18.04 system where I save the following data, each line including the data path and data description.<br><br>Sample_R1.fastq.gz: Raw sequencing reads from the DNase-seq experiment.<br>Sample_R2.fastq.gz: Paired-end reads that complement the R1 reads, often used for quality control and paired-end analysis.<br>TruSeq3-PE.fa: Adapter sequences for trimming.<br>Reference.fasta: Reference genome for the organism under study in FASTA format<br><br>I need you to help me plan what needs to be done in each step of the analysis process, and help me write the code for each step to install softwares, use tools and accomplish the following main goal:<br><br>To identify DNase I hypersensitive sites (regions of open chromatin) within the genome based on the DNase-seq data.
**Generated Results by CodeLlama-34B-Instruct**	Assistant: Sure, I can help you with that! Here's a high-level plan for the analysis process:<br><br>1. Quality Control and Trimming:<br>* Use FastQC to assess the quality of the raw sequencing reads (Sample_R1.fastq.gz and Sample_R2.fastq.gz).<br>* Use Trimmomatic to trim the adapters and low-quality bases from the reads.<br>2. Mapping:<br>* Use BWA to map the trimmed reads to the reference genome (Reference.fasta).<br>* Use Samtools to sort and index the aligned reads.<br>3. Peak Calling:<br>* Use MACS2 to identify regions of open chromatin (peaks) based on the aligned reads.<br>* Use Bedtools to convert the peak calls to BED format.<br>4. Annotation:<br>* Use Bedtools to annotate the peaks with genomic features such as gene names, transcript IDs, and chromatin marks.<br>* Use UCSC Genome Browser to visualize the peaks and their annotations.<br>5. Statistical Testing:<br>* Use DESeq2 to perform a statistical test for differentially methylated regions (DMRs) between the DNase-seq data and a control sample.<br>* Use edgeR to perform a statistical test for differentially expressed genes (DEGs) between the DNase-seq data and a control sample.<br>6. Pathway Analysis:<br>* Use DAVID to perform a pathway analysis for the DEGs identified in the previous step.<br>7. Visualization:

* Use Cytoscape to visualize the network of genomic features and their interactions.

Here's some sample code for each step:

1. Quality Control and Trimming:
```bash
# Install FastQC and Trimmomatic
sudo apt-get install fastqc trimmomatic

# Run FastQC on the raw sequencing reads
fastqc Sample_R1.fastq.gz Sample_R2.fastq.gz

# Trim the adapters and low-quality bases from the reads
trimmomatic PE -threads 4 Sample_R1.fastq.gz Sample_R2.fastq.gz \
  TA_R1.fastq.gz TA_R2.fastq.gz \
  ILLUMINACLIP:TruSeq3-PE.fa:2:30:10 \
  LEADING:3 TRAILING:3 SLIDINGWINDOW:4:15 MINLEN:36
```
2. Mapping:
```bash
# Install BWA and Samtools
sudo apt-get install bwa samtools

# Index the reference genome
samtools faidx Reference.fasta

# Map the trimmed reads to the reference genome
bwa mem -t 4 Reference.fasta TA_R1.fastq.gz TA_R2.fastq.gz >
Sample.sam

# Sort and index the aligned reads
samtools sort -o Sample.bam Sample.sam
samtools index Sample.bam
```
3. Peak Calling:
```bash
# Install MACS2
sudo apt-get install macs2

# Call peaks using MACS2
macs2 callpeak -t Sample.bam -c Sample.bam -f BAMPE -g hs -n Sample

# Convert the peak calls to BED format
bedtools bamtobed -i Sample.bam > Sample.bed
```
4. Annotation:
```bash
# Install Bedtools
sudo apt-get install bedtools

# Annotate the peaks with genomic features
```

<table>
<tr>
<td></td>
<td>bedtools annotate -i Sample.bed -db hg19_RefSeq.bed -c 4,6,8 -o Sample.annotated.bed

Visualize the peaks and their annotations using UCSC Genome Browser
```
5. Statistical Testing:
```bash
# Install DESeq2 and edgeR
sudo apt-get install r-cran-deseq2 r-cran-edger

Perform a statistical test for DMRs using DESeq2
Rscript -e "library(DESeq2); dds <- DESeqDataSetFromHTSeqCount(countData = Sample.bam, colData = Sample.annotated.bed, design = ~ condition); results <- results(dds); summary(results)"

Perform a statistical test for DEGs using edgeR
Rscript -e "library(edgeR); y <- DGEList(counts = Sample.bam, group = Sample.annotated.bed); results <- results(y); summary(results)"
```
6. Pathway Analysis:
```bash
# Install DAVID
sudo apt-get install david

Perform a pathway analysis for the DEGs identified in the previous step
david -g hs -i Sample.degs.txt -o Sample.david.txt
```
7. Visualization:
```bash
# Install Cytoscape
sudo apt-get install cytoscape

Visualize the network of genomic features and their interactions
cytoscape -i Sample.network.txt -o Sample.network.png
```
Note that this is just a high-level plan, and the specific commands and tools used may vary depending on the specific requirements of your analysis.</td>
</tr>
</table>

4C-seq data analysis   Case 7.1: Find genomics interactions

<table>
<tr><td>User's Inputs</td><td>You are a bioinformatician who specializes in writing code and performing analysis for a variety of bioinformatic data analysis tasks. Now I provide you with an Ubuntu 18.04 system where I save the following data, each line including the data path and data description.

sample_4C-seq_data.fastq: Raw sequencing reads or aligned data from the 4C-seq experiment, depending on the starting point.
viewpoint_coordinates.bed: A file specifying the genomic coordinates of the viewpoint we want to study.
reference_genome.fasta: A reference genome in FASTA format for the organism under study. This should match the genome used for the 4C-seq library preparation.

I need you to help me plan what needs to be done in each step of the analysis process, and help me write the code for each step to install softwares, use tools and accomplish the following main goal:

To identify genomic interactions originating from a specific genomic viewpoint using 4C-seq data.</td></tr>
<tr><td>Generated Results by CodeLlama-34B-Instruct</td><td>Assistant: Sure, I can help you with that! Here's a high-level plan for the analysis process:

1. Data preprocessing:
* Trim and filter the raw sequencing reads to remove low-quality reads and adapter sequences.
* Align the filtered reads to the reference genome using a tool such as BWA or Bowtie.
* Sort and index the aligned reads using a tool such as Samtools.
2. Viewpoint selection:
* Use the `bedtools` package to extract the genomic coordinates of the viewpoint from the `viewpoint_coordinates.bed` file.
* Use the `bedtools` package to extract the reads that overlap with the viewpoint coordinates.
3. Interaction identification:
* Use the `4C-seq` package to identify genomic interactions originating from the viewpoint.
* Use the `4C-seq` package to filter the interactions based on criteria such as distance, strength, and specificity.
4. Data visualization:
* Use a tool such as `IGV` or `UCSC Genome Browser` to visualize the interactions in a genome browser.
* Use a tool such as `matplotlib` or `seaborn` to create plots of the interactions.

Here's some sample code for each step:

1. Data preprocessing:
```bash
Trim and filter the raw sequencing reads
bwa mem -t 4 -M -R '@RG\tID:sample\tSM:sample' reference_genome.fasta sample_4C-seq_data.fastq | samtools view -b -F 4 -q 30 - | samtools sort -o sample_4C-seq_data.sorted.bam -</td></tr>
</table>

Align the filtered reads to the reference genome
bwa mem -t 4 -M -R '@RG\tID:sample\tSM:sample' reference_genome.fasta sample_4C-seq_data.sorted.bam | samtools view -b -F 4 -q 30 - | samtools sort -o sample_4C-seq_data.aligned.bam -

Sort and index the aligned reads
samtools sort -o sample_4C-seq_data.sorted.bam -
samtools index sample_4C-seq_data.sorted.bam
```
2. Viewpoint selection:
```bash
# Extract the genomic coordinates of the viewpoint
bedtools getfasta -fi reference_genome.fasta -bed viewpoint_coordinates.bed -fo viewpoint_coordinates.fasta

# Extract the reads that overlap with the viewpoint coordinates
bedtools intersect -a sample_4C-seq_data.aligned.bam -b viewpoint_coordinates.bed -wa -wb | samtools view -b -F 4 -q 30 - | samtools sort -o sample_4C-seq_data.viewpoint.bam -
```
3. Interaction identification:
```bash
# Identify genomic interactions originating from the viewpoint
4C-seq -i sample_4C-seq_data.viewpoint.bam -o sample_4C-seq_data.interactions.txt -r reference_genome.fasta -v viewpoint_coordinates.bed -p 4

# Filter the interactions based on criteria such as distance, strength, and specificity
4C-seq -i sample_4C-seq_data.interactions.txt -o sample_4C-seq_data.filtered.txt -r reference_genome.fasta -v viewpoint_coordinates.bed -p 4 -d 1000 -s 0.5 -c 0.5
```
4. Data visualization:
```bash
# Visualize the interactions in a genome browser
igv -g reference_genome.fasta -b sample_4C-seq_data.filtered.txt -o sample_4C-seq_data.filtered.igv

# Create plots of the interactions
matplotlib -i sample_4C-seq_data.filtered.txt -o sample_4C-seq_data.filtered.pdf -r reference_genome.fasta -v viewpoint_coordinates.bed -p 4 -d 1000 -s 0.5 -c 0.5
```
Note that this is just a high-level plan and the specific commands and parameters may need to be adjusted based on the specific requirements of your analysis. Additionally, the `4C-seq` package may require additional installation and configuration steps, which are not covered in this plan.
```

Nanopore DNA sequencing data analysis   Case 8.1: Genome assembly

<table>
<tr><td>User's Inputs</td><td>You are a bioinformatician who specializes in writing code and performing analysis for a variety of bioinformatic data analysis tasks. Now I provide you with an Ubuntu 18.04 system where I save the following data, each line including the data path and data description.

sample_nanopore_reads.fastq: Raw Nanopore sequencing data in fastq format.
eference_genome.fasta: A reference genome in FASTA format, if a closely related reference is available and used as a guide during the assembly

I need you to help me plan what needs to be done in each step of the analysis process, and help me write the code for each step to install softwares, use tools and accomplish the following main goal:

To assemble a genome from the Nanopore sequencing data.</td></tr>
<tr><td>Generated Results by CodeLlama-34B-Instruct</td><td>Assistant: Sure, I'd be happy to help you with that! Here's a general plan for the analysis process:

1. Preprocessing:
* Trim the adapters from the raw Nanopore sequencing data using a tool such as Trimmomatic.
* Remove low-quality reads using a tool such as FastQC.
* Filter out reads that are too short or too long using a tool such as FilterReads.
2. Assembly:
* Use a tool such as Canu or Flye to perform the genome assembly.
* Use the reference genome as a guide for the assembly, if available.
3. Quality control:
* Use a tool such as Quast to evaluate the quality of the assembled genome.
* Use a tool such as BUSCO to assess the completeness of the assembled genome.
4. Annotation:
* Use a tool such as Prokka to annotate the assembled genome.
* Use a tool such as InterProScan to identify protein families and functional domains.
5. Visualization:
* Use a tool such as Circos or UCSC Genome Browser to visualize the assembled genome.

Here's some sample code for each step:

1. Preprocessing:
```bash
Trim adapters from raw Nanopore sequencing data
trimmomatic sample_nanopore_reads.fastq
sample_nanopore_reads_trimmed.fastq

Remove low-quality reads
fastqc sample_nanopore_reads_trimmed.fastq</td></tr>
</table>

Filter out reads that are too short or too long
filterreads -i sample_nanopore_reads_trimmed.fastq -o sample_nanopore_reads_filtered.fastq -minlen 500 -maxlen 1000
```
2. Assembly:
```bash
# Use Canu to perform the genome assembly
canu -d sample_genome -p sample_genome_assembly -s sample_nanopore_reads_filtered.fastq

# Use Flye to perform the genome assembly
flye -d sample_genome -p sample_genome_assembly -s sample_nanopore_reads_filtered.fastq
```
3. Quality control:
```bash
# Use Quast to evaluate the quality of the assembled genome
quast sample_genome_assembly.fasta

# Use BUSCO to assess the completeness of the assembled genome
busco -i sample_genome_assembly.fasta -o sample_genome_assembly_busco.txt
```
4. Annotation:
```bash
# Use Prokka to annotate the assembled genome
prokka sample_genome_assembly.fasta -o sample_genome_annotation.gff

# Use InterProScan to identify protein families and functional domains
interproscan -i sample_genome_annotation.gff -o sample_genome_annotation_interpro.txt
```
5. Visualization:
```bash
# Use Circos to visualize the assembled genome
circos -i sample_genome_assembly.fasta -o sample_genome_assembly_circos.png

# Use UCSC Genome Browser to visualize the assembled genome
ucsc_genome_browser -i sample_genome_assembly.fasta -o sample_genome_assembly_ucsc.png
```
Note that this is just a general plan and the specific tools and parameters used may vary depending on the specific requirements of your analysis.
```

Nanopore DNA sequencing data analysis   Case 8.2: Tandem repeats variation identification

<table>
<tr><td>User’s Inputs</td><td>You are a bioinformatician who specializes in writing code and performing analysis for a variety of bioinformatic data analysis tasks. Now I provide you with an Ubuntu 18.04 system where I save the following data, each line including the data path and data description.

sample_nanopore_reads.fastq: Raw Nanopore sequencing data in fastq format.
eference_genome.fasta: A reference genome in FASTA format, if a closely related reference is available and used as a guide during the assembly

I need you to help me plan what needs to be done in each step of the analysis process, and help me write the code for each step to install softwares, use tools and accomplish the following main goal:

To identify tandem repeats.</td></tr>
<tr><td>Generated Results by CodeLlama-34B-Instruct</td><td>Assistant: Sure, I can help you with that! Here's a step-by-step plan for identifying tandem repeats in the given data:

1. Install necessary software:
* We will need a tool for aligning the sequencing reads to the reference genome. I recommend using `minimap2` for this purpose.
* We will also need a tool for identifying tandem repeats. I recommend using `RepeatMasker` for this purpose.

Here's the code to install these tools:
```bash
sudo apt-get update
sudo apt-get install minimap2
sudo apt-get install repeatmasker
```
2. Align the sequencing reads to the reference genome:
* We will use `minimap2` to align the sequencing reads to the reference genome.
* The command to align the reads is:
```bash
minimap2 -ax map-ont reference_genome.fasta
sample_nanopore_reads.fastq > aligned_reads.sam
```
This will create a SAM file called `aligned_reads.sam` that contains the aligned reads.

3. Identify tandem repeats:
* We will use `RepeatMasker` to identify tandem repeats in the aligned reads.
* The command to run `RepeatMasker` is:
```bash
RepeatMasker -paired -lib -s 1000 -nolow -noint -not -not -not -not -not -not
-not -not -not -not -not -not -not -not -not -not -not -not -not -not -not -not -
not -not -not -not -not -not -not -not -not -not -not -not -not -not -not -not -not
-not -not -not -not -not -not -not -not -not -not -not -not -not -not -not -not -
not -not -not -not -not -not -not -not -not -not -not -not -not -not -not -not -not
-not -not -not -not -not -not -not -not -not -not -not -not -not -not -not -not -</td></tr>
</table>

	not -not -not -not -not -not -not -not -not -not -not -not -not -not -not -not -not<br>-not -not -not -not -not -not -not -not -not -not -not -not -not -not -not -not -<br>not -not -not -not -not -not -not -not -not -not -not -not -not -not -not -not -not<br>-not -not -not -not -not -not -not -not -not -not -not -not -not -not -not -not -<br>not -not -not -not -not -not -not -not -not -not -not -not -not -not -not -not -not<br>-not -not -not -not -not -not -not -not -not -not -not -not -not -not -not -not -<br>not -not -not -not -not -not -not -not -not -not -not -not -not -not -not -not -not<br>-not -not -not -not -not -not -not -not -not -not -not -not -not -not -not -not -<br>not -not -not -not -not -not -not -not -not -not -not -not -not -not -not -not -not<br>-not -not -not -not -not -not -not -not -not -not -not -not -not -not -not -not -<br>not -not -not -not -not -not -not -not -not -not -not -not -not -not -not -not -not<br>-not -not -not -not -not -not -not -not -not -not -not -not -not -not -not -not -<br>not -not -not -not -not -not -not -not -not -not -not -not -not -not -not -not -not<br>-not -not -not -not -not -not -not -not -not -not -not -not -not -not -not -not -<br>not -not -not -not -not -not -not -not -not -not -not -not -not -not -not -not -not<br>-not -not -not -not -not -not -not -not -not -not -not -not -not -not -not -not -<br>not -not -not -not -not -not -not -not -not -not -not -not -not -not -not -not -not<br>-not -not -not -not -not -not -not -not -not -not -not -not -not -not -not -not -<br>not -not -not -not -not -not -not -not -not -not -not -not -not -not -not -not -not<br>-not -not -not -not -not -not -not -not -not -not -not -not -not -not -not -not -<br>not -not -not -not -not -not -not -not -not -not -not -not -not -not -not -not -not<br>-not -not -not -not -not -not -not -not -not -not -not -not -not -not -not -not -<br>not -not -not -not -not -not -not -not -not -not -not -not -not -not -not -not -not<br>-not -not -not -not -not -not -not -not -not -not -not -not -not -not -not -not -<br>not -not -not -not -not -not -not -not -not -not -not -not -not -not -not -not -not<br>-not -not -not -not -not -not -not -not -not -not -not -not -not -not -not -not -<br>not -not -not -not -not -not -not -not -not -not -not -not -not -not -not -not -not<br>-not -not -not -not -not -not -not -not -not -not -not -not -not -not -not -not -<br>not -not -not -not -not -not -not -not -not -not -not -not -not -not -not -not -not<br>-not -not -not -not -not -not -not -not -not -not -not -not -not -not -not -not -<br>not -not -not -not -not -not -not -not -not -not -not -not -not -not -not -not -not<br>-not -not -not -not -not -not -not -not -not -not -not -not -not -not -not -not -<br>not -not -not -not -not -not -not -not -not -not -not -not -not -not -not -not -not<br>-not -not -not -not -not -not -not -not -not -not -not -not -not -not -not -not -<br>not -not -not -not -not -not -not -not -not -not -not -not -not -not -not -not -not<br>-not -not -not -not -not -not -not -not -not -not -not -not -not -not -not -not -<br>not -not -not -not -not -not -not -not -not -not -not -not -not -not -not -not -not<br>-not -not -not -not -not -not -not -not -not -not -not -not -not -not -not -not -<br>not -not -not -not -not -not -not -not -not -not -not -not -not -not -not -not -not<br>-not -not -not -not -not -not -not -not -not -not -not -not -not -not -not -not -<br>not -not -not -not -not -not -not -not -not -not -not -not -not -not -not -not -not<br>-not -not -not -not -not -not -not -not -not -not -not -not -not -not -not -not -<br>not -not -not -not -not -not -not -not -not -not -not -not -not -not -not -not -not<br>-not -not -not -not -not -not -not -not -not -not -not -not -not -not -not -not -<br>not -not -not -not -not -not -not -not -not -not -not -not -not -not -not -not -not<br>-not -not -not -not -not -not -not -not -not -not -not -not -not -not -not -not -<br>not -not -not -not -not -not -not -not -not -not -not -not -not -not -not -not -not<br>-not -not -not -not -not -not -not -not -not -not -not -not -not -not -not -not -<br>not -not -not -not -not -not -not -not -not -not -not -not -not -not -not -not -not<br>-not -not -not -not -not -not -not -not -not -not -not -not -not -not -not -not -<br>not -not -not -not -not -not -not -not -not -not -not -not -not -not -not -not -not<br>-not -not -not -not -not -not -not -not -not -not -not -not -not -not -not -not -<br>not -not -not -not -not -not -not -not -not -not -not -not -not -not -not -not -not

-not -not -not -not -not -not -not -not -not -not -not -not -not -not -not -not -
not -not -not -not -not -not -not -not -not -not -not -not -not -not -not -not -not
-not -not -not -not -not -not -not -not -not -not -not -not -not -not -not -not -
not -not -not -not -not -not -not -not -not -not -not -not -not -not -not -not -not
-not -not -not -not -not -not -not -not -not -not -not -not -not -not -not -not -
not -not -not -not -not -not -not -not -not -not -not -not -not -not -not -not -not
-not -not -not -not -not -not -not -not -not -not -not -not -not -not -not -not -
not -not -not -not -not -not -not -not -not -not -not -not -not -not -not -not -not
-not -not -not -not -not -not -not -not -not -not -not -not -not -not -not -not -
not -not -not -not -not -not -not -not -not -not -not -not -not -not -not -not -not
-not -not -not -not -not -not -not -not -not -not -not -not -not -not -not -not -
not -not -not -not -not -not -not -not -not -not -not -not -not -not -not -not -not
-not -not -not -not -not -not -not -not -not -not -not -not -not -not -not -not -
not -not -not -not -not -not -not -not -not -not -not -not -not -not -not -not -not
-not -not -not -not -not -not -not -not -not -not -not -not -not -not -not -not -
not -not -not -not -not -not -not -not -not -not -not -not -not -not -not -not -not
-not -not -not -not -not -not -not -not -not -not -not -not -not -not -not -not -
not -not -not -not -not -not -not -not -not -not -not -not -not -not -not -not -not
-not -not -not -not -not -not -not -not -not -not -not -not -not -not -not -not -
not -not -not -not -not -not -not -not -not -not -not -not -not -not -not -not -not
-not -not -not -not -not -not -not -not -not -not -not -not -not -not -not -not -
not -not -not -not -not -not -not -not -not -not -not -not -not -not -not -not -not
-not -not -not -not -not -not -not -not -not -not -not -not -not -not -not -not -
not -not -not -not -not -not -not -not -not -not -not -not -not -not -not -not -not
-not -not -not -not -not -not -not -not -not -not -not -not -not -not -not -not -
not -not -not -not -not -not -not -not -not -not -not -not -not -not -not -not -not
-not -not -not -not -not -not -not -not -not -not -not -not -not -not -not -not -
not -not -not -not -not -not -not -not -not -not -not -not -not -not -not -not -not
-not -not -not -not -not -not -not -not -not -not -not -not -not -not -not -not -
not -not -not -not -not -not -not -not -not -not -not -not -not -not -not -not -not
-not -not -not -not -not -not -not -not -not -not -not -not -not -not -not -not -
not -not -not -not -not -not -not -not -not -not -not -not -not -not -not -not -not
-not -not -not -not -not -not -not -not -not -not -not -not -not -not -not -not -
not -not -not -not -not -not -not -not -not -not -not -not -not -not -not -not -not
-not -not -not -not -not -not -not -not -not -not -not -not -not -not -not -not -
not -not -not -not -not -not -not -not -not -not -not -not -not -not -not -not -not
-not -not -not -not -not -not -not -not -not -not -not -not -not -not -not -not -
not -not -not -not -not -not -not -not -not -not -not -not -not -not -not -not -not
-not -not -not -not -not -not -not -not -not -not -not -not -not -not -not -not -
not -not -not -not -not -not -not -not -not -not -not -not -not -not -not -not -not
-not -not -not -not -not -not -not -not -not -not -not -not -not -not -not -not -
not -not -not -not -not -not -not -not -not -not -not -not -not -not -not -not -not
-not -not -not -not -not -not -not -not -not -not -not -not -not -not -not -not -
not -not -not -not -not -not -not -not -not -not -not -not -not -not -not -not -not
-not -not -not -not -not -not -not -not -not -not -not -not -not -not -not -not -
not -not -not -not -not -not -not -not -not -not -not -not -not -not -not -not -not
-not -not -not -not -not -not -not -not -not -not -not -not -not -not -not -not -
not -not -not -not -not -not -not -not -not -not -not -not -not -not -not -not -not
-not -not -not -not -not -not -not -not -not -not -not -not -not -not -not -not -
not -not -not -not -not -not -not -not -not -not -not -not -not -not -not -not -not

PacBio DNA sequencing data analysis **Case 9.1: Genome assembly**

<table>
<tr><td>User's Inputs</td><td>You are a bioinformatician who specializes in writing code and performing analysis for a variety of bioinformatic data analysis tasks. Now I provide you with an Ubuntu 18.04 system where I save the following data, each line including the data path and data description.

sample_pacbio_reads.fastq: Raw PacBio sequencing data in fastq format.
eference_genome.fasta: A reference genome in FASTA format, if a closely related reference is available and used as a guide during the assembly

I need you to help me plan what needs to be done in each step of the analysis process, and help me write the code for each step to install softwares, use tools and accomplish the following main goal:

To assemble a genome from the PacBio sequencing data.</td></tr>
<tr><td>Generated Results by CodeLlama-34B-Instruct</td><td>-> Assistant: Sure, I can help you with that! Here's a high-level plan for the analysis process:

1. Preprocessing:
* Trim the adapters from the PacBio reads using a tool like Trimmomatic.
* Remove low-quality reads using a tool like FastQC.
* Filter out reads that are too short or too long using a tool like SeqKit.
2. Assembly:
* Use a tool like Canu or SPAdes to perform the genome assembly.
* Use the reference genome as a guide for the assembly process.
* Use a tool like BWA to align the assembled contigs to the reference genome.
3. Quality control:
* Use a tool like FastQC to assess the quality of the assembled contigs.
* Use a tool like BUSCO to evaluate the completeness and accuracy of the assembly.
4. Annotation:
* Use a tool like Prokka to annotate the assembled contigs with gene predictions and functional information.
* Use a tool like InterProScan to identify protein families and functional domains in the annotated genes.
5. Visualization:
* Use a tool like Circos to visualize the assembled genome and the annotations.

Here's some sample code for each step:

1. Preprocessing:
```bash
Trim adapters from PacBio reads
trimmomatic PE -threads 4 sample_pacbio_reads.fastq sample_pacbio_reads_trimmed.fastq ILLUMINACLIP:TruSeq3-PE.fa:2:30:10 LEADING:3 TRAILING:3 SLIDINGWINDOW:4:15 MINLEN:36

Remove low-quality reads</td></tr>
</table>

fastqc -t 4 -o sample_pacbio_reads_trimmed.fastq

Filter out reads that are too short or too long
seqkit filter -l 36 -L 100000 -o sample_pacbio_reads_filtered.fastq
sample_pacbio_reads_trimmed.fastq
```
2. Assembly:
```bash
# Perform genome assembly using Canu
canu -d canu_out -p canu genomeSize=50m -pacbio-raw
sample_pacbio_reads_filtered.fastq

# Perform genome assembly using SPAdes
spades.py -t 4 -m 50m -1 sample_pacbio_reads_filtered.fastq -o
spades_out
```
3. Quality control:
```bash
# Assess the quality of the assembled contigs using FastQC
fastqc -t 4 -o spades_out/contigs.fasta

# Evaluate the completeness and accuracy of the assembly using BUSCO
busco -m genome -l pacbio -i spades_out/contigs.fasta -o busco_out
```
4. Annotation:
```bash
# Annotate the assembled contigs using Prokka
prokka --cpus 4 --outdir prokka_out --prefix prokka_out
spades_out/contigs.fasta

# Identify protein families and functional domains in the annotated genes
using InterProScan
interproscan.sh -appl Pfam,SMART,TIGRFAMs -i
prokka_out/prokka_out.faa -o prokka_out/interproscan.tsv
```
5. Visualization:
```bash
# Visualize the assembled genome and the annotations using Circos
circos -conf circos.conf -o circos_out
```
Note that this is just a high-level plan and you may need to modify the code and the tools used based on your specific needs and the characteristics of your data.
```

RNA-Seq Analysis Case 10.1: Find differentially expressed genes

<table>
<tr><td>User's Inputs</td><td>You are a bioinformatician who specializes in writing code and performing analysis for a variety of bioinformatic data analysis tasks. Now I provide you with an Ubuntu 18.04 system where I save the following data, each line including the data path and data description.

SRR1374921.fastq.gz: single-end mouse rna-seq reads, replicate 1 in LoGlu group
SRR1374922.fastq.gz: single-end mouse rna-seq reads, replicate 2 in LoGlu group
SRR1374923.fastq.gz: single-end mouse rna-seq reads, replicate 1 in HiGlu group
SRR1374924.fastq.gz: single-end mouse rna-seq reads, replicate 2 in HiGlu group
TruSeq3-SE.fa: trimming adapter
mm39.fa: mouse mm39 genome fasta
mm39.ncbiRefSeq.gtf: mouse mm39 genome annotation

I need you to help me plan what needs to be done in each step of the analysis process, and help me write the code for each step to install softwares, use tools and accomplish the following main goal:

Find the differentially expressed genes</td></tr>
<tr><td>Generated Results by CodeLlama-34B-Instruct</td><td>Assistant: Sure, I can help you with that! Here's a step-by-step plan for the analysis process:

1. Install necessary software:
* FastQC: A tool for quality control of high-throughput sequencing data.
* Trimmomatic: A tool for trimming adapters and low-quality bases from sequencing reads.
* STAR: A tool for aligning RNA-seq reads to a reference genome.
* HISAT2: A tool for aligning RNA-seq reads to a reference genome.
* StringTie: A tool for assembling and quantifying RNA-seq reads.
* Ballgown: A tool for estimating gene and isoform expression levels.
* DESeq2: A tool for detecting differentially expressed genes.
2. Quality control:
* Use FastQC to assess the quality of the raw sequencing data.
* Use Trimmomatic to trim the adapters and low-quality bases from the sequencing reads.
3. Alignment:
* Use STAR or HISAT2 to align the trimmed sequencing reads to the mouse mm39 genome.
4. Assembly and quantification:
* Use StringTie to assemble and quantify the RNA-seq reads.
* Use Ballgown to estimate the gene and isoform expression levels.
5. Differential expression analysis:</td></tr>
</table>

* Use DESeq2 to detect differentially expressed genes between the LoGlu and HiGlu groups.
6. Data visualization:
    * Use a tool such as ggplot2 or plotly to visualize the results of the differential expression analysis.

Here's some sample code for each step:

1. Installing necessary software:
```bash
sudo apt-get update
sudo apt-get install fastqc trimmomatic star hisat2 stringtie ballgown deseq2
```
2. Quality control:
```bash
fastqc SRR1374921.fastq.gz SRR1374922.fastq.gz SRR1374923.fastq.gz SRR1374924.fastq.gz
```
3. Trimming adapters:
```bash
trimmomatic SE SRR1374921.fastq.gz SRR1374922.fastq.gz SRR1374923.fastq.gz SRR1374924.fastq.gz -adapter TruSeq3-SE.fa -o trimmed_reads.fastq.gz
```
4. Alignment:
```bash
star --runMode alignReads --readFilesIn trimmed_reads.fastq.gz --genomeDir mm39 --outFileNamePrefix aligned_reads
```
5. Assembly and quantification:
```bash
stringtie aligned_reads.bam --outFile assembled_transcripts.gtf --ballgown --gtf mm39.ncbiRefSeq.gtf
```
6. Differential expression analysis:
```bash
deseq2 assembled_transcripts.gtf --group LoGlu --group HiGlu --outFile differentially_expressed_genes.txt
```
7. Data visualization:
```bash
ggplot2(differentially_expressed_genes.txt, aes(x = log2FoldChange, y = pvalue)) + geom_point() + facet_wrap(~group)
```
Note that this is just a sample code and you may need to modify it according to your specific needs and the version of the software you are using.

RNA-Seq Analysis **Case 10.2: Identify top5 down-regulated genes in HiGlu group**

**User's Inputs**	You are a bioinformatician who specializes in writing code and performing analysis for a variety of bioinformatic data analysis tasks. Now I provide you with an Ubuntu 18.04 system where I save the following data, each line including the data path and data description.<br><br>SRR1374921.fastq.gz: single-end mouse rna-seq reads, replicate 1 in LoGlu group<br>SRR1374922.fastq.gz: single-end mouse rna-seq reads, replicate 2 in LoGlu group<br>SRR1374923.fastq.gz: single-end mouse rna-seq reads, replicate 1 in HiGlu group<br>SRR1374924.fastq.gz: single-end mouse rna-seq reads, replicate 2 in HiGlu group<br>TruSeq3-SE.fa: trimming adapter<br>mm39.fa: mouse mm39 genome fasta<br>mm39.ncbiRefSeq.gtf: mouse mm39 genome annotation<br><br>I need you to help me plan what needs to be done in each step of the analysis process, and help me write the code for each step to install softwares, use tools and accomplish the following main goal:<br><br>Identify top5 down-regulated genes in HiGlu group
**Generated Results by CodeLlama-34B-Instruct**	Assistant: Sure, I can help you with that! Here's a step-by-step plan for the analysis process:<br><br>1. Install necessary software:<br>* FastQC: A quality control tool for high-throughput sequencing data.<br>* Trimmomatic: A tool for trimming adapters and low-quality bases from FastQ files.<br>* STAR: A spliced aligner for RNA-seq data.<br>* HISAT2: A spliced aligner for RNA-seq data.<br>* StringTie: A tool for quantifying gene expression from RNA-seq data.<br>* Ballgown: A tool for visualizing and analyzing gene expression data.<br>2. Quality control:<br>* Run FastQC on the raw FastQ files to assess the quality of the sequencing data.<br>* Use Trimmomatic to trim the adapters and low-quality bases from the FastQ files.<br>3. Read alignment:<br>* Use STAR or HISAT2 to align the trimmed FastQ files to the mouse mm39 genome.<br>* Use StringTie to quantify gene expression from the aligned reads.<br>4. Differential gene expression analysis:<br>* Use Ballgown to identify the top 5 down-regulated genes in the HiGlu group.<br><br>Here's the code for each step:<br><br>1. Install necessary software:

```bash
sudo apt-get update
sudo apt-get install fastqc trimmomatic star hisat2 stringtie ballgown
```

2. Quality control:

```bash
fastqc SRR1374921.fastq.gz SRR1374922.fastq.gz SRR1374923.fastq.gz SRR1374924.fastq.gz
trimmomatic PE SRR1374921.fastq.gz SRR1374922.fastq.gz SRR1374923.fastq.gz SRR1374924.fastq.gz
```

3. Read alignment:

```bash
star --runMode alignReads --readFilesIn SRR1374921.fastq.gz SRR1374922.fastq.gz SRR1374923.fastq.gz SRR1374924.fastq.gz --genomeDir mm39 --outFileNamePrefix SRR1374921 --outSAMtype BAM SortedByCoordinate --outSAMunmapped Within --outSAMattributes NH HI NM MD
```

4. Differential gene expression analysis:

```bash
stringtie --fr --rf --ballgown --gtf mm39.ncbiRefSeq.gtf --o SRR1374921.gtf --ballgown-group LoGlu --ballgown-group HiGlu --ballgown-group-name LoGlu --ballgown-group-name HiGlu --ballgown-group-replicate 1 --ballgown-group-replicate 2 --ballgown-group-replicate 1 --ballgown-group-replicate 2 SRR1374921.bam SRR1374922.bam SRR1374923.bam SRR1374924.bam
```

Note that the above code assumes that the input FastQ files are in the current directory, and that the output files will be saved in the current directory. You may need to modify the code to reflect the actual file paths and output file names.

RNA-Seq Analysis   Case 10.3: Predict Fusion genes

<table>
<tr><td>User’s Inputs</td><td>You are a bioinformatician who specializes in writing code and performing analysis for a variety of bioinformatic data analysis tasks. Now I provide you with an Ubuntu 18.04 system where I save the following data, each line including the data path and data description.

rnaseq_1.fastq.gz: RNA-Seq read 1 data (left read)
rnaseq_2.fastq.gz: RNA-Seq read 2 data (right read)
CTAT_HumanFusionLib.mini.dat.gz: a small fusion annotation library
minigenome.fa: small genome sequence consisting of ~750 genes
minigenome.gtf: transcript structure annotations for these genes

I need you to help me plan what needs to be done in each step of the analysis process, and help me write the code for each step to install softwares, use tools and accomplish the following main goal:

Predict Fusion genes</td></tr>
<tr><td>Generated Results by CodeLlama-34B-Instruct</td><td>Assistant: Sure, I can help you with that! Here's a high-level plan for the analysis process:

1. Install necessary software:
    * Install the following software packages:
        + FastQC: for quality control of the RNA-Seq data
        + Trimmomatic: for trimming the adapters from the RNA-Seq data
        + STAR: for aligning the RNA-Seq data to the reference genome
        + HISAT2: for aligning the RNA-Seq data to the reference genome
        + StringTie: for assembling the transcripts and quantifying the expression levels
        + FusionAnnotator: for predicting the fusion genes
2. Quality control of the RNA-Seq data:
    * Use FastQC to assess the quality of the RNA-Seq data
    * Trim the adapters from the RNA-Seq data using Trimmomatic
3. Align the RNA-Seq data to the reference genome:
    * Use STAR or HISAT2 to align the RNA-Seq data to the reference genome
4. Assemble the transcripts and quantify the expression levels:
    * Use StringTie to assemble the transcripts and quantify the expression levels
5. Predict the fusion genes:
    * Use FusionAnnotator to predict the fusion genes

Here's some sample code for each step:

1. Installing necessary software:
```bash
sudo apt-get update
sudo apt-get install fastqc trimmomatic star hisat2 stringtie fusionannotator
```
2. Quality control of the RNA-Seq data:
```bash</td></tr>
</table>

```
fastqc rnaseq_1.fastq.gz rnaseq_2.fastq.gz
```

3. Trimming the adapters from the RNA-Seq data:

```bash
trimmomatic PE rnaseq_1.fastq.gz rnaseq_2.fastq.gz \
  rnaseq_1.trimmed.fastq.gz rnaseq_2.trimmed.fastq.gz \
  ILLUMINACLIP:TruSeq3-PE.fa:2:30:10 LEADING:3 TRAILING:3
SLIDINGWINDOW:4:15 MINLEN:36
```

4. Aligning the RNA-Seq data to the reference genome:

```bash
star --runMode alignReads \
  --readFilesIn rnaseq_1.trimmed.fastq.gz rnaseq_2.trimmed.fastq.gz \
  --readFilesCommand zcat \
  --genomeDir /path/to/reference/genome \
  --outFileNamePrefix rnaseq_aligned
```

5. Assembling the transcripts and quantifying the expression levels:

```bash
stringtie rnaseq_aligned.bam \
  --outFile rnaseq_assembled.gtf \
  --refGeneModel /path/to/reference/genome/genes.gtf \
  --covStats
```

6. Predicting the fusion genes:

```bash
fusionannotator rnaseq_assembled.gtf \
  --fusionLib CTAT_HumanFusionLib.mini.dat.gz \
  --outFile rnaseq_fusions.txt
```

Note that the above code is just a sample and may need to be modified based on your specific needs and the version of the software you are using. Additionally, you may need to install additional software packages or libraries to run the analysis.

RNA-Seq Analysis **Case 10.4: isoform expression**

<table>
<tr><td>User's Inputs</td><td>You are a bioinformatician who specializes in writing code and performing analysis for a variety of bioinformatic data analysis tasks. Now I provide you with an Ubuntu 18.04 system where I save the following data, each line including the data path and data description.

SRR14463651_1.fastq.gz: paired end RNA sequencing data from human lymphoma cell, read 1 of lymphoma samples replicate 1
SRR14463651_2.fastq.gz: paired end RNA sequencing data from human lymphoma cell, read 2 of lymphoma samples replicate 1
SRR14463652_1.fastq.gz: paired end RNA sequencing data from human lymphoma cell, read 1 of lymphoma samples replicate 2
SRR14463652_2.fastq.gz: paired end RNA sequencing data from human lymphoma cell, read 2 of lymphoma samples replicate 2
SRR14463680_1.fastq.gz: paired end RNA sequencing data from human lymphoma cell, read 1 of control samples replicate 1
SRR14463680_2.fastq.gz: paired end RNA sequencing data from human lymphoma cell, read 2 of control samples replicate 1
SRR14463681_1.fastq.gz: paired end RNA sequencing data from human lymphoma cell, read 1 of control samples replicate 2
SRR14463681_2.fastq.gz: paired end RNA sequencing data from human lymphoma cell, read 2 of control samples replicate 2
gencode.v44.transcripts.fa: transcriptome annotation
TruSeq3-PE.fa: trimming adapter

I need you to help me plan what needs to be done in each step of the analysis process, and help me write the code for each step to install softwares, use tools and accomplish the following main goal:

quantify RNA isoform expression based on transcriptome annotation</td></tr>
<tr><td>Generated Results by CodeLlama-34B-Instruct</td><td>-> Assistant: Sure, I can help you with that! Here's a step-by-step plan for the analysis process:

1. Install necessary software:
* FastQC: This tool is used to assess the quality of the raw sequencing data.
* Trimmomatic: This tool is used to trim the adapters from the raw sequencing data.
* STAR: This tool is used to align the trimmed reads to the transcriptome annotation.
* HISAT2: This tool is used to align the trimmed reads to the transcriptome annotation.
* StringTie: This tool is used to assemble the aligned reads into transcripts and quantify their expression.
* Ballgown: This tool is used to visualize the expression of the transcripts.

To install these tools, you can use the following commands:
```bash
sudo apt-get install fastqc trimmomatic star hisat2 stringtie ballgown
```
2. Trim the adapters from the raw sequencing data:</td></tr>
</table>

* Use Trimmomatic to trim the adapters from the raw sequencing data.
* The command to trim the adapters is:

```bash
trimmomatic PE -threads 4 SRR14463651_1.fastq.gz
SRR14463651_2.fastq.gz SRR14463652_1.fastq.gz
SRR14463652_2.fastq.gz SRR14463680_1.fastq.gz
SRR14463680_2.fastq.gz SRR14463681_1.fastq.gz
SRR14463681_2.fastq.gz -adapter TruSeq3-PE.fa -o
trimmed_reads.fastq.gz
```

3. Align the trimmed reads to the transcriptome annotation:
    * Use STAR or HISAT2 to align the trimmed reads to the transcriptome annotation.
    * The command to align the reads using STAR is:

```bash
star --runMode alignReads --runThreadN 4 --genomeDir
gencode.v44.transcripts.fa --readFilesIn trimmed_reads.fastq.gz --
outSAMtype BAM SortedByCoordinate --outSAMunmapped Within --
outSAMattributes NH HI NM MD --outSAMstrandField intronMotif --
outSAMtype BAM SortedByCoordinate --outSAMunmapped Within --
outSAMattributes NH HI NM MD --outSAMstrandField intronMotif --
outSAMtype BAM SortedByCoordinate --outSAMunmapped Within --
outSAMattributes NH HI NM MD --outSAMstrandField intronMotif
```

The command to align the reads using HISAT2 is:

```bash
hisat2 -p 4 --dta -x gencode.v44.transcripts.fa -1 trimmed_reads_1.fastq.gz
-2 trimmed_reads_2.fastq.gz -S alignments.sam
```

4. Assemble the aligned reads into transcripts and quantify their expression:
    * Use StringTie to assemble the aligned reads into transcripts and quantify their expression.
    * The command to assemble the reads using StringTie is:

```bash
stringtie -p 4 -G gencode.v44.transcripts.fa -o assembled_transcripts.gtf
alignments.bam
```

5. Visualize the expression of the transcripts:
    * Use Ballgown to visualize the expression of the transcripts.
    * The command to visualize the expression using Ballgown is:

```bash
ballgown -p 4 -b assembled_transcripts.gtf -o expression_data.txt
alignments.bam
```

This will create a file called `expression_data.txt` that contains the expression levels of the transcripts.

That's it! These are the basic steps for quantifying RNA isoform expression based on transcriptome annotation. Of course, there are many other tools and parameters that you can use to customize the analysis, but this should give you a good starting point.

RNA-seq Analysis Case 10.5: splicing analysis

<table>
<tr><td>User's Inputs</td><td>You are a bioinformatician who specializes in writing code and performing analysis for a variety of bioinformatic data analysis tasks. Now I provide you with an Ubuntu 18.04 system where I save the following data, each line including the data path and data description.

SRR14463651_1.fastq.gz: paired end RNA sequencing data from human lymphoma cell, read 1 of lymphoma samples replicate 1
SRR14463651_2.fastq.gz: paired end RNA sequencing data from human lymphoma cell, read 2 of lymphoma samples replicate 1
SRR14463652_1.fastq.gz: paired end RNA sequencing data from human lymphoma cell, read 1 of lymphoma samples replicate 2
SRR14463652_2.fastq.gz: paired end RNA sequencing data from human lymphoma cell, read 2 of lymphoma samples replicate 2
SRR14463680_1.fastq.gz: paired end RNA sequencing data from human lymphoma cell, read 1 of control samples replicate 1
SRR14463680_2.fastq.gz: paired end RNA sequencing data from human lymphoma cell, read 2 of control samples replicate 1
SRR14463681_1.fastq.gz: paired end RNA sequencing data from human lymphoma cell, read 1 of control samples replicate 2
SRR14463681_2.fastq.gz: paired end RNA sequencing data from human lymphoma cell, read 2 of control samples replicate 2
hg38.fa: reference genome
gencode.v38.annotation.gtf: gene annotation
TruSeq3-PE.fa: trimming adapter

I need you to help me plan what needs to be done in each step of the analysis process, and help me write the code for each step to install softwares, use tools and accomplish the following main goal:

identify alternative splicing events in lymphoma and control samples and compare them to get differentially spliced events</td></tr>
<tr><td>Generated Results by CodeLlama-34B-Instruct</td><td>Assistant: Sure, I can help you with that! Here's a plan for the analysis process and the code for each step:

Step 1: Install necessary software

* FastQC: `sudo apt-get install fastqc`
* Trimmomatic: `sudo apt-get install trimmomatic`
* STAR: `sudo apt-get install star`
* HISAT2: `sudo apt-get install hisat2`
* StringTie: `sudo apt-get install stringtie`
* Ballgown: `sudo apt-get install ballgown`

Step 2: Quality control and trimming

* Run FastQC on all input files to assess quality
* Trim adapters and low-quality reads using Trimmomatic

Step 3: Alignment

* Align reads to the reference genome using STAR</td></tr>
</table>

* Convert the output to BAM format

Step 4: Gene expression quantification

* Use StringTie to estimate gene expression levels
* Use Ballgown to generate a gene-level expression matrix

Step 5: Differential splicing analysis

* Use Ballgown to identify differentially spliced events between lymphoma and control samples
* Use the `diffsplice` function in Ballgown to perform the differential splicing analysis

Step 6: Visualization

* Use the `plot_diffsplice` function in Ballgown to visualize the results

Here's the code for each step:

Step 1: Install necessary software
```bash
sudo apt-get install fastqc trimmomatic star hisat2 stringtie ballgown
```
Step 2: Quality control and trimming
```bash
fastqc -o fastqc_output SRR14463651_1.fastq.gz SRR14463651_2.fastq.gz
SRR14463652_1.fastq.gz SRR14463652_2.fastq.gz
SRR14463680_1.fastq.gz SRR14463680_2.fastq.gz
SRR14463681_1.fastq.gz SRR14463681_2.fastq.gz

trimmomatic PE -threads 4 -phred33 SRR14463651_1.fastq.gz
SRR14463651_2.fastq.gz SRR14463652_1.fastq.gz
SRR14463652_2.fastq.gz SRR14463680_1.fastq.gz
SRR14463680_2.fastq.gz SRR14463681_1.fastq.gz
SRR14463681_2.fastq.gz
```
Step 3: Alignment
```bash
STAR --runMode alignReads --runThreadN 4 --genomeDir /path/to/hg38.fa -
-readFilesIn SRR14463651_1.fastq.gz SRR14463651_2.fastq.gz
SRR14463652_1.fastq.gz SRR14463652_2.fastq.gz
SRR14463680_1.fastq.gz SRR14463680_2.fastq.gz
SRR14463681_1.fastq.gz SRR14463681_2.fastq.gz --outSAMtype BAM
SortedByCoordinate --outSAMunmapped Within --outSAMattributes NH HI
NM MD --outSAMstrandField intronMotif --outSAMtype None

samtools view -bh -o output.bam output.sam
```
Step 4: Gene expression quantification
```bash
stringtie -p 4 -G /path/to/gencode.v38.annotation.gtf -o output.gtf
output.bam
```

	ballgown count -p 4 -o output.counts output.gtf output.bam<br>```<br>Step 5: Differential splicing analysis<br>```bash<br>ballgown diffsplice -p 4 -o output.diffsplice output.counts output.gtf output.bam<br>```<br>Step 6: Visualization<br>```bash<br>ballgown plot_diffsplice -p 4 -o output.pdf output.diffsplice output.counts output.gtf output.bam<br>```<br>Note that this is just a basic plan and you may need to adjust the parameters and options for each step based on your specific data and research question. Additionally, you may need to install additional software or packages to perform certain steps.

RNA-seq Analysis **Case 10.6: APA analysis**

**User's Inputs**	You are a bioinformatician who specializes in writing code and performing analysis for a variety of bioinformatic data analysis tasks. Now I provide you with an Ubuntu 18.04 system where I save the following data, each line including the data path and data description.<br><br>SRR14463651_1.fastq.gz: paired end RNA sequencing data from human lymphoma cell, read 1 of lymphoma samples replicate 1<br>SRR14463651_2.fastq.gz: paired end RNA sequencing data from human lymphoma cell, read 2 of lymphoma samples replicate 1<br>SRR14463652_1.fastq.gz: paired end RNA sequencing data from human lymphoma cell, read 1 of lymphoma samples replicate 2<br>SRR14463652_2.fastq.gz: paired end RNA sequencing data from human lymphoma cell, read 2 of lymphoma samples replicate 2<br>SRR14463680_1.fastq.gz: paired end RNA sequencing data from human lymphoma cell, read 1 of control samples replicate 1<br>SRR14463680_2.fastq.gz: paired end RNA sequencing data from human lymphoma cell, read 2 of control samples replicate 1<br>SRR14463681_1.fastq.gz: paired end RNA sequencing data from human lymphoma cell, read 1 of control samples replicate 2<br>SRR14463681_2.fastq.gz: paired end RNA sequencing data from human lymphoma cell, read 2 of control samples replicate 2<br>hg38.fa: reference genome<br>gencode.v38.annotation.gtf: gene annotation<br>TruSeq3-PE.fa: trimming adapter<br><br>I need you to help me plan what needs to be done in each step of the analysis process, and help me write the code for each step to install softwares, use tools and accomplish the following main goal:<br><br>identify and quantify alternative polyadenylation between lymphoma and control samples
**Generated Results by CodeLlama-34B-Instruct**	Assistant: Sure, I can help you with that! Here's a high-level plan for the analysis process:<br><br>1. Data preprocessing:<br>* Trim the adapters from the raw sequencing data using Trim Galore.<br>* Remove reads with low quality scores or that are too short/long.<br>* Filter out reads that are not properly paired.<br>2. Mapping:<br>* Map the trimmed and filtered reads to the reference genome using STAR or HISAT2.<br>* Sort the mapped reads by coordinate and strand.<br>3. Counting:<br>* Count the number of reads that map to each gene using featureCounts or RSEM.<br>* Normalize the counts using TMM or DESeq2.<br>4. Differential expression analysis:<br>* Use DESeq2 or edgeR to identify differentially expressed genes between lymphoma and control samples.

* Use the DEseq2 package in R to perform the differential expression analysis.

5. Alternative polyadenylation analysis:
    * Use the APAtools package in R to perform the alternative polyadenylation analysis.
    * Use the APAtools package to identify the alternative polyadenylation sites and quantify their abundance.
6. Visualization:
    * Use the ggplot2 package in R to visualize the results, including the distribution of read counts, the differential expression analysis, and the alternative polyadenylation analysis.

Here's some sample code for each step:

1. Data preprocessing:
```R
# Load the Trim Galore package
library(trim.galore)

# Trim the adapters from the raw sequencing data
trim_galore(fastq_file, output_dir = "trimmed_fastq")

# Remove reads with low quality scores or that are too short/long
filter_reads(fastq_file, output_dir = "filtered_fastq", min_quality = 20,
max_length = 50)

# Filter out reads that are not properly paired
filter_reads(fastq_file, output_dir = "filtered_fastq", min_quality = 20,
max_length = 50, paired = TRUE)
```
2. Mapping:
```R
# Load the STAR or HISAT2 package
library(STAR)
library(HISAT2)

# Map the trimmed and filtered reads to the reference genome
star(fastq_file, output_dir = "mapped_reads", reference_genome =
"hg38.fa")

# Sort the mapped reads by coordinate and strand
sort_reads(mapped_reads, output_dir = "sorted_reads", coordinate =
TRUE, strand = TRUE)
```
3. Counting:
```R
# Load the featureCounts or RSEM package
library(featureCounts)
library(RSEM)

# Count the number of reads that map to each gene
featureCounts(sorted_reads, output_dir = "counts", reference_genome =
"hg38.fa")
```

# Normalize the counts using TMM or DESeq2
normalize_counts(counts, output_dir = "normalized_counts", method = "TMM")
```
4. Differential expression analysis:
```R
# Load the DESeq2 package
library(DESeq2)

# Perform the differential expression analysis
deseq(normalized_counts, output_dir = "deseq_results", design = ~ condition)
```
5. Alternative polyadenylation analysis:
```R
# Load the APAtools package
library(APAtools)

# Perform the alternative polyadenylation analysis
apa(deseq_results, output_dir = "apa_results", reference_genome = "hg38.fa")
```
6. Visualization:
```R
# Load the ggplot2 package
library(ggplot2)

# Visualize the results
ggplot(apa_results, aes(x = condition, y = count, fill = condition)) +
  geom_bar(stat = "identity", position = "dodge") +
  theme_bw() +
  labs(x = "Condition", y = "Count", fill = "Condition")
```
Note that this is just a high-level plan, and you may need to modify the code and the analysis pipeline based on your specific needs and the characteristics of your data.
```

RNA-seq Analysis   Case 10.7: RNA editing

<table>
<tr>
<td>User's Inputs</td>
<td>You are a bioinformatician who specializes in writing code and performing analysis for a variety of bioinformatic data analysis tasks. Now I provide you with an Ubuntu 18.04 system where I save the following data, each line including the data path and data description.

SRR14463651_1.fastq.gz: paired end RNA sequencing data from human lymphoma cell, read 1 of lymphoma samples replicate 1
SRR14463651_2.fastq.gz: paired end RNA sequencing data from human lymphoma cell, read 2 of lymphoma samples replicate 1
SRR14463652_1.fastq.gz: paired end RNA sequencing data from human lymphoma cell, read 1 of lymphoma samples replicate 2
SRR14463652_2.fastq.gz: paired end RNA sequencing data from human lymphoma cell, read 2 of lymphoma samples replicate 2
SRR14463680_1.fastq.gz: paired end RNA sequencing data from human lymphoma cell, read 1 of control samples replicate 1
SRR14463680_2.fastq.gz: paired end RNA sequencing data from human lymphoma cell, read 2 of control samples replicate 1
SRR14463681_1.fastq.gz: paired end RNA sequencing data from human lymphoma cell, read 1 of control samples replicate 2
SRR14463681_2.fastq.gz: paired end RNA sequencing data from human lymphoma cell, read 2 of control samples replicate 2
hg38.fa: reference genome
gencode.v38.annotation.gtf: gene annotation
TruSeq3-PE.fa: trimming adapter

I need you to help me plan what needs to be done in each step of the analysis process, and help me write the code for each step to install softwares, use tools and accomplish the following main goal:

identifying RNA edting events in lymphoma and control samples</td>
</tr>
<tr>
<td>Generated Results by CodeLlama-34B-Instruct</td>
<td>## Step 1

Assistant: Sure, I can help you with that! Here's a high-level plan for the analysis process:

1. Install necessary software:
* FastQC: for quality control of raw sequencing data
* Trimmomatic: for adapter trimming and quality trimming of raw sequencing data
* STAR: for aligning trimmed reads to the reference genome
* HISAT2: for aligning trimmed reads to the reference genome
* StringTie: for quantifying gene expression
* Ballgown: for visualizing and interpreting RNA editing events
2. Quality control:
* Use FastQC to assess the quality of the raw sequencing data
* Use Trimmomatic to trim the raw sequencing data and remove low-quality reads
3. Read alignment:
* Use STAR or HISAT2 to align the trimmed reads to the reference genome
* Use StringTie to quantify gene expression
4. Identifying RNA editing events:</td>
</tr>
</table>

* Use Ballgown to identify RNA editing events in the lymphoma and control samples
* Use the RNA editing events to identify differentially edited genes between the lymphoma and control samples

5. Visualization and interpretation:
   * Use Ballgown to visualize the RNA editing events and differentially edited genes
   * Use other tools such as UCSC Genome Browser or Integrative Genomics Viewer (IGV) to visualize the genomic context of the RNA editing events

Here's some sample code for each step:

1. Installing necessary software:
```bash
sudo apt-get update
sudo apt-get install fastqc trimmomatic star hisat2 stringtie ballgown
```
2. Quality control:
```bash
fastqc SRR14463651_1.fastq.gz SRR14463651_2.fastq.gz
SRR14463652_1.fastq.gz SRR14463652_2.fastq.gz
SRR14463680_1.fastq.gz SRR14463680_2.fastq.gz
SRR14463681_1.fastq.gz SRR14463681_2.fastq.gz
```
3. Read alignment:
```bash
# Align reads using STAR
STAR --runThreadN 4 --genomeDir hg38 --readFilesIn
SRR14463651_1.fastq.gz SRR14463651_2.fastq.gz
SRR14463652_1.fastq.gz SRR14463652_2.fastq.gz
SRR14463680_1.fastq.gz SRR14463680_2.fastq.gz
SRR14463681_1.fastq.gz SRR14463681_2.fastq.gz --outFileNamePrefix
aligned_reads

# Align reads using HISAT2
hisat2 -p 4 -x hg38 -1 SRR14463651_1.fastq.gz -2
SRR14463651_2.fastq.gz -U SRR14463652_1.fastq.gz -V
SRR14463652_2.fastq.gz -U SRR14463680_1.fastq.gz -V
SRR14463680_2.fastq.gz -U SRR14463681_1.fastq.gz -V
SRR14463681_2.fastq.gz -S aligned_reads
```
4. Identifying RNA editing events:
```bash
# Quantify gene expression using StringTie
stringtie -p 4 -G hg38.fa -o gene_expression.tsv aligned_reads

# Identify RNA editing events using Ballgown
ballgown -p 4 -g hg38.fa -o rna_editing_events.tsv -e gene_expression.tsv
aligned_reads
```
5. Visualization and interpretation:
```bash
```

	# Visualize RNA editing events using Ballgown<br>ballgown -p 4 -g hg38.fa -o rna_editing_events.tsv -e gene_expression.tsv aligned_reads<br><br># Visualize RNA editing events using UCSC Genome Browser<br>ucsc_genome_browser -p 4 -g hg38.fa -o rna_editing_events.tsv -e gene_expression.tsv aligned_reads<br><br># Visualize RNA editing events using Integrative Genomics Viewer (IGV)<br>igv -p 4 -g hg38.fa -o rna_editing_events.tsv -e gene_expression.tsv aligned_reads<br>```<br>Note that this is just a high-level plan and the specific commands and parameters may need to be adjusted based on the specific requirements of your analysis. Additionally, this is just one possible approach and there are many other tools and methods that can be used for RNA editing event detection and analysis.<br><br>--------------

RNA-seq Analysis **Case 10.8: circular RNA analysis**

<table>
<tr><td>User's Inputs</td><td>You are a bioinformatician who specializes in writing code and performing analysis for a variety of bioinformatic data analysis tasks. Now I provide you with an Ubuntu 18.04 system where I save the following data, each line including the data path and data description.

SRR14463651_1.fastq.gz: paired end RNA sequencing data from human lymphoma cell, read 1 of lymphoma samples replicate 1
SRR14463651_2.fastq.gz: paired end RNA sequencing data from human lymphoma cell, read 2 of lymphoma samples replicate 1
SRR14463652_1.fastq.gz: paired end RNA sequencing data from human lymphoma cell, read 1 of lymphoma samples replicate 2
SRR14463652_2.fastq.gz: paired end RNA sequencing data from human lymphoma cell, read 2 of lymphoma samples replicate 2
SRR14463680_1.fastq.gz: paired end RNA sequencing data from human lymphoma cell, read 1 of control samples replicate 1
SRR14463680_2.fastq.gz: paired end RNA sequencing data from human lymphoma cell, read 2 of control samples replicate 1
SRR14463681_1.fastq.gz: paired end RNA sequencing data from human lymphoma cell, read 1 of control samples replicate 2
SRR14463681_2.fastq.gz: paired end RNA sequencing data from human lymphoma cell, read 2 of control samples replicate 2
hg38.fa: reference genome
gencode.v38.annotation.gtf: gene annotation
TruSeq3-PE.fa: trimming adapter

I need you to help me plan what needs to be done in each step of the analysis process, and help me write the code for each step to install softwares, use tools and accomplish the following main goal:

identify and quantify circular RNA in lymphoma and control samples</td></tr>
<tr><td>Generated Results by CodeLlama-34B-Instruct</td><td>## Step 1
Assistant: Sure, I can help you with that! Here's a step-by-step plan for the analysis process:

1. Install necessary software:
* FastQC: This tool is used to assess the quality of the raw sequencing data.
* Trimmomatic: This tool is used to trim the adapters from the raw sequencing data.
* STAR: This tool is used to align the trimmed sequencing data to the reference genome.
* HISAT2: This tool is used to align the trimmed sequencing data to the reference genome.
* Cufflinks: This tool is used to quantify the expression of circular RNA.
* Circular RNA Finder: This tool is used to identify circular RNA.

Here's the code to install these tools:
```bash
sudo apt-get install fastqc trimmomatic star hisat2 cufflinks circular-rna-finder
```</td></tr>
</table>

2. Assess the quality of the raw sequencing data using FastQC:
* Run FastQC on the raw sequencing data to assess the quality of the data.
* Use the following command to run FastQC:

```bash
fastqc SRR14463651_1.fastq.gz SRR14463651_2.fastq.gz
SRR14463652_1.fastq.gz SRR14463652_2.fastq.gz
SRR14463680_1.fastq.gz SRR14463680_2.fastq.gz
SRR14463681_1.fastq.gz SRR14463681_2.fastq.gz
```

3. Trim the adapters from the raw sequencing data using Trimmomatic:
* Use Trimmomatic to trim the adapters from the raw sequencing data.
* Use the following command to run Trimmomatic:

```bash
trimmomatic SRR14463651_1.fastq.gz SRR14463651_2.fastq.gz
SRR14463652_1.fastq.gz SRR14463652_2.fastq.gz
SRR14463680_1.fastq.gz SRR14463680_2.fastq.gz
SRR14463681_1.fastq.gz SRR14463681_2.fastq.gz -adapter TruSeq3-
PE.fa -o trimmed_data.fastq.gz
```

4. Align the trimmed sequencing data to the reference genome using STAR:
* Use STAR to align the trimmed sequencing data to the reference genome.
* Use the following command to run STAR:

```bash
star --runMode alignReads --readFilesIn trimmed_data.fastq.gz --
genomeDir hg38 --sjdbGTFfile gencode.v38.annotation.gtf --
outSAMunmapped Within --outSAMtype BAM SortedByCoordinate --
outSAMattrRGline ID:1 --outSAMattrRGline SM:sample1 --
outSAMattrRGline LB:library1 --outSAMattrRGline PL:illumina --
outSAMattrRGline PU:unit1 --outSAMattrRGline CN:center1 --
outSAMattrRGline DS:description1 --outSAMattrRGline DT:2022-02-
28T12:00:00 --outSAMattrRGline PI:300 --outSAMattrRGline PU:unit1 --
outSAMattrRGline CN:center1 --outSAMattrRGline DS:description1 --
outSAMattrRGline DT:2022-02-28T12:00:00 --outSAMattrRGline PI:300 --
outSAMattrRGline PU:unit1 --outSAMattrRGline CN:center1 --
outSAMattrRGline DS:description1 --outSAMattrRGline DT:2022-02-
28T12:00:00 --outSAMattrRGline PI:300 --outSAMattrRGline PU:unit1 --
outSAMattrRGline CN:center1 --outSAMattrRGline DS:description1 --
outSAMattrRGline DT:2022-02-28T12:00:00 --outSAMattrRGline PI:300 --
outSAMattrRGline PU:unit1 --outSAMattrRGline CN:center1 --
outSAMattrRGline DS:description1 --outSAMattrRGline DT:2022-02-
28T12:00:00 --outSAMattrRGline PI:300 --outSAMattrRGline PU:unit1 --
outSAMattrRGline CN:center1 --outSAMattrRGline DS:description1 --
outSAMattrRGline DT:2022-02-28T12:00:00 --outSAMattrRGline PI:300 --
outSAMattrRGline PU:unit1 --outSAMattrRGline CN:center1 --
outSAMattrRGline DS:description1 --outSAMattrRGline DT:2022-02-
28T12:00:00 --outSAMattrRGline PI:300 --outSAMattrRGline PU:unit1 --
outSAMattrRGline CN:center1 --outSAMattrRGline DS:description1 --
outSAMattrRGline DT:2022-02-28T12:00:00 --outSAMattrRGline PI:300 --
outSAMattrRGline PU:unit1 --outSAMattrRGline CN:center1 --
outSAMattrRGline DS:description1 --outSAMattrRGline DT:2022-02-
```

	28T12:00:00 --outSAMattrRGline PI:300 --outSAMattrRGline PU:unit1 --outSAMattrRGline CN:center1 --outSAMattrRGline DS:description1 --outSAMattrRGline DT:2022-02-28T12:00:00 --outSAMattrRGline PI:300 --outSAMattrRGline PU:unit1 --outSAMattrRGline CN:center1 --outSAMattrRGline DS:description1 --outSAMattrRGline DT:2022-02-28T12:00:00 --outSAMattrRGline PI:300 --outSAMattrRGline PU:unit1 --outSAMattrRGline CN:center1 --outSAMattrRGline DS:description1 --outSAMattrRGline DT:2022-02-28T12:00:00 --outSAMattrRGline PI:300 --outSAMattrRGline PU:unit1 --outSAMattrRGline CN:center1 --outSAMattrRGline DS:description1 --outSAMattrRGline DT:2022-02-28T12:00:00 --outSAMattrRGline PI:300 --outSAMattrRGline PU:unit1 --outSAMattrRGline CN:center1 --outSAMattrRGline DS:description1 --outSAMattrRGline DT:2022-02-28T12:00:00 --outSAMattrRGline PI:300 --outSAMattrRGline PU:unit1 --outSAMattrRGline CN:center1 --outSAMattrRGline DS:description1 --outSAMattrRGline DT:2022-02-28T12:00:00 --outSAMattrRGline PI:300 --outSAMattrRGline PU:unit1 --outSAMattrRGline CN:center1 --outSAMattrRGline DS:description1 --outSAMattrRGline DT:2022-02-28T12:00:00 --outSAMattrRGline PI:300 --outSAMattrRGline PU:unit1 --outSAMattrRGline CN:center1 --outSAMattrRGline DS:description1 --outSAMattrRGline DT:2022-02-28T12:00:00 --outSAMattrRGline PI:300 --outSAMattrRGline PU:unit1 --outSAMattrRGline CN:center1 --outSAMattrRGline DS:description1 --outSAMattrRGline DT:2022-02-28T12:00:00 --outSAMattrRGline PI:300 --outSAMattrRGline PU:unit1 --outSAMattrRGline CN:center1 --outSAMattrRGline DS:description1 --outSAMattrRGline DT:2022-02-28T12:00:00 --outSAMattrRGline PI:300 --outSAMattrRGline PU:unit1 --outSAMattrRGline CN:center1 --outSAMattrRGline DS:description1 --outSAMattrRGline DT:2022-02-28T12:00:00 --outSAMattrRGline PI:300 --outSAMattrRGline PU:unit1 --outSAMattrRGline CN:center1 --outSAMattrRGline DS:description1 --outSAMattrRGline DT:2022-02-28T12:00:00 --outSAMattrRGline PI:300 --outSAMattrRGline PU:unit1 --outSAMattrRGline CN:center1 --outSAMattrRGline DS:description1 --outSAMattrRGline DT:2022-02-28T12:00:00 --outSAMattrRGline PI:300 --outSAMattrRGline PU:unit1 --outSAMattrRGline CN:center1 --outSAMattrRGline DS:description1 --outSAMattrRGline DT:2022-02-28T12:00:00 --outSAMattrRGline PI:300 --outSAMattrRGline PU:unit1 --outSAMattrRGline CN:center1 --outSAMattrRGline DS:description1 --outSAMattrRGline DT:2022-02-28T12:00:00 --outSAMattrRGline PI:300 --outSAMattrRGline PU:unit1 --outSAMattrRGline CN:center1 --outSAMattrRGline DS:description1 --outSAMattrRGline DT:2022-02-28T12:00:00 --outSAMattrRGline PI:300 --outSAMattrRGline PU:unit1 --outSAMattrRGline CN:center1 --outSAMattrRGline DS:description1 --outSAMattrRGline DT:2022-02-28T12:00:00 --outSAMattrRGline PI:300 --outSAMattrRGline PU:unit1 --outSAMattrRGline CN:center1 --outSAMattrRGline DS:description1 --outSAMattrRGline DT:2022-02-28T12:00:00 --outSAMattrRGline PI:300 --outSAMattrRGline PU:unit1 --outSAMattrRGline CN:center1 --outSAMattrRGline DS:description1 --outSAMattrRGline DT:2022-02-28T12:00:00 --outSAMattrRGline PI:300 --outSAMattrRGline PU:unit1 --outSAMattrRGline CN:center1 --outSAMattrRGline DS:description1 --outSAMattrRGline DT:2022-02-28T12:00:00 --outSAMattrRGline PI:300 --

	outSAMattrRGline PU:unit1 --outSAMattrRGline CN:center1 --outSAMattrRGline DS:description1 --outSAMattrRGline DT:2022-02-28T12:00:00 --outSAMattrRGline PI:300 --outSAMattrRGline PU:unit1 --outSAMattrRGline CN:center1 --outSAMattrRGline DS:description1 --outSAMattrRGline DT:2022-02-28T12:00:00 --outSAMattrRGline PI:300 --outSAMattrRGline PU:unit1 --outSAMattrRGline CN:center1 --outSAMattrRGline DS:description1 --outSAMattrRGline DT:2022-02-28T12:00:00 --outSAMattrRGline PI:300 --outSAMattrRG

Small RNA-seq Analysis **Case 11.1 and Case 11.2: miRNA analysis**

**User's Inputs**	You are a bioinformatician who specializes in writing code and performing analysis for a variety of bioinformatic data analysis tasks. Now I provide you with an Ubuntu 18.04 system where I save the following data, each line including the data path and data description.<br><br>SRR10915820.fastq.gz: single end small RNA sequencing data from human cell line, control samples replicate 1<br>SRR10915821.fastq.gz: single end small RNA sequencing data from human cell line, control samples replicate 1<br>SRR10915822.fastq.gz: single end small RNA sequencing data from human cell line, control samples replicate 1<br>SRR10915823.fastq.gz: single end small RNA sequencing data from human cell line, control samples replicate 1<br>hsa.gff3: microRNA genomic coordinate from miRbase<br>TGGAATTCTCGGGTGCCAAGG: adaptor sequence<br>hg38.fa: the reference genome<br><br>I need you to help me plan what needs to be done in each step of the analysis process, and help me write the code for each step to install softwares, use tools and accomplish the following main goal:<br><br>quantify microRNA expression based on miRbase and predict novel microRNA
**Generated Results by CodeLlama-34B-Instruct**	Assistant: Sure, I can help you with that! Here's a general plan for the analysis process:<br><br>1. Install necessary software:<br>* FastQC: for quality control of the sequencing data<br>* Trimmomatic: for adapter removal and quality trimming<br>* STAR: for aligning the trimmed reads to the reference genome<br>* HISAT2: for aligning the trimmed reads to the reference genome<br>* Cufflinks: for quantifying microRNA expression<br>* miRDeep2: for predicting novel microRNAs<br>2. Quality control:<br>* Use FastQC to assess the quality of the sequencing data<br>* Use Trimmomatic to remove adapters and low-quality reads<br>3. Alignment:<br>* Use STAR or HISAT2 to align the trimmed reads to the reference genome<br>4. Quantification:<br>* Use Cufflinks to quantify microRNA expression<br>5. Prediction of novel microRNAs:<br>* Use miRDeep2 to predict novel microRNAs<br><br>Here's some sample code for each step:<br><br>1. Installing necessary software:<br>```bash<br>sudo apt-get update<br>sudo apt-get install fastqc trimmomatic star hisat2 cufflinks mirdeep2<br>```<br>2. Quality control:

```bash
fastqc SRR10915820.fastq.gz SRR10915821.fastq.gz SRR10915822.fastq.gz SRR10915823.fastq.gz
```

3. Adapter removal and quality trimming:

```bash
trimmomatic SE SRR10915820.fastq.gz SRR10915821.fastq.gz SRR10915822.fastq.gz SRR10915823.fastq.gz
```

4. Alignment:

```bash
star --runMode alignReads --readFilesIn SRR10915820.fastq.gz SRR10915821.fastq.gz SRR10915822.fastq.gz SRR10915823.fastq.gz --readFilesCommand zcat --genomeDir hg38 --outSAMunmapped Within --outSAMtype BAM SortedByCoordinate --outSAMattributes NH HI NM MD
```

5. Quantification:

```bash
cufflinks -o cufflinks_output -p 4 -u -b hg38.fa SRR10915820.fastq.gz SRR10915821.fastq.gz SRR10915822.fastq.gz SRR10915823.fastq.gz
```

6. Prediction of novel microRNAs:

```bash
mirdeep2 -t 4 -i SRR10915820.fastq.gz SRR10915821.fastq.gz SRR10915822.fastq.gz SRR10915823.fastq.gz -r hsa.gff3 -o mirdeep2_output
```

Note that the above code is just a sample and may need to be modified based on your specific needs and the version of the software you are using. Additionally, you may need to adjust the parameters for each step based on the specific requirements of your analysis.

CAGE-seq Analysis   Case 12.1: TSS analysis

<table>
<tr>
<td>User’s Inputs</td>
<td>You are a bioinformatician who specializes in writing code and performing analysis for a variety of bioinformatic data analysis tasks. Now I provide you with an Ubuntu 18.04 system where I save the following data, each line including the data path and data description.

SRR11351697.fastq.gz: CAGE-seq data from human cell line, control samples replicate 1
SRR11351698.fastq.gz: CAGE-seq data from human cell line, control samples replicate 2
SRR11351700.fastq.gz: CAGE-seq data from human cell line, treatment samples replicate 1
SRR11351701.fastq.gz: CAGE-seq data from human cell line, treatment samples replicate 1
hg38.fa: the human reference genome
gencode.v37.annotation.gtf: gene annotation
TruSeq3-SE.fa: trimming adapter

I need you to help me plan what needs to be done in each step of the analysis process, and help me write the code for each step to install softwares, use tools and accomplish the following main goal:

Identify transcription start site (TSS) in the cell line based on CAGE-seq data</td>
</tr>
<tr>
<td>Generated Results by CodeLlama-34B-Instruct</td>
<td>Assistant: Sure, I can help you with that! Here's a step-by-step plan for the analysis process:

1. Install necessary software:
    * FastQC: a quality control tool for high-throughput sequencing data.
    * Trimmomatic: a tool for trimming adapters and low-quality bases from FastQ files.
    * HISAT2: a spliced aligner for RNA-seq data.
    * StringTie: a tool for quantifying gene expression and inferring transcripts from RNA-seq data.
    * Ballgown: a tool for visualizing and analyzing RNA-seq data.

To install these software, you can use the following commands:
```bash
sudo apt-get update
sudo apt-get install fastqc trimmomatic hisat2 stringtie ballgown
```
2. Quality control:
    * Run FastQC on the raw FastQ files to assess the quality of the sequencing data.
    * Use the following command to run FastQC on the raw FastQ files:
```bash
fastqc SRR11351697.fastq.gz SRR11351698.fastq.gz
SRR11351700.fastq.gz SRR11351701.fastq.gz
```
3. Adapter trimming:</td>
</tr>
</table>

	* Use Trimmomatic to trim adapters and low-quality bases from the raw FastQ files.<br>* Use the following command to trim adapters and low-quality bases from the raw FastQ files:<br>```bash<br>trimmomatic PE -threads 4 SRR11351697.fastq.gz SRR11351698.fastq.gz SRR11351700.fastq.gz SRR11351701.fastq.gz<br>```<br>4. Alignment:<br>* Use HISAT2 to align the trimmed FastQ files to the human reference genome.<br>* Use the following command to align the trimmed FastQ files to the human reference genome:<br>```bash<br>hisat2 -p 4 -x hg38.fa -1 SRR11351697.fastq.gz -2 SRR11351698.fastq.gz -U SRR11351700.fastq.gz -V SRR11351701.fastq.gz<br>```<br>5. Quantification:<br>* Use StringTie to quantify gene expression and infer transcripts from the aligned data.<br>* Use the following command to quantify gene expression and infer transcripts from the aligned data:<br>```bash<br>stringtie -p 4 -G hg38.fa -o stringtie.gtf -A stringtie.tsv -B stringtie.bed -C stringtie.cds -D stringtie.intron -E stringtie.exon -F stringtie.gene -G stringtie.gene_name -H stringtie.gene_id -I stringtie.transcript_id -J stringtie.transcript_name -K stringtie.transcript_id -L stringtie.transcript_name -M stringtie.transcript_id -N stringtie.transcript_name -O stringtie.transcript_id -P stringtie.transcript_name -Q stringtie.transcript_id -R stringtie.transcript_name -S stringtie.transcript_id -T stringtie.transcript_name -U stringtie.transcript_id -V stringtie.transcript_name -W stringtie.transcript_id -X stringtie.transcript_name -Y stringtie.transcript_id -Z stringtie.transcript_name<br>```<br>6. Visualization:<br>* Use Ballgown to visualize and analyze the quantification results.<br>* Use the following command to visualize and analyze the quantification results:<br>```bash<br>ballgown -p 4 -i stringtie.gtf -o ballgown.html<br>```<br>7. Identify TSS:<br>* Use the Ballgown output to identify the transcription start site (TSS) in the cell line.<br>* Use the following command to identify the TSS in the cell line:<br>```bash<br>ballgown -p 4 -i stringtie.gtf -o ballgown.html -t TSS<br>```<br>This will generate a HTML file named `ballgown.html` that contains the TSS information.

	Note: The above commands are just examples and may need to be modified based on your specific needs and the version of the software you are using.

3'end-seq Analysis Case 13.1: APA analysis based on 3'end-seq data

<table>
<tr><td>User's Inputs</td><td>You are a bioinformatician who specializes in writing code and performing analysis for a variety of bioinformatic data analysis tasks. Now I provide you with an Ubuntu 18.04 system where I save the following data, each line including the data path and data description.

SRR17422754.fastq.gz: Lexongen QuantSeq 3 prime-end RNA sequencing data from human cell line THLE2, healthy liver cell line replicate 1
SRR17422755.fastq.gz: Lexongen QuantSeq 3 prime-end RNA sequencing data from human cell line THLE2, healthy liver cell line replicate 2
SRR17422756.fastq.gz: Lexongen QuantSeq 3 prime-end RNA sequencing data from human cell line SNU398, liver cancer cell line replicate 1
SRR17422757.fastq.gz: Lexongen QuantSeq 3 prime-end RNA sequencing data from human cell line SNU398, liver cancer cell line replicate 2
hg38.fa: the human reference genome
gencode.v37.annotation.gtf: gene annotation
TruSeq3-SE.fa: trimming adapter

I need you to help me plan what needs to be done in each step of the analysis process, and help me write the code for each step to install softwares, use tools and accomplish the following main goal:

Identify polyadenylation sites and quantify alternative polyadenylation based on 3'-end sequencing data</td></tr>
<tr><td>Generated Results by CodeLlama-34B-Instruct</td><td>Assistant: Sure, I can help you with that! Here's a high-level plan for the analysis process:

1. Install necessary software and tools:
* Install the following software and tools:
+ FastQC: for quality control of the sequencing data
+ Trimmomatic: for adapter trimming
+ STAR: for aligning the trimmed reads to the reference genome
+ HISAT2: for aligning the trimmed reads to the reference genome
+ StringTie: for assembling the transcripts and quantifying the abundance of transcripts
+ Cufflinks: for quantifying the abundance of transcripts
+ Ballgown: for visualizing the results
2. Quality control of the sequencing data:
* Use FastQC to assess the quality of the sequencing data
* Check for any sequencing artifacts, such as adapter contamination
* Trim the adapters from the sequencing reads using Trimmomatic
3. Align the trimmed reads to the reference genome:
* Use STAR or HISAT2 to align the trimmed reads to the reference genome
* Use StringTie to assemble the transcripts and quantify the abundance of transcripts
* Use Cufflinks to quantify the abundance of transcripts
4. Identify polyadenylation sites and quantify alternative polyadenylation:
* Use Ballgown to visualize the results and identify polyadenylation sites</td></tr>
</table>

	* Use the visualization tools in Ballgown to quantify alternative polyadenylation<br>5. Interpret the results:<br>* Interpret the results and identify any differences in polyadenylation between the healthy liver cell line and the liver cancer cell line<br>* Discuss the implications of these differences in the context of the disease<br><br>Here's some sample code for each step of the analysis process:<br><br>1. Installing necessary software and tools:<br>```bash<br># Install FastQC<br>sudo apt-get install fastqc<br><br># Install Trimmomatic<br>sudo apt-get install trimmomatic<br><br># Install STAR<br>sudo apt-get install star<br><br># Install HISAT2<br>sudo apt-get install hisat2<br><br># Install StringTie<br>sudo apt-get install stringtie<br><br># Install Cufflinks<br>sudo apt-get install cufflinks<br><br># Install Ballgown<br>sudo apt-get install ballgown<br>```<br>2. Quality control of the sequencing data:<br>```bash<br># Run FastQC on the sequencing data<br>fastqc SRR17422754.fastq.gz SRR17422755.fastq.gz SRR17422756.fastq.gz SRR17422757.fastq.gz<br><br># Check for any sequencing artifacts, such as adapter contamination<br>fastqc --adapter SRR17422754.fastq.gz SRR17422755.fastq.gz SRR17422756.fastq.gz SRR17422757.fastq.gz<br><br># Trim the adapters from the sequencing reads using Trimmomatic<br>trimmomatic SRR17422754.fastq.gz SRR17422755.fastq.gz SRR17422756.fastq.gz SRR17422757.fastq.gz<br>```<br>3. Align the trimmed reads to the reference genome:<br>```bash<br># Align the trimmed reads to the reference genome using STAR<br>star --genome hg38 --readFilesIn SRR17422754.fastq.gz SRR17422755.fastq.gz SRR17422756.fastq.gz SRR17422757.fastq.gz --readFilesCommand zcat --outSAMunmapped Within --outSAMtype BAM

	SortedByCoordinate --outSAMattrRGline ID:1 --outSAMattrRGline SM:2 --outSAMattrRGline LB:3 --outSAMattrRGline PL:4 --outSAMattrRGline PU:5 --outSAMattrRGline CN:6 --outSAMattrRGline DS:7 --outSAMattrRGline DT:8 --outSAMattrRGline PG:9 --outSAMattrRGline PI:10 --outSAMattrRGline PB:11 --outSAMattrRGline PP:12 --outSAMattrRGline PT:13 --outSAMattrRGline PM:14 --outSAMattrRGline PN:15 --outSAMattrRGline PE:16 --outSAMattrRGline PF:17 --outSAMattrRGline PG:18 --outSAMattrRGline PI:19 --outSAMattrRGline PB:20 --outSAMattrRGline PP:21 --outSAMattrRGline PT:22 --outSAMattrRGline PM:23 --outSAMattrRGline PN:24 --outSAMattrRGline PE:25 --outSAMattrRGline PF:26 --outSAMattrRGline PG:27 --outSAMattrRGline PI:28 --outSAMattrRGline PB:29 --outSAMattrRGline PP:30 --outSAMattrRGline PT:31 --outSAMattrRGline PM:32 --outSAMattrRGline PN:33 --outSAMattrRGline PE:34 --outSAMattrRGline PF:35 --outSAMattrRGline PG:36 --outSAMattrRGline PI:37 --outSAMattrRGline PB:38 --outSAMattrRGline PP:39 --outSAMattrRGline PT:40 --outSAMattrRGline PM:41 --outSAMattrRGline PN:42 --outSAMattrRGline PE:43 --outSAMattrRGline PF:44 --outSAMattrRGline PG:45 --outSAMattrRGline PI:46 --outSAMattrRGline PB:47 --outSAMattrRGline PP:48 --outSAMattrRGline PT:49 --outSAMattrRGline PM:50 --outSAMattrRGline PN:51 --outSAMattrRGline PE:52 --outSAMattrRGline PF:53 --outSAMattrRGline PG:54 --outSAMattrRGline PI:55 --outSAMattrRGline PB:56 --outSAMattrRGline PP:57 --outSAMattrRGline PT:58 --outSAMattrRGline PM:59 --outSAMattrRGline PN:60 --outSAMattrRGline PE:61 --outSAMattrRGline PF:62 --outSAMattrRGline PG:63 --outSAMattrRGline PI:64 --outSAMattrRGline PB:65 --outSAMattrRGline PP:66 --outSAMattrRGline PT:67 --outSAMattrRGline PM:68 --outSAMattrRGline PN:69 --outSAMattrRGline PE:70 --outSAMattrRGline PF:71 --outSAMattrRGline PG:72 --outSAMattrRGline PI:73 --outSAMattrRGline PB:74 --outSAMattrRGline PP:75 --outSAMattrRGline PT:76 --outSAMattrRGline PM:77 --outSAMattrRGline PN:78 --outSAMattrRGline PE:79 --outSAMattrRGline PF:80 --outSAMattrRGline PG:81 --outSAMattrRGline PI:82 --outSAMattrRGline PB:83 --outSAMattrRGline PP:84 --outSAMattrRGline PT:85 --outSAMattrRGline PM:86 --outSAMattrRGline PN:87 --outSAMattrRGline PE:88 --outSAMattrRGline PF:89 --outSAMattrRGline PG:90 --outSAMattrRGline PI:91 --outSAMattrRGline PB:92 --outSAMattrRGline PP:93 --outSAMattrRGline PT:94 --outSAMattrRGline PM:95 --outSAMattrRGline PN:96 --outSAMattrRGline PE:97 --outSAMattrRGline PF:98 --outSAMattrRGline PG:99 --outSAMattrRGline PI:100 --outSAMattrRGline PB:101 --outSAMattrRGline PP:102 --outSAMattrRGline PT:103 --outSAMattrRGline PM:104 --outSAMattrRGline PN:105 --outSAMattrRGline PE:106 --outSAMattrRGline PF:107 --outSAMattrRGline PG:108 --outSAMattrRGline PI:109 --outSAMattrRGline PB:110 --outSAMattrRGline PP:111 --outSAMattrRGline PT:112 --outSAMattrRGline PM:113 --outSAMattrRGline PN:114 --outSAMattrRGline PE:115 --outSAMattrRGline PF:116 --outSAMattrRGline PG:117 --outSAMattrRGline PI:118 --outSAMattrRGline PB:119 --outSAMattrRGline PP:120 --outSAMattrRGline PT:121 --outSAMattrRGline PM:122 --outSAMattrRGline PN:123 --outSAMattrRGline PE:124 --outSAMattrRGline PF:125 --outSAMattrRGline PG:126 --outSAMattrRGline PI:127 --outSAMattrRGline PB:128 --outSAMattrRGline PP:129 --outSAMattrRGline PT:130 --outSAMattrRGline PM:131 --outSAMattrRGline PN:132 --outSAMattrRGline

	PE:133 --outSAMattrRGline PF:134 --outSAMattrRGline PG:135 --outSAMattrRGline PI:136 --outSAMattrRGline PB:137 --outSAMattrRGline PP:138 --outSAMattrRGline PT:139 --outSAMattrRGline PM:140 --outSAMattrRGline PN:141 --outSAMattrRGline PE:142 --outSAMattrRGline PF:143 --outSAMattrRGline PG:144 --outSAMattrRGline PI:145 --outSAMattrRGline PB:146 --outSAMattrRGline PP:147 --outSAMattrRGline PT:148 --outSAMattrRGline PM:149 --outSAMattrRGline PN:150 --outSAMattrRGline PE:151 --outSAMattrRGline PF:152 --outSAMattrRGline PG:153 --outSAMattrRGline PI:154 --outSAMattrRGline PB:155 --outSAMattrRGline PP:156 --outSAMattrRGline PT:157 --outSAMattrRGline PM:158 --outSAMattrRGline PN:159 --outSAMattrRGline PE:160 --outSAMattrRGline PF:161 --outSAMattrRGline PG:162 --outSAMattrRGline PI:163 --outSAMattrRGline PB:164 --outSAMattrRGline PP:165 --outSAMattrRGline PT:166 --outSAMattrRGline PM:167 --outSAMattrRGline PN:168 --outSAMattrRGline PE:169 --outSAMattrRGline PF:170 --outSAMattrRGline PG:171 --outSAMattrRGline PI:172 --outSAMattrRGline PB:173 --outSAMattrRGline PP:174 --outSAMattrRGline PT:175 --outSAMattrRGline PM:176 --outSAMattrRGline PN:177 --outSAMattrRGline PE:178 --outSAMattrRGline PF:179 --outSAMattrRGline PG:180 --outSAMattrRGline PI:181 --outSAMattrRGline PB:182 --outSAMattrRGline PP:183 --outSAMattrRGline PT:184 --outSAMattrRGline PM:185 --outSAMattrRGline PN:186 --outSAMattrRGline PE:187 --outSAMattrRGline PF:188 --outSAMattrRGline PG:189 --outSAMattrRGline PI:190 --outSAMattrRGline PB:191 --outSAMattrRGline PP:192 --outSAMattrRGline PT:193 --outSAMattrRGline PM:194 --outSAMattrRGline PN:195 --outSAMattrRGline PE:196 --outSAMattrRGline PF:197 --outSAMattrRGline PG:198 --outSAMattrRGline PI:199 --outSAMattrRGline PB:200 --outSAMattrRGline PP:201 --outSAMattrRGline PT:202 --outSAMattrRGline PM:203 --outSAMattrRGline PN:204 --outSAMattrRGline PE:205 --outSAMattrRGline PF:206 --out

ONT sequencing Analysis   Case 14.1: isoform expression

<table>
<tr><td>User's Inputs</td><td>You are a bioinformatician who specializes in writing code and performing analysis for a variety of bioinformatic data analysis tasks. Now I provide you with an Ubuntu 18.04 system where I save the following data, each line including the data path and data description.

RHL5569-T1_SAECK_shControl_replicate_1.ont.fastq.gz: ONT sequencing data from human cell line, control samples replicate 1
RHL5570-T1_SAECK_shControl_replicate_2.ont.fastq.gz: ONT sequencing data from human cell line, control samples replicate 2
RHL5571-T1_SAECK_shPARP4-1_replicate_1.ont.fastq.gz: ONT sequencing data from human cell line, treatment samples replicate 1
RHL5572-T1_SAECK_shPARP4-1_replicate_2.ont.fastq.gz: ONT sequencing data from human cell line, treatment samples replicate 2
hg38.fa: the human reference genome
gencode.v37.annotation.gtf: gene annotation

I need you to help me plan what needs to be done in each step of the analysis process, and help me write the code for each step to install softwares, use tools and accomplish the following main goal:

Quantify isoform expression in control and treatment samples</td></tr>
<tr><td>Generated Results by CodeLlama-34B-Instruct</td><td>Assistant: Sure, I can help you with that! Here's a plan for the analysis process and the code for each step:

Step 1: Install necessary software

* Install the following software:
+ FastQC: `sudo apt-get install fastqc`
+ Trimmomatic: `sudo apt-get install trimmomatic`
+ STAR: `sudo apt-get install star`
+ HISAT2: `sudo apt-get install hisat2`
+ StringTie: `sudo apt-get install stringtie`
+ Ballgown: `sudo apt-get install ballgown`

Step 2: Quality control and trimming

* Use FastQC to assess the quality of the raw sequencing data:
```bash
fastqc RHL5569-T1_SAECK_shControl_replicate_1.ont.fastq.gz
fastqc RHL5570-T1_SAECK_shControl_replicate_2.ont.fastq.gz
fastqc RHL5571-T1_SAECK_shPARP4-1_replicate_1.ont.fastq.gz
fastqc RHL5572-T1_SAECK_shPARP4-1_replicate_2.ont.fastq.gz
```
* Use Trimmomatic to trim the adapters and low-quality reads:
```bash
trimmomatic RHL5569-T1_SAECK_shControl_replicate_1.ont.fastq.gz
RHL5569-T1_SAECK_shControl_replicate_1.ont.trimmed.fastq.gz
ILLUMINACLIP:TruSeq3-PE.fa:2:30:10 LEADING:3 TRAILING:3
SLIDINGWINDOW:4:15 MINLEN:36
trimmomatic RHL5570-T1_SAECK_shControl_replicate_2.ont.fastq.gz
RHL5570-T1_SAECK_shControl_replicate_2.ont.trimmed.fastq.gz</td></tr>
</table>

ILLUMINACLIP:TruSeq3-PE.fa:2:30:10 LEADING:3 TRAILING:3
SLIDINGWINDOW:4:15 MINLEN:36
trimmomatic RHL5571-T1_SAECK_shPARP4-1_replicate_1.ont.fastq.gz
RHL5571-T1_SAECK_shPARP4-1_replicate_1.ont.trimmed.fastq.gz
ILLUMINACLIP:TruSeq3-PE.fa:2:30:10 LEADING:3 TRAILING:3
SLIDINGWINDOW:4:15 MINLEN:36
trimmomatic RHL5572-T1_SAECK_shPARP4-1_replicate_2.ont.fastq.gz
RHL5572-T1_SAECK_shPARP4-1_replicate_2.ont.trimmed.fastq.gz
ILLUMINACLIP:TruSeq3-PE.fa:2:30:10 LEADING:3 TRAILING:3
SLIDINGWINDOW:4:15 MINLEN:36
```

Step 3: Mapping and counting

* Use STAR to map the trimmed reads to the human reference genome:
```bash
STAR --runMode alignReads --runThreadN 4 --genomeDir /path/to/hg38 --readFilesIn RHL5569-T1_SAECK_shControl_replicate_1.ont.trimmed.fastq.gz RHL5570-T1_SAECK_shControl_replicate_2.ont.trimmed.fastq.gz RHL5571-T1_SAECK_shPARP4-1_replicate_1.ont.trimmed.fastq.gz RHL5572-T1_SAECK_shPARP4-1_replicate_2.ont.trimmed.fastq.gz --outFileNamePrefix RHL5569-T1_SAECK_shControl_replicate_1.ont.trimmed.star
```

* Use HISAT2 to map the trimmed reads to the human reference genome:
```bash
hisat2 -p 4 -x /path/to/hg38 -1 RHL5569-T1_SAECK_shControl_replicate_1.ont.trimmed.fastq.gz -2 RHL5570-T1_SAECK_shControl_replicate_2.ont.trimmed.fastq.gz -S RHL5569-T1_SAECK_shControl_replicate_1.ont.trimmed.hisat2.sam
hisat2 -p 4 -x /path/to/hg38 -1 RHL5571-T1_SAECK_shPARP4-1_replicate_1.ont.trimmed.fastq.gz -2 RHL5572-T1_SAECK_shPARP4-1_replicate_2.ont.trimmed.fastq.gz -S RHL5571-T1_SAECK_shPARP4-1_replicate_1.ont.trimmed.hisat2.sam
```

* Use StringTie to quantify the expression of genes and isoforms:
```bash
stringtie -p 4 -G /path/to/gencode.v37.annotation.gtf -o RHL5569-T1_SAECK_shControl_replicate_1.ont.trimmed.stringtie.gtf RHL5569-T1_SAECK_shControl_replicate_1.ont.trimmed.star.bam
stringtie -p 4 -G /path/to/gencode.v37.annotation.gtf -o RHL5570-T1_SAECK_shControl_replicate_2.ont.trimmed.stringtie.gtf RHL5570-T1_SAECK_shControl_replicate_2.ont.trimmed.star.bam
stringtie -p 4 -G /path/to/gencode.v37.annotation.gtf -o RHL5571-T1_SAECK_shPARP4-1_replicate_1.ont.trimmed.stringtie.gtf RHL5571-T1_SAECK_shPARP4-1_replicate_1.ont.trimmed.star.bam
stringtie -p 4 -G /path/to/gencode.v37.annotation.gtf -o RHL5572-T1_SAECK_shPARP4-1_replicate_2.ont.trimmed.stringtie.gtf RHL5572-T1_SAECK_shPARP4-1_replicate_2.ont.trimmed.star.bam
```

* Use Ballgown to quantify the expression of genes and isoforms:
```bash

```
ballgown count -p 4 -o RHL5569-
T1_SAECK_shControl_replicate_1.ont.trimmed.ballgown.counts RHL5569-
T1_SAECK_shControl_replicate_1.ont.trimmed.stringtie.gtf RHL5569-
T1_SAECK_shControl_replicate_1.ont.trimmed.star.bam
ballgown count -p 4 -o RHL5570-
T1_SAECK_shControl_replicate_2.ont.trimmed.ballgown.counts RHL5570-
T1_SAECK_shControl_replicate_2.ont.trimmed.stringtie.gtf RHL5570-
T1_SAECK_shControl_replicate_2.ont.trimmed.star.bam
ballgown count -p 4 -o RHL5571-T1_SAECK_shPARP4-
1_replicate_1.ont.trimmed.ballgown.counts RHL5571-
T1_SAECK_shPARP4-1_replicate_1.ont.trimmed.stringtie.gtf RHL5571-
T1_SAECK_shPARP4-1_replicate_1.ont.trimmed.star.bam
ballgown count -p 4 -o RHL5572-T1_SAECK_shPARP4-
1_replicate_2.ont.trimmed.ballgown.counts RHL5572-
T1_SAECK_shPARP4-1_replicate_2.ont.trimmed.stringtie.gtf RHL5572-
T1_SAECK_shPARP4-1_replicate_2.ont.trimmed.star.bam
```

Step 4: Alternative polyadenylation analysis

* Use the Ballgown package to identify differentially expressed genes and isoforms:

```bash
ballgown diff -p 4 -o RHL5569-
T1_SAECK_shControl_replicate_1.ont.trimmed.ballgown.diff RHL5569-
T1_SAECK_shControl_replicate_1.ont.trimmed.ballgown.counts RHL5570-
T1_SAECK_shControl_replicate_2.ont.trimmed.ballgown.counts
ballgown diff -p 4 -o RHL5571-T1_SAECK_shPARP4-
1_replicate_1.ont.trimmed.ballgown.diff RHL5571-T1_SAECK_shPARP4-
1_replicate_1.ont.trimmed.ballgown.counts RHL5572-
T1_SAECK_shPARP4-1_replicate_2.ont.trimmed.ballgown.counts
```

* Use the Ballgown package to identify differentially expressed isoforms:

```bash
ballgown isoform -p 4 -o RHL5569-
T1_SAECK_shControl_replicate_1.ont.trimmed.ballgown.isoform RHL5569-
T1_SAECK_shControl_replicate_1.ont.trimmed.ballgown.counts RHL5570-
T1_SAECK_shControl_replicate_2.ont.trimmed.ballgown.counts
ballgown isoform -p 4 -o RHL5571-T1_SAECK_shPARP4-
1_replicate_1.ont.trimmed.ballgown.isoform RHL5571-
T1_SAECK_shPARP4-1_replicate_1.ont.trimmed.ballgown.counts
RHL5572-T1_SAECK_shPARP4-
1_replicate_2.ont.trimmed.ballgown.counts
```

* Use the Ballgown package to identify differentially alternative polyadenylation sites:

```bash
ballgown polyA -p 4 -o RHL5569-
T1_SAECK_shControl_replicate_1.ont.trimmed.ballgown.polyA RHL5569-
T1_SAECK_shControl_replicate_1.ont.trimmed.ballgown.counts RHL5570-
T1_SAECK_shControl_replicate_2.ont.trimmed.ballgown.counts
ballgown polyA -p 4 -o RHL5571-T1_SAECK_shPARP4-
1_replicate_1.ont.trimmed.ballgown.polyA RHL5571-
T1_SAECK_shPARP4-1_replicate_1.ont.trimmed.ballgown.counts
```

RHL5572-T1_SAECK_shPARP4-1_replicate_2.ont.trimmed.ballgown.counts
```

Step 5: Visualization and interpretation

* Use the Ballgown package to visualize the results:

```bash
ballgown plot -p 4 -o RHL5569-T1_SAECK_shControl_replicate_1.ont.trimmed.ballgown.plot RHL5569-T1_SAECK_shControl_replicate_1.ont.trimmed.ballgown.counts RHL5570-T1_SAECK_shControl_replicate_2.ont.trimmed.ballgown.counts
ballgown plot -p 4 -o RHL5571-T1_SAECK_shPARP4-1_replicate_1.ont.trimmed.ballgown.plot RHL5571-T1_SAECK_shPARP4-1_replicate_1.ont.trimmed.ballgown.counts RHL5572-T1_SAECK_shPARP4-1_replicate_2.ont.trimmed.ballgown.counts
```

* Use the Ballgown package to interpret the results:

```bash
ballgown interpret -p 4 -o RHL5569-T1_SAECK_shControl_replicate_1.ont.trimmed.ballgown.interpret RHL5569-T1_SAECK_shControl_replicate_1.ont.trimmed.ballgown.counts RHL5570-T1_SAECK_shControl_replicate_2.ont.trimmed.ballgown.counts
ballgown interpret -p 4 -o RHL5571-T1_SAECK_shPARP4-1_replicate_1.ont.trimmed.ballgown.interpret RHL5571-T1_SAECK_shPARP4-1_replicate_1.ont.trimmed.ballgown.counts RHL5572-T1_SAECK_shPARP4-1_replicate_2.ont.trimmed.ballgown.counts
```

Note: The above code is just a general outline of the analysis process and may need to be modified based on the specific requirements of your project. Additionally, the Ballgown package may require additional parameters to be set, such as the `--min-reads` parameter to specify the minimum number of reads required to detect an isoform.
```

PacBio iso-seq Analysis **Case 15.1: isoform analysis**

**User's Inputs**	You are a bioinformatician who specializes in writing code and performing analysis for a variety of bioinformatic data analysis tasks. Now I provide you with an Ubuntu 18.04 system where I save the following data, each line including the data path and data description.<br><br>SRR19552218.fastq.gz: pacbio sequecning data from human cell line, cell line HCT-116<br>SRR19785215.fastq.gz: pacbio sequecning data from human cell line, cell line HepG2<br>hg38.fa: the human reference genome<br>gencode.v37.annotation.gtf: gene annotation<br><br>I need you to help me plan what needs to be done in each step of the analysis process, and help me write the code for each step to install softwares, use tools and accomplish the following main goal:<br><br>identify novel isoform and quantify isoform expression in two cell lines
**Generated Results by CodeLlama-34B-Instruct**	Assistant: Sure, I can help you with that! Here's a high-level plan for the analysis process:<br><br>1. Install necessary software:<br>* PacBio BAM format conversion tool (pbzip)<br>* HISAT2 (spliced aligner)<br>* StringTie (transcript quantification)<br>* Cufflinks (transcript assembly and quantification)<br>* Ballgown (transcript quantification)<br>* DESeq2 (differential expression analysis)<br>2. Convert PacBio sequencing data to BAM format using pbzip:<br>* `pbzip -d SRR19552218.fastq.gz`<br>* `pbzip -d SRR19785215.fastq.gz`<br>3. Align the PacBio sequencing data to the human reference genome using HISAT2:<br>* `hisat2 -p 8 --dta -x hg38 -1 SRR19552218.fastq.gz -2 SRR19785215.fastq.gz -S SRR19552218.sam`<br>4. Convert the SAM alignment file to BAM format:<br>* `samtools view -bS SRR19552218.sam > SRR19552218.bam`<br>* `samtools view -bS SRR19785215.sam > SRR19785215.bam`<br>5. Sort and index the BAM files:<br>* `samtools sort SRR19552218.bam -o SRR19552218.sorted.bam`<br>* `samtools sort SRR19785215.bam -o SRR19785215.sorted.bam`<br>* `samtools index SRR19552218.sorted.bam`<br>* `samtools index SRR19785215.sorted.bam`<br>6. Quantify transcript expression using StringTie:<br>* `stringtie -p 8 -G hg38.fa -o SRR19552218.gtf -l SRR19552218.sorted.bam`<br>* `stringtie -p 8 -G hg38.fa -o SRR19785215.gtf -l SRR19785215.sorted.bam`<br>7. Quantify transcript expression using Cufflinks:<br>* `cufflinks -p 8 -o SRR19552218.gtf -l SRR19552218.sorted.bam`<br>* `cufflinks -p 8 -o SRR19785215.gtf -l SRR19785215.sorted.bam`<br>8. Quantify transcript expression using Ballgown:

	* `ballgown -p 8 -o SRR19552218.gtf -l SRR19552218.sorted.bam`<br>* `ballgown -p 8 -o SRR19785215.gtf -l SRR19785215.sorted.bam`<br>9. Perform differential expression analysis using DESeq2:<br>* `deseq2 -p 8 -o SRR19552218.gtf -l SRR19552218.sorted.bam -c SRR19785215.gtf -l SRR19785215.sorted.bam`<br><br>Note that the above commands are just a rough outline of the analysis process, and may need to be modified based on the specific requirements of your project. Additionally, the commands may need to be adjusted to accommodate any specific requirements or constraints of your Ubuntu 18.04 system.

CLIP-seq Analysis **Case 16.1: CLIP-seq data analysis**

<table>
<tr><td>User's Inputs</td><td>You are a bioinformatician who specializes in writing code and performing analysis for a variety of bioinformatic data analysis tasks. Now I provide you with an Ubuntu 18.04 system where I save the following data, each line including the data path and data description.

ENCLB742AYH.fastq.gz: single end reads of eCLIP data from HepG2 cell line, SRSF1 targeted sample replicate 1
ENCLB770EDJ.fastq.gz: single end reads of eCLIP data from HepG2 cell line, SRSF1 targeted sample replicate 2
hg38.fa: the human reference genome
gencode.v37.annotation.gtf: gene annotation

I need you to help me plan what needs to be done in each step of the analysis process, and help me write the code for each step to install softwares, use tools and accomplish the following main goal:

identify SRSF1 protein-RNA crosslink sites based on CLIP sequencing data</td></tr>
<tr><td>Generated Results by CodeLlama-34B-Instruct</td><td>## Step 1
Assistant: Sure, I'd be happy to help you plan and write the code for your eCLIP data analysis pipeline. Here's a high-level overview of the steps involved in the analysis process:

1. Data preprocessing:
* Trim the adapters from the raw sequencing reads using tools like Trimmomatic or Cutadapt.
* Remove low-quality reads using tools like FastQC or FASTX.
* Filter out reads that are not of the desired length or have low-quality base calls.
2. Mapping:
* Map the preprocessed reads to the reference genome using a spliced aligner like STAR or HISAT2.
* Sort and index the mapped reads using tools like Samtools or BWA.
3. Peak calling:
* Identify regions of the genome that are enriched with crosslinked RNA using tools like MACS2 or HISAT2.
* Filter out peaks that are not of the desired size or have low-quality scores.
4. Annotation:
* Annotate the peaks with information about the genes they overlap using tools like Bedtools or GFFTools.
* Filter out peaks that do not overlap with any known genes.
5. Visualization:
* Visualize the peaks and their annotations using tools like UCSC Genome Browser or Integrative Genomics Viewer (IGV).

Here's an example code snippet for each step:

1. Data preprocessing:
```bash
Trim adapters using Trimmomatic</td></tr>
</table>

trimmomatic PE -threads 4 ENCLB742AYH.fastq.gz
ENCLB770EDJ.fastq.gz ENCLB742AYH_trimmed.fastq.gz
ENCLB770EDJ_trimmed.fastq.gz ILLUMINACLIP:TruSeq3-PE.fa:2:30:10
LEADING:3 TRAILING:3 SLIDINGWINDOW:4:15 MINLEN:36

Remove low-quality reads using FastQC
fastqc -t 4 -o fastqc_results ENCLB742AYH_trimmed.fastq.gz
ENCLB770EDJ_trimmed.fastq.gz

Filter out reads that are not of the desired length or have low-quality base calls
awk 'BEGIN {FS="\t"; OFS="\t"} {if ($3 >= 36 && $4 >= 30) print $1, $2, $3, $4, $5, $6}' fastqc_results/ENCLB742AYH_trimmed_fastqc.txt >
ENCLB742AYH_filtered.fastq
awk 'BEGIN {FS="\t"; OFS="\t"} {if ($3 >= 36 && $4 >= 30) print $1, $2, $3, $4, $5, $6}' fastqc_results/ENCLB770EDJ_trimmed_fastqc.txt >
ENCLB770EDJ_filtered.fastq
```
2. Mapping:
```bash
# Map reads to the reference genome using STAR
STAR --runThreadN 4 --genomeDir hg38 --readFilesIn
ENCLB742AYH_filtered.fastq ENCLB770EDJ_filtered.fastq --outSAMtype
BAM SortedByCoordinate --outSAMunmapped Within --outSAMattributes
NH HI NM MD AS --outSAMstrandField intronMotif

# Sort and index the mapped reads using Samtools
samtools sort -@ 4 -o ENCLB742AYH_sorted.bam
ENCLB742AYH_filtered.fastq.gz
samtools sort -@ 4 -o ENCLB770EDJ_sorted.bam
ENCLB770EDJ_filtered.fastq.gz
samtools index -@ 4 ENCLB742AYH_sorted.bam
samtools index -@ 4 ENCLB770EDJ_sorted.bam
```
3. Peak calling:
```bash
# Identify regions of the genome that are enriched with crosslinked RNA using MACS2
macs2 callpeak -t ENCLB742AYH_sorted.bam ENCLB770EDJ_sorted.bam
-f BAM -g hg38.fa -n ENCLB742AYH_ENCLB770EDJ

# Filter out peaks that are not of the desired size or have low-quality scores
awk 'BEGIN {FS="\t"; OFS="\t"} {if ($3 >= 36 && $4 >= 30) print $1, $2, $3, $4, $5, $6}'
macs2_results/ENCLB742AYH_ENCLB770EDJ_peaks.narrowPeak >
ENCLB742AYH_ENCLB770EDJ_filtered_peaks.narrowPeak
```
4. Annotation:
```bash
# Annotate the peaks with information about the genes they overlap using Bedtools
bedtools intersect -a
ENCLB742AYH_ENCLB770EDJ_filtered_peaks.narrowPeak -b

gencode.v37.annotation.gtf -wa -wb >
ENCLB742AYH_ENCLB770EDJ_filtered_peaks_annotated.narrowPeak

# Filter out peaks that do not overlap with any known genes
awk 'BEGIN {FS="\t"; OFS="\t"} {if ($10 != ".") print $1, $2, $3, $4, $5, $6, $7, $8, $9, $10}'
ENCLB742AYH_ENCLB770EDJ_filtered_peaks_annotated.narrowPeak >
ENCLB742AYH_ENCLB770EDJ_filtered_peaks_annotated_genes.narrowPeak
```
5. Visualization:
```bash
# Visualize the peaks and their annotations using UCSC Genome Browser
wigToBigWig
ENCLB742AYH_ENCLB770EDJ_filtered_peaks_annotated_genes.narrowPeak hg38.fa
ENCLB742AYH_ENCLB770EDJ_filtered_peaks_annotated_genes.bw

# Visualize the peaks and their annotations using IGV
igv -b
ENCLB742AYH_ENCLB770EDJ_filtered_peaks_annotated_genes.bw
hg38.fa
```
Note that this is just an example code snippet and may need to be modified based on the specific requirements of your analysis. Additionally, this pipeline assumes that you have the necessary software and tools installed on your system.
```

RIP-Seq Analysis   **Case 16.2: Identify enriched genes bounded by RPB**

**User's Inputs**	You are a bioinformatician who specializes in writing code and performing analysis for a variety of bioinformatic data analysis tasks. Now I provide you with an Ubuntu 18.04 system where I save the following data, each line including the data path and data description.<br><br>rip.rep1.fastq.gz: single end reads of RIP-seq data from HepG2 cell line, pulldown samples of SRSF1 replicate 1<br>rip.rep2.fastq.gz: single end reads of RIP-seq data from HepG2 cell line, pulldown samples of SRSF1 replicate 2<br>controll.rep1.fastq.gz: single end reads of RIP-seq daata from HepG2 cell line, control sample replicate 1<br>controll.rep2.fastq.gz: single end reads of RIP-seq daata from HepG2 cell line, control sample replicate 2<br>hg38.fa: the human reference genome<br>gencode.v37.annotation.gtf: gene annotation<br><br>I need you to help me plan what needs to be done in each step of the analysis process, and help me write the code for each step to install softwares, use tools and accomplish the following main goal:<br><br>identify genes enriched in pulldown samples over the control
**Generated Results by CodeLlama-34B-Instruct**	Assistant: Sure, I can help you with that! Here's a step-by-step plan for the analysis process:<br><br>1. Install necessary software:<br>* FastQC: A quality control tool for high-throughput sequencing data.<br>* Trimmomatic: A tool for trimming adapters and low-quality bases from FastQ files.<br>* HISAT2: A spliced aligner for RNA-seq data.<br>* StringTie: A tool for quantifying gene expression and splicing from RNA-seq data.<br>* Ballgown: A tool for differential gene expression analysis.<br>* DESeq2: A tool for statistical analysis of gene expression data.<br>2. Preprocess the data:<br>* Use FastQC to assess the quality of the raw reads.<br>* Use Trimmomatic to trim adapters and low-quality bases from the reads.<br>* Use HISAT2 to align the trimmed reads to the human reference genome (hg38.fa).<br>* Use StringTie to quantify gene expression and splicing from the aligned reads.<br>3. Identify genes enriched in pulldown samples over the control:<br>* Use Ballgown to perform differential gene expression analysis between the pulldown and control samples.<br>* Use DESeq2 to perform statistical analysis of the differentially expressed genes.<br>4. Annotate the results:<br>* Use the gene annotation file (gencode.v37.annotation.gtf) to add functional information to the differentially expressed genes.<br>5. Visualize the results:

* Use a tool such as ggplot2 or Plotly to visualize the results, such as a bar plot of the top differentially expressed genes.

Here's some sample code for each step:

1. Installing necessary software:
```bash
sudo apt-get update
sudo apt-get install fastqc trimmomatic hisat2 stringtie ballgown deseq2
```
2. Preprocessing the data:
```bash
# FastQC
fastqc rip.rep1.fastq.gz rip.rep2.fastq.gz controll.rep1.fastq.gz
controll.rep2.fastq.gz

# Trimmomatic
trimmomatic PE -phred33 rip.rep1.fastq.gz rip.rep2.fastq.gz
controll.rep1.fastq.gz controll.rep2.fastq.gz

# HISAT2
hisat2-build -p 4 hg38.fa hg38
hisat2 -p 4 -x hg38 -1 rip.rep1.fastq.gz -2 rip.rep2.fastq.gz -U
controll.rep1.fastq.gz -U controll.rep2.fastq.gz

# StringTie
stringtie -p 4 -G hg38.fa -o rip.rep1.gtf rip.rep1.bam
stringtie -p 4 -G hg38.fa -o rip.rep2.gtf rip.rep2.bam
stringtie -p 4 -G hg38.fa -o controll.rep1.gtf controll.rep1.bam
stringtie -p 4 -G hg38.fa -o controll.rep2.gtf controll.rep2.bam
```
3. Identifying genes enriched in pulldown samples over the control:
```bash
# Ballgown
ballgown -p 4 -o rip.rep1.ballgown rip.rep1.gtf
ballgown -p 4 -o rip.rep2.ballgown rip.rep2.gtf
ballgown -p 4 -o controll.rep1.ballgown controll.rep1.gtf
ballgown -p 4 -o controll.rep2.ballgown controll.rep2.gtf

# DESeq2
deseq2 -p 4 -o rip.rep1.deseq2 rip.rep1.ballgown
deseq2 -p 4 -o rip.rep2.deseq2 rip.rep2.ballgown
deseq2 -p 4 -o controll.rep1.deseq2 controll.rep1.ballgown
deseq2 -p 4 -o controll.rep2.deseq2 controll.rep2.ballgown
```
4. Annotating the results:
```bash
# Add functional information to the differentially expressed genes
annotate_genes -p 4 -o rip.rep1.deseq2.annotated rip.rep1.deseq2
gencode.v37.annotation.gtf
annotate_genes -p 4 -o rip.rep2.deseq2.annotated rip.rep2.deseq2
gencode.v37.annotation.gtf
```

	annotate_genes -p 4 -o controll.rep1.deseq2.annotated<br>controll.rep1.deseq2 gencode.v37.annotation.gtf<br>annotate_genes -p 4 -o controll.rep2.deseq2.annotated<br>controll.rep2.deseq2 gencode.v37.annotation.gtf<br>```<br>5. Visualizing the results:<br>```bash<br># Visualize the results using ggplot2<br>ggplot(rip.rep1.deseq2.annotated, aes(x = log2FoldChange, y = -<br>log10(padj))) +<br>geom_point() +<br>labs(x = "log2 fold change", y = "-log10 adjusted p-value")<br><br># Visualize the results using Plotly<br>plot_ly(rip.rep1.deseq2.annotated, x = log2FoldChange, y = -log10(padj),<br>type = "scatter", mode = "markers")<br>```<br>Note that this is just a sample code and you may need to modify it based on your specific needs and the version of the software you are using. Additionally, you may need to install additional packages or libraries to run some of the commands.

Ribo-Seq Analysis **Case 17.1: Identify translated ORFs**

<table>
<tr>
<td>User’s Inputs</td>
<td>You are a bioinformatician who specializes in writing code and performing analysis for a variety of bioinformatic data analysis tasks. Now I provide you with an Ubuntu 18.04 system where I save the following data, each line including the data path and data description.

SRR12354645_1.fastq.gz: read 1 of paired-end ribo-seq data, read 1 of control samples replicate 1
SRR12354645_2.fastq.gz: read 2 of paired-end ribo-seq data, read 2 of control samples replicate 1
SRR12354646_1.fastq.gz: read 1 of paired-end ribo-seq data, read 1 of control samples replicate 2
SRR12354646_2.fastq.gz: read 2 of paired-end ribo-seq data, read 2 of control samples replicate 2
hg38.fa: the human reference genome
gencode.v37.annotation.gtf: gene annotation
TruSeq3-PE.fa: trimming adapter

I need you to help me plan what needs to be done in each step of the analysis process, and help me write the code for each step to install softwares, use tools and accomplish the following main goal:

Identify translated ORFs based on ribo-seq data</td>
</tr>
<tr>
<td>Generated Results by CodeLlama-34B-Instruct</td>
<td>Assistant: Sure, I can help you with that! Here's a step-by-step plan for the analysis process:

1. Install necessary software:
* FastQC: A quality control tool for high-throughput sequencing data.
* Trimmomatic: A tool for trimming adapters and low-quality bases from FastQ files.
* STAR: A spliced aligner for RNA-seq data.
* HISAT2: A spliced aligner for RNA-seq data.
* Cufflinks: A tool for quantifying gene expression from RNA-seq data.
* Ballgown: A tool for visualizing and analyzing gene expression data.

You can install these tools using the following commands:
```bash
sudo apt-get update
sudo apt-get install fastqc trimmomatic star hisat2 cufflinks ballgown
```
2. Preprocess the data:
* Use FastQC to assess the quality of the raw FastQ files.
* Use Trimmomatic to trim the adapters and low-quality bases from the FastQ files.
* Use STAR or HISAT2 to align the trimmed reads to the reference genome.
* Use Cufflinks to quantify the gene expression from the aligned reads.</td>
</tr>
</table>

Here's an example code for preprocessing the data using FastQC, Trimmomatic, STAR, and Cufflinks:
```bash
# Run FastQC on the raw FastQ files
fastqc SRR12354645_1.fastq.gz SRR12354645_2.fastq.gz
SRR12354646_1.fastq.gz SRR12354646_2.fastq.gz

# Trim the adapters and low-quality bases from the FastQ files using
Trimmomatic
trimmomatic PE SRR12354645_1.fastq.gz SRR12354645_2.fastq.gz
SRR12354646_1.fastq.gz SRR12354646_2.fastq.gz

# Align the trimmed reads to the reference genome using STAR
star --runMode alignReads --readFilesIn SRR12354645_1.fastq.gz
SRR12354645_2.fastq.gz SRR12354646_1.fastq.gz
SRR12354646_2.fastq.gz --genomeDir hg38 --outFileNamePrefix
SRR12354645_aligned

# Quantify the gene expression from the aligned reads using Cufflinks
cufflinks -p 4 -o SRR12354645_aligned.gtf SRR12354645_aligned.bam
```
3. Identify translated ORFs:
  * Use Ballgown to identify the translated ORFs from the gene expression data.

Here's an example code for identifying translated ORFs using Ballgown:
```bash
# Run Ballgown on the gene expression data
ballgown -p 4 -o SRR12354645_aligned.gtf SRR12354645_aligned.bam

# Identify the translated ORFs from the gene expression data
ballgown -p 4 -o SRR12354645_aligned.gtf SRR12354645_aligned.bam --
orfs
```
4. Visualize the results:
  * Use Ballgown to visualize the translated ORFs and their expression levels.

Here's an example code for visualizing the translated ORFs using Ballgown:
```bash
# Run Ballgown on the gene expression data
ballgown -p 4 -o SRR12354645_aligned.gtf SRR12354645_aligned.bam

# Visualize the translated ORFs and their expression levels
ballgown -p 4 -o SRR12354645_aligned.gtf SRR12354645_aligned.bam --
orfs --plot
```
Note that this is just a basic plan for the analysis process, and you may need to modify the code and the parameters based on your specific requirements and the characteristics of your data.

scRNA-Seq Analysis Case 18.1: Cluster cell into groups based on raw fastq files

<table>
<tr><td>User's Inputs</td><td>You are a bioinformatician who specializes in writing code and performing analysis for a variety of bioinformatic data analysis tasks. Now I provide you with an Ubuntu 18.04 system where I save the following data, each line including the data path and data description.

/pbmc_1k_v3_fastqs.tar: the packed fastq files from 10x single cell sequencing
refdata-gex-GRCh38-2020-A: directory contains transcriptome reference

I need you to help me plan what needs to be done in each step of the analysis process, and help me write the code for each step to install softwares, use tools and accomplish the following main goal:

cluster cells into different groups based on 10x single cell sequencing data</td></tr>
<tr><td>Generated Results by CodeLlama-34B-Instruct</td><td>Assistant: Sure, I can help you with that! Here's a high-level plan for the analysis process:

1. Install necessary software:
* FastQC: a quality control tool for high-throughput sequencing data.
* Cell Ranger: a tool for processing and analyzing 10x single cell sequencing data.
* Seurat: a popular R package for clustering and visualizing single-cell RNA sequencing data.
2. Preprocess the data:
* Use FastQC to assess the quality of the raw sequencing data.
* Trim and filter the reads using Cell Ranger.
* Map the reads to the transcriptome reference using Cell Ranger.
3. Normalize and transform the data:
* Normalize the read counts using the TMM (Trimmed Mean of M-values) method.
* Transform the data using the log2(x+1) method.
4. Cluster the cells:
* Use Seurat to cluster the cells based on their gene expression profiles.
* Choose an appropriate clustering algorithm (e.g. k-means, hierarchical clustering) and set the number of clusters.
* Visualize the clustering results using Seurat's built-in visualization tools.
5. Interpret the results:
* Analyze the clustering results to identify any patterns or trends in the data.
* Use Seurat's built-in tools to identify marker genes for each cluster and to perform pathway analysis.

Here's some sample code to get you started:
```bash
# Install necessary software
sudo apt-get install fastqc cellranger seurat

Preprocess the data
fastqc /pbmc_1k_v3_fastqs.tar</td></tr>
</table>

	cellranger count --transcriptome refdata-gex-GRCh38-2020-A --fastqs /pbmc_1k_v3_fastqs.tar<br><br># Normalize and transform the data<br>seurat_normalize(counts(cellranger_counts), method="TMM")<br>seurat_transform(counts(cellranger_counts), method="log2(x+1)")<br><br># Cluster the cells<br>seurat_cluster(counts(cellranger_counts), k=5, method="kmeans")<br><br># Visualize the clustering results<br>seurat_visualize(counts(cellranger_counts), clusters=seurat_cluster(counts(cellranger_counts), k=5, method="kmeans"))<br>```<br>Note that this is just a starting point, and you may need to modify the code depending on your specific needs and the characteristics of your data. Additionally, you may want to consider using other tools and methods for preprocessing, normalization, and clustering, as well as for visualizing and interpreting the results.

scRNA-Seq Analysis   Case 18.2: Clustering and Find the differentially expressed genes

<table>
<tr><td>User's Inputs</td><td>You are a bioinformatician who specializes in writing code and performing analysis for a variety of bioinformatic data analysis tasks. Now I provide you with an Ubuntu 18.04 system where I save the following data, each line including the data path and data description.

./examples/case2.1/data/filtered_gene_bc_matrices/hg19: path to 10x mtx data

I need you to help me plan what needs to be done in each step of the analysis process, and help me write the code for each step to install softwares, use tools and accomplish the following main goal:

Use scanpy to find the differentially expressed genes</td></tr>
<tr><td>Generated Results by CodeLlama-34B-Instruct</td><td>Assistant: Sure, I can help you with that! Here's a step-by-step plan for the analysis process:

1. Install necessary software:
* Install scanpy by running the following command: `pip install scanpy`
* Install other necessary packages such as pandas, numpy, and matplotlib by running the following command: `pip install pandas numpy matplotlib`
2. Load the data:
* Load the 10x mtx data into scanpy using the following command: `scanpy.read_mtx('./examples/case2.1/data/filtered_gene_bc_matrices/hg19')`
3. Preprocess the data:
* Filter out low-quality cells and genes using the following command: `scanpy.filter_cells(min_genes=200)` and `scanpy.filter_genes(min_cells=3)`
* Normalize the data using the following command: `scanpy.normalize_total(adata)`
4. Find differentially expressed genes:
* Use the following command to find differentially expressed genes between two conditions: `scanpy.de_test(adata, groupby='condition', test='t-test')`
* Use the following command to find the top 10 differentially expressed genes: `scanpy.de_test(adata, groupby='condition', test='t-test', n_genes=10)`
5. Visualize the results:
* Use the following command to visualize the results using a heatmap: `scanpy.heatmap(adata, groupby='condition', cmap='RdBu_r')`

Here's the code for each step:
```python
Step 1: Install necessary software
!pip install scanpy
!pip install pandas numpy matplotlib

# Step 2: Load the data
import scanpy</td></tr>
</table>

	adata =<br>scanpy.read_mtx('./examples/case2.1/data/filtered_gene_bc_matrices/hg19')<br><br># Step 3: Preprocess the data<br>adata = scanpy.filter_cells(adata, min_genes=200)<br>adata = scanpy.filter_genes(adata, min_cells=3)<br>adata = scanpy.normalize_total(adata)<br><br># Step 4: Find differentially expressed genes<br>de_res = scanpy.de_test(adata, groupby='condition', test='t-test')<br><br># Step 5: Visualize the results<br>scanpy.heatmap(adata, groupby='condition', cmap='RdBu_r')<br>```<br>Note that the `groupby` parameter in the `de_test` function specifies the column in the metadata that contains the condition information. In this case, it is assumed that the metadata contains a column named "condition" that contains the condition information. If your metadata contains a different column name, you will need to modify the `groupby` parameter accordingly.<br><br>--------------

scRNA-Seq Analysis Case 18.3: Identify Marker Genes

<table>
<tr><td>User's Inputs</td><td>You are a bioinformatician who specializes in writing code and performing analysis for a variety of bioinformatic data analysis tasks. Now I provide you with an Ubuntu 18.04 system where I save the following data, each line including the data path and data description.

./examples/case2.1/data/filtered_gene_bc_matrices/hg19: path to 10x mtx data

I need you to help me plan what needs to be done in each step of the analysis process, and help me write the code for each step to install softwares, use tools and accomplish the following main goal:

Use scanpy to identify top5 marker genes.</td></tr>
<tr><td>Generated Results by CodeLlama-34B-Instruct</td><td>Assistant: Sure, I can help you with that! To start, it would be helpful to know what kind of data you are working with and what your goals are for the analysis. Can you provide some more information about the data and what you hope to achieve with the analysis?--------------</td></tr>
</table>

scRNA-Seq Analysis Case 18.4: Clustering and Visualization

<table>
<tr><td>User's Inputs</td><td>You are a bioinformatician who specializes in writing code and performing analysis for a variety of bioinformatic data analysis tasks. Now I provide you with an Ubuntu 18.04 system where I save the following data, each line including the data path and data description.

./examples/case2.1/data/filtered_gene_bc_matrices/hg19: path to 10x mtx data

I need you to help me plan what needs to be done in each step of the analysis process, and help me write the code for each step to install softwares, use tools and accomplish the following main goal:

Use scanpy to perform clustering and visualize the expression level of gene PPBP in the UMAP.</td></tr>
<tr><td>Generated Results by CodeLlama-34B-Instruct</td><td>Assistant: To perform clustering and visualize the expression level of gene PPBP in the UMAP using scanpy, we will need to follow these steps:

1. Install the necessary software:
* Install scanpy using pip: `pip install scanpy`
* Install the necessary dependencies for scanpy: `pip install -r requirements.txt`
2. Load the data:
* Load the 10x mtx data into scanpy using the `read_10x_mtx()` function: `data = read_10x_mtx('./examples/case2.1/data/filtered_gene_bc_matrices/hg19')`
3. Preprocess the data:
* Filter out low-quality cells and genes using the `filter_cells()` and `filter_genes()` functions: `data = filter_cells(data, min_genes=200)` and `data = filter_genes(data, min_cells=3)`
* Normalize the data using the `normalize_total()` function: `data = normalize_total(data)`
4. Perform clustering:
* Use the `cluster()` function to perform clustering on the preprocessed data: `clusters = cluster(data, resolution=0.5)`
5. Visualize the expression level of gene PPBP in the UMAP:
* Use the `umap()` function to create a UMAP plot of the data: `umap = umap(data, clusters)`
* Use the `plot_umap()` function to plot the UMAP plot: `plot_umap(umap, cmap='viridis')`
6. Save the results:
* Use the `write_csv()` function to save the cluster assignments and the UMAP plot to a CSV file: `write_csv(clusters, 'clusters.csv')` and `write_csv(umap, 'umap.csv')`

Here is the code for each step:
```
Step 1: Install the necessary software
pip install scanpy
pip install -r requirements.txt

Step 2: Load the data</td></tr>
</table>

```
data =
read_10x_mtx('./examples/case2.1/data/filtered_gene_bc_matrices/hg19')

# Step 3: Preprocess the data
data = filter_cells(data, min_genes=200)
data = filter_genes(data, min_cells=3)
data = normalize_total(data)

# Step 4: Perform clustering
clusters = cluster(data, resolution=0.5)

# Step 5: Visualize the expression level of gene PPBP in the UMAP
umap = umap(data, clusters)
plot_umap(umap, cmap='viridis')

# Step 6: Save the results
write_csv(clusters, 'clusters.csv')
write_csv(umap, 'umap.csv')
```

Note that the `resolution` parameter in the `cluster()` function controls the granularity of the clustering. A higher resolution will result in more fine-grained clusters, while a lower resolution will result in fewer, larger clusters. The `cmap` parameter in the `plot_umap()` function controls the color scheme used to represent the clusters in the UMAP plot.

Spatial Transcriptomics **Case 19.1: Neighborhood enrichment analysis**

User's Inputs

You are a bioinformatician who specializes in writing code and performing analysis for a variety of bioinformatic data analysis tasks. Now I provide you with an Ubuntu 18.04 system where I save the following data, each line including the data path and data description.

slice1.h5ad: spatial transcriptomics data for slice 1 in AnnData format

I need you to help me plan what needs to be done in each step of the analysis process, and help me write the code for each step to install softwares, use tools and accomplish the following main goal:

Use squidpy for neighborhood enrichment analysis

Generated Results by CodeLlama-34B-Instruct

Assistant: Sure, I can help you with that! Here's a step-by-step plan for the analysis process:

1. Install necessary software:
   * Install Anaconda, a distribution of Python and other scientific computing packages, by following the instructions on the Anaconda website.
   * Install the necessary Python packages for the analysis, including squidpy, by running the following command in the terminal:

```
pip install squidpy
```
2. Load the data:
   * Load the spatial transcriptomics data from the slice1.h5ad file into an AnnData object using the `anndata` package.
   * Convert the data to a format that can be used by squidpy by running the following command:

```python
import anndata
import squidpy

adata = anndata.read_h5ad("slice1.h5ad")
adata = squidpy.convert_to_squidpy(adata)
```
3. Preprocess the data:
   * Preprocess the data to remove any unwanted features or cells, such as cells that are not in the tissue of interest or features that are not expressed in any cells.
   * Use the `squidpy.preprocess` function to perform the preprocessing.

```python
adata = squidpy.preprocess(adata)
```
4. Perform neighborhood enrichment analysis:

	* Use the `squidpy.neighborhood_enrichment` function to perform the neighborhood enrichment analysis.<br>* The function takes the preprocessed data and a set of genes of interest as input, and returns a list of genes that are enriched in the neighborhood of the genes of interest.<br><br>```python<br>genes_of_interest = ["gene1", "gene2", ...]<br>enriched_genes = squidpy.neighborhood_enrichment(adata, genes_of_interest)<br>```<br>5. Visualize the results:<br>* Use the `squidpy.plot` function to visualize the results of the neighborhood enrichment analysis.<br>* The function takes the preprocessed data and the list of enriched genes as input, and returns a plot that shows the distribution of the enriched genes in the neighborhood of the genes of interest.<br><br>```python<br>squidpy.plot(adata, enriched_genes)<br>```<br><br>I hope this helps! Let me know if you have any questions or need further assistance.<br>--------------

Spatial Transcriptomics **Case 19.2: Single-cell mapping**

<table>
<tr><td>User's Inputs</td><td>You are a bioinformatician who specializes in writing code and performing analysis for a variety of bioinformatic data analysis tasks. Now I provide you with an Ubuntu 18.04 system where I save the following data, each line including the data path and data description.

slice1.h5ad: spatial transcriptomics data for slice 1 in AnnData format
sc.h5ad: single cell data in the same tissue for slice 1 in AnnData format

I need you to help me plan what needs to be done in each step of the analysis process, and help me write the code for each step to install softwares, use tools and accomplish the following main goal:

Use Tangram to map single-cell data to spatial transcriptomics data.</td></tr>
<tr><td>Generated Results by CodeLlama-34B-Instruct</td><td>Assistant: Sure, I can help you with that! Here's a high-level plan for the analysis process:

1. Install necessary software:
* Install the necessary software for the analysis, including Tangram, by running the following command:

```
pip install tangram
```
2. Load the data:
* Load the spatial transcriptomics data from the `slice1.h5ad` file and the single-cell data from the `sc.h5ad` file using the `anndata` package.
3. Preprocess the data:
* Preprocess the data by filtering out low-quality cells and genes, normalizing the data, and transforming the data into a suitable format for Tangram.
4. Map the single-cell data to the spatial transcriptomics data:
* Use Tangram to map the single-cell data to the spatial transcriptomics data. This will create a new dataset that contains the spatial information for each cell.
5. Visualize the results:
* Visualize the results using a suitable visualization tool, such as Seurat or UCSC Genome Browser.

Here's some sample code to get you started:
```python
# Load the data
import anndata

slice1 = anndata.read_h5ad("slice1.h5ad")
sc = anndata.read_h5ad("sc.h5ad")

# Preprocess the data
from sklearn.preprocessing import StandardScaler

# Filter out low-quality cells and genes
slice1 = slice1[slice1.obs["quality"] > 0.5, :]</td></tr>
</table>

```
sc = sc[sc.obs["quality"] > 0.5, :]

# Normalize the data
scaler = StandardScaler()
slice1_scaled = scaler.fit_transform(slice1.X)
sc_scaled = scaler.fit_transform(sc.X)

# Transform the data into a suitable format for Tangram
from tangram import Tangram

tangram = Tangram(slice1_scaled, sc_scaled)

# Map the single-cell data to the spatial transcriptomics data
tangram.map_cells()

# Visualize the results
import seaborn as sns
import matplotlib.pyplot as plt

# Plot the spatial expression patterns for each gene
for gene in slice1.var.index:
    plt.figure(figsize=(10, 10))
    sns.heatmap(slice1.X[gene, :], cmap="Blues")
    plt.title(gene)
    plt.show()
```

This code assumes that the data is stored in the `slice1.h5ad` and `sc.h5ad` files, respectively. It also assumes that the data is in the AnnData format. If your data is in a different format, you may need to modify the code accordingly.

I hope this helps! Let me know if you have any questions or need further assistance.

Proteomics Case 20.1: protein expression quantification

**User's Inputs**	You are a bioinformatician who specializes in writing code and performing analysis for a variety of bioinformatic data analysis tasks. Now I provide you with an Ubuntu 18.04 system where I save the following data, each line including the data path and data description.<br><br>01CPTAC_COprospective_W_PNNL_20170123_B1S1_f01.mzML.gz: protein mass spectrometry data in mzML format, tumor samples fraction 1<br>01CPTAC_COprospective_W_PNNL_20170123_B1S1_f02.mzML.gz: protein mass spectrometry data in mzML format, tumor samples fraction 2<br>01CPTAC_COprospective_W_PNNL_20170123_B1S1_f03.mzML.gz: protein mass spectrometry data in mzML format, tumor samples fraction 3<br>98CPTAC_COprospective_W_VU_20160804_11CO047_f01.mzML.gz: protein mass spectrometry data in mzML format, normal samples fraction 1<br>98CPTAC_COprospective_W_VU_20160804_11CO047_f02.mzML.gz: protein mass spectrometry data in mzML format, normal samples fraction 2<br>98CPTAC_COprospective_W_VU_20160804_11CO047_f03.mzML.gz: protein mass spectrometry data in mzML format, normal samples fraction 3<br>UP000005640_9606.fasta: reference protein sequence from UniProt<br><br>I need you to help me plan what needs to be done in each step of the analysis process, and help me write the code for each step to install softwares, use tools and accomplish the following main goal:<br><br>identify proteins in tumor and normal samples based on mass spectrometry data
**Generated Results by CodeLlama-34B-Instruct**	Assistant: Sure, I'd be happy to help you with that! Here's a general plan for the analysis process:<br><br>1. Data preprocessing:<br>* Extract the protein sequences from the mzML files using a tool such as MS-GF+ or X!Tandem.<br>* Filter the protein sequences to remove any sequences that are not relevant to the analysis (e.g. contaminants, reverse hits).<br>* Map the filtered protein sequences to the reference protein database (e.g. UniProt) to identify known proteins.<br>2. Data analysis:<br>* Use a tool such as Percolator or MS-GF+ to perform protein inference and identify potential protein-peptide matches.<br>* Use a tool such as OpenMS or ProteoWizard to perform data processing and feature extraction.<br>* Use a tool such as R or Python to perform statistical analysis and data visualization.<br>3. Data visualization:<br>* Use a tool such as ggplot2 or matplotlib to create visualizations of the data, such as bar plots or scatter plots.<br>* Use a tool such as Tableau or Power BI to create interactive visualizations of the data.<br><br>Here's an example of how you could install and use some of the tools mentioned above on an Ubuntu 18.04 system:

1. Install MS-GF+:
```
sudo apt-get install ms-gf+
```
2. Install X!Tandem:
```
sudo apt-get install xtandem
```
3. Install Percolator:
```
sudo apt-get install percolator
```
4. Install OpenMS:
```
sudo apt-get install openms
```
5. Install ProteoWizard:
```
sudo apt-get install proteowizard
```
6. Install R:
```
sudo apt-get install r-base
```
7. Install Python:
```
sudo apt-get install python3
```
8. Install ggplot2:
```
sudo apt-get install r-cran-ggplot2
```
9. Install matplotlib:
```
sudo apt-get install python3-matplotlib
```
10. Install Tableau:
```
sudo apt-get install tableau
```
11. Install Power BI:
```
sudo apt-get install powerbi
```

I hope this helps! Let me know if you have any questions or need further assistance.

Metabolomics Case 21.1: metabolites concentration

**User's Inputs**	You are a bioinformatician who specializes in writing code and performing analysis for a variety of bioinformatic data analysis tasks. Now I provide you with an Ubuntu 18.04 system where I save the following data, each line including the data path and data description.<br><br>B1S1_f01.mzML.gz: LC-MS mass spectrometry data of metabolites in mzML format, tumor samples fraction 1<br>B1S1_f02.mzML.gz: LC-MS mass spectrometry data of metabolites in mzML format, tumor samples fraction 2<br>B1S1_f03.mzML.gz: LC-MS mass spectrometry data of metabolites in mzML format, tumor samples fraction 3<br>11CO047_f01.mzML.gz: LC-MS mass spectrometry data of metabolites in mzML format, normal samples fraction 1<br>11CO047_f02.mzML.gz: LC-MS mass spectrometry data of metabolites in mzML format, normal samples fraction 2<br>11CO047_f03.mzML.gz: LC-MS mass spectrometry data of metabolites in mzML format, normal samples fraction 3<br><br>I need you to help me plan what needs to be done in each step of the analysis process, and help me write the code for each step to install softwares, use tools and accomplish the following main goal:<br><br>calculate metabolites concentration in tumor and normal samples based on LC-MS mass spectrometry data
**Generated Results by CodeLlama-34B-Instruct**	Assistant: Sure, I can help you with that! Here's a step-by-step plan for the analysis process:<br><br>1. Install necessary software:<br>* Install the following software packages:<br>+ Python 3.x (e.g., Anaconda distribution)<br>+ R (e.g., RStudio)<br>+ MSConvert (from ProteoWizard)<br>+ MSGF+ (from the National Center for Biotechnology Information)<br>+ XCMS (from the University of California, San Diego)<br>+ R packages: "xcms", "xcmsTools", "xcmsPlot", "xcmsData", "xcmsUtils", "xcmsStats", "xcmsMetrics", "xcmsMassSpectrum", "xcmsPeakDetection", "xcmsPeakPicking", "xcmsPeakIntegration", "xcmsPeakAlignment", "xcmsPeakQuantification", "xcmsPeakIdentification", "xcmsPeakAnnotation", "xcmsPeakVisualization", "xcmsPeakClustering", "xcmsPeakSpectralMatching", "xcmsPeakSpectralAlignment", "xcmsPeakSpectralQuantification", "xcmsPeakSpectralVisualization", "xcmsPeakSpectralClustering", "xcmsPeakSpectralMatching", "xcmsPeakSpectralAlignment", "xcmsPeakSpectralQuantification", "xcmsPeakSpectralVisualization", "xcmsPeakSpectralClustering", "xcmsPeakSpectralMatching", "xcmsPeakSpectralAlignment", "xcmsPeakSpectralQuantification", "xcmsPeakSpectralVisualization", "xcmsPeakSpectralClustering", "xcmsPeakSpectralMatching", "xcmsPeakSpectralAlignment", "xcmsPeakSpectralQuantification", "xcmsPeakSpectralVisualization", "xcmsPeakSpectralClustering", "xcmsPeakSpectralMatching",

	"xcmsPeakSpectralAlignment", "xcmsPeakSpectralQuantification",<br>"xcmsPeakSpectralVisualization", "xcmsPeakSpectralClustering",<br>"xcmsPeakSpectralMatching", "xcmsPeakSpectralAlignment",<br>"xcmsPeakSpectralQuantification", "xcmsPeakSpectralVisualization",<br>"xcmsPeakSpectralClustering", "xcmsPeakSpectralMatching",<br>"xcmsPeakSpectralAlignment", "xcmsPeakSpectralQuantification",<br>"xcmsPeakSpectralVisualization", "xcmsPeakSpectralClustering",<br>"xcmsPeakSpectralMatching", "xcmsPeakSpectralAlignment",<br>"xcmsPeakSpectralQuantification", "xcmsPeakSpectralVisualization",<br>"xcmsPeakSpectralClustering", "xcmsPeakSpectralMatching",<br>"xcmsPeakSpectralAlignment", "xcmsPeakSpectralQuantification",<br>"xcmsPeakSpectralVisualization", "xcmsPeakSpectralClustering",<br>"xcmsPeakSpectralMatching", "xcmsPeakSpectralAlignment",<br>"xcmsPeakSpectralQuantification", "xcmsPeakSpectralVisualization",<br>"xcmsPeakSpectralClustering", "xcmsPeakSpectralMatching",<br>"xcmsPeakSpectralAlignment", "xcmsPeakSpectralQuantification",<br>"xcmsPeakSpectralVisualization", "xcmsPeakSpectralClustering",<br>"xcmsPeakSpectralMatching", "xcmsPeakSpectralAlignment",<br>"xcmsPeakSpectralQuantification", "xcmsPeakSpectralVisualization",<br>"xcmsPeakSpectralClustering", "xcmsPeakSpectralMatching",<br>"xcmsPeakSpectralAlignment", "xcmsPeakSpectralQuantification",<br>"xcmsPeakSpectralVisualization", "xcmsPeakSpectralClustering",<br>"xcmsPeakSpectralMatching", "xcmsPeakSpectralAlignment",<br>"xcmsPeakSpectralQuantification", "xcmsPeakSpectralVisualization",<br>"xcmsPeakSpectralClustering", "xcmsPeakSpectralMatching",<br>"xcmsPeakSpectralAlignment", "xcmsPeakSpectralQuantification",<br>"xcmsPeakSpectralVisualization", "xcmsPeakSpectralClustering",<br>"xcmsPeakSpectralMatching", "xcmsPeakSpectralAlignment",<br>"xcmsPeakSpectralQuantification", "xcmsPeakSpectralVisualization",<br>"xcmsPeakSpectralClustering", "xcmsPeakSpectralMatching",<br>"xcmsPeakSpectralAlignment", "xcmsPeakSpectralQuantification",<br>"xcmsPeakSpectralVisualization", "xcmsPeakSpectralClustering",<br>"xcmsPeakSpectralMatching", "xcmsPeakSpectralAlignment",<br>"xcmsPeakSpectralQuantification", "xcmsPeakSpectralVisualization",<br>"xcmsPeakSpectralClustering", "xcmsPeakSpectralMatching",<br>"xcmsPeakSpectralAlignment", "xcmsPeakSpectralQuantification",<br>"xcmsPeakSpectralVisualization", "xcmsPeakSpectralClustering",<br>"xcmsPeakSpectralMatching", "xcmsPeakSpectralAlignment",<br>"xcmsPeakSpectralQuantification", "xcmsPeakSpectralVisualization",<br>"xcmsPeakSpectralClustering", "xcmsPeakSpectralMatching",<br>"xcmsPeakSpectralAlignment", "xcmsPeakSpectralQuantification",<br>"xcmsPeakSpectralVisualization", "xcmsPeakSpectralClustering",<br>"xcmsPeakSpectralMatching", "xcmsPeakSpectralAlignment",<br>"xcmsPeakSpectralQuantification", "xcmsPeakSpectralVisualization",<br>"xcmsPeakSpectralClustering", "xcmsPeakSpectralMatching",<br>"xcmsPeakSpectralAlignment", "xcmsPeakSpectralQuantification",<br>"xcmsPeakSpectralVisualization", "xcmsPeakSpectralClustering",<br>"xcmsPeakSpectralMatching", "xcmsPeakSpectralAlignment",<br>"xcmsPeakSpectralQuantification", "xcmsPeakSpectralVisualization",<br>"xcmsPeakSpectralClustering", "xcmsPeakSpectralMatching",<br>"xcmsPeakSpectralAlignment", "xcmsPeakSpectralQuantification",<br>"xcmsPeakSpectralVisualization", "xcmsPeakSpectralClustering",<br>"xcmsPeakSpectralMatching", "xcmsPeakSpectralAlignment",

	"xcmsPeakSpectralQuantification", "xcmsPeakSpectralVisualization",<br>"xcmsPeakSpectralClustering", "xcmsPeakSpectralMatching",<br>"xcmsPeakSpectralAlignment", "xcmsPeakSpectralQuantification",<br>"xcmsPeakSpectralVisualization", "xcmsPeakSpectralClustering",<br>"xcmsPeakSpectralMatching", "xcmsPeakSpectralAlignment",<br>"xcmsPeakSpectralQuantification", "xcmsPeakSpectralVisualization",<br>"xcmsPeakSpectralClustering", "xcmsPeakSpectralMatching",<br>"xcmsPeakSpectralAlignment", "xcmsPeakSpectralQuantification",<br>"xcmsPeakSpectralVisualization", "xcmsPeakSpectralClustering",<br>"xcmsPeakSpectralMatching", "xcmsPeakSpectralAlignment",<br>"xcmsPeakSpectralQuantification", "xcmsPeakSpectralVisualization",<br>"xcmsPeakSpectralClustering", "xcmsPeakSpectralMatching",<br>"xcmsPeakSpectralAlignment", "xcmsPeakSpectralQuantification",<br>"xcmsPeakSpectralVisualization", "xcmsPeakSpectralClustering",<br>"xcmsPeakSpectralMatching", "xcmsPeakSpectralAlignment",<br>"xcmsPeakSpectralQuantification", "xcmsPeakSpectralVisualization",<br>"xcmsPeakSpectralClustering", "xcmsPeakSpectralMatching",<br>"xcmsPeakSpectralAlignment", "xcmsPeakSpectralQuantification",<br>"xcmsPeakSpectralVisualization", "xcmsPeakSpectralClustering",<br>"xcmsPeakSpectralMatching", "xcmsPeakSpectralAlignment",<br>"xcmsPeakSpectralQuantification", "xcmsPeakSpectralVisualization",<br>"xcmsPeakSpectralClustering", "xcmsPeakSpectralMatching",<br>"xcmsPeakSpectralAlignment", "xcmsPeakSpectralQuantification",<br>"xcmsPeakSpectralVisualization", "xcmsPeakSpectralClustering",<br>"xcmsPeakSpectralMatching", "xcmsPeakSpectralAlignment",<br>"xcmsPeakSpectralQuantification", "xcmsPeakSpectralVisualization",<br>"xcmsPeakSpectralClustering", "xcmsPeakSpectralMatching",<br>"xcmsPeakSpectralAlignment", "xcmsPeakSpectralQuantification",<br>"xcmsPeakSpectralVisualization", "xcmsPeakSpectralClustering",<br>"xcmsPeakSpectralMatching", "xcmsPeakSpectralAlignment",<br>"xcmsPeakSpectralQuantification", "xcmsPeakSpectralVisualization",<br>"xcmsPeakSpectralClustering", "xcmsPeakSpectralMatching",<br>"xcmsPeakSpectralAlignment", "xcmsPeakSpectralQuantification",<br>"xcmsPeakSpectralVisualization", "xcmsPeakSpectralClustering",<br>"xcmsPeakSpectralMatching", "xcmsPeakSpectralAlignment",<br>"xcmsPeakSpectralQuantification", "xcmsPeakSpectralVisualization",<br>"xcmsPeakSpectralClustering", "xcmsPeakSpectralMatching",<br>"xcmsPeakSpectralAlignment", "xcmsPeakSpectralQuantification",<br>"xcmsPeakSpectralVisualization", "xcmsPeakSpectralClustering",<br>"xcmsPeakSpectralMatching", "xcmsPeakSpectralAlignment",<br>"xcmsPeakSpectralQuantification", "xcmsPeakSpectralVisualization",<br>"xcmsPeakSpectralClustering", "xcmsPeakSpectralMatching",<br>"xcmsPeakSpectralAlignment", "xcmsPeakSpectralQuantification",<br>"xcmsPeakSpectralVisualization", "xcmsPeakSpectralClustering",<br>"xcmsPeakSpectralMatching", "xcmsPeakSpectralAlignment",<br>"xcmsPeakSpectralQuantification", "xcmsPeakSpectralVisualization",<br>"xcmsPeakSpectralClustering", "xcmsPeakSpectralMatching",<br>"xcmsPeakSpectralAlignment", "xcmsPeakSpectralQuantification",<br>"xcmsPeakSpectralVisualization", "xcmsPeakSpectralClustering",<br>"xcmsPeakSpectralMatching", "xcmsPeakSpectralAlignment",<br>"xcmsPeakSpectralQuantification", "xcmsPeakSpectralVisualization",<br>"xcmsPeakSpectralClustering", "xcmsPeakSpectralMatching",<br>"xcmsPeakSpectralAlignment", "xcmsPeakSpectralQuantification",

	"xcmsPeakSpectralVisualization", "xcmsPeakSpectralClustering",<br>"xcmsPeakSpectralMatching", "xcmsPeakSpectralAlignment",<br>"xcmsPeakSpectralQuantification", "xcmsPeakSpectralVisualization",<br>"xcmsPeakSpectralClustering", "xcmsPeakSpectralMatching",<br>"xcmsPeakSpectralAlignment", "xcmsPeakSpectralQuantification",<br>"xcmsPeakSpectralVisualization", "xcmsPeakSpectralClustering",<br>"xcmsPeakSpectralMatching", "xcmsPeakSpectralAlignment",<br>"xcmsPeakSpectralQuantification", "xcmsPeakSpectralVisualization",<br>"xcmsPeakSpectralClustering", "xcmsPeakSpectralMatching",<br>"xcmsPeakSpectralAlignment", "xcmsPeakSpectralQuantification",<br>"xcmsPeakSpectralVisualization", "xcmsPeakSpectralClustering",<br>"xcmsPeakSpectralMatching", "xcmsPeakSpectralAlignment",<br>"xcmsPeakSpectralQuantification", "xcmsPeakSpectralVisualization",<br>"xcmsPeakSpectralClustering", "xcmsPeakSpectralMatching",<br>"xcmsPeakSpectralAlignment", "xcmsPeakSpectralQuantification",<br>"xcmsPeakSpectralVisualization", "xcmsPeakSpectralClustering",<br>"xcmsPeakSpectralMatching", "xcmsPeakSpectralAlignment",<br>"xcmsPeakSpectralQuantification", "xcmsPeakSpectralVisualization",<br>"xcmsPeakSpectralClustering", "xcmsPeakSpectralMatching",<br>"xcmsPeakSpectralAlignment", "xcmsPeakSpectralQuantification",<br>"xcmsPeakSpectralVisualization", "xcmsPeakSpectralClustering",<br>"xcmsPeakSpectralMatching", "xcmsPeakSpectralAlignment",<br>"xcmsPeakSpectralQuantification", "xcmsPeakSpectralVisualization",<br>"xcmsPeakSpectralClustering", "xcmsPeakSpectralMatching",<br>"xcmsPeakSpectralAlignment", "xcmsPeakSpectralQuantification",<br>"xcmsPeakSpectralVisualization", "xcmsPeakSpectralClustering",<br>"xcmsPeakSpectralMatching", "xcmsPeakSpectralAlignment",<br>"xcmsPeakSpectralQuantification", "xcmsPeakSpectralVisualization",<br>"xcmsPeakSpectralClustering", "xcmsPeakSpectralMatching",<br>"xcmsPeakSpectralAlignment", "xcmsPeakSpectralQuantification",<br>"xcmsPeakSpectralVisualization", "xcmsPeakSpectralClustering",<br>"xcmsPeakSpectralMatching", "xcmsPeakSpectralAlignment",<br>"xcmsPeakSpectralQuantification", "xcmsPeakSpectralVisualization",<br>"xcmsPeakSpectralClustering", "xcmsPeakSpectralMatching",<br>"xcmsPeakSpectralAlignment", "xcmsPeakSpectralQuantification",<br>"xcmsPeakSpectralVisualization", "xcmsPeakS--------------
